# Large molecular systems landscape uncovers T cell trapping in human skin cancer

**DOI:** 10.1038/srep19012

**Published:** 2016-01-13

**Authors:** Reyk Hillert, Anne Gieseler, Andreas Krusche, Daniel Humme, Hans-Joachim Röwert-Huber, Wolfram Sterry, Peter Walden, Walter Schubert

**Affiliations:** 1 Molecular Pattern Recognition Research Group, Medical Faculty, OvG University Magdeburg, Leipziger Straße 44, D-39120 Magdeburg, Germany; 2 Human Toponome Project (HUTO), Margaretenstraße 20, D-81373 Munich, Germany; 3 Department of Dermatology, Venerology and Allergology, Charité – Universitätsmedizin Berlin, Charitéplatz 1, D-10117 Berlin, Germany; 4 Department for Structural Cell Biology, Center for Biological Systems Analysis (ZBSA), Albert-Ludwigs-University,Freiburg, Habsburger Straße 49, D-79104 Freiburg,Germany

## Abstract

Immune surveillance of tumour cells is an important function of CD8 T lymphocytes, which has failed in cancer for reasons still unknown in many respect but mainly related to cellular processes in the tumour microenvironment. Applying imaging cycler microscopy to analyse the immune contexture in a human skin cancer we could identify and map 7,000 distinct cell surface-associated multi-protein assemblies. The resulting combinatorial geometry-based high-functional resolution led to discovery of a mechanism of T cell trapping in the epidermis, which involves SPIKE, a network of suprabasal keratinocyte projections piercing and interconnecting CD8 T cells. It appears initiated by clusters of infrabasal T and dendritic cells connected via cell projections across a fractured basal lamina to suprabasal keratinocytes and T lymphocytes.

T lymphocytes have important functions in the immune-mediated control of tumour cells^1^,^2^,^3^,^4^,^5^,^6^,^7^,^8^,^9^,^10^,^11^,^12^,^13^,^14^,^15^,^16^,^17^ but in manifest cancer they appear incapacitated within the tumour microenvironment for, in many aspects, still elusive reasons^1^,^16^,^17^,^18^,^19^,^20^. Besides T cells and cancer cells, the tumour microenvironment comprises other cellular and non-cellular components such as cells of mesenchymal origin and molecules of the extracellular matrix that all influence course and outcome of the malignancies^21^. Over the past two decades a wealth of information has been acquired on various factors that may interfere with effective anti-tumour immune responses such as Tregs, cytokines, tumour matrix, immunological checkpoint receptors (PD-1, CTLA-4) and others^22^. Nonetheless, the highly diverse and varied interactions of the components in the tumour microenvironment that often support cancer development are in major aspects not understood. Such lack of understanding may in parts explain the high failure rates of new drugs^23^ targeting one or several components of the microenvironment. Like other biological systems^24^, the tumour microenvironment appears robust and is not easily upset as long as the critical interactions and corresponding nodes of robustness are not targeted and inactivated. The high attrition rate of anti cancer drugs^23^ suggests that pharmaceutical development guided by model studies does not sufficiently reflect the disease processes inside human tissues. This emphasizes the need for methods for the detection and analysis of disease mechanisms directly *in situ*. As discussed previously^25^,^26^, information on the spatiotemporal arrangements of molecules and cells in tissues are required for proper diagnosis and essential for an understanding the pathological processes.

To address directly the topology of multi-component systems in human skin cancer tissue, we employed imaging cycler microscopy (ICM)^25^,^26^ to analyse the immune contexture in skin tissue of the cutaneous CD4 T cell lymphoma *Mycosis fungoides* (for details of the clinicopathological features see Supplementary Figure 1). ICM is an automated technique that runs repetitive cycles of fluorescence labelling of biomolecules followed by imaging and bleaching *in situ*, and is capable of co-mapping the hierarchical combinatorial structure of many thousand distinct biomolecular assemblies, termed the toponome^27^. Toponome-based search for biomarkers and drug targets with the identification of lead proteins that control protein network topology and function *in situ*^27^ has been validated experimentally^28^ and clinically^29^,^30^. Here we used ICM to address directly the topology of multi-component systems in the human skin cancer *Mycosis fungoides*, a cutaneaous CD4 T cell lymphoma. ICM-based combinatorial geometry of more than 7,000 distinct cell surface-associated biomolecular assemblies disclosed a hitherto unknown multicellular structure, which we then dissected spatially using a real-time virtual anatomical slicing technique. This approach revealed direct insight into an unexpected structure and mechanism of mechanical trapping of suprabasal non-tumour CD8 T cells through keratinocyte-mediated piercing, interconnecting and thus fixing these cells with infrabasal cells in the dermis. This entire multicellular assembly constitutes a systems-robustness node protecting the tumour from cytotoxic T cell attack.

## Results

*Mycosis fungoides* (MF) is a non-Hodgkin T cell lymphoma in human skin of unknown aetiology that mostly, as in the case studied here, involves fully differentiated malignant CD4 T cells^31^ (Supplementary Figure 1). To understand the immune mechanisms in this disease and the complex cellular interactions in the tumour microenvironment outside the CD4 tumour cell clusters we applied parameter-unlimited ICM^25^,^26^ for dissecting cell surface-associated molecular systems likely to provide insight into cellular interaction patterns in the tumour tissue. ICM was performed with a robotic system programmed to run repetitive cycles of staining, imaging and bleaching of a FITC-conjugated tag library (for the mapped 25 distinct biomolecules see Supplementary Table 1) to collect z-stack images of every detected protein of a MF tissue section placed on the stage of the ICM epifluorescence scanning table^32^ (see methods section). The resulting combinatorial molecular phenotypes (CMPs) per voxel were assembled as frequency matrix (Supplementary Table 2 and 3) sorted by motifs with lead proteins present in all CMPs of the respective motif, and then mapped to and visualized at their tissue locations (exemplified in Supplementary Figure 2) as previously described^32^. In all, we found motifs together comprising 7,161 CMPs (Supplementary Table 2).

**Figure 1:**
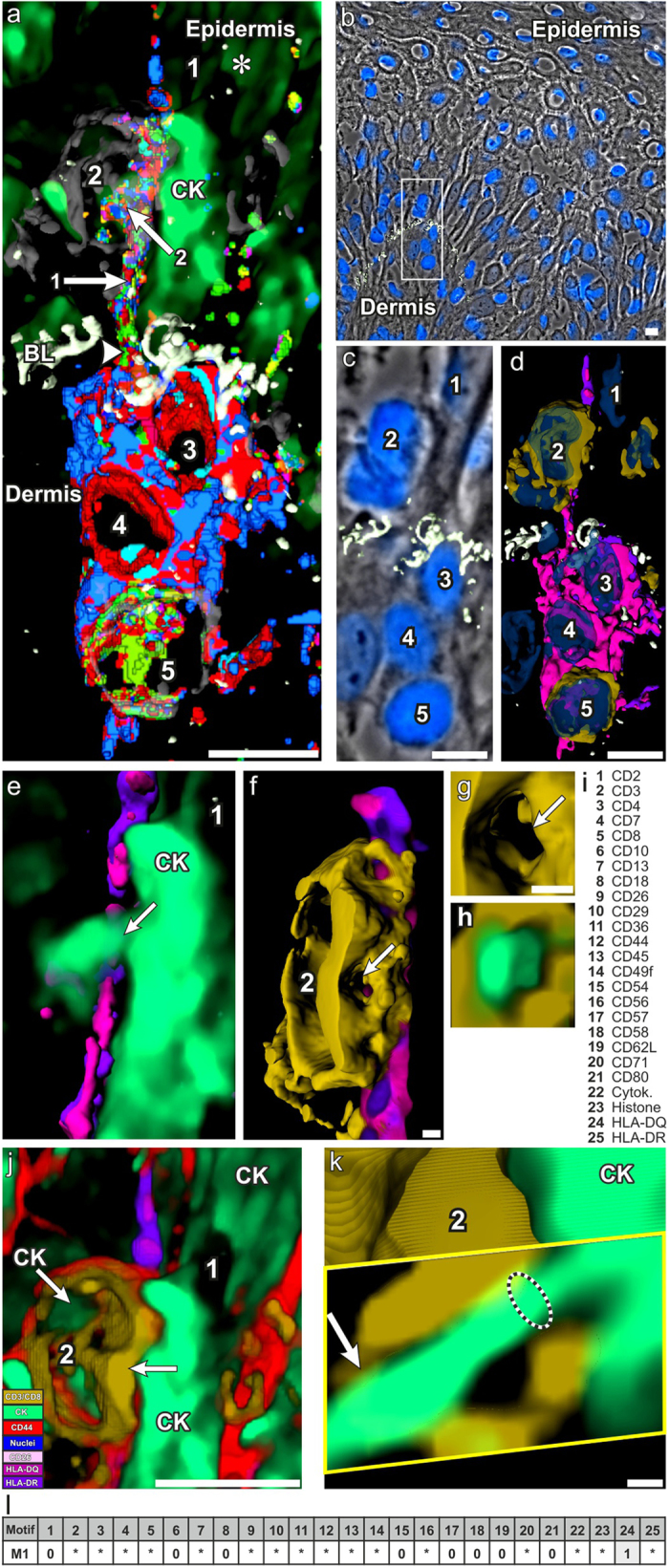
Tissue organisation of SPIKE. **(a)** 3D co-mapping of 3,213 CMPs (out of 7,161 CMPs) within an area of a MF skin tissue cryo-section (**b)** boxed area. Distinct CMPs are visualised by different colors. (**b**) phase contrast image of a cryo-preserved skin section of MF, in which nuclei were stained in blue for histone and the basal lamina in white for CD49f^24^,^25^ (**c**) magnification of the boxed area of (**b**) in direct alignment with (**d**) locating the lead proteins of the CMPs shown in (**a**) uncovering SPIKE as an elongated multicellular arrangement of five cell types (Cells 1 to 5 in **a,d,c**). A partial list of color decoding is given in Supplementary Fig. 2; the complete CMP list in provided in Supplementary Table 2). A long cell projection (arrow 1 in **a**) of Cells 3,4,5 extends from the dermis across the basal lamina (**a**, BL) into the epidermis where it is closely opposed to a CD8^+^CD3^+^ T cell (Cell 2 in **a**,**c**,**d**; brown color in **d**) and a neighboring keratinocyte (Cell 1 in **a**,**c**,**d**). (**e**) details of the suprabasal part of this structure pictured from different angles (**e**,**f**,**g**,**h**). A cytokeratin-containing cell projection (CK) extending from the keratinocyte (**e**, arrow) (Cell 1) projects through the CD3^+^CD8^+^ T cell surface (compare (**h)** showing CK in green and (**g**) the same without CK). (**k**) transverse virtual anatomical slicing across the CD8^+^CD3^+^ T cell (Cell 2 in **j**). Keratinocyte projection (CK) penetrates the T cell interior (arrow). **(i**) list of the co-mapped molecules (detailed in Supplementary Table 1). (**l**) CMP motifs specific for SPIKE as a whole (Motif M1) (for details see the complete functional CMP linkage map in Supplementary Table 2). Supplementary Video 1 illustrates the active process of spatial dissection of the SPIKE´s structures. Bars: in (**a–d,j)**: 10 μm; in (**f**,**g**,**k)**: 1 μm.

To investigate the CMPs directly in their tissue context we followed a systems-biological top down approach^33^ from transcellular to subcellular visualization of tissue features, applying stepwise visualization of all or fractions of the CMPs as combinatorial geometric structures. We then applied virtual anatomical sectioning guided by the discovered geometric structures^26^. In a first step, we extracted the most prominent proteins, lead proteins^25^, from the identified CMPs. Then we visualized the locations of the corresponding CMPs and their lead proteins simultaneously at 3D, exemplified for 3,213 CMPs in Fig. 1a,d, respectively (Supplementary Table 3). The colours are partially decoded in Supplementary Figure 2. The most prominent lead proteins were extracted and co-visualized directly in the frozen skin tissue section of MF (Fig. 1b,c,d respectively). This finally exposed the molecular details of cellular interactions and disease-specific CMP arrangements (Fig. 1e–k) (Supplementary Video 1).

As result, we identified a multicellular assembly of five cell types (Fig. 1a; Cells 1 through 5), three located in the dermis (Fig. 1a, Cells 3 to 5), two in the epidermis (Fig. 1a, Cells 1 and 2). The cells are interconnected by a cell projection extending from the dermal cell cluster to the cells 1 and 2 in the epidermis, penetrating the basal lamina (BL) (Fig. 1a, arrowhead), an extracellular matrix structure naturally separating dermis from epidermis, which in this case of MF, however, appears distorted. Henceforth we call this entire multicellular structure SPIKE (a multicellular apparatus spiking the BL). Major aspects of SPIKE (Cells 3,4,5 plus the trans-BL cell extension) are resolved by the composition of cell surface-associated CMPs, which appear to be building blocks or coherent distinctive multi-molecular components of SPIKE´s cell surface organization (Fig. 1a, partial colour decoding list in Supplementary Figure 1). These CMPs have HLA-DQ as common lead protein combined with other, variably co-mapped cell surface proteins (Fig. 1i) while others are always absent. Together this defines a CMP motif, a three symbol code with a lead protein (LP), variably associated proteins (wild cards), and absent proteins (0)^25^,^26^,^32^. In respect to SPIKE, Motif 1 (M1, Fig. 1l) selectively characterizes the SPIKE structure as visualized in Fig. 1d.

**Figure 2:**
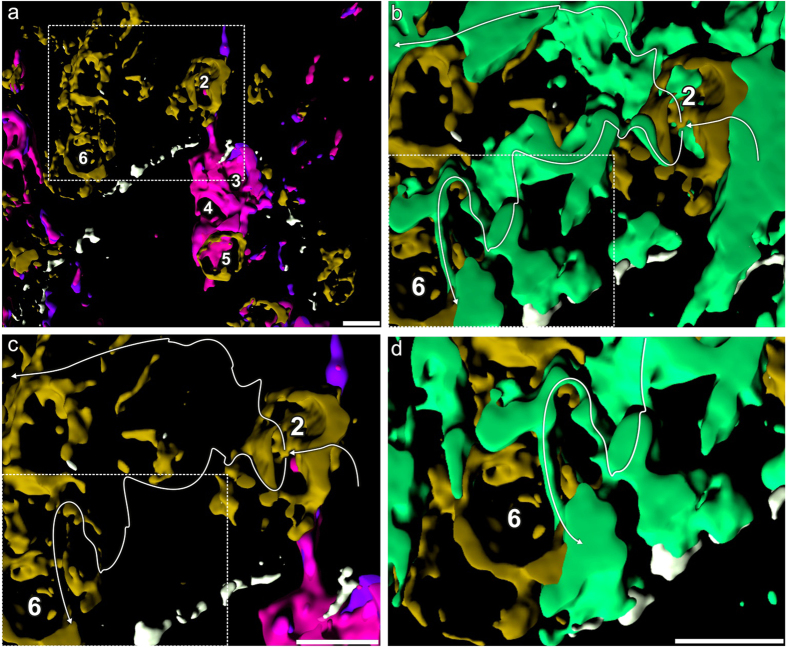
Crosslinking of suprabasal non-tumour CD8 T cells by keratinocyte extensions. (**a)** Overview of the wider area of the section detailed in Fig. 1. **(b**) magnification of the boxed area in **a**. The surface-rendered cytokeratin network of keratinocyte extensions are displayed in green, the T cells 2 and 6 in brown. The white line traces the keratinocyte extensions through the T cells. (**c**) same area as in **b** with display of the T cells in brown and the trace of the keratinocyte extensions as white line showing how the T cells are crosslinked by the extensions. (**d**) magnification of boxed area in (**b)** showing details of the penetrated CD3^+^CD8^+^ T cell 6. Note that the CK-positive network penetrating and crosslinking T cells originates at the suprabasal site of SPIKE´s multicellular assembly (**a**, Cell 2) as illustrated in Fig. 1. Bars: 10 μm.

Prominent feature of SPIKE is the presence of a large number of CMPs expressed along the supra-basal SPIKE´s cell extension (Fig. 1a, arrow 1) compared to more extended spatial distributions of only few CMPs in the dermal cell components of SPIKE (Fig. 1a, cells 3 to 5). The suprabasal SPIKE cell extension projects between a cytokeratin-expressing epidermal cell (keratinocyte) (Fig. 1a, CK, asterisk, Cell 1) and a CD3^+^CD8^+^ non-tumour T cell (Fig. 1a, cell 2) apparently bridging the cell surfaces of these two cells. This is supported by the spatial interrelationship of SPIKE’s cell extension (Fig. 1a, arrow 1), the keratinocyte (Fig. 1a, cell 1) and the CD3^+^CD8^+^ T cell (Fig. 1a, cell 2). T cells of the latter type are known to infiltrate MF tissue and exhibit anti-tumour cytotoxic activity^34^ but the ultimate clinical outcome suggests blocking mechanisms inside the tumour tissue^31^. By analyzing the contact sites of the different cell types as visualized by the combinatorial geometric structure (Fig. 1a) with the suprabasal cell types (Fig. 1, Cell 2), we identified a CMP structure apparently projecting from the cell extension (Fig. 1a, arrow 1) into the CD3^+^CD8^+^ T cell (Fig. 1a, arrow 2). We analyzed the interaction site in more detail by first extracting the lead protein HLA-DQ from Motif 1 (see above) and displaying it three-dimensionally (Fig. 1d, magenta) together with the CD3^+^CD8^+^ T cell (Fig. 1d, Cell 2, brown). This interaction site corresponds to the combinatorial geometry CMP structure in Fig. 1a. Both modes of visualizing SPIKE were aligned with the corresponding site in the phase-contrast image of the cryosection (Fig. 1b, boxed area, magnified in 1c). Next, we further inspected the interaction site shown in Fig. 1 (arrow 2) by visualizing the details in the tilted image of the structure (Fig. 1d, Cell 2) as shown in Fig. 1f,g,h. This revealed that Cell 2 (Fig. 1f) co-localizes with a cytokeratin-containing extension of the neighboring keratinocyte (Fig. 1e, arrow 1, CK). In addition, these analyses revealed a roundish area devoid of CD3 and CD8 (Fig. 1f,g, arrow) within the CD3^+^CD8^+^ T cell surface (Fig. 1f,g, brown color), leaving open whether this was due to circumscribed absence of CD3/CD8 or to a physical defect of the T cell membrane, or another structural abnormality of the cell surface. To decide between these possibilities we applied virtual anatomical sectioning across the region and found a long cytokeratin-containing extension of the keratinocyte projecting (Fig. 1e, arrow) into the CD3/CD8-devoid T cell surface area (Fig. 1g arrow, h) where it extends into the T cell (Fig. 1k arrow). Figure 1(e) gives an overview of the interaction site visualizing only the most prominent proteins. Following the route of the cytokeratin projection (Fig. 1k, arrow), we identified an extended ramified network of cytokeratin-containing projections penetrating and crossing suprabasal CD8^+^CD3^+^ T cells (Fig. 2) apparently fixing them mechanically in the suprabasal region of the skin. The traces of the keratinocyte projection cross-connecting the T cells are highlighted with white lines in Fig. 2a–d. Given the strictly intracellular location of cytokeratins in epidermal cells, it is likely that the cytokeratin-containing projections are ensheathed by cell membrane. With the tag library used in this study, we were unable to identify this membrane; this notwithstanding the topological correlation of cytokeratin with epidermal cell structures shown in (Fig. 1e, Cell 1; k) strongly suggests that intracellular cytokeratin is just a marker for an extended network of epidermal cell membrane projections.

**Figure 3:**
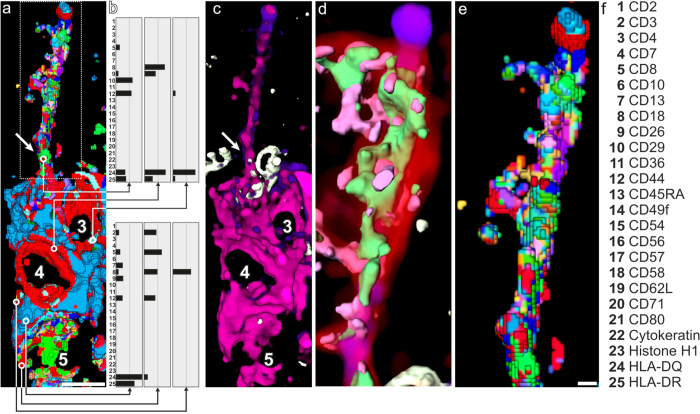
Supramolecular organisation of SPIKE. SPIKE as in Fig. 1a is depicted without the suprabasal Cells 1 and 2 to dissect its supramolecular cell surface organization by direct alignment of different methods. (**a)** 3D **s**tructure of SPIKE as seen by co-mapping 3,213 distinct CMPs listed in Supplementary Table 3 and displayed in colors in part decoded in Supplementary Figure 2. (**b**) protein profiling by real-time similarity mapping (Supplementary Video 2) shows that Cell 5 has a variable CD8^+^ T cell profile while Cells 3 and 4 display variable HLA-DQ^+^ profiles. Note that the expression profile of the cell extension (**a**, arrow) at the site where it penetrates the BL (compare with **c**, arrow) is different from that of the Cell 3. (**c**) Semitransparent volume-rendered SPIKE showing a co-map of HLA-DQ (magenta) and HLA-DR (violet). The basal lamina is marked by CD49f (white); (**d**) detail of the suprabasal section of the cell extension displaying overlaid expression of volume-rendered HLA-DQ/HLA-DR (transparent, colors as in **c**) together with CD29 in green, CD26 in pink and direct overlays of these molecules with CD44 in red; (**e**) magnification of the cell extension shown in (**a**) boxed area displaying its supramolecular CMP cell surface structure. The direct real time decoding is shown in Supplementary Video 3; (**f)** list of co-mapped biomolecules (detailed in Supplementary Table 1). Bars in **a**, **c**:10 μm; in **d**, **e**: 1 μm

Through the network of keratinocyte projections, the infiltrating T cells, many likely anti-tumour T cells, may be mechanically trapped at the suprabasal sites *in situ*. This process is associated with and appears to be mediated by the BL-penetrating cell extension of SPIKE. This hypothesis is supported by ICM data directly aligned as (i) threshold-based CMPs (Fig. 3a,e), (ii) semitransparent 3D supramolecular assembly imaging (Fig. 3c,d), and (iii) non-threshold based multi-protein profiling using real-time similarity mapping^35^,^36^ (Fig. 3b) (Supplementary Video 2). This synopsis reveals that the supramolecular organization of multicellular SPIKE is bipartite. While the dermal part expresses a CD8 T cell motif in Cell 5 (Fig. 3a,b, Cell 5) and a HLA-DQ motif in Cells 3 and 4 (Fig. 3a, cells 3 and 4), only the HLA-DQ motif, not the CD8 motif (cell 5), is continued into the suprabasal cell extension of SPIKE (Fig. 3e, partial color decoding list in Supplementary Figure 2). The large number of distinct CMPs on SPIKE´s suprabasal cell extension indicates a highly differentiated supramolecular organization at the cell surface membrane, apparently directly controlling the suprabasal cellular pathology.

Having SPIKE and the arrangement of the cells around SPIKE identified (A subbasal cluster of two CD8^+^ T cells and a dendritic cell-like cell extending SPIKE across the BL to a suprabasal cluster of a CD8^+^ T cell and a keratinocyte that fixes this T cell plus up to four additional ones with membrane extensions.) we could demonstrate that this arrangement is recurring throughout the MF plaque at about 100 μm intervals.The recurrences appear nearly identical to the example presented herein. Re-inspecting the toponome analyses published earlier for psoriasis and atopic dermatitis, two pathological conditions mediated by T cells, and healthy skin^25^, we found no evidence for SPIKE and the associated cell arrangement. These structures appear to be specific for the MF plaque lesion.

## Discussion

**Figure 4:**
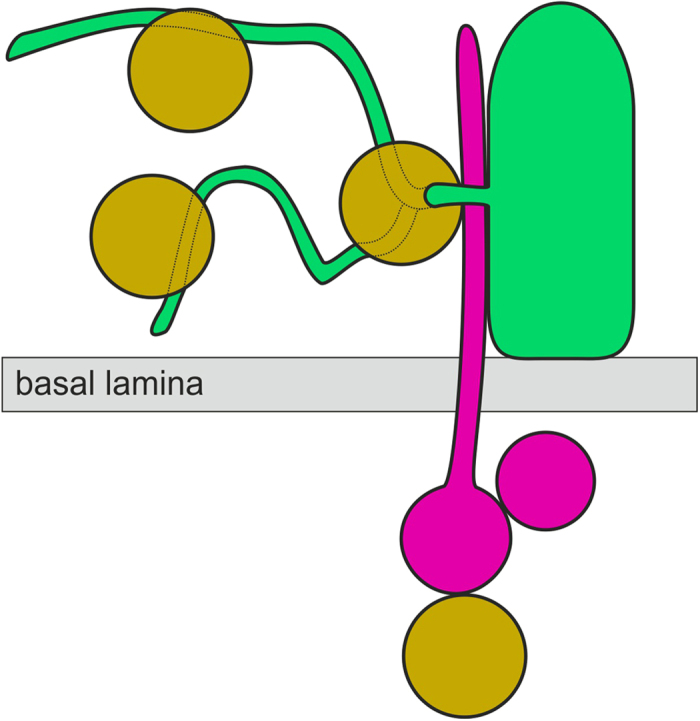
Model for SPIKE. Multicellular assembly in the dermis projects a long cell extension across the basal lamina into the epidermis where it interacts with an infiltrating non tumour CD8 T cell and a neighboring keratinocyte. This interaction induces keratinocyte projections penetrating and crosslinking CD8^+^ T cells, thereby mechanically preventing them from (anti-tumour) action. Brown: CD8^+^CD3^+^, green: cytokeratin; magenta: HLA-DQ as markers for the interacting cells.

We suggest that the whole SPIKE motif shown here is expression of a supramolecular cell surface machinery enabling SPIKE to efficiently penetrate the BL barrier and mediate T cell piercing by suprabasal keratinocyte projections. This machinery may be an efficient target for therapeutic intervention. The CD8 motif-expressing non-tumour T cell in the dermis (Fig. 3, Cell 5) is obviously not participating in the suprabasal cell extension of SPIKE, although it may be an important component of the overall SPIKE structure and function. We have not observed any similar structure in normal skin or inflammatory skin conditions^25^. Together these findings suggest that SPIKE is a higher-order disease-specific multicellular unit in MF that mechanically connects epidermis and dermis (scheme Fig. 4). Mechanical entrapment of large numbers of T cells in MF skin may contribute to loss of local immune capacities^31^. Our findings demonstrate a high dimension of pathological cell interactions in cancer, here MF. They show the complex immune contexture within the extended cancer tissue^37^ detected and dissected by direct microscopic *in situ* analyses with supramolecular to transcellular resolution for many cell types and molecular components simultaneously. ICM and combinatorial geometry-based highly resolved identification of molecular networks and molecular nods in these networks may be exploited further for the identification of systems-based drug targets. The potential efficacy of the successful blockade of such systems-based targets as therapeutic principle has been demonstrated earlier in other diseases^25^.

In these studies, ICM has revealed that chronic diseases, when analyzed for many distinct cell surface-associated protein assemblies in parallel, have a unique supramolecular order *in situ* with protein hierarchies in which lead proteins control spatial protein network topology and function. As shown before, inhibition of the lead proteins, e.g. in tumour cells results in disassembly of the corresponding protein clusters and loss of function^25^. Similar principles were demonstrated for amyothrophic lateral sclerosis (ALS) where CD16 (Fcgamma RIII) on human peripheral lymphocytes was identified as lead drug target^38^ This protein was then confirmed as potential ALS drug target in a FcgammaRIII KO mouse model^28^ and recently in a clinical phase II trial showing well tolerated downregulation of CD16 in ALS patients^29^ with a halt of disease progression in 27% of the patients^30^. This empirical verification supports the toponome hypothesis that lead proteins controlling *in vivo* protein network topology and (dys)function can be predictive drug targets in chronic diseases and that the combinatorial geometry of protein networks at the target sites of the disease, as shown in present study, can provide important functional information on disease mechanisms.

ICM-based target identification and decoding of disease mechanisms *in situ* may thus complement current strategies for discovery of checkpoint controls as therapeutic target for reconstitution or enhancement of T cell activity against cancer^2^,^6^,^7^,^15^,^39^,^40^,^41^,^42^,^43^. The T cell trapping machinery may be a disease-specific robustness node for MF that ensures cancer progression by massively blocking cytotoxic T cells. As such it may be a target for therapeutic interventions that restore efficient anti-tumour immune responses.

## Material and Methods

### Skin tissue sections

The protocol for the reported study had been carried out in strict accordance with the approved guidelines of the Institutional Ethics Committee of the Charité – Universitätsmedizin Berlin (EA1/157/08). The skin tissue sections were selected from routine diagnostic dermatohistopathological procedures at the Department of Dermatology, Venerology and Allergology, Charité and used with written informed consent by the patients. Skin biopsies were processed for formalin-fixed-paraffin-embedded (FFPE) histopathology or cryopreserved for ICM studies as described^25^,^32^.

### Imaging cycler microscopy (ICM) and data processing

**Figure 5:**
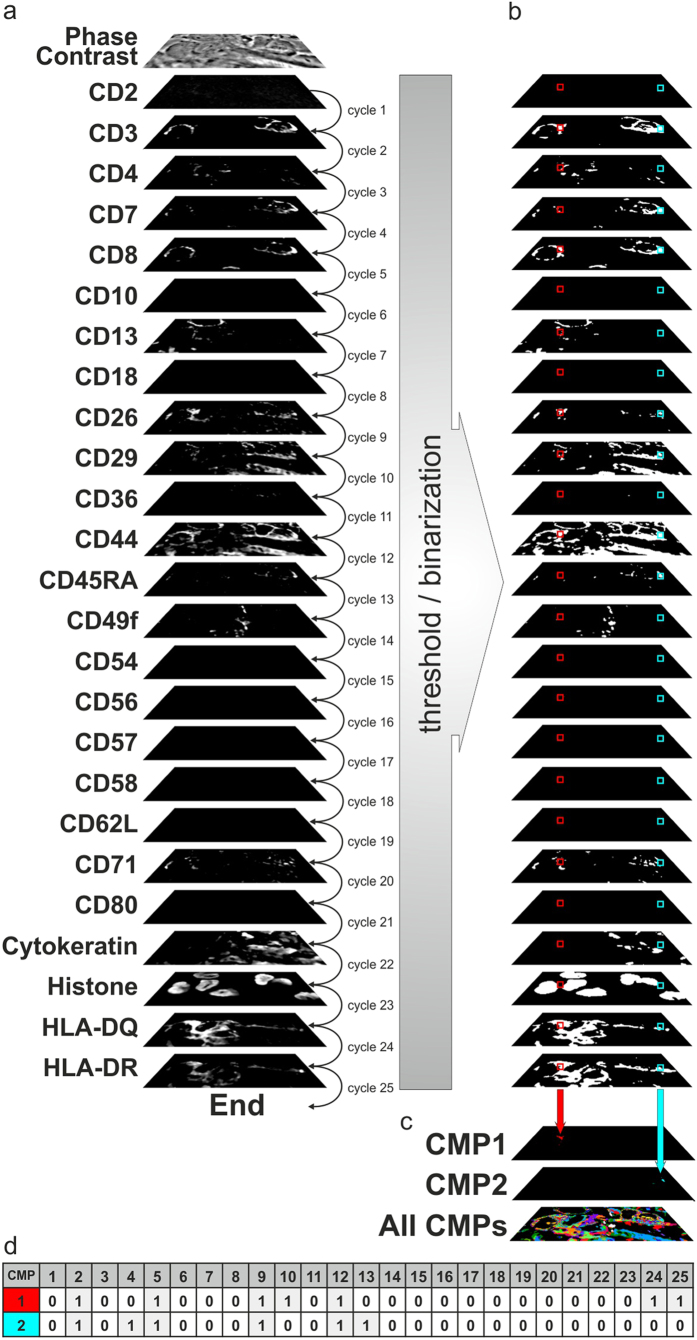
Illustration of the cyclical localization of 25 biomolecules and construction of combinatorial geometry map. (**a)** One optical plane out of 20 co-mapped planes in the z direction is shown to illustrate the fluorescence signals of the indicated biomolecules as in Supplementary Table 1. The visual field corresponds to the visual field shown in Fig. 1c. (**b)** binarization of the primary signals from **(a)** displayed parallel to **(a)**. (**c)** 2D map of the combinatorial molecular phenotypes (CMPs) for all data points computed from the binary data set of (**b)**. (**d)** two example CMPs from the data points in (**b)** indicated in red and blue, respectively, corresponding to the colored boxes in (**b)**. See Supplementary Video 4 for the scheme of CMP detection and Supplementary Video 3 for realtime 3D SPIKE interrogation.

An ICM robot (ToposNomos Ltd., Munich, Germany) was used to image random visual fields in frozen skin tissue sections at the dermo-epithelial junction, running robotically controlled, incubation – imaging – bleaching cycles on stage of an inverted epipfluorescence microscope. This can overcome the spectral resolution limit of the fluorescence microscope, as the number of cycles is principally not limited^25^. Briefly, here we used ICM to run cycles for the co-mapping of 25 cell surface-associated proteins together with histone/cytokeratin as markers for cellular nuclei/epithelial cells for orientation in a cryo-section of an MF skin lesion. Antibodies/affinity reagents for co-mapping had been validated earlier^25^. Their specificity is listed in Supplementary Table 1. Each cycle applies a specific FITC-conjugated antibody/affinity reagent recognizing and binding to a specific moiety in a preprogrammed sequence of robotic liquid handling on the sample of interest on stage. Together the robotic process involves: removal of an FITC-conjugated antibody/affinity reagent (probe from a probe container), transfer of the probe onto the sample, removal of this probe after incubation, washing the sample repeatedly with buffer, and fluorescence imaging of the corresponding moiety - binding site *in situ* of the tissue section using a CCD camera, finally repeated buffer incubations while the fluorescence signal is bleached at the excitation wavelength (472 ± 15 nm) and endpoint images after bleaching are recorded, which finalized Cycle 1. Cycle 2 is then initiated with a FITC-conjugated probe with a second binding specificity and so forth. Figure 5 shows the corresponding single-fluorescence signals per cycle of the tissue region displayed in Fig. 1(c) by exemplifying the first fluorescence signals obtained in one optical plane out of 20 imaged in z-direction of the tissue in 200 nm steps (see below). This was followed by a binarization step displaying each signal as absent or present (0/1) by using an expert-based^25^ and automated approach for threshold detection^44^,^45^ (Fig. 5b). The resulting combinatorial co-map contained 7,161 different topological assemblies (combinatorial molecular phenotypes, CMPs) within the tissue area of interest (Fig. 1b), where each pixel has a dimension of 200 nm × 200 nm (Supplementary Table 2). These distinct biomolecular arrangements (CMPs) together lead to a large combinatorial geometric structure (Fig. 5c, all CMPs) here referred to as binary 2D toponome map, in which distinct CMPs are displayed in different colors. For exemplified CMP annotation within this geometric structure see Fig. 5d. This method and proofs of specificity and selectivity of the mapping procedure has been described earlier^25^,^32^,^48^, and its use for biomarker detection has been validated experimentally^25^ and clinically^29^,^30^,^38^,^49^.

### ICM similarity mapping (SIM)

Besides the above described threshold-based method we applied a second, non-threshold-based method, called similarity mapping^35^,^36^ to extract the complete depth of information contained in multi-molecular assemblies at specific tissue sites (Fig. 3a,b). Briefly, the SIM method can compute the similarity/dissimilarity of the expression profiles of co-mapped biomolecules at any pixel of an ICM image data set. By navigating through the ICM tissue structures of the 25-component ICM image data set, SIM identifies mutually exclusive biomolecular profiles, which highlight sharp edged selectivity of cellular tissue structures (Fig. 3b; Supplementary Video 2). This is achieved by modern graphics processing unit (GPU) methods computing similarity and dissimilarity of all pixels at any time during user selected cursor movements across the 25-component ICM image. It is also possible to highlight distinct biomolecular profiles with different colors for direct interrogation of their tissue location as illustrated in Supplementary Video 2, which refers to the tissue area shown in Fig. 1c. This approach allowed us to determine protein profiles at the cell surfaces of SPIKE (Fig. 3a) and thereby precisely annotate the cell types forming the SPIKE multicellular assembly (Fig. 3a,b).

### 3D colocation mapping

For each optical plane of a z-stack the blur was computed and removed by using deconvolution software Huygens (Scientific Volume Imaging B.V., Hilversum, Netherlands). Further, the software Imaris (Bitplane AG, Zurich, Switzerland) was used for 3D co-mapping of the deconvoluted images. For generating 3D CMP data sets, we then applied expert-based or algorithm-based automated methods (see above) for determining gray value thresholds in each deconvoluted fluorescence signal of the 3D data set. The resulting binary fluorescence images were used to compute the corresponding CMPs. Display of these CMPs as 3D volume and quantitation of the resulting 3D CMPs was achieved by using the in-house software MultiCompare. (Supplementary Video 3).

## Additional Information

**How to cite this article**: Hillert, R. *et al.* Large molecular systems landscape uncovers T cell trapping in human skin cancer. *Sci. Rep.* **6**, 19012; doi: 10.1038/srep19012 (2016).

## Supplementary Material

Supplementary Information

Supplementary Video 1

Supplementary Video 2

Supplementary Video 3

Supplementary Video 4
