# Supplementary material for: Large molecular systems landscape uncovers T cell trapping in human skin cancer
Source: Sci Rep. 2016 Jan 13;6:19012. doi: 10.1038/srep19012 (PMC4725819; doi:10.1038/srep19012)
Supplement: Supplementary Information [file srep19012-s1.pdf]

Large molecular systems landscape uncovers T cell trapping in human skin cancer

Reyk Hillert, Anne Gieseler, Andreas Krusche, Daniel Humme, Hans-Joachim Röwert-Huber, Wolfram Sterry, Peter Walden, Walter Schubert

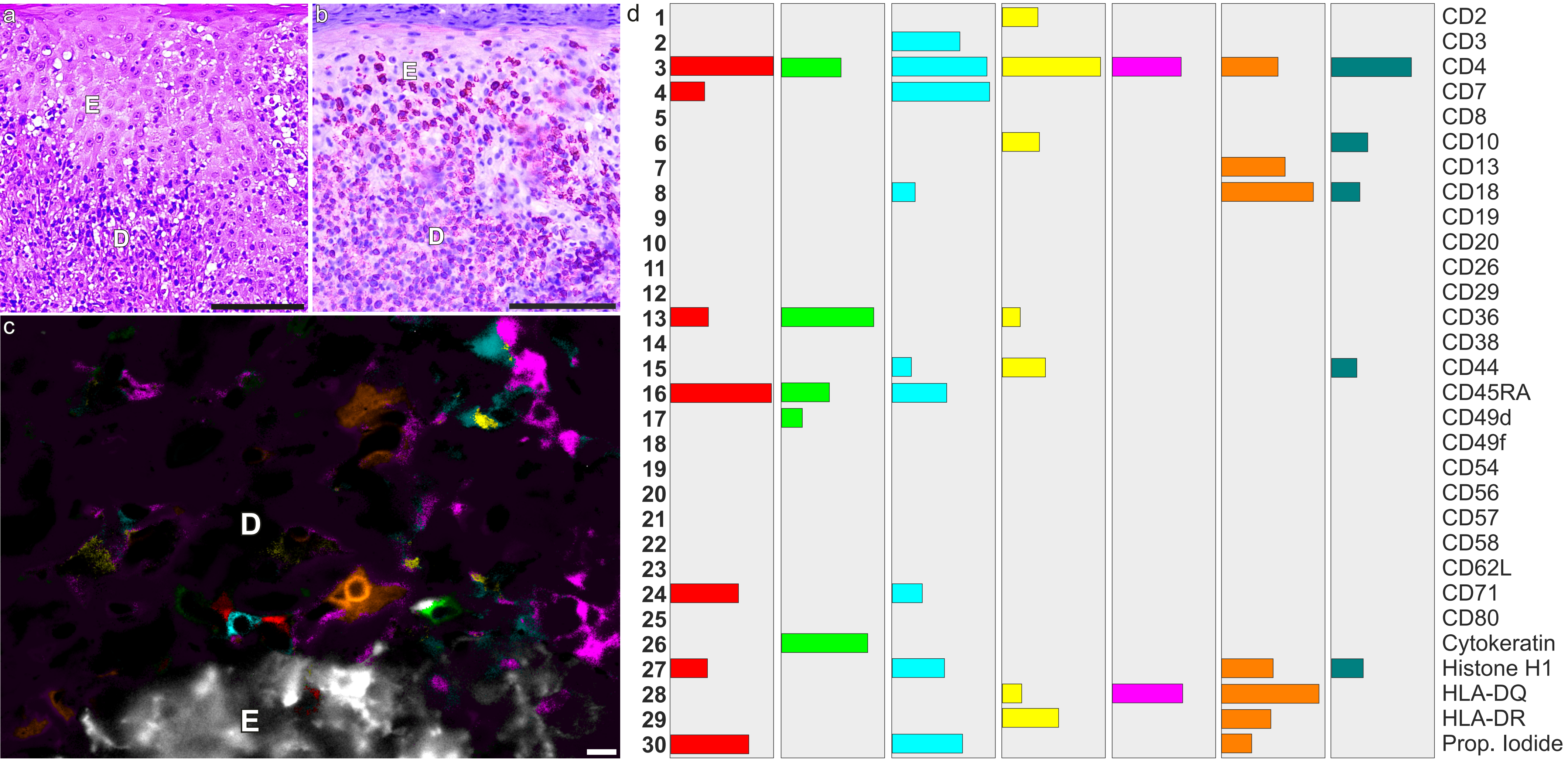

Supplementary Figure 1. a, Hematoxylin-Eosin (HE) stained formalin-fixed-paraffin-embedded (FFPE) tissue section of *Mycosis Fungoides* (MF) affected skin showing dense lymphocytic infiltrates with marked epidermotropism (relapsing stage of a MF case, plaque stage). Some lymphocytes have atypical nuclei typical for the tumor cells in MF. E=epidermis, D=dermis. **b**, immunohistochemistry staining for CD4 of a section from the same MF biopsy demonstrating that the tumor cells in this case are classical $CD4^+$ cutaneous lymphoma cells. E=epidermis, D=dermis. **c**, *in situ* 30-component molecular profiling (similarity mapping [35,36]) of the CD4 T lymphoma cells primarily clustering in dermis (D). Tumour cell clusters are observed away from the tumor microenvironment shown in Figure 1, 2 and 3. The epidermis (E) shows an abnormal pattern of cytokeratin expression. **d**, colors of 30-component profiles corresponding to the colors of CD4 cells expressing that particular profile in **c**. Protein profiles are measured per voxel in a 5 µm thick tissue section. For realtime similarity mapping see Supplementary Video 2. Bars: in a,b 100 µm; in c 10 µm

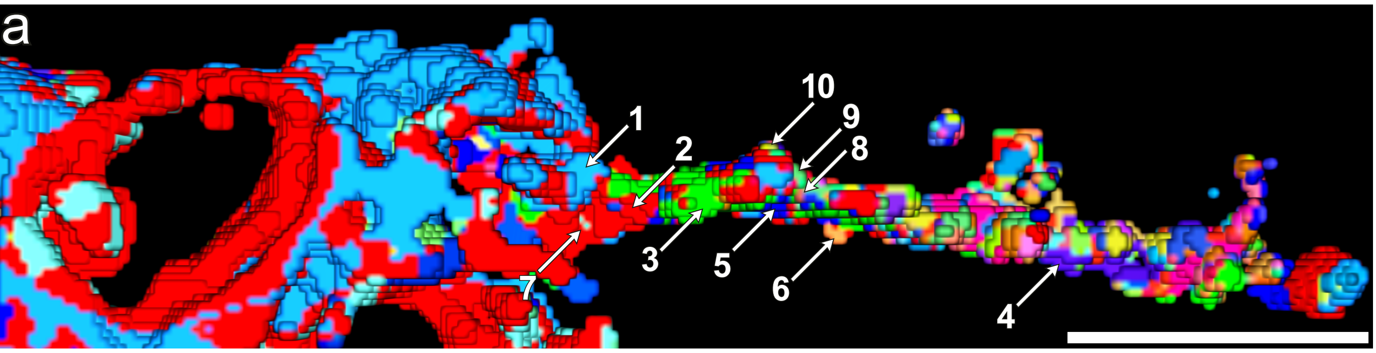

b

1 CD2
2 CD3
3 CD4
4 CD7
5 CD8
6 CD10
7 CD13
8 CD18
9 CD26
10 CD29
11 CD36
12 CD44
13 CD45
14 CD49f
15 CD54
16 CD56
17 CD57
18 CD58
19 CD62L
20 CD71
21 CD80
22 Cytok.
23 Histone
24 HLA-DQ
25 HLA-DR

c

CMP	1	2	3	4	5	6	7	8	9	10	11	12	13	14	15	16	17	18	19	20	21	22	23	24	25
1	0	0	0	0	0	0	0	0	0	0	0	0	0	0	0	0	0	0	0	0	0	0	0	1	0
2	0	0	0	0	0	0	0	0	0	0	0	1	0	0	0	0	0	0	0	0	0	0	0	1	0
3	0	0	0	0	0	0	0	0	0	1	0	1	0	0	0	0	0	0	0	0	0	0	0	1	0
4	0	0	0	0	0	0	0	0	0	0	0	0	0	0	0	0	0	0	0	0	0	0	0	1	0
5	0	0	0	0	0	0	0	0	0	0	0	1	0	0	0	0	0	0	0	0	0	0	0	1	1
6	0	0	0	0	0	0	0	0	0	0	0	1	0	0	0	0	0	0	0	0	0	1	0	1	0
7	0	0	0	0	0	0	0	0	1	0	0	1	0	0	0	0	0	0	0	0	0	0	0	1	0
8	0	0	0	0	0	0	0	0	1	1	0	1	0	0	0	0	0	0	0	0	0	0	0	1	0
9	0	0	0	1	0	0	0	0	1	1	0	1	0	0	0	0	0	0	0	0	0	0	0	1	1
10	0	1	0	1	0	0	0	0	0	0	0	1	0	0	0	0	0	0	0	0	0	0	0	1	0

Supplementary Figure 2. Supramolecular organisation of CMPs in SPIKE similar to Figure 3e with selected CMPs color decoding as indicated. Bar: 10µm

Supplementary Video Legends

Supplementary Video 1. Fly-through SPIKE for interrogation. The video sequence shows (i) alignment of the phase contrast image with superimposed fluorescence-labeled cell nuclei (blue) and basal lamina (white-yellow) and SPIKE´s lead proteins as in Figure 1 d; (ii) rocking of SPIKE to visualize the SPIKE-associated proteins and the interaction of the suprabasal cell extension of SPIKE with the suprabasal $CD8^{+}CD3^{+}$ T cell (brown color); (iii) virtual anatomical sectioning of the suprabasal $CD8^{+}CD3^{+}$ T cell revealing cytokeratin positive extension of the neighboring keratinocyte penetrating that cell; (iv) crossfading visualization of SPIKE's cell surface location of approx. 3,000 distinct 3D-CMPs (BL marker: CD49f, white color); (v) overview at wide angle; (vi) zooming onto SPIKE and direct real time interrogation of SPIKE´s 3D-CMPs along the interrogating cursor. The corresponding protein combinations per CMP are indicated at the right hand side of the screen. A more detailed interrogation of SPIKE is shown in Supplementary Video 3.

Supplementary Video 2. Realtime non-threshold based multi-protein profiling *in situ* (Similarity Mapping, SIM) [35,36] of SPIKE at 2D (one optical section). The data set corresponds to the boxed area in Figure 1.

Supplementary Video 3. 3D interactive interrogation of the SPIKE structure in realtime. The SPIKE structure found by 3D CMP combinatorial geometry in Figure 1a is magnified. Single 3D CMPs are visualized in different colors and animated by an arrow at visually selected sites while combinatorial molecular content of each so selected CMPs is seen in the molecular co-map list on the right hand side.

Supplementary Video 4. Video illustrating the step-wise acquisition of 3D-toponome data. The sequence of illustration is as follows. Video part 1: schematic illustration of the acquisition of optical sections across a tissue for each fluorochrome-labeled marker protein. Video part 2: illustration of the pixel-wise mapping of all labeled marker proteins in a given optical section of the whole 3D stack from video part 1. Note that this illustration shows protein signals as present or absent (1/0), while by using similarity mapping (SIM) protein profiles per pixel can also be mapped in a non-threshold based way [35,36,48]. (see Supplementary Video 2)

CD2	[CD2]	CD2 antigen (p50), sheep red blood cell receptor [Homo sapiens]
CD3	[CD3E]	CD3E antigen, epsilon polypeptide (TiT3 complex) [Homo sapiens]
CD4	[CD4]	CD4 antigen (p55) [Homo sapiens]
CD7	[CD7]	CD7 antigen (p41) [Homo sapiens]
CD8	[CD8A]	CD8 antigen, alpha polypeptide (p32) [Homo sapiens]
CD10	[MME]	membrane metallo-endopeptidase (neutral endopeptidase, enkephalinase, CALLA, CD10) [Homo sapiens]
CD13	[ANPEP]	alanyl (membrane) aminopeptidase (aminopeptidase N, aminopeptidase M, microsomal aminopeptidase, CD13, p150) [Homo sapiens]
CD18	[ITGB2]	integrin, beta 2 (antigen CD18 (p95), lymphocyte function-associated antigen 1; macrophage antigen 1 (mac-1) beta subunit) [Homo sapiens]
CD26	[DPP4]	dipeptidylpeptidase 4 (CD26, adenosine deaminase complexing protein 2) [Homo sapiens]
CD29	[ITGB1]	integrin, beta 1 (fibronectin receptor, beta polypeptide, antigen CD29 includes MDF2, MSK12) [Homo sapiens]
CD36	[CD36]	CD36 antigen (collagen type I receptor, thrombospondin receptor) [Homo sapiens]
CD44	[CD44]	CD44 antigen (homing function and Indian blood group system) [Homo sapiens]
CD45RA	[PTPRC]	protein tyrosine phosphatase, receptor type, C [Homo sapiens], containing the A exon
CD49f	[ITGA6]	integrin, alpha 6 [Homo sapiens]
CD54	[ICAM1]	intercellular adhesion molecule 1 (CD54), human rhinovirus receptor [Homo sapiens]
CD56	[NCAM1]	neural cell adhesion molecule 1 [Homo sapiens]
CD57	[CD57]	CD57 antigen [Homo sapiens]
CD58	[CD58]	CD58 antigen, (lymphocyte function-associated antigen 3) [Homo sapiens]
CD62L	[SELL]	selectin L (lymphocyte adhesion molecule 1) [Homo sapiens]
CD71	[TFRC]	transferrin receptor (p90, CD71) [Homo sapiens]
CD80	[CD80]	CD80 antigen (CD28 antigen ligand 1, B7-1 antigen) [Homo sapiens]
Cytokeratin	[KRT8], [KRT18]	keratin 8 [Homo sapiens] and keratin 18 [Homo sapiens]
Histone H1	[HIST1H1E]	Histone H1.4 [Homo sapiens] 10
HLA-DQ	[HLADQA1]	major histocompatibility complex, class II, DQ alpha 1 [Homo sapiens]
HLA-DR	[HLADRB1(ns)]	major histocompatibility complex, class II, DR beta 1 [Homo sapiens]

Supplementary Table 1. Annotation of the biomolecules co-mapped by ICM. While most of these molecules are associated with cell surfaces, histone and cytokeratin served as markers for subcellular localization of nuclei and intermediate filaments identifying keratinocytes for spatial histological orientation, respectively. CD49f serves as specific marker for basal lamina [26,48].

CMP	CD2	CD3	CD4	CD7	CD8	CD10	CD13	CD18	CD26	CD29	CD36	CD44	CD45RA	CD49f	CD54	CD56	CD57	CD58	CD62L	CD71	CD80	Cytokeratin	Histone_H1	HLA-DQ	HLA-DR	Freq. (voxel)
**0**	0	0	0	0	0	0	0	0	0	0	0	0	0	0	0	0	0	0	0	0	0	1	0	0	0	7.139.956
**1**	0	0	0	0	0	0	0	0	0	0	0	0	0	0	0	0	0	0	0	0	0	0	1	0	0	1.157.874
**2**	0	0	0	0	0	0	0	0	0	0	0	1	0	0	0	0	0	0	0	0	0	1	0	0	0	702.442
**3**	0	0	0	0	0	0	0	0	0	0	0	1	0	0	0	0	0	0	0	0	0	0	0	0	0	643.915
**4**	0	0	0	0	0	0	0	0	0	0	0	0	0	0	0	0	0	0	0	0	0	1	1	0	0	505.244
**5**	0	0	0	0	0	0	0	0	0	1	0	1	0	0	0	0	0	0	0	0	0	0	0	0	0	215.878
**6**	0	0	0	0	0	0	0	0	0	0	1	0	0	0	0	0	0	0	0	0	0	1	0	0	0	197.557
**7**	0	0	0	0	0	0	0	0	0	0	0	0	0	0	0	0	0	0	0	0	0	1	0	0	1	131.160
**8**	0	0	0	0	0	0	0	0	0	0	0	0	0	0	0	0	0	0	0	1	0	1	0	0	0	121.842
**9**	0	0	0	0	0	0	0	0	0	0	0	0	0	0	0	0	0	0	0	0	0	0	0	1	1	112.817
**10**	0	0	0	0	0	0	0	0	0	0	0	0	0	1	0	0	0	0	0	0	0	1	0	0	0	110.839
**11**	0	0	0	0	0	0	0	0	0	0	0	0	0	0	0	0	0	0	0	0	0	0	0	0	1	105.260
**12**	0	0	0	0	0	0	0	0	0	1	0	0	0	0	0	0	0	0	0	0	0	0	0	0	0	77.780
**13**	0	0	0	0	0	0	0	0	0	0	0	0	0	0	0	0	0	0	0	0	0	0	0	1	0	75.525
**14**	0	0	0	0	0	0	0	0	0	0	0	1	0	0	0	0	0	0	0	1	0	1	0	0	0	75.057
**15**	0	0	0	0	0	0	0	0	1	0	0	0	0	0	0	0	0	0	0	0	0	1	0	0	0	73.531
**16**	0	0	0	0	0	0	0	0	0	1	0	1	0	0	0	0	0	0	0	0	0	1	0	0	0	56.259
**17**	0	0	0	0	0	0	0	0	0	0	0	1	0	0	0	0	0	0	0	0	0	0	1	0	0	48.305
**18**	0	0	0	0	0	0	0	0	0	0	0	1	0	0	0	0	0	0	0	0	0	1	0	0	1	45.423
**19**	0	0	0	0	0	0	0	0	0	0	0	0	0	0	0	0	0	0	0	1	0	0	0	0	0	41.296
**20**	0	0	0	0	0	0	0	0	0	0	0	1	0	0	0	0	0	0	0	0	0	0	0	0	1	39.562
**21**	0	0	0	0	0	0	0	0	0	0	0	1	0	0	0	0	0	0	0	0	0	0	0	1	1	36.192
**22**	0	0	0	0	0	0	0	0	1	0	0	1	0	0	0	0	0	0	0	0	0	1	0	0	0	33.908
**23**	0	0	0	0	0	0	0	0	0	0	0	0	0	0	0	0	0	0	0	0	0	1	0	1	1	32.158
**24**	0	0	0	0	0	0	0	0	0	0	0	0	0	0	0	0	0	0	0	0	0	1	0	1	0	30.319
**25**	0	0	0	0	0	0	0	0	0	0	0	0	0	1	0	0	0	0	0	0	0	0	0	0	0	29.476
**26**	0	0	0	0	0	0	0	0	0	0	0	1	0	0	0	0	0	0	0	0	0	1	0	1	1	28.916
**27**	0	0	0	0	0	0	0	0	0	0	0	0	0	0	0	0	0	0	0	0	0	0	1	1	0	28.324
**28**	0	0	0	0	0	0	0	0	0	1	0	1	0	0	0	0	0	0	0	1	0	0	0	0	0	27.601
**29**	0	1	0	0	0	0	0	0	0	0	0	0	0	0	0	0	0	0	0	0	0	0	1	0	0	23.089
**30**	0	0	0	0	0	0	0	0	0	0	0	1	0	0	0	0	0	0	0	0	0	0	0	1	0	22.854
**31**	0	1	0	0	0	0	0	0	0	0	0	1	0	0	0	0	0	0	0	0	0	0	0	0	0	19.846
**32**	0	0	0	0	0	0	0	0	1	0	0	1	0	0	0	0	0	0	0	1	0	1	0	0	0	18.981
**33**	0	0	0	0	0	0	1	0	0	0	0	0	0	0	0	0	0	0	0	0	0	0	0	0	0	18.961
**34**	0	0	0	0	1	0	0	0	0	0	0	1	0	0	0	0	0	0	0	0	0	0	0	0	0	18.823
**35**	0	0	0	0	0	0	1	0	0	0	0	0	0	1	0	0	0	0	0	0	0	1	0	0	0	18.694
**36**	0	0	0	0	0	0	0	0	0	0	0	1	0	0	0	0	0	0	0	1	0	0	0	0	0	17.765
**37**	0	0	0	0	0	0	0	0	0	0	0	0	0	0	0	0	0	0	0	0	0	0	1	1	1	17.435
**38**	0	0	0	0	0	0	0	0	0	0	0	1	0	0	0	0	0	0	0	0	0	1	0	1	0	17.400
**39**	0	0	0	0	0	0	0	0	0	1	0	1	0	0	0	0	0	0	0	0	0	0	0	0	1	15.542
**40**	0	0	0	0	0	0	0	0	0	0	1	0	0	0	0	0	0	0	0	0	0	0	0	0	0	15.362
**41**	0	1	0	0	0	0	0	0	0	0	0	1	0	0	0	0	0	0	0	0	0	0	1	0	0	15.261
**42**	0	0	0	0	0	0	0	0	1	0	0	0	0	0	0	0	0	0	0	1	0	1	0	0	0	14.553
**43**	0	0	0	0	0	0	0	0	0	1	0	1	0	1	0	0	0	0	0	0	0	0	0	0	0	14.417
**44**	0	0	0	0	0	0	1	0	0	0	0	1	0	0	0	0	0	0	0	0	0	0	0	0	0	14.328
**45**	0	0	0	0	0	0	0	0	0	0	0	1	0	0	0	0	0	0	0	0	0	1	1	0	0	13.613
**46**	0	0	0	0	0	0	0	0	0	0	0	0	0	0	0	0	0	0	0	0	0	0	1	0	1	13.124
**47**	0	0	0	0	0	0	0	0	0	0	0	0	0	1	0	0	0	0	0	0	0	1	1	0	0	12.841
**48**	0	0	0	0	0	0	0	0	0	1	0	1	0	0	0	0	0	0	0	0	0	0	0	1	1	12.770
**49**	0	0	0	0	0	0	0	0	0	0	0	1	0	1	0	0	0	0	0	0	0	1	0	0	0	12.285
**50**	0	0	0	0	0	0	0	0	0	0	0	0	0	0	0	0	0	0	0	1	0	0	1	0	0	12.259
**51**	0	0	1	0	0	0	0	0	0	0	0	0	0	0	0	0	0	0	0	0	0	1	0	0	0	11.952
**52**	0	0	0	0	0	0	0	0	0	0	0	1	0	1	0	0	0	0	0	0	0	0	0	0	0	11.563
**53**	0	0	0	0	0	0	0	0	0	1	0	0	0	0	0	0	0	0	0	1	0	0	0	0	0	11.392
**54**	0	0	0	0	0	0	0	0	0	1	0	1	0	1	0	0	0	0	0	0	0	1	0	0	0	11.336
**55**	0	1	0	0	1	0	0	0	0	0	0	1	0	0	0	0	0	0	0	0	0	0	0	0	0	11.247
**56**	0	0	0	0	0	0	0	0	0	1	0	1	0	0	0	0	0	0	0	0	0	0	0	1	0	10.998
**57**	0	0	0	0	1	0	0	0	0	1	0	1	0	0	0	0	0	0	0	0	0	0	0	0	0	10.682
**58**	0	0	0	0	0	0	0	0	0	0	0	0	0	0	0	0	0	0	0	1	0	1	1	0	0	9.882
**59**	0	0	0	0	0	0	0	0	0	1	0	0	0	0	0	0	0	0	0	0	0	1	0	0	0	9.761
**60**	0	0	0	0	0	0	1	0	0	0	0	0	0	0	0	0	0	0	0	0	0	1	0	0	0	8.989
**61**	0	1	0	0	0	0	0	0	0	0	0	0	0	0	0	0	0	0	0	0	0	0	0	0	0	8.614
**62**	0	0	1	0	0	0	0	0	0	0	0	0	0	0	0	0	0	0	0	0	0	0	0	0	0	8.593
**63**	0	0	0	0	0	0	0	0	0	1	0	1	0	0	0	0	0	0	0	0	0	0	1	0	0	8.177
**64**	0	0	0	0	0	0	1	0	0	0	0	1	0	0	0	0	0	0	0	0	0	0	0	1	1	7.826
**65**	0	0	0	0	0	0	0	0	0	0	1	0	0	1	0	0	0	0	0	0	0	1	0	0	0	7.689
**66**	0	1	0	0	1	0	0	0	0	1	0	1	0	0	0	0	0	0	0	0	0	0	0	0	0	7.236
**67**	0	1	0	0	0	0	0	0	0	1	0	1	0	0	0	0	0	0	0	0	0	0	0	0	0	6.383
**68**	0	0	0	0	0	0	0	0	0	1	0	1	0	0	0	0	0	0	0	1	0	1	0	0	0	6.318
**69**	0	0	0	0	0	0	1	0	0	1	0	1	0	1	0	0	0	0	0	0	0	0	0	0	0	6.041
**70**	0	1	0	0	1	0	0	0	0	0	0	1	0	0	0	0	0	0	0	0	0	0	1	0	0	6.017
**71**	0	0	0	0	0	0	0	0	1	0	0	0	0	1	0	0	0	0	0	0	0	1	0	0	0	5.907
**72**	0	0	0	0	0	0	1	0	0	0	0	0	0	0	0	0	0	0	0	0	0	0	0	1	1	5.895
**73**	0	0	0	0	0	0	0	0	0	0	0	0	0	1	0	0	0	0	0	0	0	0	1	0	0	5.772
**74**	0	0	0	0	0	0	0	0	0	1	0	0	0	0	0	0	0	0	0	0	0	0	1	0	0	5.621
**75**	0	0	0	0	0	0	0	0	1	0	1	0	0	0	0	0	0	0	0	0	0	1	0	0	0	5.540
**76**	0	0	0	0	0	0	0	0	0	0	0	1	0	0	0	0	0	0	0	1	0	1	0	0	1	5.231
**77**	0	0	0	0	0	0	0	0	0	0	0	0	0	1	0	0	0	0	0	1	0	1	0	0	0	5.121
**78**	0	0	0	0	0	0	1	0	0	1	0	1	0	0	0	0	0	0	0	0	0	0	0	0	0	4.927
**79**	0	0	0	0	0	0	0	0	0	1	0	1	0	0	0	0	0	0	0	1	0	0	0	1	1	4.748
**80**	0	0	0	0	1	0	0	0	0	0	0	0	0	0	0	0	0	0	0	0	0	0	0	0	0	4.427
**81**	0	0	0	0	0	0	0	0	0	1	0	0	0	1	0	0	0	0	0	0	0	1	0	0	0	4.383
**82**	0	0	0	0	0	0	0	0	0	1	0	1	0	0	0	0	0	0	0	0	0	1	0	0	1	4.319
**83**	0	1	0	1	1	0	0	0	0	0	0	1	0	0	0	0	0	0	0	0	0	0	0	0	0	4.225
**84**	0	0	0	0	0	0	1	0	0	1	0	1	0	1	0	0	0	0	0	0	0	1	0	0	0	4.177
**85**	0	0	0	0	0	0	0	0	1	0	0	1	0	0	0	0	0	0	0	0	0	0	0	0	0	4.138
**86**	0	1	0	0	0	0	0	0	0	0	0	1	0	0	0	0	0	0	0	0	0	1	0	0	0	4.035
**87**	0	0	0	0	0	0	0	0	0	0	0	0	0	0	0	0	0	0	0	1	0	1	0	0	1	4.034
**88**	0	0	0	0	0	0	0	0	0	0	1	1	0	0	0	0	0	0	0	0	0	1	0	0	0	4.031
**89**	0	0	0	0	0	0	0	0	0	0	0	0	0	0	0	0	0	0	0	0	0	1	1	0	1	3.742
**90**	0	0	0	0	0	0	0	0	1	1	0	1	0	0	0	0	0	0	0	0	0	0	0	0	0	3.720
**91**	0	0	0	0	0	0	0	0	1	0	0	1	0	0	0	0	0	0	0	0	0	1	0	1	1	3.700
**92**	0	0	0	0	1	0	0	0	0	0	0	0	0	0	0	0	0	0	0	0	0	0	1	0	0	3.697
**93**	0	1	0	0	1	0	0	0	0	0	0	0	0	0	0	0	0	0	0	0	0	0	1	0	0	3.587
**94**	0	0	0	0	0	0	0	0	0	0	1	0	0	0	0	0	0	0	0	0	0	1	0	0	1	3.529
**95**	0	0	0	0	0	0	0	0	0	1	0	1	0	1	0	0	0	0	0	1	0	1	0	0	0	3.420
**96**	0	0	1	0	0	0	0	0	0	0	0	0	0	0	0	0	0	0	0	0	0	0	0	1	0	3.298
**97**	0	0	0	0	0	0	0	0	0	0	0	0	0	1	0	0	0	0	0	0	0	0	0	1	1	3.290
**98**	0	0	0	0	0	0	0	0	0	0	0	1	0	0	0	0	0	0	0	1	0	1	0	1	1	3.215
**99**	0	0	0	0	0	0	0	0	0	1	0	1	0	0	0	0	0	0	0	1	0	0	0	1	0	3.103
**100**	0	0	0	0	1	0	0	0	0	0	0	1	0	0	0	0	0	0	0	0	0	0	1	0	0	3.059
**101**	0	0	0	0	0	0	1	0	0	0	0	1	0	0	0	0	0	0	0	0	0	0	0	1	0	2.997
**102**	0	0	0	0	1	0	0	0	0	0	0	1	0	0	0	0	0	0	0	0	0	1	0	0	0	2.967
**103**	0	0	0	1	0	0	0	0	0	0	0	1	0	0	0	0	0	0	0	0	0	0	0	0	0	2.946
**104**	0	1	0	0	1	0	0	0	0	0	0	1	1	0	0	0	0	0	0	0	0	0	0	0	0	2.900
**105**	0	0	0	0	0	0	0	0	0	0	0	0	0	0	0	0	0	0	0	0	0	1	1	1	0	2.878
**106**	0	0	0	0	0	0	0	0	0	0	0	1	0	1	0	0	0	0	0	1	0	1	0	0	0	2.847
**107**	0	0	0	0	0	0	1	0	0	0	0	0	0	0	0	0	0	0	0	0	0	0	0	1	0	2.835
**108**	0	0	0	0	0	0	0	0	0	1	0	1	0	1	0	0	0	0	0	0	0	1	1	0	0	2.786
**109**	0	0	1	0	0	0	0	0	0	0	0	0	0	0	0	0	0	0	0	0	0	0	0	1	1	2.763
**110**	0	0	0	0	0	0	0	0	0	0	0	0	0	1	0	0	0	0	0	0	0	1	0	0	1	2.720
**111**	0	0	0	0	0	0	0	0	0	1	0	0	0	1	0	0	0	0	0	0	0	0	0	0	0	2.649
**112**	0	0	0	0	0	0	0	0	1	0	0	0	0	0	0	0	0	0	0	0	0	0	0	0	0	2.645
**113**	0	0	0	0	0	0	0	0	0	1	0	1	0	0	0	0	0	0	0	1	0	0	0	0	1	2.562
**114**	0	1	0	0	1	0	0	0	1	1	0	1	0	0	0	0	0	0	0	0	0	0	0	0	0	2.520
**115**	0	0	0	0	0	0	0	0	1	0	0	1	0	0	0	0	0	0	0	0	0	1	0	0	1	2.462
**116**	0	0	0	0	0	0	1	0	0	0	0	0	0	1	0	0	0	0	0	0	0	0	0	0	0	2.440
**117**	0	0	0	0	0	0	0	0	0	0	0	0	0	0	0	0	0	0	0	1	0	0	0	1	1	2.403
**118**	0	1	0	0	0	0	0	0	0	1	0	1	0	0	0	0	0	0	0	0	0	0	1	0	0	2.381
**119**	0	0	0	0	0	0	1	0	0	0	0	0	0	1	0	0	0	0	0	0	0	1	1	0	0	2.362
**120**	0	0	0	0	0	0	0	0	1	0	0	1	0	0	0	0	0	0	0	0	0	1	0	1	0	2.355
**121**	0	0	0	0	0	0	0	0	0	0	0	0	1	0	0	0	0	0	0	0	0	1	0	0	0	2.325

CMP	CD2	CD3	CD4	CD7	CD8	CD10	CD13	CD18	CD26	CD29	CD36	CD44	CD45RA	CD49f	CD54	CD56	CD57	CD58	CD62L	CD71	CD80	Cytokeratin	Histone_H1	HLA-DQ	HLA-DR	Freq. (voxel)
**122**	0	1	0	1	0	0	0	0	0	0	0	1	0	0	0	0	0	0	0	0	0	0	0	0	0	2.322
**123**	0	0	0	0	0	0	0	0	0	0	1	0	0	0	0	0	0	0	0	0	0	1	1	0	0	2.286
**124**	0	0	0	0	0	0	0	0	1	0	0	0	0	0	0	0	0	0	0	0	0	1	0	0	1	2.278
**125**	0	0	0	0	0	0	0	0	0	1	0	1	0	1	0	0	0	0	0	1	0	1	1	0	0	2.229
**126**	0	0	0	0	0	0	0	0	0	1	0	1	0	0	0	0	0	0	0	0	0	1	1	0	0	2.190
**127**	0	1	0	1	1	0	0	0	0	1	0	1	0	0	0	0	0	0	0	0	0	0	0	0	0	2.183
**128**	0	0	0	0	0	0	0	0	0	0	0	1	0	0	0	0	0	0	0	1	0	1	1	0	0	2.169
**129**	0	0	0	0	0	0	0	0	1	1	0	1	0	0	0	0	0	0	0	0	0	1	0	0	0	2.166
**130**	0	0	0	0	0	0	1	0	0	0	0	1	0	0	0	0	0	0	0	0	0	0	0	0	1	2.155
**131**	0	0	0	0	0	0	0	0	0	0	0	1	0	0	0	0	0	0	0	0	0	0	1	1	0	2.153
**132**	0	1	0	0	1	0	0	0	0	0	0	1	0	0	0	0	0	0	0	0	0	1	0	0	0	2.143
**133**	0	1	0	0	0	0	0	0	0	0	0	1	0	0	0	0	0	0	0	0	0	1	1	0	0	2.138
**134**	0	0	0	0	0	0	0	0	0	0	0	0	0	0	0	1	0	0	0	0	0	1	0	0	0	2.109
**135**	0	0	0	0	0	0	0	0	1	0	0	1	0	0	0	0	0	0	0	1	0	1	0	0	1	2.103
**136**	0	0	0	0	0	0	1	0	0	0	0	0	0	0	0	0	0	0	0	0	0	0	1	0	0	2.076
**137**	0	0	0	0	0	0	0	0	0	0	1	0	0	0	0	0	0	0	0	0	0	0	0	1	0	2.072
**138**	0	0	0	0	0	0	1	0	0	0	0	0	0	1	0	0	0	0	0	1	0	1	0	0	0	2.046
**139**	0	0	0	0	0	0	0	0	0	0	0	1	0	0	0	0	0	0	0	1	0	0	0	1	1	2.017
**140**	0	0	0	0	0	0	1	0	0	1	0	0	0	1	0	0	0	0	0	0	0	1	0	0	0	2.016
**141**	0	0	0	1	0	0	0	0	0	0	0	0	0	0	0	0	0	0	0	0	0	0	0	0	0	2.015
**142**	0	0	0	0	0	0	1	0	0	0	0	0	0	0	0	0	0	0	0	0	0	0	0	0	1	1.990
**143**	0	0	0	0	0	0	1	0	0	0	1	0	0	1	0	0	0	0	0	0	0	1	0	0	0	1.943
**144**	0	0	0	0	0	0	0	0	0	1	0	1	0	0	0	0	0	0	0	0	0	1	0	1	0	1.943
**145**	0	1	0	0	1	0	0	0	0	1	0	1	0	0	0	0	0	0	0	0	0	0	1	0	0	1.929
**146**	0	0	0	0	1	0	0	0	0	0	0	1	0	0	0	0	0	0	0	0	0	0	0	0	1	1.879
**147**	0	0	0	0	0	0	1	0	0	1	0	1	0	1	0	0	0	0	0	0	0	0	0	1	1	1.861
**148**	0	0	0	0	0	0	1	0	0	1	0	1	0	1	0	0	0	0	0	1	0	1	0	0	0	1.852
**149**	0	0	0	0	0	0	1	0	0	0	0	1	0	0	0	0	0	0	0	1	0	0	0	1	1	1.827
**150**	0	1	0	0	0	0	0	0	0	0	0	0	0	0	0	0	0	0	0	0	0	1	0	0	0	1.827
**151**	0	0	0	0	0	0	0	0	0	1	0	0	0	0	0	0	0	0	0	1	0	1	0	0	0	1.818
**152**	0	0	0	0	0	0	1	0	0	0	0	1	0	1	0	0	0	0	0	0	0	1	0	0	0	1.805
**153**	0	0	0	0	0	0	0	0	0	0	0	0	0	0	0	0	0	0	0	1	0	1	0	1	1	1.801
**154**	0	0	0	0	0	0	0	0	0	0	0	0	0	1	0	0	0	0	0	0	0	0	0	1	0	1.801
**155**	0	0	0	0	0	0	0	0	0	0	0	0	0	0	0	0	0	0	0	1	0	1	0	1	0	1.792
**156**	0	0	0	0	0	0	1	0	0	1	0	0	0	0	0	0	0	0	0	0	0	0	0	0	0	1.770
**157**	0	1	0	0	0	0	0	0	0	0	0	0	0	0	0	0	0	0	0	0	0	1	1	0	0	1.753
**158**	0	0	0	0	0	0	0	0	0	0	1	0	0	0	0	0	0	0	0	1	0	1	0	0	0	1.753
**159**	0	0	0	0	1	0	0	0	0	1	0	1	0	0	0	0	0	0	0	1	0	0	0	0	0	1.736
**160**	0	0	0	1	1	0	0	0	0	0	0	1	0	0	0	0	0	0	0	0	0	0	0	0	0	1.717
**161**	0	1	0	0	0	0	0	0	0	0	0	0	0	0	0	0	0	0	0	1	0	0	1	0	0	1.707
**162**	0	0	0	0	0	0	0	0	0	1	0	1	0	0	0	0	0	0	0	0	0	1	0	1	1	1.694
**163**	0	0	0	0	0	0	0	0	0	0	1	1	0	0	0	0	0	0	0	0	0	0	0	0	0	1.689
**164**	0	0	0	0	0	0	1	0	0	1	0	1	0	1	0	0	0	0	0	0	0	0	0	1	0	1.666
**165**	0	0	0	0	0	0	0	0	0	0	0	1	0	0	0	0	0	0	0	1	0	1	0	1	0	1.649
**166**	0	1	0	0	0	0	0	0	0	0	0	1	0	0	0	0	0	0	0	0	0	0	0	0	1	1.621
**167**	0	1	0	1	1	0	0	0	0	0	0	1	0	0	0	0	0	0	0	0	0	0	1	0	0	1.619
**168**	0	0	0	0	0	0	0	0	0	1	0	1	0	1	0	0	0	0	0	1	0	0	0	0	0	1.606
**169**	0	0	0	0	0	0	0	0	0	0	0	0	0	1	0	0	0	0	0	1	0	1	1	0	0	1.602
**170**	0	1	0	1	0	0	0	0	0	0	0	0	0	0	0	0	0	0	0	0	0	0	1	0	0	1.577
**171**	0	1	0	0	1	0	0	0	0	0	0	1	0	0	0	0	0	0	0	0	0	0	0	1	1	1.569
**172**	0	1	0	1	1	0	0	0	0	0	0	1	0	0	0	0	0	0	0	1	0	0	0	0	0	1.569
**173**	0	0	0	0	0	0	0	0	0	0	0	1	0	0	0	0	0	0	0	0	0	0	1	1	1	1.544
**174**	0	1	0	1	0	0	0	0	0	0	0	1	0	0	0	0	0	0	0	0	0	0	1	0	0	1.541
**175**	0	0	0	0	1	0	0	0	0	0	0	1	0	0	0	0	0	0	0	0	0	0	0	1	1	1.528
**176**	0	0	1	0	0	0	0	0	0	0	0	0	0	0	0	0	0	0	0	0	0	0	1	0	0	1.524
**177**	0	1	0	0	0	0	0	0	0	0	0	0	0	0	0	0	0	0	0	0	0	0	1	0	1	1.522
**178**	0	0	0	0	0	0	0	0	0	0	0	0	1	0	0	0	0	0	0	0	0	0	0	0	0	1.501
**179**	0	1	0	0	1	0	0	0	0	0	0	1	0	0	0	0	0	0	0	0	0	0	0	0	1	1.490
**180**	0	1	0	0	0	0	0	0	1	0	0	1	0	0	0	0	0	0	0	0	0	0	0	0	0	1.487
**181**	0	0	0	0	0	0	0	0	0	0	0	1	0	1	0	0	0	0	0	0	0	1	0	0	1	1.485
**182**	0	0	0	0	0	0	0	0	0	1	0	0	0	0	0	0	0	0	0	0	0	0	0	1	1	1.467
**183**	0	0	0	0	0	0	0	0	0	0	0	1	0	0	0	0	0	0	0	1	0	0	0	0	1	1.462
**184**	0	0	0	0	0	0	1	0	0	0	0	0	0	0	0	0	0	0	0	1	0	0	0	0	0	1.461
**185**	0	0	1	0	0	0	0	0	0	0	0	0	0	0	0	0	0	0	0	1	0	0	0	0	0	1.444
**186**	0	0	0	0	0	0	0	0	0	1	0	0	0	0	0	0	0	0	0	0	0	0	0	1	0	1.444
**187**	0	0	0	0	0	0	0	0	0	0	1	0	0	0	0	0	0	0	0	0	0	0	0	1	1	1.442
**188**	0	0	0	0	0	0	0	0	0	0	0	1	0	0	0	0	0	0	0	1	0	0	1	0	0	1.442
**189**	0	0	0	0	0	0	0	0	1	0	0	1	0	0	0	0	0	0	0	1	0	1	0	1	1	1.424
**190**	0	0	1	0	0	0	0	0	0	0	0	1	0	0	0	0	0	0	0	0	0	1	0	0	0	1.405
**191**	0	0	0	0	1	0	0	0	1	1	0	1	0	0	0	0	0	0	0	0	0	0	0	0	0	1.394
**192**	0	0	0	0	0	0	0	0	0	0	0	0	0	0	0	0	0	0	0	1	0	0	0	1	0	1.388
**193**	0	0	0	0	1	0	0	0	0	0	0	1	1	0	0	0	0	0	0	0	0	0	0	0	0	1.385
**194**	0	1	0	0	0	0	0	0	0	0	0	1	0	0	0	0	0	0	0	0	0	0	0	1	1	1.366
**195**	0	0	0	0	0	0	1	0	0	0	0	1	0	1	0	0	0	0	0	0	0	0	0	0	0	1.356
**196**	0	1	0	0	1	0	0	0	0	0	0	1	0	0	0	0	0	0	0	1	0	0	0	0	0	1.356
**197**	0	0	0	0	0	0	0	0	1	0	0	1	0	1	0	0	0	0	0	0	0	1	0	0	0	1.353
**198**	0	0	0	0	0	0	1	0	0	1	0	1	0	1	0	0	0	0	0	1	0	1	1	0	0	1.322
**199**	0	1	0	0	0	0	0	0	0	0	0	1	0	0	0	0	0	0	0	1	0	0	1	0	0	1.292
**200**	0	0	0	0	0	0	0	0	1	0	0	0	0	0	0	0	0	0	0	0	0	1	0	1	0	1.286
**201**	0	1	0	0	0	0	0	0	0	0	0	1	0	0	0	0	0	0	0	1	0	0	0	0	0	1.283
**202**	0	0	0	0	0	0	0	0	0	0	0	0	0	0	0	0	0	0	0	1	0	0	0	0	1	1.281
**203**	0	0	0	0	0	0	0	0	0	1	0	1	0	1	0	0	0	0	0	0	0	0	0	1	0	1.266
**204**	0	0	0	0	0	0	0	0	0	0	0	0	1	0	0	0	0	0	0	0	0	0	0	1	0	1.265
**205**	0	0	0	1	0	0	0	0	0	0	0	0	0	0	0	0	0	0	0	0	0	0	1	0	0	1.264
**206**	0	0	0	0	1	0	0	0	0	1	0	1	0	0	0	0	0	0	0	0	0	1	0	0	0	1.262
**207**	0	0	0	0	0	0	0	0	1	0	0	0	0	1	0	0	0	0	0	1	0	1	0	0	0	1.243
**208**	0	1	0	1	1	0	0	0	0	0	0	1	1	0	0	0	0	0	0	0	0	0	0	0	0	1.243
**209**	0	0	0	0	0	0	0	0	0	0	0	1	0	1	0	0	0	0	0	0	0	0	0	1	1	1.232
**210**	0	1	0	0	1	0	0	0	1	0	0	1	0	0	0	0	0	0	0	0	0	0	0	0	0	1.224
**211**	0	1	0	1	1	0	0	0	1	1	0	1	0	0	0	0	0	0	0	0	0	0	0	0	0	1.219
**212**	0	0	0	0	0	0	0	0	1	0	0	1	0	1	0	0	0	0	0	1	0	1	0	0	0	1.216
**213**	0	0	0	0	0	0	0	0	0	1	0	1	0	1	0	0	0	0	0	0	0	1	0	0	1	1.187
**214**	0	0	1	0	0	0	0	0	0	0	0	1	0	0	0	0	0	0	0	0	0	0	0	0	0	1.182
**215**	0	0	0	0	0	0	1	0	1	0	0	0	0	1	0	0	0	0	0	0	0	1	0	0	0	1.177
**216**	0	0	0	0	0	0	0	0	0	1	0	0	0	1	0	0	0	0	0	0	0	1	1	0	0	1.176
**217**	0	0	1	0	0	0	0	0	0	0	0	1	0	0	0	0	0	0	0	0	0	0	0	1	1	1.176
**218**	0	0	0	0	0	0	0	0	0	0	0	0	1	0	0	1	0	0	0	0	0	0	0	0	0	1.168
**219**	0	0	0	0	0	0	1	0	0	0	0	0	0	0	0	0	0	0	0	1	0	1	0	0	0	1.164
**220**	0	0	0	0	0	0	0	0	0	0	0	1	0	1	0	0	0	0	0	0	0	0	0	1	0	1.161
**221**	0	0	0	0	0	0	0	0	0	0	0	1	0	1	0	0	0	0	0	0	0	1	1	0	0	1.153
**222**	0	1	0	0	1	0	0	0	0	1	0	1	1	0	0	0	0	0	0	0	0	0	0	0	0	1.121
**223**	0	0	0	0	0	0	0	0	1	0	0	0	0	0	0	0	0	0	0	0	0	1	0	1	1	1.112
**224**	0	1	0	1	1	0	0	0	0	1	0	1	0	0	0	0	0	0	0	1	0	0	0	0	0	1.100
**225**	0	1	0	0	0	0	0	0	1	1	0	1	0	0	0	0	0	0	0	0	0	0	0	0	0	1.086
**226**	0	0	0	0	0	0	0	0	0	0	0	1	0	1	0	0	0	0	0	1	0	1	1	0	0	1.083
**227**	0	0	0	0	0	0	0	0	1	0	0	1	0	0	0	0	0	0	0	0	0	0	0	1	1	1.067
**228**	0	0	0	0	0	0	0	0	0	0	0	1	0	0	0	1	0	0	0	0	0	0	0	1	1	1.058
**229**	0	0	0	0	0	0	0	0	0	0	0	0	0	0	0	1	0	0	0	0	0	0	0	0	0	1.054
**230**	0	0	0	0	0	0	0	0	1	1	0	1	0	0	0	0	0	0	0	1	0	0	0	0	0	1.046
**231**	0	0	1	0	0	0	0	0	0	0	0	0	0	0	0	0	0	0	0	1	0	1	0	0	0	1.043
**232**	0	0	0	0	0	0	0	0	0	0	0	1	0	0	0	0	0	0	0	0	0	0	1	0	1	1.036
**233**	0	0	0	0	0	0	1	0	0	1	0	0	0	1	0	0	0	0	0	0	0	0	0	0	0	1.030
**234**	0	0	0	0	0	0	1	0	0	1	0	1	0	0	0	0	0	0	0	0	0	0	0	1	1	1.022
**235**	0	0	0	0	0	0	0	0	1	0	0	0	0	0	0	0	0	0	0	0	0	1	1	0	0	1.009
**236**	0	0	0	0	0	0	0	0	0	1	0	1	0	1	0	0	0	0	0	0	0	0	0	1	1	1.004
**237**	0	1	0	1	0	0	0	0	0	0	0	1	0	0	0	0	0	0	0	1	0	0	0	0	0	996
**238**	0	0	0	0	0	0	0	0	0	1	0	1	0	0	0	0	0	0	0	1	0	1	0	0	1	995
**239**	0	1	0	0	0	0	0	0	0	0	0	1	0	0	0	0	0	0	0	0	0	1	0	0	1	992
**240**	0	1	0	0	1	0	0	0	0	0	0	1	0	0	0	0	0	0	0	1	0	0	1	0	0	991
**241**	0	0	0	1	0	0	0	0	0	1	0	1	0	0	0	0	0	0	0	0	0	0	0	0	0	989
**242**	0	1	0	0	0	0	0	0	0	0	0	0	0	0	0	0	0	0	0	0	0	0	0	0	1	978
**243**	0	0	0	0	0	0	1	0	0	1	0	1	0	1	0	0	0	0	0	0	0	0	0	0	1	964

CMP	CD2	CD3	CD4	CD7	CD8	CD10	CD13	CD18	CD26	CD29	CD36	CD44	CD45RA	CD49f	CD54	CD56	CD57	CD58	CD62L	CD71	CD80	Cytokeratin	Histone_H1	HLA-DQ	HLA-DR	Freq. (voxel)
**244**	0	0	0	0	0	0	1	0	0	0	0	1	0	1	0	0	0	0	0	0	0	0	0	1	1	957
**245**	0	0	1	0	0	0	0	0	0	1	0	0	0	0	0	0	0	0	0	0	0	0	0	0	0	957
**246**	0	0	0	0	1	0	0	0	0	0	0	0	0	0	0	0	0	0	0	0	0	1	0	0	0	934
**247**	0	0	0	0	0	0	1	0	0	0	0	1	0	0	0	0	0	0	0	1	0	0	0	0	0	933
**248**	0	1	0	1	1	0	0	0	1	1	0	1	0	0	0	0	0	0	0	1	0	0	0	0	0	929
**249**	0	0	0	0	0	0	1	0	0	0	0	1	0	0	0	0	0	0	0	0	0	1	0	0	0	926
**250**	0	1	0	0	1	0	0	0	0	1	0	1	0	0	0	0	0	0	0	1	0	0	0	0	0	925
**251**	0	0	0	0	0	0	0	0	1	0	0	1	0	0	0	0	0	0	0	1	0	0	0	0	0	921
**252**	0	0	0	0	0	0	0	0	0	1	0	1	0	1	0	0	0	0	0	0	0	0	1	0	0	917
**253**	0	0	0	1	1	0	0	0	0	1	0	1	0	0	0	0	0	0	0	0	0	0	0	0	0	915
**254**	0	1	0	1	0	0	0	0	0	0	0	0	0	0	0	0	0	0	0	0	0	0	0	0	0	914
**255**	0	0	0	0	0	0	0	0	0	0	0	0	0	0	0	1	0	0	0	0	0	0	0	1	1	913
**256**	0	1	0	1	1	0	0	0	0	1	0	1	1	0	0	0	0	0	0	0	0	0	0	0	0	911
**257**	0	0	0	0	0	0	0	0	0	0	0	0	0	1	0	0	0	0	0	0	0	1	0	1	1	908
**258**	0	0	0	0	0	0	0	0	0	0	0	0	0	0	0	0	0	0	0	0	0	1	1	1	1	906
**259**	0	1	0	1	1	0	0	0	0	0	0	1	1	0	0	0	0	0	0	1	0	0	0	0	0	900
**260**	0	1	0	0	0	0	0	0	0	0	0	1	0	0	0	0	0	0	0	0	0	0	0	1	0	898
**261**	0	0	0	0	0	0	0	0	0	0	0	0	0	1	0	0	0	0	0	0	0	0	0	0	1	897
**262**	0	0	0	0	0	0	0	0	0	0	0	0	1	0	0	1	0	0	0	0	0	0	0	1	0	893
**263**	0	0	0	0	0	0	0	0	0	1	0	1	0	0	0	0	0	0	0	1	0	0	1	0	0	888
**264**	0	0	0	0	0	0	1	0	0	1	0	1	0	1	0	0	0	0	0	1	0	0	0	1	1	886
**265**	0	1	0	0	0	0	0	0	0	0	0	1	0	0	0	0	0	0	0	0	0	1	0	1	1	881
**266**	0	0	0	0	1	0	0	0	0	0	0	1	0	0	0	0	0	0	0	1	0	0	0	0	0	874
**267**	0	0	0	0	0	0	0	0	0	1	0	1	0	0	0	0	0	0	0	0	0	0	1	1	0	870
**268**	0	0	0	0	0	0	1	0	0	0	0	0	0	0	0	0	0	0	0	0	0	1	1	0	0	867
**269**	0	0	0	0	0	0	0	0	0	1	0	1	0	1	0	0	0	0	0	1	0	1	0	0	1	861
**270**	0	0	0	0	0	0	1	0	1	0	0	1	0	0	0	0	0	0	0	0	0	0	0	1	1	851
**271**	0	0	0	0	0	0	0	0	0	0	1	1	0	0	0	0	0	0	0	0	0	0	0	1	1	848
**272**	0	0	0	0	0	0	0	0	0	1	0	0	0	0	0	0	0	0	0	0	0	0	1	1	0	847
**273**	0	0	0	0	1	0	0	0	0	1	0	1	0	0	0	0	0	0	0	0	0	0	0	0	1	839
**274**	0	1	0	0	1	0	0	0	0	0	0	0	0	0	0	0	0	0	0	0	0	0	0	0	0	838
**275**	0	0	0	0	0	0	1	0	0	1	0	1	0	0	0	0	0	0	0	0	0	0	0	1	0	830
**276**	0	0	0	0	1	0	0	0	0	0	0	0	0	0	0	0	0	0	0	0	0	0	0	0	1	825
**277**	0	0	0	0	0	0	0	0	0	1	0	1	0	1	0	0	0	0	0	0	0	0	0	0	1	822
**278**	0	1	0	0	1	0	0	0	0	1	0	1	0	0	0	0	0	0	0	0	0	0	0	1	1	816
**279**	0	1	0	1	1	0	0	0	0	0	0	1	0	0	0	0	0	0	0	1	0	0	1	0	0	815
**280**	0	0	0	0	0	0	0	0	1	0	0	0	0	0	0	0	0	0	0	1	0	0	0	0	0	810
**281**	0	0	0	0	0	0	0	0	0	0	1	0	0	0	0	0	0	0	0	0	0	0	1	0	0	808
**282**	0	0	0	0	0	0	1	0	0	1	0	1	0	1	0	0	0	0	0	0	0	1	1	0	0	804
**283**	0	1	0	0	1	0	0	0	0	0	0	1	1	0	0	0	0	0	0	0	0	0	1	0	0	803
**284**	0	0	0	0	0	0	0	0	1	0	0	0	0	0	0	0	0	0	0	0	0	0	1	0	0	799
**285**	0	1	0	0	1	0	0	0	1	0	0	1	0	0	0	0	0	0	0	0	0	0	0	1	1	797
**286**	0	0	0	0	0	0	0	0	0	0	0	1	0	1	0	0	0	0	0	0	0	0	1	0	0	788
**287**	0	0	0	1	0	0	0	0	0	0	0	1	0	0	0	0	0	0	0	0	0	0	1	0	0	784
**288**	0	0	0	0	0	0	0	0	1	0	0	1	0	0	0	0	0	0	0	1	0	1	0	1	0	767
**289**	0	0	0	0	1	0	0	0	0	1	0	1	0	1	0	0	0	0	0	0	0	0	0	0	0	763
**290**	0	0	0	0	0	0	0	0	0	0	0	1	0	0	0	0	0	0	0	1	0	0	0	1	0	761
**291**	0	0	1	0	0	0	0	0	0	1	0	1	0	0	0	0	0	0	0	1	0	0	0	1	1	760
**292**	0	0	0	0	1	0	0	0	0	1	0	1	0	0	0	0	0	0	0	0	0	0	1	0	0	756
**293**	0	0	0	0	0	0	0	0	0	0	0	0	0	1	0	0	0	0	0	0	0	1	0	1	0	748
**294**	0	0	0	0	0	0	0	0	0	1	0	1	0	1	0	0	0	0	0	1	0	0	0	1	1	739
**295**	0	0	0	0	0	0	0	0	0	1	0	0	0	0	0	0	0	0	0	0	0	0	0	0	1	726
**296**	0	0	1	0	0	0	0	0	0	1	0	1	0	0	0	0	0	0	0	0	0	0	0	0	0	725
**297**	0	0	1	0	0	0	0	0	0	1	0	0	0	0	0	0	0	0	0	1	0	0	0	0	0	723
**298**	0	0	1	0	0	0	1	0	0	1	0	1	0	1	0	0	0	0	0	0	0	0	0	1	1	719
**299**	0	1	0	1	1	0	0	0	0	0	0	0	0	0	0	0	0	0	0	0	0	0	1	0	0	717
**300**	0	0	0	0	0	0	0	0	0	1	1	1	0	0	0	0	0	0	0	0	0	0	0	0	0	707
**301**	0	0	0	0	0	0	1	0	0	1	0	1	0	0	0	0	0	0	0	0	0	1	0	0	0	702
**302**	0	0	0	0	0	0	0	0	1	0	0	0	0	0	0	0	0	0	0	0	0	0	0	1	1	700
**303**	0	0	0	0	1	0	0	0	0	0	0	1	0	0	0	0	0	0	0	1	0	1	0	0	0	699
**304**	0	0	0	0	0	0	1	0	0	1	0	1	0	1	0	0	0	0	0	1	0	1	0	0	1	699
**305**	0	1	0	1	0	0	0	0	0	1	0	1	0	0	0	0	0	0	0	0	0	0	0	0	0	682
**306**	0	1	0	0	1	0	0	0	0	0	0	1	0	0	0	0	0	0	0	0	0	0	1	0	1	678
**307**	0	0	0	0	0	0	1	0	0	1	0	1	0	0	0	0	0	0	0	1	0	0	0	1	1	673
**308**	0	0	0	0	0	0	0	0	1	0	0	1	0	1	0	0	0	0	0	1	0	1	0	0	1	667
**309**	0	1	0	0	1	0	0	0	1	1	0	1	0	0	0	0	0	0	0	1	0	0	0	0	0	667
**310**	0	0	0	0	0	0	1	0	0	0	0	0	0	1	0	0	0	0	0	0	0	0	0	1	1	667
**311**	0	1	0	0	0	0	0	0	0	1	0	1	0	0	0	0	0	0	0	1	0	0	0	0	0	664
**312**	0	1	0	0	1	0	0	0	0	0	0	1	1	0	0	0	0	0	0	0	0	1	0	0	0	651
**313**	0	0	0	1	0	0	0	0	0	0	0	1	0	0	0	0	0	0	0	0	0	1	0	0	0	648
**314**	0	0	0	0	1	0	0	0	0	1	0	1	0	0	0	0	0	0	0	0	0	0	0	1	1	644
**315**	0	0	0	1	0	0	0	0	0	0	0	0	0	0	0	0	0	0	0	0	0	1	0	0	0	643
**316**	0	1	0	0	0	0	0	0	0	0	0	1	0	0	0	0	0	0	0	0	0	0	1	0	1	642
**317**	0	0	0	0	0	0	0	0	0	1	0	0	0	0	0	0	0	0	0	0	0	1	1	0	0	636
**318**	0	0	0	0	0	0	0	0	0	0	0	0	0	0	0	1	0	0	0	0	0	0	0	1	0	636
**319**	0	0	0	0	0	0	0	0	0	1	1	1	0	0	0	0	0	0	0	0	0	0	0	1	1	627
**320**	0	0	0	0	0	0	0	0	0	0	0	1	1	0	0	0	0	0	0	0	0	0	0	0	0	627
**321**	0	0	1	0	0	0	0	0	0	0	0	0	1	0	0	1	0	0	0	0	0	0	0	0	0	623
**322**	0	0	0	0	0	0	1	0	0	1	0	1	0	0	0	0	0	0	0	1	0	0	0	0	0	620
**323**	0	1	0	0	1	0	0	0	0	1	0	1	0	0	0	0	0	0	0	0	0	0	0	0	1	619
**324**	0	0	0	0	0	0	1	0	0	1	0	1	0	0	0	0	0	0	0	0	0	0	0	0	1	617
**325**	0	0	0	0	0	0	0	0	0	0	0	1	0	1	0	0	0	0	0	0	0	1	0	1	1	613
**326**	0	0	1	0	0	0	1	0	0	1	0	1	0	1	0	0	0	0	0	1	0	0	0	1	1	611
**327**	0	1	0	0	1	0	0	0	0	1	0	1	0	0	0	0	0	0	0	0	0	1	0	0	0	611
**328**	0	0	0	0	0	0	0	0	1	1	0	1	0	0	0	0	0	0	0	1	0	1	0	0	0	608
**329**	0	0	1	0	0	0	0	0	0	0	0	1	0	0	0	0	0	0	0	0	0	1	0	1	1	603
**330**	0	0	0	0	0	0	1	0	0	0	0	1	0	1	0	0	0	0	0	1	0	1	0	0	0	603
**331**	0	0	0	0	0	0	1	0	0	0	0	0	0	0	0	0	0	0	0	1	0	0	1	0	0	603
**332**	0	1	0	1	0	0	0	0	0	0	0	0	0	0	0	0	0	0	0	1	0	0	1	0	0	602
**333**	0	0	1	0	0	0	0	0	0	1	0	1	0	0	0	0	0	0	0	0	0	0	0	1	1	593
**334**	0	0	0	0	0	0	0	0	0	0	0	0	0	1	0	0	0	0	0	1	0	0	1	0	0	591
**335**	0	0	1	0	0	0	0	0	0	0	0	0	1	0	0	0	0	0	0	0	0	0	0	1	0	584
**336**	0	0	0	0	0	0	0	0	0	1	0	1	0	0	0	0	0	0	0	1	0	1	1	0	0	577
**337**	0	1	0	0	0	0	0	0	0	0	0	0	0	0	0	0	0	0	0	1	0	0	0	0	0	576
**338**	0	1	0	1	1	0	0	0	1	1	0	1	1	0	0	0	0	0	0	0	0	0	0	0	0	573
**339**	0	0	0	0	0	0	0	0	1	1	0	1	0	0	0	0	0	0	0	0	0	0	0	1	1	570
**340**	0	0	0	0	1	0	0	0	0	0	0	1	0	0	0	0	0	0	0	0	0	1	0	0	1	565
**341**	0	0	0	0	0	0	0	0	0	0	0	1	0	0	0	0	0	0	0	0	0	1	1	1	1	563
**342**	0	0	0	0	0	0	1	0	0	1	0	1	0	1	0	0	0	0	0	1	0	0	0	0	0	562
**343**	0	0	0	0	0	0	0	0	0	0	0	1	0	1	0	0	0	0	0	0	0	0	0	0	1	561
**344**	0	1	0	0	1	0	0	0	1	0	0	1	0	0	0	0	0	0	0	0	0	0	1	0	0	557
**345**	0	0	0	0	0	0	1	0	0	0	0	1	0	0	0	1	0	0	0	0	0	0	0	1	1	554
**346**	0	1	0	0	1	0	0	0	0	0	0	1	0	0	0	0	0	0	0	0	0	0	0	1	0	554
**347**	0	0	0	0	1	0	0	0	0	1	0	1	1	0	0	0	0	0	0	0	0	0	0	0	0	549
**348**	0	1	0	0	1	0	0	0	0	0	0	1	0	0	0	0	0	0	0	0	0	1	0	0	1	546
**349**	0	0	0	0	0	0	0	0	0	1	0	1	0	1	0	0	0	0	0	1	0	0	0	1	0	540
**350**	0	0	0	0	0	0	0	0	0	0	0	1	0	0	0	0	0	0	0	0	0	1	1	0	1	540
**351**	0	0	0	0	0	0	1	0	0	1	0	0	0	1	0	0	0	0	0	1	0	1	1	0	0	528
**352**	0	1	0	0	1	0	0	0	0	0	0	1	1	0	0	0	0	0	0	0	0	0	0	1	1	521
**353**	0	1	0	0	1	0	0	0	0	0	0	1	0	0	0	0	0	0	0	0	0	1	0	1	1	521
**354**	0	0	0	0	0	0	0	0	0	0	1	1	0	0	0	0	0	0	0	0	0	0	0	1	0	521
**355**	0	0	0	0	0	0	0	0	0	0	1	0	0	0	0	0	0	0	0	0	0	1	0	1	0	517
**356**	0	0	0	0	1	0	0	0	0	0	0	1	0	0	0	0	0	0	0	0	0	1	0	1	1	515
**357**	0	0	0	0	0	0	1	0	0	0	0	0	0	0	0	0	0	0	0	1	0	0	0	1	1	514
**358**	0	0	0	0	0	0	0	0	0	1	0	1	0	1	0	0	0	0	0	1	0	0	0	0	1	510
**359**	0	0	0	0	0	0	0	0	0	0	0	1	0	0	0	0	0	0	0	0	0	1	1	1	0	508
**360**	0	1	0	0	1	0	0	0	0	0	0	1	0	0	0	0	0	0	0	1	0	1	0	0	0	504
**361**	0	1	0	0	0	0	0	0	1	0	0	1	0	0	0	0	0	0	0	0	0	0	1	0	0	504
**362**	0	0	0	0	0	0	0	0	0	0	0	0	0	0	0	0	0	0	0	1	0	0	1	1	0	502
**363**	0	1	0	0	1	0	0	0	0	0	0	0	0	0	0	0	0	0	0	1	0	0	1	0	0	500
**364**	0	1	0	0	0	0	0	0	0	1	0	1	0	0	0	0	0	0	0	0	0	0	0	1	0	498
**365**	0	0	0	0	0	0	0	0	0	0	1	0	0	0	0	0	0	0	0	0	0	0	0	0	1	496

CMP	CD2	CD3	CD4	CD7	CD8	CD10	CD13	CD18	CD26	CD29	CD36	CD44	CD45RA	CD49f	CD54	CD56	CD57	CD58	CD62L	CD71	CD80	Cytokeratin	Histone_H1	HLA-DQ	HLA-DR	Freq. (voxel)
**366**	0	0	1	0	0	0	0	0	0	0	0	0	0	0	0	0	0	0	0	0	0	1	0	1	0	494
**367**	0	0	0	0	1	0	0	0	0	0	0	1	0	0	0	0	0	0	0	0	0	0	0	1	0	494
**368**	0	0	0	0	0	0	1	0	0	0	0	0	0	1	0	0	0	0	0	1	0	1	1	0	0	493
**369**	0	0	0	0	0	0	1	0	0	0	0	0	0	1	0	0	0	0	0	0	0	1	0	0	1	492
**370**	0	1	0	0	0	0	0	0	0	0	0	0	0	0	0	0	0	0	0	0	0	1	0	0	1	492
**371**	0	1	0	0	1	0	0	0	0	0	0	1	1	0	0	0	0	0	0	0	0	0	0	0	1	491
**372**	0	0	0	0	0	0	0	0	0	1	0	0	0	1	0	0	0	0	0	0	0	0	0	1	0	489
**373**	0	0	0	0	0	0	1	0	0	0	1	0	0	0	0	0	0	0	0	0	0	1	0	0	0	489
**374**	0	0	0	0	0	0	0	0	0	1	0	0	0	1	0	0	0	0	0	1	0	1	1	0	0	487
**375**	0	1	0	1	1	0	0	0	0	1	0	1	0	0	0	0	0	0	0	0	0	0	1	0	0	487
**376**	0	0	0	0	0	0	1	0	0	1	0	0	0	1	0	0	0	0	0	0	0	1	1	0	0	483
**377**	0	0	0	0	0	0	0	0	1	1	0	1	0	1	0	0	0	0	0	1	0	1	0	0	0	482
**378**	0	0	0	0	0	0	0	0	1	0	1	0	0	0	0	0	0	0	0	1	0	1	0	0	0	480
**379**	0	0	0	0	0	0	1	0	0	0	0	0	0	0	0	0	0	0	0	0	0	1	0	0	1	480
**380**	0	0	0	0	0	0	1	0	0	0	0	0	0	1	0	0	0	0	0	0	0	0	1	0	0	472
**381**	0	1	0	1	1	0	0	0	1	1	0	1	0	0	0	0	0	0	0	1	0	0	0	1	1	471
**382**	0	0	0	0	0	0	1	0	0	1	0	0	0	0	0	0	0	0	0	1	0	0	0	0	0	470
**383**	0	0	0	0	0	0	1	0	0	1	0	1	0	1	0	0	0	0	0	0	0	1	0	0	1	463
**384**	0	0	1	0	0	0	0	0	0	0	0	0	0	0	0	0	0	0	0	0	0	1	1	0	0	463
**385**	0	0	0	0	0	0	0	0	0	0	0	0	1	0	0	0	0	0	0	0	0	0	1	0	0	460
**386**	0	0	0	0	1	0	0	0	1	0	0	1	0	0	0	0	0	0	0	0	0	0	0	0	0	459
**387**	0	1	0	1	1	0	0	0	0	1	0	1	0	0	0	0	0	0	0	1	0	0	0	1	1	457
**388**	0	0	0	0	0	0	0	0	0	0	1	0	0	0	0	0	0	0	0	0	0	1	0	1	1	456
**389**	0	1	0	0	0	0	0	0	0	0	0	1	1	0	0	0	0	0	0	0	0	0	0	0	0	456
**390**	0	1	0	0	1	0	0	0	1	1	0	1	0	0	0	0	0	0	0	0	0	0	0	1	1	455
**391**	0	0	1	0	0	0	0	0	0	0	0	1	0	0	0	0	0	0	0	1	0	0	0	1	1	454
**392**	0	0	0	0	0	0	0	0	0	0	0	0	0	0	0	0	0	0	0	1	0	0	1	1	1	453
**393**	0	0	0	0	0	0	0	0	0	1	0	1	0	0	0	0	0	0	0	0	0	0	1	1	1	451
**394**	0	0	0	0	1	0	0	0	1	0	0	1	0	0	0	0	0	0	0	1	0	1	0	0	0	449
**395**	0	1	0	0	0	0	0	0	0	1	0	1	0	0	0	0	0	0	0	0	0	0	0	0	1	449
**396**	0	0	0	0	0	0	1	0	0	1	0	0	0	1	0	0	0	0	0	1	0	1	0	0	0	445
**397**	0	0	1	0	0	0	0	0	0	0	0	0	0	1	0	0	0	0	0	0	0	1	0	0	0	444
**398**	0	1	0	0	0	0	0	0	0	1	0	1	0	0	0	0	0	0	0	0	0	1	0	0	0	443
**399**	0	0	0	0	0	0	0	0	0	0	1	0	0	1	0	0	0	0	0	1	0	1	0	0	0	441
**400**	0	0	0	0	0	0	0	0	0	0	0	0	0	1	0	0	0	0	0	0	0	0	1	1	0	441
**401**	0	0	0	0	0	0	0	0	1	0	0	0	0	0	0	0	0	0	0	1	0	1	0	1	1	439
**402**	0	0	0	0	0	0	0	0	1	0	1	0	0	1	0	0	0	0	0	0	0	1	0	0	0	439
**403**	0	0	0	0	1	0	0	0	1	1	0	1	0	0	0	0	0	0	0	1	0	0	0	0	0	426
**404**	0	1	0	0	1	0	0	0	0	0	0	1	0	0	0	0	0	0	0	0	0	1	1	0	0	426
**405**	0	0	1	0	0	0	0	0	0	0	0	0	1	0	0	1	0	0	0	0	0	0	0	1	0	426
**406**	0	0	0	0	0	0	0	0	0	0	0	1	0	1	0	0	0	0	0	1	0	1	0	0	1	423
**407**	0	1	0	0	0	0	0	0	0	1	0	1	0	0	0	0	0	0	0	0	0	0	0	1	1	423
**408**	0	0	0	0	1	0	0	0	0	0	0	1	0	0	0	0	0	0	0	0	0	1	1	0	0	418
**409**	0	0	0	0	1	0	0	0	0	0	0	1	0	1	0	0	0	0	0	0	0	0	0	0	0	418
**410**	0	1	0	0	1	0	0	0	0	1	0	1	0	0	0	0	0	0	0	1	0	0	0	1	1	417
**411**	0	0	1	0	0	0	0	0	0	0	0	0	0	0	0	0	0	0	0	1	0	0	0	1	1	416
**412**	0	0	0	0	0	0	0	0	0	0	1	1	0	0	0	0	0	0	0	0	0	1	0	0	1	415
**413**	0	0	0	0	0	0	1	0	0	1	0	1	0	1	0	0	0	0	0	1	0	1	1	0	1	414
**414**	0	0	0	0	0	0	0	0	1	0	0	0	0	0	0	0	0	0	0	1	0	1	0	0	1	413
**415**	0	0	0	0	0	0	0	0	0	1	0	0	0	1	0	0	0	0	0	1	0	1	0	0	0	412
**416**	0	0	0	0	0	0	0	0	0	1	0	0	0	0	0	0	0	0	0	0	0	1	0	0	1	412
**417**	0	0	0	0	0	0	0	0	1	1	0	1	0	0	0	0	0	0	0	1	0	0	0	1	1	411
**418**	0	0	0	0	0	0	0	0	0	1	0	0	0	0	0	0	0	0	0	0	0	0	1	1	1	409
**419**	0	0	1	0	0	0	1	0	0	0	0	1	0	0	0	0	0	0	0	0	0	0	0	1	1	404
**420**	0	0	1	0	0	0	0	0	0	0	0	0	0	0	0	1	0	0	0	0	0	0	0	1	0	403
**421**	0	1	0	1	1	0	0	0	1	0	0	1	0	0	0	0	0	0	0	0	0	0	0	0	0	398
**422**	0	1	0	1	1	0	0	0	1	0	0	1	0	0	0	0	0	0	0	1	0	0	0	0	0	398
**423**	0	1	0	0	1	0	0	0	0	0	0	1	1	0	0	0	0	0	0	1	0	0	0	0	0	396
**424**	0	1	0	0	0	0	0	0	0	0	0	1	0	0	0	0	0	0	0	0	0	0	1	1	0	396
**425**	0	0	1	0	0	0	0	0	0	0	0	0	0	0	0	0	0	0	0	0	0	1	0	1	1	393
**426**	0	0	1	0	0	0	0	0	1	0	0	0	0	0	0	0	0	0	0	0	0	1	0	0	0	391
**427**	0	0	1	0	0	0	0	0	0	1	0	1	0	0	0	0	0	0	0	1	0	0	0	0	0	390
**428**	0	0	1	0	0	0	0	0	0	0	0	1	0	0	0	0	0	0	0	1	0	1	0	0	0	390
**429**	0	0	0	0	0	0	0	0	0	1	0	0	0	1	0	0	0	0	0	0	0	0	1	0	0	390
**430**	0	0	0	0	0	0	0	0	1	0	0	1	0	0	0	0	0	0	0	0	0	0	1	0	0	389
**431**	0	0	0	0	0	0	0	0	0	0	0	1	0	1	0	0	0	0	0	0	0	1	0	1	0	384
**432**	0	0	0	0	0	0	1	0	1	0	0	1	0	1	0	0	0	0	0	1	0	1	0	0	0	383
**433**	0	0	0	0	0	0	0	0	0	1	0	1	0	1	0	0	0	0	0	1	0	0	1	0	0	383
**434**	0	0	0	0	0	0	0	0	0	0	0	0	1	0	0	0	0	0	0	0	0	1	0	1	0	373
**435**	0	1	0	1	0	0	0	0	0	0	0	0	0	0	0	0	0	0	0	1	0	0	0	0	0	373
**436**	0	1	0	1	0	0	0	0	0	0	0	1	0	0	0	0	0	0	0	1	0	0	1	0	0	372
**437**	0	0	0	0	0	0	0	0	0	0	0	0	0	1	0	0	0	0	0	1	0	0	0	0	0	371
**438**	0	0	0	0	0	0	0	0	0	1	0	1	0	1	0	0	0	0	0	1	0	1	1	0	1	370
**439**	0	1	0	0	1	0	0	0	1	1	0	1	0	0	0	0	0	0	0	0	0	0	1	0	0	365
**440**	0	0	0	1	0	0	0	0	0	0	0	0	0	0	0	0	0	0	0	1	0	0	0	0	0	365
**441**	0	0	0	0	0	0	0	0	1	1	0	0	0	1	0	0	0	0	0	0	0	1	0	0	0	361
**442**	0	1	0	1	0	0	0	0	0	1	0	1	0	0	0	0	0	0	0	1	0	0	0	0	0	360
**443**	0	0	0	0	0	0	0	0	0	1	0	1	0	0	0	0	0	0	0	1	0	1	0	1	1	358
**444**	0	1	0	0	0	0	0	0	0	1	0	0	0	0	0	0	0	0	0	0	0	0	0	0	0	358
**445**	0	0	0	0	1	0	0	0	0	1	0	1	0	0	0	0	0	0	0	0	0	0	0	1	0	357
**446**	0	0	0	0	0	0	0	0	0	0	0	0	0	0	0	0	0	0	0	1	0	1	1	0	1	356
**447**	0	0	1	0	0	0	0	0	0	0	0	0	0	0	0	1	0	0	0	0	0	0	0	0	0	356
**448**	0	1	0	1	1	0	0	0	0	1	0	1	0	0	0	0	0	0	0	1	0	0	0	0	1	355
**449**	0	1	0	0	1	0	0	0	1	1	0	1	1	0	0	0	0	0	0	0	0	0	0	0	0	354
**450**	0	0	0	0	0	0	1	0	0	0	0	0	0	1	0	0	0	0	0	0	0	0	0	1	0	353
**451**	0	0	0	0	0	0	0	0	0	0	0	0	1	0	0	0	0	0	0	1	0	0	0	0	0	353
**452**	0	1	0	1	1	0	0	0	0	1	0	1	1	0	0	0	0	0	0	1	0	0	0	0	0	350
**453**	0	0	0	0	0	0	1	0	1	1	0	1	0	1	0	0	0	0	0	1	0	1	0	0	0	349
**454**	0	0	1	0	0	0	1	0	0	1	0	1	0	0	0	0	0	0	0	1	0	0	0	1	1	349
**455**	0	0	0	0	0	0	0	0	0	1	0	1	0	0	0	0	0	0	0	1	0	1	0	1	0	348
**456**	0	0	0	0	0	0	0	0	1	0	0	1	0	0	0	0	0	0	0	0	0	1	1	0	0	347
**457**	0	1	0	0	0	0	0	0	0	1	0	0	0	0	0	0	0	0	0	0	0	0	1	0	0	347
**458**	0	0	0	0	0	0	0	0	0	0	0	1	1	0	0	0	0	0	0	0	0	1	0	0	0	346
**459**	0	0	0	0	0	0	0	0	0	0	0	0	1	0	0	1	0	0	0	1	0	0	0	0	0	344
**460**	0	1	0	0	1	0	0	0	0	1	0	1	0	0	0	0	0	0	0	0	0	0	0	1	0	343
**461**	0	0	0	0	0	0	0	0	0	1	0	0	0	0	0	0	0	0	0	1	0	0	1	0	0	342
**462**	0	1	0	1	1	0	0	0	1	1	0	1	1	0	0	0	0	0	0	1	0	0	0	0	0	339
**463**	0	1	0	0	0	0	0	0	0	0	0	0	0	0	0	0	0	0	0	0	0	1	1	0	1	337
**464**	0	0	0	0	0	0	0	0	0	1	1	1	0	0	0	0	0	0	0	0	0	0	0	1	0	334
**465**	0	1	0	1	1	0	0	0	0	0	0	1	0	0	0	0	0	0	0	1	0	0	0	1	1	333
**466**	0	0	0	0	0	0	1	0	1	1	0	1	0	1	0	0	0	0	0	1	0	1	0	0	1	333
**467**	0	1	0	0	1	0	0	0	0	0	0	1	0	0	0	0	0	0	0	1	0	0	0	1	1	332
**468**	0	0	0	0	0	0	0	0	0	1	0	1	0	1	0	0	0	0	0	1	0	1	0	1	1	330
**469**	0	0	0	0	0	0	0	0	0	0	0	0	0	1	0	0	0	0	0	0	0	0	1	1	1	330
**470**	0	0	1	0	0	0	1	0	0	0	0	0	0	0	0	0	0	0	0	0	0	0	0	0	0	329
**471**	0	1	0	0	0	0	0	0	1	0	0	0	0	0	0	0	0	0	0	0	0	0	1	0	0	326
**472**	0	0	0	0	0	0	0	0	1	1	0	1	0	0	0	0	0	0	0	0	0	0	0	1	0	323
**473**	0	1	0	0	1	0	0	0	0	1	0	1	0	0	0	0	0	0	0	1	0	0	1	0	0	318
**474**	0	0	0	0	0	0	0	0	1	0	0	0	0	0	0	0	0	0	0	1	0	1	0	1	0	318
**475**	0	0	0	0	0	0	1	0	0	0	0	0	0	1	0	0	0	0	0	0	0	0	0	0	1	316
**476**	0	1	0	0	0	0	0	0	1	0	0	0	0	0	0	0	0	0	0	0	0	0	0	0	0	315
**477**	0	0	0	0	0	0	0	0	0	0	0	0	0	0	0	1	0	0	0	0	0	0	1	1	0	312
**478**	0	1	0	0	1	0	0	0	0	1	0	1	1	0	0	0	0	0	0	1	0	0	0	0	0	312
**479**	0	0	0	0	0	0	0	0	1	1	0	1	0	0	0	0	0	0	0	0	0	0	0	0	1	308
**480**	0	0	0	0	1	0	0	0	0	1	0	1	0	0	0	0	0	0	0	1	0	1	0	0	0	307
**481**	0	1	0	0	0	0	0	0	0	0	0	1	0	0	0	0	0	0	0	0	0	0	1	1	1	306
**482**	0	0	1	0	0	0	0	0	0	1	0	1	0	1	0	0	0	0	0	1	0	1	0	0	0	305
**483**	0	0	0	0	0	0	0	0	1	1	0	1	0	1	0	0	0	0	0	1	0	1	0	0	1	305
**484**	0	1	0	0	1	0	0	0	1	0	0	1	0	0	0	0	0	0	0	1	0	0	0	0	0	304
**485**	0	0	1	0	0	0	0	0	0	0	0	1	0	0	0	0	0	0	0	0	0	0	0	1	0	304
**486**	0	0	0	1	0	0	0	0	0	0	0	1	0	0	0	0	0	0	0	1	0	0	0	0	0	304
**487**	0	0	0	0	0	0	0	0	0	1	1	1	0	0	0	0	0	0	0	0	0	0	0	0	1	302

CMP	CD2	CD3	CD4	CD7	CD8	CD10	CD13	CD18	CD26	CD29	CD36	CD44	CD45RA	CD49f	CD54	CD56	CD57	CD58	CD62L	CD71	CD80	Cytokeratin	Histone_H1	HLA-DQ	HLA-DR	Freq. (voxel)
**488**	0	1	0	0	0	0	0	0	0	0	0	1	0	0	0	0	0	0	0	1	0	1	0	0	0	301
**489**	0	1	0	0	1	0	0	0	1	1	0	1	0	0	0	0	0	0	0	1	0	0	0	1	1	299
**490**	0	1	0	0	1	0	0	0	1	0	0	1	0	0	0	0	0	0	0	0	0	0	0	1	0	297
**491**	0	1	0	0	1	0	0	0	0	0	0	1	0	0	0	0	0	0	0	0	0	0	1	1	1	294
**492**	0	0	1	0	0	0	1	0	0	0	0	1	0	0	0	0	0	0	0	1	0	0	0	1	1	294
**493**	0	1	0	0	0	0	0	0	0	0	0	1	1	0	0	0	0	0	0	0	0	0	1	0	0	294
**494**	0	1	0	1	1	0	0	0	0	1	0	1	0	0	0	0	0	0	0	0	0	0	0	1	1	293
**495**	0	1	0	0	0	0	0	0	1	0	0	1	0	0	0	0	0	0	0	0	0	1	0	0	0	293
**496**	0	0	0	0	0	0	0	0	1	1	0	1	0	0	0	0	0	0	0	1	0	1	0	1	1	292
**497**	0	1	0	0	0	0	0	0	1	0	0	1	0	0	0	0	0	0	0	0	0	1	0	1	1	292
**498**	0	0	1	0	0	0	0	0	0	0	0	1	0	0	0	0	0	0	0	0	0	1	0	1	0	292
**499**	0	0	0	0	0	0	1	0	1	0	0	0	0	0	0	0	0	0	0	0	0	0	0	1	1	292
**500**	0	0	1	0	0	0	0	0	0	1	0	0	0	0	0	0	0	0	0	0	0	0	0	1	1	290
**501**	0	0	0	0	1	0	0	0	1	0	0	1	0	0	0	0	0	0	0	0	0	0	0	1	1	289
**502**	0	0	0	1	1	0	0	0	1	1	0	1	0	0	0	0	0	0	0	0	0	0	0	0	0	288
**503**	0	0	0	0	1	0	0	0	0	0	0	0	0	0	0	0	0	0	0	1	0	0	1	0	0	287
**504**	0	0	0	0	0	0	0	0	0	1	0	0	0	0	0	0	0	0	0	1	0	0	0	1	0	284
**505**	0	0	0	0	0	0	0	0	0	0	1	0	0	0	0	0	0	0	0	1	0	0	0	0	0	284
**506**	0	1	0	1	1	0	0	0	0	0	0	1	1	0	0	0	0	0	0	0	0	1	0	0	0	283
**507**	0	0	0	1	1	0	0	0	0	0	0	1	0	0	0	0	0	0	0	1	0	0	0	0	0	282
**508**	0	0	0	0	1	0	0	0	0	0	0	1	0	0	0	0	0	0	0	1	0	1	1	0	0	281
**509**	0	1	0	1	1	0	0	0	0	0	0	1	0	0	0	0	0	0	0	1	0	0	0	0	1	281
**510**	0	1	0	0	0	0	0	0	0	0	0	0	0	0	0	0	0	0	0	0	0	0	0	1	1	281
**511**	0	0	0	0	0	0	0	0	0	0	0	0	0	0	0	1	0	0	0	0	0	0	0	0	1	281
**512**	0	0	0	0	0	0	1	0	0	0	0	1	0	1	0	0	0	0	0	1	0	1	1	0	0	280
**513**	0	0	1	0	0	0	1	0	0	0	0	0	0	0	0	0	0	0	0	1	0	0	0	0	0	280
**514**	0	1	0	0	1	0	0	0	0	1	0	1	1	0	0	0	0	0	0	0	0	0	1	0	0	280
**515**	0	1	0	1	0	0	0	0	1	1	0	1	0	0	0	0	0	0	0	0	0	0	0	0	0	278
**516**	0	0	0	0	1	0	0	0	0	1	0	1	0	1	0	0	0	0	0	0	0	1	0	0	0	277
**517**	0	0	0	0	0	0	0	0	1	0	0	1	0	0	0	0	0	0	0	1	0	0	0	1	1	277
**518**	0	0	0	0	0	0	1	0	1	0	0	0	0	1	0	0	0	0	0	1	0	1	0	0	0	276
**519**	0	0	1	0	0	0	0	0	0	0	0	0	0	1	0	0	0	0	0	0	0	0	0	1	1	275
**520**	0	1	0	0	1	0	0	0	0	0	0	1	0	0	0	0	0	0	0	1	0	0	0	0	1	274
**521**	0	1	0	0	1	0	0	0	1	0	0	1	0	0	0	0	0	0	0	0	0	1	0	0	0	274
**522**	0	0	1	0	0	0	0	0	0	0	0	0	0	0	0	1	0	0	0	0	0	1	0	0	0	273
**523**	0	1	0	1	1	0	0	0	0	0	0	0	0	0	0	0	0	0	0	1	0	0	1	0	0	270
**524**	0	0	0	0	0	0	1	0	1	0	0	1	0	1	0	0	0	0	0	0	0	1	0	0	0	265
**525**	0	1	0	0	0	0	0	0	1	0	0	1	0	0	0	0	0	0	0	1	0	0	0	0	0	265
**526**	0	0	0	0	1	0	0	0	0	0	0	0	1	0	0	0	0	0	0	0	0	0	0	0	0	265
**527**	0	1	0	0	1	0	0	0	0	0	0	0	0	0	0	0	0	0	0	0	0	0	1	0	1	264
**528**	0	0	0	0	1	0	0	0	0	0	0	1	1	0	0	0	0	0	0	0	0	1	0	0	0	263
**529**	0	0	0	0	0	0	0	0	0	0	0	0	0	0	0	0	0	0	0	1	0	0	1	0	1	263
**530**	0	0	0	0	0	0	0	0	0	0	0	0	1	0	0	1	0	0	0	0	0	1	0	0	0	262
**531**	0	0	0	0	0	0	0	0	0	0	1	1	0	1	0	0	0	0	0	0	0	1	0	0	0	261
**532**	0	0	0	0	0	0	0	0	0	0	0	1	0	0	0	1	0	0	0	0	0	0	0	0	0	261
**533**	0	1	0	1	1	0	0	0	1	1	0	1	0	0	0	0	0	0	0	0	0	0	1	0	0	260
**534**	0	1	0	0	1	0	0	0	1	0	0	1	1	0	0	0	0	0	0	0	0	0	0	0	0	260
**535**	0	0	0	0	1	0	0	0	0	0	0	0	0	0	0	0	0	0	0	0	0	0	1	0	1	260
**536**	0	1	0	0	0	0	0	0	0	1	0	1	0	0	0	0	0	0	0	1	0	0	1	0	0	259
**537**	0	0	0	0	0	0	0	0	0	0	0	0	1	0	0	0	0	0	0	1	0	0	0	1	0	257
**538**	0	0	1	0	0	0	1	0	0	1	0	1	0	0	0	0	0	0	0	0	0	0	0	1	1	256
**539**	0	1	0	1	1	0	0	0	0	0	0	1	1	0	0	0	0	0	0	1	0	0	0	0	1	254
**540**	0	0	0	0	0	0	1	0	0	1	0	1	0	1	0	0	0	0	0	1	0	0	0	0	1	254
**541**	0	0	0	0	0	0	0	0	1	0	0	1	0	0	0	0	0	0	0	0	0	0	0	1	0	254
**542**	0	0	0	0	0	0	1	0	0	0	0	0	0	0	0	0	0	0	0	0	0	0	1	1	1	253
**543**	0	0	0	0	0	0	0	0	1	0	0	1	0	0	0	0	0	0	0	0	0	0	0	0	1	253
**544**	0	0	0	0	0	0	0	0	0	0	0	0	0	0	0	1	0	0	0	0	0	0	1	1	1	253
**545**	0	0	0	0	0	0	0	0	0	1	0	1	0	1	0	0	0	0	0	1	0	1	0	1	0	252
**546**	0	1	0	1	1	0	0	0	0	0	0	1	0	0	0	0	0	0	0	0	0	0	0	0	1	252
**547**	0	0	0	0	0	0	0	0	0	0	0	0	0	1	0	0	0	0	0	1	0	0	0	1	1	252
**548**	0	1	0	0	1	0	0	0	1	1	0	1	1	0	0	0	0	0	0	1	0	0	0	0	0	251
**549**	0	0	1	0	0	0	0	0	0	0	0	1	0	0	0	0	0	0	0	1	0	0	0	0	0	251
**550**	0	0	0	0	0	0	0	0	0	1	0	0	0	0	0	0	0	0	0	1	0	0	0	1	1	251
**551**	0	1	0	0	0	0	0	0	0	0	0	1	0	0	0	0	0	0	0	0	0	1	1	1	1	249
**552**	0	0	0	0	1	0	0	0	0	0	0	1	0	0	0	0	0	0	0	1	0	0	1	0	0	249
**553**	0	0	0	0	0	0	0	0	0	1	0	1	0	1	0	0	0	0	0	0	0	1	0	1	1	249
**554**	0	0	0	0	1	0	0	0	0	1	0	1	0	0	0	0	0	0	0	1	0	0	0	0	1	248
**555**	0	1	0	1	1	0	0	0	0	1	0	1	0	0	0	0	0	0	0	0	0	0	0	0	1	247
**556**	0	0	0	0	0	0	0	0	0	0	1	0	0	0	0	1	0	0	0	0	0	1	0	0	0	247
**557**	0	1	0	1	0	0	0	0	0	1	0	1	0	0	0	0	0	0	0	0	0	0	1	0	0	246
**558**	0	0	1	0	0	0	0	0	0	0	0	0	0	0	0	0	0	0	0	0	0	1	0	0	1	246
**559**	0	0	1	0	0	0	1	0	1	1	0	1	0	1	0	0	0	0	0	1	0	0	0	1	1	243
**560**	0	0	1	0	0	0	1	0	0	0	0	1	0	1	0	0	0	0	0	0	0	0	0	1	1	242
**561**	0	0	0	1	0	0	0	0	0	0	0	0	0	0	0	0	0	0	0	1	0	0	1	0	0	242
**562**	0	1	0	0	0	0	0	0	0	0	0	1	0	1	0	0	0	0	0	0	0	0	0	0	0	241
**563**	0	0	0	0	0	0	0	0	0	0	0	0	1	0	0	0	0	0	0	1	0	1	0	0	0	241
**564**	0	0	1	0	0	0	0	0	0	1	0	0	0	0	0	0	0	0	0	0	0	0	0	1	0	240
**565**	0	0	0	0	1	0	0	0	0	1	0	1	0	0	0	0	0	0	0	1	0	0	0	1	1	240
**566**	0	0	0	0	0	0	1	0	0	0	1	0	0	1	0	0	0	0	0	1	0	1	0	0	0	239
**567**	0	0	1	0	0	0	1	0	1	1	0	1	0	1	0	0	0	0	0	1	0	1	0	0	1	237
**568**	0	1	0	0	1	0	0	0	1	1	0	1	0	0	0	0	0	0	0	0	0	0	0	0	1	237
**569**	0	1	0	0	0	0	0	0	0	0	0	1	0	0	0	0	0	0	0	0	0	1	0	1	0	237
**570**	0	0	0	0	0	0	1	0	0	0	0	0	0	1	0	0	0	0	0	1	0	0	1	0	0	235
**571**	0	0	0	0	0	0	0	0	0	0	1	1	0	0	0	0	0	0	0	0	0	0	0	0	1	235
**572**	0	1	0	0	0	0	0	0	1	0	0	1	0	0	0	0	0	0	0	1	0	1	0	0	0	233
**573**	0	0	1	0	0	0	0	0	0	0	0	0	0	0	0	0	0	0	0	1	0	0	0	1	0	233
**574**	0	0	0	0	0	0	0	0	1	0	0	1	0	0	0	0	0	0	0	1	0	1	1	0	0	233
**575**	0	0	0	0	0	0	1	0	1	0	0	1	0	1	0	0	0	0	0	1	0	1	0	0	1	232
**576**	0	1	0	1	1	0	0	0	0	1	0	1	0	0	0	0	0	0	0	1	0	0	1	0	0	232
**577**	0	0	0	0	0	0	0	0	0	1	0	1	0	1	0	0	0	0	0	0	0	1	0	1	0	232
**578**	0	0	0	0	0	0	0	0	0	1	1	1	0	1	0	0	0	0	0	0	0	0	0	1	1	231
**579**	0	0	0	0	0	0	0	0	0	1	1	0	0	1	0	0	0	0	0	0	0	1	0	0	0	230
**580**	0	0	0	0	1	0	0	0	0	0	0	1	0	0	0	0	0	0	0	0	0	0	1	0	1	230
**581**	0	1	0	0	1	0	0	0	1	0	0	1	0	0	0	0	0	0	0	0	0	1	0	1	1	229
**582**	0	0	0	1	1	0	0	0	0	0	0	0	0	0	0	0	0	0	0	0	0	0	1	0	0	228
**583**	0	1	0	0	0	0	0	0	0	0	0	1	0	0	0	0	0	0	0	1	0	0	0	1	1	225
**584**	0	0	1	0	0	0	0	0	0	0	1	0	0	0	0	0	0	0	0	0	0	1	0	0	0	225
**585**	0	0	0	0	0	0	0	0	1	1	0	0	0	0	0	0	0	0	0	0	0	1	0	0	0	223
**586**	0	0	0	0	0	0	0	0	1	1	0	1	0	1	0	0	0	0	0	0	0	1	0	0	0	222
**587**	0	0	0	0	0	0	0	0	1	1	0	1	0	0	0	0	0	0	0	0	0	1	0	1	0	222
**588**	0	0	0	0	0	0	1	0	0	0	0	1	0	0	0	0	0	0	0	0	0	0	1	0	0	222
**589**	0	1	0	0	1	0	0	0	0	1	0	1	1	0	0	0	0	0	0	0	0	0	0	1	1	221
**590**	0	1	0	1	1	0	0	0	1	1	0	1	0	0	0	0	0	0	0	1	0	0	1	0	0	220
**591**	0	1	0	0	0	0	1	0	0	0	0	0	0	0	0	0	0	0	0	0	0	0	1	0	0	220
**592**	0	0	0	0	0	0	0	0	1	0	0	0	0	0	0	0	0	0	0	1	0	1	1	0	0	220
**593**	0	0	0	0	0	0	1	0	1	0	0	1	0	0	0	0	0	0	0	1	0	0	0	1	1	219
**594**	0	0	0	0	1	0	0	0	0	1	0	0	0	0	0	0	0	0	0	0	0	0	0	0	0	218
**595**	0	0	0	0	0	0	1	0	0	1	0	0	0	0	0	0	0	0	0	0	0	1	0	0	0	218
**596**	0	0	0	0	0	0	0	0	0	1	0	1	1	0	0	0	0	0	0	0	0	0	0	0	0	216
**597**	0	0	0	0	0	0	1	0	0	1	0	1	0	1	0	0	0	0	0	1	0	0	1	0	0	215
**598**	0	0	0	0	0	0	0	0	1	1	0	1	0	0	0	0	0	0	0	0	0	1	0	0	1	215
**599**	0	0	0	0	0	0	1	0	0	1	0	1	0	1	0	0	0	0	0	0	0	0	1	0	0	215
**600**	0	1	0	0	0	0	0	0	0	0	0	1	0	1	0	0	0	0	0	0	0	0	1	0	0	215
**601**	0	1	0	0	1	0	0	0	0	0	0	0	0	0	0	0	0	0	0	0	0	0	0	0	1	214
**602**	0	0	0	0	0	0	0	0	0	0	0	0	1	0	0	1	0	0	0	0	0	0	1	0	0	214
**603**	0	0	0	0	1	0	0	0	0	0	0	0	0	0	0	0	0	0	0	1	0	0	0	0	0	213
**604**	0	0	1	0	0	0	0	0	0	1	0	1	0	0	0	0	0	0	0	0	0	1	0	0	0	213
**605**	0	0	1	0	0	0	1	0	0	1	0	1	0	1	0	0	0	0	0	1	0	1	0	0	0	211
**606**	0	1	0	0	1	0	0	0	0	0	0	0	0	0	0	0	0	0	0	0	0	1	0	0	0	211
**607**	0	0	0	0	0	0	0	0	0	0	0	0	1	0	0	1	0	0	0	1	0	0	0	1	0	210
**608**	0	0	0	0	0	0	1	0	0	1	0	1	0	0	0	0	0	0	0	1	0	1	0	0	0	209
**609**	0	0	0	0	0	0	1	0	0	0	0	1	0	1	0	0	0	0	0	0	0	0	0	1	0	208

CMP	CD2	CD3	CD4	CD7	CD8	CD10	CD13	CD18	CD26	CD29	CD36	CD44	CD45RA	CD49f	CD54	CD56	CD57	CD58	CD62L	CD71	CD80	Cytokeratin	Histone_H1	HLA-DQ	HLA-DR	Freq. (voxel)
**610**	0	0	0	0	0	0	0	0	0	0	0	1	0	1	0	0	0	0	0	1	0	0	0	0	0	208
**611**	0	0	0	1	0	0	1	0	0	1	0	1	0	1	0	0	0	0	0	0	0	1	0	0	0	207
**612**	0	0	0	0	1	0	0	0	0	0	0	0	0	0	0	0	0	0	0	0	0	1	1	0	0	207
**613**	0	0	0	0	0	0	1	0	0	0	0	1	0	1	0	0	0	0	0	0	0	0	0	0	1	206
**614**	0	0	0	0	0	0	0	0	0	1	1	1	0	1	0	0	0	0	0	0	0	1	0	0	0	206
**615**	0	1	0	1	1	0	0	0	1	0	0	1	0	0	0	0	0	0	0	0	0	0	1	0	0	206
**616**	0	0	0	0	0	0	0	0	1	1	0	1	0	0	0	0	0	0	0	0	0	1	0	1	1	205
**617**	0	1	0	1	1	0	0	0	1	1	0	1	0	0	0	0	0	0	0	1	0	0	0	0	1	205
**618**	0	1	0	1	1	0	0	0	1	0	0	1	1	0	0	0	0	0	0	1	0	0	0	0	0	205
**619**	0	1	0	1	1	0	0	0	0	0	0	1	0	0	0	0	0	0	0	0	0	1	0	0	0	205
**620**	0	1	0	0	0	0	0	0	0	0	0	1	1	0	0	0	0	0	0	0	0	1	0	0	0	204
**621**	0	0	0	0	0	0	1	0	0	0	0	1	0	0	0	0	0	0	0	0	0	1	0	1	1	202
**622**	0	1	0	1	0	0	0	0	0	0	0	1	0	0	0	0	0	0	0	0	0	1	0	0	0	202
**623**	0	0	0	1	1	0	0	0	0	1	0	1	0	0	0	0	0	0	0	1	0	0	0	0	0	201
**624**	0	0	0	0	0	0	0	0	1	0	0	1	0	1	0	0	0	0	0	0	0	1	0	0	1	200
**625**	0	0	0	1	1	0	0	0	0	0	0	1	0	0	0	0	0	0	0	0	0	0	0	0	1	200
**626**	0	0	0	0	0	0	1	0	0	0	0	0	0	0	0	0	0	0	0	0	0	1	0	1	1	200
**627**	0	0	0	0	0	0	0	0	1	1	0	1	0	1	0	0	0	0	0	1	0	1	0	1	1	199
**628**	0	1	0	0	0	0	0	0	1	1	0	1	0	0	0	0	0	0	0	1	0	0	0	0	0	199
**629**	0	1	0	0	1	0	0	0	0	0	0	1	1	0	0	0	0	0	0	0	0	1	0	1	1	198
**630**	0	0	1	0	0	0	1	0	0	0	0	0	0	0	0	0	0	0	0	0	0	0	0	1	1	197
**631**	0	0	0	1	1	0	0	0	0	0	0	1	0	0	0	0	0	0	0	0	0	0	1	0	0	197
**632**	0	0	0	0	0	0	0	0	0	0	0	1	0	0	0	1	0	0	0	0	0	0	0	1	0	197
**633**	0	0	0	0	0	0	0	0	1	1	0	1	0	0	0	0	0	0	0	1	0	1	0	0	1	194
**634**	0	1	0	0	0	0	0	0	0	0	0	0	0	0	0	0	0	0	0	0	0	0	1	1	1	193
**635**	0	1	0	0	1	0	0	0	1	0	0	1	0	0	0	0	0	0	0	1	0	0	0	1	1	191
**636**	0	0	0	0	0	0	1	0	1	0	0	0	0	0	0	0	0	0	0	0	0	1	0	0	0	190
**637**	0	0	0	0	0	0	0	0	0	0	0	0	1	0	0	1	0	0	0	0	0	1	0	1	0	190
**638**	0	0	0	0	0	0	0	0	1	0	0	0	0	1	0	0	0	0	0	0	0	1	1	0	0	189
**639**	0	0	0	0	0	0	0	0	0	1	0	1	0	1	0	0	0	0	0	0	0	0	1	0	1	188
**640**	0	0	0	0	0	0	1	0	0	1	1	1	0	1	0	0	0	0	0	0	0	0	0	1	1	187
**641**	0	0	1	1	0	0	1	0	1	1	0	1	1	1	0	1	0	0	0	1	0	0	0	1	1	186
**642**	0	0	0	0	0	0	1	0	0	1	0	1	0	1	0	0	0	0	0	1	0	0	0	1	0	186
**643**	0	0	1	0	0	0	0	0	0	1	0	1	0	1	0	0	0	0	0	0	0	0	0	1	1	186
**644**	0	0	0	0	0	0	0	0	0	1	0	0	0	1	0	0	0	0	0	1	0	0	0	0	0	186
**645**	0	0	0	1	0	0	0	0	1	0	0	1	0	0	0	0	0	0	0	0	0	0	0	0	0	186
**646**	0	0	0	1	0	0	0	0	0	1	0	1	0	0	0	0	0	0	0	1	0	0	0	0	0	185
**647**	0	0	1	0	0	0	0	0	0	0	0	0	0	0	0	0	0	0	0	0	0	0	1	1	0	185
**648**	0	0	0	0	0	0	0	0	0	0	1	1	0	0	0	0	0	0	0	0	0	1	0	1	1	184
**649**	0	1	0	0	1	0	0	0	0	0	0	0	1	0	0	0	0	0	0	0	0	0	1	0	0	184
**650**	0	1	0	0	1	0	0	0	0	0	0	1	0	0	0	0	0	0	0	1	0	1	0	0	1	183
**651**	0	0	0	0	0	0	0	0	1	0	1	0	0	1	0	0	0	0	0	1	0	1	0	0	0	182
**652**	0	1	0	0	1	0	0	0	1	1	0	1	0	0	0	0	0	0	0	0	0	0	0	1	0	182
**653**	0	1	0	1	1	0	0	0	1	0	0	1	1	0	0	0	0	0	0	0	0	0	0	0	0	182
**654**	0	1	0	0	1	0	0	0	0	0	0	1	0	1	0	0	0	0	0	0	0	0	0	0	0	182
**655**	0	0	0	0	1	0	0	0	0	0	0	0	0	0	0	0	0	0	0	0	0	1	0	0	1	181
**656**	0	1	0	1	1	0	0	0	1	1	0	1	0	0	0	0	0	0	0	0	0	0	0	1	1	180
**657**	0	0	0	0	0	0	1	0	0	0	0	1	0	0	0	0	0	0	0	1	0	0	0	0	1	180
**658**	0	1	0	1	1	0	0	0	1	0	0	1	0	0	0	0	0	0	0	1	0	1	0	0	0	180
**659**	0	1	0	1	1	0	0	0	0	0	0	1	0	0	0	0	0	0	0	0	0	0	0	1	1	180
**660**	0	1	0	0	1	0	0	0	1	0	0	1	1	0	0	0	0	0	0	0	0	0	0	1	1	179
**661**	0	0	0	0	0	0	0	0	0	1	0	0	0	1	0	0	0	0	0	0	0	0	0	1	1	179
**662**	0	1	0	0	1	0	0	0	0	1	0	1	0	1	0	0	0	0	0	0	0	0	0	0	0	178
**663**	0	0	0	0	0	0	1	0	0	0	0	1	0	1	0	0	0	0	0	0	0	1	0	0	1	177
**664**	0	0	1	0	0	0	0	0	0	0	0	0	0	1	0	0	0	0	0	0	0	0	0	0	0	177
**665**	0	1	0	0	1	0	0	0	1	0	0	0	0	0	0	0	0	0	0	0	0	0	1	0	0	177
**666**	0	1	0	0	0	0	0	0	0	0	0	1	0	0	0	0	0	0	0	0	0	1	1	0	1	176
**667**	0	0	0	0	0	0	0	0	1	0	0	1	0	0	0	0	0	0	0	1	0	0	0	0	1	176
**668**	0	0	0	0	1	0	0	0	1	0	0	1	0	0	0	0	0	0	0	0	0	1	0	0	0	176
**669**	0	1	0	0	0	0	0	0	0	0	0	1	0	0	0	0	0	0	0	1	0	1	0	1	1	175
**670**	0	0	1	0	0	0	0	0	0	0	0	0	0	0	0	0	0	0	0	0	0	0	1	1	1	175
**671**	0	0	0	0	0	0	0	0	0	0	0	1	0	1	0	0	0	0	0	1	0	1	0	1	1	174
**672**	0	1	0	0	0	0	0	0	0	1	0	1	0	1	0	0	0	0	0	0	0	0	0	0	0	174
**673**	0	1	0	1	1	0	0	0	1	0	0	1	0	0	0	0	0	0	0	1	0	1	0	1	1	173
**674**	0	0	0	1	0	0	0	0	1	1	0	1	0	0	0	0	0	0	0	0	0	0	0	0	0	173
**675**	0	0	1	0	0	0	0	0	1	0	0	1	0	0	0	0	0	0	0	0	0	1	0	0	0	173
**676**	0	1	0	0	1	0	0	0	1	0	0	1	0	0	0	0	0	0	0	1	0	1	0	0	0	172
**677**	0	0	0	0	0	0	0	0	1	0	0	1	0	1	0	0	0	0	0	0	0	1	0	1	1	171
**678**	0	0	1	0	0	0	0	0	0	1	0	1	0	1	0	0	0	0	0	1	0	1	0	0	1	170
**679**	0	0	0	0	0	0	0	0	0	0	0	0	0	1	0	1	0	0	0	0	0	1	0	0	0	170
**680**	0	0	1	0	0	0	0	0	1	0	0	1	0	0	0	0	0	0	0	1	0	1	0	0	0	170
**681**	0	1	0	0	1	0	0	0	1	0	0	1	1	0	0	0	0	0	0	0	0	1	0	0	0	170
**682**	0	0	0	0	0	0	0	0	1	0	0	0	1	0	0	0	0	0	0	1	0	1	0	0	0	170
**683**	0	0	0	0	0	0	1	0	0	1	1	0	0	1	0	0	0	0	0	0	0	1	0	0	0	169
**684**	0	1	0	0	0	0	0	0	1	0	0	1	0	0	0	0	0	0	0	0	0	0	0	1	0	169
**685**	0	1	0	0	0	0	0	0	0	0	0	1	0	1	0	0	0	0	0	0	0	0	0	1	1	168
**686**	0	1	0	0	1	0	0	0	0	1	0	1	0	0	0	0	0	0	0	1	0	0	0	0	1	168
**687**	0	1	0	0	0	0	0	0	1	0	0	1	0	0	0	0	0	0	0	0	0	0	0	1	1	168
**688**	0	1	0	1	0	0	0	0	0	0	0	1	1	0	0	0	0	0	0	0	0	0	0	0	0	168
**689**	0	0	1	0	0	0	0	0	1	0	0	0	0	0	0	0	0	0	0	0	0	0	0	0	0	167
**690**	0	1	0	1	1	0	0	0	0	0	0	1	1	0	0	0	0	0	0	0	0	0	0	1	1	167
**691**	0	0	0	0	0	0	0	0	1	0	0	0	1	0	0	1	0	0	0	1	0	0	0	0	0	167
**692**	0	0	0	0	0	0	0	0	1	0	0	0	0	1	0	0	0	0	0	1	0	1	1	0	0	166
**693**	0	1	0	1	1	0	0	0	1	0	0	1	0	0	0	0	0	0	0	1	0	0	1	0	0	166
**694**	0	0	0	0	0	0	0	0	1	0	1	1	0	0	0	0	0	0	0	0	0	1	0	0	0	165
**695**	0	0	0	0	0	0	1	0	0	1	0	1	0	1	0	0	0	0	0	0	0	0	1	1	1	163
**696**	0	1	0	0	1	0	0	0	0	0	0	1	0	0	0	0	0	0	0	0	0	0	1	1	0	163
**697**	0	1	0	1	1	0	0	0	0	0	0	1	0	0	0	0	0	0	0	1	0	1	0	0	0	163
**698**	0	0	0	0	0	0	0	0	0	1	0	0	0	0	0	0	0	0	0	0	0	1	0	1	0	163
**699**	0	0	0	0	1	0	0	0	0	0	0	1	0	0	0	0	0	0	0	1	0	0	0	1	1	162
**700**	0	0	1	0	0	0	0	0	0	0	0	1	0	0	0	0	0	0	0	1	0	1	0	0	1	162
**701**	0	1	0	0	1	0	0	0	0	0	0	0	0	0	0	0	0	0	0	1	0	0	0	0	0	162
**702**	0	0	1	0	0	0	0	0	0	0	0	1	0	1	0	0	0	0	0	0	0	1	0	0	0	162
**703**	0	0	1	0	0	0	0	0	0	0	0	1	0	0	0	0	0	0	0	0	0	1	0	0	1	162
**704**	0	1	0	1	0	0	0	0	0	0	0	1	0	0	0	0	0	0	0	1	0	0	0	0	1	162
**705**	0	0	1	0	0	0	0	0	0	1	0	1	0	0	0	0	0	0	0	1	0	1	0	0	0	161
**706**	0	1	0	1	1	0	0	0	1	0	0	1	0	0	0	0	0	0	0	1	0	1	0	0	1	161
**707**	0	0	1	0	0	0	1	0	0	1	0	1	0	1	0	0	0	0	0	1	0	1	0	0	1	160
**708**	0	1	0	1	1	0	0	0	0	0	0	0	0	0	0	0	0	0	0	0	0	0	0	0	0	160
**709**	0	1	0	0	0	0	0	0	0	1	0	1	1	0	0	0	0	0	0	0	0	0	0	0	0	160
**710**	0	0	0	0	0	0	1	0	1	1	0	1	0	0	0	0	0	0	0	0	0	0	0	1	1	159
**711**	0	0	0	1	0	0	0	0	0	1	0	1	0	0	0	0	0	0	0	0	0	1	0	0	0	159
**712**	0	1	0	1	1	0	0	0	0	0	0	0	0	0	0	0	0	0	0	1	0	0	0	0	0	159
**713**	0	1	0	0	0	0	0	0	0	1	0	1	0	0	0	0	0	0	0	1	0	0	0	1	0	158
**714**	0	0	1	0	0	0	0	0	0	1	0	1	0	1	0	0	0	0	0	0	0	1	0	0	0	158
**715**	0	0	0	0	0	0	1	0	0	1	0	0	0	1	0	0	0	0	0	1	0	0	0	0	0	158
**716**	0	1	0	0	1	0	1	0	0	1	0	1	0	0	0	0	0	0	0	0	0	0	0	0	0	158
**717**	0	1	0	1	1	0	0	0	0	0	0	1	1	0	0	0	0	0	0	1	0	0	0	1	1	157
**718**	0	1	0	0	1	0	0	0	0	0	0	1	1	0	0	0	0	0	0	1	0	0	0	0	1	157
**719**	0	0	0	1	1	0	0	0	0	0	0	0	0	0	0	0	0	0	0	0	0	0	0	0	0	157
**720**	0	1	0	0	0	0	0	0	0	0	0	1	0	0	0	0	0	0	0	1	0	1	1	0	0	157
**721**	0	0	0	0	1	0	0	0	0	0	0	0	0	0	0	0	0	0	0	0	0	0	0	1	1	156
**722**	0	1	0	0	1	0	0	0	0	0	0	1	1	0	0	0	0	0	0	0	0	0	1	0	1	155
**723**	0	1	0	0	1	0	0	0	1	0	0	1	1	0	0	0	0	0	0	0	0	0	1	0	0	155
**724**	0	1	0	0	0	0	0	0	0	1	0	1	0	0	0	0	0	0	0	0	0	1	1	0	0	155
**725**	0	0	0	0	0	0	0	0	1	0	0	1	0	1	0	0	0	0	0	1	0	1	0	1	1	154
**726**	0	0	0	0	0	0	0	0	1	0	0	0	0	0	0	0	0	0	0	1	0	0	0	1	1	154
**727**	0	0	1	0	0	0	0	0	1	0	0	1	0	0	0	0	0	0	0	0	0	1	0	1	1	153
**728**	0	1	0	1	0	0	0	0	1	1	0	1	0	0	0	0	0	0	0	1	0	0	0	0	0	153
**729**	0	1	0	0	0	0	0	0	0	0	0	0	0	0	0	0	0	0	0	1	0	0	1	0	1	153
**730**	0	1	0	0	1	0	0	0	0	1	0	1	1	0	0	0	0	0	0	0	0	1	0	0	0	153
**731**	0	1	0	0	0	0	0	0	1	0	0	1	0	0	0	0	0	0	0	1	0	1	0	0	1	152

CMP	CD2	CD3	CD4	CD7	CD8	CD10	CD13	CD18	CD26	CD29	CD36	CD44	CD45RA	CD49f	CD54	CD56	CD57	CD58	CD62L	CD71	CD80	Cytokeratin	Histone_H1	HLA-DQ	HLA-DR	Freq. (voxel)
732	0	0	0	0	0	0	0	0	0	0	1	1	0	0	0	0	0	0	0	0	0	1	0	1	0	152
733	0	0	0	0	0	0	0	0	0	0	0	1	0	0	0	0	0	0	0	1	0	1	1	1	1	151
734	0	0	1	0	0	0	1	0	0	0	0	0	0	1	0	0	0	0	0	0	0	1	0	0	0	151
735	0	0	0	0	0	0	1	0	1	0	0	0	1	0	0	0	0	0	0	1	0	1	0	0	0	151
736	0	1	0	0	0	0	0	0	0	0	0	1	0	0	0	0	0	0	0	0	0	1	1	1	0	150
737	0	1	0	0	0	0	0	0	0	1	0	1	0	1	0	0	0	0	0	0	0	0	1	0	0	150
738	0	1	0	1	1	0	0	0	1	1	0	1	0	0	0	0	0	0	0	0	0	0	0	0	1	150
739	0	1	0	0	0	0	0	0	0	0	0	0	0	0	0	0	0	0	0	1	0	1	1	0	1	150
740	0	0	0	1	0	0	0	0	0	0	0	1	0	0	0	0	0	0	0	1	0	1	0	0	0	150
741	0	0	0	0	0	0	1	0	1	0	0	0	1	0	0	1	0	0	0	1	0	1	0	0	0	150
742	0	0	0	0	1	0	0	0	0	0	0	1	0	0	0	0	0	0	0	1	0	1	0	0	1	149
743	0	1	0	1	1	0	0	0	0	0	0	1	1	0	0	0	0	0	0	0	0	0	1	0	0	149
744	0	0	0	1	1	0	0	0	1	1	0	1	0	0	0	0	0	0	0	1	0	0	0	0	0	148
745	0	0	0	0	0	0	1	0	0	1	0	0	0	1	0	0	0	0	0	1	0	0	1	0	0	148
746	0	0	1	0	0	0	0	0	0	1	0	1	0	0	0	0	0	0	0	0	0	0	0	1	0	147
747	0	0	0	0	0	0	1	0	0	1	0	0	0	0	0	0	0	0	0	0	0	0	0	0	1	147
748	0	1	1	0	0	0	0	0	0	0	0	0	0	0	0	0	0	0	0	0	0	0	0	0	0	147
749	0	1	0	0	0	0	0	0	0	0	0	0	0	0	0	0	0	0	0	0	0	0	1	1	0	147
750	0	1	0	0	0	0	0	0	1	1	0	1	0	0	0	0	0	0	0	0	0	0	1	0	0	146
751	0	0	0	0	0	0	0	0	0	1	0	1	0	1	0	0	0	0	0	0	0	1	1	0	1	145
752	0	0	0	1	1	0	0	0	0	0	0	1	0	0	0	0	0	0	0	0	0	0	0	1	1	145
753	0	0	1	1	0	0	1	0	1	1	0	1	1	1	0	0	0	0	0	1	0	0	0	1	1	144
754	0	1	0	0	0	0	0	0	0	0	0	0	1	0	0	0	0	0	0	0	0	0	1	0	0	144
755	0	1	0	0	0	0	0	0	0	0	0	0	0	0	0	0	0	0	0	1	0	1	1	0	0	144
756	0	0	0	0	0	0	0	0	0	1	1	0	0	0	0	0	0	0	0	0	0	1	0	0	0	144
757	0	0	0	0	0	0	0	0	1	1	0	1	0	0	0	0	0	0	0	0	0	0	1	0	0	143
758	0	0	0	0	0	0	0	0	0	1	0	0	0	1	0	0	0	0	0	1	0	0	1	0	0	143
759	0	0	1	0	0	0	0	0	1	0	0	0	0	0	0	0	0	0	0	0	0	0	0	1	1	143
760	0	0	1	0	0	0	0	0	0	0	0	0	1	0	0	0	0	0	0	0	0	0	0	0	0	143
761	0	0	0	0	0	0	1	0	0	0	0	0	0	0	0	0	0	0	0	1	0	0	0	0	1	143
762	0	0	0	0	0	0	1	0	0	0	0	0	1	0	0	0	0	0	0	0	0	1	0	0	0	142
763	0	1	0	0	0	0	0	0	0	0	0	0	0	0	0	0	0	0	0	0	0	0	0	1	0	142
764	0	1	0	1	1	0	0	0	0	0	0	1	1	0	0	0	0	0	0	1	0	1	0	0	0	141
765	0	1	0	0	1	0	0	0	1	0	0	1	0	0	0	0	0	0	0	1	0	1	0	0	1	140
766	0	1	0	0	1	0	0	0	1	0	0	1	0	0	0	0	0	0	0	1	0	0	1	0	0	140
767	0	1	1	0	1	0	0	0	0	0	0	1	0	0	0	0	0	0	0	0	0	0	0	0	0	139
768	0	0	0	0	0	0	0	0	0	0	0	0	0	0	0	1	0	0	0	0	0	0	1	0	0	139
769	0	0	0	0	0	0	0	0	0	1	0	1	0	0	0	0	0	0	0	0	0	0	1	0	1	139
770	0	0	0	0	1	0	1	0	0	0	0	1	0	0	0	0	0	0	0	0	0	0	0	0	0	139
771	0	0	0	0	0	0	0	0	1	0	0	0	1	0	0	1	0	0	0	1	0	1	0	0	0	139
772	0	0	0	0	1	0	0	0	0	0	0	0	0	0	0	0	0	0	0	1	0	0	0	0	1	138
773	0	0	0	0	0	0	1	0	0	0	0	0	0	1	0	0	0	0	0	1	0	0	0	0	0	138
774	0	0	0	0	0	0	0	0	0	0	0	0	0	0	0	0	0	0	0	1	0	1	1	1	0	138
775	0	0	0	0	0	0	0	0	0	0	1	0	0	0	0	0	0	0	0	0	0	1	1	1	0	138
776	0	0	0	0	1	0	0	0	0	1	0	1	0	0	0	0	0	0	0	0	0	1	0	1	1	137
777	0	1	0	1	1	0	0	0	1	0	0	1	1	0	0	0	0	0	0	1	0	0	0	1	1	137
778	0	0	0	0	1	0	0	0	1	0	0	1	0	0	0	0	0	0	0	1	0	1	0	0	1	137
779	0	0	0	0	0	0	1	0	0	0	0	0	1	0	0	0	0	0	0	0	0	0	0	0	0	137
780	0	0	0	0	0	0	1	0	0	1	0	0	0	1	0	0	0	0	0	0	0	0	0	1	0	136
781	0	1	0	0	0	0	0	0	0	0	0	1	0	0	0	0	0	0	0	1	0	0	0	0	1	136
782	0	0	0	0	0	0	1	0	0	0	0	0	0	0	0	1	0	0	0	0	0	0	0	1	1	135
783	0	0	0	0	1	0	0	0	0	0	0	1	1	0	0	0	0	0	0	0	0	0	0	0	1	135
784	0	0	0	0	0	0	1	0	1	0	0	1	0	1	0	0	0	0	0	0	0	0	0	1	1	135
785	0	1	0	1	1	0	0	0	0	0	0	1	1	0	0	0	0	0	0	0	0	0	0	0	1	135
786	0	1	0	0	1	0	0	0	0	0	0	1	1	0	0	0	0	0	0	0	0	1	0	0	1	135
787	0	1	0	0	0	0	0	0	1	0	0	1	0	0	0	0	0	0	0	1	0	0	1	0	0	135
788	0	1	0	1	1	0	0	0	1	1	0	1	1	1	0	0	0	0	0	1	0	0	0	0	0	134
789	0	1	0	1	1	0	0	0	1	0	0	1	0	0	0	0	0	0	0	0	0	1	0	0	0	134
790	0	0	0	0	0	0	0	0	0	1	0	0	0	0	0	0	0	0	0	1	0	0	0	0	1	134
791	0	1	0	1	1	0	0	0	0	0	0	1	0	1	0	0	0	0	0	1	0	0	0	0	0	132
792	0	0	1	0	0	0	0	0	0	0	0	1	0	1	0	0	0	0	0	0	0	0	0	1	1	132
793	0	1	0	0	1	0	0	0	0	0	0	1	1	0	0	0	0	0	0	1	0	0	1	0	0	132
794	0	0	0	0	0	0	0	0	0	0	0	1	0	0	0	1	0	0	0	0	0	0	0	0	1	132
795	0	1	0	1	1	0	0	0	0	1	0	1	0	0	0	0	0	0	0	1	0	1	0	0	0	131
796	0	0	0	1	0	0	0	0	0	1	0	1	0	0	0	0	0	0	0	0	0	0	0	1	1	131
797	0	0	0	0	0	0	0	0	0	0	0	0	1	0	0	0	0	0	0	0	0	0	0	1	1	131
798	0	0	0	1	1	0	0	0	0	0	0	1	0	0	0	0	0	0	0	1	0	0	1	0	0	131
799	0	1	0	1	1	0	0	0	0	1	0	1	0	0	0	0	0	0	0	0	0	1	0	0	0	131
800	0	0	0	0	0	0	1	0	0	0	0	1	0	0	0	0	0	0	0	1	0	1	0	0	0	131
801	0	1	0	0	1	0	0	0	0	0	0	1	0	0	0	0	0	0	0	1	0	1	1	0	0	130
802	0	1	0	1	1	0	0	0	1	0	0	1	0	0	0	0	0	0	0	1	0	0	0	1	1	129
803	0	0	1	0	0	0	1	0	0	1	0	1	0	1	0	0	0	0	0	0	0	0	0	1	0	129
804	0	0	0	1	1	0	0	0	0	0	0	1	0	0	0	0	0	0	0	1	0	1	0	0	0	129
805	0	0	0	0	0	0	0	0	0	0	0	1	0	0	0	0	0	0	0	1	0	1	1	0	1	129
806	0	1	0	0	0	0	0	0	1	0	0	1	0	0	0	0	0	0	0	0	0	1	1	0	0	129
807	0	0	0	0	0	0	0	0	0	0	1	1	0	1	0	0	0	0	0	0	0	0	0	1	1	129
808	0	0	0	0	0	0	0	0	1	0	0	0	0	0	0	0	0	0	0	1	0	0	1	0	0	129
809	0	1	0	0	1	0	0	0	0	1	0	1	1	0	0	0	0	0	0	1	0	0	0	1	1	128
810	0	1	0	0	1	0	0	0	0	0	0	1	1	0	0	0	0	0	0	0	0	0	1	1	1	128
811	0	0	0	0	0	0	0	0	1	1	0	1	0	0	0	0	0	0	0	1	0	0	0	0	1	128
812	0	0	0	0	0	0	0	0	1	0	0	0	1	0	0	0	0	0	0	0	0	1	0	0	0	128
813	0	1	0	0	1	0	0	0	0	0	0	1	0	0	0	0	0	0	0	1	0	0	1	0	1	127
814	0	0	0	0	1	0	0	0	0	0	0	1	1	0	0	0	0	0	0	0	0	0	0	1	1	127
815	0	0	0	0	0	0	1	0	0	1	0	0	0	1	0	0	0	0	0	0	0	0	0	1	1	127
816	0	1	0	0	1	0	1	0	0	0	0	1	0	0	0	0	0	0	0	0	0	0	0	0	1	127
817	0	1	0	1	0	0	0	0	0	0	0	1	0	0	0	0	0	0	0	0	0	0	0	0	1	127
818	0	1	0	0	0	0	0	0	0	0	0	0	0	0	0	0	0	0	0	1	0	1	0	0	0	127
819	0	0	1	0	0	0	1	0	1	1	0	1	0	1	0	0	0	0	0	1	0	1	0	1	1	126
820	0	0	1	0	0	0	1	0	0	0	0	0	0	0	0	0	0	0	0	0	0	1	0	0	0	126
821	0	0	1	0	0	0	0	0	1	1	0	1	0	1	0	0	0	0	0	1	0	1	0	0	1	126
822	0	0	0	0	0	0	0	0	1	0	0	0	0	0	0	0	0	0	0	0	0	0	0	1	0	126
823	0	0	0	0	0	0	1	0	0	1	0	1	0	1	0	0	0	0	0	1	0	1	0	1	1	125
824	0	0	0	1	1	0	0	0	0	0	0	1	0	0	0	0	0	0	0	0	0	1	0	0	0	125
825	0	0	0	0	0	0	0	0	1	0	0	0	1	0	0	0	0	0	0	1	0	0	0	0	0	125
826	0	1	0	0	0	0	0	0	1	1	0	1	0	0	0	0	0	0	0	0	0	0	0	1	1	124
827	0	1	0	0	1	0	0	0	1	1	0	1	0	0	0	0	0	0	0	1	0	0	1	0	0	124
828	0	0	0	0	0	0	0	0	1	1	0	1	0	1	0	0	0	0	0	0	0	1	0	0	1	123
829	0	1	0	0	1	0	0	0	0	0	0	1	0	0	0	0	0	0	0	0	0	1	1	0	1	123
830	0	0	1	0	0	0	0	0	0	1	0	1	0	1	0	0	0	0	0	1	0	0	0	1	1	122
831	0	1	0	0	1	0	0	0	0	1	0	1	1	0	0	0	0	0	0	0	0	0	0	0	1	122
832	0	0	0	0	0	0	1	0	1	1	0	1	0	1	0	0	0	0	0	0	0	1	0	0	0	122
833	0	1	0	1	0	0	0	0	0	0	0	1	0	0	0	0	0	0	0	0	0	1	1	0	0	122
834	0	1	0	1	0	0	0	0	0	0	0	1	0	0	0	0	0	0	0	1	0	1	0	0	0	122
835	0	1	0	0	0	0	1	0	0	0	0	1	0	0	0	0	0	0	0	0	0	0	1	0	0	122
836	0	0	0	0	0	0	1	0	0	1	1	1	0	1	0	0	0	0	0	0	0	0	0	0	0	121
837	0	0	0	0	0	0	0	0	0	0	0	1	0	0	0	0	0	0	0	1	0	0	1	1	1	121
838	0	0	0	0	0	0	1	0	0	0	0	0	0	0	0	0	0	0	0	1	0	1	1	0	0	121
839	0	0	0	0	0	0	0	0	1	1	0	1	0	1	0	0	0	0	0	0	0	0	0	0	0	119
840	0	0	0	0	0	0	0	0	0	0	0	0	0	1	0	0	0	0	0	1	0	1	0	0	1	119
841	0	0	0	1	0	0	0	0	0	0	0	1	0	0	0	0	0	0	0	0	0	0	0	1	1	119
842	0	0	0	0	1	0	0	0	1	1	0	1	0	0	0	0	0	0	0	0	0	1	0	0	0	119
843	0	0	0	0	0	0	0	0	0	1	0	0	0	0	0	0	0	0	0	0	0	1	0	1	1	119
844	0	0	0	0	0	0	0	0	0	0	0	0	1	0	0	1	0	0	0	1	0	1	0	0	0	119
845	0	1	0	1	1	0	1	0	0	0	0	1	0	0	0	0	0	0	0	1	0	0	0	1	1	118
846	0	0	1	0	0	0	0	0	0	1	0	0	0	1	0	0	0	0	0	0	0	0	0	1	0	118
847	0	0	0	0	1	0	0	0	1	0	0	1	0	0	0	0	0	0	0	0	0	1	0	1	1	118
848	0	0	0	0	0	0	1	0	0	1	0	1	0	0	0	0	0	0	0	0	0	0	1	0	0	118
849	0	0	1	0	0	0	1	0	0	1	0	1	0	1	0	0	0	0	0	1	0	1	0	1	1	117
850	0	0	1	0	0	0	0	0	1	0	0	1	0	0	0	0	0	0	0	0	0	0	0	1	1	117
851	0	0	0	0	1	0	0	0	0	0	0	1	0	0	0	0	0	0	0	1	0	0	0	0	1	117
852	0	0	0	0	1	0	0	0	0	1	0	1	0	0	0	0	0	0	0	1	0	0	1	0	0	116
853	0	1	0	1	1	0	0	0	0	1	0	1	0	0	0	0	0	0	0	0	0	0	0	1	0	116

CMP	CD2	CD3	CD4	CD7	CD8	CD10	CD13	CD18	CD26	CD29	CD36	CD44	CD45RA	CD49f	CD54	CD56	CD57	CD58	CD62L	CD71	CD80	Cytokeratin	Histone_H1	HLA-DQ	HLA-DR	Freq. (voxel)
**854**	0	0	0	1	0	0	0	0	0	1	0	1	0	0	0	0	0	0	0	0	0	0	1	0	0	116
**855**	0	1	0	0	1	0	0	0	0	1	0	1	0	0	0	0	0	0	0	0	0	0	1	0	1	116
**856**	0	1	0	0	0	0	0	0	0	0	0	0	0	1	0	0	0	0	0	0	0	0	1	0	0	116
**857**	0	0	0	0	0	0	0	0	1	0	0	0	0	1	0	0	0	0	0	0	0	1	0	0	1	115
**858**	0	1	0	1	0	0	0	0	0	0	0	1	1	0	0	0	0	0	0	1	0	0	0	0	0	115
**859**	0	0	0	0	0	0	1	0	0	0	0	1	0	1	0	0	0	0	0	1	0	0	0	1	1	115
**860**	0	0	0	0	0	0	1	0	0	0	0	1	0	1	0	0	0	0	0	0	0	1	1	0	0	115
**861**	0	0	0	0	0	0	1	0	0	0	0	0	1	0	0	0	0	0	0	0	0	0	0	1	0	115
**862**	0	1	0	0	1	0	0	0	1	0	0	1	1	0	0	0	0	0	0	0	0	1	0	1	1	114
**863**	0	1	0	1	1	0	0	0	0	0	0	1	1	0	0	0	0	0	0	1	0	0	1	0	0	114
**864**	0	1	0	1	0	0	0	0	1	0	0	1	0	0	0	0	0	0	0	0	0	0	0	0	0	114
**865**	0	0	1	0	0	0	0	0	0	0	0	1	0	0	0	0	0	0	0	0	0	0	0	0	1	114
**866**	0	1	0	1	0	0	0	0	1	1	0	1	0	0	0	0	0	0	0	0	0	0	1	0	0	113
**867**	0	0	0	0	1	0	0	0	0	1	0	1	0	1	0	0	0	0	0	0	0	1	1	0	1	113
**868**	0	0	0	1	0	0	0	0	0	0	0	0	0	0	0	0	0	0	0	1	0	1	0	0	0	113
**869**	0	1	0	0	1	0	0	0	0	1	0	1	1	0	0	0	0	0	0	1	0	0	1	0	0	113
**870**	0	0	0	1	0	0	1	0	0	1	0	1	0	1	0	0	0	0	0	1	0	0	0	1	1	112
**871**	0	0	0	0	0	0	0	0	0	0	0	1	0	1	0	0	0	0	0	1	0	0	0	1	1	112
**872**	0	1	0	0	1	0	0	0	0	0	0	0	0	0	0	0	0	0	0	0	0	1	1	0	0	112
**873**	0	0	1	0	0	0	0	0	0	0	0	0	0	0	0	0	0	0	0	0	0	0	0	0	1	111
**874**	0	0	0	0	0	0	0	0	0	1	0	1	0	1	0	0	0	0	0	0	0	1	1	1	1	110
**875**	0	0	1	0	0	0	0	0	0	1	0	1	0	0	0	0	0	0	0	0	0	0	0	0	1	110
**876**	0	0	0	0	0	0	0	0	0	0	0	0	0	0	0	1	0	0	0	0	0	1	0	1	0	110
**877**	0	1	0	0	0	0	0	0	0	0	0	1	0	0	0	0	0	0	0	1	0	1	0	0	1	109
**878**	0	0	1	0	0	0	0	0	0	0	0	0	0	1	0	0	0	0	0	0	0	0	0	1	0	109
**879**	0	0	0	0	0	0	0	0	0	0	0	0	1	0	0	0	0	0	0	1	0	1	0	1	0	109
**880**	0	0	1	0	0	0	0	0	1	0	0	0	0	0	0	0	0	0	0	1	0	1	0	0	0	109
**881**	0	0	1	0	0	0	0	0	1	1	0	1	0	1	0	0	0	0	0	1	0	1	0	1	1	108
**882**	0	1	0	1	0	0	0	0	0	1	0	1	0	0	0	0	0	0	0	1	0	0	1	0	0	108
**883**	0	0	0	0	0	0	0	0	1	1	0	1	0	0	0	0	0	0	0	1	0	0	0	1	0	108
**884**	0	1	0	0	1	0	0	0	1	1	0	1	1	0	0	0	0	0	0	0	0	0	0	1	1	107
**885**	0	0	0	1	1	0	0	0	0	1	0	1	1	0	0	0	0	0	0	0	0	0	0	0	0	107
**886**	0	0	0	0	0	0	1	0	0	0	0	1	0	0	0	0	0	0	0	1	0	0	0	1	0	107
**887**	0	1	0	0	1	0	0	0	1	0	0	1	0	0	0	0	0	0	0	0	0	1	0	0	1	107
**888**	0	1	0	1	1	0	0	0	0	0	0	1	0	0	0	0	0	0	0	0	0	0	1	0	1	107
**889**	0	1	0	1	1	0	0	0	1	1	0	1	1	0	0	0	0	0	0	1	0	0	0	1	1	106
**890**	0	1	0	0	1	0	0	0	0	0	0	1	1	0	0	0	0	0	0	1	0	0	0	1	1	106
**891**	0	0	0	0	1	0	0	0	0	1	0	1	1	0	0	0	0	0	0	1	0	0	0	0	0	106
**892**	0	0	0	1	0	0	0	0	0	0	0	1	0	0	0	0	0	0	0	1	0	0	1	0	0	106
**893**	0	1	0	0	1	0	0	0	0	1	0	1	0	1	0	0	0	0	0	0	0	1	0	0	0	106
**894**	0	0	0	0	0	0	1	0	0	1	0	0	0	1	0	0	0	0	0	0	0	0	0	0	1	106
**895**	0	0	0	0	0	0	1	0	0	1	0	0	0	1	0	0	0	0	0	0	0	1	0	0	1	106
**896**	0	0	0	0	0	0	0	0	0	1	0	0	0	1	0	0	0	0	0	0	0	0	0	0	1	106
**897**	0	0	0	0	0	0	1	0	1	0	0	0	1	0	0	1	0	0	0	1	0	0	0	0	0	106
**898**	0	1	0	0	1	0	1	0	1	0	0	1	0	0	0	0	0	0	0	1	0	0	0	1	1	105
**899**	0	1	0	0	0	0	0	0	0	0	0	1	0	0	0	0	0	0	0	1	0	0	1	0	1	105
**900**	0	1	0	0	1	0	0	0	0	0	0	1	0	0	0	0	0	0	0	0	0	1	0	1	0	105
**901**	0	0	1	0	0	0	1	0	1	0	0	1	0	0	0	0	0	0	0	0	0	0	0	1	1	104
**902**	0	1	0	0	1	0	0	0	1	1	0	1	1	0	0	0	0	0	0	0	0	0	1	0	0	104
**903**	0	0	0	0	1	0	0	0	1	0	0	0	0	0	0	0	0	0	0	0	0	1	0	0	0	104
**904**	0	0	0	0	0	0	0	0	0	1	0	1	0	0	0	0	0	0	0	1	0	0	1	1	1	104
**905**	0	0	1	0	0	0	1	0	1	0	0	1	0	0	0	0	0	0	0	1	0	0	0	1	1	103
**906**	0	1	0	0	1	0	1	0	0	0	0	1	0	0	0	0	0	0	0	0	0	0	0	1	1	103
**907**	0	0	1	0	0	0	1	0	1	1	0	1	0	1	0	0	0	0	0	0	0	0	0	1	1	103
**908**	0	1	0	0	1	0	0	0	0	1	0	1	0	0	0	0	0	0	0	0	0	1	0	0	1	103
**909**	0	1	0	0	0	0	0	0	0	0	0	1	1	0	0	0	0	0	0	0	0	1	1	0	0	103
**910**	0	1	0	0	0	0	0	0	0	0	0	1	0	0	0	0	0	0	0	1	0	0	0	1	0	103
**911**	0	0	1	0	0	0	0	0	0	0	0	1	0	0	0	0	0	0	0	1	0	1	0	1	1	102
**912**	0	1	0	0	1	0	0	0	1	1	0	1	0	0	0	0	0	0	0	1	0	0	0	0	1	102
**913**	0	0	0	0	0	0	0	0	0	1	0	1	0	1	0	0	0	0	0	1	0	1	1	1	0	102
**914**	0	0	0	0	0	0	1	0	0	1	0	0	0	1	0	0	0	0	0	0	0	0	1	0	0	102
**915**	0	0	0	1	0	0	0	0	0	0	0	0	0	0	0	0	0	0	0	0	0	0	0	1	1	102
**916**	0	0	0	0	0	0	1	0	0	0	0	0	1	0	0	1	0	0	0	0	0	0	0	0	0	102
**917**	0	0	0	0	0	0	0	0	1	0	0	1	0	0	0	1	0	0	0	0	0	0	0	1	1	101
**918**	0	0	0	0	0	0	1	0	1	1	0	1	0	1	0	0	0	0	0	1	0	0	0	1	1	100
**919**	0	0	0	0	0	0	1	0	0	0	0	0	0	1	0	0	0	0	0	1	0	0	0	1	1	100
**920**	0	0	0	0	0	0	0	0	0	1	1	1	0	0	0	0	0	0	0	1	0	0	0	1	1	100
**921**	0	0	0	0	0	0	0	0	0	0	1	0	0	0	0	0	0	0	0	0	0	1	1	0	1	100
**922**	0	1	0	0	1	0	0	0	0	0	0	1	1	0	0	0	0	0	0	1	0	1	0	0	0	99
**923**	0	0	0	0	0	0	1	0	1	1	0	1	0	1	0	0	0	0	0	0	0	0	0	1	1	99
**924**	0	0	0	1	1	0	0	0	0	0	0	1	1	0	0	0	0	0	0	0	0	0	0	0	0	99
**925**	0	0	0	0	1	0	0	0	0	1	0	1	1	0	0	0	0	0	0	0	0	1	0	0	0	99
**926**	0	1	1	0	0	0	0	0	0	0	0	1	0	0	0	0	0	0	0	0	0	0	0	0	0	99
**927**	0	1	1	1	1	0	0	0	1	0	0	1	1	0	0	0	0	0	0	1	0	0	0	1	1	98
**928**	0	0	0	0	0	0	1	0	1	0	0	1	0	0	0	0	0	0	0	0	0	0	0	1	0	98
**929**	0	0	1	0	0	0	0	0	0	0	0	1	0	1	0	0	0	0	0	1	0	1	0	0	0	98
**930**	0	1	0	0	0	0	0	0	1	1	0	1	0	0	0	0	0	0	0	0	0	0	0	1	0	98
**931**	0	0	0	0	0	0	0	0	0	1	0	1	0	1	0	0	0	0	0	0	0	0	1	1	1	98
**932**	0	0	1	0	0	0	1	0	0	1	0	1	0	1	0	0	0	0	0	1	0	0	0	1	0	97
**933**	0	1	0	0	0	0	0	0	0	1	0	1	0	0	0	0	0	0	0	1	0	0	0	1	1	97
**934**	0	0	1	0	0	0	0	0	0	1	0	0	0	0	0	0	0	0	0	0	0	1	0	0	0	97
**935**	0	0	0	0	0	0	1	0	1	1	0	1	0	0	0	0	0	0	0	0	0	0	0	0	0	96
**936**	0	1	0	0	1	0	1	0	0	0	0	1	0	0	0	0	0	0	0	0	0	0	0	0	0	96
**937**	0	0	0	0	0	0	0	0	0	1	0	1	0	1	0	0	0	0	0	1	0	0	1	0	1	95
**938**	0	0	1	0	0	0	0	0	0	1	0	1	0	0	0	0	0	0	0	1	0	0	0	1	0	95
**939**	0	0	0	1	0	0	0	0	0	0	0	1	0	0	0	0	0	0	0	0	0	0	0	0	1	95
**940**	0	0	0	0	0	0	0	0	0	0	0	0	1	0	0	1	0	0	0	1	0	1	0	1	0	95
**941**	0	0	1	1	0	0	1	0	0	1	0	1	0	1	0	0	0	0	0	1	0	0	0	1	1	94
**942**	0	0	1	0	0	0	1	0	0	0	0	0	0	0	0	0	0	0	0	1	0	0	0	1	1	94
**943**	0	0	0	0	1	0	0	0	0	0	0	0	1	0	0	0	0	0	0	0	0	1	0	0	0	94
**944**	0	0	0	0	0	0	0	0	0	1	0	0	0	1	0	0	0	0	0	0	0	1	0	1	0	94
**945**	0	0	0	0	1	0	0	0	0	0	0	1	1	0	0	0	0	0	0	0	0	0	1	0	0	94
**946**	0	1	0	0	1	0	0	0	0	0	0	0	1	0	0	0	0	0	0	0	0	0	0	0	0	94
**947**	0	1	0	1	1	0	0	0	1	0	0	1	0	0	0	0	0	0	0	0	0	0	0	1	1	93
**948**	0	0	1	0	0	0	0	0	1	1	0	1	0	0	0	0	0	0	0	1	0	0	0	1	1	93
**949**	0	1	0	1	1	0	0	0	1	1	0	1	1	0	0	0	0	0	0	0	0	0	0	0	1	93
**950**	0	1	0	0	0	0	0	0	0	0	0	0	1	0	0	0	0	0	0	0	0	0	0	0	0	93
**951**	0	0	0	0	0	0	0	0	1	1	0	1	0	0	0	0	0	0	0	1	0	1	0	1	0	92
**952**	0	1	0	1	1	0	0	0	0	1	0	1	1	0	0	0	0	0	0	1	0	0	0	0	1	92
**953**	0	0	1	0	0	0	1	0	0	0	0	1	0	1	0	0	0	0	0	1	0	0	0	1	1	92
**954**	0	0	1	0	0	0	0	0	0	1	0	1	0	0	0	0	0	0	0	1	0	0	0	0	1	92
**955**	0	0	0	0	1	0	1	0	0	1	0	1	0	0	0	0	0	0	0	0	0	0	0	0	0	92
**956**	0	1	0	1	1	0	1	0	0	0	0	1	0	0	0	0	0	0	0	0	0	0	0	1	1	91
**957**	0	0	0	0	1	0	0	0	1	0	0	1	0	0	0	0	0	0	0	0	0	0	0	1	0	91
**958**	0	0	0	1	0	0	0	0	0	0	0	0	0	1	0	0	0	0	0	0	0	0	0	0	0	91
**959**	0	0	0	0	0	0	1	0	1	0	1	0	0	1	0	0	0	0	0	0	0	1	0	0	0	91
**960**	0	0	0	0	1	0	0	0	0	1	0	1	0	0	0	0	0	0	0	0	0	1	0	0	1	91
**961**	0	1	0	1	1	0	0	0	1	0	0	1	0	0	0	0	0	0	0	1	0	0	0	0	1	91
**962**	0	0	0	0	0	0	1	0	0	1	1	1	0	1	0	0	0	0	0	1	0	0	0	1	1	91
**963**	0	1	0	0	1	0	0	0	1	1	0	1	1	0	0	0	0	0	0	1	0	0	0	1	1	90
**964**	0	1	0	0	1	0	0	0	0	0	0	1	0	0	0	0	0	0	0	1	0	1	0	1	1	90
**965**	0	0	0	0	1	0	0	0	0	1	0	0	0	0	0	0	0	0	0	0	0	0	1	0	0	90
**966**	0	0	1	1	0	0	1	0	0	1	0	1	1	1	0	1	0	0	0	1	0	0	0	1	1	90
**967**	0	0	0	1	0	0	1	0	0	0	0	1	0	0	0	0	0	0	0	0	0	0	0	0	0	90
**968**	0	1	0	0	0	0	1	0	0	1	0	1	0	0	0	0	0	0	0	0	0	0	1	0	0	90
**969**	0	0	0	0	0	0	0	0	1	0	0	0	0	0	0	0	0	0	0	0	0	0	1	1	1	90
**970**	0	1	0	0	1	0	0	0	1	0	0	1	0	0	0	0	0	0	0	1	0	1	0	1	1	89
**971**	0	0	0	0	1	0	1	0	1	0	0	1	0	0	0	0	0	0	0	0	0	0	0	1	1	89
**972**	0	0	0	0	0	0	0	0	1	0	0	1	0	1	0	0	0	0	0	1	0	1	0	1	0	89
**973**	0	1	0	0	0	0	0	0	0	0	0	0	0	0	0	0	0	0	0	0	0	1	1	1	1	89
**974**	0	0	0	0	0	0	1	0	0	1	0	0	0	0	0	0	0	0	0	0	0	0	0	1	1	89
**975**	0	1	0	0	0	0	0	0	1	0	0	1	0	0	0	0	0	0	0	1	0	1	0	1	1	88

CMP	CD2	CD3	CD4	CD7	CD8	CD10	CD13	CD18	CD26	CD29	CD36	CD44	CD45RA	CD49f	CD54	CD56	CD57	CD58	CD62L	CD71	CD80	Cytokeratin	Histone_H1	HLA-DQ	HLA-DR	Freq. (voxel)
**976**	0	0	1	0	0	0	0	0	1	1	0	1	0	1	0	0	0	0	0	1	0	1	0	0	0	88
**977**	0	1	0	1	1	0	0	0	0	1	0	1	1	0	0	0	0	0	0	0	0	0	1	0	0	88
**978**	0	0	1	0	0	0	0	0	0	1	0	0	0	0	0	0	0	0	0	1	0	0	0	1	0	88
**979**	0	1	0	0	0	0	0	0	0	0	0	1	0	0	0	0	0	0	0	1	0	1	1	1	1	87
**980**	0	0	1	0	0	0	0	0	0	1	0	1	0	1	0	0	0	0	0	1	0	1	0	1	1	87
**981**	0	1	0	1	1	0	0	0	1	1	0	1	1	0	0	0	0	0	0	1	0	0	1	0	0	87
**982**	0	0	0	0	0	0	0	0	0	1	1	1	0	1	0	0	0	0	0	0	0	0	0	0	0	87
**983**	0	1	0	0	1	0	0	0	1	0	0	1	0	0	0	0	0	0	0	0	0	0	0	0	1	87
**984**	0	0	0	0	0	0	1	0	0	0	0	1	0	0	0	0	0	0	0	0	0	1	0	0	1	87
**985**	0	0	0	0	0	0	1	0	0	0	0	0	0	0	0	0	0	0	0	0	0	0	1	1	0	87
**986**	0	0	0	1	0	0	0	0	0	0	0	1	0	0	0	0	0	0	0	0	0	1	1	0	0	86
**987**	0	0	0	0	0	0	0	0	1	0	0	0	0	0	0	0	0	0	0	0	0	0	0	0	1	86
**988**	0	0	1	0	0	0	1	0	0	0	0	0	0	1	0	0	0	0	0	0	0	0	0	1	1	86
**989**	0	0	0	0	0	0	0	0	0	1	0	0	0	0	0	0	0	0	0	1	0	1	0	0	1	86
**990**	0	0	0	0	1	0	0	0	0	0	0	1	1	0	0	0	0	0	0	0	0	1	0	0	1	86
**991**	0	1	0	1	1	0	1	0	1	0	0	1	0	0	0	0	0	0	0	1	0	0	0	1	1	85
**992**	0	1	0	0	1	0	1	0	1	0	0	1	0	0	0	0	0	0	0	0	0	0	0	1	1	85
**993**	0	0	0	1	0	0	0	0	0	0	0	1	0	0	0	0	0	0	0	1	0	1	1	0	0	85
**994**	0	1	0	1	1	0	0	0	0	0	0	1	0	0	0	0	0	0	0	0	0	0	0	1	0	85
**995**	0	1	0	1	0	0	0	0	1	0	0	1	0	0	0	0	0	0	0	1	0	0	0	0	0	85
**996**	0	1	0	0	0	0	0	0	0	0	0	0	0	1	0	0	0	0	0	0	0	0	0	0	0	85
**997**	0	1	0	1	1	0	0	0	0	0	0	1	0	0	0	0	0	0	0	1	0	0	0	1	0	84
**998**	0	1	0	0	0	0	0	0	0	1	0	1	0	0	0	0	0	0	0	0	0	1	0	1	1	84
**999**	0	1	0	0	0	0	0	0	0	1	0	0	0	0	0	0	0	0	0	1	0	0	1	0	0	84
**1.000**	0	0	0	0	0	0	1	0	0	0	0	1	0	1	0	0	0	0	0	0	0	0	1	0	0	84
**1.001**	0	0	0	0	0	0	0	0	0	1	0	1	0	0	0	0	0	0	0	1	0	0	1	1	0	84
**1.002**	0	0	0	0	1	0	0	0	0	1	0	1	0	0	0	0	0	0	0	1	0	0	0	1	0	84
**1.003**	0	0	0	0	0	0	0	0	0	1	0	1	0	1	0	0	0	0	0	0	0	0	1	1	0	84
**1.004**	0	0	1	0	0	0	1	0	1	0	0	1	0	1	0	0	0	0	0	1	0	1	0	0	1	83
**1.005**	0	0	0	1	1	0	0	0	0	0	0	0	0	0	0	0	0	0	0	1	0	0	1	0	0	83
**1.006**	0	0	1	0	0	0	0	0	1	0	0	1	0	0	0	0	0	0	0	1	0	1	0	1	1	82
**1.007**	0	1	0	0	1	0	0	0	1	0	0	1	1	0	0	0	0	0	0	1	0	0	0	1	1	82
**1.008**	0	0	0	0	0	0	1	0	1	0	0	1	0	0	0	1	0	0	0	0	0	0	0	1	1	82
**1.009**	0	0	0	1	0	0	0	0	1	0	0	1	0	0	0	0	0	0	0	0	0	1	0	0	0	82
**1.010**	0	1	0	1	0	0	0	0	1	0	0	1	0	0	0	0	0	0	0	0	0	0	1	0	0	82
**1.011**	0	0	1	0	0	0	0	0	1	0	0	1	0	1	0	0	0	0	0	1	0	1	0	0	1	81
**1.012**	0	1	0	1	0	0	0	0	0	1	0	1	0	0	0	0	0	0	0	0	0	0	0	0	1	81
**1.013**	0	1	0	0	1	0	0	0	0	1	0	1	0	0	0	0	0	0	0	1	0	0	0	1	0	81
**1.014**	0	0	0	0	0	0	0	0	0	0	0	0	0	0	0	1	0	0	0	0	0	1	1	0	0	81
**1.015**	0	1	0	1	0	0	0	0	0	0	0	0	0	0	0	0	0	0	0	0	0	1	0	0	0	81
**1.016**	0	0	0	0	0	0	0	0	0	1	0	0	0	0	0	0	0	0	0	1	0	1	1	0	0	81
**1.017**	0	1	0	1	1	0	0	0	1	0	0	1	1	0	0	0	0	0	0	1	0	0	0	0	1	80
**1.018**	0	1	0	0	0	0	0	0	0	1	0	1	0	0	0	0	0	0	0	1	0	0	0	0	1	80
**1.019**	0	0	1	0	0	0	0	0	0	1	0	1	0	1	0	0	0	0	0	0	0	0	0	1	0	80
**1.020**	0	0	0	0	0	0	1	0	0	1	0	0	0	0	0	0	0	0	0	0	0	0	1	0	0	80
**1.021**	0	0	0	0	0	0	1	0	0	0	0	0	1	0	0	0	0	0	0	1	0	1	0	0	0	80
**1.022**	0	0	1	0	0	0	0	0	1	0	0	0	0	0	0	0	0	0	0	1	0	1	0	1	1	79
**1.023**	0	1	0	1	1	0	0	0	0	1	0	1	1	1	0	0	0	0	0	1	0	0	0	0	0	79
**1.024**	0	0	0	0	0	0	1	0	0	1	0	1	0	0	0	0	0	0	0	1	0	0	0	1	0	79
**1.025**	0	1	0	0	1	0	0	0	1	1	0	1	0	0	0	0	0	0	0	0	0	1	0	1	1	79
**1.026**	0	1	0	1	0	0	0	0	0	1	0	1	0	0	0	0	0	0	0	1	0	0	0	0	1	79
**1.027**	0	1	0	1	1	0	0	0	1	0	0	1	0	0	0	0	0	0	0	1	0	0	0	1	0	79
**1.028**	0	0	1	1	0	0	1	0	0	1	0	1	1	1	0	0	0	0	0	1	0	0	0	1	1	79
**1.029**	0	0	0	0	0	0	0	0	0	1	0	0	0	1	0	0	0	0	0	0	0	1	0	1	1	79
**1.030**	0	0	0	0	0	0	0	0	0	0	0	1	0	0	0	0	0	0	0	1	0	0	1	0	1	79
**1.031**	0	0	0	0	1	0	1	0	1	0	0	0	1	0	0	1	0	0	0	1	0	1	0	0	0	79
**1.032**	0	0	0	0	0	0	0	0	1	0	0	1	0	0	0	0	0	0	0	1	0	0	1	0	0	78
**1.033**	0	1	0	0	1	0	0	0	0	0	0	1	0	1	0	0	0	0	0	1	0	0	0	0	0	78
**1.034**	0	0	0	0	0	0	1	0	1	1	0	0	0	1	0	0	0	0	0	0	0	1	0	0	0	78
**1.035**	0	1	0	0	1	0	0	0	1	0	0	1	0	0	0	0	0	0	0	0	0	0	1	1	1	78
**1.036**	0	0	0	0	1	0	0	0	1	0	0	1	0	0	0	0	0	0	0	1	0	0	0	0	0	78
**1.037**	0	0	0	0	1	0	0	0	0	1	0	1	0	1	0	0	0	0	0	0	0	0	1	0	1	78
**1.038**	0	0	0	0	1	0	0	0	0	1	0	1	0	0	0	0	0	0	0	0	0	1	1	0	0	78
**1.039**	0	0	1	0	0	0	0	0	0	0	0	0	1	0	0	0	0	0	0	0	0	0	1	0	0	78
**1.040**	0	1	0	0	0	0	0	0	0	0	0	0	0	0	0	0	0	0	0	1	0	1	1	1	1	77
**1.041**	0	0	0	0	0	0	1	0	0	0	0	1	0	1	0	0	0	0	0	1	0	1	0	0	1	77
**1.042**	0	0	0	0	0	0	1	0	1	0	0	0	0	1	0	0	0	0	0	0	0	1	1	0	0	77
**1.043**	0	1	0	0	1	0	0	0	0	0	0	0	0	0	0	0	0	0	0	0	0	1	0	0	1	77
**1.044**	0	1	0	0	1	0	1	0	0	1	0	1	0	0	0	0	0	0	0	0	0	0	1	0	0	77
**1.045**	0	0	0	0	0	0	0	0	1	0	0	0	0	0	0	0	0	0	0	1	0	0	0	1	0	77
**1.046**	0	0	0	0	1	0	0	0	1	1	0	1	0	0	0	0	0	0	0	1	0	0	0	0	1	76
**1.047**	0	1	0	1	1	0	0	0	0	1	0	1	1	0	0	0	0	0	0	1	0	0	0	1	1	76
**1.048**	0	1	0	1	1	0	0	0	1	0	0	1	0	0	0	0	0	0	0	0	0	0	0	1	0	76
**1.049**	0	0	0	1	1	0	0	0	1	0	0	1	0	0	0	0	0	0	0	1	0	1	0	0	0	76
**1.050**	0	0	0	0	1	0	0	0	1	1	0	1	0	0	0	0	0	0	0	0	0	0	0	1	1	76
**1.051**	0	0	0	0	1	0	0	0	1	1	0	1	0	0	0	0	0	0	0	0	0	0	0	0	1	76
**1.052**	0	0	0	0	0	0	0	0	1	0	0	1	0	0	0	0	0	0	0	0	0	0	1	1	1	76
**1.053**	0	1	0	1	1	0	0	0	0	0	0	1	0	0	0	0	0	0	0	1	0	0	1	0	1	76
**1.054**	0	1	0	0	1	0	0	0	0	1	0	1	1	1	0	0	0	0	0	0	0	0	0	0	0	76
**1.055**	0	0	0	0	0	0	1	0	0	0	0	0	0	0	0	0	0	0	0	0	0	1	0	1	0	76
**1.056**	0	1	0	1	0	0	0	0	1	0	0	0	0	0	0	0	0	0	0	0	0	0	0	0	0	76
**1.057**	0	1	1	1	1	0	0	0	1	1	0	1	0	0	0	0	0	0	0	1	0	0	0	0	0	75
**1.058**	0	0	1	0	1	0	1	0	1	0	0	1	0	0	0	0	0	0	0	0	0	0	0	1	1	75
**1.059**	0	1	0	1	1	0	0	0	1	1	0	1	1	0	0	0	0	0	0	1	0	0	0	0	1	75
**1.060**	0	0	1	0	0	0	0	0	0	1	0	1	0	1	0	0	0	0	0	1	0	1	1	0	0	75
**1.061**	0	1	0	1	1	0	0	0	1	0	0	1	0	0	0	0	0	0	0	1	0	1	1	0	0	75
**1.062**	0	0	0	0	0	0	0	0	0	0	0	1	0	0	0	1	0	0	0	0	0	0	1	1	1	75
**1.063**	0	1	0	1	0	0	0	0	0	0	0	1	0	1	0	0	0	0	0	0	0	0	0	0	0	75
**1.064**	0	1	0	0	1	0	0	0	0	1	0	1	0	0	0	0	0	0	0	1	0	1	0	0	0	75
**1.065**	0	0	0	0	0	0	1	0	1	0	0	0	0	0	0	0	0	0	0	1	0	1	0	0	0	75
**1.066**	0	1	0	1	1	0	0	0	1	1	0	1	0	0	0	0	0	0	0	1	0	1	0	0	0	74
**1.067**	0	0	0	0	1	0	0	0	1	1	0	1	1	0	0	0	0	0	0	1	0	0	0	0	0	74
**1.068**	0	0	1	0	0	0	1	0	0	1	0	1	0	1	0	0	0	0	0	0	0	1	0	0	0	74
**1.069**	0	0	0	0	0	0	1	0	0	1	0	1	0	0	0	0	0	0	0	0	0	1	0	0	1	74
**1.070**	0	1	1	1	1	0	0	0	1	1	0	1	1	0	0	0	0	0	0	1	0	0	0	0	0	73
**1.071**	0	1	0	1	1	0	1	0	1	0	0	1	0	0	0	0	0	0	0	0	0	0	0	1	1	73
**1.072**	0	0	1	0	0	0	0	0	1	1	0	1	0	0	0	0	0	0	0	0	0	0	0	1	1	73
**1.073**	0	1	0	0	0	0	0	0	0	0	0	1	1	0	0	0	0	0	0	0	0	0	0	1	1	73
**1.074**	0	1	0	0	0	0	0	0	1	1	0	1	0	0	0	0	0	0	0	0	0	1	0	0	0	73
**1.075**	0	0	0	0	1	0	0	0	0	0	0	0	1	0	0	0	0	0	0	0	0	0	1	0	0	73
**1.076**	0	1	1	1	1	0	0	0	1	1	0	1	1	1	0	0	0	0	0	1	0	0	0	0	0	72
**1.077**	0	1	0	1	1	0	1	0	0	1	0	1	0	0	0	0	0	0	0	1	0	0	0	1	1	72
**1.078**	0	0	1	0	0	0	0	0	1	0	0	0	0	0	0	0	0	0	0	0	0	0	0	1	0	72
**1.079**	0	0	0	0	0	0	1	0	0	0	1	1	0	1	0	0	0	0	0	0	0	1	0	0	0	72
**1.080**	0	0	0	1	0	0	0	0	0	0	0	1	0	1	0	0	0	0	0	0	0	0	0	0	0	72
**1.081**	0	1	0	0	1	0	0	0	1	1	0	1	0	0	0	0	0	0	0	0	0	1	0	0	0	72
**1.082**	0	0	0	1	1	0	0	0	1	0	0	1	0	0	0	0	0	0	0	0	0	0	0	0	0	72
**1.083**	0	0	0	0	1	0	0	0	0	1	0	0	0	0	0	0	0	0	0	1	0	0	0	0	0	71
**1.084**	0	0	1	0	0	0	0	0	1	0	0	1	0	0	0	0	0	0	0	1	0	1	0	0	1	71
**1.085**	0	0	0	0	0	0	1	0	0	0	0	1	0	1	0	0	0	0	0	0	0	0	1	0	1	71
**1.086**	0	0	0	0	1	0	0	0	0	0	0	1	1	0	0	0	0	0	0	1	0	0	0	0	0	71
**1.087**	0	0	1	0	0	0	0	0	0	0	0	1	0	0	0	0	0	0	0	1	0	0	0	0	1	71
**1.088**	0	0	0	0	0	0	1	0	0	1	0	1	0	1	0	0	0	0	0	1	0	1	0	1	0	71
**1.089**	0	0	1	0	0	0	1	0	1	0	0	1	0	1	0	0	0	0	0	1	0	0	0	1	1	71
**1.090**	0	0	1	0	0	0	0	0	0	1	0	0	0	1	0	0	0	0	0	0	0	0	0	1	1	71
**1.091**	0	0	0	0	0	0	0	0	1	0	1	0	0	0	0	0	0	0	0	0	0	1	0	0	1	71
**1.092**	0	0	1	0	0	0	0	0	0	0	0	0	1	0	0	1	0	0	0	1	0	0	1	0	0	71
**1.093**	0	1	0	0	1	0	0	0	0	0	0	0	0	0	0	0	0	0	0	1	0	0	0	0	1	70
**1.094**	0	1	0	1	1	0	0	0	0	0	0	1	0	0	0	0	0	0	0	1	0	1	1	0	0	70
**1.095**	0	0	0	0	1	0	0	0	0	1	0	1	0	1	0	0	0	0	0	1	0	1	0	0	0	70
**1.096**	0	1	0	1	0	0	0	0	0	0	0	0	0	0	0	0	0	0	0	0	0	0	1	0	1	70
**1.097**	0	0	0	0	0	0	0	0	1	1	0	0	0	0	0	0	0	0	0	0	0	0	0	0	0	70

CMP	CD2	CD3	CD4	CD7	CD8	CD10	CD13	CD18	CD26	CD29	CD36	CD44	CD45RA	CD49f	CD54	CD56	CD57	CD58	CD62L	CD71	CD80	Cytokeratin	Histone_H1	HLA-DQ	HLA-DR	Freq. (voxel)
**1.098**	0	0	0	0	0	0	1	0	0	0	0	0	1	0	0	0	0	0	0	1	0	0	0	0	0	70
**1.099**	0	0	0	0	0	0	0	0	0	1	1	0	0	0	0	0	0	0	0	0	0	0	0	0	0	70
**1.100**	0	0	0	0	0	0	0	0	1	0	0	0	1	0	0	0	0	0	0	0	0	0	0	0	0	70
**1.101**	0	0	0	1	0	0	1	0	0	0	0	1	0	0	0	0	0	0	0	0	0	0	0	1	1	69
**1.102**	0	0	0	0	1	0	0	0	1	1	0	1	0	0	0	0	0	0	0	1	0	1	0	0	0	69
**1.103**	0	0	0	0	0	0	0	0	0	0	0	0	0	0	0	0	0	0	0	1	0	1	1	1	1	69
**1.104**	0	0	0	1	0	0	0	0	1	1	0	1	0	0	0	0	0	0	0	1	0	0	0	0	0	69
**1.105**	0	0	0	0	0	0	1	0	0	0	0	0	0	0	0	1	0	0	0	1	0	0	0	1	1	69
**1.106**	0	1	0	1	1	0	0	0	0	1	0	1	0	0	0	0	0	0	0	1	0	0	0	1	0	69
**1.107**	0	0	0	1	0	0	1	0	0	1	0	1	0	1	0	0	0	0	0	0	0	0	0	1	1	69
**1.108**	0	0	1	0	0	0	0	0	0	0	0	0	0	1	0	0	0	0	0	1	0	1	0	0	0	69
**1.109**	0	1	0	0	0	0	0	0	0	0	0	0	0	0	0	0	0	0	0	1	0	0	0	0	1	69
**1.110**	0	0	0	0	0	0	1	0	0	0	0	0	1	0	0	1	0	0	0	0	0	0	0	1	0	69
**1.111**	0	0	1	0	0	0	0	0	0	0	0	0	0	1	0	0	0	0	0	0	0	1	1	0	0	69
**1.112**	0	0	0	0	0	0	1	0	1	0	0	0	1	0	0	0	0	0	0	1	0	0	0	0	0	69
**1.113**	0	0	1	0	0	0	0	0	0	0	0	0	1	0	0	1	0	0	0	0	0	1	0	1	0	69
**1.114**	0	1	1	1	1	0	0	0	1	1	0	1	1	1	0	0	0	0	0	1	0	0	0	0	1	68
**1.115**	0	0	1	0	0	0	0	0	0	1	0	1	0	1	0	0	0	0	0	1	0	1	0	1	0	68
**1.116**	0	1	0	0	1	0	0	0	0	1	0	1	0	0	0	0	0	0	0	0	0	0	1	1	1	68
**1.117**	0	0	0	0	1	0	0	0	0	1	0	1	1	0	0	0	0	0	0	0	0	0	1	0	0	68
**1.118**	0	0	1	0	0	0	0	0	0	1	0	0	0	1	0	0	0	0	0	0	0	0	0	0	0	68
**1.119**	0	0	1	0	0	0	0	0	1	0	0	1	0	0	0	0	0	0	0	0	0	1	0	1	0	67
**1.120**	0	1	0	0	1	0	0	0	0	0	0	1	0	0	0	0	0	0	0	1	0	0	0	1	0	67
**1.121**	0	0	0	0	0	0	0	0	0	1	0	1	0	0	0	0	0	0	0	1	0	0	1	0	1	67
**1.122**	0	0	0	0	0	0	0	0	0	1	0	0	1	0	0	0	0	0	0	0	0	0	0	0	0	67
**1.123**	0	0	0	0	0	0	1	0	0	1	0	1	0	1	0	0	0	0	0	0	0	1	0	1	0	67
**1.124**	0	0	0	0	0	0	0	0	0	0	1	0	0	0	0	0	0	0	0	0	0	1	1	1	1	67
**1.125**	0	0	0	0	0	0	1	0	0	0	0	1	0	0	0	1	0	0	0	1	0	0	0	1	1	66
**1.126**	0	0	0	0	0	0	0	0	0	0	0	0	1	1	0	0	0	0	0	0	0	1	0	0	0	66
**1.127**	0	0	0	0	1	0	0	0	0	0	0	1	0	0	0	0	0	0	0	0	0	1	1	0	1	66
**1.128**	0	0	0	1	1	0	0	0	0	1	0	1	0	0	0	0	0	0	0	0	0	0	0	1	1	65
**1.129**	0	0	1	0	0	0	1	0	0	1	0	0	0	0	0	0	0	0	0	0	0	0	0	0	0	65
**1.130**	0	1	0	0	0	0	0	0	1	1	0	1	0	0	0	0	0	0	0	0	0	0	0	0	1	65
**1.131**	0	0	1	0	0	0	1	0	0	0	0	0	0	0	0	0	0	0	0	0	0	0	0	1	0	65
**1.132**	0	1	0	1	1	0	0	0	1	0	0	1	1	0	0	0	0	0	0	1	0	0	1	0	0	65
**1.133**	0	1	0	0	1	0	0	0	0	1	0	1	1	1	0	0	0	0	0	1	0	0	0	0	0	65
**1.134**	0	1	0	0	1	0	0	0	0	1	0	1	0	1	0	0	0	0	0	1	0	0	0	0	0	65
**1.135**	0	1	0	0	1	0	0	0	0	0	0	1	1	0	0	0	0	0	0	0	0	1	1	0	0	65
**1.136**	0	0	1	0	0	0	0	0	0	1	0	1	0	0	0	0	0	0	0	0	0	1	0	1	1	65
**1.137**	0	0	0	0	0	0	1	0	0	1	0	1	0	1	0	0	0	0	0	0	0	1	0	1	1	65
**1.138**	0	0	0	0	0	0	1	0	0	1	0	1	0	0	0	0	0	0	0	1	0	0	0	0	1	65
**1.139**	0	0	0	0	0	0	0	0	1	0	0	1	0	1	0	0	0	0	0	1	0	1	1	0	0	65
**1.140**	0	0	1	0	0	0	0	0	1	1	0	1	0	0	0	0	0	0	0	1	0	1	0	1	1	64
**1.141**	0	1	0	1	1	0	1	0	0	0	0	1	0	0	0	0	0	0	0	0	0	0	0	0	1	64
**1.142**	0	0	0	1	0	0	0	0	0	0	0	0	0	1	0	0	0	0	0	0	0	0	0	1	1	64
**1.143**	0	1	0	1	0	0	0	0	0	1	0	1	0	0	0	0	0	0	0	0	0	0	0	1	0	64
**1.144**	0	0	0	0	0	0	1	0	0	0	0	0	1	1	0	0	0	0	0	0	0	1	0	0	0	64
**1.145**	0	1	0	1	1	0	0	0	0	1	0	1	1	0	0	0	0	0	0	1	0	0	1	0	0	64
**1.146**	0	1	0	0	1	0	1	0	0	0	0	1	0	0	0	0	0	0	0	0	0	0	1	0	0	64
**1.147**	0	0	0	0	0	0	0	0	1	0	0	0	1	0	0	1	0	0	0	0	0	0	0	0	0	64
**1.148**	0	1	0	0	1	0	0	0	0	0	0	1	0	0	0	0	0	0	0	1	0	1	1	0	1	63
**1.149**	0	1	0	1	0	0	0	0	0	1	0	1	0	0	0	0	0	0	0	0	0	0	0	1	1	63
**1.150**	0	1	0	0	1	0	0	0	1	0	0	1	0	0	0	0	0	0	0	0	0	0	1	1	0	63
**1.151**	0	0	0	0	0	0	1	0	1	0	0	0	0	0	0	0	0	0	0	1	0	0	0	1	1	63
**1.152**	0	0	0	0	0	0	1	0	0	0	0	0	0	1	0	0	0	0	0	1	0	1	0	0	1	63
**1.153**	0	0	0	0	1	0	1	0	1	0	0	1	0	0	0	0	0	0	0	1	0	0	0	1	1	62
**1.154**	0	0	1	0	0	0	0	0	0	0	0	0	0	0	0	0	0	0	0	1	0	1	0	1	1	62
**1.155**	0	0	0	0	0	0	0	0	0	0	0	0	1	0	0	0	0	0	0	0	0	0	1	1	0	62
**1.156**	0	1	0	0	1	0	0	0	1	1	0	1	0	0	0	0	0	0	0	0	0	0	1	1	1	62
**1.157**	0	0	0	0	0	0	1	0	0	1	0	1	0	1	0	0	0	0	0	1	0	1	1	1	0	62
**1.158**	0	0	0	0	0	0	0	0	1	0	0	0	0	1	0	0	0	0	0	1	0	1	0	0	1	62
**1.159**	0	0	0	1	0	0	0	0	0	1	0	1	0	0	0	0	0	0	0	0	0	0	0	1	0	62
**1.160**	0	1	0	1	1	0	0	0	1	0	0	1	1	0	0	0	0	0	0	0	0	1	0	0	0	62
**1.161**	0	0	0	0	0	0	0	0	0	1	0	0	0	1	0	0	0	0	0	0	0	1	0	0	1	62
**1.162**	0	1	0	1	1	0	0	0	0	0	0	0	0	0	0	0	0	0	0	0	0	0	1	0	1	62
**1.163**	0	0	0	0	0	0	0	0	0	0	0	1	1	0	0	0	0	0	0	0	0	0	0	0	1	62
**1.164**	0	0	1	0	0	0	0	0	1	0	0	0	0	0	0	0	0	0	0	0	0	1	0	1	1	61
**1.165**	0	0	0	0	0	0	0	0	1	0	0	1	0	1	0	0	0	0	0	0	0	1	0	1	0	61
**1.166**	0	1	0	0	1	0	0	0	0	1	0	1	0	0	0	0	0	0	0	0	0	1	0	1	1	61
**1.167**	0	0	0	0	0	0	0	0	0	1	0	1	0	0	0	0	0	0	0	0	0	1	1	1	0	61
**1.168**	0	0	0	0	1	0	0	0	1	1	0	1	1	0	0	0	0	0	0	0	0	0	0	0	0	61
**1.169**	0	0	0	0	0	0	0	0	0	1	0	1	0	1	0	0	0	0	0	1	0	1	1	1	1	61
**1.170**	0	0	0	0	0	0	1	0	0	1	0	0	0	1	0	0	0	0	0	1	0	1	0	0	1	61
**1.171**	0	0	0	0	0	0	0	0	1	0	0	0	0	0	0	0	0	0	0	1	0	0	0	0	1	61
**1.172**	0	0	1	0	0	0	0	0	0	1	0	1	0	1	0	0	0	0	0	0	0	0	0	0	0	61
**1.173**	0	0	0	0	0	0	0	0	1	1	0	1	0	1	0	0	0	0	0	0	0	1	0	1	1	61
**1.174**	0	0	0	0	0	0	0	0	0	1	0	1	0	0	0	0	0	0	0	0	0	1	1	0	1	61
**1.175**	0	0	0	0	0	0	0	0	0	0	0	0	0	1	0	0	0	0	0	0	0	0	1	0	1	61
**1.176**	0	0	0	0	0	0	0	0	1	1	0	1	0	1	0	0	0	0	0	1	0	0	0	0	0	60
**1.177**	0	0	1	0	0	0	0	0	0	1	0	1	0	0	0	0	0	0	0	1	0	1	0	1	1	60
**1.178**	0	0	0	0	1	0	1	0	0	1	0	1	0	1	0	0	0	0	0	1	0	1	0	0	1	60
**1.179**	0	1	0	0	1	0	0	0	1	1	0	1	0	0	0	0	0	0	0	0	0	0	1	0	1	60
**1.180**	0	0	0	0	0	0	1	0	0	1	1	1	0	1	0	0	0	0	0	0	0	0	0	0	1	60
**1.181**	0	0	1	0	0	0	1	0	0	1	0	1	1	1	0	0	0	0	0	1	0	0	0	1	1	60
**1.182**	0	0	1	0	0	0	0	0	0	0	0	0	0	1	0	0	0	0	0	1	0	0	0	1	1	60
**1.183**	0	0	0	0	1	0	1	0	0	0	0	1	0	0	0	0	0	0	0	0	0	0	0	1	1	60
**1.184**	0	0	0	0	0	0	0	0	0	0	0	0	0	1	0	0	0	0	0	1	0	1	0	1	0	60
**1.185**	0	0	1	0	0	0	0	0	0	1	0	1	0	1	0	0	0	0	0	1	0	0	0	1	0	59
**1.186**	0	0	1	0	0	0	0	0	0	1	0	0	0	1	0	0	0	0	0	1	0	0	0	1	0	59
**1.187**	0	0	0	0	0	0	1	0	1	1	0	1	0	0	0	0	0	0	0	1	0	0	0	1	1	59
**1.188**	0	1	0	0	1	0	1	0	0	1	0	1	0	0	0	0	0	0	0	0	0	0	0	1	1	59
**1.189**	0	0	0	0	0	0	0	0	0	0	1	0	0	1	0	0	0	0	0	0	0	1	0	0	1	59
**1.190**	0	1	0	1	1	0	0	0	0	0	0	1	0	1	0	0	0	0	0	0	0	0	0	0	0	59
**1.191**	0	0	0	1	1	0	0	0	0	1	0	1	0	0	0	0	0	0	0	1	0	0	0	0	1	58
**1.192**	0	0	0	0	1	0	0	0	1	0	0	1	0	0	0	0	0	0	0	1	0	0	0	1	1	58
**1.193**	0	1	1	0	1	0	0	0	0	1	0	1	0	0	0	0	0	0	0	1	0	0	0	1	1	58
**1.194**	0	0	0	0	0	0	0	0	1	0	0	0	0	1	0	0	0	0	0	0	0	1	0	1	0	58
**1.195**	0	1	0	0	0	0	0	0	0	0	0	1	0	0	0	0	0	0	0	1	0	0	1	1	0	58
**1.196**	0	0	1	0	1	0	0	0	0	1	0	1	0	0	0	0	0	0	0	0	0	0	0	0	0	58
**1.197**	0	1	0	0	0	0	0	0	0	1	0	1	1	0	0	0	0	0	0	0	0	0	1	0	0	58
**1.198**	0	0	0	1	0	0	0	0	1	0	0	0	0	0	0	0	0	0	0	0	0	0	0	0	0	58
**1.199**	0	0	0	0	0	0	0	0	0	1	0	0	1	0	0	1	0	0	0	0	0	0	0	0	0	58
**1.200**	0	0	0	0	0	0	1	0	0	0	0	0	1	0	0	0	0	0	0	0	0	0	1	0	0	58
**1.201**	0	0	1	0	0	0	0	0	0	0	0	1	0	0	0	0	0	0	0	1	0	0	0	1	0	57
**1.202**	0	1	0	0	1	0	0	0	0	0	0	1	0	0	0	0	0	0	0	1	0	1	1	1	1	57
**1.203**	0	1	0	0	1	0	1	0	0	0	0	1	0	0	0	0	0	0	0	1	0	0	0	0	1	57
**1.204**	0	0	1	0	0	0	1	0	0	0	0	1	0	0	0	0	0	0	0	0	0	0	0	0	0	57
**1.205**	0	1	0	1	1	0	0	0	1	0	0	1	1	0	0	0	0	0	0	1	0	1	0	0	0	57
**1.206**	0	1	0	0	0	0	0	0	0	1	0	1	0	0	0	0	0	0	0	0	0	1	0	0	1	57
**1.207**	0	0	1	0	0	0	0	0	0	1	0	1	0	1	0	0	0	0	0	0	0	1	0	1	0	57
**1.208**	0	0	0	1	0	0	0	0	0	0	0	0	0	0	0	0	0	0	0	0	0	1	1	0	0	57
**1.209**	0	0	0	0	0	0	0	0	0	0	0	1	1	0	0	0	0	0	0	0	0	0	1	0	0	57
**1.210**	0	0	1	0	0	0	0	0	0	0	0	0	0	0	0	0	0	0	0	1	0	1	0	1	0	57
**1.211**	0	0	0	0	1	0	0	0	1	1	0	1	0	0	0	0	0	0	0	1	0	0	0	1	1	56
**1.212**	0	0	0	1	1	0	0	0	0	1	0	1	0	0	0	0	0	0	0	1	0	0	0	1	1	56
**1.213**	0	1	0	0	1	0	0	0	1	1	0	1	1	1	0	0	0	0	0	1	0	0	0	0	0	56
**1.214**	0	1	0	0	0	0	1	0	0	0	0	1	0	0	0	0	0	0	0	0	0	0	0	1	1	56
**1.215**	0	0	1	0	0	0	1	0	1	1	0	1	0	0	0	0	0	0	0	1	0	0	0	1	1	56
**1.216**	0	0	0	0	0	0	0	0	0	1	0	0	1	0	0	0	0	0	0	0	0	0	0	1	0	56
**1.217**	0	1	0	1	1	0	0	0	0	0	0	1	0	0	0	0	0	0	0	1	0	1	0	1	1	56
**1.218**	0	0	0	0	0	0	1	0	1	0	0	0	0	1	0	0	0	0	0	0	0	1	0	0	1	56
**1.219**	0	0	0	0	0	0	0	0	0	0	0	0	0	1	0	0	0	0	0	0	0	1	1	1	0	56

CMP	CD2	CD3	CD4	CD7	CD8	CD10	CD13	CD18	CD26	CD29	CD36	CD44	CD45RA	CD49f	CD54	CD56	CD57	CD58	CD62L	CD71	CD80	Cytokeratin	Histone_H1	HLA-DQ	HLA-DR	Freq. (voxel)
**1.220**	0	0	1	0	0	0	0	0	0	0	0	0	1	0	0	0	0	0	0	1	0	0	0	1	0	56
**1.221**	0	0	0	1	0	0	0	0	0	0	0	1	0	0	0	0	0	0	0	0	0	0	0	1	0	56
**1.222**	0	0	0	0	0	0	0	0	0	0	0	1	1	0	0	0	0	0	0	1	0	1	0	0	0	56
**1.223**	0	1	0	0	0	0	0	0	1	1	0	0	0	0	0	0	0	0	0	0	0	0	0	0	0	56
**1.224**	0	0	0	0	0	0	1	0	0	0	0	0	1	0	0	1	0	0	0	1	0	0	0	0	0	56
**1.225**	0	1	0	0	0	0	0	0	0	0	0	0	0	0	0	0	0	0	0	0	0	1	0	1	1	56
**1.226**	0	0	0	0	1	0	1	0	0	1	0	1	0	1	0	0	0	0	0	1	0	1	0	1	1	55
**1.227**	0	1	0	0	1	0	0	0	0	1	0	1	1	0	0	0	0	0	0	0	0	0	0	1	0	55
**1.228**	0	0	0	0	1	0	0	0	1	0	0	1	0	0	0	0	0	0	0	1	0	1	1	0	0	55
**1.229**	0	1	0	0	0	0	0	0	0	0	0	1	0	0	0	0	0	0	0	1	0	1	1	0	1	55
**1.230**	0	1	0	1	0	0	0	0	0	0	0	1	0	0	0	0	0	0	0	1	0	1	1	0	0	55
**1.231**	0	0	0	0	0	0	1	0	0	0	0	0	0	0	0	0	0	0	0	1	0	1	0	0	1	55
**1.232**	0	0	0	1	0	0	1	0	0	1	0	1	0	0	0	0	0	0	0	0	0	1	0	0	0	55
**1.233**	0	1	0	0	1	0	0	0	0	0	0	0	1	0	0	0	0	0	0	0	0	1	0	0	0	55
**1.234**	0	0	0	0	1	0	0	0	0	1	0	1	0	0	0	0	0	0	0	0	0	0	1	0	1	55
**1.235**	0	0	0	0	1	0	0	0	0	0	0	1	1	0	0	0	0	0	0	0	0	1	0	1	1	55
**1.236**	0	1	0	0	1	0	0	0	0	0	0	1	1	1	0	0	0	0	0	0	0	0	0	1	1	54
**1.237**	0	0	0	0	0	0	0	0	1	1	0	1	0	1	0	0	0	0	0	1	0	1	0	1	0	54
**1.238**	0	1	0	1	1	0	0	0	1	1	0	1	1	0	0	0	0	0	0	1	0	0	0	1	0	54
**1.239**	0	1	0	0	0	0	0	0	0	0	0	0	0	0	0	0	0	0	0	0	0	1	1	1	0	54
**1.240**	0	0	0	0	1	0	0	0	0	0	0	1	0	0	0	0	0	0	0	0	0	0	1	1	0	54
**1.241**	0	0	0	0	0	0	1	0	0	0	0	0	0	0	0	1	0	0	0	0	0	0	0	1	0	54
**1.242**	0	1	0	1	0	0	0	0	1	1	0	1	0	0	0	0	0	0	0	1	0	0	1	0	0	54
**1.243**	0	1	0	0	1	0	0	0	1	0	0	1	0	0	0	0	0	0	0	1	0	0	0	1	0	54
**1.244**	0	1	0	1	1	0	0	0	0	1	0	1	1	0	0	0	0	0	0	0	0	0	0	0	1	54
**1.245**	0	0	0	0	1	0	0	0	1	0	0	1	0	0	0	0	0	0	0	0	0	0	1	0	0	54
**1.246**	0	0	0	0	0	0	0	0	0	0	0	1	0	1	0	0	0	0	0	1	0	1	0	1	0	54
**1.247**	0	0	0	0	0	0	0	0	0	0	0	1	0	1	0	0	0	0	0	0	0	0	1	1	0	54
**1.248**	0	1	0	0	1	0	0	0	0	1	0	1	0	1	0	0	0	0	0	0	0	0	1	0	1	54
**1.249**	0	0	0	0	0	0	1	0	0	1	0	0	0	0	0	0	0	0	0	0	0	0	0	1	0	54
**1.250**	0	1	0	1	0	0	0	0	1	0	0	0	0	0	0	0	0	0	0	1	0	0	0	0	0	54
**1.251**	0	1	0	1	0	0	0	0	1	1	0	1	0	0	0	0	0	0	0	0	0	0	0	1	1	53
**1.252**	0	1	0	0	0	0	0	0	0	1	0	1	0	1	0	0	0	0	0	1	0	0	1	0	0	53
**1.253**	0	0	1	0	0	0	0	0	0	1	0	0	0	0	0	0	0	0	0	1	0	0	0	1	1	53
**1.254**	0	1	0	0	1	0	0	0	1	0	0	1	1	0	0	0	0	0	0	0	0	1	0	0	1	53
**1.255**	0	0	0	0	0	0	0	0	0	1	0	0	0	1	0	0	0	0	0	0	0	0	1	1	1	53
**1.256**	0	0	1	0	0	0	0	0	0	1	0	0	0	1	0	0	0	0	0	0	0	1	0	0	0	53
**1.257**	0	0	0	0	1	0	0	0	1	0	0	1	0	0	0	0	0	0	0	1	0	1	0	1	1	52
**1.258**	0	0	0	0	1	0	0	0	0	0	0	1	0	0	0	0	0	0	0	1	0	1	0	1	1	52
**1.259**	0	1	0	0	1	0	1	0	0	0	0	1	0	0	0	0	0	0	0	1	0	0	0	1	1	52
**1.260**	0	0	0	0	0	0	1	0	0	0	0	1	0	0	0	1	0	0	0	0	0	0	0	1	0	52
**1.261**	0	1	1	1	1	0	0	0	1	1	0	1	1	0	0	0	0	0	0	1	0	0	0	1	1	52
**1.262**	0	1	0	1	1	0	0	0	1	1	0	1	0	0	0	0	0	0	0	1	0	0	0	1	0	52
**1.263**	0	0	0	0	1	0	1	0	0	1	0	1	0	0	0	0	0	0	0	1	0	0	0	1	1	52
**1.264**	0	0	0	0	0	0	0	0	0	0	1	1	0	0	0	0	0	0	0	1	0	1	0	0	0	52
**1.265**	0	0	0	1	0	0	0	0	1	0	0	0	0	0	0	0	0	0	0	0	0	1	0	0	0	52
**1.266**	0	1	0	0	1	0	0	0	1	0	0	1	0	0	0	0	0	0	0	0	0	0	1	0	1	52
**1.267**	0	0	1	0	0	0	0	0	0	0	0	0	1	0	0	1	0	0	0	0	0	1	0	0	0	52
**1.268**	0	0	1	0	0	0	0	0	0	0	1	0	0	0	0	0	0	0	0	0	0	0	0	1	0	52
**1.269**	0	0	0	0	0	0	0	0	0	0	0	0	1	0	0	1	0	0	0	1	0	0	1	0	0	52
**1.270**	0	0	0	0	0	0	1	0	0	1	0	1	0	0	0	0	0	0	0	0	0	0	1	1	1	51
**1.271**	0	1	0	0	1	0	0	0	0	0	0	1	0	1	0	0	0	0	0	0	0	0	0	1	1	51
**1.272**	0	1	0	0	0	0	0	0	0	0	0	0	0	1	0	0	0	0	0	0	0	1	1	0	0	51
**1.273**	0	0	1	0	0	0	0	0	0	0	0	0	1	0	0	0	0	0	0	0	0	1	0	1	0	51
**1.274**	0	1	0	1	1	0	0	0	0	1	0	1	0	1	0	0	0	0	0	0	0	0	0	0	0	51
**1.275**	0	0	0	0	0	0	1	0	1	1	0	1	0	1	0	0	0	0	0	1	0	1	0	1	1	51
**1.276**	0	0	0	0	0	0	1	0	1	1	0	1	0	1	0	0	0	0	0	1	0	1	0	1	0	51
**1.277**	0	0	1	1	0	0	1	0	1	1	0	1	0	1	0	0	0	0	0	1	0	0	0	1	1	51
**1.278**	0	0	0	0	0	0	1	0	1	0	0	1	0	0	0	0	0	0	0	0	0	0	0	0	0	51
**1.279**	0	1	0	0	0	0	0	0	0	0	0	0	0	0	0	0	0	0	0	1	0	1	0	0	1	51
**1.280**	0	0	0	1	0	0	0	0	0	0	0	0	0	0	0	0	0	0	0	0	0	0	0	1	0	51
**1.281**	0	1	0	0	1	0	1	0	0	0	0	0	0	0	0	0	0	0	0	0	0	0	1	0	0	51
**1.282**	0	1	0	0	0	0	1	0	0	0	0	1	0	0	0	0	0	0	0	0	0	0	0	0	0	51
**1.283**	0	0	0	0	1	0	1	0	1	0	0	0	1	0	0	1	0	0	0	1	0	0	0	0	0	51
**1.284**	0	0	0	1	1	0	0	0	0	0	0	1	0	0	0	0	0	0	0	1	0	0	0	1	1	50
**1.285**	0	0	0	0	0	0	1	0	1	0	0	1	0	0	0	0	0	0	0	0	0	1	0	1	1	50
**1.286**	0	1	0	1	0	0	0	0	1	1	0	1	0	1	0	0	0	0	0	0	0	0	0	0	0	50
**1.287**	0	0	1	0	0	0	0	0	1	0	0	1	0	1	0	0	0	0	0	1	0	1	0	0	0	50
**1.288**	0	0	0	0	0	0	1	0	0	0	0	1	0	1	0	0	0	0	0	0	0	0	1	1	1	50
**1.289**	0	1	0	0	0	0	0	0	1	0	0	1	0	0	0	0	0	0	0	0	0	0	1	1	0	50
**1.290**	0	0	1	0	0	0	1	0	1	0	0	1	0	1	0	0	0	0	0	1	0	1	0	0	0	50
**1.291**	0	0	1	0	0	0	1	0	0	1	1	1	0	1	0	0	0	0	0	0	0	0	0	1	1	50
**1.292**	0	0	1	0	0	0	0	0	0	1	0	1	0	0	0	0	0	0	0	0	0	1	0	1	0	50
**1.293**	0	0	0	1	0	0	0	0	0	1	0	1	0	1	0	0	0	0	0	0	0	0	0	0	0	50
**1.294**	0	0	1	0	0	0	0	0	0	0	0	0	1	0	0	0	0	0	0	0	0	1	0	0	0	50
**1.295**	0	0	0	0	0	0	0	0	0	0	0	1	0	1	0	0	0	0	0	1	0	0	1	0	0	50
**1.296**	0	0	0	0	0	0	0	0	0	0	0	1	0	1	0	0	0	0	0	1	0	0	0	1	0	50
**1.297**	0	0	0	0	0	0	1	0	0	1	0	0	0	0	0	0	0	0	0	1	0	0	0	1	1	50
**1.298**	0	0	0	0	0	0	0	0	0	0	0	1	1	0	0	0	0	0	0	0	0	0	0	1	1	50
**1.299**	0	1	0	0	1	0	0	0	0	1	0	0	0	0	0	0	0	0	0	0	0	0	1	0	0	50
**1.300**	0	0	0	0	0	0	0	0	0	0	0	0	0	1	0	0	0	0	0	1	0	0	0	1	0	50
**1.301**	0	0	0	0	0	0	1	0	1	0	0	1	0	0	0	0	0	0	0	1	0	1	0	1	1	49
**1.302**	0	1	0	1	0	0	0	0	1	0	0	1	0	0	0	0	0	0	0	1	0	1	0	1	1	49
**1.303**	0	0	1	0	0	0	0	0	1	0	0	1	0	1	0	0	0	0	0	1	0	1	0	1	1	49
**1.304**	0	1	0	0	0	0	1	0	0	1	0	1	0	0	0	0	0	0	0	0	0	0	0	1	1	49
**1.305**	0	0	1	0	0	0	0	0	1	0	0	0	0	0	0	0	0	0	0	0	0	1	0	1	0	49
**1.306**	0	0	0	0	0	0	0	0	1	0	0	0	0	1	0	0	0	0	0	0	0	1	0	1	1	49
**1.307**	0	1	0	1	0	0	0	0	0	0	0	1	0	0	0	0	0	0	0	0	0	0	1	0	1	49
**1.308**	0	0	1	0	0	0	1	0	1	1	0	1	0	1	0	0	0	0	0	1	0	1	0	0	0	49
**1.309**	0	1	0	0	1	0	0	0	0	1	0	1	0	0	0	0	0	0	0	1	0	0	1	1	1	49
**1.310**	0	1	0	0	0	0	0	0	0	0	1	1	0	0	0	0	0	0	0	0	0	1	0	1	1	49
**1.311**	0	0	1	0	0	0	0	0	0	0	0	0	0	0	0	0	0	0	0	1	0	0	0	0	1	49
**1.312**	0	1	0	1	1	0	0	0	0	1	0	1	0	1	0	0	0	0	0	1	0	0	0	0	0	49
**1.313**	0	1	0	0	1	0	0	0	1	0	0	1	0	0	0	0	0	0	0	0	0	1	1	0	0	49
**1.314**	0	0	0	0	0	0	1	0	1	0	0	1	0	0	0	0	0	0	0	1	0	1	0	0	0	49
**1.315**	0	1	0	0	1	0	0	0	1	0	0	0	0	0	0	0	0	0	0	1	0	0	1	0	0	49
**1.316**	0	1	0	0	0	0	0	0	1	0	0	1	0	0	0	0	0	0	0	0	0	1	0	0	1	49
**1.317**	0	0	0	0	0	0	0	0	0	1	1	1	0	0	0	0	0	0	0	0	0	1	0	0	0	49
**1.318**	0	0	1	0	0	0	0	0	0	0	0	0	0	0	0	1	0	0	0	0	0	0	1	0	0	49
**1.319**	0	0	0	0	0	0	1	0	0	1	0	1	0	0	0	0	0	0	0	0	0	1	0	1	0	49
**1.320**	0	1	0	0	0	0	0	0	0	0	0	0	1	0	0	0	0	0	0	0	0	1	0	0	0	49
**1.321**	0	0	0	0	0	0	1	0	1	0	0	0	1	0	0	0	0	0	0	0	0	0	0	0	0	49
**1.322**	0	0	0	0	0	0	0	0	0	0	0	0	1	0	0	0	0	0	0	1	0	0	1	0	0	49
**1.323**	0	1	0	0	0	0	1	0	0	0	0	0	0	0	0	0	0	0	0	1	0	0	1	0	0	49
**1.324**	0	0	0	1	0	0	0	0	0	1	0	1	0	0	0	0	0	0	0	1	0	0	1	0	0	48
**1.325**	0	1	1	1	1	0	0	0	1	1	0	1	0	0	0	0	0	0	0	0	0	0	0	0	0	48
**1.326**	0	1	0	0	1	0	0	0	0	1	0	1	1	0	0	0	0	0	0	1	0	0	0	1	0	48
**1.327**	0	1	0	1	1	0	0	0	1	1	0	1	0	0	0	0	0	0	0	0	0	0	0	1	0	48
**1.328**	0	0	0	0	1	0	0	0	0	1	0	1	0	1	0	0	0	0	0	1	0	0	0	0	0	48
**1.329**	0	0	1	0	0	0	1	0	0	1	0	1	0	1	0	0	0	0	0	1	0	1	1	0	0	48
**1.330**	0	1	0	1	0	0	0	0	1	0	0	1	1	0	0	0	0	0	0	1	0	0	0	0	0	48
**1.331**	0	1	0	1	1	0	0	0	1	1	0	1	0	0	0	0	0	0	0	0	0	1	0	0	0	48
**1.332**	0	0	1	0	0	0	0	0	0	0	0	0	1	0	0	0	0	0	0	1	0	0	0	0	0	48
**1.333**	0	0	0	1	0	0	0	0	0	1	0	0	0	0	0	0	0	0	0	0	0	0	0	0	0	48
**1.334**	0	0	0	1	1	0	0	0	0	1	0	1	0	0	0	0	0	0	0	0	0	1	0	0	0	48
**1.335**	0	0	0	0	1	0	1	0	0	1	0	1	0	0	0	0	0	0	0	0	0	0	0	1	1	48
**1.336**	0	0	0	0	1	0	0	0	0	0	0	0	0	0	0	0	0	0	0	0	0	0	0	1	0	48
**1.337**	0	0	0	0	0	0	1	0	0	1	0	0	0	1	0	0	0	0	0	1	0	0	0	1	1	48
**1.338**	0	0	0	0	0	0	0	0	1	0	0	0	1	0	0	0	0	0	0	1	0	0	0	1	0	48
**1.339**	0	1	0	1	0	0	0	0	0	0	0	1	1	0	0	0	0	0	0	0	0	0	1	0	0	48
**1.340**	0	1	0	0	1	0	1	0	0	0	0	1	1	0	0	0	0	0	0	0	0	0	1	0	0	48
**1.341**	0	0	1	0	0	0	0	0	0	0	0	0	1	0	0	1	0	0	0	1	0	0	0	0	0	48

CMP	CD2	CD3	CD4	CD7	CD8	CD10	CD13	CD18	CD26	CD29	CD36	CD44	CD45RA	CD49f	CD54	CD56	CD57	CD58	CD62L	CD71	CD80	Cytokeratin	Histone_H1	HLA-DQ	HLA-DR	Freq. (voxel)
**1.342**	0	0	0	0	0	0	0	0	1	0	0	0	0	0	0	0	0	0	0	0	0	0	1	1	0	48
**1.343**	0	0	0	1	1	0	0	0	0	1	0	1	0	0	0	0	0	0	0	0	0	0	0	0	1	47
**1.344**	0	1	0	0	0	0	1	0	0	0	0	1	0	0	0	0	0	0	0	0	0	0	0	0	1	47
**1.345**	0	0	0	0	1	0	0	0	0	0	0	1	0	0	0	0	0	0	0	0	0	0	1	1	1	47
**1.346**	0	0	0	0	0	0	0	0	0	0	0	1	0	0	0	1	0	0	0	0	0	0	1	1	0	47
**1.347**	0	1	0	0	1	0	0	0	0	1	0	1	1	0	0	0	0	0	0	0	0	1	0	1	1	47
**1.348**	0	0	0	0	0	0	0	0	0	0	0	1	0	1	0	0	0	0	0	0	0	0	1	1	1	47
**1.349**	0	1	0	1	1	0	0	0	0	0	0	1	0	0	0	0	0	0	0	1	0	1	0	0	1	47
**1.350**	0	0	0	0	0	0	1	0	0	0	0	1	0	1	0	0	0	0	0	1	0	0	0	0	0	47
**1.351**	0	0	0	0	0	0	0	0	0	1	1	0	0	0	0	0	0	0	0	0	0	0	0	1	1	47
**1.352**	0	1	0	0	1	0	0	0	1	0	0	0	0	0	0	0	0	0	0	0	0	0	0	0	0	47
**1.353**	0	0	0	0	0	0	0	0	0	0	1	0	0	0	0	0	0	0	0	0	0	0	1	1	0	47
**1.354**	0	1	1	1	1	0	0	0	0	1	0	1	0	0	0	0	0	0	0	1	0	0	0	0	0	46
**1.355**	0	0	1	0	0	0	1	0	1	0	0	1	0	1	0	0	0	0	0	1	0	1	0	1	1	46
**1.356**	0	0	0	0	0	0	1	0	0	1	0	1	0	1	0	0	0	0	0	0	0	1	1	1	0	46
**1.357**	0	0	0	0	1	0	0	0	1	0	0	0	0	0	0	0	0	0	0	0	0	1	0	0	1	46
**1.358**	0	0	0	0	1	0	0	0	0	0	0	1	0	0	0	0	0	0	0	0	0	1	1	1	1	46
**1.359**	0	0	0	0	1	0	0	0	0	0	0	1	0	0	0	0	0	0	0	0	0	1	0	1	0	46
**1.360**	0	0	0	0	1	0	0	0	0	1	0	1	0	0	0	0	0	0	0	0	0	0	1	1	0	46
**1.361**	0	0	1	0	0	0	1	0	0	1	0	1	0	1	0	0	0	0	0	1	0	1	1	1	1	46
**1.362**	0	0	1	0	0	0	0	0	0	1	0	1	0	1	0	0	0	0	0	0	0	1	1	0	0	46
**1.363**	0	0	0	1	1	0	0	0	0	0	0	1	1	0	0	0	0	0	0	0	0	1	0	0	0	46
**1.364**	0	1	0	1	0	0	0	0	0	0	0	0	0	0	0	0	0	0	0	1	0	1	1	0	0	46
**1.365**	0	0	1	0	0	0	0	0	0	0	0	1	0	1	0	0	0	0	0	1	0	0	0	1	1	46
**1.366**	0	0	0	0	0	0	1	0	0	1	0	0	1	1	0	0	0	0	0	1	0	1	0	0	0	46
**1.367**	0	0	0	0	0	0	0	0	0	0	1	0	0	1	0	1	0	0	0	0	0	1	0	0	0	46
**1.368**	0	0	1	0	0	0	0	0	0	0	0	0	1	0	0	1	0	0	0	1	0	0	0	1	0	46
**1.369**	0	0	0	0	0	0	1	0	1	0	0	0	0	0	0	0	0	0	0	1	0	0	1	0	0	46
**1.370**	0	1	0	1	1	0	0	0	0	0	0	0	1	0	0	0	0	0	0	1	0	0	0	1	1	45
**1.371**	0	0	0	1	0	0	0	0	0	1	0	1	0	0	0	0	0	0	0	1	0	0	0	1	1	45
**1.372**	0	1	1	0	1	0	0	0	1	1	0	1	1	0	0	0	0	0	0	1	0	0	0	1	1	45
**1.373**	0	1	1	0	1	0	0	0	0	0	0	1	0	0	0	0	0	0	0	1	0	0	0	0	0	45
**1.374**	0	1	1	1	1	0	0	0	0	1	0	1	0	0	0	0	0	0	0	1	0	0	0	1	1	45
**1.375**	0	0	1	0	0	0	0	0	0	0	0	1	0	0	0	0	0	0	0	0	0	0	1	1	1	45
**1.376**	0	1	0	0	1	0	1	0	0	1	0	1	0	1	0	0	0	0	0	0	0	1	0	1	1	45
**1.377**	0	0	0	0	0	0	0	0	1	1	0	1	0	0	0	0	0	0	0	1	0	0	1	0	0	45
**1.378**	0	1	0	1	1	0	0	0	0	0	0	1	1	0	0	0	0	0	0	1	0	1	0	0	1	45
**1.379**	0	0	1	0	0	0	0	0	0	0	0	1	0	1	0	0	0	0	0	0	0	0	0	0	0	45
**1.380**	0	0	0	0	0	0	1	0	0	1	0	0	0	1	0	0	0	0	0	1	0	1	1	0	1	45
**1.381**	0	1	1	0	0	0	0	0	0	1	0	1	0	0	0	0	0	0	0	0	0	0	0	0	0	45
**1.382**	0	0	1	0	0	0	1	0	0	0	0	0	0	0	0	0	0	0	0	1	0	1	0	0	0	45
**1.383**	0	0	1	0	0	0	0	0	0	1	0	1	0	1	0	0	0	0	0	0	0	1	0	1	1	45
**1.384**	0	0	0	1	0	0	1	0	0	1	0	1	1	1	0	0	0	0	0	1	0	0	0	1	1	45
**1.385**	0	0	0	0	1	0	1	0	0	1	0	1	0	1	0	0	0	0	0	0	0	0	0	0	0	45
**1.386**	0	1	0	1	0	0	0	0	0	0	0	1	0	0	0	0	0	0	0	1	0	0	0	1	0	45
**1.387**	0	0	0	0	1	0	0	0	0	0	0	0	1	0	0	0	0	0	0	0	0	1	0	0	1	45
**1.388**	0	1	0	1	1	0	0	0	0	0	0	1	1	1	0	0	0	0	0	0	0	0	0	0	0	45
**1.389**	0	0	1	0	1	0	0	0	0	1	0	1	0	0	0	0	0	0	0	1	0	0	0	0	0	44
**1.390**	0	0	0	0	1	0	1	0	0	1	0	1	0	1	0	0	0	0	0	1	0	1	0	0	0	44
**1.391**	0	1	0	1	1	0	0	0	1	0	0	1	1	0	0	0	0	0	0	0	0	0	0	1	1	44
**1.392**	0	1	0	0	1	0	0	0	0	0	0	1	0	0	0	0	0	0	0	1	0	0	1	1	0	44
**1.393**	0	0	0	0	0	0	1	0	0	0	0	0	0	0	0	0	0	0	0	1	0	0	1	1	1	44
**1.394**	0	0	1	0	0	0	0	0	0	0	1	0	0	1	0	0	0	0	0	0	0	1	0	0	0	44
**1.395**	0	0	1	0	0	0	0	0	0	0	0	0	0	0	0	0	0	0	0	1	0	1	0	0	1	44
**1.396**	0	0	0	0	1	0	1	0	0	1	0	1	0	1	0	0	0	0	0	0	0	1	0	0	0	44
**1.397**	0	0	0	0	0	0	0	0	0	0	0	1	0	0	0	1	0	0	0	0	0	1	0	0	0	44
**1.398**	0	0	0	0	0	0	1	0	0	0	0	0	0	0	0	0	0	0	0	1	0	0	0	1	0	44
**1.399**	0	1	0	1	0	0	0	0	0	0	0	1	1	0	0	0	0	0	0	1	0	1	1	0	0	44
**1.400**	0	0	1	0	0	0	0	0	0	0	0	1	0	0	0	0	0	0	0	0	0	0	1	0	0	44
**1.401**	0	0	0	0	0	0	0	0	0	0	0	0	1	0	0	0	0	0	0	0	0	1	1	0	0	44
**1.402**	0	1	0	0	0	0	0	0	0	1	0	0	0	0	0	0	0	0	0	0	0	1	1	0	0	44
**1.403**	0	0	0	0	0	0	0	0	0	0	0	0	0	0	0	1	0	0	0	1	0	1	0	0	0	44
**1.404**	0	0	0	1	1	0	0	0	1	0	0	1	0	0	0	0	0	0	0	1	0	0	0	0	0	43
**1.405**	0	1	0	1	1	0	0	0	0	0	0	1	0	0	0	0	0	0	0	1	0	0	1	1	1	43
**1.406**	0	0	0	1	1	0	0	0	1	0	0	1	0	0	0	0	0	0	0	1	0	1	1	0	0	43
**1.407**	0	0	0	0	1	0	0	0	0	0	0	0	0	0	0	0	0	0	0	1	0	1	1	0	0	43
**1.408**	0	1	0	0	1	0	0	0	0	1	0	1	0	1	0	0	0	0	0	0	0	1	1	1	1	43
**1.409**	0	0	0	1	0	0	0	0	1	0	0	1	0	0	0	0	0	0	0	1	0	1	1	0	0	43
**1.410**	0	1	0	1	1	0	0	0	0	1	0	1	1	1	0	0	0	0	0	0	0	0	0	0	0	43
**1.411**	0	0	1	0	0	0	1	0	0	1	0	1	0	1	0	0	0	0	0	1	0	1	0	1	0	43
**1.412**	0	0	0	0	0	0	0	0	0	1	1	1	0	0	0	0	0	0	0	1	0	0	0	0	1	43
**1.413**	0	0	0	0	0	0	0	0	0	0	1	1	0	1	0	0	0	0	0	1	0	1	0	0	0	43
**1.414**	0	1	0	1	1	0	0	0	1	1	0	1	0	0	0	0	0	0	0	0	0	0	1	0	1	43
**1.415**	0	1	0	0	0	0	0	0	0	0	0	1	0	1	0	0	0	0	0	0	0	0	1	0	1	43
**1.416**	0	0	0	0	1	0	0	0	1	0	0	1	0	0	0	0	0	0	0	0	0	1	0	0	1	43
**1.417**	0	0	0	0	0	0	1	0	1	1	0	1	0	1	0	0	0	0	0	0	0	1	0	0	1	43
**1.418**	0	1	0	0	1	0	0	0	0	1	0	1	0	0	0	0	0	0	0	0	0	0	1	1	0	43
**1.419**	0	0	1	0	0	0	0	0	1	0	0	1	0	0	0	0	0	0	0	0	0	0	0	0	0	43
**1.420**	0	0	1	0	0	0	0	0	0	1	0	0	0	0	0	0	0	0	0	1	0	1	0	0	0	43
**1.421**	0	0	1	0	0	0	0	0	0	0	0	0	0	0	0	0	0	0	0	1	0	1	1	0	0	43
**1.422**	0	0	0	0	0	0	0	0	0	1	0	0	0	1	0	0	0	0	0	1	0	0	0	1	1	43
**1.423**	0	1	0	1	0	0	0	0	0	0	0	0	0	0	0	0	0	0	0	0	0	1	1	0	0	43
**1.424**	0	0	1	0	0	0	0	0	0	0	1	0	0	0	0	0	0	0	0	0	0	0	0	1	1	43
**1.425**	0	1	0	0	0	0	0	0	0	0	0	1	1	0	0	0	0	0	0	1	0	0	1	0	0	43
**1.426**	0	0	1	0	1	0	1	0	1	1	0	1	0	0	0	0	0	0	0	0	0	0	0	1	1	42
**1.427**	0	1	0	1	1	0	0	0	1	1	0	1	0	1	0	0	0	0	0	1	0	1	0	1	1	42
**1.428**	0	1	0	0	0	0	0	0	1	1	0	1	0	0	0	0	0	0	0	0	0	1	0	1	1	42
**1.429**	0	1	0	1	1	0	0	0	1	1	0	1	1	0	0	0	0	0	0	0	0	0	0	1	0	42
**1.430**	0	1	0	0	1	0	0	0	0	0	0	0	0	0	0	0	0	0	0	0	0	1	1	0	1	42
**1.431**	0	0	0	0	1	0	0	0	0	0	0	0	1	0	0	0	0	0	0	0	0	0	0	0	1	42
**1.432**	0	0	1	0	0	0	0	0	0	1	0	1	0	1	0	0	0	0	0	1	0	1	1	0	1	42
**1.433**	0	0	0	0	0	0	1	0	0	1	0	1	0	1	0	0	0	0	0	1	0	0	1	1	1	42
**1.434**	0	1	0	1	1	0	0	0	1	0	0	1	0	0	0	0	0	0	0	0	0	0	0	0	1	42
**1.435**	0	0	1	0	0	0	1	0	0	0	0	0	0	1	0	0	0	0	0	1	0	1	0	0	0	42
**1.436**	0	0	0	1	0	0	1	0	1	1	0	1	0	1	0	0	0	0	0	1	0	0	0	1	1	42
**1.437**	0	1	0	0	0	0	0	0	1	0	0	1	0	0	0	0	0	0	0	1	0	0	0	1	1	42
**1.438**	0	0	1	0	0	0	0	0	1	0	0	0	0	0	0	0	0	0	0	1	0	0	0	1	1	42
**1.439**	0	1	0	0	1	0	0	0	1	1	0	1	1	0	0	0	0	0	0	0	0	1	0	0	0	42
**1.440**	0	1	0	0	0	0	0	0	1	0	0	1	0	0	0	0	0	0	0	0	0	0	0	0	1	42
**1.441**	0	0	0	1	1	0	0	0	0	0	0	1	1	0	0	0	0	0	0	0	0	0	0	1	1	42
**1.442**	0	1	0	1	1	0	0	0	1	0	0	1	0	0	0	0	0	0	0	0	0	1	0	0	1	42
**1.443**	0	0	0	0	0	0	0	0	0	1	1	1	0	1	0	0	0	0	0	0	0	0	0	1	0	42
**1.444**	0	1	0	0	1	0	0	0	0	0	0	1	1	0	0	0	0	0	0	0	0	0	0	1	0	42
**1.445**	0	1	0	1	1	0	1	0	0	0	0	1	0	0	0	0	0	0	0	0	0	0	0	0	0	42
**1.446**	0	0	0	0	0	0	0	0	0	0	0	1	1	0	0	0	0	0	0	0	0	1	0	0	1	42
**1.447**	0	0	0	0	0	0	0	0	1	0	0	1	1	0	0	0	0	0	0	0	0	1	0	0	0	42
**1.448**	0	1	1	0	1	0	0	0	0	1	0	1	0	0	0	0	0	0	0	1	0	0	0	0	0	41
**1.449**	0	0	1	0	0	0	1	0	1	1	0	1	0	0	0	0	0	0	0	0	0	0	0	1	1	41
**1.450**	0	0	0	0	1	0	1	0	0	1	0	1	0	1	0	0	0	0	0	0	0	1	0	1	1	41
**1.451**	0	1	0	0	1	0	0	0	0	0	0	0	0	0	0	0	0	0	0	0	0	0	0	1	1	41
**1.452**	0	0	0	0	1	0	0	0	0	1	0	1	0	1	0	0	0	0	0	0	0	1	1	1	1	41
**1.453**	0	0	0	0	0	0	1	0	1	0	1	0	0	1	0	0	0	0	0	1	0	1	0	0	0	41
**1.454**	0	1	0	1	1	0	0	0	0	0	0	1	1	0	0	0	0	0	0	0	0	0	1	0	1	41
**1.455**	0	1	0	1	0	0	0	0	0	1	0	1	0	1	0	0	0	0	0	0	0	0	0	0	0	41
**1.456**	0	1	0	0	1	0	0	0	0	0	0	1	0	0	0	0	0	0	0	1	0	0	1	1	1	41
**1.457**	0	0	0	0	1	0	0	0	0	0	0	0	0	0	0	0	0	0	0	1	0	1	0	0	0	41
**1.458**	0	0	0	0	0	0	0	0	0	1	0	0	0	1	0	0	0	0	0	1	0	0	0	1	0	41
**1.459**	0	1	0	1	0	0	0	0	0	1	0	1	0	0	0	0	0	0	0	1	0	0	0	1	1	41
**1.460**	0	0	0	0	0	0	0	0	1	1	0	1	0	1	0	0	0	0	0	1	0	1	1	0	0	41
**1.461**	0	0	0	0	0	0	0	0	1	1	0	1	0	1	0	0	0	0	0	1	0	0	0	1	1	41
**1.462**	0	1	0	0	1	0	0	0	1	0	0	1	1	0	0	0	0	0	0	0	0	0	0	0	1	41
**1.463**	0	0	0	1	0	0	0	0	0	0	0	1	0	0	0	0	0	0	0	1	0	0	0	1	1	41

CMP	CD2	CD3	CD4	CD7	CD8	CD10	CD13	CD18	CD26	CD29	CD36	CD44	CD45RA	CD49f	CD54	CD56	CD57	CD58	CD62L	CD71	CD80	Cytokeratin	Histone_H1	HLA-DQ	HLA-DR	Freq. (voxel)
**1.464**	0	0	0	0	1	0	0	0	0	0	0	0	0	0	0	0	0	0	0	1	0	1	0	0	1	41
**1.465**	0	0	0	0	0	0	1	0	0	0	0	1	0	1	0	1	0	0	0	0	0	0	0	0	0	41
**1.466**	0	1	0	0	1	0	0	0	1	0	0	0	1	0	0	0	0	0	0	0	0	0	1	0	0	41
**1.467**	0	0	1	0	0	0	0	0	0	0	0	0	0	1	0	0	0	0	0	0	0	0	1	0	0	41
**1.468**	0	0	0	1	0	0	0	0	1	0	0	1	0	0	0	0	0	0	0	1	0	1	0	0	0	41
**1.469**	0	0	0	0	0	0	1	0	1	0	0	0	0	0	0	0	0	0	0	0	0	0	0	0	0	41
**1.470**	0	0	0	0	0	0	1	0	0	1	0	0	1	1	0	0	0	0	0	1	0	0	0	1	1	41
**1.471**	0	0	0	0	0	0	0	0	0	1	0	1	1	0	0	0	0	0	0	0	0	1	0	0	0	41
**1.472**	0	0	0	0	0	0	1	0	0	0	0	0	0	0	0	1	0	0	0	0	0	0	1	1	1	40
**1.473**	0	0	1	0	0	0	0	0	0	0	0	0	1	0	0	0	0	0	0	1	0	1	0	1	0	40
**1.474**	0	0	0	1	1	0	0	0	0	0	0	1	0	0	0	0	0	0	0	1	0	1	1	0	0	40
**1.475**	0	1	0	1	1	0	0	0	0	1	0	1	0	0	0	0	0	0	0	1	0	1	0	1	1	40
**1.476**	0	1	0	0	0	0	0	0	1	1	0	1	0	0	0	0	0	0	0	1	0	0	1	0	0	40
**1.477**	0	1	0	1	1	0	0	0	0	1	0	1	0	1	0	0	0	0	0	1	0	0	0	1	1	40
**1.478**	0	0	0	1	0	0	0	0	1	1	0	1	0	0	0	0	0	0	0	0	0	0	1	0	0	40
**1.479**	0	1	0	0	1	0	0	0	0	0	0	1	1	0	0	0	0	0	0	1	0	0	1	0	1	40
**1.480**	0	0	1	0	0	0	1	0	0	0	0	1	0	1	0	0	0	0	0	1	0	1	0	0	0	40
**1.481**	0	1	0	0	1	0	0	0	0	0	0	1	0	1	0	0	0	0	0	0	0	0	1	0	0	40
**1.482**	0	0	0	1	0	0	0	0	0	0	0	1	0	1	0	0	0	0	0	0	0	0	0	1	1	40
**1.483**	0	1	0	0	1	0	0	0	1	0	0	1	1	0	0	0	0	0	0	1	0	0	0	0	1	40
**1.484**	0	0	0	0	0	0	0	0	1	1	0	1	0	1	0	0	0	0	0	0	0	1	0	1	0	40
**1.485**	0	0	0	0	0	0	1	0	0	1	0	1	0	0	0	0	0	0	0	0	0	1	0	1	1	40
**1.486**	0	0	0	0	0	0	0	0	0	0	1	1	0	0	0	0	0	0	0	0	0	1	1	1	0	40
**1.487**	0	0	0	0	1	0	1	0	1	0	0	0	1	0	0	0	0	0	0	1	0	1	0	0	0	40
**1.488**	0	0	1	0	0	0	0	0	0	0	0	0	1	0	0	0	0	0	0	0	0	0	1	1	0	40
**1.489**	0	0	1	0	0	0	0	0	1	1	0	1	0	0	0	0	0	0	0	0	0	1	0	0	0	39
**1.490**	0	0	1	0	0	0	1	0	0	1	0	0	0	0	0	0	0	0	0	1	0	0	0	0	0	39
**1.491**	0	0	1	0	0	0	1	0	1	0	0	0	0	0	0	0	0	0	0	0	0	1	0	0	0	39
**1.492**	0	0	0	0	0	0	1	0	1	0	0	0	0	0	0	1	0	0	0	0	0	0	0	1	1	39
**1.493**	0	1	0	0	1	0	0	0	1	1	0	1	1	0	0	0	0	0	0	0	0	1	0	1	1	39
**1.494**	0	1	0	0	1	0	0	0	0	0	0	0	1	0	0	0	0	0	0	0	0	0	0	0	1	39
**1.495**	0	0	0	0	1	0	0	0	0	0	0	0	0	0	0	0	0	0	0	0	0	1	1	0	1	39
**1.496**	0	0	0	0	0	0	1	0	0	1	1	1	0	0	0	0	0	0	0	1	0	0	0	1	1	39
**1.497**	0	1	0	1	1	0	0	0	0	1	0	1	1	0	0	0	0	0	0	0	0	1	0	0	0	39
**1.498**	0	1	1	0	1	0	0	0	0	0	0	1	0	0	0	0	0	0	0	0	0	0	1	0	0	39
**1.499**	0	1	0	0	0	0	0	0	0	0	0	1	0	1	0	0	0	0	0	1	0	0	1	0	0	39
**1.500**	0	0	0	1	0	0	1	0	0	1	0	1	0	1	0	0	0	0	0	1	0	1	0	0	0	39
**1.501**	0	0	0	0	0	0	1	0	0	1	0	1	0	1	0	0	0	0	0	0	0	0	1	0	1	39
**1.502**	0	0	1	0	0	0	1	0	1	0	0	1	0	1	0	0	0	0	0	0	0	0	0	1	1	39
**1.503**	0	0	0	0	1	0	1	0	0	1	0	1	0	1	0	0	0	0	0	0	0	1	1	0	0	39
**1.504**	0	1	0	1	1	0	0	0	0	0	0	1	0	0	0	0	0	0	0	0	0	1	1	0	0	39
**1.505**	0	1	0	0	0	0	0	0	0	1	0	1	0	0	0	0	0	0	0	0	0	0	1	1	0	39
**1.506**	0	0	0	0	0	0	0	0	0	1	1	1	0	1	0	0	0	0	0	1	0	0	0	1	1	39
**1.507**	0	1	0	1	0	0	0	0	1	0	0	1	0	0	0	0	0	0	0	1	0	1	0	0	0	39
**1.508**	0	1	0	0	0	0	1	0	0	1	0	1	0	0	0	0	0	0	0	0	0	0	0	0	0	39
**1.509**	0	1	0	0	0	0	0	0	1	0	0	0	0	0	0	0	0	0	0	0	0	1	0	0	0	39
**1.510**	0	0	0	0	0	0	0	0	0	0	0	0	1	0	0	0	0	0	0	0	0	1	0	0	1	39
**1.511**	0	1	0	0	1	0	0	0	1	0	0	1	0	0	0	0	0	0	0	0	0	1	0	1	0	38
**1.512**	0	0	0	0	0	0	1	0	0	0	0	1	0	0	0	0	0	0	0	1	0	1	0	1	1	38
**1.513**	0	0	0	0	0	0	1	0	0	0	0	1	0	0	0	1	0	0	0	0	0	0	1	1	1	38
**1.514**	0	1	1	0	0	0	0	0	0	1	0	1	0	0	0	0	0	0	0	1	0	0	0	0	0	38
**1.515**	0	1	0	0	1	0	0	0	0	0	0	1	1	0	0	0	0	0	0	1	0	1	0	1	1	38
**1.516**	0	0	1	0	0	0	0	0	1	1	0	1	0	0	0	0	0	0	0	0	0	1	0	1	1	38
**1.517**	0	0	0	1	1	0	0	0	0	0	0	1	1	0	0	0	0	0	0	1	0	0	0	0	0	38
**1.518**	0	0	0	0	0	0	1	0	0	1	0	1	0	0	0	0	0	0	0	1	0	0	1	1	1	38
**1.519**	0	1	0	1	1	0	0	0	0	1	0	1	1	0	0	0	0	0	0	0	0	0	0	1	1	38
**1.520**	0	0	1	0	0	0	0	0	0	0	0	0	0	1	0	0	0	0	0	0	0	1	0	1	1	38
**1.521**	0	0	0	0	0	0	0	0	0	0	0	1	1	0	0	0	0	0	0	0	0	1	1	0	0	38
**1.522**	0	1	0	1	1	0	0	0	0	0	0	1	1	0	0	0	0	0	0	1	0	0	0	1	0	38
**1.523**	0	1	0	0	1	0	0	0	0	1	0	1	0	1	0	0	0	0	0	0	0	0	1	1	1	38
**1.524**	0	0	0	1	1	0	0	0	0	1	0	1	0	1	0	0	0	0	0	0	0	0	0	0	0	38
**1.525**	0	0	0	1	0	0	0	0	0	0	0	0	0	1	0	0	0	0	0	0	0	0	0	1	0	38
**1.526**	0	1	1	0	1	0	0	0	0	1	0	1	0	0	0	0	0	0	0	0	0	0	0	0	0	38
**1.527**	0	1	0	1	1	0	0	0	1	1	0	1	0	1	0	0	0	0	0	1	0	0	0	0	0	38
**1.528**	0	0	1	0	0	0	1	0	0	0	0	0	0	1	0	0	0	0	0	1	0	0	0	1	1	38
**1.529**	0	1	0	1	1	0	0	0	1	1	0	1	1	0	0	0	0	0	0	0	0	1	0	0	0	38
**1.530**	0	1	0	1	0	0	0	0	0	1	0	1	1	0	0	0	0	0	0	1	0	0	0	0	0	38
**1.531**	0	0	0	0	0	0	1	0	1	0	0	0	0	1	0	0	0	0	0	0	0	0	0	1	1	38
**1.532**	0	1	0	0	1	0	0	0	1	0	0	1	0	0	0	0	0	0	0	1	0	0	0	0	1	38
**1.533**	0	0	0	0	0	0	1	0	0	0	0	0	1	0	0	0	0	0	0	1	0	0	0	1	0	38
**1.534**	0	0	0	0	0	0	1	0	0	0	0	0	0	1	0	0	0	0	0	0	0	1	0	1	1	38
**1.535**	0	0	1	0	0	0	1	0	0	0	0	0	0	1	0	0	0	0	0	0	0	0	0	0	0	38
**1.536**	0	0	0	0	0	0	0	0	0	1	0	0	0	0	0	0	0	0	0	1	0	0	1	0	1	38
**1.537**	0	0	1	0	0	0	0	0	0	0	0	0	0	0	0	1	0	0	0	0	0	1	0	1	0	38
**1.538**	0	0	0	0	0	0	0	0	1	0	1	0	0	0	0	0	0	0	0	0	0	1	1	0	0	38
**1.539**	0	1	1	0	1	0	1	0	1	1	0	1	0	0	0	0	0	0	0	1	0	0	0	1	1	37
**1.540**	0	1	0	1	0	0	0	0	1	0	0	1	0	0	0	0	0	0	0	1	0	0	0	1	1	37
**1.541**	0	0	0	1	1	0	0	0	0	1	0	1	0	0	0	0	0	0	0	0	0	0	1	0	0	37
**1.542**	0	1	0	0	1	0	0	0	0	1	0	1	1	0	0	0	0	0	0	0	0	0	1	0	1	37
**1.543**	0	0	1	0	0	0	0	0	0	0	0	1	0	1	0	0	0	0	0	1	0	1	0	0	1	37
**1.544**	0	1	0	1	1	0	0	0	0	0	0	1	1	0	0	0	0	0	0	1	0	0	1	0	1	37
**1.545**	0	0	1	0	0	0	0	0	1	0	0	0	0	1	0	0	0	0	0	0	0	1	0	0	0	37
**1.546**	0	0	0	0	1	0	0	0	1	0	0	1	1	0	0	0	0	0	0	0	0	0	0	0	0	37
**1.547**	0	1	0	1	1	0	0	0	1	1	0	1	1	0	0	0	0	0	0	0	0	0	1	0	0	37
**1.548**	0	0	1	0	0	0	0	0	1	0	0	1	0	1	0	0	0	0	0	0	0	1	0	0	0	37
**1.549**	0	0	0	0	1	0	0	0	1	1	0	1	0	0	0	0	0	0	0	0	0	0	1	0	0	37
**1.550**	0	0	0	0	0	0	1	0	0	0	1	0	0	1	0	1	0	0	0	0	0	1	0	0	0	37
**1.551**	0	1	0	1	0	0	0	0	0	0	0	0	1	0	0	0	0	0	0	0	0	0	1	0	0	37
**1.552**	0	0	0	0	0	0	0	0	1	0	0	1	0	0	0	0	0	0	0	0	0	0	1	1	0	37
**1.553**	0	0	0	0	0	0	0	0	0	0	1	0	1	1	0	0	0	0	0	0	0	1	0	0	0	37
**1.554**	0	0	0	0	0	0	1	0	1	0	0	0	0	0	0	0	0	0	0	0	0	0	0	1	0	37
**1.555**	0	0	0	0	0	0	1	0	0	1	0	0	0	1	0	0	0	0	0	1	0	0	0	0	1	37
**1.556**	0	0	1	1	0	0	0	0	0	0	0	0	0	0	0	0	0	0	0	0	0	0	0	0	0	37
**1.557**	0	0	1	0	0	0	1	0	0	0	0	0	0	0	0	0	0	0	0	0	0	0	1	0	0	37
**1.558**	0	0	0	0	0	0	0	0	0	1	0	0	0	0	0	0	0	0	0	1	0	0	1	1	0	37
**1.559**	0	0	1	0	0	0	0	0	0	0	0	0	0	0	0	1	0	0	0	0	0	0	0	1	1	37
**1.560**	0	1	0	0	0	0	0	0	1	0	0	0	0	0	0	0	0	0	0	1	0	0	1	0	0	37
**1.561**	0	1	0	0	0	0	1	0	0	1	0	1	0	0	0	0	0	0	0	1	0	0	1	0	0	37
**1.562**	0	0	1	0	0	0	0	0	0	0	0	0	1	0	0	1	0	0	0	0	0	0	1	0	0	37
**1.563**	0	0	0	0	1	0	0	0	0	1	0	1	0	0	0	0	0	0	0	0	0	1	0	1	0	36
**1.564**	0	1	0	0	1	0	0	0	1	1	0	1	1	1	0	0	0	0	0	0	0	0	0	1	1	36
**1.565**	0	0	0	0	1	0	0	0	0	1	0	1	1	0	0	0	0	0	0	0	0	0	0	1	1	36
**1.566**	0	0	0	0	1	0	0	0	0	1	0	1	0	0	0	0	0	0	0	1	0	1	0	1	1	36
**1.567**	0	0	1	0	0	0	1	0	0	0	0	1	0	0	0	1	0	0	0	1	0	0	0	1	1	36
**1.568**	0	1	1	0	1	0	0	0	0	1	0	1	0	0	0	0	0	0	0	1	0	0	1	0	0	36
**1.569**	0	1	0	1	0	0	0	0	0	0	0	1	0	0	0	0	0	0	0	1	0	0	0	1	1	36
**1.570**	0	1	0	0	0	0	1	0	0	1	0	1	0	0	0	0	0	0	0	1	0	0	0	1	1	36
**1.571**	0	1	0	1	1	0	0	0	1	1	0	1	1	0	0	0	0	0	0	0	0	0	0	1	1	36
**1.572**	0	1	0	0	0	0	0	0	0	0	1	1	0	0	0	0	0	0	0	1	0	1	0	1	1	36
**1.573**	0	0	0	0	0	0	1	0	0	0	0	1	0	0	0	0	0	0	0	0	0	0	1	1	1	36
**1.574**	0	0	0	0	0	0	1	0	0	1	0	1	0	1	0	0	0	0	0	1	0	1	1	1	1	36
**1.575**	0	0	0	1	0	0	0	0	0	1	0	1	0	1	0	0	0	0	0	0	0	1	0	0	0	36
**1.576**	0	0	0	0	0	0	1	0	1	1	0	1	0	1	0	0	0	0	0	1	0	0	0	0	0	36
**1.577**	0	1	0	1	1	0	0	0	1	0	0	1	1	0	0	0	0	0	0	0	0	0	0	0	1	36
**1.578**	0	0	1	1	0	0	1	0	0	1	0	1	0	1	0	0	0	0	0	0	0	0	0	1	1	36
**1.579**	0	0	0	0	0	0	0	0	0	1	0	1	1	1	0	0	0	0	0	0	0	1	0	0	0	36
**1.580**	0	0	1	0	0	0	0	0	0	1	0	1	0	1	0	0	0	0	0	0	0	1	0	0	1	36
**1.581**	0	0	1	0	0	0	0	0	0	0	0	0	0	1	0	0	0	0	0	0	0	1	0	1	0	36
**1.582**	0	0	0	0	0	0	0	0	0	1	0	0	0	1	0	0	0	0	0	0	0	1	1	0	1	36
**1.583**	0	1	0	0	1	0	0	0	0	1	0	1	0	1	0	0	0	0	0	0	0	0	0	1	1	36
**1.584**	0	1	0	1	1	0	0	0	0	0	0	1	1	0	0	0	0	0	0	0	0	1	0	0	1	36
**1.585**	0	1	0	1	1	0	0	0	0	0	0	0	0	0	0	0	0	0	0	1	0	0	1	0	1	36

CMP	CD2	CD3	CD4	CD7	CD8	CD10	CD13	CD18	CD26	CD29	CD36	CD44	CD45RA	CD49f	CD54	CD56	CD57	CD58	CD62L	CD71	CD80	Cytokeratin	Histone_H1	HLA-DQ	HLA-DR	Freq. (voxel)
**1.586**	0	0	0	1	0	0	0	0	0	1	0	0	0	0	0	0	0	0	0	1	0	0	0	0	0	36
**1.587**	0	0	0	0	1	0	1	0	0	1	0	1	0	1	0	0	0	0	0	1	0	1	1	0	0	36
**1.588**	0	0	0	0	0	0	0	0	0	1	1	1	0	0	0	0	0	0	0	0	0	1	0	0	1	36
**1.589**	0	0	0	0	0	0	1	0	1	0	0	0	1	0	0	0	0	0	0	0	0	1	0	0	0	36
**1.590**	0	0	0	0	0	0	0	0	0	1	1	1	0	1	0	0	0	0	0	0	0	0	0	0	1	36
**1.591**	0	0	0	0	0	0	0	0	0	0	1	1	0	0	0	0	0	0	0	0	0	1	1	0	0	36
**1.592**	0	0	0	0	0	0	0	0	0	0	0	1	0	0	0	0	0	0	0	1	0	0	1	1	0	36
**1.593**	0	0	1	0	0	0	0	0	0	0	0	0	1	0	0	1	0	0	0	1	0	1	0	1	0	36
**1.594**	0	1	0	0	0	0	0	0	1	0	0	1	0	0	0	0	0	0	0	1	0	0	0	1	0	36
**1.595**	0	0	0	0	0	0	0	0	0	0	1	1	0	0	0	0	0	0	0	0	0	1	1	1	1	36
**1.596**	0	1	1	1	1	0	0	0	1	1	0	1	1	1	0	0	0	0	0	1	0	0	0	1	1	35
**1.597**	0	1	0	1	1	0	0	0	1	1	0	1	0	0	0	0	0	0	0	1	0	1	0	1	1	35
**1.598**	0	0	0	0	0	0	0	0	0	1	0	1	0	0	0	0	0	0	0	0	0	1	1	1	1	35
**1.599**	0	1	0	0	0	0	0	0	1	0	0	1	0	0	0	0	0	0	0	1	0	1	1	1	1	35
**1.600**	0	1	0	0	1	0	0	0	1	1	0	1	1	0	0	0	0	0	0	1	0	0	1	0	0	35
**1.601**	0	1	0	0	1	0	0	0	0	1	0	1	1	1	0	0	0	0	0	1	0	1	1	0	0	35
**1.602**	0	0	0	1	0	0	0	0	1	0	0	1	0	0	0	0	0	0	0	1	0	1	0	0	1	35
**1.603**	0	1	0	0	1	0	0	0	0	0	0	0	0	1	0	0	0	0	0	1	0	0	0	0	0	35
**1.604**	0	1	1	0	0	0	0	0	0	0	0	1	0	0	0	0	0	0	0	1	0	0	0	0	0	35
**1.605**	0	1	0	1	1	0	0	0	0	1	0	1	0	0	0	0	0	0	0	0	0	0	1	0	1	35
**1.606**	0	1	0	0	1	0	0	0	1	1	0	1	1	0	0	0	0	0	0	1	0	0	0	1	0	35
**1.607**	0	1	0	0	0	0	0	0	0	0	0	0	0	1	0	0	0	0	0	1	0	0	1	0	0	35
**1.608**	0	0	0	0	1	0	0	0	0	0	0	1	1	0	0	0	0	0	0	0	0	0	0	1	0	35
**1.609**	0	0	1	0	0	0	1	0	0	1	0	0	0	0	0	0	0	0	0	0	0	0	0	1	1	35
**1.610**	0	0	0	0	0	0	0	0	1	0	0	0	1	0	0	0	0	0	0	0	0	0	1	0	0	35
**1.611**	0	0	0	1	0	0	0	0	0	1	0	1	0	0	0	0	0	0	0	1	0	0	0	1	0	35
**1.612**	0	0	0	0	1	0	0	0	1	0	0	1	0	0	0	0	0	0	0	0	0	0	0	0	1	35
**1.613**	0	0	1	0	0	0	0	0	0	0	0	0	0	1	0	0	0	0	0	1	0	1	1	0	0	35
**1.614**	0	0	0	0	0	0	0	0	0	0	0	1	0	1	0	0	0	0	0	1	0	1	1	0	1	35
**1.615**	0	0	0	1	0	0	0	0	0	0	0	1	1	0	0	0	0	0	0	0	0	0	0	0	0	35
**1.616**	0	0	0	0	0	0	0	0	0	0	1	1	0	1	0	0	0	0	0	0	0	0	0	0	0	35
**1.617**	0	1	0	1	0	0	0	0	1	0	0	1	0	0	0	0	0	0	0	1	0	1	0	0	1	35
**1.618**	0	1	0	1	0	0	0	0	0	0	0	1	0	0	0	0	0	0	0	0	0	0	0	1	1	35
**1.619**	0	1	0	0	1	0	1	0	0	1	0	1	1	0	0	0	0	0	0	1	0	0	0	0	0	35
**1.620**	0	0	0	0	1	0	0	0	1	1	0	1	0	0	0	0	0	0	0	0	0	0	0	1	0	34
**1.621**	0	0	0	0	1	0	1	0	1	1	0	1	0	1	0	0	0	0	0	1	0	0	0	0	0	34
**1.622**	0	1	0	0	0	0	0	0	0	0	0	1	0	1	0	0	0	0	0	0	0	1	1	0	0	34
**1.623**	0	1	0	0	0	0	0	0	0	0	0	1	0	1	0	0	0	0	0	0	0	1	0	1	1	34
**1.624**	0	0	0	0	0	0	0	0	0	1	0	0	1	0	0	0	0	0	0	1	0	0	0	0	0	34
**1.625**	0	1	0	1	0	0	0	0	1	0	0	1	0	0	0	0	0	0	0	0	0	0	0	1	1	34
**1.626**	0	0	1	0	0	0	1	0	0	0	0	0	1	0	0	0	0	0	0	0	0	0	0	1	0	34
**1.627**	0	0	0	1	0	0	0	0	0	0	0	1	0	0	0	0	0	0	0	0	0	1	0	0	1	34
**1.628**	0	0	0	0	0	0	0	0	1	1	0	0	0	1	0	0	0	0	0	1	0	1	0	0	0	34
**1.629**	0	1	0	1	0	0	0	0	0	1	0	0	0	0	0	0	0	0	0	0	0	0	1	0	0	34
**1.630**	0	1	0	1	0	0	0	0	0	0	0	1	0	0	0	0	0	0	0	1	0	0	1	0	1	34
**1.631**	0	0	1	0	0	0	1	0	0	1	0	0	0	1	0	0	0	0	0	0	0	1	0	0	0	34
**1.632**	0	1	0	1	1	0	0	0	0	0	0	0	1	0	0	0	0	0	0	0	0	0	0	0	0	34
**1.633**	0	0	0	0	0	0	0	0	1	1	0	1	0	0	0	0	0	0	0	0	0	1	1	0	0	34
**1.634**	0	0	1	0	0	0	1	0	0	1	1	1	0	1	0	0	0	0	0	1	0	0	0	1	1	34
**1.635**	0	0	0	1	0	0	0	0	0	1	0	0	0	0	0	0	0	0	0	0	0	0	1	0	0	34
**1.636**	0	0	0	0	0	0	0	0	1	0	0	1	0	0	0	0	0	0	0	1	0	0	0	1	0	34
**1.637**	0	0	0	0	0	0	0	0	0	0	0	1	0	0	0	0	0	0	0	1	0	1	1	1	0	34
**1.638**	0	0	1	0	0	0	0	0	0	1	0	0	0	1	0	0	0	0	0	1	0	0	0	1	1	34
**1.639**	0	1	0	1	1	0	0	0	0	0	0	1	0	0	0	0	0	0	0	0	0	1	0	0	1	34
**1.640**	0	1	0	1	0	0	0	0	0	0	0	1	1	0	0	0	0	0	0	1	0	0	1	0	0	34
**1.641**	0	0	1	0	0	0	1	0	0	1	0	1	0	0	0	0	0	0	0	0	0	0	0	0	0	34
**1.642**	0	0	0	1	0	0	0	0	0	0	0	0	1	0	0	0	0	0	0	1	0	0	0	1	0	34
**1.643**	0	0	0	0	0	0	1	0	0	0	0	0	1	0	0	0	0	0	0	1	0	1	0	1	0	34
**1.644**	0	0	0	0	0	0	0	0	0	1	0	1	0	1	0	0	0	0	0	0	0	1	1	1	0	34
**1.645**	0	1	0	1	1	0	0	0	0	0	0	0	1	0	0	0	0	0	0	0	0	0	1	0	0	34
**1.646**	0	1	0	1	0	0	0	0	0	0	0	1	0	0	0	0	0	0	0	0	0	0	0	1	0	34
**1.647**	0	0	1	0	0	0	0	0	0	0	0	0	0	0	0	0	0	0	0	1	0	0	1	0	0	34
**1.648**	0	1	1	0	0	0	0	0	0	0	0	0	0	0	0	0	0	0	0	0	0	0	1	0	0	34
**1.649**	0	1	0	1	1	0	0	0	1	0	0	0	0	0	0	0	0	0	0	1	0	0	1	0	0	34
**1.650**	0	0	0	0	0	0	0	0	0	1	1	0	0	0	0	0	0	0	0	0	0	0	0	1	0	34
**1.651**	0	1	0	1	1	0	1	0	1	0	0	1	0	0	0	0	0	0	0	0	0	0	1	1	1	33
**1.652**	0	1	0	1	0	0	0	0	1	0	0	1	0	0	0	0	0	0	0	0	0	1	0	1	1	33
**1.653**	0	0	0	1	1	0	0	0	0	1	0	1	0	0	0	0	0	0	0	0	0	0	0	1	0	33
**1.654**	0	0	0	0	1	0	0	0	1	1	0	1	0	1	0	0	0	0	0	1	0	0	0	0	0	33
**1.655**	0	0	0	0	1	0	0	0	0	0	0	1	0	0	0	0	0	0	0	1	0	0	0	1	0	33
**1.656**	0	1	0	0	1	0	0	0	1	0	0	1	1	0	0	0	0	0	0	1	0	1	0	1	1	33
**1.657**	0	1	0	0	1	0	0	0	0	1	0	1	1	1	0	0	0	0	0	1	0	0	1	0	0	33
**1.658**	0	1	0	0	0	0	0	0	1	0	0	1	0	0	0	0	0	0	0	0	0	1	1	1	1	33
**1.659**	0	0	0	0	0	0	0	0	0	0	0	0	0	1	0	0	0	0	0	1	0	1	0	1	1	33
**1.660**	0	1	1	1	1	0	0	0	0	1	0	1	1	0	0	0	0	0	0	1	0	0	0	1	1	33
**1.661**	0	1	1	1	1	0	0	0	0	0	0	1	1	0	0	0	0	0	0	1	0	0	0	0	1	33
**1.662**	0	0	0	0	1	0	0	0	0	1	0	1	1	0	0	0	0	0	0	0	0	0	0	1	0	33
**1.663**	0	1	0	0	0	0	0	0	0	1	0	1	1	1	0	0	0	0	0	0	0	0	0	0	0	33
**1.664**	0	0	0	0	0	0	1	0	0	0	0	1	0	0	0	0	0	0	0	0	0	0	1	0	1	33
**1.665**	0	1	0	0	1	0	0	0	0	1	0	1	1	0	0	0	0	0	0	1	0	0	0	0	1	33
**1.666**	0	0	0	0	0	0	0	0	0	1	0	1	1	0	0	0	0	0	0	0	0	0	1	0	0	33
**1.667**	0	0	1	0	0	0	1	0	1	0	0	0	0	0	0	0	0	0	0	1	0	0	0	1	1	33
**1.668**	0	0	1	0	0	0	0	0	0	0	0	1	0	1	0	0	0	0	0	0	0	1	0	1	1	33
**1.669**	0	1	0	0	1	0	0	0	1	0	0	1	1	0	0	0	0	0	0	1	0	0	0	0	0	33
**1.670**	0	0	0	1	1	0	0	0	0	0	0	0	0	0	0	0	0	0	0	0	0	0	1	0	1	33
**1.671**	0	0	0	1	0	0	0	0	0	0	0	0	0	0	0	0	0	0	0	0	0	1	0	0	1	33
**1.672**	0	0	0	1	1	0	0	0	0	0	0	1	0	0	0	0	0	0	0	1	0	0	0	0	1	32
**1.673**	0	0	0	1	1	0	0	0	1	1	0	1	0	0	0	0	0	0	0	0	0	1	0	0	0	32
**1.674**	0	0	0	0	0	0	1	0	1	0	0	1	0	1	0	0	0	0	0	1	0	1	0	1	1	32
**1.675**	0	0	0	0	0	0	0	0	1	1	0	0	0	0	0	0	0	0	0	0	0	1	0	1	1	32
**1.676**	0	0	1	0	0	0	0	0	1	1	0	1	0	0	0	0	0	0	0	0	0	0	0	0	0	32
**1.677**	0	0	0	0	0	0	1	0	1	1	0	0	0	1	0	0	0	0	0	1	0	1	0	0	0	32
**1.678**	0	1	0	0	0	0	0	0	0	1	0	1	0	1	0	0	0	0	0	1	0	0	0	1	0	32
**1.679**	0	1	0	0	0	0	0	0	0	1	0	1	0	1	0	0	0	0	0	1	0	0	0	0	0	32
**1.680**	0	0	1	0	0	0	1	0	0	1	0	1	0	1	0	0	0	0	0	0	0	1	0	0	1	32
**1.681**	0	0	1	0	0	0	0	0	1	0	0	1	0	0	0	0	0	0	0	1	0	0	0	1	1	32
**1.682**	0	0	0	1	0	0	1	0	0	1	0	1	0	1	0	0	0	0	0	1	0	1	1	0	0	32
**1.683**	0	0	0	1	0	0	0	0	0	1	0	1	0	0	0	0	0	0	0	0	0	0	0	0	1	32
**1.684**	0	0	0	0	0	0	1	0	1	1	0	0	0	0	0	0	0	0	0	0	0	0	0	0	0	32
**1.685**	0	1	0	1	0	0	0	0	0	0	0	1	0	1	0	0	0	0	0	1	0	0	0	0	0	32
**1.686**	0	1	0	0	1	0	0	0	0	0	0	0	0	0	0	0	0	0	0	1	0	0	1	0	1	32
**1.687**	0	0	0	0	0	0	1	0	0	1	0	1	1	1	0	0	0	0	0	1	0	0	0	1	1	32
**1.688**	0	0	0	0	0	0	1	0	0	0	0	1	1	0	0	0	0	0	0	0	0	0	0	0	0	32
**1.689**	0	1	0	0	1	0	0	0	0	1	0	1	0	0	0	0	0	0	0	0	0	1	1	0	0	32
**1.690**	0	0	1	0	0	0	0	0	0	0	0	1	0	0	0	1	0	0	0	0	0	0	0	1	1	32
**1.691**	0	0	0	0	1	0	0	0	1	0	0	0	0	0	0	0	0	0	0	0	0	0	1	0	0	32
**1.692**	0	0	0	0	0	0	1	0	0	1	0	0	0	0	0	0	0	0	0	1	0	1	0	0	0	32
**1.693**	0	0	0	1	0	0	0	0	1	0	0	0	0	0	0	0	0	0	0	1	0	1	0	0	0	32
**1.694**	0	0	0	0	1	0	1	0	0	1	0	1	0	0	0	0	0	0	0	0	0	0	0	0	1	32
**1.695**	0	1	0	1	1	0	0	0	1	0	0	1	0	0	0	0	0	0	0	0	0	0	1	0	1	32
**1.696**	0	1	0	0	0	0	1	0	0	1	0	0	0	0	0	0	0	0	0	0	0	0	1	0	0	32
**1.697**	0	1	0	0	0	0	0	0	1	0	0	0	0	0	0	0	0	0	0	1	0	0	0	0	0	32
**1.698**	0	1	0	0	1	0	0	0	1	0	0	1	1	0	0	0	0	0	0	0	0	1	0	1	0	31
**1.699**	0	0	0	1	1	0	0	0	1	1	0	1	1	0	0	0	0	0	0	1	0	0	0	0	0	31
**1.700**	0	0	1	0	1	0	1	0	1	0	0	1	0	0	0	0	0	0	0	1	0	0	0	1	1	31
**1.701**	0	0	0	1	1	0	0	0	1	0	0	1	0	0	0	0	0	0	0	1	0	0	0	1	1	31
**1.702**	0	0	0	1	0	0	0	0	1	0	0	1	0	0	0	0	0	0	0	0	0	0	1	0	0	31
**1.703**	0	0	0	0	0	0	0	0	1	0	0	1	0	1	0	0	0	0	0	1	0	0	0	0	0	31
**1.704**	0	0	0	0	1	0	1	0	0	1	0	1	0	0	0	0	0	0	0	0	0	1	0	1	1	31
**1.705**	0	0	0	0	1	0	1	0	0	0	0	1	0	0	0	0	0	0	0	1	0	0	0	1	1	31
**1.706**	0	1	0	0	0	0	0	0	0	0	0	1	1	0	0	0	0	0	0	1	0	0	0	0	1	31
**1.707**	0	1	1	0	1	0	0	0	1	1	0	1	0	0	0	0	0	0	0	1	0	0	0	1	1	31

CMP	CD2	CD3	CD4	CD7	CD8	CD10	CD13	CD18	CD26	CD29	CD36	CD44	CD45RA	CD49f	CD54	CD56	CD57	CD58	CD62L	CD71	CD80	Cytokeratin	Histone_H1	HLA-DQ	HLA-DR	Freq. (voxel)
**1.708**	0	1	0	0	0	0	0	0	0	0	0	0	0	1	0	0	0	0	0	0	0	0	0	1	1	31
**1.709**	0	0	1	0	0	0	0	0	1	0	0	0	0	1	0	0	0	0	0	1	0	1	0	0	0	31
**1.710**	0	1	0	0	1	0	1	0	0	1	0	1	0	1	0	0	0	0	0	1	0	1	0	1	1	31
**1.711**	0	1	0	0	1	0	0	0	0	0	0	1	0	0	0	0	0	0	0	0	0	1	1	1	1	31
**1.712**	0	0	1	0	0	0	1	0	0	0	0	1	0	0	0	0	0	0	0	1	0	0	0	0	0	31
**1.713**	0	0	0	0	0	0	0	0	0	0	0	0	1	0	0	1	0	0	0	0	0	0	1	1	0	31
**1.714**	0	1	1	0	1	0	0	0	0	0	0	0	0	0	0	0	0	0	0	0	0	0	1	0	0	31
**1.715**	0	1	0	1	0	0	0	0	1	0	0	1	0	0	0	0	0	0	0	1	0	0	1	0	0	31
**1.716**	0	0	1	0	0	0	0	0	1	1	0	1	0	1	0	0	0	0	0	0	0	1	0	0	0	31
**1.717**	0	0	0	0	0	0	0	0	0	0	1	0	0	1	0	0	0	0	0	0	0	1	0	1	1	31
**1.718**	0	0	0	0	0	0	0	0	0	0	1	0	0	0	0	0	0	0	0	1	0	1	0	0	1	31
**1.719**	0	1	0	1	0	0	0	0	0	1	0	1	0	0	0	0	0	0	0	0	0	1	0	0	0	31
**1.720**	0	1	0	0	0	0	1	0	0	0	0	1	0	0	0	0	0	0	0	1	0	0	1	0	0	31
**1.721**	0	0	1	0	1	0	1	0	1	1	0	1	0	0	0	0	0	0	0	1	0	0	0	1	1	30
**1.722**	0	1	0	1	1	0	0	0	1	0	0	1	1	0	0	0	0	0	0	1	0	1	0	1	1	30
**1.723**	0	0	0	1	1	0	0	0	1	0	0	1	0	0	0	0	0	0	0	0	0	0	0	1	1	30
**1.724**	0	0	0	1	0	0	0	0	0	0	0	1	0	0	0	0	0	0	0	0	0	1	0	1	0	30
**1.725**	0	0	0	1	0	0	0	0	0	0	0	1	0	0	0	0	0	0	0	1	0	0	0	1	0	30
**1.726**	0	0	0	0	0	0	1	0	0	0	0	0	0	0	0	0	0	0	0	1	0	1	0	1	1	30
**1.727**	0	0	1	0	0	0	0	0	0	0	0	0	0	1	0	0	0	0	0	1	0	0	0	1	0	30
**1.728**	0	0	0	0	1	0	0	0	0	1	0	1	0	1	0	0	0	0	0	0	0	1	0	1	1	30
**1.729**	0	0	1	0	0	0	1	0	0	0	0	0	1	0	0	1	0	0	0	0	0	0	0	1	0	30
**1.730**	0	1	1	0	1	0	0	0	1	1	0	1	0	0	0	0	0	0	0	0	0	0	0	1	1	30
**1.731**	0	1	0	1	0	0	0	0	0	1	0	1	0	0	0	0	0	0	0	1	0	1	0	0	0	30
**1.732**	0	0	1	0	1	0	0	0	1	0	0	1	0	0	0	0	0	0	0	1	0	0	0	1	1	30
**1.733**	0	0	1	0	1	0	0	0	0	1	0	0	0	0	0	0	0	0	0	0	0	0	0	1	1	30
**1.734**	0	1	0	0	1	0	0	0	0	1	0	0	0	0	0	0	0	0	0	0	0	0	0	0	0	30
**1.735**	0	1	0	0	0	0	0	0	0	1	0	1	0	1	0	0	0	0	0	0	0	1	0	0	0	30
**1.736**	0	1	0	0	1	0	0	0	1	0	0	1	1	0	0	0	0	0	0	0	0	0	1	0	1	30
**1.737**	0	0	1	0	0	0	1	0	0	1	0	0	0	1	0	0	0	0	0	0	0	0	0	1	1	30
**1.738**	0	0	0	0	0	0	1	0	0	1	0	1	0	1	0	0	0	0	0	0	0	0	1	1	0	30
**1.739**	0	0	1	1	0	0	1	0	1	1	0	1	0	1	0	1	0	0	0	1	0	0	0	1	1	30
**1.740**	0	1	0	0	1	0	0	0	0	1	0	1	0	1	0	0	0	0	0	0	0	0	1	0	0	30
**1.741**	0	1	0	0	0	0	0	0	0	0	0	1	0	1	0	0	0	0	0	0	0	0	0	0	1	30
**1.742**	0	0	1	0	0	0	1	0	0	1	0	1	0	1	0	0	0	0	0	1	0	1	1	1	0	30
**1.743**	0	1	0	1	1	0	0	0	0	0	0	1	0	0	0	0	0	0	0	0	0	0	1	1	0	30
**1.744**	0	1	0	0	1	0	0	0	0	1	0	1	0	1	0	0	0	0	0	0	0	0	0	0	1	30
**1.745**	0	0	1	0	0	0	0	0	1	1	0	0	0	0	0	0	0	0	0	1	0	0	0	1	0	30
**1.746**	0	0	1	0	0	0	0	0	0	1	1	1	0	0	0	0	0	0	0	0	0	0	0	1	1	30
**1.747**	0	1	0	0	1	0	0	0	0	1	0	1	1	0	0	0	0	0	0	1	0	1	0	0	0	30
**1.748**	0	1	0	0	0	0	0	0	1	0	0	0	0	0	0	0	0	0	0	0	0	1	0	0	1	30
**1.749**	0	0	0	0	0	0	0	0	0	1	0	0	0	0	0	0	0	0	0	1	0	1	0	1	0	30
**1.750**	0	1	0	0	1	0	1	0	0	0	0	1	1	0	0	0	0	0	0	0	0	0	0	0	0	30
**1.751**	0	1	0	0	0	0	0	0	0	0	0	1	1	0	0	0	0	0	0	1	0	0	0	0	0	30
**1.752**	0	1	1	1	1	0	0	0	1	1	0	1	0	0	0	0	0	0	0	1	0	0	0	0	1	29
**1.753**	0	0	0	1	0	0	0	0	1	0	0	1	0	0	0	0	0	0	0	0	0	1	1	0	0	29
**1.754**	0	1	1	0	1	0	1	0	1	1	0	1	0	0	0	0	0	0	0	0	0	0	0	1	1	29
**1.755**	0	0	0	0	1	0	0	0	0	0	0	0	0	0	0	0	0	0	0	1	0	0	0	1	1	29
**1.756**	0	1	0	1	0	0	0	0	1	0	0	1	0	1	0	0	0	0	0	0	0	0	0	0	0	29
**1.757**	0	1	1	0	0	0	0	0	1	0	0	1	0	0	0	0	0	0	0	1	0	0	0	1	1	29
**1.758**	0	0	0	1	0	0	0	0	0	0	0	1	0	0	0	0	0	0	0	1	0	1	0	0	1	29
**1.759**	0	0	0	0	1	0	0	0	0	1	0	1	0	0	0	0	0	0	0	1	0	1	0	0	1	29
**1.760**	0	0	0	1	0	0	0	0	0	0	0	1	0	0	0	0	0	0	0	0	0	1	0	1	1	29
**1.761**	0	0	0	0	0	0	1	0	0	0	0	1	0	0	0	0	0	0	0	1	0	0	1	1	1	29
**1.762**	0	1	0	1	1	0	1	0	0	0	0	1	0	0	0	0	0	0	0	1	0	0	0	0	1	29
**1.763**	0	1	1	1	0	0	0	0	0	1	0	1	0	0	0	0	0	0	0	1	0	0	0	0	0	29
**1.764**	0	1	0	1	1	0	0	0	0	1	0	1	0	1	0	0	0	0	0	0	0	1	0	1	1	29
**1.765**	0	0	0	1	1	0	0	0	1	1	0	1	0	0	0	0	0	0	0	0	0	0	1	0	0	29
**1.766**	0	1	1	1	1	0	0	0	0	1	0	1	0	0	0	0	0	0	0	0	0	0	0	0	0	29
**1.767**	0	1	1	1	0	0	0	0	0	0	0	1	0	0	0	0	0	0	0	1	0	0	0	0	0	29
**1.768**	0	0	0	0	0	0	0	0	1	0	0	1	0	1	0	0	0	0	0	0	0	0	0	0	0	29
**1.769**	0	1	1	0	1	0	0	0	1	0	0	1	0	0	0	0	0	0	0	0	0	0	0	1	0	29
**1.770**	0	0	0	0	0	0	1	0	1	0	0	1	0	0	0	0	0	0	0	0	0	0	0	0	1	29
**1.771**	0	0	1	0	0	0	1	0	0	1	0	0	0	1	0	0	0	0	0	0	0	0	0	0	0	29
**1.772**	0	0	0	1	1	0	0	0	0	0	0	1	0	0	0	0	0	0	0	1	0	1	0	0	1	29
**1.773**	0	0	0	0	0	0	1	0	0	0	0	0	0	1	0	1	0	0	0	0	0	1	0	0	0	29
**1.774**	0	1	0	1	1	0	0	0	0	0	0	1	0	0	0	0	0	0	0	0	0	0	1	1	1	29
**1.775**	0	1	0	0	0	0	0	0	0	0	0	1	0	1	0	0	0	0	0	0	0	0	0	1	0	29
**1.776**	0	0	1	0	0	0	0	0	0	1	0	1	0	1	0	0	0	0	0	1	0	0	0	0	1	29
**1.777**	0	0	0	0	0	0	1	0	1	1	0	0	0	1	0	0	0	0	0	0	0	0	0	0	0	29
**1.778**	0	1	0	0	1	0	0	0	1	1	0	1	1	0	0	0	0	0	0	0	0	0	0	0	1	29
**1.779**	0	0	1	1	0	0	1	0	0	1	0	0	0	1	0	0	0	0	0	1	0	0	0	1	1	29
**1.780**	0	0	0	0	0	0	1	0	0	1	0	1	0	1	0	0	0	0	0	0	0	1	1	0	1	29
**1.781**	0	1	0	0	0	0	0	0	0	0	0	0	1	0	0	0	0	0	0	1	0	1	0	0	0	29
**1.782**	0	0	0	0	1	0	1	0	0	1	0	1	0	1	0	0	0	0	0	0	0	1	1	0	1	29
**1.783**	0	1	0	1	1	0	0	0	1	0	0	1	1	0	0	0	0	0	0	1	0	1	0	0	1	29
**1.784**	0	1	0	1	1	0	0	0	1	0	0	1	0	0	0	0	0	0	0	1	0	0	1	0	1	29
**1.785**	0	1	0	1	0	0	0	0	0	0	0	1	1	0	0	0	0	0	0	1	0	1	0	0	0	29
**1.786**	0	1	0	1	0	0	0	0	0	0	0	1	1	0	0	0	0	0	0	1	0	0	0	0	1	29
**1.787**	0	0	0	0	1	0	0	0	0	0	0	0	0	1	0	0	0	0	0	0	0	0	0	0	0	29
**1.788**	0	0	0	0	0	0	1	0	0	0	0	1	0	0	0	0	0	0	0	0	0	1	0	1	0	29
**1.789**	0	1	0	0	0	0	0	0	0	1	0	1	1	0	0	0	0	0	0	1	0	0	1	0	0	29
**1.790**	0	0	0	0	1	0	0	0	0	0	0	1	0	0	0	0	0	0	0	1	0	1	1	0	1	28
**1.791**	0	0	0	0	0	0	1	0	0	0	0	0	1	0	0	1	0	0	0	0	0	1	0	1	0	28
**1.792**	0	0	0	0	1	0	0	0	0	1	0	1	1	0	0	0	0	0	0	1	0	0	1	0	0	28
**1.793**	0	1	1	0	0	0	0	0	1	1	0	1	0	0	0	0	0	0	0	1	0	0	0	1	1	28
**1.794**	0	1	0	0	1	0	0	0	1	1	0	1	1	0	0	0	0	0	0	1	0	0	0	0	1	28
**1.795**	0	1	0	0	1	0	0	0	0	0	0	0	0	0	0	0	0	0	0	1	0	0	0	1	1	28
**1.796**	0	1	0	0	0	0	1	0	0	0	0	1	0	0	0	0	0	0	0	1	0	0	0	1	1	28
**1.797**	0	0	1	0	0	0	0	0	1	1	0	1	0	1	0	0	0	0	0	1	0	1	0	1	0	28
**1.798**	0	1	0	0	1	0	0	0	1	0	0	1	1	0	0	0	0	0	0	1	0	0	0	1	0	28
**1.799**	0	0	0	0	0	0	1	0	1	1	0	0	0	1	0	0	0	0	0	1	0	1	1	0	0	28
**1.800**	0	0	0	1	1	0	0	0	0	0	0	0	0	0	0	0	0	0	0	1	0	0	0	0	0	28
**1.801**	0	0	0	0	0	0	1	0	1	1	0	1	0	1	0	0	0	0	0	1	0	0	0	0	1	28
**1.802**	0	1	0	1	0	0	0	0	0	1	0	0	0	0	0	0	0	0	0	0	0	0	0	0	0	28
**1.803**	0	0	1	0	1	0	0	0	0	0	0	1	0	0	0	0	0	0	0	0	0	0	0	1	1	28
**1.804**	0	0	1	0	0	0	0	0	1	0	0	0	0	0	0	0	0	0	0	1	0	1	0	0	1	28
**1.805**	0	1	0	1	1	0	0	0	0	1	0	1	0	0	0	0	0	0	0	1	0	0	1	0	1	28
**1.806**	0	0	1	0	0	0	1	0	0	1	0	0	0	1	0	0	0	0	0	0	0	0	0	1	0	28
**1.807**	0	0	1	0	0	0	1	0	1	1	0	1	0	1	0	1	0	0	0	1	0	0	0	1	1	28
**1.808**	0	0	0	0	0	0	1	0	0	0	0	0	0	1	0	0	0	0	0	0	0	0	1	0	1	28
**1.809**	0	1	1	1	1	0	0	0	0	0	0	1	0	0	0	0	0	0	0	1	0	0	0	0	0	28
**1.810**	0	1	0	0	0	0	0	0	1	1	0	1	0	1	0	0	0	0	0	0	0	0	0	0	0	28
**1.811**	0	0	1	0	0	0	0	0	0	1	0	1	0	0	0	0	0	0	0	0	0	1	0	0	1	28
**1.812**	0	0	0	0	0	0	1	0	0	0	0	0	1	0	0	1	0	0	0	0	0	1	0	0	0	28
**1.813**	0	0	0	0	0	0	0	0	1	1	0	0	0	0	0	0	0	0	0	1	0	1	0	0	0	28
**1.814**	0	0	1	0	0	0	1	0	0	1	0	0	1	1	0	0	0	0	0	1	0	0	0	1	1	28
**1.815**	0	0	0	0	0	0	0	0	1	0	0	0	0	0	0	1	0	0	0	0	0	0	0	1	1	28
**1.816**	0	0	0	0	0	0	0	0	0	0	1	0	0	1	0	0	0	0	0	0	0	0	0	0	1	28
**1.817**	0	1	0	1	1	0	0	0	0	0	0	1	1	0	0	0	0	0	0	0	0	1	0	1	1	28
**1.818**	0	0	1	0	0	0	0	0	0	1	1	1	0	1	0	0	0	0	0	0	0	0	0	1	1	28
**1.819**	0	1	0	1	0	0	0	0	0	0	0	0	0	1	0	0	0	0	0	0	0	0	0	0	0	28
**1.820**	0	0	0	0	1	0	1	0	0	1	0	1	0	0	0	0	0	0	0	1	0	0	0	0	0	28
**1.821**	0	0	0	0	0	0	0	0	0	1	0	0	0	0	0	0	0	0	0	0	0	0	1	0	1	28
**1.822**	0	0	0	0	1	0	0	0	1	1	0	1	0	1	0	0	0	0	0	0	0	0	0	0	0	27
**1.823**	0	0	1	0	0	0	0	0	1	0	0	1	0	0	0	0	0	0	0	1	0	1	0	1	0	27
**1.824**	0	1	0	1	1	0	1	0	0	1	0	1	0	0	0	0	0	0	0	0	0	0	0	1	1	27
**1.825**	0	1	0	1	1	0	0	0	0	0	0	1	1	0	0	0	0	0	0	1	0	1	0	1	1	27
**1.826**	0	1	0	0	0	0	0	0	0	0	0	0	0	1	0	0	0	0	0	1	0	0	0	0	0	27
**1.827**	0	0	1	0	0	0	0	0	0	0	0	0	1	0	0	0	0	0	0	1	0	0	0	1	1	27
**1.828**	0	0	0	0	1	0	0	0	0	1	0	1	0	1	0	0	0	0	0	1	0	1	0	1	1	27
**1.829**	0	1	0	0	0	0	0	0	0	0	0	1	0	1	0	0	0	0	0	1	0	1	0	1	1	27

CMP	CD2	CD3	CD4	CD7	CD8	CD10	CD13	CD18	CD26	CD29	CD36	CD44	CD45RA	CD49f	CD54	CD56	CD57	CD58	CD62L	CD71	CD80	Cytokeratin	Histone_H1	HLA-DQ	HLA-DR	Freq. (voxel)
**1.830**	0	1	1	0	1	0	0	0	1	1	0	1	0	0	0	0	0	0	0	0	0	0	0	0	0	27
**1.831**	0	0	1	1	0	0	1	0	0	1	0	1	0	0	0	0	0	0	0	0	0	0	0	1	1	27
**1.832**	0	1	1	0	1	0	0	0	1	0	0	1	0	0	0	0	0	0	0	0	0	0	0	0	0	27
**1.833**	0	1	0	0	1	0	0	0	0	0	0	1	0	0	0	0	0	0	0	0	0	1	1	1	0	27
**1.834**	0	1	0	0	0	0	0	0	0	1	0	1	1	1	0	1	0	0	0	0	0	0	0	0	0	27
**1.835**	0	0	1	0	0	0	0	0	0	1	1	1	0	0	0	0	0	0	0	1	0	0	0	1	1	27
**1.836**	0	0	1	0	0	0	1	0	1	0	0	0	0	1	0	0	0	0	0	0	0	1	0	0	0	27
**1.837**	0	1	0	0	1	0	0	0	0	0	0	0	1	0	0	0	0	0	0	0	0	0	1	0	1	27
**1.838**	0	0	1	0	0	0	1	0	0	1	0	1	0	1	0	0	0	0	0	1	0	1	1	0	1	27
**1.839**	0	1	0	1	1	0	0	0	1	0	0	1	1	0	0	0	0	0	0	1	0	0	0	1	0	27
**1.840**	0	1	0	0	1	0	0	0	1	1	0	1	0	0	0	0	0	0	0	1	0	0	0	1	0	27
**1.841**	0	1	0	0	1	0	0	0	0	1	1	1	0	1	0	0	0	0	0	0	0	0	1	1	1	27
**1.842**	0	1	1	0	1	0	0	0	0	0	0	0	0	0	0	0	0	0	0	0	0	0	0	0	0	27
**1.843**	0	1	0	0	1	0	0	0	1	0	0	1	0	0	0	0	0	0	0	1	0	0	1	0	1	27
**1.844**	0	1	0	0	0	0	0	0	0	1	0	0	0	1	0	0	0	0	0	0	0	0	1	0	0	27
**1.845**	0	0	1	0	0	0	0	0	0	1	0	0	0	1	0	0	0	0	0	1	0	0	0	0	0	27
**1.846**	0	0	1	0	0	0	0	0	0	0	0	1	0	1	0	0	0	0	0	1	0	1	1	0	0	27
**1.847**	0	0	0	1	0	0	0	0	0	0	0	1	0	0	0	0	0	0	0	1	0	0	0	0	1	27
**1.848**	0	0	0	1	0	0	0	0	0	0	0	0	0	1	0	0	0	0	0	0	0	1	0	0	0	27
**1.849**	0	0	0	0	0	0	0	0	1	0	1	1	0	0	0	0	0	0	0	1	0	1	0	0	0	27
**1.850**	0	1	0	1	0	0	0	0	1	1	0	1	1	0	0	0	0	0	0	1	0	0	0	0	0	27
**1.851**	0	0	0	0	0	0	0	0	0	0	0	1	0	0	0	1	0	0	0	1	0	1	0	0	0	27
**1.852**	0	0	0	1	1	0	0	0	0	0	0	1	0	1	0	0	0	0	0	0	0	0	0	0	0	27
**1.853**	0	0	0	0	1	0	0	0	0	0	0	0	0	0	0	0	0	0	0	0	0	0	1	1	1	27
**1.854**	0	0	0	0	0	0	0	0	1	0	0	0	0	1	0	0	0	0	0	0	0	0	0	1	1	27
**1.855**	0	0	0	0	0	0	0	0	0	0	0	1	1	0	0	0	0	0	0	0	0	0	0	1	0	27
**1.856**	0	0	0	0	0	0	0	0	0	1	0	0	1	0	0	1	0	0	0	1	0	0	0	0	0	27
**1.857**	0	0	0	0	0	0	0	0	1	0	0	0	0	0	0	0	0	0	0	0	0	1	1	1	0	27
**1.858**	0	0	0	0	1	0	1	0	1	0	0	0	1	0	0	0	0	0	0	1	0	0	0	0	0	27
**1.859**	0	0	0	0	0	0	0	0	0	1	0	1	1	0	0	0	0	0	0	1	0	0	0	0	0	27
**1.860**	0	0	0	0	1	0	1	0	1	1	0	1	0	0	0	0	0	0	0	1	0	1	0	1	1	26
**1.861**	0	1	0	0	1	0	0	0	1	1	0	1	0	0	0	0	0	0	0	1	0	0	1	1	1	26
**1.862**	0	1	1	0	0	0	0	0	0	1	0	1	0	0	0	0	0	0	0	1	0	0	0	1	1	26
**1.863**	0	1	0	1	0	0	0	0	0	0	0	1	0	0	0	0	0	0	0	1	0	1	0	1	1	26
**1.864**	0	1	0	0	1	0	0	0	0	1	0	1	1	1	0	0	0	0	0	0	0	0	1	0	0	26
**1.865**	0	0	1	0	1	0	0	0	0	1	0	1	0	0	0	0	0	0	0	0	0	0	0	1	1	26
**1.866**	0	0	0	1	0	0	0	0	0	0	0	0	0	1	0	0	0	0	0	0	0	1	0	1	1	26
**1.867**	0	1	1	0	0	0	0	0	0	1	0	1	0	0	0	0	0	0	0	0	0	0	0	1	1	26
**1.868**	0	1	0	0	0	0	0	0	0	0	0	1	0	0	0	0	0	0	0	1	0	0	1	1	1	26
**1.869**	0	0	1	0	0	0	0	0	0	0	0	0	1	1	0	0	0	0	0	0	0	0	0	1	0	26
**1.870**	0	0	1	0	0	0	0	0	1	0	1	0	0	1	0	0	0	0	0	0	0	1	0	0	0	26
**1.871**	0	1	0	1	1	0	0	0	0	0	0	0	0	0	0	0	0	0	0	0	0	0	0	0	1	26
**1.872**	0	0	1	0	0	0	1	0	1	0	0	0	0	0	0	0	0	0	0	0	0	0	0	1	1	26
**1.873**	0	0	1	0	0	0	0	0	1	0	0	0	0	0	0	0	0	0	0	0	0	0	1	0	0	26
**1.874**	0	0	0	0	0	0	1	0	1	1	0	1	0	1	0	0	0	0	0	0	0	0	1	1	1	26
**1.875**	0	0	0	0	0	0	1	0	0	0	1	0	1	1	0	0	0	0	0	0	0	1	0	0	0	26
**1.876**	0	0	0	0	1	0	0	0	0	0	0	1	0	0	0	1	0	0	0	0	0	0	0	1	1	26
**1.877**	0	0	0	0	1	0	1	0	0	1	0	1	0	1	0	0	0	0	0	0	0	1	0	0	1	26
**1.878**	0	0	0	0	0	0	1	0	0	1	0	0	0	0	0	0	0	0	0	0	0	1	1	0	0	26
**1.879**	0	0	0	0	0	0	0	0	0	1	0	1	0	1	0	0	0	0	0	1	0	0	1	1	0	26
**1.880**	0	0	0	0	0	0	1	0	1	0	0	0	0	1	0	0	0	0	0	1	0	1	1	0	0	26
**1.881**	0	0	0	0	0	0	0	0	0	0	0	0	1	0	0	0	0	0	0	0	0	0	0	0	1	26
**1.882**	0	0	0	0	1	0	1	0	0	0	0	1	0	0	0	0	0	0	0	0	0	0	0	0	1	26
**1.883**	0	0	0	0	0	0	0	0	0	0	0	0	0	0	0	1	0	0	0	1	0	0	0	0	0	26
**1.884**	0	0	0	0	1	0	1	0	0	0	0	1	1	0	0	0	0	0	0	0	0	0	0	0	0	26
**1.885**	0	0	0	0	1	0	1	0	0	0	0	1	0	0	0	0	0	0	0	0	0	0	0	1	0	26
**1.886**	0	0	0	0	1	0	1	0	1	1	0	0	1	0	0	1	0	0	0	1	0	0	0	0	0	26
**1.887**	0	0	0	0	1	0	0	0	1	0	0	0	1	0	0	1	0	0	0	1	0	0	0	0	0	26
**1.888**	0	0	1	0	0	0	1	0	1	0	0	1	0	0	0	0	0	0	0	0	0	1	0	1	1	25
**1.889**	0	1	0	0	1	0	1	0	1	1	0	1	0	0	0	0	0	0	0	0	0	0	0	1	1	25
**1.890**	0	0	0	0	1	0	0	0	0	1	0	0	0	0	0	0	0	0	0	1	0	0	1	0	0	25
**1.891**	0	0	0	0	0	0	1	0	1	1	0	1	0	0	0	0	0	0	0	1	0	0	1	1	1	25
**1.892**	0	0	0	0	0	0	0	0	1	0	0	0	1	0	0	0	0	0	0	1	0	0	0	1	1	25
**1.893**	0	0	1	0	1	0	0	0	0	1	0	1	0	0	0	0	0	0	0	1	0	0	0	1	1	25
**1.894**	0	1	1	0	1	0	0	0	0	0	0	1	0	0	0	0	0	0	0	0	0	0	0	1	1	25
**1.895**	0	0	1	0	0	0	0	0	0	1	0	0	0	0	0	0	0	0	0	0	0	1	0	1	1	25
**1.896**	0	1	0	0	1	0	0	0	0	1	0	1	1	0	0	0	0	0	0	0	0	0	1	1	1	25
**1.897**	0	1	0	0	0	0	1	0	0	0	0	1	0	0	0	0	0	0	0	1	0	0	0	0	1	25
**1.898**	0	1	0	0	1	0	0	0	1	1	0	1	0	0	0	0	0	0	0	0	0	0	1	1	0	25
**1.899**	0	0	1	0	1	0	0	0	0	0	0	1	0	0	0	0	0	0	0	1	0	0	0	1	1	25
**1.900**	0	0	1	0	0	0	1	0	0	0	0	0	0	0	0	0	0	0	0	0	0	1	1	0	0	25
**1.901**	0	1	1	0	0	0	0	0	1	1	0	1	0	0	0	0	0	0	0	0	0	0	0	1	1	25
**1.902**	0	1	0	1	0	0	0	0	0	1	0	0	0	0	0	0	0	0	0	1	0	0	1	0	0	25
**1.903**	0	1	0	0	0	0	0	0	0	0	0	0	0	1	0	0	0	0	0	0	0	0	0	1	0	25
**1.904**	0	0	1	0	0	0	0	0	1	1	0	1	0	0	0	0	0	0	0	1	0	0	0	0	0	25
**1.905**	0	1	0	0	0	0	0	0	0	1	0	1	0	1	0	0	0	0	0	0	0	1	0	0	1	25
**1.906**	0	0	0	1	1	0	0	0	1	0	0	1	0	0	0	0	0	0	0	0	0	1	0	0	0	25
**1.907**	0	0	0	1	0	0	1	0	0	1	0	1	0	1	0	0	0	0	0	1	0	0	0	0	0	25
**1.908**	0	0	0	0	0	0	1	0	0	1	0	1	0	1	0	0	0	0	0	1	0	0	1	0	1	25
**1.909**	0	1	1	0	1	0	0	0	1	1	0	1	0	0	0	0	0	0	0	1	0	0	0	0	0	25
**1.910**	0	1	0	0	1	0	0	0	0	1	0	1	0	1	0	0	0	0	0	0	0	1	0	1	1	25
**1.911**	0	0	1	0	0	0	1	0	0	1	0	1	1	1	0	0	0	0	0	0	0	0	0	1	1	25
**1.912**	0	0	0	0	0	0	1	0	0	1	1	0	0	1	0	0	0	0	0	1	0	1	0	0	0	25
**1.913**	0	0	0	0	0	0	1	0	0	1	0	0	0	0	0	0	0	0	0	1	0	0	1	0	0	25
**1.914**	0	1	0	0	0	0	0	0	0	0	0	1	0	1	0	0	0	0	0	1	0	0	0	0	0	25
**1.915**	0	0	0	0	0	0	1	0	1	1	0	0	0	1	0	0	0	0	0	0	0	0	0	1	1	25
**1.916**	0	0	0	0	0	0	0	0	1	1	0	0	0	0	0	0	0	0	0	0	0	0	1	1	0	25
**1.917**	0	0	0	0	0	0	0	0	0	0	0	0	1	0	0	1	0	0	0	0	0	0	0	1	1	25
**1.918**	0	0	1	0	0	0	1	0	1	1	0	1	1	1	0	0	0	0	0	1	0	0	0	1	1	25
**1.919**	0	0	0	0	0	0	1	0	0	1	1	1	0	0	0	0	0	0	0	0	0	0	0	1	1	25
**1.920**	0	0	0	0	0	0	1	0	0	0	0	0	1	0	0	1	0	0	0	0	0	0	1	0	0	25
**1.921**	0	1	0	1	0	0	0	0	0	0	0	1	0	1	0	0	0	0	0	0	0	0	1	0	0	25
**1.922**	0	0	1	0	0	0	0	0	1	0	0	0	0	0	0	0	0	0	0	1	0	0	0	0	0	25
**1.923**	0	0	1	0	0	0	0	0	0	0	1	1	0	0	0	0	0	0	0	0	0	0	0	1	1	25
**1.924**	0	0	0	0	0	0	1	0	0	1	0	0	0	0	0	0	0	0	0	0	0	1	0	0	1	25
**1.925**	0	0	0	0	0	0	0	0	0	1	0	0	0	1	0	0	0	0	0	0	0	0	1	1	0	25
**1.926**	0	0	0	0	0	0	0	0	0	0	0	1	1	0	0	0	0	0	0	0	0	1	0	1	1	25
**1.927**	0	0	0	0	0	0	1	0	0	0	1	1	0	0	0	0	0	0	0	0	0	0	0	0	0	25
**1.928**	0	0	0	0	0	0	1	0	0	0	1	0	0	0	0	0	0	0	0	0	0	0	0	0	0	25
**1.929**	0	0	0	0	0	0	0	0	0	0	1	0	0	0	0	0	0	0	0	1	0	0	0	1	0	25
**1.930**	0	1	0	0	0	0	0	0	0	1	0	1	1	0	0	0	0	0	0	0	0	1	0	0	0	25
**1.931**	0	0	0	0	0	0	0	0	0	0	1	0	1	0	0	0	0	0	0	0	0	1	0	0	0	25
**1.932**	0	0	0	1	1	0	1	0	0	0	0	1	0	0	0	0	0	0	0	1	0	0	0	1	1	24
**1.933**	0	0	0	1	1	0	0	0	1	1	0	1	0	0	0	0	0	0	0	1	0	0	0	1	1	24
**1.934**	0	0	0	1	0	0	0	0	0	1	0	1	0	0	0	0	0	0	0	1	0	0	0	0	1	24
**1.935**	0	1	1	0	0	0	0	0	1	0	0	1	0	0	0	0	0	0	0	0	0	0	0	0	0	24
**1.936**	0	0	0	0	1	0	0	0	0	1	0	1	1	0	0	0	0	0	0	0	0	0	0	0	1	24
**1.937**	0	1	1	1	1	0	0	0	1	0	0	1	1	0	0	0	0	0	0	1	0	0	0	0	1	24
**1.938**	0	1	0	1	0	0	0	0	0	0	0	1	0	0	0	0	0	0	0	0	0	0	1	1	1	24
**1.939**	0	1	0	1	1	0	0	0	0	0	0	1	0	0	0	0	0	0	0	1	0	1	1	0	1	24
**1.940**	0	0	0	0	1	0	1	0	0	1	0	1	0	1	0	0	0	0	0	1	0	1	1	0	1	24
**1.941**	0	0	0	0	1	0	0	0	1	0	0	0	0	0	0	0	0	0	0	1	0	1	0	0	1	24
**1.942**	0	0	0	0	0	0	1	0	0	1	0	1	1	1	0	0	0	0	0	0	0	0	0	1	1	24
**1.943**	0	1	1	0	1	0	0	0	1	0	0	1	0	0	0	0	0	0	0	1	0	0	0	0	0	24
**1.944**	0	0	1	0	0	0	0	0	0	1	0	1	0	1	0	0	0	0	0	1	0	1	1	1	1	24
**1.945**	0	0	0	0	1	0	0	0	0	1	1	1	0	1	0	0	0	0	0	0	0	1	0	0	0	24
**1.946**	0	0	0	0	0	0	1	0	1	0	0	1	0	1	0	0	0	0	0	1	0	1	1	0	0	24
**1.947**	0	0	0	0	0	0	0	0	0	0	0	0	0	1	0	1	0	0	0	0	0	0	0	0	0	24
**1.948**	0	0	1	0	0	0	0	0	1	1	0	0	0	0	0	0	0	0	0	0	0	0	0	1	0	24
**1.949**	0	1	0	0	1	0	0	0	0	1	1	1	0	0	0	0	0	0	0	0	0	0	0	0	1	24
**1.950**	0	1	0	0	0	0	0	0	1	1	0	1	0	0	0	0	0	0	0	1	0	0	0	1	0	24
**1.951**	0	0	0	1	0	0	1	0	0	0	0	0	0	1	0	0	0	0	0	0	0	1	0	0	0	24

CMP	CD2	CD3	CD4	CD7	CD8	CD10	CD13	CD18	CD26	CD29	CD36	CD44	CD45RA	CD49f	CD54	CD56	CD57	CD58	CD62L	CD71	CD80	Cytokeratin	Histone_H1	HLA-DQ	HLA-DR	Freq. (voxel)
**1.952**	0	0	0	1	0	0	0	0	0	0	0	0	0	0	0	0	0	0	0	0	0	0	1	0	1	24
**1.953**	0	0	0	0	0	0	1	0	1	0	0	1	0	1	0	0	0	0	0	1	0	0	0	1	1	24
**1.954**	0	1	0	0	0	0	0	0	0	1	0	1	1	0	0	0	0	0	0	1	0	0	0	0	0	24
**1.955**	0	0	1	0	0	0	0	0	0	0	0	0	0	1	0	0	0	0	0	1	0	0	0	0	0	24
**1.956**	0	0	0	0	0	0	1	0	1	0	0	0	1	0	0	0	0	0	0	1	0	0	0	1	0	24
**1.957**	0	1	0	1	1	0	1	0	0	1	0	1	0	0	0	0	0	0	0	0	0	0	0	0	0	24
**1.958**	0	0	1	0	0	0	1	0	0	1	0	1	0	0	0	0	0	0	0	0	0	1	0	0	0	24
**1.959**	0	1	0	0	0	0	0	0	0	0	0	0	0	0	0	0	0	0	0	0	0	1	0	1	0	24
**1.960**	0	1	0	0	1	0	1	0	0	1	0	1	0	0	0	0	0	0	0	1	0	0	1	0	0	24
**1.961**	0	0	0	0	1	0	1	0	0	0	1	1	0	0	0	0	0	0	0	0	0	0	0	0	0	24
**1.962**	0	1	0	1	0	0	0	0	0	1	0	1	1	0	0	0	0	0	0	0	0	0	1	0	0	23
**1.963**	0	1	0	0	1	0	1	0	1	1	0	1	0	0	0	0	0	0	0	1	0	0	0	1	1	23
**1.964**	0	1	0	0	1	0	0	0	1	0	0	1	0	1	0	0	0	0	0	1	0	1	0	1	1	23
**1.965**	0	0	0	0	1	0	0	0	1	1	0	1	0	0	0	0	0	0	0	1	0	1	0	1	1	23
**1.966**	0	0	0	0	1	0	0	0	1	0	0	1	0	0	0	0	0	0	0	1	0	1	1	0	1	23
**1.967**	0	0	0	0	1	0	0	0	0	1	0	1	1	0	0	0	0	0	0	1	0	1	0	0	0	23
**1.968**	0	0	0	1	0	0	0	0	1	1	0	1	0	0	0	0	0	0	0	1	0	0	1	0	0	23
**1.969**	0	1	1	1	1	0	0	0	0	1	0	1	0	1	0	0	0	0	0	1	0	0	0	1	1	23
**1.970**	0	1	1	0	1	0	0	0	1	0	0	1	1	0	0	0	0	0	0	1	0	0	0	1	1	23
**1.971**	0	1	1	0	0	0	0	0	1	0	0	1	0	0	0	0	0	0	0	1	0	1	0	0	1	23
**1.972**	0	1	1	0	0	0	0	0	0	0	0	0	0	0	0	0	0	0	0	1	0	0	0	0	0	23
**1.973**	0	1	0	0	1	0	0	0	0	0	0	1	0	0	0	0	0	0	0	1	0	1	0	1	0	23
**1.974**	0	1	0	0	0	0	1	0	1	0	0	1	0	0	0	0	0	0	0	0	0	0	0	1	1	23
**1.975**	0	1	0	1	0	0	0	0	1	1	0	1	0	0	0	0	0	0	0	1	0	0	0	1	1	23
**1.976**	0	0	1	0	0	0	1	0	0	0	0	1	0	0	0	0	0	0	0	0	0	0	0	1	0	23
**1.977**	0	0	1	0	0	0	1	0	0	1	0	0	0	1	0	0	0	0	0	1	0	1	0	0	0	23
**1.978**	0	0	0	0	1	0	0	0	0	1	0	1	0	1	0	0	0	0	0	0	0	1	0	0	1	23
**1.979**	0	0	0	0	0	0	1	0	1	0	0	1	0	1	0	0	0	0	0	0	0	0	1	1	1	23
**1.980**	0	0	0	0	0	0	0	0	1	1	0	1	0	1	0	0	0	0	0	1	0	0	0	0	1	23
**1.981**	0	1	0	0	0	0	0	0	0	1	0	1	0	1	0	0	0	0	0	0	0	1	0	1	1	23
**1.982**	0	0	1	1	0	0	1	0	1	1	1	1	1	1	0	1	0	0	0	1	0	0	0	1	1	23
**1.983**	0	0	1	0	1	0	0	0	0	0	0	0	0	0	0	0	0	0	0	0	0	0	0	1	1	23
**1.984**	0	0	1	0	0	0	0	0	0	1	0	1	0	1	0	0	0	0	0	1	0	0	0	0	0	23
**1.985**	0	0	0	1	0	0	1	0	0	1	0	1	0	1	0	0	0	0	0	1	0	1	0	0	1	23
**1.986**	0	0	0	0	0	0	1	0	1	0	0	1	0	0	0	0	0	0	0	0	0	1	0	0	0	23
**1.987**	0	0	1	0	0	0	0	0	1	0	1	0	0	0	0	0	0	0	0	0	0	1	0	0	0	23
**1.988**	0	0	0	0	0	0	0	0	0	1	0	0	0	1	0	0	0	0	0	1	0	1	0	0	1	23
**1.989**	0	0	0	0	0	0	0	0	0	0	0	0	0	1	0	0	0	0	0	0	0	1	1	0	1	23
**1.990**	0	0	1	0	0	0	1	0	0	1	0	0	0	1	0	0	0	0	0	1	0	0	0	1	1	23
**1.991**	0	0	0	0	0	0	1	0	0	1	0	1	0	0	0	0	0	0	0	1	0	1	0	0	1	23
**1.992**	0	0	0	0	0	0	0	0	0	1	0	0	0	1	0	0	0	0	0	1	0	0	1	0	1	23
**1.993**	0	0	0	0	0	0	1	0	0	0	1	1	0	0	0	0	0	0	0	0	0	0	0	1	1	23
**1.994**	0	0	0	1	0	0	0	0	0	0	0	0	0	0	0	0	0	0	0	1	0	0	0	1	0	23
**1.995**	0	0	0	0	0	0	0	0	1	0	0	0	0	0	0	0	0	0	0	1	0	0	1	1	0	23
**1.996**	0	0	1	0	0	0	1	0	0	0	0	0	1	0	0	0	0	0	0	1	0	1	0	1	0	22
**1.997**	0	0	0	1	1	0	1	0	0	0	0	1	0	0	0	0	0	0	0	0	0	0	0	1	1	22
**1.998**	0	0	0	1	0	0	1	0	0	1	0	1	0	0	0	0	0	0	0	0	0	0	0	1	1	22
**1.999**	0	1	0	1	1	0	1	0	0	0	0	1	1	0	0	0	0	0	0	1	0	0	0	1	1	22
**2.000**	0	0	1	0	0	0	0	0	0	0	0	0	0	0	0	0	0	0	0	0	0	1	1	1	1	22
**2.001**	0	0	0	1	0	0	0	0	0	1	0	1	0	1	0	0	0	0	0	1	0	0	0	0	0	22
**2.002**	0	1	0	1	1	0	0	0	0	1	0	1	0	0	0	0	0	0	0	1	0	1	0	0	1	22
**2.003**	0	0	1	1	0	0	1	0	0	1	0	1	0	0	0	0	0	0	0	1	0	0	0	1	1	22
**2.004**	0	0	0	1	0	0	0	0	0	0	0	0	0	1	0	0	0	0	0	1	0	0	0	0	0	22
**2.005**	0	0	0	0	1	0	1	0	0	1	0	1	0	1	0	0	0	0	0	1	0	0	0	0	0	22
**2.006**	0	0	0	0	0	0	1	0	0	0	0	0	1	0	0	0	0	0	0	0	0	1	0	1	0	22
**2.007**	0	1	1	0	0	0	0	0	1	0	0	1	0	0	0	0	0	0	0	0	0	0	0	1	1	22
**2.008**	0	1	0	1	0	0	0	0	0	0	0	0	0	0	0	0	0	0	0	1	0	1	1	0	1	22
**2.009**	0	0	1	0	0	0	1	0	0	0	0	1	0	0	0	1	0	0	0	0	0	0	0	1	1	22
**2.010**	0	0	0	1	0	0	0	0	0	1	0	1	0	0	0	0	0	0	0	1	0	1	0	0	0	22
**2.011**	0	0	0	0	1	0	0	0	0	1	0	1	1	1	0	0	0	0	0	0	0	0	0	0	0	22
**2.012**	0	1	0	1	0	0	0	0	0	1	0	1	0	1	0	0	0	0	0	1	0	0	0	0	0	22
**2.013**	0	1	0	0	1	0	1	0	0	1	0	1	0	1	0	0	0	0	0	1	0	1	0	0	0	22
**2.014**	0	1	0	0	1	0	0	0	0	0	0	1	0	1	0	0	0	0	0	0	0	1	0	1	1	22
**2.015**	0	1	0	1	1	0	0	0	0	1	0	0	0	0	0	0	0	0	0	0	0	0	0	0	0	22
**2.016**	0	0	1	0	0	0	0	0	0	0	0	1	0	1	0	0	0	0	0	1	0	1	0	1	1	22
**2.017**	0	1	0	1	1	0	0	0	1	0	0	1	1	0	0	0	0	0	0	0	0	0	0	1	0	22
**2.018**	0	0	1	0	1	0	0	0	1	0	0	1	0	0	0	0	0	0	0	0	0	0	0	1	1	22
**2.019**	0	0	1	0	0	0	0	0	0	0	0	0	1	0	0	0	0	0	0	0	0	0	0	1	1	22
**2.020**	0	0	0	0	0	0	0	0	1	0	1	0	0	0	0	1	0	0	0	0	0	1	0	0	0	22
**2.021**	0	0	0	0	0	0	0	0	0	1	1	1	0	1	0	0	0	0	0	0	0	1	0	0	1	22
**2.022**	0	0	0	0	0	0	0	0	0	0	0	0	0	1	0	0	0	0	0	1	0	0	1	1	1	22
**2.023**	0	1	0	1	0	0	0	0	0	0	0	1	0	0	0	0	0	0	0	1	0	1	1	0	1	22
**2.024**	0	1	0	0	1	0	0	0	0	0	0	0	1	0	0	0	0	0	0	0	0	1	0	0	1	22
**2.025**	0	0	1	0	0	0	0	0	0	1	0	1	0	0	0	0	0	0	0	1	0	1	0	1	0	22
**2.026**	0	0	1	0	0	0	0	0	0	0	0	1	0	1	0	0	0	0	0	0	0	0	0	1	0	22
**2.027**	0	0	0	0	0	0	1	0	0	0	0	0	0	0	0	0	0	0	0	0	0	0	1	0	1	22
**2.028**	0	1	0	1	1	0	0	0	1	0	0	1	0	0	0	0	0	0	0	1	0	1	1	0	1	22
**2.029**	0	1	0	0	1	0	0	0	0	1	0	1	0	0	0	0	0	0	0	1	0	0	1	0	1	22
**2.030**	0	0	1	1	0	0	1	0	1	1	1	1	1	1	0	0	0	0	0	1	0	0	0	1	1	22
**2.031**	0	0	0	0	0	0	1	0	0	0	0	1	0	0	0	0	0	0	0	1	0	1	0	0	1	22
**2.032**	0	0	0	0	0	0	0	0	1	1	0	0	0	0	0	0	0	0	0	1	0	0	0	0	0	22
**2.033**	0	1	0	1	1	0	0	0	0	1	0	0	0	0	0	0	0	0	0	1	0	0	0	0	0	22
**2.034**	0	1	0	1	0	0	0	0	0	0	0	1	0	1	0	0	0	0	0	0	0	0	0	0	1	22
**2.035**	0	0	1	0	0	0	1	0	0	1	1	1	1	1	0	0	0	0	0	1	0	0	0	1	1	22
**2.036**	0	0	0	0	0	0	0	0	1	1	0	0	0	0	0	0	0	0	0	0	0	0	0	1	0	22
**2.037**	0	1	0	1	1	0	0	0	1	0	0	0	0	0	0	0	0	0	0	0	0	0	1	0	0	22
**2.038**	0	0	1	0	0	0	1	0	0	0	0	1	0	1	0	0	0	0	0	0	0	1	0	0	0	22
**2.039**	0	0	0	0	0	0	0	0	0	0	0	0	0	0	0	1	0	0	0	1	0	0	0	1	1	22
**2.040**	0	1	0	1	0	0	0	0	0	0	0	0	0	1	0	0	0	0	0	0	0	0	1	0	0	22
**2.041**	0	0	0	0	0	0	1	0	0	0	0	0	1	0	0	1	0	0	0	1	0	1	0	0	0	22
**2.042**	0	1	0	1	1	0	0	0	0	0	0	1	1	1	0	0	0	0	0	1	0	0	0	0	0	22
**2.043**	0	1	0	1	0	0	0	0	0	1	0	1	1	0	0	0	0	0	0	0	0	0	0	0	0	22
**2.044**	0	1	0	1	0	0	0	0	0	0	0	0	0	0	0	0	0	0	0	0	0	0	0	0	1	22
**2.045**	0	0	1	0	0	0	1	0	0	1	0	1	0	1	0	0	0	0	0	0	0	1	0	1	0	22
**2.046**	0	0	0	0	0	0	1	0	1	0	1	0	0	0	0	0	0	0	0	0	0	1	0	0	0	22
**2.047**	0	0	0	0	0	0	1	0	0	1	0	0	0	0	0	0	0	0	0	1	0	0	0	0	1	22
**2.048**	0	1	0	0	1	0	1	0	0	0	0	1	1	0	0	0	0	0	0	1	0	0	1	0	0	22
**2.049**	0	1	0	0	1	0	1	0	0	1	0	1	0	0	0	0	0	0	0	1	0	0	0	0	0	22
**2.050**	0	1	1	1	1	0	1	0	1	1	0	1	1	0	0	0	0	0	0	1	0	1	0	1	1	21
**2.051**	0	1	1	1	0	0	0	0	1	1	0	1	0	0	0	0	0	0	0	0	0	0	0	0	0	21
**2.052**	0	1	0	0	1	0	0	0	0	1	0	0	0	0	0	0	0	0	0	0	0	1	0	0	0	21
**2.053**	0	1	1	1	1	0	0	0	1	1	0	1	1	0	0	0	0	0	0	1	0	0	0	0	1	21
**2.054**	0	0	0	1	0	0	1	0	0	0	0	1	0	0	0	0	0	0	0	0	0	0	0	0	1	21
**2.055**	0	1	0	0	0	0	0	0	1	0	0	1	0	0	0	0	0	0	0	0	0	1	0	1	0	21
**2.056**	0	0	1	0	0	0	0	0	1	0	0	1	0	0	0	0	0	0	0	0	0	1	0	0	1	21
**2.057**	0	0	0	1	0	0	0	0	0	0	0	0	1	0	0	1	0	0	0	0	0	0	0	0	0	21
**2.058**	0	0	0	0	0	0	1	0	0	1	0	1	0	0	0	0	0	0	0	0	0	0	1	1	0	21
**2.059**	0	0	0	0	0	0	0	0	0	0	0	0	1	1	0	1	0	0	0	0	0	0	0	1	0	21
**2.060**	0	1	0	0	0	0	0	0	1	1	0	1	0	0	0	0	0	0	0	1	0	0	0	0	1	21
**2.061**	0	0	1	0	0	0	1	0	1	1	0	1	0	1	0	0	0	0	0	1	0	1	0	1	0	21
**2.062**	0	0	1	0	0	0	1	0	0	0	1	0	0	1	0	0	0	0	0	0	0	1	0	0	0	21
**2.063**	0	0	1	0	0	0	0	0	0	0	0	1	0	0	0	0	0	0	0	0	0	0	1	1	0	21
**2.064**	0	0	0	0	0	0	1	0	0	0	0	0	0	1	0	0	0	0	0	0	0	1	0	1	0	21
**2.065**	0	1	0	1	0	0	0	0	0	1	0	1	0	1	0	0	0	0	0	1	0	0	0	1	0	21
**2.066**	0	1	0	0	1	0	0	0	1	1	0	1	1	0	0	0	0	0	0	0	0	0	1	1	1	21
**2.067**	0	1	0	0	1	0	0	0	0	0	0	1	1	0	0	0	0	0	0	1	0	1	0	0	1	21
**2.068**	0	1	0	0	1	0	0	0	0	0	0	1	1	0	0	0	0	0	0	0	0	1	1	1	1	21
**2.069**	0	1	0	0	1	0	0	0	1	0	0	1	1	0	0	0	0	0	0	0	0	0	0	1	0	21
**2.070**	0	0	0	0	0	0	0	0	1	0	1	1	0	1	0	0	0	0	0	0	0	1	0	0	0	21
**2.071**	0	1	0	1	1	0	0	0	1	0	0	1	1	0	0	0	0	0	0	0	0	0	1	0	0	21
**2.072**	0	0	1	0	0	0	0	0	0	0	0	1	0	1	0	0	0	0	0	0	0	1	0	1	0	21
**2.073**	0	0	0	0	0	0	1	0	0	1	0	0	0	0	0	0	0	0	0	0	0	0	1	1	1	21

CMP	CD2	CD3	CD4	CD7	CD8	CD10	CD13	CD18	CD26	CD29	CD36	CD44	CD45RA	CD49f	CD54	CD56	CD57	CD58	CD62L	CD71	CD80	Cytokeratin	Histone_H1	HLA-DQ	HLA-DR	Freq. (voxel)
**2.074**	0	1	0	0	1	0	0	0	1	0	0	0	0	0	0	0	0	0	0	0	0	1	0	0	0	21
**2.075**	0	0	0	0	0	0	1	0	0	0	0	0	1	1	0	0	0	0	0	1	0	1	0	0	0	21
**2.076**	0	1	1	0	0	0	0	0	0	0	0	1	0	0	0	0	0	0	0	0	0	0	1	0	0	21
**2.077**	0	1	0	1	1	0	0	0	0	0	0	1	1	0	0	0	0	0	0	1	0	1	1	0	0	21
**2.078**	0	1	0	1	0	0	0	0	0	0	0	0	0	0	0	0	0	0	0	1	0	0	1	0	1	21
**2.079**	0	0	0	0	0	0	0	0	0	1	0	0	0	0	0	1	0	0	0	0	0	0	0	1	1	21
**2.080**	0	0	0	0	0	0	0	0	0	0	1	1	0	0	0	0	0	0	0	0	0	0	1	0	0	21
**2.081**	0	1	0	1	1	0	0	0	0	0	0	1	1	0	0	0	0	0	0	0	0	0	0	1	0	21
**2.082**	0	1	0	0	1	0	0	0	0	0	0	1	1	1	0	0	0	0	0	0	0	0	0	0	0	21
**2.083**	0	0	0	1	0	0	0	0	1	0	0	0	0	0	0	0	0	0	0	1	0	0	0	0	0	21
**2.084**	0	0	0	0	0	0	0	0	0	1	1	0	0	0	0	0	0	0	0	0	0	0	0	0	1	21
**2.085**	0	0	0	0	0	0	0	0	1	0	0	0	1	0	0	0	0	0	0	0	0	0	0	1	0	21
**2.086**	0	1	0	1	0	0	0	0	1	0	0	0	0	0	0	0	0	0	0	1	0	0	1	0	0	21
**2.087**	0	0	0	0	1	0	0	0	1	0	0	0	1	0	0	1	0	0	0	1	0	1	0	0	0	21
**2.088**	0	1	0	1	0	0	0	0	0	0	0	0	0	1	0	0	0	0	0	1	0	0	0	0	0	20
**2.089**	0	1	0	1	1	0	0	0	1	0	0	1	0	1	0	0	0	0	0	1	0	1	0	1	1	20
**2.090**	0	1	0	1	1	0	0	0	0	0	0	0	0	0	0	0	0	0	0	1	0	0	0	1	1	20
**2.091**	0	0	1	0	1	0	0	0	1	1	0	1	0	0	0	0	0	0	0	1	0	0	0	1	1	20
**2.092**	0	1	1	0	1	0	0	0	1	1	0	1	1	0	0	0	0	0	0	1	0	0	0	0	0	20
**2.093**	0	0	0	1	0	0	0	0	0	0	0	0	0	0	0	0	0	0	0	0	0	0	1	1	0	20
**2.094**	0	0	0	0	0	0	0	0	0	1	0	0	0	0	0	0	0	0	0	1	0	1	0	1	1	20
**2.095**	0	0	0	1	1	0	0	0	0	1	0	1	0	0	0	0	0	0	0	1	0	0	1	0	0	20
**2.096**	0	0	0	0	1	0	0	0	1	0	0	0	0	0	0	0	0	0	0	1	0	0	0	0	0	20
**2.097**	0	1	1	0	1	0	0	0	1	0	0	1	0	0	0	0	0	0	0	1	0	0	0	1	1	20
**2.098**	0	1	1	0	1	0	0	0	1	0	0	1	0	0	0	0	0	0	0	0	0	0	0	1	1	20
**2.099**	0	0	1	0	0	0	0	0	0	0	0	1	0	0	0	0	0	0	0	1	0	1	0	1	0	20
**2.100**	0	1	1	0	1	0	0	0	1	1	0	1	1	0	0	0	0	0	0	1	0	0	0	1	0	20
**2.101**	0	1	1	0	1	0	0	0	0	0	0	1	1	0	0	0	0	0	0	0	0	0	0	0	0	20
**2.102**	0	0	1	0	0	0	1	0	0	1	0	1	1	1	0	0	0	0	0	1	0	1	0	1	1	20
**2.103**	0	1	1	0	1	0	0	0	0	0	0	0	0	0	0	0	0	0	0	0	0	0	0	1	1	20
**2.104**	0	1	0	0	0	0	0	0	0	0	0	1	0	0	0	0	0	0	0	1	0	1	0	1	0	20
**2.105**	0	0	0	1	1	0	0	0	1	0	0	1	0	0	0	0	0	0	0	1	0	1	0	0	1	20
**2.106**	0	0	0	0	1	0	0	0	0	1	0	1	0	1	0	0	0	0	0	1	0	1	0	0	1	20
**2.107**	0	1	1	1	1	0	0	0	1	0	0	1	1	0	0	0	0	0	0	1	0	0	0	1	0	20
**2.108**	0	1	0	1	1	0	0	0	0	1	1	1	0	1	0	0	0	0	0	0	0	0	1	1	1	20
**2.109**	0	0	0	1	0	0	0	0	0	1	0	1	0	1	0	0	0	0	0	1	0	1	0	0	0	20
**2.110**	0	0	0	0	0	0	0	0	1	1	0	0	0	0	0	0	0	0	0	0	0	1	0	1	0	20
**2.111**	0	1	0	1	0	0	0	0	0	0	0	1	0	1	0	0	0	0	0	0	0	0	1	0	1	20
**2.112**	0	0	0	0	0	0	1	0	0	1	1	1	0	1	0	0	0	0	0	0	0	1	0	0	0	20
**2.113**	0	0	0	0	0	0	1	0	0	0	0	1	0	1	0	0	0	0	0	0	0	0	1	1	0	20
**2.114**	0	0	1	0	1	0	0	0	0	0	0	0	0	0	0	0	0	0	0	0	0	0	0	0	0	20
**2.115**	0	0	0	1	0	0	0	0	0	0	0	1	0	0	0	0	0	0	0	0	0	0	1	0	1	20
**2.116**	0	0	0	0	1	0	0	0	0	1	0	1	0	1	0	0	0	0	0	0	0	0	0	1	1	20
**2.117**	0	1	0	1	0	0	0	0	1	0	0	1	1	0	0	0	0	0	0	0	0	0	0	0	0	20
**2.118**	0	0	1	0	0	0	1	0	0	1	0	0	0	1	0	0	0	0	0	1	0	1	0	0	1	20
**2.119**	0	0	0	0	1	0	0	0	1	0	0	0	0	0	0	0	0	0	0	0	0	0	0	1	1	20
**2.120**	0	0	0	0	1	0	0	0	0	1	0	1	1	1	0	0	0	0	0	0	0	1	0	0	0	20
**2.121**	0	1	1	1	1	0	0	0	1	0	0	1	0	0	0	0	0	0	0	1	0	0	0	0	0	20
**2.122**	0	1	0	0	0	0	0	0	0	1	0	1	0	0	0	0	0	0	0	0	0	0	1	0	1	20
**2.123**	0	0	0	0	1	0	0	0	0	1	0	1	0	1	0	0	0	0	0	0	0	1	1	0	0	20
**2.124**	0	0	0	0	0	0	1	0	0	1	0	0	0	1	0	0	0	0	0	0	0	0	1	0	1	20
**2.125**	0	0	0	0	0	0	0	0	1	0	0	1	0	1	0	0	0	0	0	0	0	0	0	1	1	20
**2.126**	0	0	0	0	0	0	0	0	1	0	0	0	0	0	0	1	0	0	0	0	0	1	0	0	0	20
**2.127**	0	0	0	0	0	0	0	0	0	1	0	0	0	1	0	0	0	0	0	0	0	0	1	0	1	20
**2.128**	0	0	0	1	0	0	0	0	0	0	0	0	1	0	0	0	0	0	0	1	0	0	0	0	0	20
**2.129**	0	1	0	0	0	0	0	0	0	1	0	0	0	0	0	0	0	0	0	0	0	1	0	0	0	20
**2.130**	0	0	1	0	0	0	0	0	0	0	1	0	0	0	0	0	0	0	0	0	0	0	0	0	0	20
**2.131**	0	0	0	0	0	0	1	0	1	0	0	0	0	0	0	0	0	0	0	0	0	0	0	0	1	20
**2.132**	0	0	0	0	0	0	1	0	0	1	1	1	0	0	0	0	0	0	0	0	0	0	0	0	1	20
**2.133**	0	0	0	0	0	0	0	0	0	1	0	0	0	0	0	0	0	0	0	1	0	0	1	1	1	20
**2.134**	0	0	0	0	0	0	0	0	0	0	1	1	0	1	0	0	0	0	0	0	0	0	0	0	1	20
**2.135**	0	0	0	1	0	0	0	0	0	0	0	0	1	0	0	1	0	0	0	1	0	0	0	1	0	20
**2.136**	0	0	0	0	0	0	1	0	0	0	1	0	0	0	0	0	0	0	0	0	0	0	0	1	1	20
**2.137**	0	0	0	0	0	0	1	0	0	0	0	1	0	0	0	0	0	0	0	0	0	1	1	0	0	20
**2.138**	0	0	0	0	0	0	1	0	0	0	0	1	0	0	0	0	0	0	0	0	0	0	1	1	0	20
**2.139**	0	0	0	0	0	0	0	0	1	0	0	0	1	0	0	1	0	0	0	1	0	0	0	1	0	20
**2.140**	0	1	0	0	0	0	0	0	0	0	0	1	1	0	0	0	0	0	0	0	0	0	0	0	1	20
**2.141**	0	0	0	0	1	0	1	0	1	0	0	0	0	0	0	0	0	0	0	1	0	0	1	0	0	20
**2.142**	0	0	0	0	1	0	0	0	1	0	0	0	0	0	0	0	0	0	0	0	0	0	0	0	0	20
**2.143**	0	0	0	0	0	0	0	0	0	0	1	0	0	0	0	1	0	0	0	0	0	0	0	0	0	20
**2.144**	0	1	0	1	1	0	1	0	0	0	0	1	0	0	0	0	0	0	0	0	0	0	1	1	1	19
**2.145**	0	0	1	0	0	0	0	0	0	0	0	1	0	0	0	0	0	0	0	1	0	0	1	0	1	19
**2.146**	0	0	0	1	1	0	1	0	0	0	0	1	0	0	0	0	0	0	0	0	0	0	0	0	1	19
**2.147**	0	0	0	0	1	0	0	0	1	1	0	1	0	0	0	0	0	0	0	1	0	0	1	0	0	19
**2.148**	0	0	0	1	1	0	0	0	0	1	0	1	0	0	0	0	0	0	0	1	0	0	0	1	0	19
**2.149**	0	1	1	0	1	0	0	0	0	0	0	0	0	0	0	0	0	0	0	1	0	0	0	1	1	19
**2.150**	0	1	0	0	1	0	0	0	1	0	0	1	1	0	0	0	0	0	0	1	0	0	1	1	1	19
**2.151**	0	1	0	0	1	0	0	0	1	0	0	1	0	1	0	0	0	0	0	0	0	1	0	1	1	19
**2.152**	0	1	1	1	1	0	0	0	0	0	0	1	0	0	0	0	0	0	0	1	0	0	0	1	1	19
**2.153**	0	1	0	1	1	0	0	0	1	1	0	1	1	1	0	0	0	0	0	1	0	0	1	0	0	19
**2.154**	0	0	1	0	0	0	0	0	1	1	0	1	0	0	0	0	0	0	0	1	0	1	0	0	1	19
**2.155**	0	1	1	1	1	0	0	0	1	1	0	1	1	0	0	0	0	0	0	1	0	0	0	1	0	19
**2.156**	0	1	0	1	0	0	0	0	1	1	0	1	1	1	0	0	0	0	0	1	0	0	0	0	0	19
**2.157**	0	1	0	1	0	0	0	0	0	1	0	1	0	1	0	0	0	0	0	1	0	0	0	0	1	19
**2.158**	0	0	1	0	0	0	0	0	0	0	0	0	1	0	0	0	0	0	0	1	0	1	0	0	0	19
**2.159**	0	0	0	0	0	0	0	0	0	1	0	1	0	1	0	1	0	0	0	1	0	1	1	0	0	19
**2.160**	0	1	0	1	1	0	0	0	0	0	0	1	1	0	0	0	0	0	0	0	0	0	1	1	1	19
**2.161**	0	0	0	0	0	0	1	0	0	0	1	1	0	1	0	0	0	0	0	1	0	1	0	0	0	19
**2.162**	0	0	0	0	1	0	0	0	0	1	0	0	0	0	0	0	0	0	0	0	0	1	0	0	0	19
**2.163**	0	0	1	0	0	0	0	0	1	0	0	0	1	0	0	0	0	0	0	0	0	0	0	1	0	19
**2.164**	0	0	1	0	0	0	0	0	0	0	0	0	0	1	0	1	0	0	0	0	0	1	0	0	0	19
**2.165**	0	0	0	0	0	0	0	0	1	0	0	1	0	1	0	0	0	0	0	0	0	1	1	0	0	19
**2.166**	0	1	0	0	0	0	0	0	0	1	0	1	0	1	0	0	0	0	0	0	0	0	0	1	0	19
**2.167**	0	0	1	0	1	0	0	0	1	0	0	0	0	0	0	0	0	0	0	0	0	0	0	1	1	19
**2.168**	0	0	1	0	0	0	0	0	1	1	0	1	0	0	0	0	0	0	0	0	0	0	0	1	0	19
**2.169**	0	0	0	0	0	0	1	0	1	1	1	0	0	1	0	0	0	0	0	0	0	1	0	0	0	19
**2.170**	0	1	0	0	1	0	0	0	1	1	0	1	0	0	0	0	0	0	0	1	0	1	0	0	0	19
**2.171**	0	1	0	0	1	0	0	0	0	0	0	1	1	0	0	0	0	0	0	0	0	0	1	1	0	19
**2.172**	0	0	1	0	0	0	1	0	0	0	0	1	0	1	0	0	0	0	0	1	0	1	1	0	0	19
**2.173**	0	0	1	0	0	0	0	0	1	1	0	1	0	1	0	0	0	0	0	0	0	0	0	1	1	19
**2.174**	0	0	1	0	0	0	0	0	1	0	0	1	0	0	0	0	0	0	0	1	0	0	0	0	0	19
**2.175**	0	0	0	0	0	0	0	0	1	1	0	1	0	0	0	0	0	0	0	0	0	0	1	1	1	19
**2.176**	0	0	0	0	0	0	0	0	0	0	0	0	1	0	0	0	0	0	0	1	0	0	0	1	1	19
**2.177**	0	1	0	0	1	0	0	0	0	0	0	0	0	0	0	0	0	0	0	0	0	0	1	1	1	19
**2.178**	0	0	0	0	0	0	1	0	1	0	0	0	1	0	0	1	0	0	0	0	0	0	0	0	0	19
**2.179**	0	0	0	0	0	0	0	0	0	0	0	1	0	0	0	1	0	0	0	1	0	0	0	1	1	19
**2.180**	0	0	1	0	0	0	0	0	0	1	0	0	0	0	0	0	0	0	0	0	0	1	0	0	1	19
**2.181**	0	0	0	0	0	0	0	0	0	0	1	0	0	1	0	0	0	0	0	0	0	0	0	1	1	19
**2.182**	0	0	0	0	0	0	0	0	0	0	1	0	0	1	0	0	0	0	0	0	0	0	0	0	0	19
**2.183**	0	0	0	0	1	0	1	0	0	0	0	1	0	0	0	0	0	0	0	0	0	0	1	0	0	19
**2.184**	0	0	0	0	1	0	1	0	0	0	0	0	0	0	0	0	0	0	0	0	0	0	1	0	0	19
**2.185**	0	0	0	0	0	0	0	0	0	0	1	1	0	0	0	0	0	0	0	1	0	0	0	1	1	19
**2.186**	0	1	0	0	1	0	1	0	0	1	0	1	1	0	0	0	0	0	0	1	0	0	1	0	0	19
**2.187**	0	0	0	1	0	0	0	0	0	0	0	0	0	0	0	0	0	0	0	0	0	1	0	1	1	19
**2.188**	0	0	0	0	0	0	0	0	0	0	1	0	0	0	0	0	0	0	0	1	0	1	0	1	0	19
**2.189**	0	1	0	0	0	0	1	0	0	1	0	1	0	0	0	0	0	0	0	1	0	0	0	0	0	19
**2.190**	0	0	0	0	1	0	1	0	0	0	0	1	0	0	0	0	0	0	0	1	0	0	0	0	0	19
**2.191**	0	0	0	0	0	0	0	0	0	0	0	0	0	0	0	1	0	0	0	1	0	0	0	1	0	19
**2.192**	0	1	0	1	0	0	0	0	1	1	0	1	0	0	0	0	0	0	0	0	0	1	0	1	1	18
**2.193**	0	0	0	1	1	0	0	0	1	1	0	1	0	0	0	0	0	0	0	1	0	0	1	0	0	18
**2.194**	0	0	0	1	0	0	0	0	1	0	0	0	0	0	0	0	0	0	0	1	0	1	0	1	0	18
**2.195**	0	1	0	1	1	0	0	0	1	1	0	1	0	1	0	0	0	0	0	1	0	0	0	0	1	18

CMP	CD2	CD3	CD4	CD7	CD8	CD10	CD13	CD18	CD26	CD29	CD36	CD44	CD45RA	CD49f	CD54	CD56	CD57	CD58	CD62L	CD71	CD80	Cytokeratin	Histone_H1	HLA-DQ	HLA-DR	Freq. (voxel)
**2.196**	0	1	0	1	1	0	0	0	0	1	0	1	1	0	0	0	0	0	0	1	0	1	0	0	1	18
**2.197**	0	1	0	0	1	0	0	0	1	1	0	1	1	1	0	0	0	0	0	1	0	1	1	0	0	18
**2.198**	0	0	0	0	1	0	0	0	0	1	0	1	0	0	0	0	0	0	0	1	0	1	0	1	0	18
**2.199**	0	0	0	1	1	0	0	0	0	1	0	0	0	0	0	0	0	0	0	1	0	0	0	0	0	18
**2.200**	0	0	0	0	1	0	0	0	0	1	0	1	0	0	0	0	0	0	0	0	0	1	1	1	1	18
**2.201**	0	0	0	0	0	0	0	0	0	0	0	1	1	0	0	0	0	0	0	1	0	0	0	0	0	18
**2.202**	0	1	0	1	1	0	0	0	0	1	0	1	0	1	0	0	0	0	0	1	0	1	0	1	1	18
**2.203**	0	1	0	1	1	0	0	0	0	0	0	1	0	1	0	0	0	0	0	1	0	1	0	1	1	18
**2.204**	0	1	0	0	1	0	0	0	1	1	0	0	0	0	0	0	0	0	0	0	0	0	0	0	0	18
**2.205**	0	1	0	0	1	0	0	0	0	1	0	1	0	1	0	0	0	0	0	1	0	1	1	0	0	18
**2.206**	0	1	0	0	1	0	0	0	0	0	0	1	1	0	0	0	0	0	0	1	0	1	1	1	1	18
**2.207**	0	0	1	0	0	0	0	0	0	0	0	0	1	1	0	0	0	0	0	1	0	0	0	1	0	18
**2.208**	0	1	0	1	1	0	0	0	0	1	0	1	0	0	0	0	0	0	0	1	0	1	1	0	0	18
**2.209**	0	1	0	1	0	0	1	0	0	0	0	0	0	0	0	0	0	0	0	1	0	0	1	0	0	18
**2.210**	0	0	1	0	0	0	1	0	0	0	0	0	1	0	0	0	0	0	0	1	0	1	0	0	0	18
**2.211**	0	0	0	1	0	0	0	0	0	1	0	0	0	0	0	0	0	0	0	1	0	0	1	0	0	18
**2.212**	0	0	0	0	1	0	1	0	1	0	0	1	0	0	0	1	0	0	0	0	0	0	0	1	1	18
**2.213**	0	1	0	1	0	0	0	0	0	1	0	1	0	1	0	0	0	0	0	0	0	0	1	0	0	18
**2.214**	0	0	0	1	1	0	0	0	0	1	0	1	0	0	0	0	0	0	0	1	0	1	0	0	0	18
**2.215**	0	0	0	1	0	0	0	0	1	0	0	0	0	1	0	0	0	0	0	0	0	1	0	0	0	18
**2.216**	0	0	0	0	0	0	1	0	1	1	0	1	0	1	0	0	0	0	0	0	0	0	0	0	0	18
**2.217**	0	0	0	0	0	0	1	0	1	0	0	1	0	0	0	0	0	0	0	1	0	1	0	0	1	18
**2.218**	0	1	0	0	0	0	0	0	1	1	0	1	1	0	0	0	0	0	0	0	0	0	0	0	0	18
**2.219**	0	1	0	0	0	0	0	0	0	0	0	1	1	1	0	0	0	0	0	0	0	0	0	1	1	18
**2.220**	0	0	0	1	0	0	1	0	0	1	0	1	0	1	0	0	0	0	0	0	0	0	0	0	1	18
**2.221**	0	1	0	1	1	0	0	0	1	1	0	1	1	1	0	0	0	0	0	0	0	0	0	0	0	18
**2.222**	0	1	0	1	1	0	0	0	0	1	0	1	1	0	0	0	0	0	0	0	0	0	0	1	0	18
**2.223**	0	0	0	1	1	0	0	0	0	0	0	0	0	0	0	0	0	0	0	0	0	0	0	0	1	18
**2.224**	0	0	0	0	0	0	0	0	1	1	0	0	0	0	0	0	0	0	0	0	0	0	1	0	0	18
**2.225**	0	1	1	1	1	0	0	0	0	0	0	1	0	0	0	0	0	0	0	0	0	0	0	0	0	18
**2.226**	0	1	0	0	0	0	0	0	0	0	0	0	0	1	0	0	0	0	0	0	0	0	1	0	1	18
**2.227**	0	0	0	0	0	0	1	0	0	0	0	0	1	1	0	0	0	0	0	0	0	0	0	0	0	18
**2.228**	0	0	0	0	0	0	0	0	0	0	0	1	0	0	0	1	0	0	0	0	0	1	0	1	0	18
**2.229**	0	1	0	0	0	0	0	0	1	0	0	1	0	1	0	0	0	0	0	0	0	0	0	1	1	18
**2.230**	0	0	0	1	1	0	0	0	0	1	0	1	1	0	0	0	0	0	0	1	0	0	0	0	0	18
**2.231**	0	0	0	0	0	0	1	0	1	1	0	1	0	1	0	0	0	0	0	1	0	1	1	0	0	18
**2.232**	0	0	0	0	0	0	1	0	1	0	0	1	0	1	0	0	0	0	0	0	0	1	0	0	1	18
**2.233**	0	0	0	0	0	0	0	0	1	1	0	1	0	1	0	0	0	0	0	1	0	1	1	0	1	18
**2.234**	0	1	0	1	0	0	0	0	0	1	0	1	0	0	0	0	0	0	0	1	0	0	0	1	0	18
**2.235**	0	0	1	0	0	0	0	0	0	0	0	0	0	0	0	1	0	0	0	0	0	0	1	1	0	18
**2.236**	0	0	0	0	0	0	0	0	1	1	0	1	0	1	0	0	0	0	0	0	0	0	0	1	1	18
**2.237**	0	1	0	1	1	0	0	0	0	1	0	1	0	0	0	0	0	0	0	0	0	1	0	0	1	18
**2.238**	0	1	0	0	0	0	0	0	0	1	0	1	0	0	0	0	0	0	0	1	0	1	0	0	0	18
**2.239**	0	1	0	0	0	0	0	0	0	0	0	1	1	0	0	0	0	0	0	1	0	1	1	0	0	18
**2.240**	0	0	1	0	0	0	1	0	0	1	0	1	0	0	0	0	0	0	0	1	0	0	0	0	0	18
**2.241**	0	0	0	0	0	0	1	0	0	0	1	0	0	1	0	0	0	0	0	0	0	1	0	1	1	18
**2.242**	0	1	0	0	0	0	0	0	1	0	0	1	1	0	0	0	0	0	0	1	0	0	0	0	0	18
**2.243**	0	0	0	0	0	0	1	0	0	0	1	0	0	0	0	0	0	0	0	1	0	1	0	0	0	18
**2.244**	0	0	0	0	0	0	0	0	1	0	0	1	1	0	0	0	0	0	0	0	0	1	0	0	1	18
**2.245**	0	0	0	0	0	0	0	0	1	0	0	0	0	0	0	0	0	0	0	0	0	1	1	0	1	18
**2.246**	0	1	0	1	0	0	0	0	0	0	0	1	1	0	0	0	0	0	0	0	0	1	0	0	0	18
**2.247**	0	0	0	0	1	0	1	0	0	1	0	1	0	0	0	0	0	0	0	0	0	1	0	0	0	18
**2.248**	0	0	0	0	0	0	0	0	0	1	0	0	1	0	0	1	0	0	0	0	0	0	0	1	0	18
**2.249**	0	1	0	1	0	0	0	0	1	0	0	0	0	0	0	0	0	0	0	0	0	0	1	0	0	18
**2.250**	0	1	0	0	1	0	1	0	0	0	0	1	1	0	0	0	0	0	0	1	0	0	0	0	0	18
**2.251**	0	0	0	0	0	0	0	0	0	0	0	0	1	0	0	0	0	0	0	1	0	1	0	0	1	18
**2.252**	0	0	0	1	0	0	0	0	1	0	0	1	0	0	0	0	0	0	0	1	0	0	0	0	0	17
**2.253**	0	0	0	1	0	0	0	0	1	0	0	1	0	0	0	0	0	0	0	0	0	1	0	1	0	17
**2.254**	0	1	0	1	1	0	1	0	1	1	0	1	1	0	0	0	0	0	0	1	0	0	0	0	0	17
**2.255**	0	1	0	1	1	0	1	0	0	0	0	1	1	0	0	0	0	0	0	1	0	0	0	0	1	17
**2.256**	0	1	0	1	1	0	0	0	1	1	0	1	0	1	0	0	0	0	0	0	0	0	0	0	0	17
**2.257**	0	1	0	0	1	0	0	0	1	1	0	1	0	0	0	0	0	0	0	1	0	1	0	1	1	17
**2.258**	0	0	0	0	0	0	0	0	0	1	0	1	1	0	0	0	0	0	0	1	0	0	1	0	0	17
**2.259**	0	1	0	1	0	0	0	0	1	0	0	1	0	1	0	0	0	0	0	1	0	0	0	0	0	17
**2.260**	0	0	1	0	0	0	0	0	1	1	0	1	0	0	0	0	0	0	0	1	0	1	0	0	0	17
**2.261**	0	0	0	1	1	0	0	0	1	1	0	1	0	0	0	0	0	0	0	0	0	0	0	0	1	17
**2.262**	0	1	0	0	0	0	0	0	1	0	0	1	0	1	0	0	0	0	0	0	0	0	1	0	0	17
**2.263**	0	0	1	0	1	0	1	0	0	0	0	0	1	0	0	1	0	0	0	1	0	1	0	1	0	17
**2.264**	0	0	1	0	0	0	1	0	0	1	0	1	0	0	0	0	0	0	0	1	0	0	0	1	0	17
**2.265**	0	0	1	0	0	0	0	0	1	0	0	1	0	1	0	0	0	0	0	1	0	1	0	1	0	17
**2.266**	0	0	0	0	0	0	0	0	1	0	0	1	1	0	0	0	0	0	0	1	0	1	0	1	1	17
**2.267**	0	0	0	0	0	0	0	0	0	0	0	1	0	1	0	1	0	0	0	0	0	1	0	0	0	17
**2.268**	0	1	1	0	1	0	0	0	0	0	0	0	0	0	0	0	0	0	0	1	0	0	1	0	0	17
**2.269**	0	1	0	0	0	0	0	0	0	0	0	1	0	1	0	0	0	0	0	0	0	0	1	1	1	17
**2.270**	0	0	1	0	0	0	1	0	0	1	0	1	0	1	0	0	0	0	0	0	0	0	1	1	1	17
**2.271**	0	0	1	0	0	0	1	0	0	1	0	0	0	0	0	0	0	0	0	1	0	0	0	1	1	17
**2.272**	0	0	1	0	0	0	0	0	0	1	0	0	0	1	0	0	0	0	0	0	0	0	1	1	0	17
**2.273**	0	0	0	0	1	0	0	0	0	1	0	1	0	0	0	0	0	0	0	0	0	0	1	1	1	17
**2.274**	0	0	0	0	0	0	1	0	1	1	0	0	1	1	0	0	0	0	0	1	0	1	1	0	0	17
**2.275**	0	0	0	0	0	0	0	0	0	1	0	0	0	0	0	0	0	0	0	0	0	1	1	1	1	17
**2.276**	0	1	0	1	1	0	0	0	0	0	0	1	0	0	0	0	0	0	0	1	0	1	0	1	0	17
**2.277**	0	0	1	0	0	0	0	0	0	1	0	0	0	1	0	0	0	0	0	0	0	0	1	0	0	17
**2.278**	0	0	0	1	0	0	1	0	0	1	0	0	0	1	0	0	0	0	0	1	0	1	0	0	0	17
**2.279**	0	0	0	1	0	0	0	0	0	0	0	1	0	1	0	0	0	0	0	0	0	1	0	0	0	17
**2.280**	0	0	1	1	0	0	1	0	1	1	0	1	0	1	0	0	0	0	0	1	0	1	0	1	1	17
**2.281**	0	0	1	0	0	0	1	0	1	0	0	0	0	0	0	0	0	0	0	0	0	0	0	1	0	17
**2.282**	0	0	0	0	1	0	0	0	1	1	0	1	1	0	0	0	0	0	0	0	0	1	0	0	0	17
**2.283**	0	0	0	0	0	0	1	0	0	0	0	0	0	1	0	0	0	0	0	0	0	0	1	1	1	17
**2.284**	0	0	0	0	0	0	0	0	1	0	1	1	0	1	0	0	0	0	0	1	0	1	0	0	0	17
**2.285**	0	0	0	0	0	0	0	0	1	0	0	0	1	0	0	1	0	0	0	0	0	0	0	1	0	17
**2.286**	0	0	0	0	0	0	0	0	0	0	1	0	0	0	0	0	0	0	0	0	0	0	1	1	1	17
**2.287**	0	1	0	0	1	0	0	0	1	1	0	1	1	0	0	0	0	0	0	0	0	0	1	0	1	17
**2.288**	0	1	0	0	1	0	0	0	0	0	0	1	0	0	0	0	0	0	0	1	0	1	1	1	0	17
**2.289**	0	1	0	0	0	0	1	0	0	1	0	1	0	1	0	0	0	0	0	0	0	1	0	1	1	17
**2.290**	0	0	1	0	0	0	1	0	0	1	0	1	0	1	0	0	0	0	0	1	0	0	1	1	1	17
**2.291**	0	0	0	0	0	0	0	0	0	0	0	1	0	1	0	0	0	0	0	1	0	0	0	0	1	17
**2.292**	0	1	0	1	0	0	0	0	0	0	0	0	1	0	0	0	0	0	0	1	0	0	1	0	0	17
**2.293**	0	1	0	0	1	0	0	0	1	0	0	1	0	0	0	0	0	0	0	1	0	1	1	0	0	17
**2.294**	0	1	0	0	0	0	0	0	1	0	0	1	0	0	0	0	0	0	0	0	0	0	1	1	1	17
**2.295**	0	0	1	0	0	0	0	0	1	0	0	0	0	0	0	0	0	0	0	0	0	1	0	0	1	17
**2.296**	0	0	1	0	1	0	0	0	0	0	0	1	0	0	0	0	0	0	0	0	0	0	0	0	0	17
**2.297**	0	0	1	0	0	0	1	0	1	1	1	1	0	1	0	0	0	0	0	1	0	0	0	1	1	17
**2.298**	0	0	0	1	0	0	0	0	0	1	0	0	0	0	0	0	0	0	0	0	0	0	1	1	1	17
**2.299**	0	0	0	0	0	0	1	0	1	1	0	1	0	1	0	1	0	0	0	0	0	0	0	1	1	17
**2.300**	0	0	0	0	0	0	1	0	0	1	1	0	0	1	0	0	0	0	0	0	0	0	0	0	0	17
**2.301**	0	0	0	0	0	0	1	0	0	1	0	0	0	1	0	0	0	0	0	1	0	1	1	1	0	17
**2.302**	0	0	0	0	0	0	0	0	0	0	1	1	0	1	0	0	0	0	0	1	0	0	0	1	1	17
**2.303**	0	0	0	0	0	0	0	0	0	0	0	0	0	1	0	0	0	0	0	1	0	0	0	0	1	17
**2.304**	0	1	0	0	1	0	1	0	0	1	0	1	0	1	0	0	0	0	0	1	0	1	0	0	1	17
**2.305**	0	1	0	0	1	0	0	0	0	1	0	1	0	1	0	0	0	0	0	1	0	1	0	0	0	17
**2.306**	0	1	0	0	1	0	0	0	0	0	0	1	1	0	0	0	0	0	0	0	0	1	1	0	1	17
**2.307**	0	0	1	0	0	0	1	0	0	1	0	1	0	1	0	0	0	0	0	0	0	0	0	0	0	17
**2.308**	0	0	0	0	0	0	0	0	0	1	1	0	0	0	0	0	0	0	0	1	0	1	0	0	0	17
**2.309**	0	0	0	0	0	0	0	0	0	0	1	0	0	0	0	0	0	0	0	1	0	1	1	0	0	17
**2.310**	0	1	0	1	0	0	0	0	1	0	0	1	0	0	0	0	0	0	0	0	0	0	0	1	0	17
**2.311**	0	0	1	0	0	0	0	0	0	1	0	0	0	0	0	1	0	0	0	0	0	0	0	0	0	17
**2.312**	0	0	0	1	0	0	0	0	0	0	0	0	0	0	0	0	0	0	0	0	0	0	0	0	1	17
**2.313**	0	0	0	0	0	0	1	0	0	1	0	1	0	0	0	0	0	0	0	0	0	1	1	0	0	17
**2.314**	0	0	0	0	0	0	0	0	0	1	0	1	0	0	0	0	0	0	0	1	0	1	1	0	1	17
**2.315**	0	1	0	0	1	0	0	0	0	1	0	0	0	0	0	0	0	0	0	1	0	0	1	0	0	17
**2.316**	0	0	0	0	0	0	0	0	0	1	1	0	0	1	0	0	0	0	0	0	0	0	0	1	1	17
**2.317**	0	1	0	1	0	0	0	0	1	0	0	1	0	0	0	0	0	0	0	0	0	1	0	0	0	17

CMP	CD2	CD3	CD4	CD7	CD8	CD10	CD13	CD18	CD26	CD29	CD36	CD44	CD45RA	CD49f	CD54	CD56	CD57	CD58	CD62L	CD71	CD80	Cytokeratin	Histone_H1	HLA-DQ	HLA-DR	Freq. (voxel)
**2.318**	0	0	0	0	0	0	0	0	0	1	1	1	0	0	0	0	0	0	0	0	0	1	0	1	0	17
**2.319**	0	1	0	1	0	0	0	0	0	0	0	0	1	0	0	0	0	0	0	0	0	0	0	0	0	17
**2.320**	0	0	0	0	0	0	0	0	0	0	1	1	0	0	0	0	0	0	0	0	0	1	1	0	1	17
**2.321**	0	0	0	0	0	0	0	0	1	0	0	0	1	0	0	1	0	0	0	1	0	0	1	0	0	17
**2.322**	0	0	0	1	0	0	0	0	0	1	0	0	0	0	0	0	0	0	0	1	0	0	0	1	1	16
**2.323**	0	1	0	1	1	0	0	0	1	1	0	1	0	0	0	0	0	0	0	1	0	1	0	0	1	16
**2.324**	0	1	0	1	1	0	0	0	0	1	0	1	1	1	0	0	0	0	0	1	0	0	1	0	0	16
**2.325**	0	0	0	1	1	0	0	0	1	1	0	1	0	0	0	0	0	0	0	0	0	0	0	1	1	16
**2.326**	0	0	0	1	1	0	0	0	0	0	0	0	0	0	0	0	0	0	0	1	0	0	0	1	1	16
**2.327**	0	0	0	0	1	0	0	0	1	1	0	0	0	0	0	0	0	0	0	0	0	0	0	0	0	16
**2.328**	0	1	0	1	1	0	1	0	1	0	0	1	0	0	0	1	0	0	0	1	0	0	0	1	1	16
**2.329**	0	1	0	1	1	0	0	0	0	1	0	1	1	0	0	0	0	0	0	1	0	1	0	1	1	16
**2.330**	0	0	0	0	1	0	0	0	1	0	0	1	1	0	0	0	0	0	0	1	0	1	0	1	1	16
**2.331**	0	1	0	0	1	0	0	0	1	1	0	1	0	0	0	0	0	0	0	0	0	1	1	0	0	16
**2.332**	0	1	0	0	1	0	0	0	0	1	0	1	0	1	0	0	0	0	0	1	0	0	1	0	0	16
**2.333**	0	0	0	0	0	0	0	0	0	1	0	0	1	1	0	0	0	0	0	0	0	0	0	1	0	16
**2.334**	0	1	1	0	1	0	0	0	0	0	0	1	0	0	0	0	0	0	0	1	0	0	1	0	0	16
**2.335**	0	1	0	0	0	0	0	0	1	1	0	1	1	0	0	0	0	0	0	1	0	0	1	0	0	16
**2.336**	0	1	0	0	0	0	0	0	0	1	0	1	0	0	0	0	0	0	0	0	0	1	1	1	0	16
**2.337**	0	0	1	0	0	0	1	0	0	0	0	0	1	0	0	1	0	0	0	0	0	1	0	1	0	16
**2.338**	0	0	0	1	0	0	1	0	0	1	0	1	0	1	0	1	0	0	0	0	0	0	0	1	1	16
**2.339**	0	0	0	0	0	0	1	0	0	0	0	1	0	1	0	0	0	0	0	1	0	1	0	1	1	16
**2.340**	0	1	1	0	1	0	0	0	0	1	0	1	1	0	0	0	0	0	0	0	0	0	1	1	1	16
**2.341**	0	1	1	0	1	0	0	0	0	1	0	0	0	0	0	0	0	0	0	0	0	0	0	1	1	16
**2.342**	0	1	0	1	1	0	0	0	1	1	0	1	1	0	0	0	0	0	0	0	0	0	1	0	1	16
**2.343**	0	1	0	0	0	0	0	0	1	1	0	1	1	1	0	0	0	0	0	1	0	0	0	0	0	16
**2.344**	0	1	0	0	0	0	0	0	0	1	0	1	1	1	0	0	0	0	0	0	0	0	1	0	0	16
**2.345**	0	0	0	1	1	0	0	0	0	0	0	1	0	0	0	0	0	0	0	0	0	1	0	0	1	16
**2.346**	0	0	0	0	0	0	0	0	0	1	0	1	0	0	0	1	0	0	0	0	0	1	0	1	0	16
**2.347**	0	0	0	0	0	0	0	0	0	0	1	0	1	0	0	0	0	0	0	0	0	0	0	0	0	16
**2.348**	0	1	0	1	0	0	0	0	0	0	0	1	0	0	0	0	0	0	0	1	0	1	0	1	0	16
**2.349**	0	0	0	0	0	0	0	0	0	1	0	1	1	0	0	0	0	0	0	0	0	0	0	1	0	16
**2.350**	0	1	1	0	0	0	0	0	1	0	0	1	0	0	0	0	0	0	0	0	0	0	0	1	0	16
**2.351**	0	0	0	0	0	0	1	0	1	1	0	1	0	1	0	0	0	0	0	1	0	0	1	1	1	16
**2.352**	0	0	0	0	0	0	1	0	1	0	0	0	0	1	0	0	0	0	0	0	0	1	0	1	1	16
**2.353**	0	0	0	0	0	0	1	0	0	0	0	1	0	1	0	0	0	0	0	0	0	1	0	1	1	16
**2.354**	0	0	0	0	0	0	0	0	0	0	1	1	0	0	0	0	0	0	0	1	0	1	0	1	1	16
**2.355**	0	0	0	1	0	0	1	0	0	1	1	1	0	1	0	0	0	0	0	1	0	0	0	1	1	16
**2.356**	0	0	0	1	0	0	0	0	0	0	0	0	0	0	0	0	0	0	0	1	0	1	0	0	1	16
**2.357**	0	0	0	0	0	0	0	0	1	0	0	0	0	1	0	0	0	0	0	0	0	0	0	0	0	16
**2.358**	0	1	0	0	1	0	0	0	1	1	0	1	1	0	0	0	0	0	0	1	0	1	0	0	0	16
**2.359**	0	0	0	1	0	0	0	0	0	0	0	0	0	0	0	0	0	0	0	0	0	0	1	1	1	16
**2.360**	0	1	0	1	1	0	0	0	0	0	0	0	0	0	0	0	0	0	0	1	0	0	0	0	1	16
**2.361**	0	1	0	0	1	0	0	0	1	0	0	0	0	0	0	0	0	0	0	0	0	1	0	0	1	16
**2.362**	0	1	0	0	1	0	0	0	0	0	0	1	1	0	0	0	0	0	0	1	0	1	1	0	0	16
**2.363**	0	1	0	0	0	0	0	0	1	1	0	0	0	0	0	0	0	0	0	0	0	0	1	0	0	16
**2.364**	0	0	1	0	0	0	0	0	0	1	0	0	0	0	0	0	0	0	0	0	0	1	0	1	0	16
**2.365**	0	0	1	0	0	0	0	0	0	0	0	1	0	0	0	0	0	0	0	1	0	1	1	0	0	16
**2.366**	0	0	0	0	0	0	1	0	1	1	0	1	0	1	0	1	0	0	0	1	0	0	0	1	1	16
**2.367**	0	0	0	0	0	0	1	0	0	1	1	1	0	1	0	0	0	0	0	0	0	0	0	1	0	16
**2.368**	0	1	0	1	1	0	0	0	1	0	0	1	1	0	0	0	0	0	0	0	0	1	0	1	1	16
**2.369**	0	1	0	1	1	0	0	0	1	0	0	1	1	0	0	0	0	0	0	0	0	1	0	0	1	16
**2.370**	0	1	0	1	1	0	0	0	0	1	0	0	0	0	0	0	0	0	0	0	0	0	1	0	0	16
**2.371**	0	0	1	1	0	0	0	0	0	0	0	0	1	0	0	1	0	0	0	0	0	0	0	1	0	16
**2.372**	0	0	0	0	0	0	1	0	0	1	0	0	1	1	0	0	0	0	0	0	0	1	0	0	0	16
**2.373**	0	0	0	0	0	0	0	0	1	0	0	0	0	1	0	0	0	0	0	1	0	0	0	1	1	16
**2.374**	0	0	0	0	0	0	0	0	0	1	0	1	0	1	0	0	0	0	0	1	0	0	1	1	1	16
**2.375**	0	0	1	0	0	0	1	0	0	0	0	0	0	1	0	0	0	0	0	1	0	0	0	0	0	16
**2.376**	0	0	0	1	1	0	1	0	0	0	0	1	0	0	0	0	0	0	0	0	0	0	0	0	0	16
**2.377**	0	1	0	0	0	0	0	0	0	1	0	0	0	0	0	0	0	0	0	1	0	0	0	0	0	16
**2.378**	0	0	1	0	0	0	0	0	0	1	1	0	0	1	0	0	0	0	0	0	0	0	0	1	1	16
**2.379**	0	0	0	0	0	0	0	0	1	1	0	1	0	0	0	0	0	0	0	0	0	0	1	1	0	16
**2.380**	0	1	0	0	1	0	0	0	0	0	0	1	1	0	0	0	0	0	0	0	0	1	0	1	0	16
**2.381**	0	0	0	0	1	0	0	0	0	0	1	1	0	0	0	0	0	0	0	0	0	0	0	0	1	16
**2.382**	0	0	0	0	1	0	0	0	0	0	0	1	0	0	0	1	0	0	0	0	0	0	0	0	0	16
**2.383**	0	1	0	0	0	0	0	0	1	0	0	1	1	0	0	0	0	0	0	0	0	0	0	0	0	16
**2.384**	0	1	0	0	0	0	0	0	0	1	0	1	0	0	0	0	0	0	0	1	0	1	1	0	0	16
**2.385**	0	0	0	0	0	0	1	0	0	0	0	1	0	0	0	0	0	0	0	1	0	0	1	0	0	16
**2.386**	0	0	0	0	0	0	1	0	0	0	0	0	0	0	0	0	0	0	0	1	0	1	0	1	0	16
**2.387**	0	1	0	1	0	0	1	0	0	0	0	1	0	0	0	0	0	0	0	1	0	0	0	0	0	16
**2.388**	0	1	0	0	1	0	1	0	0	1	0	1	0	0	0	0	0	0	0	0	0	1	0	0	0	16
**2.389**	0	0	1	0	0	0	0	0	0	0	0	0	1	1	0	1	0	0	0	0	0	1	0	1	0	16
**2.390**	0	1	0	0	0	0	0	0	0	0	0	1	1	0	0	0	0	0	0	0	0	1	0	0	1	16
**2.391**	0	0	0	0	1	0	0	0	0	0	0	0	1	0	0	0	0	0	0	1	0	0	0	0	0	16
**2.392**	0	0	0	0	0	0	1	0	1	0	0	0	0	0	0	0	0	0	0	1	0	1	1	0	0	16
**2.393**	0	1	0	0	0	0	0	0	1	1	0	1	0	0	0	0	0	0	0	0	0	0	1	1	0	15
**2.394**	0	0	0	0	1	0	0	0	1	0	0	1	0	0	0	0	0	0	0	0	0	1	0	1	0	15
**2.395**	0	0	0	0	1	0	0	0	0	0	0	1	1	0	0	0	0	0	0	1	0	1	0	0	0	15
**2.396**	0	1	0	1	1	0	1	0	1	0	0	1	0	0	0	0	0	0	0	1	0	0	1	1	1	15
**2.397**	0	1	0	1	1	0	1	0	1	1	0	1	0	0	0	0	0	0	0	0	0	0	0	1	1	15
**2.398**	0	0	0	1	1	0	0	0	0	0	0	1	1	0	0	0	0	0	0	1	0	0	0	1	1	15
**2.399**	0	0	0	1	0	0	0	0	1	0	0	1	0	0	0	0	0	0	0	0	0	1	0	1	1	15
**2.400**	0	0	1	0	0	0	1	0	0	0	0	0	1	0	0	1	0	0	0	1	0	1	0	1	0	15
**2.401**	0	0	0	0	1	0	0	0	1	1	0	1	0	1	0	0	0	0	0	1	0	1	0	1	1	15
**2.402**	0	0	0	0	0	0	1	0	0	1	0	0	1	0	0	0	0	0	0	0	0	0	0	0	0	15
**2.403**	0	1	0	1	0	0	0	0	1	1	0	1	0	1	0	0	0	0	0	1	0	0	0	0	0	15
**2.404**	0	1	0	0	0	0	1	0	0	0	0	1	0	0	0	0	0	0	0	0	0	0	0	1	0	15
**2.405**	0	1	0	0	0	0	0	0	0	1	0	1	1	0	0	0	0	0	0	0	0	1	0	1	0	15
**2.406**	0	0	0	1	0	0	0	0	1	1	0	1	0	0	0	0	0	0	0	0	0	1	0	0	0	15
**2.407**	0	1	1	1	1	0	1	0	0	1	0	1	0	0	0	0	0	0	0	1	0	0	0	1	1	15
**2.408**	0	1	1	0	1	0	0	0	0	0	0	1	0	0	0	0	0	0	0	1	0	0	0	1	1	15
**2.409**	0	1	0	0	0	0	0	0	1	1	0	1	1	0	0	0	0	0	0	0	0	1	1	0	0	15
**2.410**	0	0	1	0	1	0	0	0	0	0	0	0	0	0	0	0	0	0	0	1	0	0	0	1	1	15
**2.411**	0	0	0	0	1	0	0	0	1	1	0	0	0	0	0	0	0	0	0	0	0	0	1	0	0	15
**2.412**	0	1	1	1	1	0	0	0	0	0	0	1	0	0	0	0	0	0	0	1	0	0	0	0	1	15
**2.413**	0	1	1	0	0	0	0	0	0	0	0	1	0	0	0	0	0	0	0	1	0	0	1	0	0	15
**2.414**	0	1	0	1	1	0	0	0	0	1	0	1	1	0	0	0	0	0	0	1	0	0	0	1	0	15
**2.415**	0	1	0	0	0	0	0	0	0	0	0	0	0	1	0	0	0	0	0	0	0	1	1	0	1	15
**2.416**	0	0	1	0	0	0	1	0	0	0	1	0	0	0	0	0	0	0	0	0	0	0	0	0	0	15
**2.417**	0	0	0	1	0	0	0	0	1	1	0	1	0	0	0	0	0	0	0	0	0	0	0	1	1	15
**2.418**	0	0	0	0	0	0	0	0	1	1	0	1	1	0	0	0	0	0	0	0	0	0	0	0	0	15
**2.419**	0	0	0	0	0	0	0	0	0	1	1	0	0	1	0	0	0	0	0	1	0	1	0	0	0	15
**2.420**	0	1	1	1	1	0	0	0	1	1	0	1	0	0	0	0	0	0	0	0	0	0	0	1	0	15
**2.421**	0	1	1	1	0	0	0	0	1	1	0	1	0	0	0	0	0	0	0	1	0	0	0	0	0	15
**2.422**	0	1	1	0	0	0	0	0	0	0	0	0	1	0	0	0	0	0	0	0	0	0	0	0	0	15
**2.423**	0	1	0	0	0	0	0	0	0	1	0	1	0	0	0	0	0	0	0	1	0	1	0	1	1	15
**2.424**	0	1	0	0	0	0	0	0	0	0	0	1	0	1	0	0	0	0	0	1	0	0	0	1	1	15
**2.425**	0	1	0	1	1	0	0	0	0	0	0	0	1	0	0	0	0	0	0	1	0	0	1	0	0	15
**2.426**	0	0	1	0	0	0	0	0	0	0	0	0	0	1	0	0	0	0	0	0	0	0	1	1	0	15
**2.427**	0	0	0	0	1	0	0	0	1	0	0	1	0	0	0	0	0	0	0	0	0	1	1	0	1	15
**2.428**	0	1	0	0	1	0	0	0	0	0	0	0	1	0	0	0	0	0	0	1	0	0	0	0	0	15
**2.429**	0	0	0	1	1	0	0	0	1	0	0	0	0	0	0	0	0	0	0	0	0	1	0	0	0	15
**2.430**	0	0	0	0	1	0	0	0	1	0	0	1	1	0	0	0	0	0	0	0	0	1	0	0	0	15
**2.431**	0	1	0	0	0	0	0	0	0	0	0	0	1	0	0	0	0	0	0	0	0	0	1	0	1	15
**2.432**	0	0	1	0	0	0	1	0	0	1	0	0	0	1	0	0	0	0	0	1	0	0	0	0	0	15
**2.433**	0	0	1	0	0	0	0	0	0	0	0	0	1	0	0	1	0	0	0	0	0	0	0	1	1	15
**2.434**	0	0	0	1	0	0	0	0	0	0	1	0	0	0	0	0	0	0	0	0	0	1	0	0	0	15
**2.435**	0	0	0	0	0	0	0	0	0	1	0	1	1	1	0	0	0	0	0	1	0	0	0	1	1	15
**2.436**	0	0	0	0	0	0	0	0	0	1	0	0	0	0	0	0	0	0	0	0	0	1	1	1	0	15
**2.437**	0	1	0	1	1	0	0	0	0	0	0	1	0	1	0	0	0	0	0	0	0	0	1	0	0	15
**2.438**	0	0	1	0	0	0	0	0	0	1	0	1	0	0	0	0	0	0	0	1	0	1	0	0	1	15
**2.439**	0	0	0	0	0	0	1	0	0	1	0	0	1	1	0	0	0	0	0	1	0	1	1	0	0	15

CMP	CD2	CD3	CD4	CD7	CD8	CD10	CD13	CD18	CD26	CD29	CD36	CD44	CD45RA	CD49f	CD54	CD56	CD57	CD58	CD62L	CD71	CD80	Cytokeratin	Histone_H1	HLA-DQ	HLA-DR	Freq. (voxel)
**2.440**	0	1	0	1	0	0	0	0	0	1	0	0	0	0	0	0	0	0	0	1	0	0	0	0	0	15
**2.441**	0	1	0	0	1	0	1	0	0	1	0	1	0	1	0	0	0	0	0	0	0	1	0	0	1	15
**2.442**	0	1	0	0	0	0	0	0	0	1	0	1	0	1	0	0	0	0	0	0	0	0	0	0	1	15
**2.443**	0	0	0	0	0	0	1	0	1	0	0	1	0	1	0	0	0	0	0	0	0	1	1	0	0	15
**2.444**	0	0	0	0	0	0	1	0	1	0	0	0	1	0	0	0	0	0	0	0	0	0	0	1	0	15
**2.445**	0	0	0	0	0	0	1	0	0	1	0	0	0	1	0	0	0	0	0	0	0	1	1	0	1	15
**2.446**	0	1	0	0	0	0	0	0	1	0	0	1	0	0	0	0	0	0	0	1	0	0	0	0	1	15
**2.447**	0	0	1	1	0	0	0	0	0	1	0	0	0	1	0	0	0	0	0	1	0	0	0	1	1	15
**2.448**	0	0	0	0	0	0	0	0	0	0	0	1	0	1	0	0	0	0	0	0	0	0	1	0	1	15
**2.449**	0	1	0	0	1	0	0	0	0	1	1	1	0	1	0	0	0	0	0	0	0	0	0	0	1	15
**2.450**	0	0	0	1	0	0	1	0	0	1	0	1	0	0	0	0	0	0	0	0	0	0	0	0	0	15
**2.451**	0	0	0	0	0	0	1	0	0	0	1	1	0	1	0	0	0	0	0	0	0	0	0	0	0	15
**2.452**	0	1	0	0	0	0	0	0	0	0	0	0	1	0	0	0	0	0	0	1	0	0	1	0	0	15
**2.453**	0	1	0	1	1	0	0	0	1	0	0	0	0	0	0	0	0	0	0	1	0	0	0	0	0	15
**2.454**	0	1	0	0	1	0	1	0	0	0	0	1	0	0	0	0	0	0	0	1	0	0	1	0	0	15
**2.455**	0	1	0	0	0	0	0	0	0	0	0	0	1	0	0	0	0	0	0	0	0	1	0	0	1	15
**2.456**	0	1	0	0	0	0	0	0	0	0	0	0	1	0	0	0	0	0	0	0	0	0	0	0	1	15
**2.457**	0	1	1	1	1	0	0	0	1	0	0	1	0	0	0	0	0	0	0	1	0	1	0	1	1	14
**2.458**	0	0	0	1	1	0	0	0	1	1	0	1	1	0	0	0	0	0	0	1	0	0	0	1	1	14
**2.459**	0	0	0	0	1	0	0	0	1	0	0	1	1	0	0	0	0	0	0	0	0	0	0	0	1	14
**2.460**	0	1	0	1	1	0	0	0	1	0	0	1	0	0	0	0	0	0	0	0	0	1	0	1	1	14
**2.461**	0	1	0	0	1	0	0	0	1	1	0	1	1	0	0	0	0	0	0	0	0	1	0	1	0	14
**2.462**	0	1	0	0	1	0	0	0	1	1	0	1	0	0	0	0	0	0	0	0	0	1	1	1	0	14
**2.463**	0	0	0	1	1	0	0	0	1	1	0	1	0	0	0	0	0	0	0	1	0	1	0	0	0	14
**2.464**	0	0	0	1	1	0	0	0	0	1	0	1	1	0	0	0	0	0	0	0	0	0	1	0	0	14
**2.465**	0	1	1	1	1	0	0	0	1	1	0	1	1	0	0	0	0	0	0	1	0	1	0	1	1	14
**2.466**	0	1	0	0	1	0	0	0	1	1	0	1	0	1	0	0	0	0	0	0	0	0	0	1	1	14
**2.467**	0	1	0	0	0	0	0	0	1	0	0	1	1	0	0	0	0	0	0	1	0	1	0	1	1	14
**2.468**	0	1	0	0	0	0	0	0	1	0	0	1	0	1	0	0	0	0	0	0	0	1	0	1	1	14
**2.469**	0	0	0	1	0	0	0	0	0	0	0	0	0	0	0	0	0	0	0	1	0	1	0	1	1	14
**2.470**	0	0	0	0	1	0	0	0	0	0	0	1	0	0	0	0	0	0	0	1	0	0	1	0	1	14
**2.471**	0	0	0	0	0	0	1	0	1	1	0	1	0	0	0	0	0	0	0	1	0	1	0	0	0	14
**2.472**	0	0	0	0	0	0	0	0	1	1	0	1	0	1	0	0	0	0	0	1	0	0	0	1	0	14
**2.473**	0	0	0	0	0	0	0	0	0	1	0	1	0	0	0	0	0	0	0	1	0	1	1	1	0	14
**2.474**	0	1	0	1	0	0	0	0	0	0	0	1	0	0	0	0	0	0	0	0	0	0	1	1	0	14
**2.475**	0	1	0	0	1	0	0	0	0	1	0	1	1	1	0	0	0	0	0	1	0	1	0	0	0	14
**2.476**	0	1	0	0	0	0	0	0	1	1	0	1	0	1	0	0	0	0	0	0	0	0	1	0	0	14
**2.477**	0	0	0	1	1	0	0	0	0	0	0	1	1	1	0	0	0	0	0	0	0	0	0	0	0	14
**2.478**	0	1	1	1	1	0	0	0	0	1	0	1	0	0	0	0	0	0	0	1	0	0	0	0	1	14
**2.479**	0	1	1	0	1	0	0	0	1	0	0	1	0	0	0	0	0	0	0	1	0	0	0	0	1	14
**2.480**	0	1	1	0	1	0	0	0	0	1	0	1	0	0	0	0	0	0	0	1	0	0	0	1	0	14
**2.481**	0	1	1	0	0	0	1	0	0	0	0	1	0	0	0	0	0	0	0	1	0	0	1	0	0	14
**2.482**	0	1	0	1	0	0	1	0	0	0	0	1	0	0	0	0	0	0	0	1	0	0	1	0	0	14
**2.483**	0	1	0	0	0	0	0	0	0	0	0	1	0	0	0	0	0	0	0	1	0	1	1	1	0	14
**2.484**	0	0	0	0	0	0	1	0	0	0	0	1	0	1	0	0	0	0	0	1	0	0	1	1	1	14
**2.485**	0	1	1	0	1	0	0	0	0	1	0	1	1	0	0	0	0	0	0	0	0	0	0	1	1	14
**2.486**	0	1	1	0	1	0	0	0	0	1	0	1	0	0	0	0	0	0	0	0	0	0	0	1	1	14
**2.487**	0	0	1	1	0	0	1	0	0	1	0	1	0	1	0	1	0	0	0	1	0	0	0	1	1	14
**2.488**	0	0	1	0	0	0	1	0	0	1	0	1	1	1	0	0	0	0	0	1	0	1	0	0	0	14
**2.489**	0	0	0	0	0	0	1	0	1	0	0	1	0	1	0	0	0	0	0	0	0	1	0	1	1	14
**2.490**	0	0	0	0	0	0	0	0	0	0	0	0	1	1	0	1	0	0	0	0	0	0	0	0	0	14
**2.491**	0	1	0	1	1	0	1	0	1	1	0	1	0	0	0	0	0	0	0	0	0	0	0	0	0	14
**2.492**	0	1	0	1	1	0	0	0	0	1	1	1	0	1	0	0	0	0	0	0	0	1	0	1	1	14
**2.493**	0	1	0	1	0	0	0	0	1	1	0	1	0	0	0	0	0	0	0	1	0	0	0	0	1	14
**2.494**	0	1	0	0	0	0	0	0	1	1	0	1	0	0	0	0	0	0	0	1	0	0	0	1	1	14
**2.495**	0	0	0	0	0	0	1	0	1	1	0	1	0	1	0	0	0	0	0	1	0	1	1	1	1	14
**2.496**	0	0	0	0	0	0	0	0	0	1	0	0	0	0	0	1	0	0	0	0	0	1	0	0	0	14
**2.497**	0	1	0	1	1	0	0	0	0	0	0	0	1	0	0	0	0	0	0	0	0	0	0	1	0	14
**2.498**	0	0	1	0	0	0	1	0	1	0	0	0	0	1	0	0	0	0	0	0	0	1	1	0	0	14
**2.499**	0	0	1	0	0	0	1	0	0	0	0	1	0	1	0	0	0	0	0	0	0	1	0	0	1	14
**2.500**	0	0	0	1	1	0	0	0	1	0	0	1	0	0	0	0	0	0	0	0	0	0	0	0	1	14
**2.501**	0	0	0	0	0	0	1	0	0	0	1	1	0	1	0	0	0	0	0	0	0	0	0	1	1	14
**2.502**	0	0	0	0	0	0	0	0	0	1	1	1	0	1	0	0	0	0	0	1	0	1	0	0	0	14
**2.503**	0	1	1	1	0	0	0	0	0	1	0	1	0	0	0	0	0	0	0	0	0	0	0	0	0	14
**2.504**	0	1	0	1	1	0	0	0	0	0	0	0	0	0	0	0	0	0	0	1	0	1	1	0	0	14
**2.505**	0	0	1	0	0	0	0	0	1	0	0	0	0	1	0	0	0	0	0	0	0	0	0	0	0	14
**2.506**	0	0	0	1	0	0	0	0	1	0	0	1	0	0	0	0	0	0	0	0	0	0	0	1	1	14
**2.507**	0	0	0	0	0	0	1	0	0	0	0	1	0	1	0	0	0	0	0	1	0	1	1	0	1	14
**2.508**	0	0	0	0	0	0	0	0	1	0	0	0	1	0	0	1	0	0	0	0	0	0	1	0	0	14
**2.509**	0	0	1	0	0	0	1	0	0	1	0	1	0	1	0	0	0	0	0	0	0	0	0	0	1	14
**2.510**	0	0	0	0	0	0	1	0	0	1	0	1	0	1	0	0	0	0	0	1	0	0	1	1	0	14
**2.511**	0	0	0	0	0	0	1	0	0	1	0	1	0	0	0	0	0	0	0	1	0	0	1	0	0	14
**2.512**	0	0	0	0	0	0	0	0	0	1	1	1	0	1	0	0	0	0	0	0	0	1	1	0	1	14
**2.513**	0	0	1	0	0	0	0	0	1	1	0	1	0	1	0	0	0	0	0	0	0	1	0	1	1	14
**2.514**	0	0	0	0	0	0	0	0	1	0	1	1	0	0	0	0	0	0	0	0	0	1	0	0	1	14
**2.515**	0	0	0	0	0	0	0	0	0	0	0	0	1	1	0	0	0	0	0	1	0	1	0	0	0	14
**2.516**	0	0	0	1	1	0	1	0	0	1	0	1	0	1	0	0	0	0	0	1	0	1	1	0	0	14
**2.517**	0	0	0	1	0	0	0	0	1	0	0	1	0	0	0	0	0	0	0	1	0	0	0	1	1	14
**2.518**	0	0	0	1	0	0	0	0	0	0	0	1	1	0	0	0	0	0	0	0	0	1	0	0	0	14
**2.519**	0	0	0	0	0	0	0	0	0	1	1	1	0	1	0	0	0	0	0	0	0	0	1	0	1	14
**2.520**	0	1	0	0	1	0	0	0	0	1	0	1	0	1	0	0	0	0	0	0	0	1	1	0	1	14
**2.521**	0	1	0	0	0	0	0	0	1	0	0	1	0	0	0	0	0	0	0	1	0	1	1	0	0	14
**2.522**	0	0	1	0	0	0	1	0	1	0	0	0	0	1	0	0	0	0	0	0	0	0	0	1	1	14
**2.523**	0	0	1	0	0	0	1	0	0	1	0	0	0	0	0	0	0	0	0	0	0	1	0	0	1	14
**2.524**	0	0	0	0	1	0	0	0	0	1	0	1	0	0	0	0	0	0	0	0	0	1	1	0	1	14
**2.525**	0	0	0	0	0	0	0	0	1	0	1	0	0	0	0	0	0	0	0	0	0	1	1	0	1	14
**2.526**	0	0	0	0	0	0	0	0	1	0	0	1	0	1	0	0	0	0	0	1	0	0	0	1	1	14
**2.527**	0	1	0	0	0	0	0	0	1	1	0	1	1	0	0	0	0	0	0	1	0	0	0	0	0	14
**2.528**	0	0	1	0	0	0	0	0	0	1	0	1	0	1	0	0	0	0	0	0	0	0	1	1	0	14
**2.529**	0	1	0	1	0	0	1	0	0	0	0	1	0	0	0	0	0	0	0	0	0	0	0	0	0	14
**2.530**	0	1	0	0	1	0	0	0	0	0	0	0	1	0	0	0	0	0	0	1	0	0	1	0	0	14
**2.531**	0	0	0	1	0	0	0	0	0	0	0	0	1	0	0	0	0	0	0	0	0	0	0	0	0	14
**2.532**	0	0	0	0	1	0	0	0	1	0	0	0	0	0	0	0	0	0	0	1	0	0	1	0	0	14
**2.533**	0	0	0	0	1	0	1	0	0	0	0	0	0	0	0	0	0	0	0	0	0	0	0	0	0	14
**2.534**	0	0	0	1	0	0	1	0	1	0	0	1	0	0	0	0	0	0	0	0	0	0	0	0	0	13
**2.535**	0	0	0	0	1	0	1	0	0	0	0	1	0	0	0	0	0	0	0	1	0	0	0	0	1	13
**2.536**	0	0	0	0	0	0	1	0	1	0	0	0	0	0	0	0	0	0	0	0	0	1	0	1	1	13
**2.537**	0	0	0	0	0	0	0	0	1	1	0	1	0	1	0	0	0	0	0	0	0	0	0	1	0	13
**2.538**	0	1	1	1	1	0	0	0	0	1	0	1	0	1	0	0	0	0	0	1	0	0	0	0	0	13
**2.539**	0	1	0	1	1	0	0	0	1	1	0	1	1	0	0	0	0	0	0	1	0	1	0	1	1	13
**2.540**	0	1	0	1	1	0	0	0	1	0	0	1	0	0	0	0	0	0	0	1	0	0	1	1	1	13
**2.541**	0	1	0	0	1	0	0	0	1	1	0	1	1	0	0	0	0	0	0	1	0	1	0	1	1	13
**2.542**	0	1	0	0	0	0	1	0	1	1	0	1	0	0	0	0	0	0	0	0	0	0	0	1	1	13
**2.543**	0	0	0	1	1	0	0	0	0	1	0	1	0	0	0	0	0	0	0	1	0	1	0	1	1	13
**2.544**	0	0	0	0	1	0	0	0	1	1	0	1	1	0	0	0	0	0	0	1	0	0	1	0	0	13
**2.545**	0	0	0	0	0	0	0	0	1	1	0	0	0	0	0	0	0	0	0	1	0	1	0	1	1	13
**2.546**	0	1	0	1	1	0	0	0	1	1	0	1	0	0	0	0	0	0	0	1	0	0	1	0	1	13
**2.547**	0	1	0	0	1	0	0	0	1	0	0	1	1	0	0	0	0	0	0	1	0	1	1	1	1	13
**2.548**	0	1	0	0	1	0	0	0	1	0	0	1	0	0	0	0	0	0	0	1	0	1	1	1	1	13
**2.549**	0	0	1	0	0	0	0	0	1	1	0	1	0	0	0	0	0	0	0	1	0	1	0	1	0	13
**2.550**	0	0	1	0	0	0	0	0	1	1	0	1	0	0	0	0	0	0	0	0	0	1	0	0	1	13
**2.551**	0	0	0	1	0	0	0	0	0	0	0	0	0	1	0	1	0	0	0	0	0	1	0	0	0	13
**2.552**	0	0	0	0	1	0	0	0	0	1	0	1	1	0	0	0	0	0	0	1	0	0	0	1	1	13
**2.553**	0	0	0	0	1	0	0	0	0	1	0	1	0	0	0	0	0	0	0	1	0	1	1	0	0	13
**2.554**	0	1	1	0	1	0	0	0	1	1	0	1	1	1	0	0	0	0	0	1	0	0	0	0	0	13
**2.555**	0	1	0	1	1	0	0	0	0	0	0	0	0	0	0	0	0	0	0	0	0	0	1	1	1	13
**2.556**	0	1	0	0	1	0	0	0	1	0	0	0	0	0	0	0	0	0	0	1	0	1	1	1	1	13
**2.557**	0	0	1	1	0	0	0	0	0	0	0	0	1	0	0	0	0	0	0	1	0	0	0	0	0	13
**2.558**	0	0	0	0	1	0	0	0	0	1	0	0	0	0	0	0	0	0	0	0	0	0	0	0	1	13
**2.559**	0	0	0	0	1	0	0	0	0	0	0	1	1	0	0	0	0	0	0	1	0	0	0	1	1	13
**2.560**	0	0	1	0	0	0	1	0	0	0	0	0	0	0	0	0	0	0	0	1	0	0	0	1	0	13
**2.561**	0	0	0	1	0	0	0	0	0	0	0	0	1	0	0	1	0	0	0	1	0	1	0	0	0	13

CMP	CD2	CD3	CD4	CD7	CD8	CD10	CD13	CD18	CD26	CD29	CD36	CD44	CD45RA	CD49f	CD54	CD56	CD57	CD58	CD62L	CD71	CD80	Cytokeratin	Histone_H1	HLA-DQ	HLA-DR	Freq. (voxel)
**2.562**	0	0	0	0	0	0	0	0	0	1	0	0	1	0	0	0	0	0	0	1	0	0	0	1	0	13
**2.563**	0	1	0	0	1	0	0	0	0	1	0	1	0	0	0	0	0	0	0	1	0	1	1	0	0	13
**2.564**	0	0	1	0	0	0	1	0	0	1	0	1	0	0	0	0	0	0	0	0	0	0	0	1	0	13
**2.565**	0	0	0	0	0	0	1	0	1	0	0	0	0	0	0	0	0	0	0	0	0	0	1	1	1	13
**2.566**	0	0	0	0	0	0	0	0	0	0	0	0	0	1	0	0	0	0	0	0	0	1	1	1	1	13
**2.567**	0	1	1	1	0	0	0	0	0	0	0	1	0	0	0	0	0	0	0	0	0	0	0	0	0	13
**2.568**	0	1	0	0	0	0	1	0	0	1	0	0	0	0	0	0	0	0	0	1	0	0	1	0	0	13
**2.569**	0	1	0	0	0	0	0	0	0	0	0	1	1	0	0	0	0	0	0	0	0	1	1	1	0	13
**2.570**	0	1	0	0	0	0	0	0	0	0	0	1	1	0	0	0	0	0	0	0	0	1	0	1	1	13
**2.571**	0	1	0	0	0	0	0	0	0	0	0	0	0	1	0	0	0	0	0	1	0	0	0	1	0	13
**2.572**	0	0	0	0	1	0	0	0	1	1	0	1	0	0	0	0	0	0	0	0	0	1	0	1	1	13
**2.573**	0	0	0	0	0	0	1	0	0	1	0	1	1	1	0	0	0	0	0	1	0	1	0	0	0	13
**2.574**	0	0	0	0	0	0	0	0	0	1	0	1	1	1	0	0	0	0	0	0	0	0	0	0	0	13
**2.575**	0	0	0	0	0	0	0	0	0	1	0	0	1	0	0	0	0	0	0	0	0	0	1	0	0	13
**2.576**	0	0	1	1	0	0	1	0	1	1	0	1	0	1	0	0	0	0	0	0	0	0	0	1	1	13
**2.577**	0	0	0	1	1	0	0	0	1	0	0	1	1	0	0	0	0	0	0	0	0	0	0	0	0	13
**2.578**	0	0	0	1	1	0	0	0	1	0	0	1	0	0	0	0	0	0	0	0	0	0	1	0	0	13
**2.579**	0	0	0	0	1	0	0	0	0	1	0	0	0	0	0	0	0	0	0	0	0	0	0	1	1	13
**2.580**	0	1	0	0	0	0	0	0	0	1	1	1	0	1	0	0	0	0	0	0	0	1	0	0	0	13
**2.581**	0	0	1	0	0	0	1	0	0	1	0	0	1	1	0	0	0	0	0	1	0	1	0	0	0	13
**2.582**	0	0	1	0	0	0	1	0	0	1	0	0	0	0	0	0	0	0	0	0	0	0	0	1	0	13
**2.583**	0	0	1	0	0	0	1	0	0	0	0	1	0	1	0	0	0	0	0	0	0	0	0	0	0	13
**2.584**	0	0	0	1	0	0	0	0	0	1	0	1	0	0	0	0	0	0	0	0	0	1	0	0	1	13
**2.585**	0	0	0	0	1	0	0	0	1	0	0	1	0	0	0	0	0	0	0	0	0	0	1	1	0	13
**2.586**	0	0	0	0	0	0	1	0	0	0	0	0	0	1	0	0	0	0	0	0	0	0	1	1	0	13
**2.587**	0	1	1	0	0	0	0	0	0	0	0	1	0	0	0	0	0	0	0	0	0	0	0	1	1	13
**2.588**	0	1	0	0	1	0	0	0	1	0	0	1	0	1	0	0	0	0	0	0	0	0	0	1	1	13
**2.589**	0	0	1	1	1	0	0	0	0	1	0	1	0	0	0	0	0	0	0	0	0	0	0	0	0	13
**2.590**	0	0	0	1	0	0	1	0	1	1	0	1	1	1	0	0	0	0	0	1	0	0	0	1	1	13
**2.591**	0	0	0	1	0	0	0	0	0	0	0	0	1	0	0	1	0	0	0	0	0	0	0	1	0	13
**2.592**	0	0	0	0	1	0	0	0	0	0	0	1	0	0	0	0	0	0	0	1	0	0	1	1	1	13
**2.593**	0	0	0	0	0	0	0	0	1	0	1	0	0	0	0	0	0	0	0	0	0	0	0	0	0	13
**2.594**	0	1	0	1	1	0	0	0	1	0	0	0	0	0	0	0	0	0	0	0	0	1	0	0	0	13
**2.595**	0	1	0	1	0	0	0	0	1	1	0	1	1	0	0	0	0	0	0	1	0	0	1	0	0	13
**2.596**	0	1	0	1	0	0	0	0	1	1	0	1	0	0	0	0	0	0	0	0	0	0	0	1	0	13
**2.597**	0	1	0	0	0	0	0	0	0	0	1	1	0	0	0	0	0	0	0	0	0	0	0	1	0	13
**2.598**	0	0	1	0	0	0	1	0	1	1	1	1	1	1	0	1	0	0	0	1	0	0	0	1	1	13
**2.599**	0	0	0	0	0	0	1	0	0	1	0	0	0	0	0	0	0	0	0	0	0	0	1	1	0	13
**2.600**	0	0	0	0	0	0	0	0	0	1	1	1	0	0	0	0	0	0	0	1	0	0	0	1	0	13
**2.601**	0	1	0	0	0	0	0	0	0	1	0	1	0	1	0	0	0	0	0	0	0	0	0	1	1	13
**2.602**	0	0	1	0	0	0	0	0	1	1	0	1	0	1	0	0	0	0	0	1	0	0	0	1	1	13
**2.603**	0	0	1	0	0	0	0	0	0	0	0	1	0	1	0	0	0	0	0	0	0	1	0	0	1	13
**2.604**	0	0	0	1	0	0	1	0	0	1	0	1	0	1	0	0	0	0	0	0	0	0	0	0	0	13
**2.605**	0	0	0	0	0	0	0	0	1	1	0	1	0	1	0	0	0	0	0	0	0	0	1	0	1	13
**2.606**	0	0	0	0	0	0	0	0	0	1	0	1	0	0	0	1	0	0	0	0	0	0	0	1	1	13
**2.607**	0	1	0	1	1	0	0	0	0	0	0	0	0	1	0	0	0	0	0	0	0	0	1	0	0	13
**2.608**	0	1	0	0	1	0	0	0	0	1	0	1	0	1	0	0	0	0	0	0	0	1	1	0	0	13
**2.609**	0	0	1	0	0	0	0	0	0	0	1	1	0	1	0	0	0	0	0	0	0	0	0	1	1	13
**2.610**	0	0	0	0	1	0	1	0	0	1	0	1	0	0	0	0	0	0	0	1	0	0	0	0	1	13
**2.611**	0	0	0	0	0	0	1	0	1	0	0	0	0	0	0	0	0	0	0	1	0	0	0	0	0	13
**2.612**	0	0	0	0	0	0	0	0	1	1	0	1	0	1	0	0	0	0	0	0	0	1	1	0	0	13
**2.613**	0	1	0	0	1	0	0	0	0	0	0	0	0	0	0	0	0	0	0	1	0	1	0	0	0	13
**2.614**	0	1	0	0	0	0	0	0	1	1	0	1	0	0	0	0	0	0	0	1	0	1	0	0	0	13
**2.615**	0	0	0	1	0	0	0	0	0	0	0	0	1	0	0	1	0	0	0	1	0	0	0	0	0	13
**2.616**	0	0	0	0	0	0	0	0	0	1	1	1	0	0	0	0	0	0	0	0	0	1	0	1	1	13
**2.617**	0	0	0	0	0	0	0	0	0	1	0	1	1	0	0	0	0	0	0	1	0	1	0	0	0	13
**2.618**	0	0	0	0	0	0	0	0	0	0	0	0	1	1	0	1	0	0	0	1	0	0	0	1	0	13
**2.619**	0	0	0	0	0	0	0	0	1	0	0	0	1	0	0	1	0	0	0	0	0	1	0	0	0	13
**2.620**	0	0	0	1	1	0	0	0	0	1	0	1	1	0	0	0	0	0	0	0	0	0	0	1	1	12
**2.621**	0	0	0	0	0	0	1	0	1	0	0	0	0	0	0	0	0	0	0	1	0	1	0	1	1	12
**2.622**	0	1	1	0	1	0	0	0	1	0	0	1	0	0	0	0	0	0	0	1	0	1	0	1	1	12
**2.623**	0	1	1	0	1	0	0	0	0	0	0	1	0	1	0	0	0	0	0	1	0	0	0	0	0	12
**2.624**	0	1	0	0	1	0	1	0	0	0	0	1	1	0	0	0	0	0	0	1	0	0	0	0	1	12
**2.625**	0	1	0	0	1	0	1	0	0	0	0	1	1	0	0	0	0	0	0	0	0	0	0	0	1	12
**2.626**	0	1	0	0	1	0	0	0	1	0	0	1	0	0	0	0	0	0	0	1	0	0	1	1	1	12
**2.627**	0	1	0	0	0	0	1	0	1	1	0	1	0	0	0	0	0	0	0	0	0	0	0	0	0	12
**2.628**	0	0	0	1	0	0	0	0	1	0	0	1	0	1	0	0	0	0	0	1	0	1	0	1	1	12
**2.629**	0	1	0	1	1	0	0	0	1	1	0	1	0	0	0	0	0	0	0	0	0	1	0	1	1	12
**2.630**	0	1	0	1	0	0	0	0	1	0	0	1	0	1	0	0	0	0	0	1	0	1	0	1	1	12
**2.631**	0	1	0	0	1	0	0	0	1	1	0	1	0	0	0	0	0	0	0	1	0	0	1	0	1	12
**2.632**	0	1	0	0	1	0	0	0	1	0	0	1	0	1	0	0	0	0	0	0	0	0	1	0	0	12
**2.633**	0	1	0	0	1	0	0	0	0	0	0	1	0	1	0	0	0	0	0	1	0	0	0	0	1	12
**2.634**	0	0	1	0	1	0	0	0	0	0	0	0	1	0	0	0	0	0	0	1	0	1	0	1	0	12
**2.635**	0	0	1	0	0	0	0	0	0	0	0	1	1	0	0	0	0	0	0	0	0	0	0	1	1	12
**2.636**	0	0	0	0	0	0	0	0	0	1	0	0	1	0	0	0	0	0	0	0	0	0	0	1	1	12
**2.637**	0	1	1	1	1	0	0	0	1	0	0	1	1	0	0	0	0	0	0	1	0	0	0	0	0	12
**2.638**	0	1	1	1	1	0	0	0	0	0	0	1	0	1	0	0	0	0	0	1	0	0	0	0	0	12
**2.639**	0	1	1	0	1	0	1	0	0	1	0	1	1	0	0	0	0	0	0	1	0	0	0	0	0	12
**2.640**	0	1	0	1	0	0	1	0	0	0	0	1	0	0	0	0	0	0	0	0	0	0	0	1	1	12
**2.641**	0	0	1	0	0	0	1	0	0	1	0	1	0	0	0	0	0	0	0	1	0	0	1	1	1	12
**2.642**	0	0	1	0	0	0	1	0	0	1	0	1	0	0	0	0	0	0	0	0	0	0	1	1	1	12
**2.643**	0	0	1	0	0	0	0	0	1	1	0	0	0	0	0	0	0	0	0	1	0	1	0	1	1	12
**2.644**	0	0	0	1	0	0	0	0	0	0	0	0	1	0	0	0	0	0	0	0	0	0	0	1	1	12
**2.645**	0	0	0	0	0	0	0	0	0	0	1	1	0	0	0	0	0	0	0	1	0	1	0	0	1	12
**2.646**	0	1	1	1	1	0	0	0	0	1	0	1	1	1	0	0	0	0	0	1	0	0	0	1	1	12
**2.647**	0	1	1	1	0	0	0	0	0	1	0	1	0	0	0	0	0	0	0	1	0	0	0	1	1	12
**2.648**	0	1	0	1	0	0	0	0	0	0	0	1	0	1	0	0	0	0	0	1	0	0	1	0	0	12
**2.649**	0	1	0	0	0	0	0	0	0	0	0	1	1	0	0	0	0	0	0	1	0	0	1	0	1	12
**2.650**	0	0	1	0	0	0	1	0	0	1	0	0	0	0	0	0	0	0	0	0	0	0	1	1	0	12
**2.651**	0	0	0	0	0	0	1	0	0	1	0	1	0	1	0	0	0	0	0	0	0	1	1	1	1	12
**2.652**	0	0	0	0	0	0	0	0	0	1	0	1	0	0	0	1	0	0	0	0	0	1	0	0	0	12
**2.653**	0	1	1	0	1	0	0	0	0	0	0	1	0	0	0	0	0	0	0	1	0	0	0	0	1	12
**2.654**	0	1	1	0	0	0	0	0	1	1	0	1	0	0	0	0	0	0	0	0	0	0	0	0	0	12
**2.655**	0	1	1	0	0	0	0	0	0	1	0	1	0	0	0	0	0	0	0	1	0	0	0	1	0	12
**2.656**	0	1	0	1	0	0	0	0	0	0	0	1	0	1	0	0	0	0	0	0	0	0	0	1	1	12
**2.657**	0	0	0	0	0	0	1	0	1	0	0	1	0	0	0	0	0	0	0	0	0	0	1	1	1	12
**2.658**	0	1	1	1	1	0	0	0	0	1	0	1	0	0	0	0	0	0	0	1	0	0	0	1	0	12
**2.659**	0	1	0	0	0	0	1	0	0	1	0	1	0	0	0	0	0	0	0	0	0	0	0	0	1	12
**2.660**	0	0	1	0	0	0	0	0	0	0	0	1	1	0	0	0	0	0	0	0	0	0	0	0	0	12
**2.661**	0	0	0	0	0	0	0	0	1	0	0	1	0	0	0	0	0	0	0	0	0	1	1	1	0	12
**2.662**	0	0	0	0	0	0	0	0	1	0	0	0	0	0	0	1	0	0	0	0	0	0	1	1	1	12
**2.663**	0	0	0	0	0	0	0	0	0	0	0	0	0	1	0	1	0	0	0	0	0	0	0	1	0	12
**2.664**	0	1	0	0	1	0	0	0	1	1	0	1	1	1	0	0	0	0	0	0	0	0	0	0	0	12
**2.665**	0	1	0	0	0	0	1	0	0	0	0	0	0	0	0	0	0	0	0	0	0	1	1	0	0	12
**2.666**	0	0	1	1	0	0	1	0	0	1	0	1	0	1	0	0	0	0	0	1	0	1	0	1	1	12
**2.667**	0	0	1	0	0	0	0	0	0	0	0	0	0	1	0	1	0	0	0	0	0	1	0	1	0	12
**2.668**	0	0	0	1	1	0	0	0	0	0	0	1	0	0	0	0	0	0	0	0	0	0	0	1	0	12
**2.669**	0	0	0	0	1	0	0	0	0	0	0	1	1	0	0	0	0	0	0	1	0	1	0	0	1	12
**2.670**	0	0	0	0	0	0	0	0	0	1	0	0	0	1	0	1	0	0	0	0	0	0	0	1	0	12
**2.671**	0	1	0	1	1	0	0	0	1	0	0	1	0	0	0	0	0	0	0	0	0	0	1	1	0	12
**2.672**	0	1	0	0	1	0	1	0	1	1	0	1	1	0	0	0	0	0	0	1	0	0	0	0	0	12
**2.673**	0	1	0	0	1	0	1	0	0	1	0	1	0	1	0	0	0	0	0	0	0	0	0	0	0	12
**2.674**	0	1	0	0	1	0	0	0	1	1	0	1	1	0	0	0	0	0	0	0	0	0	0	1	0	12
**2.675**	0	1	0	0	0	0	0	0	1	0	0	0	0	0	0	0	0	0	0	0	0	0	1	1	0	12
**2.676**	0	1	0	0	0	0	0	0	0	0	1	1	0	0	0	0	0	0	0	0	0	0	0	1	1	12
**2.677**	0	0	1	0	0	0	0	0	0	1	0	1	1	1	0	0	0	0	0	0	0	0	0	1	1	12
**2.678**	0	0	1	0	0	0	0	0	0	0	0	0	0	1	0	0	0	0	0	0	0	1	0	0	1	12
**2.679**	0	1	0	0	1	0	0	0	0	1	1	1	0	1	0	0	0	0	0	0	0	0	1	1	0	12
**2.680**	0	1	0	0	1	0	0	0	1	1	0	1	0	1	0	0	0	0	0	1	0	0	0	0	0	12
**2.681**	0	1	0	0	0	0	0	0	0	0	0	1	0	1	0	0	0	0	0	0	0	1	0	0	1	12
**2.682**	0	0	1	0	0	0	1	0	0	1	1	1	1	1	0	0	0	0	0	0	0	0	0	1	1	12
**2.683**	0	0	0	0	0	0	0	0	0	1	0	0	0	1	0	0	0	0	0	1	0	1	1	0	1	12

CMP	CD2	CD3	CD4	CD7	CD8	CD10	CD13	CD18	CD26	CD29	CD36	CD44	CD45RA	CD49f	CD54	CD56	CD57	CD58	CD62L	CD71	CD80	Cytokeratin	Histone_H1	HLA-DQ	HLA-DR	Freq. (voxel)
**2.684**	0	0	0	1	1	0	0	0	0	0	0	1	0	0	0	0	0	0	0	0	0	0	1	0	1	12
**2.685**	0	0	0	0	0	0	1	0	1	1	0	1	0	1	0	0	0	0	0	1	0	1	1	0	1	12
**2.686**	0	0	0	0	0	0	1	0	0	0	1	0	0	1	0	0	0	0	0	0	0	1	0	0	1	12
**2.687**	0	0	0	0	0	0	1	0	0	0	0	1	0	1	0	0	0	0	0	0	0	1	0	1	0	12
**2.688**	0	0	0	0	0	0	0	0	0	0	0	0	1	0	0	0	0	0	0	0	0	1	0	1	1	12
**2.689**	0	1	0	0	1	0	0	0	0	0	0	0	1	0	0	0	0	0	0	1	0	1	0	0	0	12
**2.690**	0	0	0	0	0	0	0	0	1	0	1	1	0	0	0	0	0	0	0	0	0	0	0	1	1	12
**2.691**	0	0	0	0	0	0	0	0	0	1	0	0	0	1	0	0	0	0	0	1	0	0	0	0	1	12
**2.692**	0	1	0	1	1	0	0	0	0	0	0	1	0	0	0	0	0	0	0	0	0	1	0	1	1	12
**2.693**	0	1	0	1	0	0	0	0	0	0	0	1	1	0	0	0	0	0	0	0	0	1	1	0	0	12
**2.694**	0	0	0	0	0	0	1	0	0	0	0	0	1	0	0	0	0	0	0	1	0	0	1	0	0	12
**2.695**	0	0	0	0	0	0	1	0	0	0	0	0	1	0	0	1	0	0	0	1	0	0	1	0	0	12
**2.696**	0	0	0	1	1	0	0	0	0	0	0	0	0	0	0	0	0	0	0	0	0	1	0	0	0	12
**2.697**	0	0	0	0	1	0	0	0	0	0	0	0	0	1	0	0	0	0	0	0	0	1	0	0	0	12
**2.698**	0	0	1	0	0	0	0	0	0	0	0	0	0	0	0	0	0	0	0	1	0	0	1	1	1	12
**2.699**	0	0	0	1	1	0	1	0	0	0	0	1	0	0	0	0	0	0	0	1	0	0	0	0	1	11
**2.700**	0	0	0	1	1	0	0	0	0	0	0	1	1	0	0	0	0	0	0	0	0	1	0	1	1	11
**2.701**	0	1	1	1	1	0	1	0	0	0	0	1	0	0	0	0	0	0	0	1	0	0	0	1	1	11
**2.702**	0	1	0	1	1	0	0	0	0	0	0	1	1	1	0	0	0	0	0	1	0	0	0	1	1	11
**2.703**	0	1	0	1	0	0	0	0	1	0	0	1	0	0	0	0	0	0	0	0	0	1	1	0	0	11
**2.704**	0	0	1	0	0	0	0	0	1	1	0	1	0	0	0	0	0	0	0	0	0	1	0	1	0	11
**2.705**	0	0	0	0	1	0	0	0	1	0	0	1	0	0	0	0	0	0	0	0	0	0	1	1	1	11
**2.706**	0	0	0	0	1	0	0	0	0	1	0	1	0	1	0	0	0	0	0	1	0	1	0	1	0	11
**2.707**	0	0	0	0	1	0	0	0	0	0	0	1	0	0	0	0	0	0	0	1	0	1	1	1	1	11
**2.708**	0	0	0	0	0	0	0	0	1	0	0	1	1	1	0	0	0	0	0	0	0	1	0	0	0	11
**2.709**	0	1	0	1	1	0	0	0	0	1	0	1	1	0	0	0	0	0	0	1	0	1	0	1	0	11
**2.710**	0	1	0	0	1	0	0	0	0	1	0	1	0	0	0	0	0	0	0	1	0	1	0	1	1	11
**2.711**	0	1	0	0	0	0	0	0	1	1	0	1	0	0	0	0	0	0	0	1	0	1	0	1	1	11
**2.712**	0	1	0	0	0	0	0	0	1	0	0	1	0	1	0	0	0	0	0	1	0	1	0	1	1	11
**2.713**	0	0	0	0	1	0	0	0	1	0	0	0	0	0	0	0	0	0	0	1	0	0	0	0	1	11
**2.714**	0	0	0	0	0	0	0	0	1	0	0	0	1	0	0	0	0	0	0	0	0	0	0	1	1	11
**2.715**	0	0	0	0	0	0	0	0	1	0	0	0	0	1	0	0	0	0	0	1	0	1	0	1	0	11
**2.716**	0	1	1	1	1	0	0	0	1	1	0	1	1	1	0	0	0	0	0	1	0	0	0	1	0	11
**2.717**	0	1	0	1	1	0	0	0	1	1	0	1	1	1	0	0	0	0	0	1	0	0	0	0	1	11
**2.718**	0	1	0	1	0	0	0	0	0	1	0	1	0	1	0	0	0	0	0	1	0	0	1	0	0	11
**2.719**	0	0	1	1	0	0	0	0	0	0	0	0	1	0	0	0	0	0	0	1	0	0	0	1	0	11
**2.720**	0	0	1	0	0	0	0	0	0	0	0	1	1	0	0	0	0	0	0	1	0	1	0	0	0	11
**2.721**	0	0	0	0	1	0	0	0	1	1	0	1	0	1	0	0	0	0	0	1	0	0	0	1	0	11
**2.722**	0	1	1	0	1	0	1	0	0	0	0	1	0	0	0	0	0	0	0	0	0	0	0	1	1	11
**2.723**	0	1	1	0	0	0	0	0	0	0	0	1	0	0	0	0	0	0	0	0	0	1	1	1	1	11
**2.724**	0	1	0	1	0	0	0	0	0	0	0	1	0	0	0	0	0	0	0	1	0	1	1	1	1	11
**2.725**	0	1	0	0	1	0	0	0	0	0	0	1	0	1	0	0	0	0	0	1	0	1	0	1	1	11
**2.726**	0	0	0	0	0	0	1	0	0	1	0	0	1	1	0	0	0	0	0	0	0	0	0	0	0	11
**2.727**	0	1	1	0	1	0	0	0	0	1	0	1	0	1	0	0	0	0	0	1	0	0	0	1	1	11
**2.728**	0	1	1	0	0	0	0	0	0	0	0	1	0	0	0	0	0	0	0	1	0	0	0	1	1	11
**2.729**	0	1	1	0	0	0	0	0	0	0	0	1	0	0	0	0	0	0	0	1	0	0	0	0	1	11
**2.730**	0	1	0	1	0	0	0	0	0	0	0	1	0	0	0	0	0	0	0	1	0	0	1	1	1	11
**2.731**	0	1	0	0	0	0	0	0	1	1	0	1	1	0	0	0	0	0	0	0	0	0	1	0	0	11
**2.732**	0	1	0	0	0	0	0	0	0	1	0	1	0	1	0	0	0	0	0	1	0	0	0	1	1	11
**2.733**	0	1	0	0	0	0	0	0	0	0	0	0	0	1	0	0	0	0	0	1	0	1	0	1	1	11
**2.734**	0	0	1	0	0	0	1	0	0	0	0	0	1	0	0	0	0	0	0	1	0	0	0	1	0	11
**2.735**	0	0	1	0	0	0	0	0	0	1	0	0	0	0	0	0	0	0	0	0	0	0	1	1	0	11
**2.736**	0	0	0	1	0	0	1	0	1	0	0	0	0	1	0	0	0	0	0	0	0	1	0	0	0	11
**2.737**	0	0	0	1	0	0	0	0	0	0	0	1	0	1	0	0	0	0	0	0	0	1	0	1	1	11
**2.738**	0	0	0	0	0	0	1	0	1	0	0	0	0	1	0	0	0	0	0	1	0	1	0	0	1	11
**2.739**	0	0	0	0	0	0	1	0	1	0	0	0	0	1	0	0	0	0	0	0	0	0	1	1	1	11
**2.740**	0	1	0	1	1	0	0	0	0	0	0	1	1	0	0	0	0	0	0	1	0	1	0	1	0	11
**2.741**	0	1	0	1	0	0	0	0	0	0	0	1	0	0	0	0	0	0	0	0	0	1	1	0	1	11
**2.742**	0	1	0	0	1	0	0	0	0	1	0	1	1	1	0	0	0	0	0	0	0	1	0	0	0	11
**2.743**	0	1	0	0	1	0	0	0	0	1	0	0	0	0	0	0	0	0	0	0	0	0	0	1	1	11
**2.744**	0	1	0	0	1	0	0	0	0	0	0	1	1	0	0	0	0	0	0	1	0	0	1	1	1	11
**2.745**	0	1	0	0	0	0	0	0	0	0	0	0	0	0	0	0	0	0	0	1	0	0	0	1	0	11
**2.746**	0	0	1	0	0	0	0	0	0	0	1	0	0	0	0	0	0	0	0	0	0	1	0	1	1	11
**2.747**	0	0	0	0	0	0	0	0	0	0	0	0	1	1	0	0	0	0	0	0	0	0	0	1	0	11
**2.748**	0	1	0	0	1	0	0	0	0	1	0	0	0	0	0	0	0	0	0	0	0	0	1	1	1	11
**2.749**	0	1	0	0	0	0	0	0	0	0	0	0	0	1	0	0	0	0	0	1	0	0	1	0	1	11
**2.750**	0	0	1	1	0	0	1	0	0	1	0	1	1	1	0	0	0	0	0	0	0	0	0	1	1	11
**2.751**	0	0	1	0	0	0	1	0	0	0	0	1	0	1	0	0	0	0	0	1	0	1	0	0	1	11
**2.752**	0	0	0	0	1	0	1	0	0	1	0	1	0	1	0	0	0	0	0	0	0	1	1	1	1	11
**2.753**	0	0	0	0	0	0	1	0	0	0	0	1	1	0	0	0	0	0	0	0	0	0	1	0	0	11
**2.754**	0	1	0	1	1	0	0	0	0	1	0	1	0	1	0	0	0	0	0	0	0	0	1	1	1	11
**2.755**	0	1	0	1	0	0	0	0	0	0	0	0	0	0	0	0	0	0	0	0	0	0	1	1	1	11
**2.756**	0	1	0	0	1	0	0	0	1	1	0	1	0	0	0	0	0	0	0	0	0	1	1	1	1	11
**2.757**	0	1	0	0	1	0	0	0	1	0	0	1	0	0	0	0	0	0	0	0	0	1	1	1	1	11
**2.758**	0	1	0	0	1	0	0	0	0	1	0	1	0	1	0	0	0	0	0	1	0	0	0	1	1	11
**2.759**	0	1	0	0	0	0	0	0	1	0	0	0	0	0	0	0	0	0	0	0	0	0	1	0	1	11
**2.760**	0	1	0	0	0	0	0	0	0	0	0	1	0	1	0	0	0	0	0	0	0	1	0	1	0	11
**2.761**	0	0	0	0	1	0	0	0	1	0	0	1	0	0	0	0	0	0	0	0	0	1	1	0	0	11
**2.762**	0	0	0	0	0	0	0	0	1	0	1	0	0	0	0	0	0	0	0	0	0	1	0	1	0	11
**2.763**	0	1	0	1	1	0	0	0	0	1	0	0	0	0	0	0	0	0	0	1	0	0	1	0	0	11
**2.764**	0	0	1	0	0	0	1	0	1	1	0	1	0	1	0	0	0	0	0	1	0	1	1	1	1	11
**2.765**	0	0	1	0	0	0	1	0	1	1	0	1	0	1	0	0	0	0	0	1	0	0	1	1	1	11
**2.766**	0	0	1	0	0	0	1	0	0	1	0	0	0	1	0	0	0	0	0	0	0	1	1	0	0	11
**2.767**	0	0	0	1	0	0	0	0	1	0	0	0	0	1	0	0	0	0	0	0	0	1	0	0	1	11
**2.768**	0	0	0	0	0	0	0	0	0	0	0	1	1	0	0	0	0	0	0	1	0	1	0	0	1	11
**2.769**	0	1	1	0	1	0	0	0	1	1	0	1	0	0	0	0	0	0	0	0	0	0	0	1	0	11
**2.770**	0	1	0	1	0	0	0	0	1	0	0	1	1	0	0	0	0	0	0	1	0	0	1	0	0	11
**2.771**	0	1	0	0	1	0	0	0	0	1	0	1	0	1	0	0	0	0	0	0	0	1	0	0	1	11
**2.772**	0	1	0	0	0	0	0	0	0	0	1	0	0	0	0	0	0	0	0	0	0	1	0	1	1	11
**2.773**	0	1	0	0	0	0	0	0	0	0	0	0	1	0	0	0	0	0	0	1	0	0	1	0	1	11
**2.774**	0	0	0	1	1	0	0	0	0	0	0	1	1	0	0	0	0	0	0	0	0	0	0	0	1	11
**2.775**	0	0	0	0	0	0	1	0	1	1	0	0	0	1	0	0	0	0	0	1	0	0	0	1	1	11
**2.776**	0	0	0	0	0	0	1	0	1	1	0	0	0	1	0	0	0	0	0	1	0	0	0	0	0	11
**2.777**	0	0	0	0	0	0	1	0	1	1	0	0	0	0	0	0	0	0	0	0	0	0	0	1	0	11
**2.778**	0	1	1	0	0	0	0	0	1	0	0	1	0	0	0	0	0	0	0	1	0	1	0	0	0	11
**2.779**	0	0	1	0	0	0	0	0	1	1	0	0	0	0	0	0	0	0	0	1	0	0	0	1	1	11
**2.780**	0	0	1	0	0	0	0	0	0	1	0	0	0	0	0	0	0	0	0	1	0	0	0	0	1	11
**2.781**	0	0	1	0	0	0	0	0	0	0	1	0	0	0	0	0	0	0	0	1	0	1	0	0	0	11
**2.782**	0	0	0	0	0	0	1	0	1	1	0	0	0	0	0	0	0	0	0	1	0	0	0	0	0	11
**2.783**	0	0	0	0	0	0	1	0	1	0	0	0	0	1	0	0	0	0	0	1	0	0	0	1	1	11
**2.784**	0	0	0	0	0	0	1	0	0	1	0	1	1	1	0	0	0	0	0	1	0	0	1	0	0	11
**2.785**	0	1	0	0	0	0	0	0	0	0	1	1	0	0	0	0	0	0	0	0	0	0	0	0	0	11
**2.786**	0	1	0	0	0	0	0	0	0	0	0	1	1	0	0	0	0	0	0	1	0	1	0	0	0	11
**2.787**	0	0	0	0	0	0	1	0	1	1	0	0	0	1	0	0	0	0	0	0	0	1	0	0	1	11
**2.788**	0	0	0	0	0	0	1	0	1	0	0	0	0	1	0	0	0	0	0	0	0	0	0	0	0	11
**2.789**	0	0	1	0	0	0	0	0	1	0	0	0	0	0	0	0	0	0	0	0	0	1	1	0	0	11
**2.790**	0	0	1	0	0	0	0	0	0	1	1	1	0	0	0	0	0	0	0	0	0	0	0	0	1	11
**2.791**	0	1	0	0	1	0	0	0	0	0	0	1	1	1	0	0	0	0	0	1	0	0	0	0	0	11
**2.792**	0	1	0	0	1	0	0	0	0	0	0	1	0	0	0	1	0	0	0	0	0	0	0	0	0	11
**2.793**	0	0	0	0	1	0	1	0	1	0	0	0	1	0	0	1	0	0	0	1	0	0	0	1	0	11
**2.794**	0	1	0	0	1	0	1	0	0	1	0	1	1	0	0	0	0	0	0	0	0	0	0	0	0	11
**2.795**	0	1	0	0	0	0	0	0	1	0	0	1	1	0	0	0	0	0	0	0	0	1	0	0	0	11
**2.796**	0	0	1	0	0	0	0	0	1	0	0	0	1	0	0	0	0	0	0	0	0	1	0	0	0	11
**2.797**	0	0	0	1	0	0	0	0	1	0	0	0	0	0	0	0	0	0	0	1	0	0	1	0	0	11
**2.798**	0	0	0	0	1	0	0	0	0	0	1	1	0	0	0	0	0	0	0	0	0	0	0	0	0	11
**2.799**	0	1	0	1	1	0	1	0	1	1	0	1	0	0	0	0	0	0	0	1	0	0	0	1	1	10
**2.800**	0	0	0	1	1	0	0	0	1	0	0	1	1	0	0	0	0	0	0	0	0	0	0	1	1	10
**2.801**	0	0	0	1	1	0	0	0	0	0	0	1	0	0	0	0	0	0	0	0	0	0	1	1	1	10
**2.802**	0	1	0	1	1	0	0	0	1	1	0	1	0	0	0	1	0	0	0	0	0	0	0	0	0	10
**2.803**	0	1	0	1	1	0	0	0	0	0	0	1	0	0	0	0	0	0	0	0	0	1	1	1	1	10
**2.804**	0	1	0	0	1	0	0	0	1	0	0	1	0	0	0	0	0	0	0	1	0	1	1	0	1	10
**2.805**	0	0	0	1	0	0	1	0	0	0	0	1	0	0	0	0	0	0	0	1	0	0	0	1	1	10

CMP	CD2	CD3	CD4	CD7	CD8	CD10	CD13	CD18	CD26	CD29	CD36	CD44	CD45RA	CD49f	CD54	CD56	CD57	CD58	CD62L	CD71	CD80	Cytokeratin	Histone_H1	HLA-DQ	HLA-DR	Freq. (voxel)
**2.806**	0	0	0	1	0	0	0	0	0	0	0	0	1	0	0	0	0	0	0	1	0	0	1	0	0	10
**2.807**	0	0	0	0	1	0	0	0	1	0	0	1	1	0	0	0	0	0	0	0	0	1	0	0	1	10
**2.808**	0	0	0	0	1	0	0	0	1	0	0	1	0	0	0	0	0	0	0	1	0	0	1	0	0	10
**2.809**	0	1	1	0	0	0	0	0	1	0	0	1	0	0	0	0	0	0	0	1	0	0	0	0	0	10
**2.810**	0	1	0	1	1	0	0	0	1	0	0	1	0	0	0	0	0	0	0	0	0	0	1	1	1	10
**2.811**	0	1	0	0	1	0	0	0	1	0	0	1	1	1	0	0	0	0	0	0	0	0	0	1	1	10
**2.812**	0	0	1	0	1	0	0	0	1	1	0	1	0	0	0	0	0	0	0	1	0	0	0	0	0	10
**2.813**	0	0	0	0	1	0	0	0	0	1	0	1	1	1	0	0	0	0	0	1	0	0	0	0	0	10
**2.814**	0	1	1	0	0	0	0	0	1	1	0	1	1	0	0	0	0	0	0	1	0	0	1	0	0	10
**2.815**	0	1	1	0	0	0	0	0	1	1	0	1	1	0	0	0	0	0	0	1	0	0	0	0	0	10
**2.816**	0	1	1	0	0	0	0	0	0	0	0	1	0	0	0	0	0	0	0	1	0	1	1	1	1	10
**2.817**	0	1	0	1	0	0	0	0	0	0	0	1	1	1	0	0	0	0	0	1	0	1	0	1	0	10
**2.818**	0	1	0	0	1	0	0	0	1	1	0	1	1	0	0	0	0	0	0	1	0	0	1	1	1	10
**2.819**	0	1	0	0	1	0	0	0	0	1	0	1	0	0	0	0	0	0	0	0	0	1	1	1	1	10
**2.820**	0	1	0	0	0	0	0	0	1	0	0	0	0	0	0	0	0	0	0	0	0	1	1	1	1	10
**2.821**	0	0	0	1	0	0	0	0	1	0	0	1	0	1	0	0	0	0	0	0	0	0	0	0	0	10
**2.822**	0	0	0	1	0	0	0	0	0	0	0	1	0	1	0	0	0	0	0	1	0	0	0	1	1	10
**2.823**	0	0	0	0	1	0	1	0	1	1	0	1	0	1	0	0	0	0	0	1	0	1	0	0	1	10
**2.824**	0	0	0	0	1	0	0	0	0	1	0	1	0	1	0	0	0	0	0	1	0	0	0	0	1	10
**2.825**	0	0	0	0	0	0	0	0	0	1	0	0	1	0	0	0	0	0	0	1	0	0	1	0	0	10
**2.826**	0	1	1	1	1	0	0	0	1	0	0	1	0	0	0	0	0	0	0	1	0	0	0	1	1	10
**2.827**	0	1	1	0	1	0	0	0	0	1	0	1	1	0	0	0	0	0	0	1	0	0	0	1	1	10
**2.828**	0	1	0	0	1	0	0	0	0	1	0	1	1	0	0	0	0	0	0	0	0	1	0	1	0	10
**2.829**	0	0	1	0	0	0	0	0	1	0	0	1	0	0	0	0	0	0	0	1	0	0	0	0	1	10
**2.830**	0	0	0	1	1	0	0	0	0	1	0	1	1	1	0	0	0	0	0	0	0	0	0	0	0	10
**2.831**	0	0	0	0	0	0	0	0	1	1	0	1	1	0	0	0	0	0	0	0	0	0	1	0	0	10
**2.832**	0	1	1	0	1	0	0	0	1	1	0	1	1	0	0	0	0	0	0	0	0	0	0	0	0	10
**2.833**	0	1	1	0	1	0	0	0	0	0	0	1	0	0	0	0	0	0	0	0	0	0	0	0	1	10
**2.834**	0	1	0	1	1	0	1	0	0	0	0	1	0	0	0	0	0	0	0	0	0	0	1	0	1	10
**2.835**	0	1	0	1	1	0	0	0	0	1	0	1	1	0	0	0	0	0	0	0	0	0	1	1	1	10
**2.836**	0	1	0	1	1	0	0	0	0	1	0	1	0	1	0	0	0	0	0	0	0	1	1	1	1	10
**2.837**	0	1	0	1	0	0	0	0	0	1	0	1	0	1	0	0	0	0	0	0	0	0	0	1	1	10
**2.838**	0	0	1	1	0	0	0	0	0	0	0	1	0	0	0	0	0	0	0	0	0	0	0	0	0	10
**2.839**	0	0	1	0	1	0	1	0	0	0	0	0	0	0	0	0	0	0	0	0	0	0	0	1	1	10
**2.840**	0	0	1	0	0	0	1	0	1	0	0	0	0	1	0	0	0	0	0	1	0	1	0	0	0	10
**2.841**	0	0	1	0	0	0	0	0	0	0	0	0	0	1	0	0	0	0	0	1	0	1	0	1	1	10
**2.842**	0	0	0	0	0	0	0	0	1	1	0	1	1	0	0	0	0	0	0	0	0	1	1	0	0	10
**2.843**	0	1	0	1	0	0	0	0	1	1	0	1	0	0	0	0	0	0	0	0	0	0	0	0	1	10
**2.844**	0	1	0	0	0	0	0	0	1	1	0	1	1	1	0	0	0	0	0	0	0	0	0	0	0	10
**2.845**	0	1	0	0	0	0	0	0	0	1	0	1	1	0	0	0	0	0	0	0	0	0	0	1	0	10
**2.846**	0	0	1	0	0	0	1	0	0	0	0	0	1	0	0	0	0	0	0	0	0	1	0	0	0	10
**2.847**	0	0	1	0	0	0	0	0	0	0	0	1	1	1	0	0	0	0	0	1	0	1	0	1	1	10
**2.848**	0	0	0	1	1	0	1	0	0	0	0	1	0	0	0	0	0	0	0	0	0	0	1	0	0	10
**2.849**	0	1	1	1	1	0	0	0	1	1	0	1	0	0	0	0	0	0	0	1	0	0	0	1	0	10
**2.850**	0	1	0	0	1	0	0	0	0	0	0	1	1	0	0	0	0	0	0	1	0	0	0	1	0	10
**2.851**	0	1	0	0	0	0	0	0	1	0	0	1	0	0	0	0	0	0	0	0	0	1	1	1	0	10
**2.852**	0	1	0	0	0	0	0	0	0	0	0	1	1	0	0	0	0	0	0	0	0	0	1	1	0	10
**2.853**	0	0	1	1	0	0	0	0	1	0	0	1	0	1	0	0	0	0	0	1	0	1	0	1	1	10
**2.854**	0	0	1	1	0	0	0	0	0	1	0	1	0	1	0	0	0	0	0	0	0	0	0	1	1	10
**2.855**	0	0	1	0	0	0	1	0	1	0	0	0	0	0	0	0	0	0	0	1	0	1	0	0	0	10
**2.856**	0	0	1	0	0	0	0	0	0	1	0	1	1	0	0	0	0	0	0	0	0	0	0	0	0	10
**2.857**	0	0	0	0	1	0	0	0	0	0	0	1	1	0	0	0	0	0	0	0	0	1	1	0	0	10
**2.858**	0	0	0	0	0	0	0	0	1	0	0	1	0	1	0	0	0	0	0	1	0	0	0	0	1	10
**2.859**	0	0	0	0	0	0	0	0	0	0	0	0	0	0	0	1	0	0	0	0	0	0	1	0	1	10
**2.860**	0	1	1	1	1	0	0	0	0	0	0	1	1	0	0	0	0	0	0	1	0	0	0	0	0	10
**2.861**	0	1	0	1	1	0	0	0	0	1	1	1	0	1	0	0	0	0	0	0	0	1	1	1	1	10
**2.862**	0	0	1	0	0	0	0	0	0	0	0	0	0	1	0	0	0	0	0	0	0	0	0	0	1	10
**2.863**	0	0	0	0	1	0	0	0	1	0	0	1	1	0	0	0	0	0	0	0	0	0	0	1	1	10
**2.864**	0	0	0	0	1	0	0	0	0	0	0	0	0	0	0	0	0	0	0	0	0	1	0	1	1	10
**2.865**	0	0	0	0	0	0	1	0	1	1	0	0	0	1	0	0	0	0	0	0	0	1	1	0	0	10
**2.866**	0	0	0	0	0	0	1	0	0	1	0	1	0	1	0	1	0	0	0	0	0	0	0	1	1	10
**2.867**	0	0	0	0	0	0	0	0	0	1	0	0	0	1	0	0	0	0	0	1	0	1	0	1	1	10
**2.868**	0	0	0	0	0	0	0	0	0	1	0	0	0	0	0	1	0	0	0	0	0	0	0	0	0	10
**2.869**	0	0	0	0	0	0	0	0	0	0	0	1	0	1	0	0	0	0	0	0	0	1	1	1	0	10
**2.870**	0	1	0	1	0	0	0	0	0	0	0	1	0	0	0	0	0	0	0	0	0	1	1	1	0	10
**2.871**	0	1	0	0	1	0	0	0	1	0	0	0	0	0	0	0	0	0	0	1	0	0	1	0	1	10
**2.872**	0	1	0	0	0	0	0	0	0	0	0	0	1	0	0	0	0	0	0	0	0	1	1	0	0	10
**2.873**	0	0	1	1	0	0	0	0	0	1	0	1	0	1	0	0	0	0	0	0	0	1	0	0	0	10
**2.874**	0	0	0	0	1	0	0	0	0	1	0	1	0	1	0	0	0	0	0	0	0	0	1	1	1	10
**2.875**	0	0	0	0	0	0	1	0	0	1	0	1	0	1	0	1	0	0	0	0	0	0	1	1	1	10
**2.876**	0	0	0	0	0	0	1	0	0	0	1	1	0	0	0	0	0	0	0	0	0	1	0	0	0	10
**2.877**	0	0	0	0	0	0	0	0	0	1	0	1	0	1	0	1	0	0	0	0	0	1	0	0	0	10
**2.878**	0	1	0	1	1	0	0	0	1	0	0	1	0	0	0	0	0	0	0	0	0	1	1	0	0	10
**2.879**	0	1	0	0	1	0	0	0	1	1	0	1	0	1	0	0	0	0	0	0	0	0	0	0	0	10
**2.880**	0	1	0	0	0	0	0	0	1	0	0	0	0	0	0	0	0	0	0	0	0	1	1	0	0	10
**2.881**	0	1	0	0	0	0	0	0	0	0	0	0	0	1	0	0	0	0	0	0	0	1	0	0	0	10
**2.882**	0	0	1	0	0	0	1	0	0	1	1	1	0	1	0	1	0	0	0	0	0	0	0	1	1	10
**2.883**	0	0	1	0	0	0	0	0	0	0	0	1	1	1	0	0	0	0	0	0	0	1	0	0	0	10
**2.884**	0	0	0	1	1	0	0	0	1	0	0	0	0	0	0	0	0	0	0	0	0	1	0	0	1	10
**2.885**	0	0	0	0	1	0	0	0	0	0	0	1	0	0	0	1	0	0	0	0	0	0	0	0	1	10
**2.886**	0	0	0	0	0	0	1	0	1	1	0	1	0	0	0	0	0	0	0	0	0	1	0	0	1	10
**2.887**	0	0	0	0	0	0	1	0	0	0	1	1	0	1	0	0	0	0	0	0	0	0	0	0	1	10
**2.888**	0	1	0	1	1	0	0	0	1	0	0	0	1	0	0	0	0	0	0	0	0	0	0	0	0	10
**2.889**	0	1	0	1	1	0	0	0	0	0	0	0	1	0	0	0	0	0	0	1	0	0	0	0	0	10
**2.890**	0	0	1	0	0	0	0	0	1	0	0	1	0	1	0	0	0	0	0	1	0	0	0	1	1	10
**2.891**	0	0	0	1	0	0	0	0	1	0	1	0	0	0	0	0	0	0	0	0	0	1	0	0	0	10
**2.892**	0	0	0	0	0	0	1	0	1	0	0	1	0	0	0	0	0	0	0	1	0	0	0	0	0	10
**2.893**	0	0	0	0	0	0	1	0	1	0	0	0	0	1	0	0	0	0	0	1	0	0	0	0	0	10
**2.894**	0	1	0	1	0	0	0	0	0	0	0	0	1	0	0	0	0	0	0	1	0	0	0	0	0	10
**2.895**	0	1	0	0	1	0	0	0	1	0	0	1	1	0	0	0	0	0	0	1	0	1	0	0	1	10
**2.896**	0	1	0	0	0	0	0	0	1	0	0	0	0	0	0	0	0	0	0	0	0	0	0	0	1	10
**2.897**	0	0	0	0	0	0	0	0	1	1	0	1	0	1	0	0	0	0	0	0	0	1	1	0	1	10
**2.898**	0	0	0	0	0	0	0	0	1	1	0	0	0	0	0	0	0	0	0	1	0	0	0	1	0	10
**2.899**	0	0	0	0	0	0	0	0	1	0	0	1	0	0	0	0	0	0	0	0	0	1	1	0	1	10
**2.900**	0	0	1	1	0	0	0	0	0	0	0	0	0	0	0	0	0	0	0	0	0	0	0	1	0	10
**2.901**	0	0	1	0	0	0	0	0	1	0	0	0	0	1	0	0	0	0	0	0	0	0	0	1	1	10
**2.902**	0	0	0	0	0	0	0	0	1	0	0	1	1	0	0	0	0	0	0	0	0	0	0	0	0	10
**2.903**	0	0	0	0	0	0	0	0	0	1	0	0	0	0	0	0	0	0	0	1	0	1	1	0	1	10
**2.904**	0	0	0	0	0	0	0	0	0	0	1	0	0	0	0	0	0	0	0	1	0	0	0	1	1	10
**2.905**	0	1	0	1	1	0	0	0	0	0	0	1	0	0	0	0	0	0	0	1	0	0	1	1	0	10
**2.906**	0	1	0	0	0	0	0	0	0	0	0	1	1	1	0	0	0	0	0	0	0	0	1	0	0	10
**2.907**	0	0	1	1	0	0	0	0	0	0	0	0	0	0	0	0	0	0	0	1	0	0	0	0	0	10
**2.908**	0	0	0	1	0	0	0	0	0	0	0	0	1	0	0	0	0	0	0	0	0	0	0	1	0	10
**2.909**	0	0	0	0	1	0	0	0	0	0	0	1	0	0	0	1	0	0	0	0	0	0	0	1	0	10
**2.910**	0	1	0	0	1	0	0	0	0	1	0	1	1	0	0	0	0	0	0	0	0	1	1	0	0	10
**2.911**	0	0	0	0	0	0	1	0	1	0	0	0	0	0	0	0	0	0	0	0	0	1	1	0	0	10
**2.912**	0	0	1	0	0	0	0	0	0	1	0	0	1	0	0	0	0	0	0	1	0	0	0	0	0	10
**2.913**	0	0	1	0	0	0	0	0	0	0	1	0	0	0	0	0	0	0	0	0	0	0	1	0	0	10
**2.914**	0	0	0	0	0	0	0	0	0	0	1	1	0	0	0	0	0	0	0	1	0	0	0	0	0	10
**2.915**	0	0	1	0	0	0	0	0	0	0	0	0	0	0	0	1	0	0	0	1	0	0	0	1	0	10
**2.916**	0	0	0	0	0	0	0	0	1	0	0	0	1	0	0	1	0	0	0	1	0	1	0	1	0	10
**2.917**	0	1	1	0	0	0	0	0	0	0	0	0	0	0	0	0	0	0	0	0	0	1	0	0	0	10
**2.918**	0	0	0	0	0	0	0	0	1	1	0	0	1	0	0	1	0	0	0	1	0	0	0	0	0	10
**2.919**	0	0	1	1	1	0	0	0	1	1	0	1	1	0	0	0	0	0	0	1	0	0	0	1	1	9
**2.920**	0	0	0	0	1	0	0	0	1	0	0	1	1	0	0	0	0	0	0	1	0	0	0	0	0	9
**2.921**	0	1	1	1	1	0	0	0	1	1	0	1	0	0	0	0	0	0	0	1	0	1	0	1	1	9
**2.922**	0	1	0	0	0	0	0	0	0	0	0	0	0	1	0	0	0	0	0	1	0	1	1	0	0	9
**2.923**	0	0	1	0	1	0	0	0	1	0	0	1	0	0	0	0	0	0	0	1	0	1	0	1	1	9
**2.924**	0	0	0	1	1	0	0	0	1	0	0	1	1	0	0	0	0	0	0	0	0	0	0	0	1	9
**2.925**	0	0	0	0	1	0	0	0	1	1	0	1	1	0	0	0	0	0	0	0	0	0	1	0	0	9
**2.926**	0	1	0	1	1	0	1	0	1	0	0	1	1	0	0	0	0	0	0	1	0	0	0	1	1	9
**2.927**	0	1	0	1	1	0	0	0	0	1	0	1	1	1	0	0	0	0	0	1	0	1	1	0	0	9

CMP	CD2	CD3	CD4	CD7	CD8	CD10	CD13	CD18	CD26	CD29	CD36	CD44	CD45RA	CD49f	CD54	CD56	CD57	CD58	CD62L	CD71	CD80	Cytokeratin	Histone_H1	HLA-DQ	HLA-DR	Freq. (voxel)
2.928	0	1	0	0	1	0	1	0	1	0	0	1	0	0	0	0	0	0	0	0	0	0	0	0	1	9
2.929	0	1	0	0	1	0	0	0	1	0	0	1	1	1	0	0	0	0	0	1	0	0	0	1	1	9
2.930	0	1	0	0	0	0	0	0	1	1	0	1	0	0	0	0	0	0	0	0	0	1	0	1	0	9
2.931	0	0	0	1	1	0	0	0	0	1	0	1	0	0	0	0	0	0	0	1	0	1	0	1	0	9
2.932	0	0	0	0	1	0	1	0	1	1	0	1	0	0	0	0	0	0	0	1	0	0	0	1	1	9
2.933	0	0	0	0	1	0	0	0	0	1	0	1	0	1	0	0	0	0	0	1	0	0	0	1	1	9
2.934	0	1	0	1	1	0	1	0	0	1	0	1	1	0	0	0	0	0	0	1	0	0	0	1	1	9
2.935	0	0	1	0	0	0	0	0	1	0	0	0	0	0	0	0	0	0	0	1	0	1	0	1	0	9
2.936	0	0	0	1	1	0	0	0	0	1	0	1	1	0	0	0	0	0	0	1	0	0	1	0	0	9
2.937	0	0	0	1	0	0	1	0	0	0	0	1	0	0	0	1	0	0	0	1	0	0	0	1	1	9
2.938	0	0	0	0	1	0	1	0	1	1	0	1	0	1	0	0	0	0	0	1	0	1	0	1	1	9
2.939	0	0	0	0	1	0	1	0	1	0	0	0	0	0	0	1	0	0	0	0	0	0	0	1	1	9
2.940	0	0	0	0	1	0	1	0	0	1	0	1	0	0	0	0	0	0	0	1	0	1	0	1	1	9
2.941	0	0	0	0	1	0	0	0	1	0	0	0	0	0	0	0	0	0	0	0	0	1	0	1	1	9
2.942	0	0	0	0	0	0	0	0	1	0	0	1	1	0	0	0	0	0	0	0	0	1	0	1	0	9
2.943	0	0	0	0	0	0	0	0	0	1	0	0	0	0	0	1	0	0	0	1	0	0	0	0	0	9
2.944	0	0	0	0	0	0	0	0	0	0	0	0	1	1	0	0	0	0	0	1	0	0	0	0	0	9
2.945	0	1	1	1	1	0	0	0	0	1	0	1	1	0	0	0	0	0	0	1	0	0	0	0	0	9
2.946	0	1	1	0	1	0	0	0	1	1	0	1	1	1	0	0	0	0	0	1	0	0	0	0	1	9
2.947	0	1	0	0	0	0	0	0	1	0	0	1	1	1	0	0	0	0	0	1	0	1	0	1	1	9
2.948	0	0	0	1	0	0	0	0	0	0	0	0	0	0	0	0	0	0	0	1	0	0	0	1	1	9
2.949	0	0	0	0	1	0	0	0	0	1	0	1	1	0	0	0	0	0	0	0	0	1	0	1	1	9
2.950	0	1	1	0	1	0	1	0	1	0	0	1	0	0	0	0	0	0	0	0	0	0	0	1	1	9
2.951	0	1	1	0	1	0	0	0	1	0	0	0	1	0	0	0	0	0	0	1	0	0	0	1	1	9
2.952	0	1	0	1	0	0	0	0	0	0	0	0	0	0	0	0	0	0	0	1	0	1	1	1	1	9
2.953	0	1	0	0	1	0	0	0	0	1	0	1	1	1	0	0	0	0	0	1	0	0	0	1	0	9
2.954	0	1	0	0	0	0	0	0	1	1	0	1	0	0	0	0	0	0	0	0	0	1	1	0	0	9
2.955	0	1	0	0	0	0	0	0	1	0	0	1	1	0	0	0	0	0	0	1	0	0	1	0	0	9
2.956	0	0	1	1	0	0	0	0	0	0	0	0	1	1	0	0	0	0	0	1	0	0	0	1	0	9
2.957	0	0	1	1	0	0	0	0	0	0	0	0	0	0	0	0	0	0	0	1	0	0	0	1	0	9
2.958	0	0	0	0	1	0	0	0	0	1	0	0	1	0	0	0	0	0	0	0	0	0	0	0	0	9
2.959	0	0	0	0	0	0	0	0	0	0	0	1	0	0	0	1	0	0	0	1	0	0	1	1	1	9
2.960	0	1	1	1	1	0	0	0	0	1	0	1	1	0	0	0	0	0	0	1	0	0	0	0	1	9
2.961	0	1	1	1	1	0	0	0	0	0	0	1	0	0	0	0	0	0	0	0	0	0	0	1	1	9
2.962	0	1	0	1	1	0	0	0	0	0	0	1	1	0	0	0	0	0	0	0	0	1	1	1	0	9
2.963	0	1	0	1	0	0	0	0	0	0	0	1	0	1	0	0	0	0	0	1	0	0	0	0	1	9
2.964	0	1	0	0	1	0	0	0	0	1	0	1	1	1	0	0	0	0	0	0	0	0	0	1	1	9
2.965	0	1	0	0	0	0	0	0	1	1	0	1	1	1	0	0	0	0	0	1	0	0	0	1	0	9
2.966	0	1	0	0	0	0	0	0	0	0	1	1	0	1	0	0	0	0	0	1	0	1	0	1	1	9
2.967	0	0	0	1	0	0	0	0	0	0	0	1	0	1	0	0	0	0	0	1	0	1	0	0	0	9
2.968	0	0	0	0	1	0	1	0	1	1	0	1	0	1	0	0	0	0	0	1	0	1	0	0	0	9
2.969	0	0	0	0	0	0	0	0	0	0	0	1	0	0	0	1	0	0	0	0	0	1	1	0	0	9
2.970	0	1	0	1	1	0	0	0	0	1	0	1	1	0	0	0	0	0	0	0	0	0	1	0	1	9
2.971	0	1	0	1	1	0	0	0	0	0	0	1	1	0	0	0	0	0	0	1	0	0	1	1	0	9
2.972	0	1	0	0	1	0	0	0	1	0	0	0	0	0	0	0	0	0	0	0	0	0	0	1	1	9
2.973	0	1	0	0	0	0	0	0	1	0	0	1	0	0	0	0	0	0	0	0	0	0	1	0	1	9
2.974	0	1	0	0	0	0	0	0	0	0	0	1	1	0	0	0	0	0	0	0	0	1	1	1	1	9
2.975	0	0	1	1	0	0	1	0	1	1	0	1	1	1	0	0	0	0	0	1	0	1	0	1	1	9
2.976	0	0	0	1	0	0	1	0	0	0	0	0	0	1	0	0	0	0	0	1	0	1	0	0	0	9
2.977	0	1	1	1	1	0	0	0	1	0	0	1	0	0	0	0	0	0	0	0	0	0	0	1	0	9
2.978	0	1	1	1	1	0	0	0	0	0	0	1	0	0	0	0	0	0	0	1	0	0	1	0	0	9
2.979	0	1	0	1	0	0	0	0	1	1	0	0	0	0	0	0	0	0	0	0	0	0	0	0	0	9
2.980	0	1	0	0	0	0	0	0	0	1	0	1	1	0	0	0	0	0	0	0	0	0	0	0	1	9
2.981	0	1	0	0	0	0	0	0	0	0	0	1	0	1	0	0	0	0	0	1	0	0	0	1	0	9
2.982	0	0	0	0	0	0	1	0	1	1	0	1	0	1	0	0	0	0	0	0	0	1	0	1	1	9
2.983	0	0	0	0	0	0	0	0	0	0	1	0	1	0	0	1	0	0	0	0	0	0	0	0	0	9
2.984	0	1	1	1	1	0	0	0	1	0	0	1	0	0	0	0	0	0	0	0	0	0	0	0	0	9
2.985	0	1	0	1	1	0	0	0	0	0	0	0	1	0	0	0	0	0	0	0	0	0	0	0	1	9
2.986	0	1	0	0	0	0	1	0	0	0	0	0	0	0	0	0	0	0	0	0	0	1	1	0	1	9
2.987	0	1	0	0	0	0	0	0	1	0	0	0	1	0	0	0	0	0	0	0	0	0	1	0	0	9
2.988	0	1	0	0	0	0	0	0	0	1	0	0	0	1	0	0	0	0	0	1	0	0	1	0	0	9
2.989	0	1	0	0	0	0	0	0	0	0	0	0	0	1	0	0	0	0	0	0	0	1	0	1	0	9
2.990	0	0	1	0	1	0	0	0	0	0	0	0	0	0	0	0	0	0	0	0	0	0	1	0	0	9
2.991	0	0	1	0	0	0	0	0	1	0	1	0	0	1	0	0	0	0	0	1	0	1	0	0	0	9
2.992	0	0	1	0	0	0	0	0	0	1	0	1	0	0	0	0	0	0	0	0	0	0	1	1	0	9
2.993	0	0	1	0	0	0	0	0	0	0	0	0	0	0	0	0	0	0	0	0	0	0	1	0	1	9
2.994	0	0	0	0	0	0	1	0	0	0	0	1	0	0	0	0	0	0	0	1	0	1	1	0	0	9
2.995	0	1	0	1	1	0	0	0	1	1	0	1	1	0	0	0	0	0	0	1	0	1	0	0	0	9
2.996	0	1	0	1	1	0	0	0	0	1	0	1	0	0	0	0	0	0	0	1	0	0	1	1	1	9
2.997	0	1	0	1	0	0	0	0	1	1	0	1	1	0	0	0	0	0	0	0	0	0	0	0	0	9
2.998	0	1	0	1	0	0	0	0	0	0	0	1	0	0	0	0	0	0	0	0	0	1	0	1	0	9
2.999	0	0	1	1	0	0	1	0	1	1	0	1	1	1	0	0	0	0	0	1	0	0	1	1	1	9
3.000	0	0	1	0	0	0	1	0	1	1	0	0	0	1	0	0	0	0	0	1	0	0	0	1	1	9
3.001	0	0	1	0	0	0	0	0	1	1	0	0	0	0	0	0	0	0	0	0	0	0	0	0	0	9
3.002	0	0	0	1	1	0	0	0	0	0	0	0	1	0	0	0	0	0	0	0	0	0	0	0	0	9
3.003	0	0	0	1	0	0	1	0	1	1	0	1	0	1	0	0	0	0	0	0	0	0	0	1	1	9
3.004	0	0	0	1	0	0	0	0	0	0	0	0	0	1	0	0	0	0	0	0	0	1	0	0	1	9
3.005	0	0	0	0	1	0	0	0	1	0	0	0	0	0	0	0	0	0	0	1	0	1	0	0	0	9
3.006	0	0	0	0	1	0	0	0	0	1	0	1	0	1	0	0	0	0	0	1	0	1	1	0	1	9
3.007	0	0	0	0	0	0	0	0	1	0	0	1	0	1	0	0	0	0	0	0	0	0	1	1	1	9
3.008	0	0	0	0	0	0	0	0	0	0	0	0	1	1	0	0	0	0	0	0	0	1	0	1	0	9
3.009	0	1	0	1	1	0	0	0	1	0	0	1	0	0	0	0	0	0	0	1	0	0	1	1	0	9
3.010	0	0	1	1	0	0	1	0	1	1	1	1	0	1	0	0	0	0	0	1	0	0	0	1	1	9
3.011	0	0	1	0	0	0	1	0	1	0	0	0	0	1	0	0	0	0	0	1	0	0	0	1	1	9
3.012	0	0	1	0	0	0	0	0	0	0	0	0	1	1	0	0	0	0	0	0	0	1	0	1	0	9
3.013	0	0	0	1	0	0	1	0	0	1	1	1	0	1	0	0	0	0	0	0	0	0	0	1	0	9
3.014	0	0	0	0	1	0	0	0	0	1	1	1	0	1	0	0	0	0	0	0	0	1	1	0	1	9
3.015	0	0	0	0	0	0	1	0	1	1	0	1	0	0	0	0	0	0	0	1	0	0	0	0	0	9
3.016	0	0	0	0	0	0	1	0	0	1	0	1	1	1	0	0	0	0	0	1	0	1	0	0	1	9
3.017	0	0	0	0	0	0	0	0	1	0	0	1	0	0	0	1	0	0	0	0	0	1	0	0	0	9
3.018	0	0	0	0	0	0	0	0	1	0	0	0	1	0	0	0	0	0	0	0	0	1	0	1	0	9
3.019	0	0	1	0	1	0	1	0	0	1	0	1	0	0	0	0	0	0	0	0	0	0	0	0	0	9
3.020	0	0	1	0	0	0	0	0	0	1	0	0	0	1	0	0	0	0	0	0	0	1	1	0	0	9
3.021	0	0	0	0	0	0	1	0	1	0	0	0	0	0	0	0	0	0	0	0	0	0	1	0	0	9
3.022	0	0	0	0	0	0	0	0	1	1	0	0	0	0	0	0	0	0	0	1	0	0	0	1	1	9
3.023	0	0	0	0	0	0	0	0	1	1	0	0	0	0	0	0	0	0	0	0	0	1	0	0	1	9
3.024	0	0	0	0	0	0	0	0	1	0	0	0	0	0	0	0	0	0	0	1	0	0	1	1	1	9
3.025	0	0	0	0	0	0	0	0	0	1	1	1	0	0	0	0	0	0	0	1	0	1	0	0	0	9
3.026	0	0	0	0	0	0	0	0	0	0	0	0	0	0	0	1	0	0	0	1	0	0	1	1	1	9
3.027	0	0	1	1	0	0	1	0	0	1	1	1	1	1	0	1	0	0	0	1	0	0	0	1	1	9
3.028	0	0	1	1	0	0	0	0	0	0	0	0	0	0	0	1	0	0	0	0	0	0	0	1	0	9
3.029	0	0	1	0	0	0	0	0	1	1	0	1	0	0	0	0	0	0	0	1	0	0	0	1	0	9
3.030	0	0	1	0	0	0	0	0	0	1	1	1	0	1	0	0	0	0	0	1	0	0	0	1	1	9
3.031	0	1	0	0	0	0	0	0	1	0	0	0	0	0	0	0	0	0	0	0	0	0	0	1	1	9
3.032	0	0	1	1	0	0	0	0	0	0	0	0	0	0	0	1	0	0	0	0	0	0	0	0	0	9
3.033	0	0	1	0	0	0	1	0	1	1	0	1	0	1	0	0	0	0	0	0	0	1	0	0	0	9
3.034	0	0	1	0	0	0	0	0	0	1	0	1	0	1	0	0	0	0	0	1	0	1	1	1	0	9
3.035	0	0	1	0	0	0	0	0	0	1	0	0	0	0	0	0	0	0	0	0	0	0	1	0	0	9
3.036	0	0	0	1	0	0	1	0	0	1	0	1	0	1	0	0	0	0	0	1	0	0	0	0	1	9
3.037	0	0	0	0	1	0	0	0	0	0	0	0	0	0	0	0	0	0	0	0	0	0	1	1	0	9
3.038	0	0	0	0	0	0	0	0	1	0	1	1	0	0	0	0	0	0	0	1	0	0	0	1	1	9
3.039	0	0	0	0	0	0	0	0	1	0	0	1	0	0	0	0	0	0	0	0	0	1	1	1	1	9
3.040	0	0	0	0	0	0	0	0	1	0	0	0	0	1	0	1	0	0	0	0	0	1	0	0	0	9
3.041	0	0	0	0	0	0	0	0	0	0	0	1	0	1	0	0	0	0	0	0	0	1	1	0	1	9
3.042	0	1	0	0	0	0	0	0	0	0	0	1	0	1	0	0	0	0	0	0	0	1	0	0	0	9
3.043	0	0	0	1	0	0	0	0	0	0	0	0	0	0	0	0	0	0	0	1	0	1	0	1	0	9
3.044	0	0	0	0	0	0	1	0	1	1	0	1	0	1	0	0	0	0	0	0	0	1	0	1	0	9
3.045	0	0	0	0	0	0	1	0	0	0	0	0	0	0	0	0	0	0	0	0	0	1	1	0	1	9
3.046	0	0	0	0	0	0	0	0	0	1	0	0	1	1	0	0	0	0	0	1	0	0	0	1	1	9
3.047	0	1	0	0	0	0	0	0	0	0	0	1	1	0	0	0	0	0	0	0	0	0	0	1	0	9
3.048	0	0	1	0	0	0	0	0	0	0	0	1	0	0	0	0	0	0	0	0	0	1	1	0	0	9
3.049	0	1	0	1	0	0	0	0	1	0	0	0	0	0	0	0	0	0	0	0	0	1	0	0	0	9

CMP	CD2	CD3	CD4	CD7	CD8	CD10	CD13	CD18	CD26	CD29	CD36	CD44	CD45RA	CD49f	CD54	CD56	CD57	CD58	CD62L	CD71	CD80	Cytokeratin	Histone_H1	HLA-DQ	HLA-DR	Freq. (voxel)
**3.050**	0	0	1	0	0	0	0	0	0	0	1	0	0	0	0	0	0	0	0	0	0	1	1	0	0	9
**3.051**	0	1	0	0	1	0	1	0	0	0	0	1	0	0	0	0	0	0	0	1	0	0	0	0	0	9
**3.052**	0	1	0	0	1	0	1	0	0	0	0	0	0	0	0	0	0	0	0	1	0	0	1	0	0	9
**3.053**	0	1	1	0	1	0	1	0	1	1	0	1	1	0	0	0	0	0	0	1	0	1	0	1	1	8
**3.054**	0	1	0	1	1	0	1	0	1	1	0	1	0	0	0	0	0	0	0	0	0	0	0	0	1	8
**3.055**	0	0	1	0	0	0	1	0	0	0	0	1	0	0	0	0	0	0	0	0	0	1	0	1	1	8
**3.056**	0	0	0	1	0	0	1	0	0	0	0	0	1	0	0	1	0	0	0	0	0	1	0	1	0	8
**3.057**	0	0	0	1	0	0	0	0	1	0	0	0	0	0	0	0	0	0	0	1	0	1	0	1	1	8
**3.058**	0	0	0	0	1	0	0	0	0	0	0	1	1	0	0	0	0	0	0	1	0	0	0	0	1	8
**3.059**	0	1	1	1	1	0	0	0	1	0	0	1	1	0	0	0	0	0	0	1	0	1	0	1	1	8
**3.060**	0	1	0	1	1	0	0	0	1	0	0	1	1	1	0	0	0	0	0	1	0	0	0	1	1	8
**3.061**	0	1	0	1	1	0	0	0	0	0	0	1	1	1	0	0	0	0	0	0	0	0	0	1	0	8
**3.062**	0	1	0	1	0	0	0	0	1	0	0	1	0	0	0	0	0	0	0	1	0	1	1	1	1	8
**3.063**	0	1	0	0	1	0	0	0	1	1	0	1	1	0	0	0	0	0	0	0	0	1	1	1	0	8
**3.064**	0	1	0	0	1	0	0	0	1	0	0	1	1	1	0	0	0	0	0	1	0	1	0	1	1	8
**3.065**	0	0	1	0	1	0	0	0	0	0	0	1	0	0	0	0	0	0	0	1	0	1	0	1	1	8
**3.066**	0	0	1	0	0	0	1	0	1	1	0	1	0	0	0	0	0	0	0	1	0	0	1	1	1	8
**3.067**	0	0	1	0	0	0	0	0	1	0	0	0	1	0	0	0	0	0	0	1	0	1	0	1	1	8
**3.068**	0	0	0	1	1	0	1	0	1	1	0	1	0	0	0	0	0	0	0	1	0	1	0	1	1	8
**3.069**	0	0	0	1	1	0	1	0	1	0	0	1	0	0	0	1	0	0	0	1	0	0	0	1	1	8
**3.070**	0	0	0	1	1	0	0	0	1	1	0	1	0	0	0	0	0	0	0	0	0	0	0	1	0	8
**3.071**	0	0	0	1	0	0	0	0	0	0	0	0	1	0	0	1	0	0	0	0	0	1	0	1	0	8
**3.072**	0	0	0	0	1	0	0	0	1	1	0	1	0	1	0	0	0	0	0	1	0	0	0	1	1	8
**3.073**	0	0	0	0	1	0	0	0	1	1	0	1	0	1	0	0	0	0	0	1	0	0	0	0	1	8
**3.074**	0	0	0	0	1	0	0	0	1	0	0	1	1	0	0	0	0	0	0	0	0	1	1	0	1	8
**3.075**	0	0	0	0	0	0	1	0	0	1	0	0	0	0	0	0	0	0	0	1	0	0	0	1	0	8
**3.076**	0	1	1	0	0	0	0	0	1	0	0	1	1	0	0	0	0	0	0	1	0	0	0	0	0	8
**3.077**	0	1	0	1	1	0	0	0	0	0	0	0	0	0	0	0	0	0	0	1	0	0	1	1	1	8
**3.078**	0	1	0	0	1	0	0	0	1	1	0	1	1	1	0	0	0	0	0	0	0	1	1	0	0	8
**3.079**	0	1	0	0	1	0	0	0	0	0	0	1	0	1	0	0	0	0	0	1	0	1	0	0	0	8
**3.080**	0	0	1	0	0	0	0	0	0	1	0	0	0	1	0	0	0	0	0	0	0	1	0	1	0	8
**3.081**	0	0	0	0	1	0	1	0	0	0	0	1	0	0	0	0	0	0	0	0	0	1	0	1	1	8
**3.082**	0	0	0	0	1	0	0	0	0	1	0	1	0	1	0	0	0	0	0	0	0	0	1	0	0	8
**3.083**	0	0	0	0	0	0	1	0	0	1	0	1	0	0	0	0	0	0	0	1	0	1	0	1	1	8
**3.084**	0	0	0	0	0	0	0	0	1	0	0	1	1	0	0	0	0	0	0	1	0	0	0	1	1	8
**3.085**	0	0	0	0	0	0	0	0	0	1	0	1	1	0	0	0	0	0	0	1	0	1	1	0	0	8
**3.086**	0	0	0	0	0	0	0	0	0	0	1	0	0	0	0	0	0	0	0	1	0	0	0	0	1	8
**3.087**	0	0	0	0	0	0	0	0	0	0	0	1	1	1	0	0	0	0	0	0	0	1	0	1	1	8
**3.088**	0	1	1	0	1	0	0	0	0	1	0	1	0	0	0	0	0	0	0	1	0	0	0	0	1	8
**3.089**	0	1	1	0	0	0	1	0	1	0	0	1	0	0	0	0	0	0	0	1	0	0	0	1	1	8
**3.090**	0	1	1	0	0	0	0	0	1	0	0	1	0	0	0	0	0	0	0	1	0	0	1	0	0	8
**3.091**	0	1	1	0	0	0	0	0	0	0	0	0	0	0	0	0	0	0	0	0	0	0	0	1	1	8
**3.092**	0	1	0	1	1	0	1	0	0	1	0	1	0	0	0	0	0	0	0	0	0	0	0	1	0	8
**3.093**	0	1	0	1	0	0	0	0	0	1	0	1	0	0	0	0	0	0	0	1	0	1	0	0	1	8
**3.094**	0	1	0	0	1	0	0	0	1	1	0	1	1	1	0	0	0	0	0	1	0	0	0	1	0	8
**3.095**	0	1	0	0	0	0	1	0	0	0	0	1	0	0	0	0	0	0	0	0	0	0	1	0	1	8
**3.096**	0	0	1	0	1	0	1	0	0	1	0	0	0	0	0	0	0	0	0	0	0	0	0	1	1	8
**3.097**	0	0	1	0	1	0	0	0	1	1	0	1	0	0	0	0	0	0	0	0	0	0	0	1	1	8
**3.098**	0	0	1	0	0	0	0	0	0	1	0	1	0	0	0	0	0	0	0	1	0	0	1	0	0	8
**3.099**	0	0	0	1	1	0	0	0	0	0	0	1	1	0	0	0	0	0	0	1	0	0	1	0	0	8
**3.100**	0	0	0	1	1	0	0	0	0	0	0	0	1	0	0	0	0	0	0	1	0	0	1	0	0	8
**3.101**	0	0	0	1	0	0	0	0	0	0	1	0	0	1	0	0	0	0	0	0	0	1	0	0	0	8
**3.102**	0	0	0	0	1	0	1	0	1	1	0	1	0	1	0	0	0	0	0	1	0	0	0	0	1	8
**3.103**	0	0	0	0	1	0	0	0	0	1	0	1	1	0	0	0	0	0	0	1	0	0	0	1	0	8
**3.104**	0	0	0	0	1	0	0	0	0	1	0	0	1	0	0	0	0	0	0	0	0	0	1	0	0	8
**3.105**	0	0	0	0	0	0	1	0	0	1	0	0	1	1	0	0	0	0	0	0	0	0	0	1	0	8
**3.106**	0	0	0	0	0	0	0	0	1	1	0	1	0	0	0	0	0	0	0	1	0	1	1	0	0	8
**3.107**	0	0	0	0	0	0	0	0	1	0	1	1	0	0	0	0	0	0	0	0	0	1	0	1	1	8
**3.108**	0	0	0	0	0	0	0	0	0	0	0	1	1	0	0	0	0	0	0	1	0	0	0	1	1	8
**3.109**	0	1	0	1	1	0	1	0	0	0	0	1	0	0	0	0	0	0	0	0	0	0	0	1	0	8
**3.110**	0	1	0	1	1	0	0	0	1	0	0	1	0	1	0	0	0	0	0	1	0	0	0	0	0	8
**3.111**	0	1	0	0	0	0	0	0	0	0	0	0	0	0	0	0	0	0	0	1	0	0	0	1	1	8
**3.112**	0	0	1	0	1	0	1	0	0	0	0	0	0	0	0	0	0	0	0	1	0	0	0	1	1	8
**3.113**	0	0	1	0	0	0	1	0	0	0	0	0	1	1	0	0	0	0	0	1	0	1	0	0	0	8
**3.114**	0	0	1	0	0	0	1	0	0	0	0	0	0	0	0	1	0	0	0	1	0	0	0	1	1	8
**3.115**	0	0	0	0	1	0	1	0	0	0	0	1	0	0	0	0	0	0	0	0	0	0	1	0	1	8
**3.116**	0	0	0	0	0	0	1	0	0	1	0	0	1	1	0	0	0	0	0	0	0	0	1	1	0	8
**3.117**	0	0	0	0	0	0	0	0	0	1	0	0	1	1	0	1	0	0	0	0	0	0	0	0	0	8
**3.118**	0	1	1	1	1	0	0	0	0	1	0	1	0	1	0	0	0	0	0	1	0	0	0	1	0	8
**3.119**	0	1	1	0	0	0	0	0	1	1	0	1	1	0	0	0	0	0	0	0	0	0	1	0	0	8
**3.120**	0	1	0	1	1	0	0	0	0	1	0	1	0	1	0	0	0	0	0	1	0	0	0	0	1	8
**3.121**	0	1	0	0	0	0	1	0	0	1	0	1	0	1	0	0	0	0	0	1	0	1	0	0	0	8
**3.122**	0	1	0	0	0	0	0	0	0	1	0	1	1	1	0	0	0	0	0	1	0	0	0	0	0	8
**3.123**	0	1	0	0	0	0	0	0	0	0	0	0	0	0	0	0	0	0	0	1	0	1	1	1	0	8
**3.124**	0	0	0	1	1	0	0	0	0	1	0	1	0	1	0	0	0	0	0	0	0	1	0	1	1	8
**3.125**	0	0	0	0	1	0	0	0	1	1	0	1	0	1	0	0	0	0	0	0	0	1	0	0	0	8
**3.126**	0	0	0	0	0	0	0	0	1	1	0	1	1	0	0	0	0	0	0	1	0	0	0	0	0	8
**3.127**	0	1	0	1	1	0	1	0	0	0	0	1	0	0	0	0	0	0	0	0	0	0	1	0	0	8
**3.128**	0	1	0	1	0	0	1	0	0	0	0	0	0	0	0	0	0	0	0	0	0	0	1	0	0	8
**3.129**	0	1	0	0	1	0	0	0	1	0	0	1	1	0	0	0	0	0	0	0	0	0	1	1	1	8
**3.130**	0	1	0	0	1	0	0	0	1	0	0	0	1	0	0	0	0	0	0	0	0	0	0	0	1	8
**3.131**	0	1	0	0	1	0	0	0	0	0	0	0	1	0	0	0	0	0	0	0	0	0	0	1	0	8
**3.132**	0	1	0	0	0	0	1	0	0	0	0	1	0	1	0	0	0	0	0	0	0	0	1	0	0	8
**3.133**	0	1	0	0	0	0	0	0	0	1	0	1	0	1	0	0	0	0	0	1	0	0	0	0	1	8
**3.134**	0	0	1	1	0	0	1	0	1	1	0	1	1	1	0	0	0	0	0	0	0	0	0	1	1	8
**3.135**	0	0	1	0	1	0	0	0	0	0	0	1	0	0	0	0	0	0	0	0	0	0	0	1	0	8
**3.136**	0	0	1	0	0	0	0	0	0	1	0	0	0	0	0	0	0	0	0	0	0	0	0	0	1	8
**3.137**	0	0	1	0	0	0	0	0	0	0	0	0	1	1	0	1	0	0	0	0	0	1	0	0	0	8
**3.138**	0	0	0	0	0	0	1	0	0	0	0	1	0	0	0	1	0	0	0	0	0	0	0	0	1	8
**3.139**	0	0	0	0	0	0	0	0	0	1	0	1	1	0	0	0	0	0	0	0	0	1	1	0	0	8
**3.140**	0	1	0	0	1	0	0	0	0	0	0	0	1	0	0	0	0	0	0	0	0	1	1	0	1	8
**3.141**	0	1	0	0	0	0	0	0	1	0	0	1	1	0	0	0	0	0	0	0	0	1	1	0	0	8
**3.142**	0	1	0	0	0	0	0	0	0	1	1	1	0	0	0	0	0	0	0	0	0	0	0	1	1	8
**3.143**	0	0	1	0	0	0	0	0	0	0	0	0	1	1	0	0	0	0	0	0	0	1	0	0	0	8
**3.144**	0	0	0	1	0	0	1	0	0	0	0	0	0	0	0	0	0	0	0	0	0	0	1	0	0	8
**3.145**	0	1	1	1	1	0	0	0	0	1	0	1	0	0	0	0	0	0	0	0	0	0	0	1	0	8
**3.146**	0	1	1	1	0	0	0	0	1	1	0	1	0	0	0	0	0	0	0	0	0	0	0	1	0	8
**3.147**	0	1	0	0	0	0	0	0	0	1	0	1	0	1	0	0	0	0	0	1	0	0	1	0	1	8
**3.148**	0	0	1	0	0	0	1	0	1	1	0	1	1	1	0	0	0	0	0	1	0	0	1	1	1	8
**3.149**	0	0	1	0	0	0	1	0	0	1	0	1	0	1	0	0	0	0	0	0	0	1	1	0	0	8
**3.150**	0	0	0	1	0	0	0	0	1	1	0	1	0	1	0	0	0	0	0	0	0	0	0	0	0	8
**3.151**	0	0	0	0	0	0	1	0	0	1	0	1	1	1	0	0	0	0	0	0	0	1	0	0	0	8
**3.152**	0	1	0	0	0	0	0	0	0	1	0	1	0	0	0	0	0	0	0	1	0	0	1	0	1	8
**3.153**	0	1	0	0	0	0	0	0	0	0	1	1	0	0	0	0	0	0	0	0	0	1	0	0	0	8
**3.154**	0	0	1	0	0	0	1	0	0	0	0	0	0	1	0	0	0	0	0	0	0	1	0	0	1	8
**3.155**	0	0	1	0	0	0	0	0	0	0	1	0	0	0	0	0	0	0	0	0	0	1	0	0	1	8
**3.156**	0	0	0	1	0	0	1	0	1	1	0	1	1	1	0	0	0	0	0	1	0	0	1	1	1	8
**3.157**	0	0	0	1	0	0	1	0	0	1	0	1	0	1	0	0	0	0	0	0	0	1	0	0	1	8
**3.158**	0	0	0	0	0	0	1	0	1	1	0	1	0	1	0	0	0	0	0	1	0	0	1	0	1	8
**3.159**	0	0	0	0	0	0	0	0	0	0	0	0	1	1	0	0	0	0	0	0	0	0	0	0	0	8
**3.160**	0	0	1	1	0	0	1	0	0	1	1	1	0	1	0	0	0	0	0	0	0	0	0	1	1	8
**3.161**	0	0	1	1	0	0	0	0	0	0	0	0	1	0	0	1	0	0	0	0	0	0	0	0	0	8
**3.162**	0	0	1	1	0	0	0	0	0	0	0	0	0	0	0	0	0	0	0	1	0	1	0	0	0	8
**3.163**	0	0	1	0	0	0	0	0	1	1	0	1	0	1	0	0	0	0	0	0	0	1	0	0	1	8
**3.164**	0	0	1	0	0	0	0	0	0	0	1	0	0	1	0	0	0	0	0	1	0	1	0	0	0	8
**3.165**	0	0	0	1	0	0	1	0	0	1	1	1	0	1	0	0	0	0	0	0	0	0	0	1	1	8
**3.166**	0	0	0	1	0	0	0	0	0	1	0	1	0	1	0	0	0	0	0	1	0	0	0	1	0	8
**3.167**	0	0	0	1	0	0	0	0	0	0	0	0	0	0	0	0	0	0	0	0	0	1	0	1	0	8
**3.168**	0	0	0	0	0	0	1	0	1	0	0	0	1	0	0	1	0	0	0	0	0	1	0	0	0	8
**3.169**	0	0	0	0	0	0	0	0	1	0	0	1	0	1	0	0	0	0	0	0	0	0	0	1	0	8
**3.170**	0	1	0	0	1	0	0	0	0	1	1	1	0	1	0	0	0	0	0	0	0	0	0	0	0	8
**3.171**	0	0	1	0	0	0	1	0	1	1	1	1	0	1	0	0	0	0	0	0	0	0	0	1	1	8

CMP	CD2	CD3	CD4	CD7	CD8	CD10	CD13	CD18	CD26	CD29	CD36	CD44	CD45RA	CD49f	CD54	CD56	CD57	CD58	CD62L	CD71	CD80	Cytokeratin	Histone_H1	HLA-DQ	HLA-DR	Freq. (voxel)
**3.172**	0	0	1	0	0	0	1	0	0	1	1	1	0	1	0	0	0	0	0	0	0	0	0	1	0	8
**3.173**	0	0	1	0	0	0	0	0	1	1	0	1	0	1	0	0	0	0	0	1	0	1	1	1	1	8
**3.174**	0	0	1	0	0	0	0	0	1	1	0	1	0	1	0	0	0	0	0	0	0	0	1	1	1	8
**3.175**	0	0	1	0	0	0	0	0	0	1	0	0	0	1	0	0	0	0	0	1	0	0	0	0	1	8
**3.176**	0	0	1	0	0	0	0	0	0	0	0	1	0	1	0	0	0	0	0	0	0	1	1	0	0	8
**3.177**	0	0	0	0	1	0	0	0	1	1	0	1	0	0	0	0	0	0	0	0	0	0	1	0	1	8
**3.178**	0	0	0	0	0	0	1	0	0	1	0	1	0	1	0	1	0	0	0	1	0	0	0	1	1	8
**3.179**	0	0	0	0	0	0	0	0	1	0	0	1	0	0	0	0	0	0	0	1	0	1	1	0	1	8
**3.180**	0	1	0	0	1	0	1	0	0	1	0	1	0	1	0	0	0	0	0	0	0	1	0	0	0	8
**3.181**	0	0	1	1	0	0	1	0	0	1	0	0	1	1	0	0	0	0	0	1	0	0	0	1	1	8
**3.182**	0	0	1	0	0	0	0	0	0	0	0	1	0	1	0	0	0	0	0	1	0	0	0	0	0	8
**3.183**	0	1	0	0	0	0	0	0	0	0	0	1	0	0	0	1	0	0	0	0	0	0	0	0	0	8
**3.184**	0	0	1	0	0	0	0	0	0	1	0	1	0	1	0	1	0	0	0	0	0	1	0	1	0	8
**3.185**	0	0	1	0	0	0	0	0	0	0	0	0	0	0	0	1	0	0	0	1	0	0	0	0	0	8
**3.186**	0	0	0	0	1	0	1	0	0	1	0	1	0	0	0	0	0	0	0	1	0	1	0	0	0	8
**3.187**	0	0	0	0	0	0	1	0	1	0	0	0	0	0	0	0	0	0	0	1	0	0	0	1	0	8
**3.188**	0	0	0	0	0	0	0	0	1	1	0	1	0	1	0	0	0	0	0	0	0	0	1	1	1	8
**3.189**	0	0	0	0	0	0	0	0	1	0	0	0	1	0	0	0	0	0	0	1	0	1	0	1	0	8
**3.190**	0	1	0	0	0	0	0	0	0	0	1	0	0	0	0	0	0	0	0	0	0	0	0	0	0	8
**3.191**	0	0	0	0	1	0	1	0	0	0	0	1	0	1	0	0	0	0	0	0	0	1	1	0	0	8
**3.192**	0	0	0	0	1	0	0	0	0	1	1	1	0	0	0	0	0	0	0	0	0	0	0	0	0	8
**3.193**	0	0	0	0	0	0	1	0	0	0	0	0	0	1	0	0	0	0	0	1	0	1	1	0	1	8
**3.194**	0	0	0	0	0	0	0	0	1	1	1	1	0	0	0	0	0	0	0	1	0	0	0	1	1	8
**3.195**	0	1	0	0	0	0	0	0	1	0	0	0	0	0	0	0	0	0	0	0	0	0	0	1	0	8
**3.196**	0	0	0	0	1	0	1	0	0	1	0	1	0	0	0	0	0	0	0	0	0	0	0	1	0	8
**3.197**	0	1	0	1	1	0	1	0	0	0	0	1	0	0	0	0	0	0	0	1	0	0	0	0	0	8
**3.198**	0	1	0	0	1	0	1	0	0	1	0	1	0	0	0	0	0	0	0	0	0	0	0	0	1	8
**3.199**	0	1	0	0	0	0	0	0	1	0	0	0	0	0	0	0	0	0	0	1	0	1	0	0	0	8
**3.200**	0	1	0	0	0	0	0	0	0	0	0	0	1	0	0	0	0	0	0	1	0	0	0	0	0	8
**3.201**	0	0	1	0	0	0	0	0	0	1	1	0	0	0	0	0	0	0	0	0	0	0	0	1	1	8
**3.202**	0	0	0	0	1	0	0	0	1	0	0	0	1	0	0	0	0	0	0	1	0	1	0	0	0	8
**3.203**	0	0	1	0	0	0	0	0	0	0	0	0	1	1	0	1	0	0	0	0	0	0	0	1	0	8
**3.204**	0	0	1	0	0	0	0	0	0	0	0	0	1	0	0	0	0	0	0	1	0	0	1	0	0	8
**3.205**	0	0	0	0	0	0	0	0	0	1	1	0	0	0	0	0	0	0	0	0	0	1	0	1	0	8
**3.206**	0	1	0	0	0	0	1	0	0	0	0	1	0	0	0	0	0	0	0	1	0	0	0	0	0	8
**3.207**	0	0	0	0	0	0	0	0	0	1	0	0	1	0	0	0	0	0	0	0	0	1	0	0	0	8
**3.208**	0	0	0	0	0	0	0	0	0	0	0	0	0	0	0	1	0	0	0	1	0	1	0	1	0	8
**3.209**	0	0	0	0	1	0	1	0	1	0	0	1	0	0	0	0	0	0	0	0	0	1	0	1	1	7
**3.210**	0	1	1	1	1	0	1	0	1	0	0	1	1	0	0	0	0	0	0	1	0	1	0	1	1	7
**3.211**	0	1	0	1	1	0	1	0	1	1	0	1	1	0	0	0	0	0	0	1	0	1	0	1	1	7
**3.212**	0	1	0	1	1	0	0	0	1	0	0	1	0	1	0	0	0	0	0	0	0	0	1	0	0	7
**3.213**	0	1	0	0	1	0	1	0	0	0	0	1	0	0	0	0	0	0	0	0	0	0	0	1	0	7
**3.214**	0	1	0	0	1	0	0	0	1	1	0	1	0	1	0	0	0	0	0	1	0	0	0	1	1	7
**3.215**	0	1	0	0	1	0	0	0	1	0	0	1	0	0	0	0	0	0	0	0	0	1	1	1	0	7
**3.216**	0	0	0	1	1	0	1	0	1	1	0	1	0	0	0	0	0	0	0	0	0	0	0	0	0	7
**3.217**	0	0	0	1	0	0	0	0	1	1	0	1	0	0	0	0	0	0	0	1	0	0	0	1	0	7
**3.218**	0	0	0	0	1	0	0	0	1	1	0	1	1	0	0	0	0	0	0	1	0	0	0	1	0	7
**3.219**	0	0	0	0	1	0	0	0	1	0	0	1	0	0	0	0	0	0	0	0	0	0	1	0	1	7
**3.220**	0	0	0	0	0	0	0	0	0	1	0	1	0	0	0	1	0	0	0	1	0	0	0	1	1	7
**3.221**	0	1	1	1	1	0	0	0	1	1	0	1	0	0	0	0	0	0	0	0	0	0	0	0	1	7
**3.222**	0	1	1	0	1	0	1	0	1	0	0	1	0	0	0	0	0	0	0	1	0	0	0	1	1	7
**3.223**	0	1	0	1	1	0	1	0	1	0	0	1	0	0	0	0	0	0	0	0	0	0	0	0	1	7
**3.224**	0	1	0	1	1	0	1	0	0	0	0	1	0	0	0	0	0	0	0	1	0	0	1	1	1	7
**3.225**	0	1	0	1	1	0	0	0	1	1	0	1	1	1	0	0	0	0	0	1	0	1	1	0	0	7
**3.226**	0	1	0	1	0	0	0	0	1	0	0	1	0	0	0	0	0	0	0	0	0	1	1	1	1	7
**3.227**	0	1	0	1	0	0	0	0	1	0	0	1	0	0	0	0	0	0	0	0	0	1	1	0	1	7
**3.228**	0	1	0	0	1	0	0	0	1	0	0	1	1	1	0	0	0	0	0	0	0	1	0	1	1	7
**3.229**	0	0	1	1	0	0	0	0	1	0	0	1	0	0	0	0	0	0	0	0	0	1	0	1	1	7
**3.230**	0	0	1	0	1	0	0	0	1	1	0	1	0	0	0	0	0	0	0	0	0	0	0	0	0	7
**3.231**	0	0	0	1	1	0	0	0	1	0	0	1	0	0	0	0	0	0	0	0	0	0	0	1	0	7
**3.232**	0	0	0	1	1	0	0	0	0	1	0	1	0	0	0	0	0	0	0	1	0	1	0	0	1	7
**3.233**	0	0	0	1	0	0	1	0	1	0	0	1	0	0	0	0	0	0	0	0	0	0	0	1	1	7
**3.234**	0	0	0	1	0	0	1	0	0	1	0	1	0	0	0	0	0	0	0	1	0	0	0	1	1	7
**3.235**	0	0	0	1	0	0	0	0	0	0	0	1	0	0	0	0	0	0	0	1	0	1	0	1	0	7
**3.236**	0	0	0	0	1	0	0	0	1	1	0	1	1	0	0	0	0	0	0	1	0	1	0	0	1	7
**3.237**	0	0	0	0	1	0	0	0	1	1	0	1	0	0	0	0	0	0	0	1	0	0	0	1	0	7
**3.238**	0	0	0	0	1	0	0	0	1	0	0	1	1	0	0	0	0	0	0	0	0	1	0	1	0	7
**3.239**	0	0	0	0	0	0	0	0	1	0	0	0	0	1	0	0	0	0	0	1	0	1	0	1	1	7
**3.240**	0	1	1	1	1	0	1	0	0	0	0	1	0	0	0	0	0	0	0	0	0	0	0	1	1	7
**3.241**	0	1	1	0	0	0	1	0	1	1	0	1	0	0	0	0	0	0	0	1	0	0	0	1	1	7
**3.242**	0	1	0	1	1	0	0	0	1	1	0	1	1	0	0	0	0	0	0	1	0	1	0	0	1	7
**3.243**	0	1	0	1	1	0	0	0	0	1	0	0	1	0	0	0	0	0	0	1	0	0	0	1	1	7
**3.244**	0	1	0	1	0	0	0	0	1	0	0	1	0	0	0	0	0	0	0	0	0	0	1	1	0	7
**3.245**	0	1	0	0	1	0	0	0	1	1	0	1	1	0	0	0	0	0	0	0	0	1	1	0	0	7
**3.246**	0	1	0	0	1	0	0	0	1	1	0	1	0	1	0	0	0	0	0	1	0	0	0	0	1	7
**3.247**	0	1	0	0	1	0	0	0	1	1	0	1	0	1	0	0	0	0	0	0	0	0	1	0	0	7
**3.248**	0	1	0	0	0	0	1	0	0	1	0	1	0	1	0	0	0	0	0	1	0	0	0	1	1	7
**3.249**	0	0	1	1	0	0	0	0	1	1	0	1	0	1	0	0	0	0	0	1	0	1	0	0	0	7
**3.250**	0	0	1	0	1	0	0	0	1	1	0	1	1	0	0	0	0	0	0	1	0	0	0	0	0	7
**3.251**	0	0	1	0	0	0	0	0	0	0	0	1	0	1	0	0	0	0	0	0	0	0	1	1	1	7
**3.252**	0	0	1	0	0	0	0	0	0	0	0	0	1	1	0	0	0	0	0	1	0	0	0	1	1	7
**3.253**	0	0	1	0	0	0	0	0	0	0	0	0	0	0	0	0	0	0	0	1	0	0	1	0	1	7
**3.254**	0	0	0	1	1	0	0	0	0	1	0	1	0	1	0	0	0	0	0	1	0	0	0	0	0	7
**3.255**	0	0	0	1	1	0	0	0	0	0	0	1	0	0	0	0	0	0	0	1	0	0	0	1	0	7
**3.256**	0	0	0	1	0	0	0	0	1	0	0	1	0	0	0	0	0	0	0	1	0	0	1	0	0	7
**3.257**	0	0	0	1	0	0	0	0	0	0	0	1	1	0	0	0	0	0	0	1	0	0	0	0	0	7
**3.258**	0	0	0	0	1	0	0	0	1	1	0	1	0	0	0	0	0	0	0	0	0	1	1	0	0	7
**3.259**	0	0	0	0	1	0	0	0	0	0	0	1	1	0	0	0	0	0	0	1	0	0	1	0	0	7
**3.260**	0	0	0	0	0	0	0	0	0	1	0	0	1	1	0	1	0	0	0	0	0	0	0	1	0	7
**3.261**	0	1	0	1	0	0	0	0	1	0	0	1	0	0	0	0	0	0	0	1	0	1	1	0	0	7
**3.262**	0	1	0	0	1	0	0	0	0	1	0	1	1	1	0	0	0	0	0	0	0	1	1	0	0	7
**3.263**	0	1	0	0	1	0	0	0	0	1	0	1	0	1	0	0	0	0	0	1	0	1	0	1	1	7
**3.264**	0	1	0	0	0	0	1	0	0	1	0	1	1	0	0	0	0	0	0	1	0	0	1	0	0	7
**3.265**	0	1	0	0	0	0	0	0	1	0	0	1	1	0	0	0	0	0	0	1	0	1	1	1	1	7
**3.266**	0	1	0	0	0	0	0	0	1	0	0	1	1	0	0	0	0	0	0	1	0	1	0	0	0	7
**3.267**	0	0	1	0	1	0	1	0	1	0	0	0	0	0	0	0	0	0	0	1	0	0	0	1	1	7
**3.268**	0	0	1	0	0	0	0	0	0	1	0	0	0	0	0	0	0	0	0	1	0	1	0	1	1	7
**3.269**	0	0	1	0	0	0	0	0	0	0	0	1	0	0	0	0	0	0	0	1	0	0	1	0	0	7
**3.270**	0	0	0	1	0	0	0	0	0	0	0	1	0	1	0	0	0	0	0	0	0	0	0	1	0	7
**3.271**	0	0	0	0	1	0	1	0	0	1	0	1	0	1	0	0	0	0	0	1	0	0	0	0	1	7
**3.272**	0	0	0	0	0	0	0	0	0	0	0	0	1	1	0	1	0	0	0	1	0	0	0	0	0	7
**3.273**	0	1	1	0	1	0	1	0	0	0	0	0	0	0	0	0	0	0	0	0	0	0	0	1	1	7
**3.274**	0	1	1	0	1	0	0	0	1	1	0	1	1	1	0	0	0	0	0	1	0	0	0	1	0	7
**3.275**	0	1	1	0	0	0	0	0	1	1	0	1	1	1	0	0	0	0	0	1	0	0	0	1	0	7
**3.276**	0	1	0	1	1	0	0	0	0	0	0	1	1	0	0	0	0	0	0	0	0	0	1	1	0	7
**3.277**	0	1	0	1	0	0	1	0	0	0	0	1	0	0	0	0	0	0	0	1	0	0	0	1	1	7
**3.278**	0	1	0	0	1	0	0	0	0	1	0	1	0	0	0	0	0	0	0	0	0	1	1	1	0	7
**3.279**	0	1	0	0	0	0	0	0	1	1	0	1	1	0	0	0	0	0	0	0	0	1	0	0	0	7
**3.280**	0	1	0	0	0	0	0	0	0	1	0	1	1	1	0	0	0	0	0	1	0	0	1	0	0	7
**3.281**	0	1	0	0	0	0	0	0	0	0	0	0	0	1	0	0	0	0	0	0	0	1	1	1	1	7
**3.282**	0	0	1	1	0	0	0	0	0	0	0	1	0	0	0	0	0	0	0	1	0	0	0	1	0	7
**3.283**	0	0	1	1	0	0	0	0	0	0	0	0	1	1	0	0	0	0	0	1	0	1	0	0	0	7
**3.284**	0	0	1	0	1	0	0	0	0	1	0	1	1	0	0	0	0	0	0	0	0	0	0	0	0	7
**3.285**	0	0	1	0	0	0	0	0	0	0	0	0	1	1	0	1	0	0	0	1	0	0	0	0	0	7
**3.286**	0	0	0	1	0	0	0	0	1	1	0	1	0	1	0	0	0	0	0	1	0	1	0	0	0	7
**3.287**	0	0	0	1	0	0	0	0	0	0	0	0	0	1	0	0	0	0	0	1	0	1	0	0	0	7
**3.288**	0	0	0	1	0	0	0	0	0	0	0	0	0	0	0	1	0	0	0	0	0	1	0	0	0	7
**3.289**	0	0	0	0	1	0	0	0	0	1	0	1	1	1	0	0	0	0	0	1	0	1	0	0	0	7
**3.290**	0	0	0	0	1	0	0	0	0	1	0	1	1	0	0	1	0	0	0	0	0	0	0	0	0	7
**3.291**	0	0	0	0	0	0	1	0	1	0	0	0	1	1	0	0	0	0	0	1	0	1	0	0	0	7
**3.292**	0	0	0	0	0	0	0	0	0	1	0	0	1	0	0	0	0	0	0	1	0	1	0	0	0	7
**3.293**	0	1	1	0	1	0	0	0	1	1	0	1	1	0	0	0	0	0	0	1	0	0	0	0	1	7

CMP	CD2	CD3	CD4	CD7	CD8	CD10	CD13	CD18	CD26	CD29	CD36	CD44	CD45RA	CD49f	CD54	CD56	CD57	CD58	CD62L	CD71	CD80	Cytokeratin	Histone_H1	HLA-DQ	HLA-DR	Freq. (voxel)
**3.294**	0	1	1	0	0	0	0	0	1	1	0	1	0	0	0	0	0	0	0	0	0	0	1	0	0	7
**3.295**	0	1	0	1	1	0	0	0	0	1	0	1	1	1	0	0	0	0	0	1	0	0	0	1	1	7
**3.296**	0	1	0	1	1	0	0	0	0	0	0	0	1	0	0	0	0	0	0	0	0	0	1	0	1	7
**3.297**	0	1	0	1	0	0	0	0	0	1	0	1	1	1	0	1	0	0	0	0	0	0	0	0	0	7
**3.298**	0	1	0	0	1	0	0	0	0	1	0	1	1	0	0	0	0	0	0	0	0	0	1	1	0	7
**3.299**	0	1	0	0	0	0	0	0	0	0	0	1	1	0	0	0	0	0	0	0	0	0	1	0	1	7
**3.300**	0	0	1	0	1	0	0	0	1	0	0	1	0	0	0	0	0	0	0	1	0	1	0	0	1	7
**3.301**	0	0	1	0	0	0	1	0	1	1	0	1	0	0	0	0	0	0	0	0	0	0	0	0	0	7
**3.302**	0	0	1	0	0	0	1	0	0	0	0	0	1	0	0	1	0	0	0	0	0	1	0	0	0	7
**3.303**	0	0	1	0	0	0	0	0	0	0	0	0	1	1	0	1	0	0	0	0	0	0	0	0	0	7
**3.304**	0	0	0	1	1	0	0	0	0	1	0	0	0	0	0	0	0	0	0	0	0	0	1	0	0	7
**3.305**	0	0	0	1	0	0	0	0	0	0	0	1	0	1	0	0	0	0	0	0	0	1	0	1	0	7
**3.306**	0	0	0	0	0	0	1	0	1	0	1	1	0	1	0	0	0	0	0	1	0	1	0	0	0	7
**3.307**	0	1	1	1	1	0	0	0	1	1	0	1	0	0	0	0	0	0	0	1	0	0	0	1	1	7
**3.308**	0	1	1	0	1	0	0	0	0	0	0	0	1	0	0	0	0	0	0	0	0	0	0	0	0	7
**3.309**	0	1	0	1	0	0	0	0	0	1	0	1	0	1	0	0	0	0	0	0	0	0	0	1	0	7
**3.310**	0	1	0	0	0	0	0	0	0	0	0	0	0	1	0	0	0	0	0	1	0	0	0	1	1	7
**3.311**	0	0	1	0	1	0	0	0	0	0	0	0	0	0	0	0	0	0	0	0	0	1	0	0	0	7
**3.312**	0	0	1	0	0	0	1	0	1	1	0	1	0	1	0	0	0	0	0	0	0	1	0	1	1	7
**3.313**	0	0	1	0	0	0	1	0	0	1	0	1	0	1	0	0	0	0	0	0	0	1	0	1	1	7
**3.314**	0	0	1	0	0	0	1	0	0	1	0	0	0	0	0	0	0	0	0	0	0	0	1	0	0	7
**3.315**	0	0	1	0	0	0	0	0	1	0	0	0	0	1	0	0	0	0	0	0	0	0	0	1	0	7
**3.316**	0	0	0	1	0	0	0	0	0	1	0	1	0	0	0	0	0	0	0	0	0	1	0	1	0	7
**3.317**	0	0	0	0	0	0	1	0	0	1	0	0	0	1	0	0	0	0	0	0	0	0	1	1	0	7
**3.318**	0	0	0	0	0	0	1	0	0	0	0	0	1	0	0	0	0	0	0	0	0	0	1	1	0	7
**3.319**	0	0	0	0	0	0	0	0	0	0	1	1	0	1	0	0	0	0	0	1	0	1	0	1	0	7
**3.320**	0	0	0	0	0	0	0	0	0	0	0	0	1	1	0	1	0	0	0	0	0	1	0	0	0	7
**3.321**	0	1	1	1	0	0	0	0	0	0	0	0	0	0	0	0	0	0	0	1	0	0	0	0	0	7
**3.322**	0	1	1	0	1	0	0	0	1	1	0	1	1	0	0	0	0	0	0	0	0	0	0	1	1	7
**3.323**	0	1	1	0	0	0	0	0	1	1	0	1	0	0	0	0	0	0	0	1	0	0	0	0	0	7
**3.324**	0	1	0	1	1	0	0	0	0	0	0	0	0	0	0	0	0	0	0	0	0	1	1	0	0	7
**3.325**	0	1	0	0	0	0	0	0	0	0	0	1	0	1	0	0	0	0	0	1	0	0	1	0	1	7
**3.326**	0	0	1	0	1	0	0	0	0	1	0	1	0	0	0	0	0	0	0	0	0	0	0	1	0	7
**3.327**	0	0	1	0	0	0	1	0	1	0	0	1	0	1	0	0	0	0	0	0	0	1	0	0	0	7
**3.328**	0	0	1	0	0	0	0	0	0	0	0	1	1	0	0	0	0	0	0	0	0	0	0	1	0	7
**3.329**	0	0	1	0	0	0	0	0	0	0	0	0	1	1	0	0	0	0	0	0	0	0	0	1	1	7
**3.330**	0	0	0	1	0	0	0	0	1	0	0	0	0	0	0	0	0	0	0	0	0	0	1	0	0	7
**3.331**	0	0	0	1	0	0	0	0	0	0	0	0	1	0	0	0	0	0	0	1	0	1	0	0	0	7
**3.332**	0	0	0	0	1	0	1	0	1	1	0	1	0	0	0	0	0	0	0	0	0	0	0	1	1	7
**3.333**	0	0	0	0	1	0	1	0	0	1	0	1	1	1	0	0	0	0	0	0	0	1	0	0	0	7
**3.334**	0	0	0	0	1	0	0	0	0	0	0	0	1	0	0	0	0	0	0	0	0	0	0	1	0	7
**3.335**	0	0	0	0	0	0	0	0	0	1	0	1	0	1	0	1	0	0	0	0	0	1	1	0	0	7
**3.336**	0	1	1	1	0	0	0	0	1	1	0	1	0	1	0	0	0	0	0	0	0	0	0	0	0	7
**3.337**	0	1	0	1	1	0	0	0	0	0	0	0	0	0	0	0	0	0	0	1	0	1	1	0	1	7
**3.338**	0	1	0	1	1	0	0	0	0	0	0	0	0	0	0	0	0	0	0	0	0	0	1	1	0	7
**3.339**	0	1	0	0	0	0	0	0	1	1	1	1	0	0	0	0	0	0	0	0	0	0	0	1	1	7
**3.340**	0	1	0	0	0	0	0	0	0	1	0	0	1	0	0	0	0	0	0	0	0	0	0	0	0	7
**3.341**	0	0	1	1	0	0	1	0	0	1	1	1	1	1	0	1	0	0	0	0	0	0	0	1	1	7
**3.342**	0	0	1	1	0	0	0	0	0	1	0	1	0	1	0	0	0	0	0	1	0	1	0	0	0	7
**3.343**	0	0	1	0	0	0	0	0	0	1	0	0	0	1	0	1	0	0	0	0	0	0	0	1	0	7
**3.344**	0	0	1	0	0	0	0	0	0	1	0	0	0	1	0	0	0	0	0	1	0	1	0	0	0	7
**3.345**	0	0	0	1	0	0	0	0	0	1	0	0	0	0	0	0	0	0	0	0	0	0	0	1	0	7
**3.346**	0	1	0	1	1	0	0	0	0	0	0	1	0	1	0	0	0	0	0	0	0	0	0	0	1	7
**3.347**	0	1	0	1	0	0	0	0	0	0	0	1	1	0	0	0	0	0	0	0	0	0	0	0	1	7
**3.348**	0	1	0	0	1	0	0	0	1	1	0	1	0	0	0	0	0	0	0	0	0	1	0	0	1	7
**3.349**	0	1	0	0	0	0	0	0	0	1	1	1	0	0	0	0	0	0	0	0	0	0	0	1	0	7
**3.350**	0	0	1	0	0	0	1	0	1	1	0	1	1	1	0	1	0	0	0	1	0	0	0	1	1	7
**3.351**	0	0	1	0	0	0	0	0	1	0	0	1	0	0	0	0	0	0	0	0	0	0	0	1	0	7
**3.352**	0	0	0	1	0	0	1	0	0	1	0	1	0	1	0	0	0	0	0	1	0	1	0	1	1	7
**3.353**	0	0	0	0	1	0	0	0	0	1	0	1	1	1	0	0	0	0	0	0	0	1	1	0	1	7
**3.354**	0	0	0	0	0	0	1	0	1	1	0	1	0	1	0	0	0	0	0	1	0	0	1	0	0	7
**3.355**	0	0	0	0	0	0	1	0	0	1	1	1	0	1	0	0	0	0	0	1	0	0	0	0	1	7
**3.356**	0	0	0	0	0	0	0	0	0	0	1	1	0	0	0	1	0	0	0	0	0	0	0	1	1	7
**3.357**	0	1	0	1	1	0	0	0	1	1	0	1	1	0	0	0	0	0	0	0	0	1	0	0	1	7
**3.358**	0	1	0	0	1	0	0	0	0	0	0	1	0	1	0	0	0	0	0	1	0	0	1	0	0	7
**3.359**	0	1	0	0	0	0	0	0	0	1	0	1	0	1	0	0	0	0	0	0	0	0	1	1	1	7
**3.360**	0	0	1	0	0	0	1	0	1	1	0	1	0	1	0	0	0	0	0	0	0	0	1	1	1	7
**3.361**	0	0	1	0	0	0	0	0	0	0	0	1	1	1	0	0	0	0	0	0	0	1	0	1	0	7
**3.362**	0	0	1	0	0	0	0	0	0	0	0	0	0	1	0	0	0	0	0	0	0	0	1	1	1	7
**3.363**	0	0	0	0	0	0	1	0	1	1	1	1	0	1	0	0	0	0	0	1	0	0	0	1	1	7
**3.364**	0	0	0	0	0	0	1	0	1	0	0	0	0	0	0	0	0	0	0	0	0	0	1	1	0	7
**3.365**	0	0	0	0	0	0	0	0	0	1	1	1	0	0	0	0	0	0	0	1	0	1	0	1	0	7
**3.366**	0	0	0	0	0	0	0	0	0	0	0	1	1	1	0	0	0	0	0	0	0	1	0	0	0	7
**3.367**	0	0	0	0	0	0	0	0	0	0	0	1	1	0	0	0	0	0	0	0	0	1	0	1	0	7
**3.368**	0	0	0	0	0	0	0	0	0	0	0	1	0	0	0	1	0	0	0	0	0	0	1	0	0	7
**3.369**	0	1	1	0	0	0	0	0	1	0	0	1	0	0	0	0	0	0	0	0	0	1	0	0	0	7
**3.370**	0	1	0	1	1	0	0	0	1	1	0	1	0	0	0	0	0	0	0	1	0	0	1	1	0	7
**3.371**	0	1	0	0	1	0	0	0	0	0	0	0	0	0	0	0	0	0	0	0	0	0	0	1	0	7
**3.372**	0	1	0	0	0	0	0	0	0	1	0	1	0	0	0	0	0	0	0	0	0	0	1	1	1	7
**3.373**	0	0	1	0	0	0	0	0	0	0	0	0	0	0	0	1	0	0	0	1	0	1	0	0	0	7
**3.374**	0	0	0	1	0	0	0	0	0	1	0	1	1	0	0	0	0	0	0	0	0	0	0	0	0	7
**3.375**	0	0	0	1	0	0	0	0	0	0	0	0	0	0	0	1	0	0	0	1	0	1	0	0	0	7
**3.376**	0	0	0	0	0	0	1	0	0	1	0	1	1	1	0	0	0	0	0	1	0	0	0	0	0	7
**3.377**	0	0	0	0	0	0	0	0	1	1	0	0	0	0	0	0	0	0	0	0	0	0	0	1	1	7
**3.378**	0	1	0	1	1	0	0	0	0	0	0	1	1	0	0	0	0	0	0	0	0	1	1	0	1	7
**3.379**	0	1	0	0	0	0	0	0	1	1	0	1	0	1	0	0	0	0	0	1	0	0	0	0	0	7
**3.380**	0	1	0	0	0	0	0	0	0	0	0	1	1	0	0	0	0	0	0	0	0	1	0	1	0	7
**3.381**	0	0	1	1	0	0	0	0	0	0	0	0	0	0	0	1	0	0	0	0	0	1	0	0	0	7
**3.382**	0	0	1	0	0	0	1	0	0	1	0	1	0	0	0	0	0	0	0	0	0	0	0	0	1	7
**3.383**	0	0	0	1	0	0	0	0	0	0	0	0	1	0	0	1	0	0	0	0	0	1	0	0	0	7
**3.384**	0	0	0	0	1	0	1	0	1	0	0	0	1	0	0	0	0	0	0	0	0	0	0	1	0	7
**3.385**	0	0	0	0	0	0	1	0	1	0	0	0	1	0	0	1	0	0	0	1	0	0	0	1	0	7
**3.386**	0	0	0	0	0	0	0	0	0	1	0	0	0	1	0	1	0	0	0	0	0	0	0	1	1	7
**3.387**	0	1	0	0	1	0	1	0	0	1	0	1	0	1	0	0	0	0	0	0	0	0	0	0	1	7
**3.388**	0	0	1	1	0	0	0	0	0	1	0	0	0	0	0	0	0	0	0	1	0	0	0	1	1	7
**3.389**	0	0	0	1	0	0	1	0	0	0	0	0	0	0	0	0	0	0	0	0	0	1	0	0	0	7
**3.390**	0	0	0	0	0	0	1	0	0	1	0	1	0	0	0	0	0	0	0	1	0	1	1	0	0	7
**3.391**	0	0	0	0	0	0	0	0	1	0	1	1	0	0	0	0	0	0	0	0	0	1	1	1	1	7
**3.392**	0	1	0	1	0	0	0	0	0	0	0	0	0	0	0	0	0	0	0	1	0	1	0	0	0	7
**3.393**	0	0	0	0	0	0	1	0	0	0	0	0	1	0	0	1	0	0	0	1	0	0	0	1	0	7
**3.394**	0	1	0	1	0	0	0	0	1	0	0	1	0	0	0	0	0	0	0	1	0	0	0	0	1	7
**3.395**	0	1	0	0	0	0	0	0	1	0	0	1	1	0	0	0	0	0	0	0	0	0	1	0	0	7
**3.396**	0	0	0	1	0	0	0	0	0	0	0	0	1	0	0	1	0	0	0	1	0	1	0	1	0	7
**3.397**	0	0	0	0	1	0	0	0	0	0	0	0	1	0	0	1	0	0	0	1	0	0	0	0	0	7
**3.398**	0	0	0	0	0	0	1	0	1	0	1	0	0	0	0	0	0	0	0	1	0	1	0	0	0	7
**3.399**	0	0	0	0	0	0	0	0	0	1	1	0	0	0	0	0	0	0	0	1	0	0	0	1	1	7
**3.400**	0	1	0	1	0	0	0	0	1	0	0	1	0	0	0	0	0	0	0	1	0	0	0	1	0	7
**3.401**	0	1	0	0	1	0	0	0	0	0	0	0	1	0	0	0	0	0	0	0	0	1	1	0	0	7
**3.402**	0	0	0	0	0	0	1	0	1	1	0	0	1	0	0	1	0	0	0	1	0	0	0	0	0	7
**3.403**	0	0	0	0	0	0	0	0	1	0	0	0	0	1	0	0	0	0	0	1	0	0	1	0	0	7
**3.404**	0	0	0	0	0	0	0	0	0	1	1	0	0	0	0	0	0	0	0	1	0	1	0	1	0	7
**3.405**	0	1	0	1	1	0	0	0	1	0	0	0	0	0	0	0	0	0	0	0	0	0	0	0	0	7
**3.406**	0	1	0	0	1	0	0	0	1	0	0	0	0	0	0	0	0	0	0	1	0	0	0	0	0	7
**3.407**	0	0	0	0	0	0	1	0	0	0	0	0	0	0	0	0	0	0	0	1	0	0	1	1	0	7
**3.408**	0	0	0	0	1	0	0	0	1	0	0	0	1	0	0	0	0	0	0	1	0	0	0	0	0	7
**3.409**	0	0	0	0	0	0	0	0	1	0	0	0	0	0	0	1	0	0	0	1	0	1	0	0	0	7
**3.410**	0	0	1	1	0	0	1	0	1	0	0	1	0	0	0	0	0	0	0	1	0	0	0	1	1	6
**3.411**	0	0	0	1	1	0	1	0	1	0	0	1	0	0	0	0	0	0	0	0	0	0	1	1	0	6
**3.412**	0	0	0	1	1	0	0	0	0	1	0	1	1	0	0	0	0	0	0	0	0	0	0	1	0	6
**3.413**	0	0	0	0	0	0	0	0	0	0	0	0	0	1	0	1	0	0	0	0	0	0	1	1	0	6
**3.414**	0	1	1	0	1	0	1	0	1	1	0	1	1	0	0	0	0	0	0	1	0	0	1	0	0	6
**3.415**	0	1	0	1	0	0	0	0	0	0	0	1	0	1	0	0	0	0	0	1	0	1	1	0	0	6

CMP	CD2	CD3	CD4	CD7	CD8	CD10	CD13	CD18	CD26	CD29	CD36	CD44	CD45RA	CD49f	CD54	CD56	CD57	CD58	CD62L	CD71	CD80	Cytokeratin	Histone_H1	HLA-DQ	HLA-DR	Freq. (voxel)
**3.416**	0	1	0	1	0	0	0	0	0	0	0	0	1	0	0	0	0	0	0	1	0	0	0	0	1	6
**3.417**	0	1	0	0	1	0	0	0	1	1	0	1	1	0	0	0	0	0	0	1	0	1	1	1	1	6
**3.418**	0	1	0	0	1	0	0	0	1	0	0	1	1	0	0	0	0	0	0	0	0	1	1	1	0	6
**3.419**	0	1	0	0	1	0	0	0	0	0	0	1	1	1	0	0	0	0	0	0	0	1	0	1	1	6
**3.420**	0	0	1	1	0	0	0	0	0	0	0	1	0	0	0	0	0	0	0	0	0	1	0	1	1	6
**3.421**	0	0	1	0	0	0	0	0	1	0	0	1	0	1	0	0	0	0	0	0	0	1	0	1	0	6
**3.422**	0	0	0	1	1	0	0	0	0	0	0	1	0	0	0	0	0	0	0	1	0	0	1	1	1	6
**3.423**	0	0	0	1	0	0	1	0	0	1	0	1	0	0	0	0	0	0	0	0	0	0	0	1	0	6
**3.424**	0	0	0	1	0	0	1	0	0	0	0	1	0	0	0	0	0	0	0	0	0	0	0	1	0	6
**3.425**	0	0	0	1	0	0	0	0	1	0	0	1	0	1	0	0	0	0	0	0	0	1	0	1	1	6
**3.426**	0	0	0	1	0	0	0	0	0	1	0	0	0	1	0	0	0	0	0	0	0	1	0	1	1	6
**3.427**	0	0	0	0	1	0	1	0	1	0	0	1	0	0	0	0	0	0	0	1	0	1	0	1	1	6
**3.428**	0	0	0	0	1	0	0	0	1	1	0	1	1	0	0	0	0	0	0	0	0	0	0	1	1	6
**3.429**	0	0	0	0	1	0	0	0	1	1	0	1	0	1	0	0	0	0	0	0	0	0	0	1	0	6
**3.430**	0	0	0	0	1	0	0	0	0	1	0	1	0	1	0	0	0	0	0	0	0	0	0	1	0	6
**3.431**	0	0	0	0	0	0	0	0	1	0	0	0	1	0	0	0	0	0	0	0	0	1	0	1	1	6
**3.432**	0	0	0	0	0	0	0	0	0	1	0	0	1	0	0	0	0	0	0	0	0	1	0	1	0	6
**3.433**	0	1	1	0	1	0	0	0	1	0	0	1	1	0	0	0	0	0	0	0	0	1	0	0	1	6
**3.434**	0	1	0	1	1	0	1	0	0	1	0	1	0	0	0	0	0	0	0	0	0	0	1	1	1	6
**3.435**	0	1	0	1	1	0	0	0	1	1	0	1	0	0	0	0	0	0	0	1	0	1	1	0	0	6
**3.436**	0	1	0	0	1	0	0	0	1	1	0	1	1	0	0	0	0	0	0	1	0	1	0	0	1	6
**3.437**	0	1	0	0	1	0	0	0	1	0	0	1	0	1	0	0	0	0	0	0	0	0	0	0	0	6
**3.438**	0	0	1	0	0	0	1	0	1	1	0	1	1	0	0	0	0	0	0	1	0	0	0	1	1	6
**3.439**	0	0	1	0	0	0	0	0	1	0	0	1	0	1	0	0	0	0	0	1	0	0	0	0	1	6
**3.440**	0	0	0	1	1	0	0	0	1	1	0	1	0	0	0	0	0	0	0	1	0	0	0	0	1	6
**3.441**	0	0	0	1	1	0	0	0	0	0	0	1	0	0	0	0	0	0	0	1	0	0	1	0	1	6
**3.442**	0	0	0	1	1	0	0	0	0	0	0	1	0	0	0	0	0	0	0	0	0	1	1	0	0	6
**3.443**	0	0	0	1	0	0	0	0	1	1	0	1	1	0	0	0	0	0	0	1	0	0	0	1	1	6
**3.444**	0	0	0	1	0	0	0	0	1	1	0	1	0	0	0	0	0	0	0	1	0	0	0	1	1	6
**3.445**	0	0	0	1	0	0	0	0	0	1	0	1	1	1	0	0	0	0	0	1	0	1	0	1	0	6
**3.446**	0	0	0	0	1	0	0	0	1	1	0	1	0	0	0	0	0	0	0	1	0	1	0	0	1	6
**3.447**	0	0	0	0	1	0	0	0	1	0	0	1	1	0	0	0	0	0	0	0	0	1	0	1	1	6
**3.448**	0	0	0	0	1	0	0	0	1	0	0	0	0	0	0	0	0	0	0	1	0	1	0	1	1	6
**3.449**	0	0	0	0	0	0	0	0	1	0	0	1	0	0	0	0	0	0	0	1	0	0	1	1	0	6
**3.450**	0	1	1	0	1	0	0	0	1	1	0	1	1	0	0	0	0	0	0	1	0	0	1	0	0	6
**3.451**	0	1	1	0	0	0	0	0	1	0	0	1	1	0	0	0	0	0	0	1	0	1	0	1	1	6
**3.452**	0	1	0	1	1	0	0	0	0	0	0	1	1	0	0	0	0	0	0	1	0	1	1	1	1	6
**3.453**	0	1	0	1	0	0	1	0	0	0	0	1	1	1	0	0	0	0	0	1	0	1	0	1	1	6
**3.454**	0	1	0	0	1	0	0	0	0	1	0	1	1	0	0	0	0	0	0	1	0	1	0	1	1	6
**3.455**	0	1	0	0	0	0	1	0	0	1	0	1	0	1	0	0	0	0	0	0	0	0	0	1	1	6
**3.456**	0	1	0	0	0	0	1	0	0	0	0	1	0	1	0	0	0	0	0	1	0	0	0	1	1	6
**3.457**	0	1	0	0	0	0	1	0	0	0	0	0	0	0	0	0	0	0	0	0	0	0	1	0	1	6
**3.458**	0	0	1	0	1	0	0	0	0	1	0	1	0	0	0	0	0	0	0	1	0	1	0	0	0	6
**3.459**	0	0	1	0	0	0	0	0	0	1	0	1	0	0	0	0	0	0	0	0	0	1	1	0	0	6
**3.460**	0	0	0	1	1	0	0	0	0	0	0	1	1	0	0	0	0	0	0	0	0	0	1	0	0	6
**3.461**	0	0	0	0	1	0	1	0	0	1	0	1	0	1	0	0	0	0	0	1	0	0	0	1	1	6
**3.462**	0	0	0	0	1	0	0	0	1	1	0	1	1	0	0	0	0	0	0	1	0	1	0	0	0	6
**3.463**	0	0	0	0	0	0	1	0	0	0	0	0	1	1	0	0	0	0	0	1	0	0	0	0	0	6
**3.464**	0	0	0	0	0	0	0	0	1	0	0	1	1	0	0	0	0	0	0	1	0	1	0	0	0	6
**3.465**	0	0	0	0	0	0	0	0	0	1	0	1	0	0	0	1	0	0	0	0	0	0	1	1	0	6
**3.466**	0	1	1	1	1	0	0	0	1	1	0	1	1	0	0	0	0	0	0	0	0	0	0	0	0	6
**3.467**	0	1	1	0	1	0	0	0	1	0	0	1	1	0	0	0	0	0	0	1	0	0	0	0	0	6
**3.468**	0	1	1	0	1	0	0	0	0	1	0	1	1	0	0	0	0	0	0	1	0	0	0	0	0	6
**3.469**	0	1	1	0	0	0	0	0	1	0	0	1	0	0	0	0	0	0	0	0	0	0	1	0	0	6
**3.470**	0	1	0	1	0	0	0	0	1	1	0	0	1	0	0	0	0	0	0	1	0	0	1	0	0	6
**3.471**	0	1	0	0	1	0	0	0	1	0	0	1	1	0	0	0	0	0	0	1	0	0	1	1	0	6
**3.472**	0	1	0	0	0	0	1	0	0	0	0	1	0	1	0	0	0	0	0	0	0	0	0	1	1	6
**3.473**	0	1	0	0	0	0	0	0	1	1	0	1	1	0	0	0	0	0	0	1	0	1	0	1	0	6
**3.474**	0	0	1	1	0	0	0	0	1	1	0	1	0	0	0	0	0	0	0	0	0	0	0	1	1	6
**3.475**	0	0	1	1	0	0	0	0	0	1	0	1	0	0	0	0	0	0	0	1	0	0	0	1	0	6
**3.476**	0	0	1	0	1	0	0	0	0	0	0	1	0	0	0	0	0	0	0	1	0	0	1	0	0	6
**3.477**	0	0	1	0	0	0	1	0	0	0	0	0	1	0	0	1	0	0	0	1	0	0	0	0	0	6
**3.478**	0	0	1	0	0	0	1	0	0	0	0	0	0	0	0	0	0	0	0	1	0	1	0	1	0	6
**3.479**	0	0	1	0	0	0	0	0	1	0	0	1	0	0	0	0	0	0	0	0	0	0	0	0	1	6
**3.480**	0	0	1	0	0	0	0	0	0	0	0	0	0	0	0	0	0	0	0	1	0	0	1	1	0	6
**3.481**	0	0	0	1	1	0	0	0	0	1	0	1	0	1	0	0	0	0	0	1	0	1	0	1	1	6
**3.482**	0	0	0	1	0	0	1	0	0	1	0	1	0	1	0	0	0	0	0	1	0	0	1	1	1	6
**3.483**	0	0	0	0	1	0	1	0	1	1	0	1	0	1	0	0	0	0	0	1	0	0	0	1	0	6
**3.484**	0	1	1	1	0	0	1	0	0	0	0	1	1	0	0	0	0	0	0	1	0	0	1	0	0	6
**3.485**	0	1	1	0	1	0	0	0	1	0	0	1	0	0	0	0	0	0	0	1	0	1	0	0	1	6
**3.486**	0	1	0	1	1	0	0	0	1	0	0	0	1	0	0	0	0	0	0	0	0	0	0	0	1	6
**3.487**	0	1	0	1	0	0	1	0	0	1	0	1	0	0	0	0	0	0	0	0	0	0	0	0	0	6
**3.488**	0	1	0	1	0	0	0	0	0	0	0	0	0	0	0	0	0	0	0	0	0	0	1	1	0	6
**3.489**	0	1	0	0	1	0	0	0	0	1	0	1	1	0	0	0	0	0	0	1	0	0	1	1	0	6
**3.490**	0	1	0	0	1	0	0	0	0	1	0	1	0	0	0	0	0	0	0	1	0	0	1	1	0	6
**3.491**	0	1	0	0	1	0	0	0	0	0	0	0	1	0	0	0	0	0	0	1	0	0	0	0	1	6
**3.492**	0	0	1	1	0	0	0	0	1	0	0	1	0	0	0	0	0	0	0	1	0	1	0	0	0	6
**3.493**	0	0	1	0	0	0	1	0	0	0	0	0	1	0	0	0	0	0	0	1	0	0	0	1	1	6
**3.494**	0	0	1	0	0	0	0	0	0	0	0	0	1	1	0	0	0	0	0	1	0	1	0	0	0	6
**3.495**	0	0	0	1	1	0	1	0	0	0	0	1	0	0	0	0	0	0	0	0	0	0	1	0	1	6
**3.496**	0	0	0	0	1	0	0	0	0	1	0	1	0	1	0	0	0	0	0	1	0	1	1	0	0	6
**3.497**	0	0	0	0	0	0	1	0	1	0	0	0	0	0	0	0	0	0	0	0	0	1	0	0	1	6
**3.498**	0	0	0	0	0	0	1	0	0	0	0	0	1	1	0	1	0	0	0	1	0	0	0	0	0	6
**3.499**	0	0	0	0	0	0	1	0	0	0	0	0	0	1	0	0	0	0	0	1	0	1	0	1	1	6
**3.500**	0	0	0	0	0	0	0	0	1	0	0	1	1	0	0	0	0	0	0	0	0	1	0	1	1	6
**3.501**	0	0	0	0	0	0	0	0	0	0	0	1	1	0	0	0	0	0	0	1	0	1	0	1	1	6
**3.502**	0	1	1	1	0	0	0	0	0	1	0	1	0	1	0	0	0	0	0	1	0	0	0	0	1	6
**3.503**	0	1	0	1	0	0	0	0	0	0	0	1	0	1	0	0	0	0	0	0	0	1	0	1	0	6
**3.504**	0	1	0	1	0	0	0	0	0	0	0	0	0	1	0	0	0	0	0	0	0	1	1	1	0	6
**3.505**	0	1	0	0	1	0	0	0	1	0	0	1	1	0	0	0	0	0	0	1	0	1	0	0	0	6
**3.506**	0	1	0	0	1	0	0	0	0	1	0	1	1	0	0	0	0	0	0	1	0	0	1	1	1	6
**3.507**	0	1	0	0	0	0	0	0	0	1	0	1	0	0	0	0	0	0	0	1	0	1	1	0	1	6
**3.508**	0	1	0	0	0	0	0	0	0	1	0	1	0	0	0	0	0	0	0	1	0	1	0	0	1	6
**3.509**	0	1	0	0	0	0	0	0	0	0	0	1	0	1	0	0	0	0	0	0	0	1	1	1	1	6
**3.510**	0	0	1	0	1	0	0	0	1	0	0	1	0	0	0	0	0	0	0	1	0	1	0	0	0	6
**3.511**	0	0	1	0	0	0	1	0	0	1	0	0	0	0	0	0	0	0	0	0	0	1	1	0	0	6
**3.512**	0	0	1	0	0	0	1	0	0	0	0	0	1	1	0	1	0	0	0	0	0	0	0	0	0	6
**3.513**	0	0	0	0	1	0	1	0	0	0	0	0	0	1	0	0	0	0	0	0	0	0	0	0	0	6
**3.514**	0	0	0	0	0	0	1	0	1	0	1	1	0	0	0	0	0	0	0	1	0	1	0	0	0	6
**3.515**	0	0	0	0	0	0	1	0	1	0	0	0	1	1	0	0	0	0	0	0	0	1	0	0	0	6
**3.516**	0	0	0	0	0	0	1	0	0	0	0	0	1	0	0	1	0	0	0	0	0	1	1	0	0	6
**3.517**	0	0	0	0	0	0	0	0	0	1	0	0	0	0	0	1	0	0	0	0	0	0	0	1	0	6
**3.518**	0	0	0	0	0	0	0	0	0	0	0	0	1	0	0	1	0	0	0	0	0	1	1	0	0	6
**3.519**	0	1	1	0	0	0	0	0	0	1	0	1	0	1	0	0	0	0	0	0	0	0	0	1	0	6
**3.520**	0	1	0	0	1	0	0	0	1	0	0	0	0	0	0	0	0	0	0	0	0	0	1	1	1	6
**3.521**	0	1	0	0	1	0	0	0	0	0	0	1	0	1	0	0	0	0	0	0	0	1	0	0	1	6
**3.522**	0	0	1	1	0	0	0	0	1	0	0	1	0	1	0	0	0	0	0	1	0	1	0	0	1	6
**3.523**	0	0	1	0	1	0	1	0	0	0	0	1	0	0	0	0	0	0	0	0	0	0	0	0	0	6
**3.524**	0	0	1	0	0	0	1	0	1	0	1	0	0	1	0	0	0	0	0	0	0	1	0	0	0	6
**3.525**	0	0	1	0	0	0	1	0	0	0	1	1	0	1	0	0	0	0	0	1	0	1	0	1	1	6
**3.526**	0	0	1	0	0	0	1	0	0	0	0	0	1	0	0	0	0	0	0	0	0	0	0	0	0	6
**3.527**	0	0	1	0	0	0	0	0	0	0	0	1	0	0	0	1	0	0	0	0	0	1	0	0	0	6
**3.528**	0	0	0	1	0	0	0	0	1	0	0	1	0	0	0	0	0	0	0	0	0	0	0	0	1	6
**3.529**	0	0	0	0	0	0	1	0	1	0	0	0	0	1	0	0	0	0	0	1	0	1	0	1	1	6
**3.530**	0	0	0	0	0	0	1	0	0	0	1	1	0	0	0	0	0	0	0	0	0	1	0	1	0	6
**3.531**	0	0	0	0	0	0	1	0	0	0	0	0	0	0	0	1	0	0	0	0	0	0	0	0	1	6
**3.532**	0	0	0	0	0	0	0	0	0	1	0	1	1	0	0	0	0	0	0	0	0	0	0	1	1	6
**3.533**	0	0	0	0	0	0	0	0	0	0	1	1	0	1	0	0	0	0	0	1	0	1	0	1	1	6
**3.534**	0	0	0	0	0	0	0	0	0	0	0	0	1	1	0	0	0	0	0	0	0	0	0	1	1	6
**3.535**	0	1	1	1	0	0	0	0	1	0	0	1	0	0	0	0	0	0	0	0	0	0	0	1	0	6
**3.536**	0	1	0	1	0	0	0	0	1	1	1	1	0	0	0	0	0	0	0	0	0	0	0	1	1	6
**3.537**	0	1	0	0	1	0	1	0	0	1	0	1	0	1	0	0	0	0	0	0	0	1	1	1	1	6

CMP	CD2	CD3	CD4	CD7	CD8	CD10	CD13	CD18	CD26	CD29	CD36	CD44	CD45RA	CD49f	CD54	CD56	CD57	CD58	CD62L	CD71	CD80	Cytokeratin	Histone_H1	HLA-DQ	HLA-DR	Freq. (voxel)
3.538	0	1	0	0	1	0	0	0	1	0	0	1	1	0	0	0	0	0	0	0	0	0	1	1	0	6
3.539	0	1	0	0	1	0	0	0	1	0	0	0	1	0	0	0	0	0	0	0	0	0	1	0	1	6
3.540	0	0	1	0	1	0	0	0	1	0	0	1	1	0	0	0	0	0	0	1	0	0	0	1	1	6
3.541	0	0	1	0	0	0	1	0	1	1	0	1	0	1	0	0	0	0	0	0	0	1	0	0	1	6
3.542	0	0	1	0	0	0	1	0	1	0	0	1	0	0	0	0	0	0	0	0	0	0	0	1	0	6
3.543	0	0	1	0	0	0	0	0	1	1	0	0	0	1	0	0	0	0	0	0	0	0	0	0	0	6
3.544	0	0	1	0	0	0	0	0	1	0	0	0	0	1	0	0	0	0	0	1	0	1	1	0	0	6
3.545	0	0	1	0	0	0	0	0	0	1	0	1	0	1	0	0	0	0	0	1	0	0	1	0	0	6
3.546	0	0	0	1	0	0	0	0	0	0	0	1	0	0	0	0	0	0	0	0	0	0	1	1	1	6
3.547	0	0	0	0	0	0	1	0	0	0	0	1	0	1	0	1	0	0	0	0	0	0	0	1	1	6
3.548	0	0	0	0	0	0	0	0	0	1	0	1	1	1	0	0	0	0	0	1	0	1	1	0	0	6
3.549	0	1	0	1	1	0	0	0	0	1	0	1	1	0	0	0	0	0	0	0	0	1	0	0	1	6
3.550	0	1	0	1	0	0	0	0	0	0	0	0	0	1	0	0	0	0	0	0	0	0	1	0	1	6
3.551	0	1	0	0	1	0	1	0	1	1	0	1	1	0	0	0	0	0	0	0	0	0	0	0	0	6
3.552	0	1	0	0	0	0	0	0	0	0	0	0	0	1	0	0	0	0	0	0	0	0	0	0	1	6
3.553	0	0	1	1	0	0	1	0	1	1	0	1	0	1	0	0	0	0	0	1	0	1	0	0	1	6
3.554	0	0	1	0	0	0	1	0	1	0	0	1	1	1	0	0	0	0	0	1	0	1	0	0	0	6
3.555	0	0	0	0	0	0	1	0	1	1	0	0	0	1	0	0	0	0	0	0	0	1	0	1	1	6
3.556	0	0	0	0	0	0	1	0	1	0	0	0	0	1	0	0	0	0	0	0	0	1	0	1	0	6
3.557	0	0	0	0	0	0	0	0	1	1	1	0	0	1	0	0	0	0	0	0	0	1	0	0	0	6
3.558	0	0	0	0	0	0	0	0	0	0	1	0	0	1	0	0	0	0	0	0	0	0	0	1	0	6
3.559	0	0	0	0	0	0	0	0	0	0	0	1	0	1	0	1	0	0	0	1	0	1	0	1	0	6
3.560	0	1	1	0	1	0	0	0	1	0	0	1	0	0	0	0	0	0	0	1	0	1	0	0	0	6
3.561	0	1	1	0	0	0	0	0	0	1	0	1	0	0	0	0	0	0	0	1	0	0	0	0	1	6
3.562	0	1	0	1	0	0	0	0	1	1	0	1	0	0	0	0	0	0	0	1	0	0	0	1	0	6
3.563	0	1	0	0	1	0	0	0	1	1	0	1	1	0	0	0	0	0	0	0	0	1	1	1	1	6
3.564	0	1	0	0	1	0	0	0	1	0	0	0	0	0	0	0	0	0	0	0	0	0	1	0	1	6
3.565	0	1	0	0	1	0	0	0	0	0	0	0	0	0	0	0	0	0	0	1	0	1	1	0	1	6
3.566	0	0	1	0	0	0	1	0	0	1	1	1	0	0	0	0	0	0	0	0	0	0	0	1	1	6
3.567	0	0	1	0	0	0	1	0	0	0	0	0	0	1	0	0	0	0	0	0	0	1	1	0	0	6
3.568	0	0	0	1	1	0	0	0	0	1	0	1	1	0	0	0	0	0	0	0	0	1	0	0	0	6
3.569	0	0	0	1	0	0	0	0	0	1	1	1	0	1	0	0	0	0	0	0	0	1	0	0	0	6
3.570	0	0	0	1	0	0	0	0	0	1	0	1	0	1	0	0	0	0	0	0	0	1	0	0	1	6
3.571	0	0	0	1	0	0	0	0	0	0	0	1	0	1	0	0	0	0	0	0	0	0	0	0	1	6
3.572	0	0	0	0	0	0	1	0	1	1	0	0	0	1	0	0	0	0	0	0	0	0	0	1	0	6
3.573	0	0	0	0	0	0	1	0	1	1	0	0	0	0	0	0	0	0	0	1	0	0	0	1	0	6
3.574	0	0	0	0	0	0	0	0	1	1	0	0	0	1	0	0	0	0	0	0	0	0	0	0	0	6
3.575	0	0	0	0	0	0	0	0	1	0	0	1	0	1	0	1	0	0	0	0	0	0	0	1	1	6
3.576	0	0	0	0	0	0	0	0	0	1	1	1	0	0	0	0	0	0	0	1	0	1	0	0	1	6
3.577	0	1	0	1	0	0	0	0	0	0	0	0	0	0	0	0	0	0	0	1	0	0	0	0	1	6
3.578	0	1	0	0	1	0	0	0	0	0	0	0	1	0	0	0	0	0	0	0	0	0	1	1	1	6
3.579	0	1	0	0	1	0	0	0	0	0	0	0	0	1	0	0	0	0	0	0	0	0	0	0	0	6
3.580	0	1	0	0	1	0	0	0	0	0	0	0	0	0	0	0	0	0	0	1	0	1	1	0	0	6
3.581	0	1	0	0	0	0	0	0	1	0	0	1	0	0	0	1	0	0	0	0	0	0	0	0	0	6
3.582	0	1	0	0	0	0	0	0	0	1	0	1	0	1	0	0	0	0	0	0	0	0	1	0	1	6
3.583	0	0	1	0	0	0	1	0	1	1	1	1	1	1	0	0	0	0	0	1	0	0	0	1	1	6
3.584	0	0	1	0	0	0	1	0	1	1	0	1	0	1	0	1	0	0	0	0	0	0	0	1	1	6
3.585	0	0	1	0	0	0	1	0	0	1	0	1	0	1	0	1	0	0	0	0	0	0	0	1	1	6
3.586	0	0	1	0	0	0	1	0	0	0	0	1	0	0	0	0	0	0	0	1	0	1	0	0	0	6
3.587	0	0	0	0	1	0	0	0	0	0	0	1	0	1	0	0	0	0	0	0	0	0	0	0	1	6
3.588	0	0	0	0	0	0	1	0	0	1	0	1	1	1	0	0	0	0	0	1	0	1	1	0	0	6
3.589	0	0	0	0	0	0	1	0	0	1	0	0	0	1	0	0	0	0	0	0	0	0	1	1	1	6
3.590	0	0	0	0	0	0	0	0	1	0	1	1	0	1	0	0	0	0	0	1	0	0	0	1	1	6
3.591	0	1	1	0	1	0	1	0	0	1	0	1	0	0	0	0	0	0	0	0	0	0	0	0	0	6
3.592	0	1	0	1	0	0	0	0	1	1	0	1	1	0	0	0	0	0	0	0	0	0	1	0	0	6
3.593	0	1	0	1	0	0	0	0	1	0	0	1	1	0	0	0	0	0	0	0	0	0	1	0	0	6
3.594	0	1	0	0	1	0	0	0	1	1	0	1	0	0	0	0	0	0	0	1	0	0	1	1	0	6
3.595	0	1	0	0	1	0	0	0	0	0	0	0	1	0	0	0	0	0	0	0	0	1	0	1	1	6
3.596	0	0	1	1	0	0	1	0	0	1	1	1	0	1	0	1	0	0	0	1	0	0	0	1	1	6
3.597	0	0	1	1	0	0	0	0	0	1	0	1	0	1	0	0	0	0	0	0	0	0	0	0	0	6
3.598	0	0	1	0	0	0	0	0	0	1	0	1	0	1	0	0	0	0	0	1	0	0	1	1	1	6
3.599	0	0	1	0	0	0	0	0	0	1	0	1	0	1	0	0	0	0	0	0	0	0	0	0	1	6
3.600	0	0	0	0	1	0	1	0	0	1	0	1	1	1	0	0	0	0	0	1	0	1	1	0	0	6
3.601	0	0	0	0	0	0	1	0	0	1	0	0	0	1	0	0	0	0	0	1	0	0	1	0	1	6
3.602	0	0	0	0	0	0	0	0	1	1	0	0	0	1	0	0	0	0	0	0	0	0	0	1	1	6
3.603	0	0	0	0	0	0	0	0	1	0	0	1	0	0	0	1	0	0	0	1	0	0	0	1	1	6
3.604	0	0	0	0	0	0	0	0	0	1	0	0	1	1	0	0	0	0	0	0	0	1	0	0	0	6
3.605	0	0	1	0	0	0	1	0	0	1	0	1	1	1	0	1	0	0	0	1	0	0	0	1	1	6
3.606	0	0	1	0	0	0	1	0	0	1	0	1	0	1	0	1	0	0	0	1	0	0	0	1	1	6
3.607	0	0	1	0	0	0	0	0	0	1	0	0	0	0	0	0	0	0	0	1	0	1	0	0	1	6
3.608	0	0	0	1	0	0	0	0	0	0	0	0	1	0	0	0	0	0	0	0	0	1	0	0	0	6
3.609	0	0	0	0	1	0	1	0	0	1	0	1	0	1	0	0	0	0	0	0	0	0	0	1	1	6
3.610	0	0	0	0	1	0	1	0	0	1	0	1	0	1	0	0	0	0	0	0	0	0	0	0	1	6
3.611	0	0	0	0	0	0	1	0	1	0	0	0	0	1	0	0	0	0	0	0	0	0	0	0	1	6
3.612	0	1	0	0	1	0	0	0	1	0	0	0	1	0	0	0	0	0	0	0	0	1	1	0	0	6
3.613	0	1	0	0	0	0	0	0	0	0	1	1	0	0	0	0	0	0	0	0	0	0	1	0	0	6
3.614	0	0	1	0	0	0	1	0	0	0	1	1	0	0	0	0	0	0	0	0	0	0	0	1	1	6
3.615	0	0	1	0	0	0	0	0	1	1	0	1	0	1	0	0	0	0	0	0	0	1	0	1	0	6
3.616	0	0	1	0	0	0	0	0	0	1	1	0	0	1	0	0	0	0	0	1	0	0	0	1	1	6
3.617	0	0	0	1	0	0	1	0	0	1	0	0	0	1	0	0	0	0	0	0	0	1	0	0	0	6
3.618	0	0	0	0	1	0	0	0	0	1	0	1	0	1	0	0	0	0	0	0	0	0	0	0	1	6
3.619	0	0	0	0	1	0	0	0	0	0	0	1	0	1	0	0	0	0	0	0	0	0	1	0	0	6
3.620	0	0	0	0	0	0	0	0	0	0	1	1	0	0	0	0	0	0	0	0	0	0	1	1	0	6
3.621	0	1	0	1	0	0	0	0	0	0	0	1	1	1	0	0	0	0	0	0	0	0	0	0	0	6
3.622	0	0	1	0	0	0	1	0	0	0	1	0	0	0	0	0	0	0	0	0	0	0	0	1	1	6
3.623	0	0	1	0	0	0	1	0	0	0	0	1	0	1	0	0	0	0	0	1	0	0	0	0	0	6
3.624	0	0	0	1	1	0	1	0	0	0	0	1	1	0	0	0	0	0	0	0	0	0	0	0	0	6
3.625	0	0	0	1	0	0	1	0	0	0	0	1	0	0	0	0	0	0	0	0	0	1	0	0	0	6
3.626	0	0	0	0	0	0	1	0	0	0	0	0	0	1	0	0	0	0	0	0	0	1	1	0	1	6
3.627	0	0	0	0	0	0	0	0	0	1	0	1	1	0	0	0	0	0	0	0	0	1	0	0	1	6
3.628	0	1	0	1	1	0	0	0	0	0	0	1	0	0	0	0	0	0	0	0	0	1	0	1	0	6
3.629	0	1	0	1	0	0	0	0	1	0	0	1	1	0	0	0	0	0	0	1	0	1	0	0	1	6
3.630	0	1	0	0	1	0	1	0	0	1	0	1	0	0	0	0	0	0	0	1	0	1	0	0	0	6
3.631	0	0	0	0	1	0	1	0	0	0	0	1	0	0	0	0	0	0	0	0	0	1	0	0	0	6
3.632	0	0	0	0	1	0	1	0	0	0	0	0	1	0	0	0	0	0	0	1	0	0	0	0	0	6
3.633	0	0	0	0	0	0	1	0	0	0	1	1	0	0	0	0	0	0	0	0	0	0	0	1	0	6
3.634	0	0	0	0	0	0	1	0	0	0	1	1	0	0	0	0	0	0	0	0	0	0	0	0	1	6
3.635	0	0	0	0	0	0	1	0	0	1	0	1	1	0	0	0	0	0	0	1	0	1	0	0	0	6
3.636	0	0	0	0	0	0	0	0	1	0	0	0	1	0	0	0	0	0	0	1	0	0	1	0	0	6
3.637	0	0	0	0	0	0	0	0	1	0	0	0	0	0	0	0	0	0	0	0	0	1	1	1	1	6
3.638	0	1	0	1	0	0	0	0	0	0	0	0	1	0	0	0	0	0	0	0	0	0	0	0	1	6
3.639	0	0	1	0	0	0	1	0	0	0	0	0	0	0	0	0	0	0	0	0	0	0	0	0	1	6
3.640	0	0	0	0	0	0	0	0	1	0	0	0	0	0	0	0	0	0	0	0	0	0	1	0	1	6
3.641	0	0	0	0	1	0	0	0	1	1	0	0	1	0	0	1	0	0	0	1	0	0	0	0	0	6
3.642	0	0	0	0	0	0	1	0	1	0	0	0	0	0	0	0	0	0	0	1	0	1	0	1	0	6
3.643	0	1	0	1	1	0	0	0	1	1	0	1	0	0	0	0	0	0	0	0	0	1	0	1	0	5
3.644	0	1	0	1	0	0	0	0	0	0	0	0	1	0	0	0	0	0	0	1	0	0	0	1	1	5
3.645	0	1	0	0	0	0	0	0	1	0	0	1	0	0	0	0	0	0	0	1	0	1	0	1	0	5
3.646	0	0	0	1	1	0	0	0	1	0	0	1	0	0	0	0	0	0	0	0	0	1	0	1	0	5
3.647	0	0	0	1	1	0	0	0	0	0	0	1	1	0	0	0	0	0	0	1	0	1	0	0	0	5
3.648	0	0	0	1	1	0	0	0	0	0	0	1	1	0	0	0	0	0	0	1	0	0	0	0	1	5
3.649	0	1	1	1	1	0	1	0	1	0	0	1	1	0	0	0	0	0	0	1	0	0	0	1	1	5
3.650	0	1	1	0	0	0	1	0	1	1	0	1	1	0	0	0	0	0	0	1	0	1	0	1	1	5
3.651	0	1	0	1	1	0	0	0	1	1	0	1	0	1	0	0	0	0	0	0	0	0	1	0	0	5
3.652	0	1	0	1	0	0	0	0	1	0	0	1	0	0	0	0	0	0	0	0	0	1	0	1	0	5
3.653	0	1	0	0	1	0	0	0	1	0	0	0	0	0	0	0	0	0	0	1	0	1	0	1	1	5
3.654	0	1	0	0	0	0	1	0	1	1	0	1	0	0	0	0	0	0	0	1	0	0	0	1	1	5
3.655	0	1	0	0	0	0	0	0	1	0	0	1	1	0	0	0	0	0	0	1	0	1	1	0	1	5
3.656	0	0	1	1	1	0	0	0	1	1	0	1	0	0	0	0	0	0	0	1	0	0	0	0	0	5
3.657	0	0	1	0	0	0	1	0	1	0	0	1	0	0	0	0	0	0	0	1	0	1	0	1	1	5
3.658	0	0	1	0	0	0	1	0	0	0	0	0	0	0	0	1	0	0	0	0	0	0	0	1	1	5
3.659	0	0	1	0	0	0	0	0	1	1	0	0	0	0	0	0	0	0	0	0	0	1	0	1	1	5

CMP	CD2	CD3	CD4	CD7	CD8	CD10	CD13	CD18	CD26	CD29	CD36	CD44	CD45RA	CD49f	CD54	CD56	CD57	CD58	CD62L	CD71	CD80	Cytokeratin	Histone_H1	HLA-DQ	HLA-DR	Freq. (voxel)
**3.660**	0	0	0	1	1	0	1	0	1	0	0	1	1	0	0	0	0	0	0	0	0	0	0	0	0	5
**3.661**	0	0	0	1	1	0	1	0	1	0	0	1	0	0	0	0	0	0	0	0	0	0	1	1	1	5
**3.662**	0	0	0	1	1	0	1	0	1	0	0	1	0	0	0	0	0	0	0	0	0	0	1	0	0	5
**3.663**	0	0	0	1	1	0	0	0	1	0	0	1	0	0	0	1	0	0	0	1	0	0	0	1	1	5
**3.664**	0	0	0	1	0	0	1	0	1	1	0	1	1	0	0	0	0	0	0	0	0	0	0	0	0	5
**3.665**	0	0	0	1	0	0	1	0	1	0	0	0	0	0	0	0	0	0	0	0	0	0	1	0	0	5
**3.666**	0	0	0	1	0	0	1	0	0	0	0	1	0	0	0	0	0	0	0	1	0	0	0	0	0	5
**3.667**	0	0	0	1	0	0	0	0	1	1	0	1	1	1	0	0	0	0	0	1	0	0	0	1	0	5
**3.668**	0	0	0	1	0	0	0	0	1	0	0	1	1	0	0	0	0	0	0	0	0	0	0	1	1	5
**3.669**	0	0	0	1	0	0	0	0	0	1	0	1	1	0	0	1	0	0	0	0	0	0	0	1	0	5
**3.670**	0	0	0	0	1	0	0	0	1	1	0	1	1	0	0	0	0	0	0	0	0	0	0	1	0	5
**3.671**	0	0	0	0	1	0	0	0	1	0	0	1	1	0	0	0	0	0	0	0	0	0	1	0	0	5
**3.672**	0	1	1	0	1	0	0	0	0	0	0	1	1	0	0	0	0	0	0	0	0	0	0	1	1	5
**3.673**	0	1	0	1	1	0	0	0	1	1	0	1	1	1	0	0	0	0	0	1	0	1	0	0	0	5
**3.674**	0	1	0	1	1	0	0	0	1	1	0	1	1	0	0	0	0	0	0	1	0	0	1	1	0	5
**3.675**	0	1	0	1	1	0	0	0	1	1	0	1	0	0	0	0	0	0	0	0	0	0	1	1	0	5
**3.676**	0	1	0	1	1	0	0	0	1	0	0	1	1	0	0	0	0	0	0	0	0	0	1	1	0	5
**3.677**	0	1	0	1	1	0	0	0	1	0	0	1	0	0	0	0	0	0	0	1	0	1	1	1	1	5
**3.678**	0	1	0	1	0	0	0	0	0	0	0	1	1	1	0	0	0	0	0	0	0	0	1	0	0	5
**3.679**	0	1	0	0	1	0	0	0	1	0	0	1	1	0	0	0	0	0	0	0	0	1	1	0	0	5
**3.680**	0	1	0	0	0	0	0	0	1	0	0	1	1	1	0	0	0	0	0	1	0	0	0	1	1	5
**3.681**	0	0	1	1	1	0	0	0	1	1	0	1	1	0	0	0	0	0	0	1	0	0	0	0	0	5
**3.682**	0	0	1	0	1	0	0	0	0	0	0	1	0	0	0	0	0	0	0	1	0	0	0	0	0	5
**3.683**	0	0	1	0	0	0	1	0	1	1	0	0	0	0	0	0	0	0	0	1	0	0	0	1	1	5
**3.684**	0	0	0	1	0	0	0	0	1	0	0	0	0	1	0	0	0	0	0	1	0	1	0	1	0	5
**3.685**	0	0	0	1	0	0	0	0	0	1	0	0	1	0	0	0	0	0	0	1	0	0	0	1	0	5
**3.686**	0	0	0	0	1	0	0	0	0	1	0	1	1	0	0	0	0	0	0	1	0	0	0	0	1	5
**3.687**	0	0	0	0	0	0	0	0	1	1	0	1	1	0	0	0	0	0	0	1	0	0	1	0	0	5
**3.688**	0	0	0	0	0	0	0	0	0	0	0	1	1	0	0	1	0	0	0	0	0	0	0	1	0	5
**3.689**	0	1	1	1	1	0	0	0	1	1	0	1	1	0	0	0	0	0	0	1	0	0	1	0	0	5
**3.690**	0	1	1	0	0	0	0	0	1	0	0	1	1	0	0	0	0	0	0	1	0	0	1	0	0	5
**3.691**	0	1	1	0	0	0	0	0	1	0	0	1	0	0	0	0	0	0	0	1	0	1	1	1	1	5
**3.692**	0	1	0	0	1	0	0	0	1	1	0	1	1	1	0	0	0	0	0	1	0	0	1	0	0	5
**3.693**	0	1	0	0	1	0	0	0	1	1	0	1	1	1	0	0	0	0	0	0	0	0	1	0	0	5
**3.694**	0	1	0	0	1	0	0	0	1	0	0	1	1	0	0	0	0	0	0	1	0	0	1	0	0	5
**3.695**	0	1	0	0	1	0	0	0	0	0	0	1	0	1	0	0	0	0	0	1	0	1	0	0	1	5
**3.696**	0	0	1	1	0	0	0	0	1	1	0	1	0	0	0	0	0	0	0	1	0	1	0	0	0	5
**3.697**	0	0	1	1	0	0	0	0	0	0	0	1	1	0	0	0	0	0	0	1	0	1	0	0	0	5
**3.698**	0	0	1	1	0	0	0	0	0	0	0	0	1	0	0	0	0	0	0	1	0	1	0	1	1	5
**3.699**	0	0	1	0	0	0	0	0	1	1	0	1	0	0	0	0	0	0	0	1	0	0	0	0	1	5
**3.700**	0	0	1	0	0	0	0	0	0	0	0	1	0	0	0	0	0	0	0	1	0	0	1	1	1	5
**3.701**	0	0	1	0	0	0	0	0	0	0	0	0	1	1	0	0	0	0	0	1	0	0	0	0	1	5
**3.702**	0	0	0	1	1	0	0	0	1	1	0	1	0	1	0	0	0	0	0	1	0	1	0	1	1	5
**3.703**	0	0	0	1	0	0	1	0	1	0	0	1	0	0	0	0	0	0	0	1	0	0	0	1	1	5
**3.704**	0	0	0	1	0	0	0	0	0	0	0	1	0	1	0	0	0	0	0	1	0	1	0	1	0	5
**3.705**	0	0	0	1	0	0	0	0	0	0	0	1	0	0	0	0	0	0	0	1	0	0	1	1	1	5
**3.706**	0	0	0	1	0	0	0	0	0	0	0	0	0	1	0	0	0	0	0	1	0	1	0	1	0	5
**3.707**	0	0	0	0	1	0	1	0	1	0	0	1	0	0	0	1	0	0	0	1	0	0	0	1	1	5
**3.708**	0	0	0	0	1	0	0	0	0	1	0	1	1	0	0	0	0	0	0	1	0	1	1	0	0	5
**3.709**	0	0	0	0	1	0	0	0	0	1	0	1	1	0	0	0	0	0	0	0	0	1	0	0	1	5
**3.710**	0	0	0	0	1	0	0	0	0	0	0	1	1	0	0	0	0	0	0	1	0	1	0	1	1	5
**3.711**	0	0	0	0	0	0	1	0	0	0	0	1	0	1	0	0	0	0	0	1	0	0	0	0	1	5
**3.712**	0	0	0	0	0	0	1	0	0	0	0	1	0	0	0	1	0	0	0	0	0	0	1	1	0	5
**3.713**	0	0	0	0	0	0	0	0	1	0	0	0	0	0	0	0	0	0	0	1	0	1	1	1	0	5
**3.714**	0	0	0	0	0	0	0	0	0	0	0	0	1	0	0	0	0	0	0	1	0	1	0	1	1	5
**3.715**	0	1	1	0	1	0	0	0	0	0	0	0	1	0	0	0	0	0	0	1	0	0	0	1	1	5
**3.716**	0	1	1	0	0	0	0	0	1	1	0	1	0	0	0	1	0	0	0	1	0	0	0	1	1	5
**3.717**	0	1	1	0	0	0	0	0	0	1	0	1	0	0	0	0	0	0	0	1	0	0	1	0	0	5
**3.718**	0	1	1	0	0	0	0	0	0	0	0	1	0	0	0	0	0	0	0	0	0	1	0	1	1	5
**3.719**	0	1	0	1	1	0	0	0	1	1	0	1	0	1	0	0	0	0	0	0	0	1	0	1	1	5
**3.720**	0	1	0	1	1	0	0	0	1	1	0	0	0	0	0	0	0	0	0	1	0	0	0	0	0	5
**3.721**	0	1	0	1	1	0	0	0	0	1	0	1	0	1	0	0	0	0	0	0	0	1	0	0	1	5
**3.722**	0	1	0	1	1	0	0	0	0	0	0	0	1	0	0	0	0	0	0	1	0	0	0	0	1	5
**3.723**	0	1	0	1	1	0	0	0	0	0	0	0	0	1	0	0	0	0	0	1	0	0	0	0	0	5
**3.724**	0	1	0	1	0	0	0	0	0	0	0	1	1	0	0	0	0	0	0	1	0	1	0	1	1	5
**3.725**	0	1	0	1	0	0	0	0	0	0	0	1	0	1	0	0	0	0	0	1	0	1	0	1	1	5
**3.726**	0	1	0	1	0	0	0	0	0	0	0	1	0	1	0	0	0	0	0	1	0	0	0	1	1	5
**3.727**	0	1	0	1	0	0	0	0	0	0	0	1	0	0	0	0	0	0	0	0	0	1	1	1	1	5
**3.728**	0	1	0	0	1	0	0	0	0	1	0	1	1	0	0	0	0	0	0	1	0	1	0	1	0	5
**3.729**	0	1	0	0	1	0	0	0	0	1	0	1	1	0	0	0	0	0	0	0	0	1	1	1	1	5
**3.730**	0	1	0	0	1	0	0	0	0	0	0	1	1	0	0	0	0	0	0	1	0	1	0	1	0	5
**3.731**	0	1	0	0	0	0	0	0	1	0	0	1	1	0	0	0	0	0	0	0	0	1	0	1	1	5
**3.732**	0	1	0	0	0	0	0	0	0	0	0	1	1	1	0	0	0	0	0	0	0	1	1	0	0	5
**3.733**	0	0	1	0	0	0	1	0	0	0	0	0	1	1	0	1	0	0	0	1	0	0	0	0	0	5
**3.734**	0	0	1	0	0	0	0	0	1	0	0	1	0	0	0	0	0	0	0	1	0	1	1	0	0	5
**3.735**	0	0	1	0	0	0	0	0	0	1	0	1	0	0	0	0	0	0	0	0	0	0	1	0	0	5
**3.736**	0	0	0	1	0	0	0	0	0	1	0	1	0	0	0	0	0	0	0	1	0	1	0	1	0	5
**3.737**	0	0	0	1	0	0	0	0	0	1	0	0	0	1	0	0	0	0	0	1	0	0	0	0	0	5
**3.738**	0	0	0	1	0	0	0	0	0	0	0	1	0	1	0	0	0	0	0	1	0	1	0	1	1	5
**3.739**	0	0	0	0	1	0	0	0	0	1	0	1	1	0	0	0	0	0	0	0	0	1	1	0	0	5
**3.740**	0	0	0	0	1	0	0	0	0	0	0	0	1	0	0	0	0	0	0	1	0	0	0	0	1	5
**3.741**	0	0	0	0	0	0	0	0	1	0	0	1	1	1	0	0	0	0	0	1	0	1	0	1	1	5
**3.742**	0	0	0	0	0	0	0	0	1	0	0	1	0	1	0	0	0	0	0	1	0	0	1	0	0	5
**3.743**	0	1	1	1	1	0	0	0	0	0	0	1	1	0	0	0	0	0	0	1	0	0	0	1	0	5
**3.744**	0	1	1	0	1	0	0	0	0	0	0	1	0	0	0	0	0	0	0	0	0	1	0	0	0	5
**3.745**	0	1	1	0	0	0	0	0	1	1	0	1	1	1	0	0	0	0	0	1	0	0	0	0	0	5
**3.746**	0	1	1	0	0	0	0	0	1	0	0	0	0	0	0	0	0	0	0	1	0	0	0	1	1	5
**3.747**	0	1	0	1	1	0	0	0	0	1	0	1	1	1	0	0	0	0	0	1	0	0	0	1	0	5
**3.748**	0	1	0	1	1	0	0	0	0	1	0	0	1	0	0	0	0	0	0	1	0	0	1	0	0	5
**3.749**	0	1	0	1	1	0	0	0	0	0	0	1	0	1	0	0	0	0	0	1	0	0	0	0	1	5
**3.750**	0	1	0	1	1	0	0	0	0	0	0	1	0	0	0	0	0	0	0	1	0	1	1	1	1	5
**3.751**	0	1	0	0	1	0	0	0	0	1	0	1	0	0	0	0	0	0	0	1	0	1	0	0	1	5
**3.752**	0	1	0	0	0	0	0	0	0	1	0	1	0	1	0	0	0	0	0	1	0	0	1	1	0	5
**3.753**	0	0	1	1	1	0	0	0	0	1	0	1	0	0	0	0	0	0	0	1	0	0	0	0	0	5
**3.754**	0	0	1	0	1	0	0	0	0	1	0	1	0	0	0	0	0	0	0	1	0	0	0	1	0	5
**3.755**	0	0	1	0	0	0	0	0	1	1	0	1	0	0	0	0	0	0	0	0	0	0	0	0	1	5
**3.756**	0	0	0	1	0	0	1	0	0	1	0	1	0	1	0	1	0	0	0	1	0	0	0	1	1	5
**3.757**	0	0	0	1	0	0	0	0	0	1	0	1	0	0	0	0	0	0	0	1	0	1	0	0	1	5
**3.758**	0	0	0	0	1	0	0	0	0	1	0	1	0	0	0	0	0	0	0	1	0	0	1	1	0	5
**3.759**	0	0	0	0	0	0	0	0	1	0	0	1	1	0	0	0	0	0	0	0	0	1	1	0	0	5
**3.760**	0	0	0	0	0	0	0	0	0	1	0	1	1	0	0	0	0	0	0	0	0	1	0	1	0	5
**3.761**	0	1	1	1	0	0	0	0	0	1	0	1	0	0	0	0	0	0	0	0	0	0	0	1	1	5
**3.762**	0	1	1	0	1	0	0	0	1	0	0	1	1	0	0	0	0	0	0	1	0	0	0	0	1	5
**3.763**	0	1	1	0	1	0	0	0	0	1	0	1	0	0	0	0	0	0	0	0	0	0	1	0	0	5
**3.764**	0	1	1	0	0	0	0	0	1	1	0	1	1	1	0	0	0	0	0	0	0	0	0	0	0	5
**3.765**	0	1	0	1	0	0	1	0	0	1	0	1	0	0	0	0	0	0	0	1	0	0	1	0	0	5
**3.766**	0	1	0	1	0	0	0	0	1	1	0	1	1	0	0	0	0	0	0	0	0	1	0	0	0	5
**3.767**	0	1	0	0	0	0	0	0	1	1	0	1	0	1	0	0	0	0	0	1	0	0	0	1	0	5
**3.768**	0	1	0	0	0	0	0	0	0	1	0	1	1	0	0	0	0	0	0	0	0	1	1	0	0	5
**3.769**	0	1	0	0	0	0	0	0	0	1	0	1	0	0	0	0	0	0	0	1	0	0	1	1	0	5
**3.770**	0	1	0	0	0	0	0	0	0	1	0	1	0	0	0	0	0	0	0	0	0	1	1	0	1	5
**3.771**	0	0	1	1	0	0	0	0	0	1	0	1	0	0	0	0	0	0	0	0	0	0	0	0	0	5
**3.772**	0	0	1	1	0	0	0	0	0	0	0	0	0	0	0	0	0	0	0	0	0	1	0	0	0	5
**3.773**	0	0	1	0	1	0	0	0	0	0	0	1	0	0	0	0	0	0	0	0	0	0	1	0	0	5
**3.774**	0	0	1	0	0	0	1	0	0	1	0	1	1	1	0	0	0	0	0	1	0	1	0	1	0	5
**3.775**	0	0	1	0	0	0	1	0	0	1	0	0	0	1	0	0	0	0	0	0	0	0	1	1	0	5
**3.776**	0	0	1	0	0	0	1	0	0	0	0	1	0	1	0	0	0	0	0	1	0	1	0	1	1	5
**3.777**	0	0	1	0	0	0	1	0	0	0	0	0	0	0	0	0	0	0	0	1	0	1	1	0	0	5
**3.778**	0	0	1	0	0	0	0	0	0	1	0	0	0	0	0	0	0	0	0	0	0	1	1	0	0	5
**3.779**	0	0	1	0	0	0	0	0	0	0	1	1	0	0	0	0	0	0	0	0	0	0	0	0	0	5
**3.780**	0	0	1	0	0	0	0	0	0	0	0	0	1	0	0	1	0	0	0	1	0	1	0	0	0	5
**3.781**	0	0	0	0	1	0	1	0	0	1	0	1	0	1	0	0	0	0	0	1	0	1	1	1	1	5

CMP	CD2	CD3	CD4	CD7	CD8	CD10	CD13	CD18	CD26	CD29	CD36	CD44	CD45RA	CD49f	CD54	CD56	CD57	CD58	CD62L	CD71	CD80	Cytokeratin	Histone_H1	HLA-DQ	HLA-DR	Freq. (voxel)
**3.782**	0	0	0	0	1	0	1	0	0	1	0	1	0	1	0	0	0	0	0	1	0	1	0	1	0	5
**3.783**	0	0	0	0	0	0	1	0	0	0	1	0	1	0	0	1	0	0	0	0	0	0	0	0	0	5
**3.784**	0	0	0	0	0	0	1	0	0	0	1	0	1	0	0	0	0	0	0	0	0	0	0	0	0	5
**3.785**	0	0	0	0	0	0	0	0	0	1	0	1	1	1	0	0	0	0	0	1	0	0	0	0	0	5
**3.786**	0	0	0	0	0	0	0	0	0	1	0	1	0	0	0	1	0	0	0	0	0	1	1	0	0	5
**3.787**	0	1	1	0	0	0	0	0	0	1	0	1	0	0	0	0	0	0	0	0	0	0	1	0	0	5
**3.788**	0	1	0	1	0	0	0	0	0	1	0	1	0	1	0	0	0	0	0	1	0	0	0	1	1	5
**3.789**	0	1	0	0	1	0	0	0	1	0	0	0	1	0	0	0	0	0	0	0	0	0	0	1	1	5
**3.790**	0	1	0	0	1	0	0	0	0	1	0	1	1	1	0	0	0	0	0	1	0	0	0	1	1	5
**3.791**	0	1	0	0	1	0	0	0	0	1	0	1	0	1	0	0	0	0	0	1	0	0	0	0	1	5
**3.792**	0	1	0	0	0	0	0	0	1	0	0	1	1	0	0	0	0	0	0	0	0	0	0	0	1	5
**3.793**	0	1	0	0	0	0	0	0	0	0	0	0	0	1	0	0	0	0	0	0	0	0	1	1	1	5
**3.794**	0	0	1	1	0	0	1	0	0	1	0	1	1	1	0	0	0	0	0	1	0	1	0	1	1	5
**3.795**	0	0	1	1	0	0	0	0	1	0	0	1	1	1	0	0	0	0	0	1	0	1	0	1	1	5
**3.796**	0	0	1	1	0	0	0	0	0	0	0	0	1	0	0	0	0	0	0	0	0	0	0	1	0	5
**3.797**	0	0	1	0	1	0	0	0	0	0	0	1	0	0	0	0	0	0	0	0	0	1	0	0	0	5
**3.798**	0	0	1	0	0	0	1	0	0	0	1	1	0	1	0	0	0	0	0	1	0	1	0	0	0	5
**3.799**	0	0	1	0	0	0	1	0	0	0	0	1	0	1	0	0	0	0	0	0	0	0	0	1	0	5
**3.800**	0	0	1	0	0	0	0	0	1	1	0	1	0	1	0	0	0	0	0	0	0	0	0	1	0	5
**3.801**	0	0	1	0	0	0	0	0	1	0	0	1	1	1	0	0	0	0	0	1	0	1	0	1	1	5
**3.802**	0	0	1	0	0	0	0	0	0	1	0	0	1	1	0	0	0	0	0	0	0	0	0	1	0	5
**3.803**	0	0	1	0	0	0	0	0	0	1	0	0	1	0	0	0	0	0	0	0	0	0	0	0	0	5
**3.804**	0	0	0	0	1	0	0	0	1	0	0	0	0	0	0	0	0	0	0	0	0	0	0	0	1	5
**3.805**	0	0	0	0	0	0	0	0	1	0	0	1	1	0	0	0	0	0	0	0	0	0	1	0	0	5
**3.806**	0	1	1	0	0	0	0	0	0	1	0	1	0	0	0	0	0	0	0	0	0	0	0	0	1	5
**3.807**	0	1	1	0	0	0	0	0	0	0	0	1	0	1	0	0	0	0	0	0	0	0	1	0	0	5
**3.808**	0	1	0	1	1	0	0	0	0	1	0	1	0	1	0	0	0	0	0	0	0	0	0	1	1	5
**3.809**	0	1	0	0	1	0	0	0	0	0	0	1	0	1	0	0	0	0	0	0	0	1	0	1	0	5
**3.810**	0	1	0	0	1	0	0	0	0	0	0	0	1	0	0	0	0	0	0	0	0	1	1	1	1	5
**3.811**	0	1	0	0	0	0	1	0	0	1	0	1	0	1	0	0	0	0	0	0	0	0	1	0	0	5
**3.812**	0	1	0	0	0	0	0	0	1	0	0	0	0	0	0	0	0	0	0	1	0	0	1	1	0	5
**3.813**	0	1	0	0	0	0	0	0	0	0	0	1	0	1	0	0	0	0	0	0	0	1	1	0	1	5
**3.814**	0	1	0	0	0	0	0	0	0	0	0	0	1	0	0	0	0	0	0	0	0	1	1	1	0	5
**3.815**	0	0	1	0	0	0	1	0	1	0	0	1	1	1	0	0	0	0	0	1	0	1	0	0	1	5
**3.816**	0	0	1	0	0	0	1	0	1	0	0	1	0	1	0	0	0	0	0	0	0	1	0	0	1	5
**3.817**	0	0	1	0	0	0	0	0	1	0	1	0	0	0	0	0	0	0	0	0	0	1	0	1	0	5
**3.818**	0	0	0	1	1	0	0	0	1	0	0	0	0	0	0	0	0	0	0	1	0	1	0	0	0	5
**3.819**	0	0	0	1	0	0	0	0	0	1	0	1	0	1	0	0	0	0	0	0	0	0	0	1	1	5
**3.820**	0	0	0	0	0	0	1	0	1	0	1	1	0	1	0	0	0	0	0	0	0	1	0	0	0	5
**3.821**	0	0	0	0	0	0	1	0	0	1	0	0	1	0	0	1	0	0	0	0	0	0	0	0	0	5
**3.822**	0	0	0	0	0	0	0	0	0	1	0	1	1	1	0	0	0	0	0	0	0	0	0	1	1	5
**3.823**	0	0	0	0	0	0	0	0	0	0	1	0	0	1	0	0	0	0	0	0	0	1	0	1	0	5
**3.824**	0	1	0	1	1	0	0	0	0	0	0	1	0	0	0	0	0	0	0	1	0	1	1	1	0	5
**3.825**	0	1	0	0	1	0	0	0	0	0	0	0	0	1	0	0	0	0	0	1	0	0	1	0	0	5
**3.826**	0	1	0	0	0	0	0	0	0	0	0	0	0	0	0	0	0	0	0	1	0	0	1	1	0	5
**3.827**	0	0	1	1	0	0	1	0	1	1	0	1	1	1	0	1	0	0	0	1	0	0	1	1	1	5
**3.828**	0	0	1	0	0	0	1	0	1	0	0	0	1	0	0	0	0	0	0	0	0	0	0	1	0	5
**3.829**	0	0	1	0	0	0	1	0	0	1	0	1	0	1	0	1	0	0	0	1	0	1	0	1	1	5
**3.830**	0	0	0	1	0	0	1	0	0	1	0	1	0	1	0	0	0	0	0	1	0	1	1	0	1	5
**3.831**	0	0	0	1	0	0	0	0	0	1	0	0	0	1	0	0	0	0	0	0	0	0	0	1	0	5
**3.832**	0	0	0	0	0	0	1	0	1	1	0	1	0	1	0	0	0	0	0	0	0	0	1	0	1	5
**3.833**	0	0	0	0	0	0	0	0	1	0	0	0	0	1	0	0	0	0	0	0	0	0	0	1	0	5
**3.834**	0	1	1	1	1	0	0	0	1	0	0	1	0	0	0	0	0	0	0	1	0	0	0	1	0	5
**3.835**	0	1	1	0	1	0	0	0	0	0	0	1	0	0	0	0	0	0	0	0	0	0	0	1	0	5
**3.836**	0	1	0	0	1	0	0	0	1	1	0	1	1	0	0	0	0	0	0	0	0	1	0	0	1	5
**3.837**	0	1	0	0	1	0	0	0	0	1	1	1	0	0	0	0	0	0	0	0	0	0	1	0	1	5
**3.838**	0	1	0	0	0	0	0	0	1	1	0	1	0	0	0	0	0	0	0	0	0	0	1	1	1	5
**3.839**	0	0	1	1	0	0	1	0	0	1	1	1	1	1	0	0	0	0	0	0	0	0	0	1	1	5
**3.840**	0	0	1	1	0	0	1	0	0	1	0	0	0	1	0	0	0	0	0	0	0	1	0	0	0	5
**3.841**	0	0	1	1	0	0	0	0	0	0	0	0	0	0	0	0	0	0	0	0	0	1	0	0	1	5
**3.842**	0	0	1	0	0	0	1	0	1	1	0	0	0	0	0	0	0	0	0	1	0	0	0	1	0	5
**3.843**	0	0	1	0	0	0	1	0	0	0	0	1	0	0	0	0	0	0	0	0	0	0	0	0	1	5
**3.844**	0	0	1	0	0	0	0	0	1	0	1	0	0	0	0	0	0	0	0	0	0	0	0	0	0	5
**3.845**	0	0	0	1	0	0	1	0	0	1	0	1	0	1	0	0	0	0	0	1	0	0	0	1	0	5
**3.846**	0	0	0	1	0	0	1	0	0	1	0	1	0	1	0	0	0	0	0	0	0	1	1	0	0	5
**3.847**	0	0	0	0	0	0	1	0	1	1	0	1	0	0	0	0	0	0	0	0	0	1	0	0	0	5
**3.848**	0	0	0	0	0	0	1	0	1	0	0	1	0	0	0	0	0	0	0	0	0	1	0	0	1	5
**3.849**	0	0	0	0	0	0	1	0	0	1	1	1	0	1	0	1	0	0	0	0	0	0	0	1	1	5
**3.850**	0	0	0	0	0	0	0	0	1	1	0	0	0	1	0	0	0	0	0	1	0	1	1	0	0	5
**3.851**	0	0	0	0	0	0	0	0	1	0	1	0	0	0	0	0	0	0	0	0	0	0	1	0	0	5
**3.852**	0	0	0	0	0	0	0	0	0	1	1	1	0	0	0	1	0	0	0	0	0	0	0	1	1	5
**3.853**	0	0	0	0	0	0	0	0	0	1	0	0	1	1	0	0	0	0	0	1	0	1	1	0	0	5
**3.854**	0	0	0	0	0	0	0	0	0	0	0	0	0	0	0	1	0	0	0	0	0	1	0	0	1	5
**3.855**	0	1	0	1	0	0	0	0	0	0	0	0	0	0	0	0	0	0	0	0	0	0	0	1	0	5
**3.856**	0	1	0	0	1	0	1	0	1	1	0	1	0	0	0	0	0	0	0	0	0	0	0	0	0	5
**3.857**	0	1	0	0	1	0	0	0	0	1	1	1	0	1	0	0	0	0	0	0	0	1	0	0	0	5
**3.858**	0	1	0	0	1	0	0	0	0	1	1	1	0	0	0	0	0	0	0	0	0	0	1	1	1	5
**3.859**	0	1	0	0	1	0	0	0	0	1	0	1	1	1	0	0	0	0	0	0	0	0	0	0	1	5
**3.860**	0	1	0	0	0	0	0	0	0	0	0	1	0	0	0	1	0	0	0	0	0	0	0	1	0	5
**3.861**	0	0	1	1	0	0	1	0	0	1	1	1	1	1	0	0	0	0	0	1	0	0	0	1	1	5
**3.862**	0	0	1	0	0	0	1	0	1	1	0	1	1	1	0	0	0	0	0	0	0	0	0	1	1	5
**3.863**	0	0	1	0	0	0	1	0	0	1	0	1	0	1	0	0	0	0	0	1	0	0	0	0	0	5
**3.864**	0	0	1	0	0	0	1	0	0	1	0	1	0	0	0	0	0	0	0	1	0	1	0	0	0	5
**3.865**	0	0	0	1	1	0	0	0	1	0	0	1	0	0	0	0	0	0	0	0	0	1	0	0	1	5
**3.866**	0	0	0	0	1	0	1	0	1	1	0	1	0	0	0	0	0	0	0	1	0	0	0	0	0	5
**3.867**	0	0	0	0	1	0	1	0	0	1	0	1	1	1	0	0	0	0	0	1	0	1	1	0	1	5
**3.868**	0	0	0	0	0	0	0	0	1	1	0	1	0	1	0	0	0	0	0	0	0	0	0	0	1	5
**3.869**	0	0	0	0	0	0	0	0	0	1	0	1	0	1	0	1	0	0	0	1	0	1	0	0	0	5
**3.870**	0	0	0	0	0	0	0	0	0	0	1	0	0	0	0	1	0	0	0	0	0	1	0	0	1	5
**3.871**	0	0	0	0	0	0	0	0	0	0	1	0	0	0	0	1	0	0	0	0	0	0	0	1	0	5
**3.872**	0	0	0	0	0	0	0	0	0	0	0	1	1	0	0	0	0	0	0	1	0	0	0	0	1	5
**3.873**	0	1	0	1	0	0	0	0	0	0	0	0	0	0	0	0	0	0	0	0	0	1	0	0	1	5
**3.874**	0	1	0	0	1	0	0	0	0	1	1	1	0	1	0	0	0	0	0	0	0	0	1	0	0	5
**3.875**	0	1	0	0	1	0	0	0	0	1	1	1	0	0	0	0	0	0	0	0	0	0	0	0	0	5
**3.876**	0	1	0	0	1	0	0	0	0	0	0	1	0	1	0	0	0	0	0	0	0	0	1	0	1	5
**3.877**	0	1	0	0	0	0	1	0	0	1	0	1	0	1	0	0	0	0	0	0	0	0	0	0	0	5
**3.878**	0	1	0	0	0	0	0	0	1	0	0	0	1	0	0	0	0	0	0	0	0	0	1	0	1	5
**3.879**	0	1	0	0	0	0	0	0	0	0	0	0	1	0	0	0	0	0	0	0	0	0	0	1	1	5
**3.880**	0	0	1	1	0	0	1	0	1	1	0	0	0	1	0	0	0	0	0	1	0	0	0	1	1	5
**3.881**	0	0	1	0	0	0	1	0	0	1	1	1	1	1	0	1	0	0	0	1	0	0	0	1	1	5
**3.882**	0	0	1	0	0	0	1	0	0	1	0	0	0	1	0	0	0	0	0	0	0	1	0	0	1	5
**3.883**	0	0	0	0	0	0	1	0	0	1	0	1	1	1	0	0	0	0	0	1	0	1	1	0	1	5
**3.884**	0	0	0	0	0	0	1	0	0	1	0	1	1	1	0	0	0	0	0	1	0	1	0	1	1	5
**3.885**	0	0	0	0	0	0	1	0	0	0	0	1	1	1	0	0	0	0	0	0	0	0	0	0	0	5
**3.886**	0	0	0	0	0	0	0	0	1	1	0	0	0	0	0	0	0	0	0	1	0	0	1	1	0	5
**3.887**	0	0	0	0	0	0	0	0	0	1	1	1	0	1	0	0	0	0	0	1	0	0	0	0	0	5
**3.888**	0	0	0	0	0	0	0	0	0	0	1	0	0	1	0	0	0	0	0	0	0	1	1	0	0	5
**3.889**	0	1	1	0	0	0	0	0	0	1	0	0	0	0	0	0	0	0	0	0	0	0	0	0	0	5
**3.890**	0	1	0	0	1	0	0	0	1	1	0	0	0	0	0	0	0	0	0	1	0	0	1	0	0	5
**3.891**	0	1	0	0	0	0	1	0	0	0	1	1	0	1	0	0	0	0	0	0	0	0	0	0	0	5
**3.892**	0	1	0	0	0	0	1	0	0	0	0	1	0	1	0	0	0	0	0	0	0	0	0	0	0	5
**3.893**	0	0	1	0	0	0	0	0	1	0	0	1	0	1	0	0	0	0	0	0	0	0	0	1	1	5
**3.894**	0	0	0	0	1	0	0	0	0	0	0	0	0	0	0	0	0	0	0	1	0	0	1	0	1	5
**3.895**	0	0	0	0	0	0	0	0	1	1	1	0	0	1	0	0	0	0	0	0	0	0	0	1	1	5
**3.896**	0	0	0	0	0	0	0	0	0	0	0	0	1	0	0	1	0	0	0	1	0	0	1	1	0	5
**3.897**	0	1	0	1	0	0	0	0	0	0	0	1	1	1	0	0	0	0	0	1	0	0	0	0	0	5
**3.898**	0	1	0	0	1	0	0	0	1	0	0	1	0	0	0	0	0	0	0	0	0	1	1	0	1	5
**3.899**	0	1	0	0	1	0	0	0	0	0	0	1	1	1	0	0	0	0	0	0	0	0	1	0	0	5
**3.900**	0	1	0	0	1	0	0	0	0	0	0	0	0	0	0	0	0	0	0	1	0	1	0	0	1	5
**3.901**	0	0	1	0	0	0	0	0	0	0	1	0	0	0	0	1	0	0	0	0	0	1	0	0	0	5
**3.902**	0	0	0	0	1	0	0	0	0	0	0	0	1	0	0	1	0	0	0	1	0	0	0	1	0	5
**3.903**	0	0	0	0	0	0	0	0	0	1	0	0	0	1	0	0	0	0	0	1	0	0	1	1	0	5

CMP	CD2	CD3	CD4	CD7	CD8	CD10	CD13	CD18	CD26	CD29	CD36	CD44	CD45RA	CD49f	CD54	CD56	CD57	CD58	CD62L	CD71	CD80	Cytokeratin	Histone_H1	HLA-DQ	HLA-DR	Freq. (voxel)
**3.904**	0	0	0	0	0	0	0	0	0	1	0	0	0	0	0	0	0	0	0	0	0	1	1	0	1	5
**3.905**	0	1	0	1	1	0	1	0	1	0	0	1	0	0	0	0	0	0	0	1	0	1	0	0	1	5
**3.906**	0	0	1	0	0	0	0	0	0	1	0	1	0	1	0	0	0	0	0	0	0	1	1	1	0	5
**3.907**	0	0	0	1	0	0	0	0	0	1	0	0	0	0	0	0	0	0	0	0	0	0	0	1	1	5
**3.908**	0	0	0	0	0	0	0	0	0	0	1	1	0	1	0	0	0	0	0	0	0	0	0	1	0	5
**3.909**	0	1	0	1	1	0	1	0	0	1	0	1	0	0	0	0	0	0	0	1	0	0	0	0	0	5
**3.910**	0	1	0	1	1	0	1	0	0	0	0	1	1	0	0	0	0	0	0	1	0	0	0	0	0	5
**3.911**	0	1	0	1	1	0	0	0	1	0	0	0	0	0	0	0	0	0	0	0	0	0	1	0	1	5
**3.912**	0	1	0	1	1	0	0	0	0	0	0	1	1	0	0	0	0	0	0	0	0	1	0	1	0	5
**3.913**	0	1	0	0	0	0	1	0	0	1	0	1	1	0	0	0	0	0	0	0	0	0	1	0	0	5
**3.914**	0	1	0	0	0	0	0	0	1	1	0	0	0	0	0	0	0	0	0	1	0	0	0	0	0	5
**3.915**	0	0	1	0	0	0	0	0	0	0	0	0	0	0	0	0	0	0	0	0	0	1	1	1	0	5
**3.916**	0	0	0	0	1	0	0	0	0	1	0	0	0	1	0	0	0	0	0	0	0	0	0	0	0	5
**3.917**	0	0	0	0	0	0	0	0	0	1	0	0	0	1	0	0	0	0	0	0	0	1	1	1	0	5
**3.918**	0	0	1	0	0	0	0	0	0	0	0	0	1	1	0	1	0	0	0	1	0	0	0	1	0	5
**3.919**	0	0	1	0	0	0	0	0	0	0	0	0	1	0	0	1	0	0	0	0	0	0	1	1	0	5
**3.920**	0	1	0	0	1	0	1	0	0	1	0	1	1	0	0	0	0	0	0	0	0	0	1	0	0	5
**3.921**	0	0	0	0	1	0	1	0	1	1	0	0	1	0	0	1	0	0	0	1	0	1	0	0	0	5
**3.922**	0	1	1	1	1	0	1	0	1	0	0	1	0	0	0	0	0	0	0	1	0	0	0	0	1	4
**3.923**	0	1	1	0	0	0	0	0	0	0	0	1	0	0	0	0	0	0	0	1	0	0	0	1	0	4
**3.924**	0	1	0	1	0	0	0	0	0	0	0	0	1	0	0	0	0	0	0	1	0	0	0	1	0	4
**3.925**	0	0	1	0	0	0	1	0	1	0	0	1	0	0	0	0	0	0	0	0	0	1	0	1	0	4
**3.926**	0	0	0	1	1	0	1	0	1	0	0	1	1	0	0	0	0	0	0	0	0	0	0	1	1	4
**3.927**	0	0	0	1	1	0	1	0	1	0	0	1	0	0	0	0	0	0	0	0	0	0	0	1	1	4
**3.928**	0	0	0	1	1	0	0	0	1	1	0	1	0	0	0	0	0	0	0	1	0	0	0	1	0	4
**3.929**	0	0	0	1	0	0	1	0	1	0	0	1	0	1	0	0	0	0	0	1	0	1	0	1	1	4
**3.930**	0	0	0	0	1	0	0	0	1	1	0	1	0	0	0	0	0	0	0	0	0	1	0	1	0	4
**3.931**	0	0	0	0	1	0	0	0	1	0	0	0	0	0	0	0	0	0	0	0	0	1	0	1	0	4
**3.932**	0	0	0	0	0	0	1	0	1	1	0	0	0	0	0	0	0	0	0	0	0	0	0	0	1	4
**3.933**	0	0	0	0	0	0	1	0	1	0	0	1	0	0	0	0	0	0	0	0	0	1	0	1	0	4
**3.934**	0	1	1	1	1	0	1	0	1	0	0	1	0	0	0	1	0	0	0	0	0	0	0	1	1	4
**3.935**	0	1	1	0	1	0	0	0	1	1	0	1	1	1	0	0	0	0	0	1	0	0	1	0	0	4
**3.936**	0	1	1	0	0	0	0	0	1	1	0	1	1	0	0	0	0	0	0	1	0	1	0	1	1	4
**3.937**	0	1	0	1	1	0	0	0	1	0	0	1	1	0	0	0	0	0	0	1	0	1	1	0	0	4
**3.938**	0	1	0	1	0	0	1	0	1	1	0	1	0	0	0	0	0	0	0	0	0	0	0	1	1	4
**3.939**	0	1	0	0	1	0	1	0	1	1	0	1	1	1	0	0	0	0	0	1	0	0	0	1	0	4
**3.940**	0	1	0	0	1	0	1	0	1	1	0	1	1	0	0	0	0	0	0	1	0	1	0	1	1	4
**3.941**	0	1	0	0	1	0	1	0	1	1	0	1	1	0	0	0	0	0	0	1	0	0	1	0	0	4
**3.942**	0	1	0	0	1	0	0	0	0	1	0	0	1	0	0	0	0	0	0	0	0	0	0	0	0	4
**3.943**	0	0	1	1	0	0	0	0	0	0	0	0	1	0	0	0	0	0	0	1	0	1	0	0	1	4
**3.944**	0	0	1	0	1	0	1	0	0	0	0	1	0	0	0	0	0	0	0	0	0	0	0	1	1	4
**3.945**	0	0	1	0	0	0	0	0	0	0	0	1	1	0	0	0	0	0	0	1	0	0	0	1	1	4
**3.946**	0	0	1	0	0	0	0	0	0	0	0	0	1	0	0	0	0	0	0	1	0	0	0	0	1	4
**3.947**	0	0	0	1	1	0	0	0	1	1	0	1	1	0	0	0	0	0	0	1	0	0	1	0	0	4
**3.948**	0	0	0	1	1	0	0	0	1	1	0	1	1	0	0	0	0	0	0	1	0	0	0	0	1	4
**3.949**	0	0	0	1	1	0	0	0	1	1	0	0	0	0	0	0	0	0	0	1	0	0	1	0	0	4
**3.950**	0	0	0	1	0	0	0	0	1	0	0	0	0	1	0	0	0	0	0	0	0	1	0	1	0	4
**3.951**	0	0	0	1	0	0	0	0	0	1	0	1	0	1	0	0	0	0	0	0	0	0	0	1	0	4
**3.952**	0	0	0	0	1	0	1	0	0	1	0	1	1	0	0	0	0	0	0	1	0	1	0	0	0	4
**3.953**	0	0	0	0	1	0	0	0	1	1	0	1	0	0	0	0	0	0	0	0	0	1	0	0	1	4
**3.954**	0	0	0	0	1	0	0	0	1	0	0	0	0	0	0	0	0	0	0	1	0	1	1	0	0	4
**3.955**	0	0	0	0	1	0	0	0	0	0	0	1	1	0	0	0	0	0	0	0	0	1	1	1	1	4
**3.956**	0	0	0	0	0	0	1	0	1	1	0	1	0	0	0	0	0	0	0	0	0	1	0	1	1	4
**3.957**	0	0	0	0	0	0	0	0	1	0	0	0	0	1	0	0	0	0	0	1	0	0	0	0	0	4
**3.958**	0	0	0	0	0	0	0	0	0	1	0	0	1	0	0	0	0	0	0	0	0	0	1	1	0	4
**3.959**	0	1	1	1	1	0	1	0	0	1	0	1	1	0	0	0	0	0	0	1	0	1	0	1	1	4
**3.960**	0	1	1	1	1	0	0	0	1	1	0	1	0	1	0	0	0	0	0	1	0	0	0	0	0	4
**3.961**	0	1	1	1	1	0	0	0	0	1	0	1	1	1	0	0	0	0	0	1	0	0	0	0	0	4
**3.962**	0	1	1	0	1	0	0	0	0	0	0	1	0	0	0	0	0	0	0	1	0	1	0	1	1	4
**3.963**	0	1	1	0	0	0	0	0	0	1	0	1	0	0	0	0	0	0	0	1	0	1	1	1	1	4
**3.964**	0	1	0	1	1	0	0	0	0	1	0	1	0	1	0	0	0	0	0	0	0	0	1	0	0	4
**3.965**	0	1	0	1	0	0	0	0	0	0	0	0	1	0	0	0	0	0	0	0	0	0	0	1	0	4
**3.966**	0	1	0	0	1	0	1	0	1	1	0	1	1	1	0	0	0	0	0	1	0	1	0	0	0	4
**3.967**	0	1	0	0	1	0	0	0	1	0	0	1	1	1	0	0	0	0	0	1	0	0	0	0	0	4
**3.968**	0	1	0	0	1	0	0	0	1	0	0	0	0	1	0	0	0	0	0	1	0	1	0	1	1	4
**3.969**	0	1	0	0	1	0	0	0	0	1	0	0	1	0	0	0	0	0	0	0	0	1	0	0	0	4
**3.970**	0	1	0	0	0	0	0	0	0	1	0	1	1	0	0	0	0	0	0	1	0	1	0	1	1	4
**3.971**	0	0	1	1	1	0	0	0	1	1	0	1	0	0	0	0	0	0	0	0	0	0	0	0	0	4
**3.972**	0	0	1	1	1	0	0	0	0	1	0	1	0	0	0	0	0	0	0	0	0	0	0	0	1	4
**3.973**	0	0	1	1	1	0	0	0	0	0	0	1	0	0	0	0	0	0	0	0	0	0	0	0	0	4
**3.974**	0	0	1	0	1	0	0	0	0	1	0	1	0	0	0	0	0	0	0	0	0	1	0	1	0	4
**3.975**	0	0	0	1	1	0	0	0	0	1	0	1	0	0	0	0	0	0	0	0	0	1	1	0	0	4
**3.976**	0	0	0	1	1	0	0	0	0	1	0	0	0	0	0	0	0	0	0	1	0	0	1	0	0	4
**3.977**	0	0	0	1	0	0	1	0	1	1	0	1	1	0	0	0	0	0	0	1	0	0	0	0	0	4
**3.978**	0	0	0	1	0	0	1	0	0	1	0	1	0	0	0	0	0	0	0	1	0	1	0	0	0	4
**3.979**	0	0	0	1	0	0	0	0	1	1	0	1	0	0	0	0	0	0	0	1	0	1	0	1	1	4
**3.980**	0	0	0	1	0	0	0	0	1	0	0	0	0	0	0	0	0	0	0	0	0	1	0	1	1	4
**3.981**	0	0	0	1	0	0	0	0	0	0	0	1	1	0	0	1	0	0	0	0	0	0	0	1	0	4
**3.982**	0	0	0	0	1	0	0	0	0	0	0	1	1	0	0	0	0	0	0	0	0	0	1	1	1	4
**3.983**	0	0	0	0	0	0	1	0	0	0	0	1	0	0	0	0	0	0	0	1	0	1	0	1	0	4
**3.984**	0	0	0	0	0	0	1	0	0	0	0	0	0	0	0	1	0	0	0	0	0	0	0	0	0	4
**3.985**	0	0	0	0	0	0	0	0	1	1	0	0	0	0	0	0	0	0	0	0	0	1	1	0	0	4
**3.986**	0	0	0	0	0	0	0	0	1	0	1	0	0	0	0	0	0	0	0	1	0	1	0	0	1	4
**3.987**	0	0	0	0	0	0	0	0	1	0	0	1	1	0	0	0	0	0	0	1	0	1	0	1	0	4
**3.988**	0	0	0	0	0	0	0	0	1	0	0	1	0	1	0	0	0	0	0	1	0	0	0	1	0	4
**3.989**	0	0	0	0	0	0	0	0	0	0	0	1	1	0	0	0	0	0	0	1	0	1	1	0	0	4
**3.990**	0	1	1	1	0	0	0	0	1	0	0	1	0	1	0	0	0	0	0	0	0	0	0	0	0	4
**3.991**	0	1	1	0	1	0	0	0	1	1	0	1	0	0	0	0	0	0	0	1	0	0	1	0	0	4
**3.992**	0	1	1	0	1	0	0	0	1	1	0	1	0	0	0	0	0	0	0	0	0	0	1	0	0	4
**3.993**	0	1	0	1	1	0	0	0	1	1	0	1	0	0	0	0	0	0	0	0	0	0	1	1	1	4
**3.994**	0	1	0	1	1	0	0	0	1	0	0	1	1	1	0	0	0	0	0	0	0	0	0	0	0	4
**3.995**	0	1	0	1	1	0	0	0	1	0	0	1	1	0	0	0	0	0	0	1	0	1	1	1	1	4
**3.996**	0	1	0	1	1	0	0	0	0	1	0	1	0	0	0	1	0	0	0	0	0	0	0	0	0	4
**3.997**	0	1	0	1	1	0	0	0	0	0	0	1	0	1	0	0	0	0	0	0	0	1	0	1	1	4
**3.998**	0	1	0	1	1	0	0	0	0	0	0	0	1	0	0	0	0	0	0	0	0	0	0	1	1	4
**3.999**	0	1	0	1	0	0	1	0	0	0	0	1	0	0	0	0	0	0	0	0	0	0	1	0	1	4
**4.000**	0	1	0	1	0	0	0	0	1	1	0	1	0	1	0	0	0	0	0	1	0	1	0	1	1	4
**4.001**	0	1	0	0	1	0	1	0	0	1	0	1	1	1	0	0	0	0	0	1	0	1	1	0	0	4
**4.002**	0	1	0	0	1	0	0	0	0	1	0	1	1	1	0	0	0	0	0	0	0	1	0	1	1	4
**4.003**	0	1	0	0	1	0	0	0	0	0	0	0	1	0	0	0	0	0	0	1	0	0	0	1	1	4
**4.004**	0	1	0	0	0	0	0	0	1	1	0	1	0	0	0	0	0	0	0	0	0	1	1	1	0	4
**4.005**	0	1	0	0	0	0	0	0	1	0	0	1	1	0	0	0	0	0	0	1	0	0	0	1	1	4
**4.006**	0	0	1	1	0	0	0	0	1	1	0	1	1	0	0	0	0	0	0	1	0	0	0	0	0	4
**4.007**	0	0	1	1	0	0	0	0	1	0	0	1	1	0	0	0	0	0	0	1	0	0	0	1	1	4
**4.008**	0	0	1	0	1	0	1	0	0	0	0	0	1	0	0	0	0	0	0	1	0	1	0	1	0	4
**4.009**	0	0	0	1	1	0	0	0	1	1	0	1	0	0	0	0	0	0	0	1	0	1	0	1	1	4
**4.010**	0	0	0	1	0	0	0	0	0	1	0	1	1	1	0	0	0	0	0	1	0	1	1	1	0	4
**4.011**	0	0	0	1	0	0	0	0	0	1	0	1	1	0	0	0	0	0	0	0	0	0	1	0	0	4
**4.012**	0	0	0	1	0	0	0	0	0	1	0	0	1	0	0	0	0	0	0	1	0	0	0	0	0	4
**4.013**	0	0	0	1	0	0	0	0	0	0	0	1	0	1	0	0	0	0	0	1	0	0	0	1	0	4
**4.014**	0	0	0	1	0	0	0	0	0	0	0	1	0	0	0	0	0	0	0	1	0	1	0	1	1	4
**4.015**	0	0	0	1	0	0	0	0	0	0	0	1	0	0	0	0	0	0	0	0	0	0	1	1	0	4
**4.016**	0	0	0	0	1	0	1	0	1	1	0	1	0	0	0	0	0	0	0	1	0	1	0	0	0	4
**4.017**	0	0	0	0	1	0	0	0	0	1	0	0	1	0	0	0	0	0	0	1	0	0	0	0	0	4
**4.018**	0	0	0	0	1	0	0	0	0	0	0	0	1	0	0	0	0	0	0	1	0	1	0	0	0	4
**4.019**	0	0	0	0	0	0	0	0	1	1	0	0	0	0	0	0	0	0	0	1	0	1	0	1	0	4
**4.020**	0	0	0	0	0	0	0	0	1	0	0	0	1	0	0	0	0	0	0	1	0	1	0	1	1	4
**4.021**	0	0	0	0	0	0	0	0	0	1	0	1	1	0	0	0	0	0	0	1	0	0	0	1	0	4
**4.022**	0	0	0	0	0	0	0	0	0	0	0	1	1	1	0	0	0	0	0	1	0	1	0	1	1	4
**4.023**	0	1	1	1	1	0	0	0	0	0	0	1	0	0	0	0	0	0	0	1	0	0	0	1	0	4
**4.024**	0	1	1	0	1	0	0	0	0	0	0	1	0	0	0	0	0	0	0	1	0	1	0	1	0	4
**4.025**	0	1	1	0	1	0	0	0	0	0	0	0	0	0	0	0	0	0	0	1	0	0	0	0	1	4

CMP	CD2	CD3	CD4	CD7	CD8	CD10	CD13	CD18	CD26	CD29	CD36	CD44	CD45RA	CD49f	CD54	CD56	CD57	CD58	CD62L	CD71	CD80	Cytokeratin	Histone_H1	HLA-DQ	HLA-DR	Freq. (voxel)
**4.026**	0	1	1	0	0	0	0	0	0	1	0	1	1	0	0	0	0	0	0	1	0	0	1	0	0	4
**4.027**	0	1	0	1	1	0	0	0	0	0	0	1	1	0	0	0	0	0	0	1	0	1	1	1	0	4
**4.028**	0	1	0	1	0	0	1	0	0	0	0	1	0	0	0	0	0	0	0	0	0	0	0	0	1	4
**4.029**	0	1	0	1	0	0	1	0	0	0	0	0	1	0	0	0	0	0	0	1	0	0	1	0	0	4
**4.030**	0	1	0	1	0	0	0	0	1	0	0	1	0	1	0	0	0	0	0	1	0	0	1	0	0	4
**4.031**	0	1	0	1	0	0	0	0	0	1	0	1	0	0	0	0	0	0	0	1	0	1	1	0	1	4
**4.032**	0	1	0	1	0	0	0	0	0	1	0	1	0	0	0	0	0	0	0	0	0	1	0	1	1	4
**4.033**	0	1	0	1	0	0	0	0	0	0	0	1	0	1	0	0	0	0	0	0	0	1	0	1	1	4
**4.034**	0	1	0	1	0	0	0	0	0	0	0	1	0	0	0	0	0	0	0	0	0	1	0	1	1	4
**4.035**	0	1	0	1	0	0	0	0	0	0	0	1	0	0	0	0	0	0	0	0	0	1	0	0	1	4
**4.036**	0	1	0	0	1	0	1	0	0	0	0	0	0	0	0	0	0	0	0	0	0	0	1	0	1	4
**4.037**	0	1	0	0	0	0	0	0	1	0	0	1	0	0	0	0	0	0	0	1	0	0	1	1	1	4
**4.038**	0	1	0	0	0	0	0	0	0	1	0	0	1	0	0	0	0	0	0	0	0	0	1	0	0	4
**4.039**	0	1	0	0	0	0	0	0	0	0	0	1	1	0	0	0	0	0	0	1	0	1	0	1	1	4
**4.040**	0	1	0	0	0	0	0	0	0	0	0	1	0	1	0	0	0	0	0	1	0	0	1	1	0	4
**4.041**	0	1	0	0	0	0	0	0	0	0	0	0	0	1	0	0	0	0	0	1	0	1	1	1	1	4
**4.042**	0	0	1	1	0	0	0	0	0	0	0	0	1	1	0	1	0	0	0	1	0	0	0	0	0	4
**4.043**	0	0	1	1	0	0	0	0	0	0	0	0	1	1	0	0	0	0	0	1	0	0	0	0	0	4
**4.044**	0	0	1	0	0	0	1	0	0	0	0	0	1	0	0	0	0	0	0	1	0	0	0	0	0	4
**4.045**	0	0	1	0	0	0	0	0	1	0	0	0	1	0	0	0	0	0	0	0	0	0	0	1	1	4
**4.046**	0	0	1	0	0	0	0	0	0	1	0	1	0	1	0	0	0	0	0	1	0	0	1	0	1	4
**4.047**	0	0	1	0	0	0	0	0	0	1	0	0	0	0	0	0	0	0	0	1	0	1	1	0	0	4
**4.048**	0	0	1	0	0	0	0	0	0	0	0	0	0	0	0	1	0	0	0	0	0	0	1	1	1	4
**4.049**	0	0	0	1	1	0	1	0	0	1	0	1	0	1	0	0	0	0	0	1	0	1	0	1	1	4
**4.050**	0	0	0	1	0	0	0	0	0	0	0	1	0	1	0	0	0	0	0	1	0	0	0	0	0	4
**4.051**	0	0	0	0	1	0	1	0	1	0	0	0	0	0	0	0	0	0	0	1	0	0	0	1	1	4
**4.052**	0	0	0	0	1	0	0	0	0	0	0	1	0	1	0	0	0	0	0	0	0	1	0	0	1	4
**4.053**	0	0	0	0	1	0	0	0	0	0	0	1	0	1	0	0	0	0	0	0	0	0	0	1	1	4
**4.054**	0	0	0	0	0	0	1	0	0	0	0	0	0	1	0	1	0	0	0	1	0	0	0	1	1	4
**4.055**	0	0	0	0	0	0	0	0	0	1	0	1	1	1	0	1	0	0	0	1	0	1	1	0	0	4
**4.056**	0	0	0	0	0	0	0	0	0	0	1	1	0	1	0	0	0	0	0	1	0	0	0	0	0	4
**4.057**	0	1	1	1	1	0	0	0	0	1	0	1	0	0	0	0	0	0	0	1	0	0	1	0	0	4
**4.058**	0	1	1	1	0	0	0	0	1	1	0	1	1	1	0	0	0	0	0	1	0	0	0	0	0	4
**4.059**	0	1	1	1	0	0	0	0	0	0	0	1	0	1	0	0	0	0	0	0	0	0	0	0	0	4
**4.060**	0	1	1	0	1	0	0	0	1	1	0	1	0	0	0	0	0	0	0	1	0	0	0	0	1	4
**4.061**	0	1	1	0	1	0	0	0	1	0	0	0	0	0	0	0	0	0	0	0	0	0	0	1	1	4
**4.062**	0	1	1	0	1	0	0	0	0	0	0	0	0	0	0	0	0	0	0	1	0	0	0	0	0	4
**4.063**	0	1	1	0	0	0	1	0	0	0	0	1	1	0	0	0	0	0	0	1	0	0	1	0	0	4
**4.064**	0	1	1	0	0	0	0	0	0	1	0	1	0	1	0	0	0	0	0	1	0	0	1	0	0	4
**4.065**	0	1	1	0	0	0	0	0	0	1	0	1	0	0	0	0	0	0	0	0	0	0	0	1	0	4
**4.066**	0	1	1	0	0	0	0	0	0	0	0	1	1	0	0	0	0	0	0	1	0	0	1	0	0	4
**4.067**	0	1	1	0	0	0	0	0	0	0	0	1	0	0	0	0	0	0	0	0	0	0	1	0	1	4
**4.068**	0	1	0	1	1	0	0	0	0	0	1	1	0	0	0	0	0	0	0	1	0	1	0	1	1	4
**4.069**	0	1	0	1	1	0	0	0	0	0	0	1	0	1	0	0	0	0	0	1	0	0	1	0	0	4
**4.070**	0	1	0	1	0	0	1	0	0	0	0	1	0	0	0	0	0	0	0	0	0	0	0	1	0	4
**4.071**	0	1	0	1	0	0	0	0	1	1	0	1	1	1	0	0	0	0	0	1	0	0	0	0	1	4
**4.072**	0	1	0	1	0	0	0	0	0	1	0	0	1	0	0	0	0	0	0	1	0	0	1	0	0	4
**4.073**	0	1	0	1	0	0	0	0	0	0	1	1	0	0	0	0	0	0	0	1	0	1	0	1	1	4
**4.074**	0	1	0	0	1	0	0	0	0	1	0	0	0	0	0	0	0	0	0	1	0	0	0	0	0	4
**4.075**	0	1	0	0	0	0	1	0	1	0	0	1	0	1	0	0	0	0	0	1	0	1	0	1	1	4
**4.076**	0	1	0	0	0	0	1	0	1	0	0	1	0	0	0	0	0	0	0	0	0	0	0	0	0	4
**4.077**	0	1	0	0	0	0	1	0	0	0	0	1	0	1	0	0	0	0	0	1	0	1	0	1	1	4
**4.078**	0	1	0	0	0	0	0	0	0	1	0	1	0	0	0	0	0	0	0	0	0	1	0	1	0	4
**4.079**	0	1	0	0	0	0	0	0	0	0	0	1	1	1	0	0	0	0	0	1	0	0	0	0	0	4
**4.080**	0	0	1	1	0	0	0	0	0	0	0	0	1	0	0	1	0	0	0	1	0	1	0	1	0	4
**4.081**	0	0	1	1	0	0	0	0	0	0	0	0	1	0	0	0	0	0	0	1	0	1	0	1	0	4
**4.082**	0	0	1	0	0	0	1	0	0	1	0	1	1	1	0	0	0	0	0	1	0	0	0	1	0	4
**4.083**	0	0	1	0	0	0	0	0	0	1	1	1	0	0	0	0	0	0	0	0	0	0	0	0	0	4
**4.084**	0	0	1	0	0	0	0	0	0	1	0	1	1	1	0	0	0	0	0	1	0	1	0	0	0	4
**4.085**	0	0	1	0	0	0	0	0	0	0	0	1	0	1	0	0	0	0	0	1	0	0	0	1	0	4
**4.086**	0	0	1	0	0	0	0	0	0	0	0	1	0	1	0	0	0	0	0	0	0	0	1	0	0	4
**4.087**	0	0	1	0	0	0	0	0	0	0	0	1	0	0	0	0	0	0	0	0	0	1	1	0	1	4
**4.088**	0	0	1	0	0	0	0	0	0	0	0	0	0	1	0	0	0	0	0	1	0	1	0	1	0	4
**4.089**	0	0	0	1	1	0	0	0	0	1	0	0	0	0	0	0	0	0	0	0	0	0	0	0	0	4
**4.090**	0	0	0	0	1	0	0	0	0	1	0	1	1	1	0	0	0	0	0	1	0	1	1	0	0	4
**4.091**	0	0	0	0	0	0	1	0	0	0	0	0	1	1	0	1	0	0	0	0	0	0	0	0	0	4
**4.092**	0	0	0	0	0	0	0	0	0	0	1	1	0	0	0	0	0	0	0	1	0	1	0	1	0	4
**4.093**	0	0	0	0	0	0	0	0	0	0	1	0	1	0	0	0	0	0	0	0	0	0	0	1	0	4
**4.094**	0	1	1	1	1	0	0	0	1	0	0	1	0	0	0	0	0	0	0	0	0	0	0	1	1	4
**4.095**	0	1	1	1	1	0	0	0	0	0	0	1	0	0	0	0	0	0	0	0	0	0	0	0	1	4
**4.096**	0	1	1	0	1	0	0	0	0	0	0	1	1	0	0	0	0	0	0	1	0	0	0	1	1	4
**4.097**	0	1	0	1	1	0	0	0	0	1	0	1	1	1	0	0	0	0	0	0	0	1	0	1	1	4
**4.098**	0	1	0	1	0	0	1	0	0	0	0	0	0	0	0	0	0	0	0	1	0	0	0	0	0	4
**4.099**	0	1	0	1	0	0	0	0	0	1	0	1	1	1	0	0	0	0	0	0	0	0	0	1	1	4
**4.100**	0	1	0	1	0	0	0	0	0	0	0	0	1	0	0	0	0	0	0	0	0	1	1	1	0	4
**4.101**	0	1	0	1	0	0	0	0	0	0	0	0	0	1	0	0	0	0	0	0	0	1	0	1	0	4
**4.102**	0	1	0	0	1	0	0	0	0	0	0	1	1	0	0	0	0	0	0	0	0	1	1	1	0	4
**4.103**	0	1	0	0	1	0	0	0	0	0	0	1	0	1	0	0	0	0	0	1	0	0	0	1	1	4
**4.104**	0	1	0	0	0	0	0	0	0	1	0	1	0	1	0	0	0	0	0	1	0	1	0	0	0	4
**4.105**	0	1	0	0	0	0	0	0	0	0	0	0	0	0	0	0	0	0	0	1	0	1	0	1	1	4
**4.106**	0	0	1	1	0	0	1	0	1	1	0	1	0	1	0	1	0	0	0	0	0	0	0	1	1	4
**4.107**	0	0	1	1	0	0	0	0	0	0	0	1	0	0	0	0	0	0	0	1	0	1	0	0	0	4
**4.108**	0	0	1	1	0	0	0	0	0	0	0	1	0	0	0	0	0	0	0	0	0	0	0	1	0	4
**4.109**	0	0	1	0	1	0	1	0	0	0	0	0	1	0	0	1	0	0	0	1	0	0	0	1	0	4
**4.110**	0	0	1	0	0	0	1	0	1	0	0	0	0	0	0	0	0	0	0	0	0	0	0	0	0	4
**4.111**	0	0	1	0	0	0	1	0	0	1	1	1	0	0	0	0	0	0	0	1	0	0	0	1	1	4
**4.112**	0	0	1	0	0	0	1	0	0	0	0	0	0	1	0	0	0	0	0	0	0	0	0	1	0	4
**4.113**	0	0	1	0	0	0	0	0	1	0	0	0	0	0	0	0	0	0	0	0	0	0	1	1	0	4
**4.114**	0	0	1	0	0	0	0	0	0	0	0	1	0	1	0	0	0	0	0	0	0	1	1	0	1	4
**4.115**	0	0	1	0	0	0	0	0	0	0	0	0	1	0	0	0	0	0	0	0	0	1	1	0	0	4
**4.116**	0	0	0	1	1	0	1	0	0	1	0	1	0	1	0	0	0	0	0	1	0	1	0	0	0	4
**4.117**	0	0	0	1	0	0	0	0	0	0	1	0	0	1	0	0	0	0	0	1	0	1	0	0	0	4
**4.118**	0	0	0	1	0	0	0	0	0	0	0	0	0	0	0	0	0	0	0	0	0	1	1	1	0	4
**4.119**	0	0	0	0	0	0	1	0	1	1	0	1	1	1	0	0	0	0	0	1	0	1	0	0	0	4
**4.120**	0	0	0	0	0	0	1	0	1	0	0	1	0	1	0	0	0	0	0	1	0	0	1	1	1	4
**4.121**	0	0	0	0	0	0	1	0	0	1	0	0	0	1	0	0	0	0	0	0	0	1	0	1	0	4
**4.122**	0	0	0	0	0	0	0	0	0	0	1	1	0	0	0	0	0	0	0	0	0	0	1	1	1	4
**4.123**	0	1	1	0	1	0	0	0	1	0	0	1	1	0	0	0	0	0	0	1	0	0	0	1	0	4
**4.124**	0	1	1	0	1	0	0	0	1	0	0	0	1	0	0	0	0	0	0	0	0	0	0	1	1	4
**4.125**	0	1	0	1	1	0	0	0	1	0	0	1	1	0	0	0	0	0	0	0	0	0	1	0	1	4
**4.126**	0	1	0	1	0	0	0	0	0	0	0	0	0	1	0	0	0	0	0	0	0	0	0	1	1	4
**4.127**	0	1	0	0	1	0	0	0	1	0	0	0	1	0	0	0	0	0	0	0	0	0	0	1	0	4
**4.128**	0	1	0	0	1	0	0	0	0	0	0	0	0	0	0	0	0	0	0	0	0	1	1	1	1	4
**4.129**	0	1	0	0	0	0	1	0	0	0	0	0	0	0	0	0	0	0	0	0	0	1	1	1	1	4
**4.130**	0	1	0	0	0	0	0	0	0	1	0	1	1	1	0	0	0	0	0	0	0	0	0	1	0	4
**4.131**	0	1	0	0	0	0	0	0	0	0	0	1	1	0	0	0	0	0	0	0	0	0	1	1	1	4
**4.132**	0	1	0	0	0	0	0	0	0	0	0	0	0	1	0	0	0	0	0	0	0	1	0	1	1	4
**4.133**	0	0	1	1	0	0	1	0	0	1	0	1	0	1	0	1	0	0	0	0	0	0	0	1	1	4
**4.134**	0	0	1	0	0	0	1	0	0	1	0	1	0	1	0	1	0	0	0	1	0	0	0	1	0	4
**4.135**	0	0	1	0	0	0	1	0	0	0	1	0	1	0	0	1	0	0	0	0	0	0	0	0	0	4
**4.136**	0	0	1	0	0	0	0	0	0	1	0	1	1	0	0	0	0	0	0	0	0	0	0	0	1	4
**4.137**	0	0	1	0	0	0	0	0	0	1	0	1	0	1	0	0	0	0	0	0	0	0	1	0	0	4
**4.138**	0	0	0	1	0	0	1	0	0	1	0	0	0	1	0	0	0	0	0	0	0	1	1	0	0	4
**4.139**	0	0	0	0	0	0	1	0	0	1	1	1	0	1	0	0	0	0	0	1	0	1	0	0	0	4
**4.140**	0	0	0	0	0	0	0	0	1	1	0	0	0	1	0	0	0	0	0	0	0	1	1	0	0	4
**4.141**	0	1	1	1	1	0	0	0	1	1	0	0	1	0	0	0	0	0	0	0	0	0	0	1	0	4
**4.142**	0	1	1	1	1	0	0	0	1	0	0	0	1	0	0	0	0	0	0	0	0	0	0	1	0	4
**4.143**	0	1	1	1	1	0	0	0	0	0	0	1	1	0	0	0	0	0	0	1	0	0	0	1	1	4
**4.144**	0	1	1	1	0	0	0	0	0	1	0	1	0	1	0	0	0	0	0	0	0	0	0	0	0	4
**4.145**	0	1	1	1	0	0	0	0	0	0	0	1	0	0	0	0	0	0	0	1	0	0	1	0	0	4
**4.146**	0	1	1	0	1	0	0	0	0	1	0	1	1	0	0	0	0	0	0	0	0	0	1	0	1	4
**4.147**	0	1	1	0	1	0	0	0	0	0	0	1	1	0	0	0	0	0	0	0	0	1	0	0	1	4

CMP	CD2	CD3	CD4	CD7	CD8	CD10	CD13	CD18	CD26	CD29	CD36	CD44	CD45RA	CD49f	CD54	CD56	CD57	CD58	CD62L	CD71	CD80	Cytokeratin	Histone_H1	HLA-DQ	HLA-DR	Freq. (voxel)
**4.148**	0	1	0	1	1	0	0	0	0	0	0	1	1	1	0	0	0	0	0	0	0	0	1	0	0	4
**4.149**	0	1	0	0	1	0	0	0	1	1	0	0	0	0	0	0	0	0	0	0	0	0	1	0	0	4
**4.150**	0	1	0	0	1	0	0	0	0	1	0	1	0	0	0	0	0	0	0	0	0	1	0	1	0	4
**4.151**	0	1	0	0	0	0	1	0	0	1	0	1	1	0	0	0	0	0	0	0	0	0	0	0	0	4
**4.152**	0	1	0	0	0	0	1	0	0	1	0	1	0	1	0	0	0	0	0	1	0	1	0	1	1	4
**4.153**	0	0	1	1	0	0	0	0	1	1	0	1	0	1	0	0	0	0	0	1	0	1	0	0	1	4
**4.154**	0	0	1	1	0	0	0	0	1	1	0	1	0	1	0	0	0	0	0	0	0	1	0	0	1	4
**4.155**	0	0	1	0	0	0	1	0	1	1	0	1	1	1	0	0	0	0	0	1	0	1	0	1	1	4
**4.156**	0	0	1	0	0	0	1	0	1	1	0	1	0	1	0	1	0	0	0	1	0	1	0	0	1	4
**4.157**	0	0	1	0	0	0	1	0	0	1	0	0	1	1	0	0	0	0	0	0	0	0	0	1	1	4
**4.158**	0	0	1	0	0	0	1	0	0	0	0	0	1	0	0	1	0	0	0	0	0	0	0	0	0	4
**4.159**	0	0	1	0	0	0	0	0	0	1	0	1	0	1	0	1	0	0	0	1	0	1	0	1	1	4
**4.160**	0	0	1	0	0	0	0	0	0	0	0	0	0	0	0	1	0	0	0	0	0	1	0	1	1	4
**4.161**	0	0	0	1	1	0	0	0	0	0	0	0	1	0	0	0	0	0	0	0	0	0	0	0	1	4
**4.162**	0	0	0	1	0	0	1	0	0	1	0	1	0	1	0	0	0	0	0	1	0	1	1	1	0	4
**4.163**	0	0	0	0	1	0	1	0	0	1	1	1	0	1	0	0	0	0	0	0	0	1	0	0	0	4
**4.164**	0	0	0	0	1	0	0	0	0	0	0	1	1	0	0	0	0	0	0	0	0	0	1	0	1	4
**4.165**	0	0	0	0	1	0	0	0	0	0	0	1	0	1	0	0	0	0	0	0	0	1	0	0	0	4
**4.166**	0	0	0	0	0	0	1	0	0	1	0	0	1	0	0	0	0	0	0	0	0	0	1	0	0	4
**4.167**	0	0	0	0	0	0	1	0	0	1	0	0	0	1	0	0	0	0	0	0	0	1	1	1	0	4
**4.168**	0	0	0	0	0	0	0	0	0	1	0	1	1	1	0	0	0	0	0	1	0	1	0	1	1	4
**4.169**	0	1	1	1	1	0	0	0	1	1	0	1	1	0	0	0	0	0	0	0	0	0	0	1	0	4
**4.170**	0	1	1	1	1	0	0	0	0	0	0	1	1	0	0	0	0	0	0	0	0	0	0	1	0	4
**4.171**	0	1	1	1	0	0	0	0	1	1	0	1	0	0	0	0	0	0	0	1	0	0	0	1	0	4
**4.172**	0	1	1	0	1	0	0	0	1	1	0	1	0	0	0	0	0	0	0	0	0	0	1	0	1	4
**4.173**	0	1	0	1	0	0	0	0	1	0	0	1	1	0	0	0	0	0	0	1	0	1	0	0	0	4
**4.174**	0	1	0	0	1	0	0	0	1	1	0	1	0	0	0	0	0	0	0	1	0	1	0	0	1	4
**4.175**	0	1	0	0	1	0	0	0	1	0	0	0	0	0	0	0	0	0	0	0	0	0	0	0	1	4
**4.176**	0	1	0	0	0	0	0	0	0	0	1	1	0	0	0	0	0	0	0	0	0	1	0	1	0	4
**4.177**	0	1	0	0	0	0	0	0	0	0	0	0	0	1	0	0	0	0	0	0	0	0	1	1	0	4
**4.178**	0	0	1	1	0	0	1	0	0	1	0	1	1	1	0	1	0	0	0	0	0	0	0	1	1	4
**4.179**	0	0	1	0	1	0	0	0	0	0	0	1	1	0	0	0	0	0	0	0	0	0	0	1	1	4
**4.180**	0	0	1	0	0	0	1	0	1	0	0	0	0	1	0	0	0	0	0	0	0	1	1	1	0	4
**4.181**	0	0	1	0	0	0	1	0	1	0	0	0	0	1	0	0	0	0	0	0	0	0	0	0	0	4
**4.182**	0	0	1	0	0	0	0	0	1	0	0	0	1	0	0	0	0	0	0	0	0	0	1	0	0	4
**4.183**	0	0	1	0	0	0	0	0	0	1	0	0	1	1	0	0	0	0	0	0	0	0	0	1	1	4
**4.184**	0	0	1	0	0	0	0	0	0	1	0	0	0	1	0	0	0	0	0	0	0	1	0	1	1	4
**4.185**	0	0	0	1	1	0	0	0	0	1	0	1	0	1	0	0	0	0	0	0	0	1	0	0	0	4
**4.186**	0	0	0	1	1	0	0	0	0	0	0	1	1	0	0	0	0	0	0	0	0	0	0	1	0	4
**4.187**	0	0	0	1	0	0	1	0	1	1	0	1	0	1	0	1	0	0	0	1	0	0	0	1	1	4
**4.188**	0	0	0	0	1	0	1	0	1	0	0	0	1	0	0	0	0	0	0	0	0	0	0	0	0	4
**4.189**	0	0	0	0	0	0	1	0	1	1	0	1	0	1	0	0	0	0	0	0	0	0	0	0	1	4
**4.190**	0	0	0	0	0	0	0	0	1	1	0	1	1	1	0	0	0	0	0	0	0	1	0	0	0	4
**4.191**	0	0	0	0	0	0	0	0	1	0	0	0	0	1	0	0	0	0	0	0	0	0	1	0	0	4
**4.192**	0	0	0	0	0	0	0	0	0	1	0	1	0	0	0	1	0	0	0	0	0	0	0	0	0	4
**4.193**	0	0	0	0	0	0	0	0	0	0	1	1	1	1	0	0	0	0	0	0	0	1	0	0	0	4
**4.194**	0	0	0	0	0	0	0	0	0	0	1	1	0	1	0	1	0	0	0	0	0	0	0	1	1	4
**4.195**	0	0	0	0	0	0	0	0	0	0	0	1	1	1	0	1	0	0	0	0	0	1	0	1	1	4
**4.196**	0	0	0	0	0	0	0	0	0	0	0	0	0	1	0	1	0	0	0	0	0	1	0	1	0	4
**4.197**	0	1	1	0	1	0	0	0	1	1	0	1	0	0	0	0	0	0	0	1	0	0	0	1	0	4
**4.198**	0	1	1	0	0	0	0	0	0	1	0	1	0	1	0	0	0	0	0	0	0	1	0	1	1	4
**4.199**	0	1	0	1	0	0	0	0	1	1	0	1	1	0	0	0	0	0	0	1	0	0	0	1	0	4
**4.200**	0	1	0	1	0	0	0	0	0	0	0	0	1	0	0	0	0	0	0	0	0	0	1	0	1	4
**4.201**	0	1	0	0	1	0	0	0	1	0	0	1	1	0	0	0	0	0	0	0	0	1	1	1	1	4
**4.202**	0	1	0	0	1	0	0	0	1	0	0	0	0	0	0	0	0	0	0	1	0	1	0	0	1	4
**4.203**	0	0	1	1	0	0	0	0	1	0	0	0	0	0	0	0	0	0	0	0	0	1	0	0	1	4
**4.204**	0	0	1	0	0	0	1	0	1	0	0	0	0	1	0	0	0	0	0	1	0	0	0	0	0	4
**4.205**	0	0	1	0	0	0	1	0	0	1	0	1	1	1	0	0	0	0	0	0	0	0	0	0	1	4
**4.206**	0	0	1	0	0	0	1	0	0	1	0	1	0	1	0	0	0	0	0	1	0	0	0	0	1	4
**4.207**	0	0	1	0	0	0	1	0	0	1	0	0	0	1	0	0	0	0	0	1	0	1	1	0	0	4
**4.208**	0	0	1	0	0	0	0	0	1	0	0	0	0	0	0	0	0	0	0	1	0	0	0	1	0	4
**4.209**	0	0	1	0	0	0	0	0	0	1	0	1	1	1	0	0	0	0	0	0	0	1	0	1	1	4
**4.210**	0	0	1	0	0	0	0	0	0	1	0	1	1	1	0	0	0	0	0	0	0	0	0	0	1	4
**4.211**	0	0	1	0	0	0	0	0	0	1	0	1	0	1	0	1	0	0	0	1	0	1	0	0	0	4
**4.212**	0	0	0	1	1	0	0	0	1	1	0	1	1	0	0	0	0	0	0	0	0	0	0	0	0	4
**4.213**	0	0	0	1	0	0	0	0	1	0	0	0	0	0	0	0	0	0	0	0	0	1	0	0	1	4
**4.214**	0	0	0	1	0	0	0	0	0	1	0	1	1	0	0	0	0	0	0	1	0	0	0	0	0	4
**4.215**	0	0	0	1	0	0	0	0	0	0	0	1	0	1	0	0	0	0	0	0	0	1	0	0	1	4
**4.216**	0	0	0	0	0	0	1	0	0	1	0	1	0	1	0	1	0	0	0	1	0	1	0	0	0	4
**4.217**	0	0	0	0	0	0	0	0	1	0	0	0	0	1	0	0	0	0	0	0	0	1	1	0	1	4
**4.218**	0	1	1	0	1	0	0	0	1	0	0	0	0	0	0	0	0	0	0	1	0	0	0	1	0	4
**4.219**	0	1	1	0	0	0	0	0	0	1	0	1	0	0	0	0	0	0	0	0	0	1	0	0	1	4
**4.220**	0	1	0	0	1	0	1	0	1	1	0	1	0	0	0	0	0	0	0	1	0	0	0	0	0	4
**4.221**	0	1	0	0	1	0	0	0	1	0	0	0	1	0	0	0	0	0	0	1	0	0	1	0	0	4
**4.222**	0	1	0	0	1	0	0	0	0	0	0	0	0	0	0	0	0	0	0	0	0	1	0	1	1	4
**4.223**	0	1	0	0	0	0	0	0	1	0	0	1	1	0	0	0	0	0	0	1	0	1	0	0	1	4
**4.224**	0	0	1	0	0	0	1	0	0	0	0	1	0	1	0	0	0	0	0	1	0	1	0	1	0	4
**4.225**	0	0	1	0	0	0	0	0	1	0	0	0	1	0	0	1	0	0	0	1	0	0	0	0	0	4
**4.226**	0	0	1	0	0	0	0	0	1	0	0	0	1	0	0	0	0	0	0	1	0	0	0	1	0	4
**4.227**	0	0	0	0	1	0	0	0	0	0	0	1	0	0	0	0	0	0	0	1	0	0	1	1	0	4
**4.228**	0	0	0	0	0	0	0	0	0	1	1	1	0	0	0	0	0	0	0	0	0	0	1	0	0	4
**4.229**	0	0	0	0	0	0	0	0	0	0	0	0	1	0	0	0	0	0	0	1	0	0	0	0	1	4
**4.230**	0	1	1	0	0	0	0	0	1	0	0	0	0	0	0	0	0	0	0	0	0	1	0	0	0	4
**4.231**	0	1	0	1	1	0	1	0	1	0	0	1	0	0	0	0	0	0	0	1	0	0	0	0	1	4
**4.232**	0	1	0	1	0	0	0	0	1	0	0	0	1	0	0	0	0	0	0	0	0	0	0	0	0	4
**4.233**	0	1	0	1	0	0	0	0	0	1	1	1	0	0	0	0	0	0	0	0	0	0	0	0	0	4
**4.234**	0	1	0	1	0	0	0	0	0	1	0	1	1	0	0	0	0	0	0	1	0	0	1	0	0	4
**4.235**	0	0	1	1	0	0	1	0	0	1	1	1	0	1	0	0	0	0	0	1	0	0	0	1	1	4
**4.236**	0	0	1	0	0	0	1	0	1	1	0	0	0	1	0	0	0	0	0	1	0	1	0	0	1	4
**4.237**	0	0	1	0	0	0	1	0	1	1	0	0	0	1	0	0	0	0	0	0	0	0	0	1	1	4
**4.238**	0	0	1	0	0	0	0	0	1	1	0	1	0	1	0	0	0	0	0	1	0	0	1	1	1	4
**4.239**	0	0	1	0	0	0	0	0	0	1	0	0	0	1	0	0	0	0	0	0	0	1	0	0	1	4
**4.240**	0	0	0	1	1	0	0	0	0	0	0	0	0	0	0	0	0	0	0	0	0	0	0	1	1	4
**4.241**	0	0	0	1	0	0	1	0	0	1	0	0	0	1	0	0	0	0	0	1	0	1	1	0	0	4
**4.242**	0	0	0	1	0	0	1	0	0	1	0	0	0	1	0	0	0	0	0	0	0	0	0	1	1	4
**4.243**	0	0	0	1	0	0	0	0	1	0	0	1	0	0	0	0	0	0	0	1	0	0	0	0	1	4
**4.244**	0	0	0	0	1	0	0	0	0	1	1	1	0	1	0	0	0	0	0	0	0	0	1	0	1	4
**4.245**	0	0	0	0	0	0	1	0	1	0	0	1	0	1	0	0	0	0	0	1	0	1	0	1	0	4
**4.246**	0	0	0	0	0	0	1	0	0	1	1	1	0	1	0	0	0	0	0	1	0	0	0	0	0	4
**4.247**	0	0	0	0	0	0	0	0	1	1	0	1	0	0	0	0	0	0	0	0	0	0	1	0	1	4
**4.248**	0	0	0	0	0	0	0	0	0	1	1	0	0	1	0	0	0	0	0	0	0	0	0	0	0	4
**4.249**	0	0	0	0	0	0	0	0	0	1	0	0	1	0	0	0	0	0	0	1	0	1	0	0	1	4
**4.250**	0	0	0	0	0	0	0	0	0	0	1	0	0	1	0	0	0	0	0	1	0	0	0	1	1	4
**4.251**	0	0	0	0	0	0	0	0	0	0	0	1	0	1	0	1	0	0	0	1	0	1	0	0	0	4
**4.252**	0	1	0	0	0	0	0	0	1	0	0	1	0	0	0	0	0	0	0	1	0	0	1	1	0	4
**4.253**	0	1	0	0	0	0	0	0	0	0	1	1	0	0	0	0	0	0	0	0	0	0	1	1	0	4
**4.254**	0	1	0	0	0	0	0	0	0	0	0	0	1	0	0	0	0	0	0	0	0	1	0	1	1	4
**4.255**	0	0	1	1	0	0	0	0	0	1	0	0	0	1	0	0	0	0	0	0	0	0	0	1	1	4
**4.256**	0	0	1	0	0	0	0	0	0	1	0	1	0	0	0	1	0	0	0	0	0	0	0	1	1	4
**4.257**	0	0	0	0	0	0	1	0	0	1	0	1	0	0	0	0	0	0	0	1	0	1	1	0	1	4
**4.258**	0	0	0	0	0	0	1	0	0	1	0	0	1	0	0	1	0	0	0	1	0	0	0	0	0	4
**4.259**	0	0	0	0	0	0	1	0	0	0	0	1	0	1	0	0	0	0	0	1	0	0	0	1	0	4
**4.260**	0	0	0	0	0	0	0	0	0	1	0	1	0	1	0	1	0	0	0	0	0	1	0	1	0	4
**4.261**	0	0	1	1	0	0	0	0	0	1	1	0	0	1	0	0	0	0	0	1	0	0	0	1	1	4
**4.262**	0	0	1	0	0	0	0	0	1	0	0	0	0	0	0	1	0	0	0	0	0	1	0	0	0	4
**4.263**	0	0	1	0	0	0	0	0	0	1	0	0	1	1	0	0	0	0	0	1	0	0	0	1	1	4
**4.264**	0	0	0	1	1	0	1	0	0	0	0	1	0	0	0	0	0	0	0	1	0	0	0	0	0	4
**4.265**	0	0	0	0	0	0	1	0	0	1	0	1	0	0	0	1	0	0	0	0	0	0	0	1	1	4
**4.266**	0	0	0	0	0	0	1	0	0	0	0	0	0	1	0	0	0	0	0	1	0	0	0	0	1	4
**4.267**	0	0	0	0	0	0	0	0	1	1	1	1	0	0	0	0	0	0	0	0	0	0	0	1	1	4
**4.268**	0	0	0	0	0	0	0	0	0	0	1	1	0	1	0	0	0	0	0	0	0	0	1	0	0	4
**4.269**	0	0	0	0	0	0	0	0	0	0	1	0	0	1	0	0	0	0	0	1	0	1	0	0	1	4

CMP	CD2	CD3	CD4	CD7	CD8	CD10	CD13	CD18	CD26	CD29	CD36	CD44	CD45RA	CD49f	CD54	CD56	CD57	CD58	CD62L	CD71	CD80	Cytokeratin	Histone_H1	HLA-DQ	HLA-DR	Freq. (voxel)
**4.270**	0	1	0	1	0	0	0	0	1	0	0	1	0	0	0	0	0	0	0	0	0	0	0	0	1	4
**4.271**	0	1	0	0	1	0	0	0	0	0	1	1	0	0	0	0	0	0	0	0	0	0	0	0	1	4
**4.272**	0	1	0	0	0	0	0	0	0	0	0	1	1	1	0	0	0	0	0	0	0	0	0	0	0	4
**4.273**	0	0	0	1	0	0	0	0	1	0	0	0	1	0	0	0	0	0	0	1	0	1	0	0	0	4
**4.274**	0	0	0	0	1	0	1	0	0	1	0	1	0	0	0	0	0	0	0	0	0	1	0	0	1	4
**4.275**	0	0	0	0	1	0	1	0	0	1	0	1	0	0	0	0	0	0	0	0	0	0	1	0	0	4
**4.276**	0	0	0	0	1	0	1	0	0	0	0	0	1	0	0	0	0	0	0	1	0	1	0	0	0	4
**4.277**	0	0	0	0	1	0	0	0	0	0	0	0	1	0	0	0	0	0	0	0	0	0	1	0	1	4
**4.278**	0	0	1	1	0	0	0	0	0	1	0	1	0	0	0	0	0	0	0	0	0	0	0	1	0	4
**4.279**	0	0	0	1	0	0	1	0	0	0	0	0	0	0	0	0	0	0	0	0	0	0	0	0	0	4
**4.280**	0	0	0	0	1	0	0	0	0	1	1	1	0	0	0	0	0	0	0	0	0	0	0	0	1	4
**4.281**	0	0	0	1	0	0	0	0	1	0	0	0	1	0	0	0	0	0	0	0	0	1	0	0	0	4
**4.282**	0	0	0	0	0	0	0	0	0	1	1	0	0	0	0	0	0	0	0	1	0	0	0	1	0	4
**4.283**	0	1	0	0	1	0	0	0	0	1	0	0	0	0	0	0	0	0	0	0	0	1	1	0	0	4
**4.284**	0	0	0	0	0	0	0	0	1	0	0	0	1	0	0	0	0	0	0	1	0	1	0	0	1	4
**4.285**	0	1	0	1	0	0	0	0	1	0	0	0	0	0	0	0	0	0	0	0	0	0	0	0	1	4
**4.286**	0	0	0	0	1	0	0	0	1	0	0	0	1	0	0	0	0	0	0	0	0	0	0	0	0	4
**4.287**	0	0	0	0	0	0	1	0	0	0	0	0	0	0	0	0	0	0	0	1	0	0	1	0	1	4
**4.288**	0	1	1	1	1	0	1	0	1	1	0	1	1	0	0	0	0	0	0	1	0	0	0	1	0	3
**4.289**	0	1	1	1	1	0	1	0	1	1	0	1	0	0	0	0	0	0	0	1	0	1	0	1	1	3
**4.290**	0	1	1	1	1	0	1	0	1	0	0	1	0	0	0	0	0	0	0	1	0	1	0	1	1	3
**4.291**	0	1	1	0	1	0	1	0	1	1	0	1	1	0	0	0	0	0	0	1	0	0	1	1	0	3
**4.292**	0	1	1	0	1	0	1	0	1	0	0	1	1	1	0	0	0	0	0	1	0	1	0	1	1	3
**4.293**	0	1	1	0	1	0	0	0	1	0	0	1	0	0	0	0	0	0	0	1	0	0	1	0	0	3
**4.294**	0	1	0	1	1	0	0	0	1	0	0	1	1	0	0	0	0	0	0	0	0	1	0	1	0	3
**4.295**	0	1	0	1	1	0	0	0	1	0	0	0	0	0	0	0	0	0	0	0	0	0	1	1	0	3
**4.296**	0	1	0	0	1	0	1	0	1	0	0	1	0	0	0	0	0	0	0	1	0	1	0	1	1	3
**4.297**	0	1	0	0	0	0	0	0	1	0	0	1	1	0	0	0	0	0	0	1	0	0	1	1	1	3
**4.298**	0	0	1	1	0	0	0	0	0	1	0	0	0	0	0	0	0	0	0	1	0	0	0	0	0	3
**4.299**	0	0	1	0	0	0	1	0	1	0	0	0	0	0	0	0	0	0	0	1	0	1	0	1	1	3
**4.300**	0	0	0	1	1	0	1	0	1	0	0	1	0	0	0	0	0	0	0	0	0	0	0	0	1	3
**4.301**	0	0	0	1	1	0	0	0	1	0	0	1	1	1	0	0	0	0	0	0	0	0	0	1	1	3
**4.302**	0	0	0	1	1	0	0	0	0	0	0	1	0	0	0	0	0	0	0	0	0	1	1	1	0	3
**4.303**	0	0	0	1	1	0	0	0	0	0	0	0	1	0	0	0	0	0	0	1	0	0	0	1	1	3
**4.304**	0	0	0	1	0	0	1	0	1	1	0	1	0	0	0	0	0	0	0	0	0	0	0	0	0	3
**4.305**	0	0	0	1	0	0	1	0	0	0	0	0	1	0	0	1	0	0	0	1	0	0	0	1	0	3
**4.306**	0	0	0	1	0	0	0	0	1	1	0	1	1	1	0	0	0	0	0	1	0	0	0	1	1	3
**4.307**	0	0	0	0	1	0	1	0	1	1	0	1	0	0	0	0	0	0	0	1	0	0	1	0	0	3
**4.308**	0	0	0	0	1	0	1	0	0	0	0	1	0	0	0	0	0	0	0	0	0	1	0	0	1	3
**4.309**	0	0	0	0	1	0	0	0	1	1	0	1	1	0	0	0	0	0	0	1	0	0	0	1	1	3
**4.310**	0	0	0	0	1	0	0	0	1	0	0	1	1	1	0	0	0	0	0	0	0	0	0	1	1	3
**4.311**	0	0	0	0	1	0	0	0	1	0	0	1	1	0	0	0	0	0	0	1	0	0	1	1	1	3
**4.312**	0	0	0	0	0	0	1	0	1	1	0	1	0	0	0	0	0	0	0	1	0	1	0	1	1	3
**4.313**	0	0	0	0	0	0	0	0	1	1	0	1	0	0	0	1	0	0	0	0	0	1	0	0	0	3
**4.314**	0	1	1	1	1	0	1	0	1	1	0	1	1	0	0	0	0	0	0	1	0	0	0	1	1	3
**4.315**	0	1	1	1	1	0	0	0	1	1	0	1	0	1	0	0	0	0	0	1	0	0	0	0	1	3
**4.316**	0	1	1	0	1	0	1	0	1	1	0	1	1	0	0	0	0	0	0	1	0	0	0	1	0	3
**4.317**	0	1	1	0	1	0	0	0	1	0	0	1	0	1	0	0	0	0	0	1	0	1	0	1	1	3
**4.318**	0	1	1	0	1	0	0	0	1	0	0	1	0	0	0	0	0	0	0	0	0	1	0	1	1	3
**4.319**	0	1	0	1	1	0	1	0	1	0	0	1	1	0	0	0	0	0	0	1	0	1	0	1	1	3
**4.320**	0	1	0	1	1	0	1	0	1	0	0	1	0	0	0	0	0	0	0	0	0	0	1	1	0	3
**4.321**	0	1	0	1	1	0	0	0	1	1	0	1	1	1	0	0	0	0	0	0	0	0	0	1	0	3
**4.322**	0	1	0	1	0	0	0	0	0	0	0	0	0	1	0	0	0	0	0	1	0	1	1	0	0	3
**4.323**	0	1	0	0	1	0	1	0	1	0	0	1	0	0	0	0	0	0	0	1	0	0	0	0	1	3
**4.324**	0	1	0	0	1	0	1	0	0	1	0	1	0	0	0	0	0	0	0	0	0	0	0	1	0	3
**4.325**	0	1	0	0	1	0	0	0	1	1	0	1	1	0	0	0	0	0	0	0	0	0	1	1	0	3
**4.326**	0	1	0	0	1	0	0	0	1	1	0	0	0	0	0	0	0	0	0	1	0	1	0	1	1	3
**4.327**	0	1	0	0	1	0	0	0	1	0	0	1	0	0	0	0	0	0	0	1	0	0	1	1	0	3
**4.328**	0	1	0	0	1	0	0	0	0	1	0	1	0	1	0	0	0	0	0	1	0	1	0	1	0	3
**4.329**	0	1	0	0	0	0	1	0	1	0	0	1	0	0	0	0	0	0	0	1	0	0	0	1	1	3
**4.330**	0	1	0	0	0	0	0	0	1	1	0	1	0	0	0	0	0	0	0	0	0	1	1	0	1	3
**4.331**	0	1	0	0	0	0	0	0	1	0	0	1	1	1	0	0	0	0	0	1	0	1	1	1	1	3
**4.332**	0	1	0	0	0	0	0	0	1	0	0	1	0	1	0	0	0	0	0	0	0	1	1	1	1	3
**4.333**	0	1	0	0	0	0	0	0	0	1	0	0	0	1	0	0	0	0	0	1	0	0	0	0	0	3
**4.334**	0	0	1	1	1	0	1	0	0	0	0	1	0	0	0	0	0	0	0	1	0	0	0	1	1	3
**4.335**	0	0	1	1	0	0	0	0	1	0	0	1	0	0	0	0	0	0	0	1	0	0	0	1	1	3
**4.336**	0	0	1	1	0	0	0	0	0	0	0	0	1	0	0	1	0	0	0	1	0	0	0	0	1	3
**4.337**	0	0	1	1	0	0	0	0	0	0	0	0	1	0	0	0	0	0	0	1	0	0	0	0	1	3
**4.338**	0	0	1	0	1	0	0	0	1	0	0	1	0	0	0	0	0	0	0	1	0	0	0	0	0	3
**4.339**	0	0	1	0	0	0	0	0	1	1	0	1	0	0	0	0	0	0	0	0	0	0	1	0	0	3
**4.340**	0	0	1	0	0	0	0	0	1	0	0	0	0	1	0	0	0	0	0	1	0	0	0	1	1	3
**4.341**	0	0	0	1	1	0	0	0	1	1	0	0	0	0	0	0	0	0	0	0	0	0	1	0	0	3
**4.342**	0	0	0	1	1	0	0	0	0	1	0	1	1	0	0	0	0	0	0	1	0	1	1	0	0	3
**4.343**	0	0	0	1	1	0	0	0	0	1	0	1	0	1	0	0	0	0	0	1	0	0	1	0	0	3
**4.344**	0	0	0	1	1	0	0	0	0	0	0	1	0	0	0	0	0	0	0	1	0	1	0	1	1	3
**4.345**	0	0	0	1	1	0	0	0	0	0	0	0	0	0	0	0	0	0	0	0	0	0	1	1	1	3
**4.346**	0	0	0	1	0	0	0	0	1	0	0	1	0	0	0	0	0	0	0	1	0	1	0	1	1	3
**4.347**	0	0	0	1	0	0	0	0	1	0	0	0	0	0	0	0	0	0	0	1	0	1	0	0	1	3
**4.348**	0	0	0	1	0	0	0	0	1	0	0	0	0	0	0	0	0	0	0	0	0	1	0	1	0	3
**4.349**	0	0	0	1	0	0	0	0	0	1	0	1	1	1	0	0	0	0	0	1	0	0	0	1	0	3
**4.350**	0	0	0	1	0	0	0	0	0	1	0	0	0	0	0	0	0	0	0	1	0	0	0	1	0	3
**4.351**	0	0	0	1	0	0	0	0	0	1	0	0	0	0	0	0	0	0	0	0	0	1	0	1	1	3
**4.352**	0	0	0	0	1	0	0	0	1	1	0	1	1	0	0	0	0	0	0	0	0	1	0	1	1	3
**4.353**	0	0	0	0	1	0	0	0	1	1	0	1	0	1	0	0	0	0	0	0	0	0	0	1	1	3
**4.354**	0	0	0	0	1	0	0	0	1	1	0	1	0	0	0	0	0	0	0	1	0	1	0	1	0	3
**4.355**	0	0	0	0	1	0	0	0	1	1	0	1	0	0	0	0	0	0	0	1	0	0	1	1	0	3
**4.356**	0	0	0	0	1	0	0	0	1	0	0	1	0	1	0	0	0	0	0	0	0	0	0	1	1	3
**4.357**	0	0	0	0	1	0	0	0	0	1	0	1	1	0	0	0	0	0	0	1	0	1	0	0	1	3
**4.358**	0	0	0	0	1	0	0	0	0	1	0	0	1	0	0	0	0	0	0	1	0	1	1	0	0	3
**4.359**	0	0	0	0	0	0	1	0	1	0	0	0	0	0	0	1	0	0	0	0	0	0	0	0	0	3
**4.360**	0	0	0	0	0	0	1	0	0	1	0	1	0	0	0	0	0	0	0	1	0	1	0	1	0	3
**4.361**	0	0	0	0	0	0	1	0	0	0	0	1	0	0	0	1	0	0	0	0	0	1	0	1	0	3
**4.362**	0	0	0	0	0	0	1	0	0	0	0	0	0	0	0	1	0	0	0	0	0	1	0	1	0	3
**4.363**	0	0	0	0	0	0	1	0	0	0	0	0	0	0	0	0	0	0	0	0	0	1	1	1	1	3
**4.364**	0	0	0	0	0	0	0	0	1	1	0	0	0	1	0	0	0	0	0	1	0	0	0	0	0	3
**4.365**	0	0	0	0	0	0	0	0	1	0	0	1	0	0	0	1	0	0	0	0	0	0	0	1	0	3
**4.366**	0	0	0	0	0	0	0	0	1	0	0	0	0	0	0	0	0	0	0	1	0	1	1	1	1	3
**4.367**	0	1	1	1	1	0	0	0	1	1	0	1	1	0	0	1	0	0	0	1	0	0	0	0	0	3
**4.368**	0	1	1	0	1	0	1	0	1	1	0	1	1	0	0	0	0	0	0	1	0	0	0	1	1	3
**4.369**	0	1	1	0	1	0	0	0	1	0	0	1	1	0	0	0	0	0	0	0	0	1	0	1	1	3
**4.370**	0	1	1	0	1	0	0	0	0	1	0	1	0	1	0	0	0	0	0	1	0	0	1	0	0	3
**4.371**	0	1	0	1	1	0	1	0	1	1	0	1	0	0	0	0	0	0	0	1	0	0	0	0	0	3
**4.372**	0	1	0	1	1	0	1	0	0	1	0	1	1	0	0	0	0	0	0	1	0	1	0	1	0	3
**4.373**	0	1	0	1	1	0	0	0	0	1	0	1	1	1	0	0	0	0	0	1	0	1	0	1	1	3
**4.374**	0	1	0	1	0	0	1	0	1	0	0	1	0	0	0	0	0	0	0	0	0	0	0	1	1	3
**4.375**	0	1	0	1	0	0	1	0	0	1	0	1	0	1	0	0	0	0	0	1	0	1	0	0	0	3
**4.376**	0	1	0	1	0	0	0	0	0	1	0	1	1	0	0	0	0	0	0	1	0	0	0	1	1	3
**4.377**	0	1	0	1	0	0	0	0	0	0	0	1	1	0	0	0	0	0	0	0	0	0	0	1	0	3
**4.378**	0	1	0	0	1	0	1	0	1	0	0	1	0	0	0	0	0	0	0	1	0	0	1	1	1	3
**4.379**	0	1	0	0	1	0	1	0	0	0	0	1	0	0	0	0	0	0	0	1	0	0	1	1	1	3
**4.380**	0	1	0	0	1	0	0	0	0	0	0	1	1	0	0	0	0	0	0	1	0	1	1	0	1	3
**4.381**	0	1	0	0	0	0	0	0	1	0	0	1	1	0	0	0	0	0	0	1	0	1	1	0	0	3
**4.382**	0	0	1	1	1	0	0	0	0	0	0	1	0	0	0	0	0	0	0	0	0	0	0	0	1	3
**4.383**	0	0	1	1	0	0	0	0	1	0	0	1	0	0	0	0	0	0	0	1	0	1	0	1	1	3
**4.384**	0	0	1	0	1	0	1	0	0	1	0	1	0	0	0	0	0	0	0	1	0	0	0	1	1	3
**4.385**	0	0	1	0	1	0	0	0	1	0	0	1	0	0	0	0	0	0	0	0	0	0	1	1	1	3
**4.386**	0	0	0	1	1	0	1	0	0	0	0	0	0	0	0	0	0	0	0	0	0	0	1	1	0	3
**4.387**	0	0	0	1	1	0	0	0	1	0	0	1	0	0	0	0	0	0	0	1	0	1	1	0	1	3
**4.388**	0	0	0	1	0	0	1	0	1	1	0	1	0	0	0	0	0	0	0	0	0	0	0	1	1	3
**4.389**	0	0	0	1	0	0	0	0	1	1	0	1	1	0	0	0	0	0	0	1	0	1	1	1	0	3
**4.390**	0	0	0	1	0	0	0	0	1	1	0	1	1	0	0	0	0	0	0	1	0	0	0	0	0	3
**4.391**	0	0	0	1	0	0	0	0	1	0	0	1	1	0	0	0	0	0	0	1	0	0	0	1	1	3

CMP	CD2	CD3	CD4	CD7	CD8	CD10	CD13	CD18	CD26	CD29	CD36	CD44	CD45RA	CD49f	CD54	CD56	CD57	CD58	CD62L	CD71	CD80	Cytokeratin	Histone_H1	HLA-DQ	HLA-DR	Freq. (voxel)
**4.392**	0	0	0	1	0	0	0	0	1	0	0	1	0	0	0	0	0	0	0	1	0	1	0	1	0	3
**4.393**	0	0	0	1	0	0	0	0	1	0	0	0	1	0	0	0	0	0	0	1	0	0	0	1	1	3
**4.394**	0	0	0	1	0	0	0	0	0	1	0	1	1	0	0	0	0	0	0	0	0	0	0	1	0	3
**4.395**	0	0	0	1	0	0	0	0	0	0	0	1	1	0	0	0	0	0	0	0	0	0	0	1	0	3
**4.396**	0	0	0	1	0	0	0	0	0	0	0	1	0	0	0	0	0	0	0	0	0	1	1	1	1	3
**4.397**	0	0	0	1	0	0	0	0	0	0	0	0	1	1	0	0	0	0	0	1	0	0	0	0	0	3
**4.398**	0	0	0	1	0	0	0	0	0	0	0	0	1	0	0	0	0	0	0	1	0	0	0	1	1	3
**4.399**	0	0	0	0	1	0	0	0	0	0	0	1	1	0	0	0	0	0	0	0	0	1	1	0	1	3
**4.400**	0	0	0	0	1	0	0	0	0	0	0	1	1	0	0	0	0	0	0	0	0	1	0	1	0	3
**4.401**	0	0	0	0	1	0	0	0	0	0	0	1	0	0	0	0	0	0	0	1	0	1	0	1	0	3
**4.402**	0	0	0	0	1	0	0	0	0	0	0	0	0	0	0	0	0	0	0	1	0	1	0	1	1	3
**4.403**	0	0	0	0	0	0	1	0	1	1	0	1	0	0	0	0	0	0	0	0	0	0	1	1	1	3
**4.404**	0	0	0	0	0	0	1	0	1	0	0	1	0	0	0	0	0	0	0	1	0	0	1	0	0	3
**4.405**	0	0	0	0	0	0	1	0	0	1	0	0	1	1	0	0	0	0	0	1	0	0	0	0	0	3
**4.406**	0	0	0	0	0	0	1	0	0	0	0	1	1	0	0	1	0	0	0	0	0	1	0	1	0	3
**4.407**	0	0	0	0	0	0	0	0	1	0	0	1	1	1	0	0	0	0	0	0	0	1	0	1	1	3
**4.408**	0	0	0	0	0	0	0	0	0	1	0	1	0	0	0	0	0	0	0	1	0	1	1	1	1	3
**4.409**	0	1	1	1	1	0	0	0	1	1	0	1	1	0	0	0	0	0	0	0	0	1	0	0	0	3
**4.410**	0	1	1	1	1	0	0	0	0	0	0	1	1	0	0	0	0	0	0	1	0	1	0	1	1	3
**4.411**	0	1	1	1	0	0	0	0	1	1	0	1	1	0	0	0	0	0	0	1	0	0	0	0	0	3
**4.412**	0	1	1	0	1	0	1	0	1	1	0	1	1	1	0	0	0	0	0	1	0	0	0	0	0	3
**4.413**	0	1	1	0	1	0	0	0	0	1	0	0	0	0	0	0	0	0	0	1	0	0	1	0	0	3
**4.414**	0	1	1	0	0	0	0	0	1	0	0	1	1	0	0	0	0	0	0	1	0	0	0	1	1	3
**4.415**	0	1	1	0	0	0	0	0	1	0	0	1	0	0	0	0	0	0	0	1	0	1	0	1	1	3
**4.416**	0	1	1	0	0	0	0	0	0	1	0	1	1	0	0	0	0	0	0	1	0	0	0	0	0	3
**4.417**	0	1	1	0	0	0	0	0	0	1	0	1	0	0	0	0	0	0	0	0	0	1	1	1	1	3
**4.418**	0	1	0	1	1	0	0	0	1	1	0	1	0	1	0	0	0	0	0	0	0	0	0	0	1	3
**4.419**	0	1	0	1	1	0	0	0	1	1	0	1	0	0	0	0	0	0	0	1	0	1	1	0	1	3
**4.420**	0	1	0	1	1	0	0	0	0	1	0	1	1	0	0	0	0	0	0	1	0	0	1	1	0	3
**4.421**	0	1	0	1	0	0	1	0	1	0	0	1	0	0	0	1	0	0	0	1	0	0	0	1	1	3
**4.422**	0	1	0	1	0	0	1	0	1	0	0	1	0	0	0	0	0	0	0	1	0	0	0	0	0	3
**4.423**	0	1	0	1	0	0	1	0	0	0	0	1	0	1	0	0	0	0	0	1	0	0	0	1	1	3
**4.424**	0	1	0	1	0	0	1	0	0	0	0	1	0	0	0	0	0	0	0	0	0	0	1	0	0	3
**4.425**	0	1	0	1	0	0	0	0	1	0	0	1	1	1	0	0	0	0	0	1	0	1	0	1	1	3
**4.426**	0	1	0	1	0	0	0	0	0	0	0	1	1	1	0	0	0	0	0	1	0	1	0	1	1	3
**4.427**	0	1	0	1	0	0	0	0	0	0	0	0	1	0	0	0	0	0	0	0	0	0	1	1	0	3
**4.428**	0	1	0	0	1	0	1	0	1	1	0	1	1	1	0	0	0	0	0	1	0	1	1	0	0	3
**4.429**	0	1	0	0	1	0	1	0	0	0	0	1	0	1	0	0	0	0	0	0	0	1	0	0	1	3
**4.430**	0	1	0	0	1	0	0	0	1	1	0	1	0	1	0	0	0	0	0	1	0	1	0	1	1	3
**4.431**	0	1	0	0	1	0	0	0	1	1	0	1	0	0	0	0	0	0	0	1	0	1	1	0	1	3
**4.432**	0	1	0	0	0	0	0	0	1	1	0	1	0	1	0	0	0	0	0	1	0	1	0	0	0	3
**4.433**	0	1	0	0	0	0	0	0	1	0	0	0	0	0	0	0	0	0	0	1	0	1	1	1	1	3
**4.434**	0	1	0	0	0	0	0	0	0	1	0	1	1	0	0	0	0	0	0	1	0	1	0	1	0	3
**4.435**	0	1	0	0	0	0	0	0	0	0	0	1	1	0	0	0	0	0	0	1	0	1	1	1	0	3
**4.436**	0	1	0	0	0	0	0	0	0	0	0	0	0	0	0	0	0	0	0	1	0	0	1	1	1	3
**4.437**	0	0	1	1	0	0	0	0	1	1	0	1	0	0	0	0	0	0	0	0	0	0	0	0	0	3
**4.438**	0	0	1	1	0	0	0	0	0	1	0	0	1	0	0	0	0	0	0	1	0	0	0	1	1	3
**4.439**	0	0	1	1	0	0	0	0	0	0	0	1	1	0	0	0	0	0	0	0	0	0	0	1	1	3
**4.440**	0	0	1	0	1	0	1	0	0	1	0	1	0	0	0	0	0	0	0	0	0	0	0	1	1	3
**4.441**	0	0	1	0	1	0	0	0	0	1	0	1	1	0	0	0	0	0	0	1	0	0	0	0	0	3
**4.442**	0	0	1	0	0	0	1	0	0	0	0	0	1	1	0	0	0	0	0	1	0	0	0	0	0	3
**4.443**	0	0	1	0	0	0	0	0	1	1	0	0	0	0	0	0	0	0	0	0	0	1	0	0	0	3
**4.444**	0	0	1	0	0	0	0	0	1	0	0	1	1	0	0	0	0	0	0	1	0	0	0	1	1	3
**4.445**	0	0	1	0	0	0	0	0	1	0	0	0	0	1	0	0	0	0	0	1	0	1	0	1	1	3
**4.446**	0	0	1	0	0	0	0	0	0	0	0	0	0	1	0	0	0	0	0	1	0	1	0	0	1	3
**4.447**	0	0	0	1	0	0	1	0	1	0	0	1	0	0	0	1	0	0	0	1	0	0	0	1	1	3
**4.448**	0	0	0	1	0	0	1	0	0	0	0	0	0	0	0	0	0	0	0	1	0	0	0	0	0	3
**4.449**	0	0	0	1	0	0	0	0	1	0	0	1	0	0	0	1	0	0	0	1	0	0	0	1	1	3
**4.450**	0	0	0	1	0	0	0	0	0	1	0	1	1	1	0	1	0	0	0	1	0	1	1	1	0	3
**4.451**	0	0	0	1	0	0	0	0	0	1	0	1	0	1	0	0	0	0	0	1	0	1	1	1	0	3
**4.452**	0	0	0	1	0	0	0	0	0	1	0	1	0	1	0	0	0	0	0	1	0	0	1	0	0	3
**4.453**	0	0	0	1	0	0	0	0	0	0	0	1	1	0	0	0	0	0	0	1	0	1	0	0	0	3
**4.454**	0	0	0	1	0	0	0	0	0	0	0	1	1	0	0	0	0	0	0	0	0	0	0	1	1	3
**4.455**	0	0	0	1	0	0	0	0	0	0	0	1	0	1	0	0	0	0	0	1	0	0	1	0	0	3
**4.456**	0	0	0	1	0	0	0	0	0	0	0	1	0	0	0	1	0	0	0	1	0	0	0	1	1	3
**4.457**	0	0	0	0	1	0	0	0	1	1	0	1	1	1	0	0	0	0	0	1	0	0	0	0	0	3
**4.458**	0	0	0	0	1	0	0	0	0	1	0	1	0	1	0	0	0	0	0	1	0	0	1	0	0	3
**4.459**	0	0	0	0	1	0	0	0	0	0	0	1	0	1	0	0	0	0	0	0	0	1	0	1	1	3
**4.460**	0	0	0	0	1	0	0	0	0	0	0	0	1	1	0	0	0	0	0	0	0	0	0	0	0	3
**4.461**	0	0	0	0	0	0	1	0	0	0	0	0	0	1	0	0	0	0	0	1	0	0	1	1	1	3
**4.462**	0	0	0	0	0	0	0	0	0	1	0	0	1	1	0	1	0	0	0	0	0	0	1	1	0	3
**4.463**	0	0	0	0	0	0	0	0	0	0	0	1	1	0	0	1	0	0	0	0	0	1	0	1	0	3
**4.464**	0	1	1	1	1	0	0	0	1	1	0	1	1	1	0	1	0	0	0	1	0	0	0	0	0	3
**4.465**	0	1	1	1	0	0	0	0	0	1	0	1	0	1	0	0	0	0	0	1	0	0	0	1	1	3
**4.466**	0	1	1	1	0	0	0	0	0	1	0	1	0	0	0	0	0	0	0	1	0	0	0	0	1	3
**4.467**	0	1	1	1	0	0	0	0	0	0	0	1	1	1	0	0	0	0	0	1	0	0	0	0	0	3
**4.468**	0	1	1	1	0	0	0	0	0	0	0	1	0	0	0	0	0	0	0	1	0	0	0	0	1	3
**4.469**	0	1	1	0	1	0	0	0	1	0	0	0	0	0	0	0	0	0	0	1	0	0	0	1	1	3
**4.470**	0	1	1	0	1	0	0	0	0	1	0	1	1	1	0	0	0	0	0	1	0	0	0	0	0	3
**4.471**	0	1	1	0	0	0	1	0	1	0	0	1	1	0	0	0	0	0	0	1	0	0	1	0	0	3
**4.472**	0	1	1	0	0	0	1	0	1	0	0	1	0	0	0	0	0	0	0	1	0	0	1	0	0	3
**4.473**	0	1	1	0	0	0	0	0	1	0	0	1	0	0	0	1	0	0	0	1	0	0	0	1	1	3
**4.474**	0	1	1	0	0	0	0	0	0	0	0	0	0	0	0	0	0	0	0	0	0	1	1	1	1	3
**4.475**	0	1	0	1	1	0	0	0	0	1	0	1	1	0	0	0	0	0	0	1	0	0	1	1	1	3
**4.476**	0	1	0	1	1	0	0	0	0	1	0	1	0	0	0	0	0	0	0	1	0	1	1	0	1	3
**4.477**	0	1	0	1	1	0	0	0	0	0	0	1	1	1	0	0	0	0	0	1	0	1	0	1	1	3
**4.478**	0	1	0	1	1	0	0	0	0	0	0	1	1	0	0	0	0	0	0	0	0	1	1	1	1	3
**4.479**	0	1	0	1	0	0	1	0	0	1	0	1	0	0	0	0	0	0	0	0	0	0	0	1	1	3
**4.480**	0	1	0	1	0	0	0	0	0	1	0	1	0	1	0	0	0	0	0	1	0	1	0	1	1	3
**4.481**	0	1	0	1	0	0	0	0	0	0	1	1	0	1	0	0	0	0	0	1	0	1	0	1	1	3
**4.482**	0	1	0	1	0	0	0	0	0	0	0	0	0	1	0	0	0	0	0	1	0	1	0	1	1	3
**4.483**	0	1	0	0	1	0	0	0	1	1	0	1	0	0	0	0	0	0	0	0	0	1	1	0	1	3
**4.484**	0	1	0	0	1	0	0	0	1	0	0	1	1	0	0	0	0	0	0	1	0	1	1	1	0	3
**4.485**	0	1	0	0	1	0	0	0	0	1	0	1	1	0	0	1	0	0	0	0	0	0	0	0	0	3
**4.486**	0	1	0	0	0	0	0	0	1	0	0	0	0	0	0	0	0	0	0	1	0	1	1	0	1	3
**4.487**	0	1	0	0	0	0	0	0	0	1	0	1	1	0	0	0	0	0	0	0	0	1	1	1	0	3
**4.488**	0	1	0	0	0	0	0	0	0	1	0	0	0	0	0	0	0	0	0	0	0	0	0	1	1	3
**4.489**	0	0	1	0	1	0	1	0	0	0	0	1	0	0	0	0	0	0	0	1	0	0	0	1	1	3
**4.490**	0	0	1	0	1	0	0	0	0	1	0	1	0	0	0	0	0	0	0	0	0	1	0	0	0	3
**4.491**	0	0	1	0	1	0	0	0	0	1	0	0	0	0	0	0	0	0	0	1	0	0	0	1	1	3
**4.492**	0	0	1	0	0	0	1	0	0	0	0	1	0	0	0	0	0	0	0	0	0	1	0	0	0	3
**4.493**	0	0	1	0	0	0	0	0	1	1	0	1	1	0	0	0	0	0	0	1	0	0	0	0	0	3
**4.494**	0	0	1	0	0	0	0	0	1	0	0	1	0	1	0	0	0	0	0	0	0	0	0	0	0	3
**4.495**	0	0	1	0	0	0	0	0	0	1	0	0	1	1	0	0	0	0	0	1	0	0	0	1	0	3
**4.496**	0	0	1	0	0	0	0	0	0	1	0	0	1	0	0	0	0	0	0	1	0	1	0	0	0	3
**4.497**	0	0	1	0	0	0	0	0	0	1	0	0	1	0	0	0	0	0	0	0	0	0	0	1	0	3
**4.498**	0	0	0	1	0	0	0	0	0	1	0	1	0	0	0	0	0	0	0	0	0	1	1	0	0	3
**4.499**	0	0	0	1	0	0	0	0	0	1	0	1	0	0	0	0	0	0	0	0	0	1	0	1	1	3
**4.500**	0	0	0	1	0	0	0	0	0	0	0	0	0	1	0	0	0	0	0	1	0	0	1	0	0	3
**4.501**	0	0	0	0	1	0	1	0	0	1	0	1	0	1	0	0	0	0	0	1	0	0	0	1	0	3
**4.502**	0	0	0	0	0	0	1	0	1	1	0	1	0	0	0	0	0	0	0	0	0	0	0	1	0	3
**4.503**	0	0	0	0	0	0	0	0	0	1	0	0	1	1	0	0	0	0	0	1	0	0	0	1	0	3
**4.504**	0	0	0	0	0	0	0	0	0	1	0	0	1	0	0	1	0	0	0	0	0	1	0	0	0	3
**4.505**	0	0	0	0	0	0	0	0	0	0	1	1	0	1	0	0	0	0	0	1	0	1	0	0	1	3
**4.506**	0	0	0	0	0	0	0	0	0	0	0	0	1	1	0	0	0	0	0	1	0	0	0	1	1	3
**4.507**	0	0	0	0	0	0	0	0	0	0	0	0	1	0	0	1	0	0	0	0	0	1	0	1	1	3
**4.508**	0	1	1	1	0	0	1	0	0	0	0	1	0	0	0	0	0	0	0	1	0	0	1	0	0	3
**4.509**	0	1	1	1	0	0	0	0	1	0	0	1	0	0	0	0	0	0	0	1	0	0	0	1	1	3
**4.510**	0	1	1	0	1	0	0	0	0	1	0	1	1	1	0	0	0	0	0	1	0	0	0	1	1	3
**4.511**	0	1	1	0	1	0	0	0	0	1	0	1	1	0	0	0	0	0	0	0	0	0	0	0	0	3
**4.512**	0	1	1	0	1	0	0	0	0	0	0	1	1	0	0	0	0	0	0	1	0	0	0	0	0	3
**4.513**	0	1	1	0	1	0	0	0	0	0	0	0	1	0	0	0	0	0	0	1	0	0	0	0	1	3

CMP	CD2	CD3	CD4	CD7	CD8	CD10	CD13	CD18	CD26	CD29	CD36	CD44	CD45RA	CD49f	CD54	CD56	CD57	CD58	CD62L	CD71	CD80	Cytokeratin	Histone_H1	HLA-DQ	HLA-DR	Freq. (voxel)
**4.514**	0	1	1	0	1	0	0	0	0	0	0	0	0	0	0	0	0	0	0	1	0	0	1	0	1	3
**4.515**	0	1	1	0	0	0	1	0	0	1	0	1	0	0	0	0	0	0	0	0	0	0	1	0	0	3
**4.516**	0	1	1	0	0	0	0	0	1	1	0	1	1	1	0	0	0	0	0	1	0	1	0	0	0	3
**4.517**	0	1	1	0	0	0	0	0	0	0	0	1	0	1	0	0	0	0	0	1	0	0	1	0	0	3
**4.518**	0	1	1	0	0	0	0	0	0	0	0	1	0	1	0	0	0	0	0	1	0	0	0	0	1	3
**4.519**	0	1	1	0	0	0	0	0	0	0	0	1	0	0	0	0	0	0	0	1	0	1	0	0	1	3
**4.520**	0	1	1	0	0	0	0	0	0	0	0	1	0	0	0	0	0	0	0	0	0	0	0	0	1	3
**4.521**	0	1	0	1	1	0	1	0	0	1	0	1	0	0	0	0	0	0	0	0	0	0	0	0	1	3
**4.522**	0	1	0	1	1	0	0	0	0	1	0	1	1	1	0	1	0	0	0	1	0	0	0	0	0	3
**4.523**	0	1	0	1	1	0	0	0	0	1	0	1	0	1	0	0	0	0	0	0	0	1	1	0	1	3
**4.524**	0	1	0	1	1	0	0	0	0	1	0	0	1	0	0	0	0	0	0	0	0	0	1	1	1	3
**4.525**	0	1	0	1	1	0	0	0	0	0	0	0	1	0	0	0	0	0	0	0	0	0	1	1	1	3
**4.526**	0	1	0	1	0	0	1	0	0	1	0	1	0	1	0	0	0	0	0	0	0	0	0	1	1	3
**4.527**	0	1	0	1	0	0	1	0	0	0	0	1	0	0	0	0	0	0	0	1	0	0	0	0	1	3
**4.528**	0	1	0	1	0	0	0	0	0	1	0	1	0	0	0	0	0	0	0	0	0	1	0	0	1	3
**4.529**	0	1	0	1	0	0	0	0	0	0	0	0	1	1	0	0	0	0	0	0	0	1	0	1	0	3
**4.530**	0	1	0	1	0	0	0	0	0	0	0	0	0	0	0	0	0	0	0	1	0	0	1	1	1	3
**4.531**	0	1	0	0	1	0	0	0	0	1	0	1	0	0	0	0	0	0	0	1	0	1	1	0	1	3
**4.532**	0	1	0	0	1	0	0	0	0	0	0	0	0	1	0	0	0	0	0	0	0	0	1	0	0	3
**4.533**	0	1	0	0	0	0	0	0	1	1	0	1	1	1	0	0	0	0	0	1	0	1	0	0	0	3
**4.534**	0	1	0	0	0	0	0	0	1	0	0	1	1	0	0	0	0	0	0	1	0	0	1	0	1	3
**4.535**	0	1	0	0	0	0	0	0	0	1	0	0	0	0	0	0	0	0	0	0	0	0	0	0	1	3
**4.536**	0	0	1	1	0	0	0	0	0	0	0	0	0	1	0	0	0	0	0	1	0	1	0	0	0	3
**4.537**	0	0	1	0	1	0	0	0	0	0	0	1	1	0	0	0	0	0	0	1	0	1	0	0	1	3
**4.538**	0	0	1	0	0	0	1	0	1	0	0	1	0	1	0	0	0	0	0	1	0	1	0	1	0	3
**4.539**	0	0	1	0	0	0	1	0	0	0	0	0	1	1	0	1	0	0	0	1	0	1	0	0	0	3
**4.540**	0	0	1	0	0	0	1	0	0	0	0	0	0	1	0	0	0	0	0	1	0	0	0	0	1	3
**4.541**	0	0	1	0	0	0	0	0	1	0	0	1	0	1	0	0	0	0	0	0	0	1	0	1	1	3
**4.542**	0	0	1	0	0	0	0	0	0	1	1	1	0	0	0	0	0	0	0	1	0	0	0	0	1	3
**4.543**	0	0	0	1	0	0	1	0	1	0	0	0	0	1	0	0	0	0	0	1	0	1	0	0	0	3
**4.544**	0	0	0	0	1	0	0	0	1	0	0	0	0	0	0	0	0	0	0	1	0	0	0	1	1	3
**4.545**	0	0	0	0	0	0	1	0	1	1	0	0	0	0	0	0	0	0	0	0	0	1	0	0	0	3
**4.546**	0	0	0	0	0	0	0	0	1	0	0	0	0	0	0	1	0	0	0	1	0	1	0	0	1	3
**4.547**	0	1	1	1	1	0	0	0	0	1	0	1	0	0	0	0	0	0	0	0	0	0	0	0	1	3
**4.548**	0	1	1	1	0	0	0	0	1	0	0	1	1	0	0	0	0	0	0	1	0	0	0	1	0	3
**4.549**	0	1	1	1	0	0	0	0	1	0	0	1	0	0	0	0	0	0	0	1	0	1	0	0	1	3
**4.550**	0	1	1	1	0	0	0	0	1	0	0	1	0	0	0	0	0	0	0	1	0	1	0	0	0	3
**4.551**	0	1	1	0	1	0	0	0	1	1	0	1	1	1	0	0	0	0	0	1	0	0	0	1	1	3
**4.552**	0	1	1	0	1	0	0	0	0	1	0	1	1	1	0	0	0	0	0	0	0	0	0	1	0	3
**4.553**	0	1	1	0	1	0	0	0	0	1	0	1	1	0	0	0	0	0	0	1	0	0	0	0	1	3
**4.554**	0	1	1	0	1	0	0	0	0	1	0	1	0	1	0	0	0	0	0	1	0	0	0	1	0	3
**4.555**	0	1	1	0	1	0	0	0	0	0	0	1	1	0	0	0	0	0	0	1	0	0	0	0	1	3
**4.556**	0	1	1	0	1	0	0	0	0	0	0	0	0	0	0	0	0	0	0	0	0	0	1	0	1	3
**4.557**	0	1	0	1	1	0	0	0	0	1	1	1	1	1	0	0	0	0	0	0	0	1	0	1	1	3
**4.558**	0	1	0	1	1	0	0	0	0	0	0	1	0	0	0	0	0	0	0	0	0	1	1	1	0	3
**4.559**	0	1	0	1	0	0	0	0	0	0	0	0	1	1	0	0	0	0	0	0	0	0	0	1	1	3
**4.560**	0	1	0	1	0	0	0	0	0	0	0	0	0	0	0	0	0	0	0	1	0	1	0	1	1	3
**4.561**	0	1	0	0	0	0	0	0	0	1	0	1	1	0	0	0	0	0	0	0	0	0	0	1	1	3
**4.562**	0	1	0	0	0	0	0	0	0	0	0	0	1	0	0	0	0	0	0	0	0	1	1	0	1	3
**4.563**	0	0	1	1	0	0	1	0	0	1	0	1	0	1	0	0	0	0	0	1	0	1	0	1	0	3
**4.564**	0	0	1	1	0	0	0	0	0	0	0	0	1	1	0	1	0	0	0	0	0	0	0	1	0	3
**4.565**	0	0	1	1	0	0	0	0	0	0	0	0	1	1	0	1	0	0	0	0	0	0	0	0	0	3
**4.566**	0	0	1	0	0	0	1	0	1	1	0	0	0	1	0	0	0	0	0	0	0	0	0	1	0	3
**4.567**	0	0	1	0	0	0	1	0	0	0	1	1	0	1	0	0	0	0	0	0	0	0	0	1	1	3
**4.568**	0	0	1	0	0	0	1	0	0	0	0	0	1	0	0	1	0	0	0	1	0	0	0	1	0	3
**4.569**	0	0	1	0	0	0	0	0	1	0	0	1	1	1	0	0	0	0	0	1	0	1	0	0	1	3
**4.570**	0	0	1	0	0	0	0	0	0	1	0	0	0	0	0	0	0	0	0	0	0	0	1	1	1	3
**4.571**	0	0	1	0	0	0	0	0	0	0	1	1	0	1	0	0	0	0	0	1	0	1	0	1	0	3
**4.572**	0	0	1	0	0	0	0	0	0	0	0	1	0	1	0	0	0	0	0	0	0	0	1	1	0	3
**4.573**	0	0	0	1	0	0	1	0	1	1	0	1	0	1	0	0	0	0	0	1	0	1	0	1	1	3
**4.574**	0	0	0	1	0	0	0	0	0	0	0	0	0	1	0	0	0	0	0	0	0	0	1	0	0	3
**4.575**	0	0	0	0	1	0	0	0	0	1	0	0	0	0	0	0	0	0	0	0	0	0	1	1	1	3
**4.576**	0	0	0	0	0	0	1	0	0	0	1	1	0	1	0	0	0	0	0	1	0	1	0	1	1	3
**4.577**	0	0	0	0	0	0	1	0	0	0	1	1	0	1	0	0	0	0	0	1	0	1	0	0	1	3
**4.578**	0	0	0	0	0	0	0	0	0	1	0	0	0	1	0	1	0	0	0	0	0	0	0	0	0	3
**4.579**	0	0	0	0	0	0	0	0	0	0	0	0	1	1	0	0	0	0	0	1	0	0	0	1	0	3
**4.580**	0	1	1	1	1	0	1	0	0	1	0	1	0	0	0	0	0	0	0	0	0	0	0	0	0	3
**4.581**	0	1	1	1	1	0	0	0	1	0	0	1	1	0	0	0	0	0	0	0	0	0	0	1	0	3
**4.582**	0	1	1	1	0	0	0	0	1	1	0	1	0	1	0	0	0	0	0	1	0	0	0	0	0	3
**4.583**	0	1	1	1	0	0	0	0	0	1	0	1	0	1	0	0	0	0	0	1	0	0	0	0	0	3
**4.584**	0	1	1	0	1	0	0	0	1	0	0	1	1	0	0	0	0	0	0	0	0	0	0	1	0	3
**4.585**	0	1	1	0	1	0	0	0	0	1	0	1	1	0	0	0	0	0	0	0	0	0	1	1	0	3
**4.586**	0	1	0	1	1	0	0	0	0	1	0	1	0	0	0	0	0	0	0	0	0	1	1	0	0	3
**4.587**	0	1	0	1	0	0	0	0	0	0	0	1	0	1	0	0	0	0	0	0	0	1	1	0	0	3
**4.588**	0	1	0	1	0	0	0	0	0	0	0	1	0	1	0	0	0	0	0	0	0	0	1	1	1	3
**4.589**	0	1	0	0	1	0	1	0	0	1	1	1	0	1	0	0	0	0	0	0	0	1	0	0	0	3
**4.590**	0	1	0	0	0	0	1	0	0	1	1	1	0	1	0	0	0	0	0	0	0	1	0	0	0	3
**4.591**	0	1	0	0	0	0	0	0	1	0	0	1	1	0	0	0	0	0	0	0	0	0	1	0	1	3
**4.592**	0	1	0	0	0	0	0	0	0	1	0	1	0	1	0	0	0	0	0	1	0	1	0	1	1	3
**4.593**	0	1	0	0	0	0	0	0	0	1	0	1	0	1	0	0	0	0	0	1	0	0	1	1	1	3
**4.594**	0	1	0	0	0	0	0	0	0	0	1	1	0	1	0	0	0	0	0	0	0	1	0	1	1	3
**4.595**	0	0	1	0	1	0	1	0	0	0	0	0	1	0	0	0	0	0	0	0	0	0	0	1	0	3
**4.596**	0	0	1	0	1	0	1	0	0	0	0	0	0	0	0	0	0	0	0	0	0	0	0	1	0	3
**4.597**	0	0	1	0	1	0	0	0	0	0	0	1	1	0	0	0	0	0	0	0	0	0	0	1	0	3
**4.598**	0	0	1	0	0	0	1	0	1	1	0	1	1	1	0	0	0	0	0	1	0	1	0	0	1	3
**4.599**	0	0	1	0	0	0	1	0	1	0	0	1	0	0	0	1	0	0	0	0	0	0	0	1	1	3
**4.600**	0	0	1	0	0	0	1	0	0	0	1	1	0	1	0	0	0	0	0	1	0	1	0	0	1	3
**4.601**	0	0	1	0	0	0	1	0	0	0	0	0	0	0	0	0	0	0	0	0	0	0	1	1	0	3
**4.602**	0	0	1	0	0	0	0	0	1	1	0	1	0	1	0	0	0	0	0	1	0	1	1	0	1	3
**4.603**	0	0	1	0	0	0	0	0	1	1	0	0	0	1	0	0	0	0	0	0	0	0	0	1	0	3
**4.604**	0	0	0	1	1	0	0	0	0	0	0	0	0	1	0	0	0	0	0	0	0	0	0	0	0	3
**4.605**	0	0	0	1	0	0	1	0	1	1	0	1	0	1	0	0	0	0	0	0	0	1	0	1	1	3
**4.606**	0	0	0	1	0	0	1	0	0	0	0	1	0	0	0	0	0	0	0	0	0	0	1	0	0	3
**4.607**	0	0	0	0	1	0	0	0	0	0	0	1	0	1	0	0	0	0	0	1	0	1	0	0	0	3
**4.608**	0	1	1	0	1	0	0	0	1	1	0	1	1	0	0	0	0	0	0	0	0	0	1	1	1	3
**4.609**	0	1	1	0	1	0	0	0	1	1	0	1	0	0	0	0	0	0	0	0	0	0	1	1	1	3
**4.610**	0	1	0	1	1	0	0	0	1	0	0	1	1	1	0	0	0	0	0	0	0	0	0	0	1	3
**4.611**	0	1	0	1	1	0	0	0	1	0	0	1	0	1	0	0	0	0	0	0	0	0	0	0	1	3
**4.612**	0	1	0	1	1	0	0	0	0	1	1	1	1	1	0	0	0	0	0	0	0	1	1	1	1	3
**4.613**	0	1	0	1	1	0	0	0	0	1	1	1	0	1	0	0	0	0	0	0	0	0	0	1	1	3
**4.614**	0	1	0	1	1	0	0	0	0	0	0	1	1	0	0	0	0	0	0	0	0	1	1	0	0	3
**4.615**	0	1	0	1	1	0	0	0	0	0	0	1	0	1	0	0	0	0	0	0	0	1	0	0	1	3
**4.616**	0	1	0	0	1	0	0	0	0	0	0	1	1	0	0	0	0	0	0	1	0	1	1	1	0	3
**4.617**	0	1	0	0	0	0	1	0	0	0	0	0	0	0	0	0	0	0	0	0	0	1	1	1	0	3
**4.618**	0	1	0	0	0	0	0	0	0	0	0	0	1	1	0	0	0	0	0	0	0	0	0	1	0	3
**4.619**	0	0	1	1	0	0	1	0	1	1	1	1	1	1	0	0	0	0	0	1	0	1	0	1	1	3
**4.620**	0	0	1	1	0	0	1	0	1	1	1	1	0	1	0	0	0	0	0	1	0	1	0	1	1	3
**4.621**	0	0	1	1	0	0	1	0	0	1	0	1	0	1	0	0	0	0	0	1	0	0	0	1	0	3
**4.622**	0	0	1	1	0	0	1	0	0	1	0	1	0	1	0	0	0	0	0	0	0	1	0	0	0	3
**4.623**	0	0	1	1	0	0	1	0	0	1	0	1	0	1	0	0	0	0	0	0	0	0	0	0	1	3
**4.624**	0	0	1	1	0	0	0	0	0	0	0	1	0	1	0	0	0	0	0	1	0	1	0	1	1	3
**4.625**	0	0	1	0	0	0	1	0	1	1	0	1	0	1	0	1	0	0	0	1	0	1	0	1	1	3
**4.626**	0	0	1	0	0	0	1	0	1	0	0	0	0	0	0	0	0	0	0	1	0	1	1	0	0	3
**4.627**	0	0	1	0	0	0	1	0	0	0	1	1	0	1	0	0	0	0	0	0	0	1	0	0	0	3
**4.628**	0	0	1	0	0	0	0	0	0	0	1	1	0	0	0	0	0	0	0	0	0	1	0	0	0	3
**4.629**	0	0	0	1	1	0	0	0	1	0	0	1	1	0	0	0	0	0	0	0	0	0	0	1	0	3
**4.630**	0	0	0	1	1	0	0	0	0	0	0	0	0	0	0	0	0	0	0	0	0	1	1	0	0	3
**4.631**	0	0	0	1	0	0	0	0	1	1	0	1	0	1	0	0	0	0	0	0	0	1	0	0	0	3
**4.632**	0	0	0	0	0	0	1	0	1	0	0	0	1	0	0	0	0	0	0	0	0	0	1	0	0	3
**4.633**	0	0	0	0	0	0	1	0	1	0	0	0	0	0	0	1	0	0	0	0	0	0	0	0	1	3
**4.634**	0	0	0	0	0	0	1	0	0	1	0	1	1	0	0	0	0	0	0	0	0	0	0	0	0	3
**4.635**	0	0	0	0	0	0	1	0	0	0	1	1	0	1	0	0	0	0	0	0	0	1	0	0	1	3

CMP	CD2	CD3	CD4	CD7	CD8	CD10	CD13	CD18	CD26	CD29	CD36	CD44	CD45RA	CD49f	CD54	CD56	CD57	CD58	CD62L	CD71	CD80	Cytokeratin	Histone_H1	HLA-DQ	HLA-DR	Freq. (voxel)
**4.636**	0	0	0	0	0	0	0	0	1	1	0	0	0	1	0	0	0	0	0	0	0	1	0	1	1	3
**4.637**	0	0	0	0	0	0	0	0	1	0	1	0	0	0	0	0	0	0	0	0	0	1	0	1	1	3
**4.638**	0	0	0	0	0	0	0	0	1	0	0	0	1	1	0	0	0	0	0	0	0	1	0	0	0	3
**4.639**	0	0	0	0	0	0	0	0	0	1	1	1	0	1	0	0	0	0	0	0	0	1	0	1	0	3
**4.640**	0	0	0	0	0	0	0	0	0	0	0	0	0	0	0	1	0	0	0	0	0	1	0	1	1	3
**4.641**	0	1	1	1	1	0	0	0	1	0	0	1	0	0	0	0	0	0	0	1	0	1	0	0	0	3
**4.642**	0	1	1	0	1	0	0	0	1	1	0	1	1	0	0	0	0	0	0	0	0	0	1	0	1	3
**4.643**	0	1	1	0	1	0	0	0	1	1	0	1	0	0	0	0	0	0	0	0	0	0	0	0	1	3
**4.644**	0	1	1	0	1	0	0	0	1	1	0	0	1	0	0	0	0	0	0	0	0	0	0	1	0	3
**4.645**	0	1	1	0	1	0	0	0	0	1	0	0	0	0	0	0	0	0	0	0	0	0	0	0	0	3
**4.646**	0	1	1	0	1	0	0	0	0	0	0	1	0	1	0	0	0	0	0	0	0	0	0	0	0	3
**4.647**	0	1	1	0	0	0	0	0	1	1	0	1	0	0	0	0	0	0	0	0	0	0	0	0	1	3
**4.648**	0	1	1	0	0	0	0	0	1	0	0	0	0	0	0	0	0	0	0	0	0	0	1	0	0	3
**4.649**	0	1	1	0	0	0	0	0	1	0	0	0	0	0	0	0	0	0	0	0	0	0	0	0	0	3
**4.650**	0	1	0	1	1	0	0	0	1	1	0	1	1	0	0	0	0	0	0	1	0	0	1	0	1	3
**4.651**	0	1	0	1	0	0	0	0	1	1	0	0	0	0	0	0	0	0	0	0	0	0	1	0	0	3
**4.652**	0	1	0	1	0	0	0	0	0	1	0	1	0	0	0	0	0	0	0	0	0	0	1	0	1	3
**4.653**	0	1	0	0	1	0	0	0	0	1	0	1	0	1	0	0	0	0	0	0	0	0	1	1	0	3
**4.654**	0	1	0	0	0	0	1	0	0	1	0	1	0	1	0	0	0	0	0	0	0	1	0	0	0	3
**4.655**	0	1	0	0	0	0	0	0	0	1	0	1	0	1	0	0	0	0	0	0	0	0	1	1	0	3
**4.656**	0	0	1	0	1	0	0	0	1	0	0	1	1	0	0	0	0	0	0	0	0	0	0	1	1	3
**4.657**	0	0	1	0	0	0	1	0	1	1	0	1	1	1	0	1	0	0	0	1	0	0	1	1	1	3
**4.658**	0	0	1	0	0	0	1	0	1	1	0	0	0	1	0	0	0	0	0	0	0	0	0	0	0	3
**4.659**	0	0	1	0	0	0	0	0	1	0	0	0	0	1	0	0	0	0	0	0	0	1	1	0	0	3
**4.660**	0	0	1	0	0	0	0	0	0	1	0	1	0	1	0	1	0	0	0	1	0	1	0	0	1	3
**4.661**	0	0	1	0	0	0	0	0	0	1	0	0	1	1	0	0	0	0	0	0	0	1	0	1	1	3
**4.662**	0	0	1	0	0	0	0	0	0	1	0	0	1	0	0	1	0	0	0	0	0	0	0	1	0	3
**4.663**	0	0	1	0	0	0	0	0	0	0	0	1	0	1	0	1	0	0	0	0	0	1	0	0	1	3
**4.664**	0	0	0	1	0	0	1	0	0	1	0	1	0	1	0	0	0	0	0	0	0	0	0	1	0	3
**4.665**	0	0	0	1	0	0	0	0	0	1	0	0	0	1	0	0	0	0	0	0	0	1	0	0	0	3
**4.666**	0	0	0	0	1	0	0	0	0	1	0	1	0	0	0	0	0	0	0	1	0	0	1	1	1	3
**4.667**	0	0	0	0	0	0	1	0	1	1	1	0	0	1	0	0	0	0	0	1	0	1	0	0	0	3
**4.668**	0	0	0	0	0	0	1	0	1	1	0	1	1	1	0	0	0	0	0	0	0	1	0	0	0	3
**4.669**	0	0	0	0	0	0	1	0	0	1	0	1	1	0	0	0	0	0	0	0	0	0	0	1	1	3
**4.670**	0	0	0	0	0	0	0	0	1	1	0	0	0	0	0	0	0	0	0	1	0	0	0	0	1	3
**4.671**	0	0	0	0	0	0	0	0	1	0	0	1	0	0	0	1	0	0	0	0	0	0	1	1	1	3
**4.672**	0	0	0	0	0	0	0	0	1	0	0	0	0	0	0	1	0	0	0	1	0	0	0	1	1	3
**4.673**	0	0	0	0	0	0	0	0	0	1	0	1	1	1	0	0	0	0	0	1	0	1	0	0	1	3
**4.674**	0	0	0	0	0	0	0	0	0	0	1	0	0	0	0	1	0	0	0	0	0	0	0	1	1	3
**4.675**	0	0	0	0	0	0	0	0	0	0	0	1	0	0	0	1	0	0	0	0	0	0	1	0	1	3
**4.676**	0	1	1	1	1	0	0	0	0	0	0	1	0	0	0	0	0	0	0	0	0	0	0	1	0	3
**4.677**	0	1	1	1	1	0	0	0	0	0	0	0	0	0	0	0	0	0	0	0	0	0	0	0	0	3
**4.678**	0	1	1	1	0	0	0	0	1	0	0	1	1	0	0	0	0	0	0	0	0	0	0	1	0	3
**4.679**	0	1	1	1	0	0	0	0	0	0	0	0	0	0	0	0	0	0	0	0	0	0	0	0	0	3
**4.680**	0	1	1	0	1	0	0	0	1	1	0	0	0	0	0	0	0	0	0	1	0	0	1	1	0	3
**4.681**	0	1	0	0	1	0	0	0	1	0	0	0	0	0	0	0	0	0	0	1	0	0	0	0	1	3
**4.682**	0	1	0	0	1	0	0	0	0	0	0	1	1	1	0	0	0	0	0	0	0	0	1	0	1	3
**4.683**	0	1	0	0	0	0	1	0	0	1	0	1	0	1	0	0	0	0	0	0	0	1	0	0	1	3
**4.684**	0	1	0	0	0	0	0	0	1	0	0	1	0	0	0	1	0	0	0	0	0	0	0	1	0	3
**4.685**	0	0	1	1	0	0	0	0	0	1	0	0	0	1	0	0	0	0	0	0	0	1	0	0	0	3
**4.686**	0	0	1	1	0	0	0	0	0	0	0	0	1	0	0	1	0	0	0	1	0	0	0	0	0	3
**4.687**	0	0	1	0	0	0	1	0	1	1	0	0	0	0	0	0	0	0	0	0	0	0	0	1	0	3
**4.688**	0	0	1	0	0	0	1	0	1	0	0	0	0	0	0	0	0	0	0	1	0	0	0	0	0	3
**4.689**	0	0	1	0	0	0	1	0	0	0	0	1	0	0	0	0	0	0	0	1	0	0	0	0	1	3
**4.690**	0	0	1	0	0	0	0	0	1	1	0	1	0	1	0	0	0	0	0	1	0	1	1	0	0	3
**4.691**	0	0	1	0	0	0	0	0	1	0	0	0	1	0	0	1	0	0	0	1	0	0	0	1	0	3
**4.692**	0	0	1	0	0	0	0	0	0	0	0	0	1	0	0	1	0	0	0	0	0	0	0	0	1	3
**4.693**	0	0	1	0	0	0	0	0	0	0	0	0	0	1	0	0	0	0	0	0	0	1	1	1	0	3
**4.694**	0	0	0	1	1	0	0	0	1	0	0	0	0	0	0	0	0	0	0	1	0	1	0	0	1	3
**4.695**	0	0	0	1	0	0	0	0	0	0	0	0	1	0	0	1	0	0	0	0	0	0	0	1	1	3
**4.696**	0	0	0	0	0	0	1	0	1	1	1	1	0	1	0	0	0	0	0	0	0	0	0	1	1	3
**4.697**	0	0	0	0	0	0	1	0	1	1	0	1	0	1	0	1	0	0	0	0	0	0	1	1	1	3
**4.698**	0	0	0	0	0	0	1	0	0	1	0	1	1	1	0	0	0	0	0	1	0	0	1	1	1	3
**4.699**	0	0	0	0	0	0	1	0	0	0	0	0	1	0	0	0	0	0	0	0	0	0	0	0	1	3
**4.700**	0	0	0	0	0	0	1	0	0	0	0	0	0	0	0	1	0	0	0	0	0	1	0	0	0	3
**4.701**	0	0	0	0	0	0	0	0	1	0	1	0	0	1	0	0	0	0	0	1	0	1	1	0	0	3
**4.702**	0	0	0	0	0	0	0	0	0	0	1	0	0	1	0	0	0	0	0	0	0	0	1	1	0	3
**4.703**	0	1	0	1	1	0	0	0	0	1	0	0	1	0	0	0	0	0	0	0	0	0	1	0	0	3
**4.704**	0	1	0	0	1	0	0	0	0	1	1	1	0	1	0	0	0	0	0	0	0	0	0	1	0	3
**4.705**	0	1	0	0	0	0	0	0	1	0	0	0	0	0	0	0	0	0	0	1	0	0	1	0	1	3
**4.706**	0	1	0	0	0	0	0	0	0	1	1	1	0	1	0	0	0	0	0	0	0	0	0	0	0	3
**4.707**	0	1	0	0	0	0	0	0	0	1	0	0	0	1	0	0	0	0	0	0	0	0	0	0	0	3
**4.708**	0	0	1	1	0	0	0	0	0	0	0	0	1	0	0	1	0	0	0	0	0	1	0	0	0	3
**4.709**	0	0	1	0	0	0	1	0	0	1	0	1	0	0	0	0	0	0	0	1	0	1	0	0	1	3
**4.710**	0	0	1	0	0	0	0	0	1	1	0	0	0	1	0	0	0	0	0	1	0	0	0	1	1	3
**4.711**	0	0	0	1	1	0	0	0	0	0	0	0	1	0	0	0	0	0	0	0	0	1	0	0	0	3
**4.712**	0	0	0	1	0	0	1	0	1	1	1	1	0	1	0	0	0	0	0	1	0	0	0	1	1	3
**4.713**	0	0	0	1	0	0	0	0	1	0	0	0	0	0	0	0	0	0	0	0	0	1	1	0	0	3
**4.714**	0	0	0	1	0	0	0	0	0	1	0	0	0	1	0	0	0	0	0	0	0	0	0	0	0	3
**4.715**	0	0	0	0	1	0	0	0	1	0	0	0	0	0	0	0	0	0	0	0	0	0	0	1	0	3
**4.716**	0	0	0	0	0	0	1	0	1	1	0	1	1	1	0	0	0	0	0	1	0	1	0	0	1	3
**4.717**	0	0	0	0	0	0	1	0	0	1	1	0	0	1	0	0	0	0	0	0	0	1	0	0	1	3
**4.718**	0	0	0	0	0	0	1	0	0	0	0	1	0	1	0	0	0	0	0	1	0	1	1	1	0	3
**4.719**	0	0	0	0	0	0	0	0	1	1	0	1	1	1	0	0	0	0	0	1	0	0	1	0	1	3
**4.720**	0	0	0	0	0	0	0	0	1	1	0	0	0	0	0	0	0	0	0	1	0	0	1	0	0	3
**4.721**	0	0	0	0	0	0	0	0	1	0	0	0	0	0	0	0	0	0	0	1	0	0	1	0	1	3
**4.722**	0	0	0	0	0	0	0	0	0	1	1	0	0	1	0	0	0	0	0	0	0	1	0	0	1	3
**4.723**	0	0	0	0	0	0	0	0	0	0	0	0	0	1	0	0	0	0	0	1	0	1	1	0	1	3
**4.724**	0	1	0	1	1	0	0	0	1	1	0	0	0	0	0	0	0	0	0	1	0	0	1	0	0	3
**4.725**	0	1	0	0	1	0	1	0	0	1	0	1	0	1	0	0	0	0	0	1	0	0	0	0	0	3
**4.726**	0	1	0	0	1	0	1	0	0	1	0	1	0	1	0	0	0	0	0	0	0	0	0	1	1	3
**4.727**	0	1	0	0	1	0	0	0	0	1	1	1	0	1	0	0	0	0	0	0	0	0	1	0	1	3
**4.728**	0	1	0	0	0	0	0	0	1	0	0	1	0	1	0	0	0	0	0	0	0	0	0	0	0	3
**4.729**	0	0	1	1	0	0	0	0	1	0	1	0	0	0	0	0	0	0	0	0	0	1	0	0	0	3
**4.730**	0	0	1	1	0	0	0	0	0	1	0	0	0	1	0	0	0	0	0	1	0	0	0	0	0	3
**4.731**	0	0	1	0	0	0	1	0	0	1	1	1	0	1	0	1	0	0	0	1	0	0	0	1	1	3
**4.732**	0	0	1	0	0	0	0	0	1	1	1	0	0	1	0	0	0	0	0	0	0	0	0	1	1	3
**4.733**	0	0	1	0	0	0	0	0	1	1	0	0	0	0	0	0	0	0	0	0	0	0	0	1	1	3
**4.734**	0	0	1	0	0	0	0	0	0	1	0	0	0	1	0	0	0	0	0	1	0	1	0	0	1	3
**4.735**	0	0	1	0	0	0	0	0	0	0	0	0	0	0	0	1	0	0	0	0	0	0	0	0	1	3
**4.736**	0	0	0	1	1	0	1	0	0	1	0	1	0	1	0	0	0	0	0	0	0	1	1	0	0	3
**4.737**	0	0	0	1	0	0	1	0	0	1	0	1	1	1	0	1	0	0	0	1	0	0	0	1	1	3
**4.738**	0	0	0	0	0	0	1	0	0	1	1	0	0	0	0	0	0	0	0	0	0	0	0	0	0	3
**4.739**	0	0	0	0	0	0	1	0	0	1	0	0	1	1	0	0	0	0	0	1	0	1	0	0	1	3
**4.740**	0	0	0	0	0	0	1	0	0	0	0	1	0	1	0	0	0	0	0	1	0	0	1	0	0	3
**4.741**	0	0	0	0	0	0	0	0	0	0	1	1	0	0	0	1	0	0	0	0	0	1	0	0	0	3
**4.742**	0	0	0	0	0	0	0	0	0	0	0	0	0	1	0	0	0	0	0	1	0	0	1	1	0	3
**4.743**	0	1	0	1	0	0	0	0	0	0	0	0	1	0	0	0	0	0	0	0	0	1	1	0	0	3
**4.744**	0	1	0	0	1	0	0	0	1	0	0	1	1	0	0	0	0	0	0	0	0	1	1	0	1	3
**4.745**	0	1	0	0	1	0	0	0	0	1	0	1	1	1	0	0	0	0	0	0	0	0	1	0	1	3
**4.746**	0	1	0	0	0	0	0	0	0	1	0	0	0	0	0	0	0	0	0	1	0	1	0	0	0	3
**4.747**	0	1	0	0	0	0	0	0	0	0	1	1	0	1	0	0	0	0	0	0	0	0	0	0	0	3
**4.748**	0	1	0	0	0	0	0	0	0	0	0	0	1	0	0	0	0	0	0	0	0	1	0	1	0	3
**4.749**	0	0	1	1	0	0	0	0	0	0	0	1	0	0	0	0	0	0	0	0	0	0	0	1	1	3
**4.750**	0	0	1	0	0	0	1	0	0	1	0	1	0	0	0	1	0	0	0	1	0	0	0	1	1	3
**4.751**	0	0	1	0	0	0	0	0	1	1	0	1	0	0	0	1	0	0	0	0	0	0	0	1	1	3
**4.752**	0	0	1	0	0	0	0	0	0	1	0	0	0	0	0	1	0	0	0	0	0	0	0	1	0	3
**4.753**	0	0	1	0	0	0	0	0	0	0	0	1	0	0	0	1	0	0	0	1	0	0	0	1	1	3
**4.754**	0	0	0	1	0	0	1	0	0	0	0	1	0	1	0	0	0	0	0	0	0	1	0	0	0	3
**4.755**	0	0	0	1	0	0	1	0	0	0	0	0	1	0	0	1	0	0	0	1	0	0	0	0	0	3
**4.756**	0	0	0	1	0	0	1	0	0	0	0	0	1	0	0	0	0	0	0	1	0	0	0	0	0	3
**4.757**	0	0	0	1	0	0	0	0	0	0	1	0	0	0	0	0	0	0	0	1	0	1	0	0	0	3

CMP	CD2	CD3	CD4	CD7	CD8	CD10	CD13	CD18	CD26	CD29	CD36	CD44	CD45RA	CD49f	CD54	CD56	CD57	CD58	CD62L	CD71	CD80	Cytokeratin	Histone_H1	HLA-DQ	HLA-DR	Freq. (voxel)
**4.758**	0	0	0	0	1	0	1	0	1	0	0	0	1	0	0	0	0	0	0	1	0	0	0	1	0	3
**4.759**	0	0	0	0	1	0	1	0	0	1	0	1	1	1	0	0	0	0	0	0	0	1	1	0	0	3
**4.760**	0	0	0	0	0	0	1	0	0	1	0	0	1	1	0	0	0	0	0	0	0	1	0	0	1	3
**4.761**	0	0	0	0	0	0	1	0	0	0	0	0	0	1	0	1	0	0	0	0	0	0	0	0	0	3
**4.762**	0	0	0	0	0	0	0	0	0	1	1	0	0	1	0	0	0	0	0	1	0	0	0	1	1	3
**4.763**	0	0	0	0	0	0	0	0	0	1	0	0	0	0	0	0	0	0	0	1	0	1	1	1	0	3
**4.764**	0	1	0	1	1	0	0	0	0	0	0	1	0	0	0	0	0	0	0	0	0	1	1	0	1	3
**4.765**	0	0	1	1	0	0	0	0	0	1	0	1	0	0	0	0	0	0	0	0	0	0	0	1	1	3
**4.766**	0	0	0	1	1	0	1	0	0	0	0	1	1	0	0	0	0	0	0	1	0	0	0	0	0	3
**4.767**	0	0	0	1	0	0	0	0	1	0	0	1	1	0	0	0	0	0	0	0	0	0	0	0	0	3
**4.768**	0	0	0	0	0	0	0	0	1	0	1	0	0	0	0	0	0	0	0	0	0	1	1	1	1	3
**4.769**	0	0	0	0	0	0	0	0	0	1	1	1	0	1	0	0	0	0	0	0	0	0	1	0	0	3
**4.770**	0	0	0	0	0	0	0	0	0	1	0	0	0	1	0	1	0	0	0	0	0	1	0	0	0	3
**4.771**	0	1	0	1	0	0	0	0	0	0	0	0	0	0	0	0	0	0	0	1	0	0	0	1	0	3
**4.772**	0	0	1	1	0	0	0	0	1	0	0	0	0	0	0	0	0	0	0	1	0	1	0	0	0	3
**4.773**	0	0	0	1	0	0	0	0	0	0	0	0	0	1	0	0	0	0	0	1	0	0	0	1	1	3
**4.774**	0	0	0	0	1	0	1	0	0	0	0	1	0	1	0	0	0	0	0	0	0	0	0	0	0	3
**4.775**	0	0	0	0	1	0	1	0	0	0	0	0	1	0	0	1	0	0	0	1	0	0	0	1	0	3
**4.776**	0	0	0	0	1	0	0	0	0	1	1	1	0	1	0	0	0	0	0	0	0	0	0	0	1	3
**4.777**	0	0	0	0	0	0	1	0	0	0	1	0	0	1	0	0	0	0	0	0	0	0	0	0	0	3
**4.778**	0	0	0	0	0	0	0	0	0	0	0	0	0	1	0	1	0	0	0	1	0	1	1	0	0	3
**4.779**	0	1	0	1	0	0	1	0	1	0	0	1	0	0	0	0	0	0	0	1	0	1	0	0	0	3
**4.780**	0	0	1	0	0	0	0	0	0	1	0	0	0	0	0	0	0	0	0	1	0	0	1	0	0	3
**4.781**	0	0	1	0	0	0	0	0	0	0	0	1	0	0	0	1	0	0	0	0	0	0	0	1	0	3
**4.782**	0	0	0	0	1	0	1	0	0	0	1	1	0	0	0	0	0	0	0	0	0	0	0	0	1	3
**4.783**	0	0	0	0	1	0	0	0	0	1	0	0	0	1	0	0	0	0	0	0	0	1	0	0	0	3
**4.784**	0	1	0	0	0	0	1	0	0	0	0	1	1	0	0	0	0	0	0	0	0	1	0	0	0	3
**4.785**	0	1	0	0	0	0	1	0	0	0	0	1	1	0	0	0	0	0	0	0	0	0	1	0	0	3
**4.786**	0	0	0	1	0	0	0	0	0	0	0	1	0	0	0	1	0	0	0	0	0	0	0	0	0	3
**4.787**	0	0	0	0	0	0	1	0	0	0	1	0	0	0	0	0	0	0	0	0	0	0	0	1	0	3
**4.788**	0	1	0	1	0	0	0	0	1	0	0	0	0	0	0	0	0	0	0	1	0	0	0	0	1	3
**4.789**	0	1	0	0	1	0	1	0	0	1	0	1	1	0	0	0	0	0	0	1	0	1	0	0	0	3
**4.790**	0	1	0	0	1	0	1	0	0	1	0	1	1	0	0	0	0	0	0	0	0	1	1	0	0	3
**4.791**	0	1	0	0	0	0	1	0	0	0	0	1	0	0	0	0	0	0	0	0	0	1	0	0	0	3
**4.792**	0	1	0	0	0	0	0	0	0	0	0	0	1	0	0	0	0	0	0	1	0	0	0	0	1	3
**4.793**	0	0	1	0	0	0	0	0	1	0	0	0	0	0	0	0	0	0	0	0	0	0	1	1	1	3
**4.794**	0	0	0	0	0	0	0	0	1	0	0	1	0	0	0	0	0	0	0	0	0	0	1	0	1	3
**4.795**	0	1	0	0	0	0	1	0	0	0	0	1	0	0	0	0	0	0	0	0	0	1	1	0	0	3
**4.796**	0	0	1	0	0	0	0	0	0	0	0	0	0	1	0	1	0	0	0	0	0	0	0	1	0	3
**4.797**	0	0	0	0	0	0	1	0	1	0	0	0	1	0	0	1	0	0	0	1	0	1	0	1	0	3
**4.798**	0	1	1	1	0	0	1	0	1	1	0	1	1	0	0	0	0	0	0	1	0	1	0	1	1	2
**4.799**	0	1	1	0	1	0	1	0	1	1	0	1	0	0	0	0	0	0	0	1	0	1	0	1	1	2
**4.800**	0	1	1	0	1	0	1	0	1	0	0	1	1	0	0	0	0	0	0	1	0	1	0	1	1	2
**4.801**	0	1	1	0	1	0	0	0	0	0	0	1	1	0	0	0	0	0	0	0	0	0	0	0	1	2
**4.802**	0	1	1	0	0	0	0	0	1	1	0	1	0	0	0	0	0	0	0	0	0	1	1	1	1	2
**4.803**	0	1	0	1	1	0	1	0	1	1	0	1	1	0	0	0	0	0	0	1	0	0	0	1	0	2
**4.804**	0	1	0	1	1	0	1	0	1	1	0	1	0	0	0	0	0	0	0	0	0	0	1	1	1	2
**4.805**	0	1	0	1	1	0	1	0	0	0	0	1	1	0	0	0	0	0	0	0	0	0	0	0	1	2
**4.806**	0	1	0	1	1	0	0	0	1	1	0	1	1	1	0	0	0	0	0	0	0	0	1	0	0	2
**4.807**	0	1	0	1	1	0	0	0	1	1	0	1	0	1	0	0	0	0	0	0	0	0	0	1	0	2
**4.808**	0	1	0	1	1	0	0	0	1	1	0	1	0	0	0	1	0	0	0	0	0	0	0	0	1	2
**4.809**	0	1	0	1	1	0	0	0	1	0	0	1	1	0	0	0	0	0	0	1	0	0	1	1	1	2
**4.810**	0	1	0	1	1	0	0	0	1	0	0	1	0	0	0	0	0	0	0	1	0	1	1	1	0	2
**4.811**	0	1	0	1	1	0	0	0	1	0	0	1	0	0	0	0	0	0	0	0	0	1	1	1	0	2
**4.812**	0	1	0	1	1	0	0	0	1	0	0	1	0	0	0	0	0	0	0	0	0	1	0	1	0	2
**4.813**	0	1	0	1	1	0	0	0	0	0	0	1	1	1	0	0	0	0	0	1	0	0	1	0	0	2
**4.814**	0	1	0	1	1	0	0	0	0	0	0	1	0	1	0	0	0	0	0	1	0	0	0	1	1	2
**4.815**	0	1	0	1	0	0	0	0	1	0	0	0	1	0	0	0	0	0	0	0	0	0	1	0	1	2
**4.816**	0	1	0	0	1	0	1	0	1	1	0	1	1	1	0	0	0	0	0	1	0	0	0	0	0	2
**4.817**	0	1	0	0	1	0	1	0	1	0	0	1	1	1	0	0	0	0	0	1	0	1	0	1	1	2
**4.818**	0	1	0	0	1	0	1	0	1	0	0	1	0	1	0	0	0	0	0	0	0	1	0	1	1	2
**4.819**	0	1	0	0	1	0	0	0	1	1	0	1	0	1	0	0	0	0	0	0	0	0	0	1	0	2
**4.820**	0	1	0	0	1	0	0	0	1	1	0	1	0	0	0	1	0	0	0	0	0	0	0	0	1	2
**4.821**	0	1	0	0	1	0	0	0	1	1	0	1	0	0	0	1	0	0	0	0	0	0	0	0	0	2
**4.822**	0	1	0	0	1	0	0	0	1	1	0	1	0	0	0	0	0	0	0	0	0	1	0	1	0	2
**4.823**	0	1	0	0	1	0	0	0	0	1	0	1	0	0	0	0	0	0	0	1	0	1	0	1	0	2
**4.824**	0	1	0	0	1	0	0	0	0	0	0	1	0	1	0	0	0	0	0	0	0	1	1	0	0	2
**4.825**	0	1	0	0	0	0	1	0	0	0	0	1	0	0	0	0	0	0	0	1	0	1	0	1	1	2
**4.826**	0	1	0	0	0	0	0	0	1	0	0	0	0	0	0	0	0	0	0	0	0	1	1	0	1	2
**4.827**	0	0	1	1	0	0	1	0	1	1	0	0	0	0	0	0	0	0	0	0	0	0	0	0	0	2
**4.828**	0	0	1	1	0	0	0	0	1	0	0	1	1	0	0	0	0	0	0	0	0	0	0	1	0	2
**4.829**	0	0	1	1	0	0	0	0	1	0	0	1	0	0	0	0	0	0	0	0	0	1	0	1	0	2
**4.830**	0	0	1	1	0	0	0	0	1	0	0	0	0	1	0	0	0	0	0	0	0	1	0	1	0	2
**4.831**	0	0	1	1	0	0	0	0	0	0	0	1	0	0	0	0	0	0	0	1	0	1	0	1	1	2
**4.832**	0	0	1	0	1	0	0	0	1	1	0	1	1	0	0	0	0	0	0	1	0	0	0	1	1	2
**4.833**	0	0	1	0	1	0	0	0	1	1	0	1	0	0	0	0	0	0	0	1	0	0	0	0	1	2
**4.834**	0	0	1	0	1	0	0	0	0	1	0	1	0	0	0	0	0	0	0	1	0	0	0	0	1	2
**4.835**	0	0	1	0	1	0	0	0	0	1	0	0	0	0	0	0	0	0	0	0	0	0	0	0	0	2
**4.836**	0	0	1	0	1	0	0	0	0	0	0	1	0	0	0	0	0	0	0	0	0	1	0	1	1	2
**4.837**	0	0	1	0	0	0	1	0	1	0	0	0	0	0	0	0	0	0	0	0	0	1	0	1	1	2
**4.838**	0	0	0	1	1	0	1	0	1	1	0	1	1	0	0	0	0	0	0	1	0	0	0	1	1	2
**4.839**	0	0	0	1	1	0	1	0	0	0	0	1	0	0	0	0	0	0	0	0	0	0	0	1	0	2
**4.840**	0	0	0	1	1	0	0	0	1	1	0	1	1	0	0	0	0	0	0	1	0	1	0	0	0	2
**4.841**	0	0	0	1	1	0	0	0	1	0	0	1	1	1	0	0	0	0	0	1	0	0	0	1	1	2
**4.842**	0	0	0	1	1	0	0	0	1	0	0	1	0	0	0	0	0	0	0	1	0	0	0	1	0	2
**4.843**	0	0	0	1	1	0	0	0	0	0	0	1	0	0	0	0	0	0	0	1	0	1	0	1	0	2
**4.844**	0	0	0	1	1	0	0	0	0	0	0	1	0	0	0	0	0	0	0	0	0	1	1	1	1	2
**4.845**	0	0	0	1	0	0	1	0	1	1	0	1	1	0	0	0	0	0	0	1	0	0	0	1	1	2
**4.846**	0	0	0	1	0	0	1	0	1	1	0	0	1	0	0	0	0	0	0	1	0	0	0	1	1	2
**4.847**	0	0	0	1	0	0	1	0	1	0	0	1	0	0	0	0	0	0	0	0	0	0	1	1	0	2
**4.848**	0	0	0	1	0	0	1	0	0	0	0	1	0	1	0	0	0	0	0	1	0	0	0	1	1	2
**4.849**	0	0	0	1	0	0	1	0	0	0	0	1	0	0	0	1	0	0	0	0	0	0	0	1	1	2
**4.850**	0	0	0	1	0	0	1	0	0	0	0	1	0	0	0	0	0	0	0	1	0	0	0	1	0	2
**4.851**	0	0	0	1	0	0	0	0	1	1	0	1	0	1	0	0	0	0	0	1	0	0	0	0	0	2
**4.852**	0	0	0	1	0	0	0	0	1	0	0	1	1	1	0	0	0	0	0	0	0	1	0	1	0	2
**4.853**	0	0	0	1	0	0	0	0	1	0	0	0	0	0	0	0	0	0	0	1	0	0	0	0	1	2
**4.854**	0	0	0	1	0	0	0	0	0	0	0	1	0	1	0	0	0	0	0	1	0	1	0	0	1	2
**4.855**	0	0	0	0	1	0	1	0	1	0	0	1	0	1	0	0	0	0	0	1	0	1	0	1	1	2
**4.856**	0	0	0	0	1	0	1	0	1	0	0	1	0	0	0	0	0	0	0	1	0	0	0	0	1	2
**4.857**	0	0	0	0	1	0	1	0	1	0	0	1	0	0	0	0	0	0	0	0	0	0	1	1	1	2
**4.858**	0	0	0	0	1	0	0	0	1	1	0	1	0	1	0	0	0	0	0	1	0	1	0	1	0	2
**4.859**	0	0	0	0	1	0	0	0	1	0	0	1	1	0	0	0	0	0	0	1	0	0	0	1	1	2
**4.860**	0	0	0	0	1	0	0	0	1	0	0	1	0	0	0	0	0	0	0	1	0	1	1	1	1	2
**4.861**	0	0	0	0	1	0	0	0	1	0	0	1	0	0	0	0	0	0	0	0	0	1	1	1	0	2
**4.862**	0	0	0	0	1	0	0	0	0	1	0	0	0	0	0	0	0	0	0	0	0	0	0	1	0	2
**4.863**	0	0	0	0	1	0	0	0	0	0	0	1	1	1	0	0	0	0	0	0	0	0	0	1	1	2
**4.864**	0	0	0	0	0	0	1	0	1	0	0	1	1	0	0	0	0	0	0	0	0	1	0	0	0	2
**4.865**	0	0	0	0	0	0	1	0	1	0	0	1	0	0	0	1	0	0	0	0	0	0	0	1	0	2
**4.866**	0	0	0	0	0	0	1	0	1	0	0	0	0	0	0	0	0	0	0	0	0	1	0	1	0	2
**4.867**	0	0	0	0	0	0	0	0	1	0	0	1	0	0	0	1	0	0	0	1	0	0	0	1	0	2
**4.868**	0	0	0	0	0	0	0	0	1	0	0	0	0	0	0	1	0	0	0	0	0	0	1	1	0	2
**4.869**	0	0	0	0	0	0	0	0	1	0	0	0	0	0	0	0	0	0	0	1	0	1	1	0	1	2
**4.870**	0	0	0	0	0	0	0	0	0	1	0	1	0	0	0	1	0	0	0	1	0	1	0	0	0	2
**4.871**	0	0	0	0	0	0	0	0	0	0	0	1	1	1	0	0	0	0	0	0	0	0	0	0	0	2
**4.872**	0	1	1	1	1	0	1	0	1	1	0	1	1	1	0	0	0	0	0	1	0	0	1	0	0	2
**4.873**	0	1	1	1	1	0	1	0	1	0	0	1	0	0	0	1	0	0	0	1	0	0	0	1	1	2
**4.874**	0	1	1	1	1	0	0	0	1	1	0	1	1	1	0	0	0	0	0	0	0	0	0	1	0	2
**4.875**	0	1	1	1	1	0	0	0	1	1	0	1	0	1	0	0	0	0	0	1	0	1	0	1	1	2
**4.876**	0	1	1	1	1	0	0	0	0	1	0	1	0	1	0	0	0	0	0	0	0	0	0	0	0	2
**4.877**	0	1	1	0	1	0	1	0	1	1	0	1	1	0	0	0	0	0	0	1	0	0	0	0	0	2
**4.878**	0	1	1	0	1	0	0	0	1	1	0	1	1	1	0	0	0	0	0	0	0	0	1	0	0	2

CMP	CD2	CD3	CD4	CD7	CD8	CD10	CD13	CD18	CD26	CD29	CD36	CD44	CD45RA	CD49f	CD54	CD56	CD57	CD58	CD62L	CD71	CD80	Cytokeratin	Histone_H1	HLA-DQ	HLA-DR	Freq. (voxel)
**4.879**	0	1	1	0	1	0	0	0	1	0	0	1	1	1	0	0	0	0	0	1	0	1	0	1	1	2
**4.880**	0	1	1	0	1	0	0	0	1	0	0	1	1	1	0	0	0	0	0	1	0	0	0	1	1	2
**4.881**	0	1	1	0	1	0	0	0	1	0	0	1	0	1	0	0	0	0	0	0	0	0	0	0	0	2
**4.882**	0	1	1	0	1	0	0	0	0	1	0	1	0	1	0	0	0	0	0	1	0	0	0	0	0	2
**4.883**	0	1	1	0	0	0	0	0	1	0	0	0	0	0	0	0	0	0	0	1	0	1	1	1	1	2
**4.884**	0	1	1	0	0	0	0	0	0	0	0	1	0	0	0	0	0	0	0	1	0	1	0	1	1	2
**4.885**	0	1	0	1	1	0	1	0	1	1	0	1	1	1	0	0	0	0	0	1	0	0	1	1	0	2
**4.886**	0	1	0	1	1	0	1	0	1	0	0	1	0	0	0	0	0	0	0	0	0	0	0	1	0	2
**4.887**	0	1	0	1	1	0	1	0	0	1	0	1	1	1	0	0	0	0	0	1	0	1	0	0	0	2
**4.888**	0	1	0	1	1	0	1	0	0	0	0	1	0	0	0	0	0	0	0	0	0	0	1	1	0	2
**4.889**	0	1	0	1	1	0	0	0	1	1	0	1	1	1	0	0	0	0	0	1	0	1	0	1	1	2
**4.890**	0	1	0	1	1	0	0	0	1	1	0	1	1	1	0	0	0	0	0	1	0	1	0	1	0	2
**4.891**	0	1	0	1	1	0	0	0	1	1	0	1	0	0	0	0	0	0	0	0	0	1	1	0	0	2
**4.892**	0	1	0	1	1	0	0	0	1	0	0	1	1	1	0	0	0	0	0	1	0	1	0	1	1	2
**4.893**	0	1	0	1	1	0	0	0	1	0	0	1	1	1	0	0	0	0	0	0	0	0	0	1	1	2
**4.894**	0	1	0	1	1	0	0	0	0	1	0	1	1	1	0	0	0	0	0	1	0	1	0	0	0	2
**4.895**	0	1	0	1	0	0	1	0	0	0	0	1	0	0	0	0	0	0	0	0	0	0	1	1	0	2
**4.896**	0	1	0	0	1	0	1	0	0	1	0	1	1	1	0	0	0	0	0	1	0	0	1	1	0	2
**4.897**	0	1	0	0	1	0	0	0	0	1	0	1	1	1	0	0	0	0	0	1	0	0	1	1	0	2
**4.898**	0	1	0	0	1	0	0	0	0	0	0	0	1	0	0	0	0	0	0	1	0	0	1	0	1	2
**4.899**	0	1	0	0	0	0	0	0	1	1	0	1	1	0	0	0	0	0	0	0	0	1	1	1	1	2
**4.900**	0	1	0	0	0	0	0	0	1	1	0	1	0	0	0	0	0	0	0	0	0	1	1	1	1	2
**4.901**	0	1	0	0	0	0	0	0	0	0	0	1	0	1	0	0	0	0	0	1	0	1	1	0	0	2
**4.902**	0	0	1	1	1	0	0	0	1	0	0	1	0	0	0	0	0	0	0	0	0	0	0	0	0	2
**4.903**	0	0	1	1	0	0	1	0	1	0	0	0	1	0	0	0	0	0	0	0	0	0	0	0	0	2
**4.904**	0	0	1	1	0	0	0	0	1	0	0	0	0	0	0	0	0	0	0	1	0	0	0	0	0	2
**4.905**	0	0	1	0	1	0	0	0	1	1	0	1	0	0	0	0	0	0	0	1	0	0	1	0	0	2
**4.906**	0	0	1	0	1	0	0	0	0	1	0	1	1	0	0	0	0	0	0	0	0	0	1	0	0	2
**4.907**	0	0	1	0	1	0	0	0	0	1	0	1	0	0	0	0	0	0	0	0	0	1	0	0	1	2
**4.908**	0	0	1	0	1	0	0	0	0	0	0	0	1	1	0	0	0	0	0	1	0	0	0	0	0	2
**4.909**	0	0	1	0	1	0	0	0	0	0	0	0	1	0	0	0	0	0	0	1	0	0	0	0	0	2
**4.910**	0	0	1	0	0	0	1	0	1	0	0	1	0	0	0	0	0	0	0	0	0	0	1	1	1	2
**4.911**	0	0	0	1	1	0	1	0	1	0	0	1	0	0	0	0	0	0	0	0	0	0	1	0	1	2
**4.912**	0	0	0	1	1	0	0	0	1	0	0	1	1	0	0	0	0	0	0	1	0	0	0	0	1	2
**4.913**	0	0	0	1	1	0	0	0	1	0	0	1	1	0	0	0	0	0	0	1	0	0	0	0	0	2
**4.914**	0	0	0	1	1	0	0	0	1	0	0	1	0	0	0	0	0	0	0	1	0	0	1	0	0	2
**4.915**	0	0	0	1	1	0	0	0	1	0	0	0	0	0	0	0	0	0	0	1	0	0	0	0	0	2
**4.916**	0	0	0	1	1	0	0	0	0	1	0	1	1	0	0	0	0	0	0	1	0	0	0	1	1	2
**4.917**	0	0	0	1	1	0	0	0	0	1	0	1	1	0	0	0	0	0	0	0	0	1	1	0	0	2
**4.918**	0	0	0	1	1	0	0	0	0	0	0	1	1	0	0	0	0	0	0	1	0	1	0	1	1	2
**4.919**	0	0	0	1	1	0	0	0	0	0	0	0	0	0	0	0	0	0	0	1	0	0	0	0	1	2
**4.920**	0	0	0	1	0	0	1	0	1	1	0	1	1	0	0	0	0	0	0	1	0	1	0	0	0	2
**4.921**	0	0	0	1	0	0	1	0	1	1	0	1	1	0	0	0	0	0	0	1	0	0	0	1	0	2
**4.922**	0	0	0	1	0	0	1	0	1	0	0	1	0	0	0	1	0	0	0	0	0	0	0	1	1	2
**4.923**	0	0	0	1	0	0	1	0	0	0	0	0	1	0	0	1	0	0	0	0	0	0	0	1	0	2
**4.924**	0	0	0	1	0	0	1	0	0	0	0	0	1	0	0	0	0	0	0	1	0	0	0	1	0	2
**4.925**	0	0	0	1	0	0	0	0	1	1	0	1	1	0	0	0	0	0	0	1	0	1	0	1	0	2
**4.926**	0	0	0	1	0	0	0	0	1	1	0	1	1	0	0	0	0	0	0	0	0	0	1	0	0	2
**4.927**	0	0	0	1	0	0	0	0	1	1	0	0	0	0	0	0	0	0	0	1	0	0	1	0	0	2
**4.928**	0	0	0	1	0	0	0	0	1	1	0	0	0	0	0	0	0	0	0	1	0	0	0	0	0	2
**4.929**	0	0	0	1	0	0	0	0	1	0	0	1	1	0	0	0	0	0	0	0	0	1	0	0	0	2
**4.930**	0	0	0	1	0	0	0	0	1	0	0	1	0	1	0	0	0	0	0	1	0	0	0	1	0	2
**4.931**	0	0	0	1	0	0	0	0	1	0	0	1	0	0	0	0	0	0	0	0	0	0	0	1	0	2
**4.932**	0	0	0	1	0	0	0	0	1	0	0	0	0	0	0	0	0	0	0	0	0	0	1	1	1	2
**4.933**	0	0	0	1	0	0	0	0	0	1	0	1	0	0	0	1	0	0	0	1	0	0	0	0	0	2
**4.934**	0	0	0	1	0	0	0	0	0	1	0	0	0	0	0	1	0	0	0	0	0	0	0	0	0	2
**4.935**	0	0	0	1	0	0	0	0	0	0	0	1	0	1	0	0	0	0	0	0	0	1	1	0	0	2
**4.936**	0	0	0	1	0	0	0	0	0	0	0	0	1	0	0	1	0	0	0	1	0	0	1	0	0	2
**4.937**	0	0	0	1	0	0	0	0	0	0	0	0	1	0	0	1	0	0	0	0	0	0	1	0	0	2
**4.938**	0	0	0	0	1	0	1	0	1	1	0	1	0	0	0	0	0	0	0	0	0	1	0	1	1	2
**4.939**	0	0	0	0	1	0	1	0	0	0	0	1	0	1	0	0	0	0	0	0	0	0	0	1	1	2
**4.940**	0	0	0	0	1	0	0	0	1	1	0	1	1	1	0	0	0	0	0	0	0	0	0	1	0	2
**4.941**	0	0	0	0	1	0	0	0	1	1	0	1	1	0	0	0	0	0	0	1	0	0	0	0	1	2
**4.942**	0	0	0	0	1	0	0	0	1	1	0	1	1	0	0	0	0	0	0	0	0	0	0	0	1	2
**4.943**	0	0	0	0	1	0	0	0	1	1	0	0	0	0	0	0	0	0	0	1	0	0	0	0	0	2
**4.944**	0	0	0	0	1	0	0	0	1	0	0	1	0	0	0	0	0	0	0	1	0	0	1	0	1	2
**4.945**	0	0	0	0	1	0	0	0	1	0	0	1	0	0	0	0	0	0	0	1	0	0	0	1	0	2
**4.946**	0	0	0	0	1	0	0	0	0	1	0	1	1	1	0	0	0	0	0	1	0	0	1	0	0	2
**4.947**	0	0	0	0	1	0	0	0	0	1	0	0	0	0	0	0	0	0	0	1	0	1	1	0	0	2
**4.948**	0	0	0	0	1	0	0	0	0	0	0	1	1	0	0	0	0	0	0	1	0	1	1	1	1	2
**4.949**	0	0	0	0	1	0	0	0	0	0	0	1	0	1	0	0	0	0	0	1	0	0	0	1	1	2
**4.950**	0	0	0	0	1	0	0	0	0	0	0	1	0	1	0	0	0	0	0	0	0	0	0	1	0	2
**4.951**	0	0	0	0	0	0	1	0	1	1	0	1	1	0	0	0	0	0	0	1	0	1	1	0	0	2
**4.952**	0	0	0	0	0	0	1	0	1	0	0	1	0	0	0	1	0	0	0	0	0	0	1	1	1	2
**4.953**	0	0	0	0	0	0	1	0	1	0	0	0	0	0	0	1	0	0	0	0	0	0	1	1	1	2
**4.954**	0	0	0	0	0	0	1	0	1	0	0	0	0	0	0	0	0	0	0	0	0	1	1	1	1	2
**4.955**	0	0	0	0	0	0	1	0	0	0	0	1	0	0	0	1	0	0	0	0	0	0	0	0	0	2
**4.956**	0	0	0	0	0	0	0	0	1	1	0	1	0	1	0	0	0	0	0	0	0	0	1	0	0	2
**4.957**	0	0	0	0	0	0	0	0	1	1	0	1	0	0	0	1	0	0	0	0	0	0	1	1	1	2
**4.958**	0	0	0	0	0	0	0	0	1	1	0	1	0	0	0	0	0	0	0	1	0	0	1	1	1	2
**4.959**	0	0	0	0	0	0	0	0	1	0	0	1	0	0	0	0	0	0	0	1	0	1	1	1	1	2
**4.960**	0	0	0	0	0	0	0	0	0	0	1	1	0	1	0	0	0	0	0	1	0	0	0	1	0	2
**4.961**	0	1	1	1	1	0	0	0	1	1	0	1	1	1	0	0	0	0	0	0	0	0	0	0	0	2
**4.962**	0	1	1	1	0	0	0	0	1	0	0	1	1	0	0	0	0	0	0	1	0	0	0	0	0	2
**4.963**	0	1	1	0	1	0	1	0	0	1	0	1	1	0	0	0	0	0	0	1	0	1	0	1	1	2
**4.964**	0	1	1	0	1	0	0	0	1	1	0	1	1	0	0	0	0	0	0	1	0	0	1	1	0	2
**4.965**	0	1	1	0	1	0	0	0	1	1	0	1	0	0	0	0	0	0	0	1	0	1	0	0	0	2
**4.966**	0	1	1	0	1	0	0	0	1	0	0	1	1	0	0	0	0	0	0	1	0	0	1	1	1	2
**4.967**	0	1	1	0	0	0	1	0	1	1	0	0	0	0	0	0	0	0	0	1	0	0	0	1	1	2
**4.968**	0	1	1	0	0	0	0	0	1	0	0	1	0	0	0	0	0	0	0	0	0	1	1	1	1	2
**4.969**	0	1	1	0	0	0	0	0	0	0	0	0	0	0	0	0	0	0	0	1	0	0	0	1	1	2
**4.970**	0	1	0	1	1	0	1	0	1	1	0	1	0	0	0	0	0	0	0	1	0	1	0	1	1	2
**4.971**	0	1	0	1	1	0	1	0	0	1	0	1	0	0	0	0	0	0	0	0	0	0	1	1	0	2
**4.972**	0	1	0	1	1	0	1	0	0	0	0	1	1	0	0	0	0	0	0	1	0	1	0	1	1	2
**4.973**	0	1	0	1	1	0	0	0	1	1	0	1	1	0	0	0	0	0	0	1	0	1	0	1	0	2
**4.974**	0	1	0	1	1	0	0	0	1	1	0	1	0	0	0	1	0	0	0	1	0	0	0	0	0	2
**4.975**	0	1	0	1	1	0	0	0	0	0	0	1	1	1	0	0	0	0	0	1	0	1	0	1	0	2
**4.976**	0	1	0	1	0	0	0	0	1	0	0	1	0	0	0	0	0	0	0	1	0	0	1	1	1	2
**4.977**	0	1	0	1	0	0	0	0	1	0	0	1	0	0	0	0	0	0	0	0	0	0	1	0	1	2
**4.978**	0	1	0	1	0	0	0	0	0	0	0	1	1	1	0	0	0	0	0	0	0	0	0	1	0	2
**4.979**	0	1	0	1	0	0	0	0	0	0	0	1	1	0	0	0	0	0	0	0	0	1	0	0	1	2
**4.980**	0	1	0	0	1	0	1	0	0	1	0	1	0	0	0	0	0	0	0	0	0	0	1	1	1	2
**4.981**	0	1	0	0	1	0	1	0	0	0	0	1	0	0	0	0	0	0	0	0	0	0	1	1	1	2
**4.982**	0	1	0	0	1	0	1	0	0	0	0	0	0	0	0	0	0	0	0	0	0	0	1	1	1	2
**4.983**	0	1	0	0	1	0	0	0	1	1	0	1	0	1	0	0	0	0	0	1	0	1	1	0	0	2
**4.984**	0	1	0	0	1	0	0	0	1	0	0	1	0	1	0	0	0	0	0	1	0	0	0	0	0	2
**4.985**	0	1	0	0	1	0	0	0	1	0	0	0	0	0	0	0	0	0	0	1	0	0	0	1	1	2
**4.986**	0	1	0	0	1	0	0	0	0	1	0	0	0	0	0	0	0	0	0	1	0	1	1	1	1	2
**4.987**	0	1	0	0	0	0	1	0	1	1	0	1	1	0	0	0	0	0	0	0	0	0	0	0	0	2
**4.988**	0	1	0	0	0	0	1	0	0	1	0	1	0	0	0	0	0	0	0	0	0	0	0	1	0	2
**4.989**	0	1	0	0	0	0	0	0	1	0	0	1	1	1	0	0	0	0	0	1	0	1	1	0	1	2
**4.990**	0	1	0	0	0	0	0	0	1	0	0	1	1	1	0	0	0	0	0	1	0	0	1	0	0	2
**4.991**	0	1	0	0	0	0	0	0	1	0	0	1	0	1	0	0	0	0	0	0	0	1	1	0	1	2
**4.992**	0	1	0	0	0	0	0	0	0	0	0	1	1	1	0	0	0	0	0	1	0	1	1	0	1	2
**4.993**	0	1	0	0	0	0	0	0	0	0	0	1	1	0	0	0	0	0	0	1	0	1	1	1	1	2
**4.994**	0	1	0	0	0	0	0	0	0	0	0	1	1	0	0	0	0	0	0	1	0	0	1	1	0	2
**4.995**	0	1	0	0	0	0	0	0	0	0	0	1	1	0	0	0	0	0	0	1	0	0	0	1	1	2
**4.996**	0	0	1	1	1	0	0	0	1	1	0	1	0	0	0	0	0	0	0	1	0	1	0	0	0	2
**4.997**	0	0	1	1	1	0	0	0	1	1	0	1	0	0	0	0	0	0	0	0	0	0	0	0	1	2
**4.998**	0	0	1	1	0	0	1	0	1	1	0	1	1	0	0	1	0	0	0	1	0	0	0	1	1	2
**4.999**	0	0	1	1	0	0	1	0	0	1	0	0	0	0	0	0	0	0	0	1	0	0	0	1	1	2
**5.000**	0	0	1	1	0	0	0	0	1	1	0	1	0	0	0	0	0	0	0	1	0	0	0	0	0	2

CMP	CD2	CD3	CD4	CD7	CD8	CD10	CD13	CD18	CD26	CD29	CD36	CD44	CD45RA	CD49f	CD54	CD56	CD57	CD58	CD62L	CD71	CD80	Cytokeratin	Histone_H1	HLA-DQ	HLA-DR	Freq. (voxel)
**5.001**	0	0	1	1	0	0	0	0	1	0	0	1	0	0	0	0	0	0	0	1	0	1	0	0	1	2
**5.002**	0	0	1	1	0	0	0	0	1	0	0	0	0	0	0	0	0	0	0	1	0	1	0	1	1	2
**5.003**	0	0	1	1	0	0	0	0	0	1	0	1	0	0	0	0	0	0	0	1	0	1	0	0	0	2
**5.004**	0	0	1	1	0	0	0	0	0	0	0	1	0	1	0	0	0	0	0	0	0	0	0	1	1	2
**5.005**	0	0	1	1	0	0	0	0	0	0	0	0	1	0	0	1	0	0	0	1	0	1	0	0	1	2
**5.006**	0	0	1	0	1	0	0	0	0	0	0	1	0	0	0	0	0	0	0	0	0	0	1	1	1	2
**5.007**	0	0	1	0	0	0	0	0	1	0	0	1	1	0	0	0	0	0	0	1	0	1	0	1	0	2
**5.008**	0	0	1	0	0	0	0	0	0	0	0	0	1	1	0	0	0	0	0	1	0	0	0	0	0	2
**5.009**	0	0	0	1	1	0	1	0	0	1	0	1	0	0	0	0	0	0	0	0	0	0	0	0	0	2
**5.010**	0	0	0	1	1	0	0	0	0	1	0	1	0	0	0	0	0	0	0	0	0	1	0	1	1	2
**5.011**	0	0	0	1	1	0	0	0	0	1	0	1	0	0	0	0	0	0	0	0	0	1	0	1	0	2
**5.012**	0	0	0	1	0	0	1	0	1	1	0	1	0	0	0	0	0	0	0	1	0	0	0	0	0	2
**5.013**	0	0	0	1	0	0	1	0	1	0	0	0	0	0	0	1	0	0	0	1	0	0	1	1	1	2
**5.014**	0	0	0	1	0	0	1	0	0	1	0	1	1	0	0	0	0	0	0	1	0	0	0	1	1	2
**5.015**	0	0	0	1	0	0	1	0	0	1	0	1	1	0	0	0	0	0	0	0	0	0	0	1	1	2
**5.016**	0	0	0	1	0	0	0	0	1	1	0	1	0	0	0	0	0	0	0	0	0	0	0	1	0	2
**5.017**	0	0	0	1	0	0	0	0	0	0	0	1	1	0	0	0	0	0	0	0	0	0	1	0	0	2
**5.018**	0	0	0	1	0	0	0	0	0	0	0	0	1	1	0	1	0	0	0	0	0	1	0	0	0	2
**5.019**	0	0	0	0	1	0	1	0	1	0	0	0	0	0	0	1	0	0	0	1	0	0	0	1	1	2
**5.020**	0	0	0	0	1	0	0	0	1	1	0	1	0	1	0	0	0	0	0	1	0	1	0	0	1	2
**5.021**	0	0	0	0	1	0	0	0	1	1	0	1	0	1	0	0	0	0	0	0	0	0	0	0	1	2
**5.022**	0	0	0	0	1	0	0	0	1	0	0	1	1	0	0	0	0	0	0	0	0	1	1	0	0	2
**5.023**	0	0	0	0	1	0	0	0	1	0	0	1	1	0	0	0	0	0	0	0	0	0	1	0	1	2
**5.024**	0	0	0	0	1	0	0	0	1	0	0	1	0	1	0	0	0	0	0	0	0	1	0	1	1	2
**5.025**	0	0	0	0	1	0	0	0	1	0	0	0	0	0	0	0	0	0	0	0	0	0	1	1	1	2
**5.026**	0	0	0	0	1	0	0	0	0	0	0	1	0	1	0	0	0	0	0	1	0	1	0	1	1	2
**5.027**	0	0	0	0	0	0	1	0	1	1	0	1	0	0	0	0	0	0	0	0	0	1	0	1	0	2
**5.028**	0	0	0	0	0	0	1	0	1	0	0	0	1	0	0	0	0	0	0	1	0	0	0	1	1	2
**5.029**	0	0	0	0	0	0	1	0	1	0	0	0	0	0	0	1	0	0	0	1	0	0	0	1	1	2
**5.030**	0	0	0	0	0	0	0	0	1	1	0	1	0	0	0	0	0	0	0	1	0	1	1	1	0	2
**5.031**	0	0	0	0	0	0	0	0	1	0	0	1	1	0	0	0	0	0	0	1	0	1	1	0	0	2
**5.032**	0	0	0	0	0	0	0	0	1	0	0	1	0	0	0	0	0	0	0	1	0	0	1	0	1	2
**5.033**	0	0	0	0	0	0	0	0	0	1	1	1	0	1	0	0	0	0	0	1	0	0	0	1	0	2
**5.034**	0	0	0	0	0	0	0	0	0	0	0	0	0	1	0	0	0	0	0	1	0	1	1	1	0	2
**5.035**	0	1	1	1	1	0	1	0	1	1	0	1	1	0	0	0	0	0	0	1	0	0	0	0	0	2
**5.036**	0	1	1	1	1	0	1	0	0	1	0	1	0	1	0	0	0	0	0	1	0	0	0	0	0	2
**5.037**	0	1	1	1	1	0	0	0	1	1	0	1	0	0	0	0	0	0	0	1	0	0	1	0	0	2
**5.038**	0	1	1	1	0	0	0	0	1	1	0	1	0	0	0	0	0	0	0	1	0	0	0	0	1	2
**5.039**	0	1	1	1	0	0	0	0	1	1	0	1	0	0	0	0	0	0	0	0	0	0	0	0	1	2
**5.040**	0	1	1	0	1	0	1	0	0	1	0	1	1	1	0	0	0	0	0	1	0	0	1	0	0	2
**5.041**	0	1	1	0	1	0	0	0	1	0	0	1	1	0	0	0	0	0	0	0	0	0	0	0	0	2
**5.042**	0	1	1	0	1	0	0	0	1	0	0	0	0	0	0	0	0	0	0	1	0	0	0	0	1	2
**5.043**	0	1	1	0	0	0	0	0	1	1	0	1	0	1	0	0	0	0	0	1	0	1	0	0	0	2
**5.044**	0	1	1	0	0	0	0	0	1	0	0	0	0	0	0	0	0	0	0	0	0	0	0	1	1	2
**5.045**	0	1	1	0	0	0	0	0	0	1	0	1	0	1	0	0	0	0	0	1	0	0	0	0	0	2
**5.046**	0	1	1	0	0	0	0	0	0	0	0	1	1	0	0	0	0	0	0	1	0	0	0	0	0	2
**5.047**	0	1	1	0	0	0	0	0	0	0	0	1	0	0	0	0	0	0	0	0	0	0	1	1	1	2
**5.048**	0	1	0	1	1	0	1	0	1	0	0	1	1	0	0	0	0	0	0	1	0	0	0	0	0	2
**5.049**	0	1	0	1	1	0	0	0	1	0	0	1	1	1	0	0	0	0	0	1	0	0	0	1	0	2
**5.050**	0	1	0	1	1	0	0	0	1	0	0	1	0	1	0	0	0	0	0	0	0	1	0	1	1	2
**5.051**	0	1	0	1	1	0	0	0	0	1	0	1	1	0	0	0	0	0	0	1	0	1	1	1	0	2
**5.052**	0	1	0	1	1	0	0	0	0	1	0	1	0	1	0	0	0	0	0	1	0	1	0	0	0	2
**5.053**	0	1	0	1	1	0	0	0	0	1	0	1	0	0	0	0	0	0	0	0	0	1	0	1	1	2
**5.054**	0	1	0	1	1	0	0	0	0	0	0	1	1	1	0	0	0	0	0	1	0	0	1	1	0	2
**5.055**	0	1	0	1	0	0	1	0	1	1	0	1	0	1	0	0	0	0	0	1	0	1	0	1	1	2
**5.056**	0	1	0	1	0	0	1	0	1	0	0	1	0	0	0	1	0	0	0	1	0	0	1	1	1	2
**5.057**	0	1	0	1	0	0	1	0	1	0	0	0	0	0	0	0	0	0	0	1	0	0	0	0	0	2
**5.058**	0	1	0	1	0	0	1	0	0	1	0	0	0	0	0	0	0	0	0	1	0	0	0	0	0	2
**5.059**	0	1	0	1	0	0	0	0	1	1	0	0	1	0	0	0	0	0	0	0	0	0	0	0	0	2
**5.060**	0	1	0	1	0	0	0	0	0	1	0	1	0	1	0	0	0	0	0	0	0	1	0	0	0	2
**5.061**	0	1	0	1	0	0	0	0	0	0	0	1	1	1	0	0	0	0	0	1	0	0	1	0	0	2
**5.062**	0	1	0	1	0	0	0	0	0	0	0	1	1	1	0	0	0	0	0	0	0	0	1	1	0	2
**5.063**	0	1	0	1	0	0	0	0	0	0	0	1	1	0	0	0	0	0	0	1	0	1	1	1	1	2
**5.064**	0	1	0	1	0	0	0	0	0	0	0	1	0	0	0	0	0	0	0	1	0	0	1	1	0	2
**5.065**	0	1	0	0	1	0	1	0	0	1	0	1	1	1	0	0	0	0	0	1	0	1	0	0	0	2
**5.066**	0	1	0	0	1	0	0	0	1	1	1	1	0	0	0	0	0	0	0	0	0	0	0	0	0	2
**5.067**	0	1	0	0	1	0	0	0	1	1	0	1	1	0	0	0	0	0	0	1	0	1	1	0	0	2
**5.068**	0	1	0	0	1	0	0	0	1	1	0	1	0	0	0	0	0	0	0	1	0	1	1	0	0	2
**5.069**	0	1	0	0	1	0	0	0	1	0	0	1	1	1	0	0	0	0	0	0	0	0	1	0	0	2
**5.070**	0	1	0	0	1	0	0	0	0	1	1	1	1	0	0	0	0	0	0	0	0	0	0	1	0	2
**5.071**	0	1	0	0	1	0	0	0	0	0	0	1	1	1	0	0	0	0	0	1	0	0	0	1	1	2
**5.072**	0	1	0	0	1	0	0	0	0	0	0	1	1	1	0	0	0	0	0	1	0	0	0	0	1	2
**5.073**	0	1	0	0	1	0	0	0	0	0	0	0	0	0	0	0	0	0	0	1	0	1	1	1	1	2
**5.074**	0	1	0	0	0	0	1	0	1	0	0	1	0	1	0	0	0	0	0	0	0	1	0	1	1	2
**5.075**	0	1	0	0	0	0	1	0	1	0	0	1	0	0	0	0	0	0	0	1	0	0	0	0	0	2
**5.076**	0	1	0	0	0	0	0	0	1	1	0	1	1	0	0	0	0	0	0	0	0	0	0	1	0	2
**5.077**	0	1	0	0	0	0	0	0	1	1	0	0	0	0	0	0	0	0	0	1	0	0	0	1	1	2
**5.078**	0	1	0	0	0	0	0	0	0	1	0	1	1	0	0	0	0	0	0	1	0	1	1	0	0	2
**5.079**	0	1	0	0	0	0	0	0	0	0	0	1	1	0	0	0	0	0	0	1	0	1	1	0	1	2
**5.080**	0	1	0	0	0	0	0	0	0	0	0	1	1	0	0	0	0	0	0	1	0	1	0	0	1	2
**5.081**	0	0	1	1	1	0	1	0	1	1	0	1	0	1	0	0	0	0	0	1	0	0	0	1	1	2
**5.082**	0	0	1	1	0	0	0	0	0	0	0	0	0	0	0	0	0	0	0	1	0	0	0	1	1	2
**5.083**	0	0	1	0	1	0	1	0	1	1	0	1	1	0	0	0	0	0	0	1	0	0	0	0	0	2
**5.084**	0	0	1	0	1	0	0	0	1	1	0	1	1	0	0	0	0	0	0	1	0	0	1	0	0	2
**5.085**	0	0	1	0	1	0	0	0	0	1	0	1	0	1	0	0	0	0	0	0	0	0	0	0	0	2
**5.086**	0	0	1	0	1	0	0	0	0	1	0	1	0	0	0	0	0	0	0	1	0	0	1	0	0	2
**5.087**	0	0	1	0	1	0	0	0	0	0	0	0	0	0	0	0	0	0	0	1	0	0	1	0	0	2
**5.088**	0	0	1	0	0	0	1	0	1	1	0	1	0	0	0	0	0	0	0	1	0	0	0	0	0	2
**5.089**	0	0	1	0	0	0	1	0	1	0	0	1	0	0	0	0	0	0	0	1	0	1	0	0	0	2
**5.090**	0	0	1	0	0	0	1	0	0	1	0	0	0	0	0	0	0	0	0	1	0	0	1	1	0	2
**5.091**	0	0	1	0	0	0	0	0	1	1	0	1	0	1	0	0	0	0	0	1	0	0	0	0	0	2
**5.092**	0	0	1	0	0	0	0	0	1	0	0	1	0	0	0	0	0	0	0	0	0	1	1	0	0	2
**5.093**	0	0	1	0	0	0	0	0	1	0	0	0	0	1	0	0	0	0	0	0	0	1	0	1	1	2
**5.094**	0	0	1	0	0	0	0	0	0	1	0	1	1	0	0	0	0	0	0	0	0	0	1	0	0	2
**5.095**	0	0	1	0	0	0	0	0	0	1	0	1	0	0	0	0	0	0	0	0	0	0	1	1	1	2
**5.096**	0	0	1	0	0	0	0	0	0	1	0	0	1	0	0	0	0	0	0	1	0	0	0	1	1	2
**5.097**	0	0	1	0	0	0	0	0	0	0	0	1	0	0	0	1	0	0	0	0	0	0	1	1	1	2
**5.098**	0	0	1	0	0	0	0	0	0	0	0	0	1	1	0	1	0	0	0	1	0	0	0	0	1	2
**5.099**	0	0	0	1	1	0	1	0	1	1	0	1	0	1	0	0	0	0	0	1	0	0	0	1	0	2
**5.100**	0	0	0	1	1	0	0	0	0	1	0	1	0	1	0	0	0	0	0	1	0	1	0	0	0	2
**5.101**	0	0	0	1	1	0	0	0	0	1	0	1	0	1	0	0	0	0	0	0	0	1	0	0	1	2
**5.102**	0	0	0	1	1	0	0	0	0	0	0	0	0	1	0	0	0	0	0	1	0	0	0	0	0	2
**5.103**	0	0	0	1	1	0	0	0	0	0	0	0	0	0	0	0	0	0	0	0	0	1	0	0	1	2
**5.104**	0	0	0	1	0	0	1	0	0	0	0	0	0	0	0	0	0	0	0	0	0	0	0	1	1	2
**5.105**	0	0	0	1	0	0	0	0	0	1	0	0	0	1	0	0	0	0	0	1	0	1	0	0	0	2
**5.106**	0	0	0	1	0	0	0	0	0	1	0	0	0	0	0	0	0	0	0	0	0	1	0	0	0	2
**5.107**	0	0	0	1	0	0	0	0	0	0	0	1	0	1	0	0	0	0	0	0	0	0	1	0	0	2
**5.108**	0	0	0	1	0	0	0	0	0	0	0	0	0	0	0	0	0	0	0	1	0	1	1	0	0	2
**5.109**	0	0	0	0	1	0	1	0	1	1	0	1	0	1	0	0	0	0	0	1	0	0	0	1	1	2
**5.110**	0	0	0	0	1	0	0	0	0	1	0	0	0	0	0	0	0	0	0	0	0	1	1	0	0	2
**5.111**	0	0	0	0	1	0	0	0	0	0	0	1	1	1	0	0	0	0	0	0	0	0	0	0	0	2
**5.112**	0	0	0	0	1	0	0	0	0	0	0	0	0	1	0	0	0	0	0	1	0	0	0	0	0	2
**5.113**	0	0	0	0	0	0	1	0	1	0	0	1	0	0	0	0	0	0	0	1	0	0	0	0	1	2
**5.114**	0	0	0	0	0	0	1	0	0	1	0	0	1	0	0	1	0	0	0	1	0	1	0	0	0	2
**5.115**	0	0	0	0	0	0	1	0	0	0	0	0	0	0	0	1	0	0	0	0	0	0	1	1	0	2
**5.116**	0	0	0	0	0	0	0	0	0	1	1	1	0	1	0	0	0	0	0	1	0	1	1	0	1	2
**5.117**	0	0	0	0	0	0	0	0	0	1	0	0	1	1	0	0	0	0	0	0	0	0	1	1	0	2
**5.118**	0	0	0	0	0	0	0	0	0	0	0	1	1	0	0	0	0	0	0	1	0	0	1	0	0	2
**5.119**	0	0	0	0	0	0	0	0	0	0	0	0	1	1	0	1	0	0	0	1	0	0	0	0	1	2
**5.120**	0	1	1	1	1	0	0	0	0	1	0	1	1	1	0	0	0	0	0	1	0	0	0	0	1	2
**5.121**	0	1	1	1	1	0	0	0	0	0	0	0	0	0	0	0	0	0	0	1	0	0	0	0	0	2
**5.122**	0	1	1	1	0	0	0	0	1	1	0	1	1	1	0	0	0	0	0	1	0	0	0	0	1	2

CMP	CD2	CD3	CD4	CD7	CD8	CD10	CD13	CD18	CD26	CD29	CD36	CD44	CD45RA	CD49f	CD54	CD56	CD57	CD58	CD62L	CD71	CD80	Cytokeratin	Histone_H1	HLA-DQ	HLA-DR	Freq. (voxel)
**5.123**	0	1	1	1	0	0	0	0	0	0	0	1	1	1	0	0	0	0	0	0	0	0	0	0	0	2
**5.124**	0	1	1	1	0	0	0	0	0	0	0	1	0	1	0	0	0	0	0	1	0	0	1	0	0	2
**5.125**	0	1	1	1	0	0	0	0	0	0	0	1	0	1	0	0	0	0	0	1	0	0	0	0	0	2
**5.126**	0	1	1	1	0	0	0	0	0	0	0	1	0	1	0	0	0	0	0	0	0	0	1	0	0	2
**5.127**	0	1	1	0	1	0	0	0	1	0	0	0	0	0	0	0	0	0	0	1	0	0	0	0	0	2
**5.128**	0	1	1	0	0	0	0	0	1	0	0	1	1	0	0	0	0	0	0	0	0	0	1	0	0	2
**5.129**	0	1	1	0	0	0	0	0	0	0	0	1	1	0	0	0	0	0	0	0	0	0	0	0	0	2
**5.130**	0	1	0	1	1	0	1	0	1	0	0	1	0	0	0	0	0	0	0	0	0	0	0	0	0	2
**5.131**	0	1	0	1	1	0	0	0	0	0	0	1	0	1	0	0	0	0	0	1	0	1	0	0	1	2
**5.132**	0	1	0	1	0	0	1	0	0	1	0	1	0	0	0	0	0	0	0	0	0	0	0	0	1	2
**5.133**	0	1	0	1	0	0	1	0	0	0	0	0	0	0	0	0	0	0	0	0	0	1	1	1	1	2
**5.134**	0	1	0	1	0	0	0	0	1	1	0	0	0	0	0	0	0	0	0	1	0	0	1	0	0	2
**5.135**	0	1	0	1	0	0	0	0	0	1	0	1	1	1	0	0	0	0	0	1	0	1	0	1	0	2
**5.136**	0	1	0	1	0	0	0	0	0	1	0	1	1	0	0	0	0	0	0	1	0	1	0	1	0	2
**5.137**	0	1	0	1	0	0	0	0	0	0	0	1	1	0	0	0	0	0	0	1	0	1	0	1	0	2
**5.138**	0	1	0	1	0	0	0	0	0	0	0	0	0	0	0	0	0	0	0	1	0	0	0	1	1	2
**5.139**	0	1	0	0	1	0	1	0	1	0	0	1	0	0	0	0	0	0	0	1	0	0	0	0	0	2
**5.140**	0	1	0	0	1	0	1	0	0	0	0	1	0	0	0	0	0	0	0	0	0	1	0	0	1	2
**5.141**	0	1	0	0	1	0	0	0	0	1	0	1	0	0	0	1	0	0	0	0	0	0	0	0	0	2
**5.142**	0	1	0	0	1	0	0	0	0	1	0	1	0	0	0	0	0	0	0	0	0	1	1	0	1	2
**5.143**	0	1	0	0	1	0	0	0	0	0	0	1	0	1	0	0	0	0	0	0	0	1	0	0	0	2
**5.144**	0	1	0	0	1	0	0	0	0	0	0	0	0	0	0	0	0	0	0	1	0	0	0	1	0	2
**5.145**	0	1	0	0	0	0	1	0	0	0	0	1	1	0	0	0	0	0	0	0	0	0	0	0	0	2
**5.146**	0	1	0	0	0	0	0	0	1	1	0	1	1	1	0	0	0	0	0	1	0	0	1	0	0	2
**5.147**	0	1	0	0	0	0	0	0	0	1	1	1	0	0	0	0	0	0	0	0	0	0	0	0	0	2
**5.148**	0	1	0	0	0	0	0	0	0	1	0	0	1	0	0	0	0	0	0	1	0	0	1	0	0	2
**5.149**	0	1	0	0	0	0	0	0	0	1	0	0	1	0	0	0	0	0	0	0	0	0	1	0	1	2
**5.150**	0	1	0	0	0	0	0	0	0	1	0	0	0	0	0	0	0	0	0	1	0	0	0	1	1	2
**5.151**	0	1	0	0	0	0	0	0	0	0	0	0	0	1	0	0	0	0	0	1	0	1	1	0	1	2
**5.152**	0	0	1	1	1	0	1	0	0	1	0	1	1	1	0	1	0	0	0	1	0	0	0	1	1	2
**5.153**	0	0	1	1	1	0	0	0	0	1	0	1	1	0	0	0	0	0	0	1	0	0	0	0	0	2
**5.154**	0	0	1	1	0	0	0	0	0	1	0	1	0	0	0	0	0	0	0	1	0	0	1	0	0	2
**5.155**	0	0	1	1	0	0	0	0	0	0	0	0	0	1	0	0	0	0	0	1	0	0	0	1	1	2
**5.156**	0	0	1	1	0	0	0	0	0	0	0	0	0	0	0	0	0	0	0	0	0	0	1	0	0	2
**5.157**	0	0	1	0	0	0	1	0	0	0	0	0	1	1	0	1	0	0	0	1	0	1	0	1	0	2
**5.158**	0	0	1	0	0	0	1	0	0	0	0	0	1	1	0	1	0	0	0	0	0	0	0	1	0	2
**5.159**	0	0	1	0	0	0	1	0	0	0	0	0	0	0	0	0	0	0	0	0	0	1	0	1	0	2
**5.160**	0	0	1	0	0	0	1	0	0	0	0	0	0	0	0	0	0	0	0	0	0	0	1	1	1	2
**5.161**	0	0	1	0	0	0	0	0	1	0	0	1	0	1	0	0	0	0	0	1	0	1	1	0	0	2
**5.162**	0	0	1	0	0	0	0	0	1	0	0	0	0	0	0	0	0	0	0	0	0	0	0	0	1	2
**5.163**	0	0	0	1	1	0	0	0	1	1	0	1	0	0	0	0	0	0	0	1	0	1	0	0	1	2
**5.164**	0	0	0	1	1	0	0	0	0	1	0	0	1	0	0	0	0	0	0	1	0	0	1	0	0	2
**5.165**	0	0	0	1	1	0	0	0	0	0	0	0	1	0	0	0	0	0	0	1	0	0	0	0	0	2
**5.166**	0	0	0	1	0	0	1	0	0	1	0	1	1	1	0	0	0	0	0	0	0	0	0	1	1	2
**5.167**	0	0	0	1	0	0	0	0	1	1	0	1	0	0	0	0	0	0	0	1	0	1	0	0	0	2
**5.168**	0	0	0	1	0	0	0	0	1	0	0	1	0	1	0	0	0	0	0	0	0	1	0	0	0	2
**5.169**	0	0	0	1	0	0	0	0	1	0	0	1	0	0	0	0	0	0	0	1	0	1	1	0	1	2
**5.170**	0	0	0	1	0	0	0	0	0	0	0	0	1	1	0	0	0	0	0	0	0	0	0	1	0	2
**5.171**	0	0	0	1	0	0	0	0	0	0	0	0	0	0	0	0	0	0	0	0	0	1	1	1	1	2
**5.172**	0	0	0	0	1	0	0	0	1	0	0	1	0	0	0	1	0	0	0	1	0	0	0	1	1	2
**5.173**	0	0	0	0	1	0	0	0	0	0	0	1	0	1	0	0	0	0	0	1	0	0	0	0	0	2
**5.174**	0	0	0	0	1	0	0	0	0	0	0	0	1	0	0	0	0	0	0	1	0	0	1	0	1	2
**5.175**	0	0	0	0	1	0	0	0	0	0	0	0	0	1	0	0	0	0	0	0	0	0	1	0	0	2
**5.176**	0	0	0	0	0	0	1	0	1	0	0	0	0	1	0	0	0	0	0	1	0	0	1	1	1	2
**5.177**	0	0	0	0	0	0	1	0	0	1	0	0	1	1	0	1	0	0	0	0	0	0	0	1	0	2
**5.178**	0	0	0	0	0	0	1	0	0	0	0	0	1	1	0	1	0	0	0	1	0	1	0	0	0	2
**5.179**	0	0	0	0	0	0	1	0	0	0	0	0	1	1	0	1	0	0	0	0	0	0	0	1	0	2
**5.180**	0	0	0	0	0	0	0	0	1	0	0	1	1	1	0	0	0	0	0	1	0	1	0	0	1	2
**5.181**	0	0	0	0	0	0	0	0	1	0	0	1	1	0	0	0	0	0	0	1	0	1	0	0	1	2
**5.182**	0	0	0	0	0	0	0	0	0	1	1	1	0	1	0	0	0	0	0	1	0	1	1	0	0	2
**5.183**	0	0	0	0	0	0	0	0	0	1	0	1	1	1	0	1	0	0	0	0	0	1	1	0	0	2
**5.184**	0	0	0	0	0	0	0	0	0	1	0	1	1	1	0	0	0	0	0	1	0	0	0	1	0	2
**5.185**	0	0	0	0	0	0	0	0	0	1	0	1	1	0	0	1	0	0	0	0	0	1	1	0	0	2
**5.186**	0	0	0	0	0	0	0	0	0	1	0	0	1	1	0	0	0	0	0	0	0	0	0	0	0	2
**5.187**	0	0	0	0	0	0	0	0	0	0	1	1	0	1	0	0	0	0	0	0	0	1	0	1	0	2
**5.188**	0	0	0	0	0	0	0	0	0	0	0	0	1	1	0	1	0	0	0	0	0	1	0	1	0	2
**5.189**	0	0	0	0	0	0	0	0	0	0	0	0	0	1	0	1	0	0	0	1	0	1	0	0	0	2
**5.190**	0	1	1	1	1	0	0	0	0	0	0	1	1	0	0	0	0	0	0	1	0	0	1	1	1	2
**5.191**	0	1	1	1	0	0	0	0	1	1	0	1	1	1	0	0	0	0	0	0	0	0	0	0	0	2
**5.192**	0	1	1	0	1	0	0	0	1	0	0	1	0	0	0	0	0	0	0	1	0	0	0	1	0	2
**5.193**	0	1	1	0	1	0	0	0	0	1	0	0	0	0	0	0	0	0	0	1	0	0	0	1	1	2
**5.194**	0	1	1	0	0	0	1	0	0	1	0	1	0	0	0	0	0	0	0	1	0	0	1	0	0	2
**5.195**	0	1	1	0	0	0	0	0	1	1	0	1	1	0	0	0	0	0	0	1	0	0	0	1	0	2
**5.196**	0	1	1	0	0	0	0	0	1	0	0	1	0	0	0	0	0	0	0	1	0	0	0	0	1	2
**5.197**	0	1	1	0	0	0	0	0	0	1	0	1	1	1	0	0	0	0	0	1	0	0	1	0	1	2
**5.198**	0	1	1	0	0	0	0	0	0	1	0	1	1	1	0	0	0	0	0	1	0	0	0	1	1	2
**5.199**	0	1	1	0	0	0	0	0	0	0	0	1	0	1	0	0	0	0	0	0	0	0	0	0	0	2
**5.200**	0	1	1	0	0	0	0	0	0	0	0	0	0	0	0	0	0	0	0	1	0	0	1	0	0	2
**5.201**	0	1	0	1	1	0	0	0	1	1	0	0	0	0	0	0	0	0	0	0	0	0	0	0	0	2
**5.202**	0	1	0	1	1	0	0	0	0	0	0	1	0	1	0	0	0	0	0	1	0	1	0	0	0	2
**5.203**	0	1	0	1	0	0	1	0	0	1	0	1	0	1	0	0	0	0	0	1	0	0	1	0	0	2
**5.204**	0	1	0	1	0	0	1	0	0	1	0	0	0	0	0	0	0	0	0	1	0	0	1	0	0	2
**5.205**	0	1	0	1	0	0	0	0	1	0	0	1	0	1	0	0	0	0	0	0	0	0	1	0	0	2
**5.206**	0	1	0	1	0	0	0	0	1	0	0	1	0	0	0	0	0	0	0	0	0	0	1	1	1	2
**5.207**	0	1	0	1	0	0	0	0	0	1	0	1	1	1	0	0	0	0	0	1	0	0	0	0	1	2
**5.208**	0	1	0	1	0	0	0	0	0	0	0	1	1	1	0	0	0	0	0	1	0	0	0	0	1	2
**5.209**	0	1	0	1	0	0	0	0	0	0	0	1	1	1	0	0	0	0	0	0	0	1	0	1	0	2
**5.210**	0	1	0	1	0	0	0	0	0	0	0	1	1	0	0	0	0	0	0	0	0	0	0	1	1	2
**5.211**	0	1	0	1	0	0	0	0	0	0	0	1	0	0	0	0	0	0	0	1	0	1	1	1	0	2
**5.212**	0	1	0	0	1	0	1	0	1	1	0	1	0	1	0	0	0	0	0	1	0	1	0	1	0	2
**5.213**	0	1	0	0	1	0	1	0	0	1	0	1	0	0	0	0	0	0	0	1	0	0	0	1	1	2
**5.214**	0	1	0	0	1	0	0	0	0	1	0	0	1	0	0	0	0	0	0	0	0	0	1	0	1	2
**5.215**	0	1	0	0	1	0	0	0	0	1	0	0	0	0	0	0	0	0	0	1	0	0	0	1	1	2
**5.216**	0	1	0	0	0	0	1	0	0	1	0	1	1	1	0	0	0	0	0	1	0	0	0	0	0	2
**5.217**	0	1	0	0	0	0	1	0	0	1	0	0	0	1	0	0	0	0	0	1	0	0	1	0	0	2
**5.218**	0	1	0	0	0	0	0	0	1	1	0	1	1	1	0	0	0	0	0	1	0	0	0	0	1	2
**5.219**	0	1	0	0	0	0	0	0	1	0	0	1	0	0	0	0	0	0	0	0	0	1	1	0	1	2
**5.220**	0	1	0	0	0	0	0	0	0	1	0	1	1	1	0	0	0	0	0	1	0	0	1	1	0	2
**5.221**	0	1	0	0	0	0	0	0	0	1	0	1	0	1	0	0	0	0	0	0	0	1	1	0	1	2
**5.222**	0	0	1	1	0	0	1	0	0	1	0	1	0	1	0	0	0	0	0	1	0	1	0	0	1	2
**5.223**	0	0	1	1	0	0	0	0	0	0	0	0	1	0	0	0	0	0	0	1	0	1	0	0	0	2
**5.224**	0	0	1	0	1	0	0	0	0	0	0	0	1	0	0	1	0	0	0	1	0	1	0	1	0	2
**5.225**	0	0	1	0	0	0	1	0	1	0	0	1	0	0	0	0	0	0	0	0	0	1	0	0	0	2
**5.226**	0	0	1	0	0	0	1	0	0	0	0	1	0	0	0	0	0	0	0	1	0	0	0	1	0	2
**5.227**	0	0	1	0	0	0	1	0	0	0	0	0	0	1	0	0	0	0	0	0	0	0	0	0	1	2
**5.228**	0	0	1	0	0	0	0	0	1	1	0	1	0	1	0	0	0	0	0	1	0	0	0	1	0	2
**5.229**	0	0	1	0	0	0	0	0	0	0	0	0	0	0	0	1	0	0	0	0	0	1	1	0	0	2
**5.230**	0	0	0	1	1	0	1	0	0	1	0	1	0	1	0	0	0	0	0	0	0	1	0	1	1	2
**5.231**	0	0	0	1	1	0	0	0	0	0	0	1	0	1	0	0	0	0	0	1	0	0	0	0	0	2
**5.232**	0	0	0	1	1	0	0	0	0	0	0	0	1	0	0	0	0	0	0	0	0	0	1	0	0	2
**5.233**	0	0	0	1	0	0	0	0	1	0	0	1	0	1	0	0	0	0	0	1	0	1	0	0	0	2
**5.234**	0	0	0	1	0	0	0	0	0	0	0	1	0	0	0	0	0	0	0	1	0	1	1	0	1	2
**5.235**	0	0	0	1	0	0	0	0	0	0	0	0	1	1	0	0	0	0	0	1	0	1	0	0	0	2
**5.236**	0	0	0	0	0	0	1	0	1	1	0	0	1	1	0	0	0	0	0	1	0	1	0	0	0	2
**5.237**	0	0	0	0	0	0	1	0	1	0	0	1	0	0	0	0	0	0	0	1	0	0	1	1	1	2
**5.238**	0	0	0	0	0	0	1	0	1	0	0	0	0	0	0	0	0	0	0	1	0	0	1	1	1	2
**5.239**	0	0	0	0	0	0	1	0	0	1	0	1	0	0	0	0	0	0	0	0	0	0	1	0	1	2
**5.240**	0	0	0	0	0	0	1	0	0	1	0	0	1	1	0	0	0	0	0	0	0	0	1	0	0	2
**5.241**	0	0	0	0	0	0	1	0	0	1	0	0	0	0	0	0	0	0	0	1	0	0	1	1	1	2
**5.242**	0	0	0	0	0	0	1	0	0	0	1	0	1	0	0	0	0	0	0	0	0	1	0	0	0	2
**5.243**	0	0	0	0	0	0	1	0	0	0	0	0	1	0	0	0	0	0	0	0	0	1	1	0	0	2
**5.244**	0	0	0	0	0	0	0	0	1	0	1	1	0	1	0	0	0	0	0	1	0	1	0	1	1	2

CMP	CD2	CD3	CD4	CD7	CD8	CD10	CD13	CD18	CD26	CD29	CD36	CD44	CD45RA	CD49f	CD54	CD56	CD57	CD58	CD62L	CD71	CD80	Cytokeratin	Histone_H1	HLA-DQ	HLA-DR	Freq. (voxel)
**5.245**	0	0	0	0	0	0	0	0	1	0	0	1	0	1	0	1	0	0	0	1	0	1	0	1	1	2
**5.246**	0	0	0	0	0	0	0	0	0	1	1	1	0	1	0	0	0	0	0	0	0	1	1	0	0	2
**5.247**	0	0	0	0	0	0	0	0	0	1	1	1	0	0	0	0	0	0	0	1	0	0	0	0	0	2
**5.248**	0	0	0	0	0	0	0	0	0	1	0	1	1	0	0	1	0	0	0	0	0	0	0	0	0	2
**5.249**	0	0	0	0	0	0	0	0	0	1	0	0	0	0	0	1	0	0	0	0	0	1	0	1	0	2
**5.250**	0	0	0	0	0	0	0	0	0	0	0	1	1	0	0	0	0	0	0	1	0	0	0	1	0	2
**5.251**	0	0	0	0	0	0	0	0	0	0	0	1	0	0	0	1	0	0	0	0	0	1	0	1	1	2
**5.252**	0	1	1	1	1	0	0	0	0	0	0	1	1	0	0	0	0	0	0	0	0	0	1	1	1	2
**5.253**	0	1	1	1	0	0	0	0	1	0	0	1	0	0	0	1	0	0	0	1	0	1	0	0	1	2
**5.254**	0	1	1	1	0	0	0	0	1	0	0	1	0	0	0	0	0	0	0	1	0	0	0	1	0	2
**5.255**	0	1	1	1	0	0	0	0	0	1	0	1	1	1	0	1	0	0	0	0	0	0	0	0	0	2
**5.256**	0	1	1	0	1	0	0	0	0	0	0	1	0	1	0	0	0	0	0	1	0	0	0	1	1	2
**5.257**	0	1	1	0	1	0	0	0	0	0	0	1	0	0	0	0	0	0	0	1	0	0	1	1	1	2
**5.258**	0	1	1	0	0	0	0	0	0	1	0	1	0	1	0	0	0	0	0	0	0	0	0	1	1	2
**5.259**	0	1	1	0	0	0	0	0	0	0	0	1	1	0	0	0	0	0	0	0	0	0	1	0	0	2
**5.260**	0	1	1	0	0	0	0	0	0	0	0	1	0	0	0	0	0	0	0	1	0	1	0	0	0	2
**5.261**	0	1	1	0	0	0	0	0	0	0	0	1	0	0	0	0	0	0	0	1	0	0	1	0	1	2
**5.262**	0	1	1	0	0	0	0	0	0	0	0	0	0	0	0	0	0	0	0	0	0	0	1	0	1	2
**5.263**	0	1	1	0	0	0	0	0	0	0	0	0	0	0	0	0	0	0	0	0	0	0	0	0	1	2
**5.264**	0	1	0	1	1	0	0	0	1	0	0	0	1	0	0	0	0	0	0	0	0	0	1	0	0	2
**5.265**	0	1	0	1	1	0	0	0	0	1	0	1	1	1	0	0	0	0	0	0	0	1	1	0	1	2
**5.266**	0	1	0	1	1	0	0	0	0	1	0	1	0	1	0	0	0	0	0	1	0	0	0	1	0	2
**5.267**	0	1	0	1	0	0	0	0	1	1	0	1	1	1	0	0	0	0	0	1	0	0	0	1	0	2
**5.268**	0	1	0	1	0	0	0	0	0	1	0	1	1	0	0	0	0	0	0	1	0	0	0	1	0	2
**5.269**	0	1	0	1	0	0	0	0	0	0	0	1	1	0	0	0	0	0	0	0	0	1	0	1	0	2
**5.270**	0	1	0	1	0	0	0	0	0	0	0	0	1	0	0	0	0	0	0	0	0	1	0	1	0	2
**5.271**	0	1	0	1	0	0	0	0	0	0	0	0	0	1	0	0	0	0	0	1	0	0	0	1	1	2
**5.272**	0	1	0	0	1	0	1	0	0	1	0	1	0	1	0	0	0	0	0	1	0	1	1	1	1	2
**5.273**	0	1	0	0	1	0	1	0	0	1	0	1	0	1	0	0	0	0	0	0	0	1	1	0	1	2
**5.274**	0	1	0	0	1	0	0	0	0	1	0	1	1	1	0	0	0	0	0	0	0	1	1	0	1	2
**5.275**	0	1	0	0	1	0	0	0	0	0	0	0	0	1	0	0	0	0	0	1	0	0	0	1	1	2
**5.276**	0	1	0	0	0	0	1	0	0	0	0	1	0	0	0	0	0	0	0	1	0	0	1	0	1	2
**5.277**	0	1	0	0	0	0	0	0	1	1	0	1	1	1	0	0	0	0	0	0	0	0	0	1	0	2
**5.278**	0	1	0	0	0	0	0	0	1	1	0	1	0	1	0	0	0	0	0	0	0	0	0	1	0	2
**5.279**	0	1	0	0	0	0	0	0	1	0	0	1	0	0	0	1	0	0	0	0	0	0	0	1	1	2
**5.280**	0	1	0	0	0	0	0	0	1	0	0	0	1	0	0	0	0	0	0	0	0	0	0	0	1	2
**5.281**	0	1	0	0	0	0	0	0	0	0	0	0	0	1	0	0	0	0	0	1	0	1	1	1	0	2
**5.282**	0	1	0	0	0	0	0	0	0	0	0	0	0	1	0	0	0	0	0	1	0	1	0	1	0	2
**5.283**	0	0	1	1	0	0	1	0	1	0	0	1	0	1	0	0	0	0	0	1	0	1	0	0	1	2
**5.284**	0	0	1	1	0	0	0	0	0	0	0	1	1	1	0	0	0	0	0	1	0	1	0	1	1	2
**5.285**	0	0	1	1	0	0	0	0	0	0	0	0	1	1	0	1	0	0	0	0	0	1	0	1	0	2
**5.286**	0	0	1	1	0	0	0	0	0	0	0	0	1	1	0	0	0	0	0	0	0	1	0	1	0	2
**5.287**	0	0	1	1	0	0	0	0	0	0	0	0	1	1	0	0	0	0	0	0	0	0	0	1	0	2
**5.288**	0	0	1	0	1	0	1	0	1	0	0	0	0	0	0	0	0	0	0	0	0	0	0	1	1	2
**5.289**	0	0	1	0	1	0	0	0	0	1	0	1	1	0	0	0	0	0	0	0	0	0	0	1	0	2
**5.290**	0	0	1	0	1	0	0	0	0	0	0	0	1	0	0	0	0	0	0	0	0	0	0	1	0	2
**5.291**	0	0	1	0	0	0	1	0	1	1	0	1	0	1	0	0	0	0	0	0	0	0	0	0	0	2
**5.292**	0	0	1	0	0	0	1	0	0	1	0	1	0	1	0	1	0	0	0	0	0	0	1	1	1	2
**5.293**	0	0	1	0	0	0	1	0	0	1	0	0	0	1	0	0	0	0	0	0	0	1	1	1	0	2
**5.294**	0	0	1	0	0	0	1	0	0	0	0	1	1	1	0	0	0	0	0	1	0	0	0	1	1	2
**5.295**	0	0	1	0	0	0	1	0	0	0	0	1	0	1	0	1	0	0	0	1	0	0	0	1	1	2
**5.296**	0	0	1	0	0	0	0	0	1	1	0	1	1	0	0	0	0	0	0	0	0	0	1	0	0	2
**5.297**	0	0	1	0	0	0	0	0	1	0	0	1	0	0	0	0	0	0	0	0	0	0	1	1	0	2
**5.298**	0	0	1	0	0	0	0	0	0	1	0	0	0	0	0	1	0	0	0	0	0	0	0	1	1	2
**5.299**	0	0	0	1	1	0	1	0	0	0	0	0	0	0	0	0	0	0	0	0	0	0	1	0	0	2
**5.300**	0	0	0	1	0	0	0	0	0	1	1	0	0	1	0	0	0	0	0	1	0	1	0	0	0	2
**5.301**	0	0	0	0	0	0	1	0	0	1	0	1	1	1	0	0	0	0	0	0	0	0	0	0	1	2
**5.302**	0	0	0	0	0	0	1	0	0	0	0	0	0	0	0	1	0	0	0	0	0	0	1	0	0	2
**5.303**	0	0	0	0	0	0	0	0	1	1	0	1	1	0	0	0	0	0	0	0	0	1	0	0	0	2
**5.304**	0	0	0	0	0	0	0	0	1	1	0	1	0	1	0	0	0	0	0	1	0	1	1	1	1	2
**5.305**	0	0	0	0	0	0	0	0	1	1	0	0	0	1	0	0	0	0	0	0	0	1	0	0	1	2
**5.306**	0	0	0	0	0	0	0	0	0	1	1	1	1	0	0	0	0	0	0	0	0	0	0	0	1	2
**5.307**	0	0	0	0	0	0	0	0	0	1	0	1	0	0	0	1	0	0	0	1	0	1	1	0	0	2
**5.308**	0	1	1	0	1	0	1	0	0	1	0	1	0	1	0	0	0	0	0	0	0	1	0	1	1	2
**5.309**	0	1	1	0	1	0	0	0	0	1	0	1	1	0	0	0	0	0	0	1	0	0	0	1	0	2
**5.310**	0	1	1	0	1	0	0	0	0	1	0	1	1	0	0	0	0	0	0	0	0	0	0	1	0	2
**5.311**	0	1	1	0	1	0	0	0	0	1	0	1	0	0	0	0	0	0	0	0	0	0	1	1	1	2
**5.312**	0	1	1	0	1	0	0	0	0	1	0	1	0	0	0	0	0	0	0	0	0	0	1	0	1	2
**5.313**	0	1	1	0	1	0	0	0	0	0	0	1	1	0	0	0	0	0	0	0	0	0	1	0	0	2
**5.314**	0	1	1	0	1	0	0	0	0	0	0	1	0	0	0	0	0	0	0	0	0	0	1	1	1	2
**5.315**	0	1	1	0	0	0	1	0	0	0	0	1	0	0	0	0	0	0	0	1	0	0	0	0	0	2
**5.316**	0	1	1	0	0	0	0	0	0	0	0	0	1	0	0	0	0	0	0	0	0	0	1	0	0	2
**5.317**	0	1	0	1	1	0	0	0	0	1	0	1	0	1	0	0	0	0	0	1	0	0	1	1	1	2
**5.318**	0	1	0	1	1	0	0	0	0	0	0	1	1	1	0	0	0	0	0	0	0	0	0	0	1	2
**5.319**	0	1	0	1	1	0	0	0	0	0	0	1	0	0	0	1	0	0	0	1	0	1	0	0	1	2
**5.320**	0	1	0	1	0	0	1	0	0	1	0	0	0	0	0	0	0	0	0	0	0	0	1	0	0	2
**5.321**	0	1	0	1	0	0	0	0	0	1	0	1	1	0	0	0	0	0	0	0	0	0	0	1	0	2
**5.322**	0	1	0	1	0	0	0	0	0	0	0	1	0	1	0	0	0	0	0	0	0	0	0	1	0	2
**5.323**	0	1	0	0	1	0	1	0	0	1	0	1	1	1	0	0	0	0	0	0	0	0	0	0	0	2
**5.324**	0	1	0	0	1	0	0	0	1	0	0	1	0	0	0	1	0	0	0	1	0	1	0	0	1	2
**5.325**	0	1	0	0	1	0	0	0	1	0	0	0	1	0	0	0	0	0	0	0	0	1	0	0	1	2
**5.326**	0	1	0	0	1	0	0	0	1	0	0	0	1	0	0	0	0	0	0	0	0	0	1	1	1	2
**5.327**	0	1	0	0	1	0	0	0	0	1	0	1	0	1	0	0	0	0	0	1	0	0	1	0	1	2
**5.328**	0	1	0	0	0	0	1	0	1	0	0	1	0	1	0	0	0	0	0	0	0	0	0	0	0	2
**5.329**	0	1	0	0	0	0	0	0	0	1	0	1	1	1	0	0	0	0	0	1	0	0	0	1	0	2
**5.330**	0	1	0	0	0	0	0	0	0	1	0	0	0	0	0	0	0	0	0	0	0	0	1	1	0	2
**5.331**	0	1	0	0	0	0	0	0	0	0	1	1	0	1	0	0	0	0	0	1	0	0	0	1	0	2
**5.332**	0	1	0	0	0	0	0	0	0	0	1	0	0	1	0	0	0	0	0	1	0	0	0	1	0	2
**5.333**	0	1	0	0	0	0	0	0	0	0	0	1	0	1	0	0	0	0	0	1	0	0	1	1	1	2
**5.334**	0	0	1	0	1	0	0	0	1	0	0	0	0	0	0	0	0	0	0	1	0	0	0	1	1	2
**5.335**	0	0	1	0	1	0	0	0	0	0	0	1	0	1	0	0	0	0	0	0	0	0	0	0	0	2
**5.336**	0	0	1	0	1	0	0	0	0	0	0	0	0	0	0	0	0	0	0	0	0	0	0	1	0	2
**5.337**	0	0	1	0	0	0	1	0	1	0	1	0	0	1	0	0	0	0	0	1	0	1	0	0	0	2
**5.338**	0	0	1	0	0	0	1	0	1	0	0	0	0	1	0	0	0	0	0	1	0	1	1	0	0	2
**5.339**	0	0	1	0	0	0	1	0	0	1	0	1	1	1	0	0	0	0	0	1	0	0	1	1	1	2
**5.340**	0	0	1	0	0	0	1	0	0	1	0	0	1	1	0	0	0	0	0	1	0	1	0	0	1	2
**5.341**	0	0	1	0	0	0	1	0	0	0	1	1	0	1	0	0	0	0	0	0	0	1	0	1	1	2
**5.342**	0	0	1	0	0	0	1	0	0	0	0	0	1	1	0	0	0	0	0	0	0	0	0	1	1	2
**5.343**	0	0	1	0	0	0	0	0	1	0	0	0	1	0	0	1	0	0	0	0	0	0	0	1	0	2
**5.344**	0	0	1	0	0	0	0	0	0	1	1	0	0	1	0	0	0	0	0	0	0	1	0	1	0	2
**5.345**	0	0	1	0	0	0	0	0	0	1	0	0	1	0	0	1	0	0	0	0	0	1	0	1	0	2
**5.346**	0	0	1	0	0	0	0	0	0	1	0	0	1	0	0	0	0	0	0	0	0	1	0	0	0	2
**5.347**	0	0	1	0	0	0	0	0	0	1	0	0	0	0	0	1	0	0	0	1	0	0	0	0	0	2
**5.348**	0	0	1	0	0	0	0	0	0	0	0	1	1	1	0	0	0	0	0	1	0	1	0	0	1	2
**5.349**	0	0	0	1	0	0	1	0	0	0	1	0	0	1	0	0	0	0	0	1	0	1	0	0	0	2
**5.350**	0	0	0	0	1	0	0	0	1	1	0	0	0	0	0	0	0	0	0	0	0	0	0	0	1	2
**5.351**	0	0	0	0	0	0	1	0	1	0	0	0	1	0	0	0	0	0	0	0	0	0	1	1	0	2
**5.352**	0	0	0	0	0	0	1	0	0	1	0	1	1	1	0	0	0	0	0	1	0	1	1	1	0	2
**5.353**	0	0	0	0	0	0	1	0	0	1	0	1	0	1	0	1	0	0	0	0	0	0	1	1	0	2
**5.354**	0	0	0	0	0	0	1	0	0	0	0	1	1	1	0	0	0	0	0	1	0	1	0	0	1	2
**5.355**	0	0	0	0	0	0	1	0	0	0	0	1	1	0	0	0	0	0	0	1	0	0	0	0	0	2
**5.356**	0	0	0	0	0	0	0	0	0	1	0	0	0	1	0	0	0	0	0	1	0	1	0	1	0	2
**5.357**	0	0	0	0	0	0	0	0	0	0	0	0	0	1	0	1	0	0	0	0	0	0	0	0	1	2
**5.358**	0	1	1	1	1	0	0	0	0	0	0	0	1	0	0	0	0	0	0	0	0	0	0	1	0	2
**5.359**	0	1	1	0	1	0	0	0	1	0	0	0	0	0	0	0	0	0	0	0	0	0	1	0	0	2
**5.360**	0	1	1	0	0	0	0	0	1	1	0	1	0	0	0	0	0	0	0	1	0	0	0	0	1	2
**5.361**	0	1	0	1	1	0	0	0	0	1	1	1	1	1	0	0	0	0	0	0	0	0	1	1	1	2
**5.362**	0	1	0	1	1	0	0	0	0	0	0	0	0	0	0	0	0	0	0	0	0	1	1	0	1	2
**5.363**	0	1	0	1	0	0	0	0	1	1	0	1	0	0	0	0	0	0	0	0	0	1	0	0	0	2
**5.364**	0	1	0	1	0	0	0	0	1	0	0	1	0	0	0	0	0	0	0	1	0	0	1	0	1	2
**5.365**	0	1	0	0	1	0	0	0	1	1	0	0	1	0	0	0	0	0	0	0	0	0	1	1	0	2
**5.366**	0	1	0	0	1	0	0	0	0	1	1	1	0	0	0	0	0	0	0	0	0	0	0	1	1	2

CMP	CD2	CD3	CD4	CD7	CD8	CD10	CD13	CD18	CD26	CD29	CD36	CD44	CD45RA	CD49f	CD54	CD56	CD57	CD58	CD62L	CD71	CD80	Cytokeratin	Histone_H1	HLA-DQ	HLA-DR	Freq. (voxel)
5.367	0	1	0	0	1	0	0	0	0	0	0	0	0	0	0	0	0	0	0	1	0	0	1	1	0	2
5.368	0	1	0	0	0	0	0	0	1	1	0	1	0	1	0	0	0	0	0	1	0	1	0	0	1	2
5.369	0	1	0	0	0	0	0	0	0	1	0	1	0	1	0	0	0	0	0	0	0	1	1	0	0	2
5.370	0	1	0	0	0	0	0	0	0	0	0	1	1	1	0	0	0	0	0	0	0	0	0	1	0	2
5.371	0	1	0	0	0	0	0	0	0	0	0	1	0	1	0	0	0	0	0	1	0	0	0	0	1	2
5.372	0	1	0	0	0	0	0	0	0	0	0	1	0	1	0	0	0	0	0	0	0	0	1	1	0	2
5.373	0	0	1	1	0	0	1	0	1	1	1	1	0	1	0	0	0	0	0	0	0	0	0	1	1	2
5.374	0	0	1	1	0	0	0	0	0	0	0	1	0	0	0	0	0	0	0	1	0	0	0	0	0	2
5.375	0	0	1	0	0	0	1	0	0	0	1	1	0	1	0	0	0	0	0	0	0	1	0	0	1	2
5.376	0	0	1	0	0	0	1	0	0	0	0	0	1	1	0	0	0	0	0	0	0	1	0	0	0	2
5.377	0	0	1	0	0	0	0	0	1	0	0	1	0	1	0	0	0	0	0	0	0	1	0	0	1	2
5.378	0	0	1	0	0	0	0	0	0	0	1	1	0	1	0	0	0	0	0	0	0	1	0	1	0	2
5.379	0	0	1	0	0	0	0	0	0	0	1	0	0	1	0	0	0	0	0	0	0	1	0	1	0	2
5.380	0	0	0	1	0	0	1	0	0	0	0	0	0	0	0	0	0	0	0	1	0	0	1	0	0	2
5.381	0	0	0	1	0	0	0	0	0	1	0	1	0	1	0	0	0	0	0	0	0	0	0	0	1	2
5.382	0	0	0	0	1	0	1	0	0	0	0	0	1	0	0	0	0	0	0	0	0	0	0	1	0	2
5.383	0	0	0	0	1	0	0	0	1	0	0	1	1	0	0	0	0	0	0	0	0	0	0	1	0	2
5.384	0	0	0	0	1	0	0	0	1	0	0	1	0	0	0	1	0	0	0	0	0	0	0	1	1	2
5.385	0	0	0	0	1	0	0	0	1	0	0	1	0	0	0	0	0	0	0	0	0	1	1	1	1	2
5.386	0	0	0	0	1	0	0	0	0	1	0	0	0	0	0	0	0	0	0	1	0	0	1	1	1	2
5.387	0	0	0	0	0	0	1	0	1	1	1	1	0	0	0	0	0	0	0	1	0	0	0	1	1	2
5.388	0	0	0	0	0	0	1	0	1	1	0	1	1	1	0	0	0	0	0	1	0	0	0	1	1	2
5.389	0	0	0	0	0	0	1	0	0	1	1	1	1	1	0	0	0	0	0	1	0	0	0	1	1	2
5.390	0	0	0	0	0	0	1	0	0	0	0	0	0	1	0	1	0	0	0	0	0	1	0	0	1	2
5.391	0	0	0	0	0	0	0	0	1	1	1	1	0	1	0	0	0	0	0	1	0	0	0	1	1	2
5.392	0	0	0	0	0	0	0	0	0	1	0	1	0	1	0	1	0	0	0	1	0	1	0	0	1	2
5.393	0	0	0	0	0	0	0	0	0	0	1	0	0	1	0	0	0	0	0	1	0	1	0	1	0	2
5.394	0	0	0	0	0	0	0	0	0	0	0	1	0	1	0	1	0	0	0	1	0	1	0	0	1	2
5.395	0	1	1	1	1	0	0	0	1	0	0	1	0	0	0	0	0	0	0	1	0	1	0	0	1	2
5.396	0	1	1	0	1	0	0	0	1	0	0	1	0	0	0	0	0	0	0	0	0	1	0	0	0	2
5.397	0	1	1	0	1	0	0	0	1	0	0	0	1	0	0	0	0	0	0	1	0	0	1	0	0	2
5.398	0	1	1	0	0	0	0	0	0	1	0	1	0	1	0	0	0	0	0	0	0	0	0	0	0	2
5.399	0	1	0	1	1	0	0	0	1	0	0	1	1	1	0	0	0	0	0	1	0	0	0	0	1	2
5.400	0	1	0	1	0	0	0	0	1	0	0	1	1	0	0	0	0	0	0	1	0	0	0	0	1	2
5.401	0	1	0	1	0	0	0	0	0	1	0	1	0	1	0	0	0	0	0	1	0	0	1	1	1	2
5.402	0	1	0	0	1	0	0	0	1	1	1	1	0	0	0	0	0	0	0	0	0	0	0	1	0	2
5.403	0	1	0	0	1	0	0	0	1	0	0	0	1	0	0	0	0	0	0	1	0	0	1	0	1	2
5.404	0	1	0	0	1	0	0	0	0	1	0	1	0	1	0	0	0	0	0	0	0	0	0	1	0	2
5.405	0	1	0	0	0	0	0	0	0	1	0	0	0	0	0	0	0	0	0	0	0	0	1	0	1	2
5.406	0	1	0	0	0	0	0	0	0	0	1	0	0	1	0	0	0	0	0	0	0	0	0	0	0	2
5.407	0	1	0	0	0	0	0	0	0	0	0	0	0	1	0	0	0	0	0	0	0	1	1	1	0	2
5.408	0	0	1	1	0	0	0	0	0	1	0	1	0	1	0	0	0	0	0	0	0	1	0	0	1	2
5.409	0	0	1	0	0	0	1	0	1	1	0	1	0	1	0	1	0	0	0	0	0	0	1	1	1	2
5.410	0	0	1	0	0	0	1	0	0	1	0	1	0	1	0	1	0	0	0	1	0	1	0	1	0	2
5.411	0	0	1	0	0	0	1	0	0	0	1	0	0	1	0	0	0	0	0	1	0	1	0	0	0	2
5.412	0	0	1	0	0	0	0	0	0	1	0	1	0	1	0	0	0	0	0	0	0	1	1	0	1	2
5.413	0	0	1	0	0	0	0	0	0	0	0	1	1	1	0	0	0	0	0	1	0	1	0	0	0	2
5.414	0	0	0	0	1	0	1	0	1	0	0	0	0	0	0	0	0	0	0	1	0	0	0	0	0	2
5.415	0	0	0	0	0	0	1	0	1	0	0	1	0	0	0	1	0	0	0	1	0	0	0	1	1	2
5.416	0	0	0	0	0	0	1	0	0	0	0	1	0	1	0	1	0	0	0	0	0	0	0	1	0	2
5.417	0	0	0	0	0	0	1	0	0	0	0	1	0	1	0	0	0	0	0	1	0	1	1	1	1	2
5.418	0	0	0	0	0	0	1	0	0	0	0	1	0	1	0	0	0	0	0	1	0	0	1	0	1	2
5.419	0	0	0	0	0	0	0	0	1	1	0	0	0	1	0	0	0	0	0	0	0	0	0	1	0	2
5.420	0	0	0	0	0	0	0	0	1	0	0	1	0	0	0	1	0	0	0	1	0	1	0	0	1	2
5.421	0	0	0	0	0	0	0	0	1	0	0	0	1	1	0	0	0	0	0	0	0	0	0	1	0	2
5.422	0	0	0	0	0	0	0	0	1	0	0	0	1	0	0	0	0	0	0	0	0	0	1	1	0	2
5.423	0	0	0	0	0	0	0	0	1	0	0	0	0	1	0	0	0	0	0	1	0	1	1	0	1	2
5.424	0	0	0	0	0	0	0	0	0	1	1	1	0	1	0	1	0	0	0	0	0	0	0	1	1	2
5.425	0	0	0	0	0	0	0	0	0	1	0	1	1	1	0	0	0	0	0	0	0	1	0	0	1	2
5.426	0	0	0	0	0	0	0	0	0	1	0	1	1	0	0	0	0	0	0	0	0	0	0	0	1	2
5.427	0	0	0	0	0	0	0	0	0	0	0	1	0	1	0	1	0	0	0	0	0	1	0	0	1	2
5.428	0	0	0	0	0	0	0	0	0	0	0	0	1	1	0	0	0	0	0	0	0	0	1	1	0	2
5.429	0	1	1	1	1	0	0	0	0	0	0	0	0	0	0	0	0	0	0	0	0	0	0	1	0	2
5.430	0	1	1	1	0	0	0	0	0	0	0	1	1	0	0	0	0	0	0	1	0	0	0	0	0	2
5.431	0	1	1	0	1	0	0	0	1	1	0	0	0	0	0	0	0	0	0	1	0	0	0	1	0	2
5.432	0	1	1	0	1	0	0	0	1	0	0	1	1	0	0	0	0	0	0	1	0	1	0	0	0	2
5.433	0	1	0	1	1	0	0	0	1	0	0	0	0	0	0	0	0	0	0	0	0	1	0	0	1	2
5.434	0	1	0	1	1	0	0	0	0	0	0	0	0	0	0	0	0	0	0	0	0	0	0	1	0	2
5.435	0	1	0	1	0	0	0	0	1	1	0	1	1	0	0	0	0	0	0	0	0	0	0	1	0	2
5.436	0	1	0	1	0	0	0	0	1	0	0	0	1	0	0	0	0	0	0	1	0	0	1	0	0	2
5.437	0	1	0	1	0	0	0	0	0	0	0	0	0	1	0	0	0	0	0	1	0	0	1	0	0	2
5.438	0	1	0	0	1	0	0	0	1	1	1	1	0	0	0	0	0	0	0	0	0	0	1	1	1	2
5.439	0	1	0	0	1	0	0	0	1	0	0	0	1	0	0	0	0	0	0	1	0	0	0	0	1	2
5.440	0	1	0	0	0	0	0	0	1	1	1	1	0	0	0	0	0	0	0	0	0	0	0	1	0	2
5.441	0	1	0	0	0	0	0	0	1	0	1	1	0	0	0	0	0	0	0	0	0	0	1	1	0	2
5.442	0	0	1	1	0	0	1	0	1	1	0	1	1	1	0	0	0	0	0	1	0	0	1	0	1	2
5.443	0	0	1	1	0	0	0	0	1	0	0	0	0	0	0	0	0	0	0	0	0	0	0	0	0	2
5.444	0	0	1	1	0	0	0	0	0	0	1	0	0	0	0	0	0	0	0	0	0	1	0	0	0	2
5.445	0	0	1	0	0	0	1	0	1	1	0	1	0	1	0	0	0	0	0	1	0	0	1	0	1	2
5.446	0	0	1	0	0	0	1	0	1	0	0	0	1	0	0	0	0	0	0	1	0	0	0	1	0	2
5.447	0	0	1	0	0	0	1	0	0	1	0	1	1	1	0	0	0	0	0	0	0	1	0	0	1	2
5.448	0	0	1	0	0	0	1	0	0	1	0	1	0	1	0	0	0	0	0	1	0	0	1	0	0	2
5.449	0	0	1	0	0	0	1	0	0	0	0	1	0	1	0	0	0	0	0	1	0	1	1	0	1	2
5.450	0	0	1	0	0	0	0	0	1	1	0	0	0	1	0	0	0	0	0	0	0	0	0	1	1	2
5.451	0	0	1	0	0	0	0	0	0	1	0	1	1	1	0	0	0	0	0	1	0	1	0	0	1	2
5.452	0	0	1	0	0	0	0	0	0	1	0	1	0	1	0	1	0	0	0	0	0	1	0	1	1	2
5.453	0	0	0	1	0	0	0	0	0	0	0	0	0	0	0	0	0	0	0	1	0	1	1	0	1	2
5.454	0	0	0	0	1	0	1	0	0	1	0	1	1	0	0	0	0	0	0	1	0	0	0	0	0	2
5.455	0	0	0	0	0	0	1	0	1	1	0	0	0	0	0	0	0	0	0	1	0	0	1	1	0	2
5.456	0	0	0	0	0	0	1	0	0	0	0	0	1	0	0	0	0	0	0	0	0	0	0	1	1	2
5.457	0	0	0	0	0	0	0	0	1	1	0	1	1	1	0	0	0	0	0	0	0	1	0	0	1	2
5.458	0	0	0	0	0	0	0	0	1	0	0	1	1	1	0	0	0	0	0	1	0	1	0	0	0	2
5.459	0	0	0	0	0	0	0	0	0	1	1	1	0	0	0	0	0	0	0	0	0	0	1	1	1	2
5.460	0	1	1	0	0	0	1	0	0	0	0	1	0	0	0	0	0	0	0	0	0	0	0	0	0	2
5.461	0	1	0	1	1	0	0	0	0	1	1	1	0	0	0	0	0	0	0	0	0	0	0	0	0	2
5.462	0	1	0	1	0	0	0	0	1	0	0	1	1	0	0	0	0	0	0	1	0	0	0	1	0	2
5.463	0	1	0	1	0	0	0	0	1	0	0	1	1	0	0	0	0	0	0	0	0	0	0	1	0	2
5.464	0	1	0	1	0	0	0	0	1	0	0	0	1	0	0	0	0	0	0	1	0	0	0	0	0	2
5.465	0	1	0	1	0	0	0	0	1	0	0	0	1	0	0	0	0	0	0	0	0	0	0	1	0	2
5.466	0	1	0	1	0	0	0	0	0	1	0	1	0	0	0	0	0	0	0	1	0	0	1	1	0	2
5.467	0	1	0	1	0	0	0	0	0	1	0	1	0	0	0	0	0	0	0	1	0	0	1	0	1	2
5.468	0	1	0	1	0	0	0	0	0	0	0	1	0	1	0	0	0	0	0	0	0	1	0	0	1	2
5.469	0	1	0	0	1	0	0	0	0	1	1	1	0	0	0	0	0	0	0	0	0	0	1	0	0	2
5.470	0	1	0	0	1	0	0	0	0	0	0	1	1	1	0	0	0	0	0	1	0	0	1	0	0	2
5.471	0	1	0	0	0	0	1	0	0	0	0	0	0	0	0	0	0	0	0	0	0	0	0	0	1	2
5.472	0	1	0	0	0	0	0	0	1	0	0	1	0	1	0	1	0	0	0	0	0	0	0	0	0	2
5.473	0	1	0	0	0	0	0	0	0	0	1	0	0	0	0	0	0	0	0	0	0	1	0	0	1	2
5.474	0	0	1	0	0	0	1	0	1	1	1	1	1	1	0	1	0	0	0	0	0	0	0	1	1	2
5.475	0	0	1	0	0	0	1	0	0	0	1	0	0	1	0	0	0	0	0	0	0	1	0	0	1	2
5.476	0	0	1	0	0	0	1	0	0	0	1	0	0	0	0	0	0	0	0	0	0	0	0	1	0	2
5.477	0	0	1	0	0	0	1	0	0	0	0	1	0	1	0	0	0	0	0	1	0	1	1	1	0	2
5.478	0	0	1	0	0	0	0	0	1	1	0	1	0	1	0	0	0	0	0	1	0	0	0	0	1	2
5.479	0	0	1	0	0	0	0	0	1	1	0	0	0	1	0	0	0	0	0	1	0	0	0	1	0	2
5.480	0	0	1	0	0	0	0	0	1	0	0	0	1	0	0	0	0	0	0	0	0	0	0	0	0	2
5.481	0	0	1	0	0	0	0	0	0	0	1	1	0	1	0	0	0	0	0	0	0	1	0	0	0	2
5.482	0	0	1	0	0	0	0	0	0	0	0	0	1	1	0	0	0	0	0	0	0	0	0	0	0	2
5.483	0	0	0	1	0	0	1	0	1	0	0	0	0	0	0	0	0	0	0	1	0	0	0	0	0	2
5.484	0	0	0	1	0	0	0	0	1	0	0	1	0	0	0	0	0	0	0	0	0	1	0	0	1	2
5.485	0	0	0	0	0	0	1	0	1	1	1	1	0	1	0	1	0	0	0	1	0	0	0	1	1	2
5.486	0	0	0	0	0	0	1	0	1	1	1	0	0	1	0	0	0	0	0	0	0	0	0	1	1	2
5.487	0	0	0	0	0	0	1	0	1	0	0	0	0	1	0	0	0	0	0	0	0	1	1	1	0	2
5.488	0	0	0	0	0	0	1	0	0	1	0	1	1	1	0	1	0	0	0	1	0	0	1	0	0	2

CMP	CD2	CD3	CD4	CD7	CD8	CD10	CD13	CD18	CD26	CD29	CD36	CD44	CD45RA	CD49f	CD54	CD56	CD57	CD58	CD62L	CD71	CD80	Cytokeratin	Histone_H1	HLA-DQ	HLA-DR	Freq. (voxel)
**5.489**	0	0	0	0	0	0	1	0	0	0	1	0	0	0	0	1	0	0	0	0	0	1	0	0	0	2
**5.490**	0	0	0	0	0	0	1	0	0	0	0	1	0	1	0	0	0	0	0	1	0	1	0	1	0	2
**5.491**	0	0	0	0	0	0	0	0	1	0	1	1	0	1	0	0	0	0	0	0	0	0	0	1	1	2
**5.492**	0	0	0	0	0	0	0	0	0	1	1	1	0	0	0	0	0	0	0	0	0	0	1	1	0	2
**5.493**	0	0	0	0	0	0	0	0	0	0	1	1	0	0	0	0	0	0	0	1	0	0	0	1	0	2
**5.494**	0	1	1	0	0	0	0	0	0	1	0	0	0	0	0	0	0	0	0	0	0	0	1	0	0	2
**5.495**	0	1	0	1	1	0	1	0	1	0	0	1	0	0	0	0	0	0	0	1	0	0	1	0	1	2
**5.496**	0	1	0	1	1	0	1	0	1	0	0	1	0	0	0	0	0	0	0	1	0	0	0	0	0	2
**5.497**	0	1	0	1	1	0	0	0	1	1	0	1	1	0	0	0	0	0	0	0	0	1	0	1	1	2
**5.498**	0	1	0	0	1	0	0	0	0	1	0	1	1	0	0	0	0	0	0	0	0	1	0	0	1	2
**5.499**	0	1	0	0	1	0	0	0	0	0	1	1	0	0	0	0	0	0	0	0	0	0	1	1	0	2
**5.500**	0	1	0	0	1	0	0	0	0	0	1	1	0	0	0	0	0	0	0	0	0	0	1	0	0	2
**5.501**	0	1	0	0	0	0	0	0	0	1	1	1	0	0	0	0	0	0	0	0	0	0	0	0	1	2
**5.502**	0	1	0	0	0	0	0	0	0	0	0	1	0	0	0	1	0	0	0	0	0	1	1	0	0	2
**5.503**	0	1	0	0	0	0	0	0	0	0	0	0	0	0	0	1	0	0	0	0	0	1	1	0	0	2
**5.504**	0	0	1	1	0	0	1	0	0	0	0	1	0	1	0	0	0	0	0	1	0	1	0	0	0	2
**5.505**	0	0	1	1	0	0	0	0	0	1	0	0	0	1	0	0	0	0	0	0	0	0	0	0	0	2
**5.506**	0	0	1	0	0	0	1	0	1	0	0	1	0	1	0	0	0	0	0	1	0	1	1	0	0	2
**5.507**	0	0	1	0	0	0	1	0	0	1	0	1	0	1	0	0	0	0	0	1	0	0	1	1	0	2
**5.508**	0	0	1	0	0	0	0	0	1	1	0	1	0	1	0	1	0	0	0	0	0	0	0	1	1	2
**5.509**	0	0	1	0	0	0	0	0	0	1	1	1	0	1	0	1	0	0	0	0	0	0	0	1	1	2
**5.510**	0	0	1	0	0	0	0	0	0	1	0	1	0	1	0	0	0	0	0	0	0	1	1	1	1	2
**5.511**	0	0	1	0	0	0	0	0	0	0	0	0	0	0	0	0	0	0	0	1	0	1	1	0	1	2
**5.512**	0	0	0	1	0	0	1	0	0	1	0	0	0	1	0	0	0	0	0	1	0	0	0	1	1	2
**5.513**	0	0	0	1	0	0	0	0	0	1	0	0	0	1	0	0	0	0	0	0	0	1	1	0	0	2
**5.514**	0	0	0	1	0	0	0	0	0	1	0	0	0	1	0	0	0	0	0	0	0	0	1	1	1	2
**5.515**	0	0	0	0	0	0	1	0	1	1	0	0	0	1	0	0	0	0	0	1	0	1	0	0	1	2
**5.516**	0	0	0	0	0	0	1	0	1	0	0	0	0	1	0	0	0	0	0	1	0	1	1	1	0	2
**5.517**	0	0	0	0	0	0	1	0	0	0	0	1	1	0	0	0	0	0	0	0	0	0	0	0	1	2
**5.518**	0	0	0	0	0	0	1	0	0	0	0	0	0	1	0	0	0	0	0	1	0	1	1	1	0	2
**5.519**	0	0	0	0	0	0	0	0	1	1	0	1	0	1	0	1	0	0	0	0	0	0	1	1	1	2
**5.520**	0	0	0	0	0	0	0	0	1	1	0	1	0	1	0	0	0	0	0	1	0	0	1	1	1	2
**5.521**	0	0	0	0	0	0	0	0	1	1	0	0	0	1	0	0	0	0	0	1	0	0	0	1	0	2
**5.522**	0	0	0	0	0	0	0	0	1	1	0	0	0	1	0	0	0	0	0	0	0	0	1	1	1	2
**5.523**	0	0	0	0	0	0	0	0	1	0	1	0	0	1	0	0	0	0	0	0	0	0	0	1	1	2
**5.524**	0	0	0	0	0	0	0	0	1	0	0	1	0	1	0	0	0	0	0	1	0	0	1	1	1	2
**5.525**	0	0	0	0	0	0	0	0	0	1	1	1	0	1	0	0	0	0	0	0	0	0	1	1	1	2
**5.526**	0	0	0	0	0	0	0	0	0	1	0	1	1	1	0	0	0	0	0	0	0	1	0	1	1	2
**5.527**	0	0	0	0	0	0	0	0	0	0	1	1	1	0	0	0	0	0	0	0	0	1	0	0	0	2
**5.528**	0	0	0	0	0	0	0	0	0	0	0	0	0	1	0	0	0	0	0	1	0	0	1	0	1	2
**5.529**	0	1	0	1	0	0	0	0	0	0	0	1	0	0	0	0	0	0	0	1	0	1	0	0	1	2
**5.530**	0	1	0	0	1	0	1	0	0	1	0	1	0	1	0	0	0	0	0	1	0	1	1	0	0	2
**5.531**	0	1	0	0	1	0	0	0	1	0	0	0	0	0	0	0	0	0	0	0	0	1	1	0	0	2
**5.532**	0	0	1	0	0	0	1	0	0	0	0	1	0	0	0	0	0	0	0	0	0	1	0	0	1	2
**5.533**	0	0	1	0	0	0	0	0	1	1	0	1	0	1	0	0	0	0	0	0	0	1	1	0	1	2
**5.534**	0	0	1	0	0	0	0	0	0	1	1	1	0	1	0	0	0	0	0	0	0	0	0	1	0	2
**5.535**	0	0	1	0	0	0	0	0	0	1	0	1	0	1	0	1	0	0	0	0	0	0	0	1	1	2
**5.536**	0	0	1	0	0	0	0	0	0	1	0	1	0	0	0	1	0	0	0	0	0	1	0	0	0	2
**5.537**	0	0	0	1	1	0	0	0	0	0	0	1	0	1	0	0	0	0	0	0	0	0	1	0	0	2
**5.538**	0	0	0	0	1	0	1	0	0	0	0	0	0	0	0	0	0	0	0	1	0	0	1	0	0	2
**5.539**	0	0	0	0	0	0	1	0	0	1	1	0	0	1	0	0	0	0	0	1	0	0	0	1	1	2
**5.540**	0	0	0	0	0	0	1	0	0	0	1	1	0	1	0	0	0	0	0	0	0	0	1	0	0	2
**5.541**	0	0	0	0	0	0	1	0	0	0	1	0	0	1	0	0	0	0	0	1	0	1	0	0	1	2
**5.542**	0	0	0	0	0	0	0	0	1	1	1	1	0	1	0	0	0	0	0	0	0	0	1	0	1	2
**5.543**	0	0	0	0	0	0	0	0	1	0	1	0	0	0	0	0	0	0	0	0	0	0	0	1	1	2
**5.544**	0	0	0	0	0	0	0	0	1	0	0	0	0	1	0	1	0	0	0	0	0	0	0	1	1	2
**5.545**	0	0	0	0	0	0	0	0	1	0	0	0	0	1	0	0	0	0	0	0	0	0	1	1	1	2
**5.546**	0	0	0	0	0	0	0	0	0	1	0	1	0	1	0	1	0	0	0	0	0	1	1	1	0	2
**5.547**	0	0	0	0	0	0	0	0	0	1	0	0	1	0	0	1	0	0	0	1	0	0	0	1	0	2
**5.548**	0	0	0	0	0	0	0	0	0	0	1	1	1	0	0	0	0	0	0	1	0	1	0	0	0	2
**5.549**	0	0	0	0	0	0	0	0	0	0	1	0	0	0	0	0	0	0	0	1	0	0	1	0	0	2
**5.550**	0	0	0	0	0	0	0	0	0	0	0	1	0	1	0	0	0	0	0	1	0	0	1	1	1	2
**5.551**	0	1	0	0	0	0	0	0	1	1	0	1	1	0	0	0	0	0	0	1	0	0	0	0	1	2
**5.552**	0	1	0	0	0	0	0	0	1	0	0	0	0	0	0	0	0	0	0	1	0	1	0	0	1	2
**5.553**	0	1	0	0	0	0	0	0	0	0	0	0	1	0	0	0	0	0	0	0	0	0	1	1	1	2
**5.554**	0	0	1	0	0	0	1	0	0	1	1	0	0	0	0	0	0	0	0	0	0	0	0	1	1	2
**5.555**	0	0	1	0	0	0	0	0	0	1	1	0	0	0	0	0	0	0	0	1	0	0	0	1	1	2
**5.556**	0	0	1	0	0	0	0	0	0	0	1	0	0	0	0	0	0	0	0	1	0	0	0	1	1	2
**5.557**	0	0	0	1	0	0	1	0	0	1	0	0	1	1	0	0	0	0	0	1	0	0	0	1	1	2
**5.558**	0	0	0	0	1	0	1	0	0	1	0	1	0	0	0	0	0	0	0	0	0	1	1	0	0	2
**5.559**	0	0	0	0	1	0	1	0	0	0	0	1	1	0	0	0	0	0	0	1	0	0	0	0	0	2
**5.560**	0	0	0	0	1	0	1	0	0	0	0	1	0	1	0	0	0	0	0	0	0	1	0	0	1	2
**5.561**	0	0	0	0	1	0	1	0	0	0	0	1	0	1	0	0	0	0	0	0	0	1	0	0	0	2
**5.562**	0	0	0	0	1	0	0	0	0	0	0	0	1	0	0	1	0	0	0	0	0	0	0	1	0	2
**5.563**	0	0	0	0	0	0	0	0	1	1	0	1	0	1	0	0	0	0	0	0	0	0	1	1	0	2
**5.564**	0	0	0	0	0	0	0	0	0	0	0	0	1	1	0	0	0	0	0	0	0	1	1	0	0	2
**5.565**	0	1	0	1	1	0	1	0	0	0	0	1	1	0	0	0	0	0	0	0	0	0	0	0	0	2
**5.566**	0	1	0	1	1	0	0	0	1	0	0	1	0	0	0	1	0	0	0	1	0	0	0	0	0	2
**5.567**	0	1	0	1	1	0	0	0	1	0	0	1	0	0	0	1	0	0	0	0	0	0	0	0	0	2
**5.568**	0	1	0	0	1	0	1	0	0	0	1	1	0	0	0	0	0	0	0	0	0	0	0	0	1	2
**5.569**	0	1	0	0	0	0	0	0	0	0	0	0	1	1	0	0	0	0	0	0	0	0	1	0	0	2
**5.570**	0	0	1	1	0	0	0	0	0	1	0	0	0	0	0	0	0	0	0	0	0	0	0	1	1	2
**5.571**	0	0	1	1	0	0	0	0	0	1	0	0	0	0	0	0	0	0	0	0	0	0	0	1	0	2
**5.572**	0	0	0	1	1	0	0	0	1	0	0	0	0	0	0	0	0	0	0	0	0	0	1	0	1	2
**5.573**	0	0	0	1	0	0	0	0	0	1	0	0	0	1	0	0	0	0	0	1	0	0	0	1	1	2
**5.574**	0	0	0	0	1	0	1	0	0	0	0	1	0	0	0	0	0	0	0	0	0	1	1	0	0	2
**5.575**	0	0	0	0	0	0	0	0	1	0	0	0	0	0	0	1	0	0	0	1	0	0	0	1	0	2
**5.576**	0	1	0	1	1	0	1	0	0	1	0	1	0	0	0	0	0	0	0	0	0	0	1	0	0	2
**5.577**	0	1	0	1	1	0	1	0	0	0	0	1	1	0	0	0	0	0	0	0	0	0	1	0	0	2
**5.578**	0	1	0	1	1	0	0	0	0	0	0	0	0	0	0	0	0	0	0	0	0	1	0	0	0	2
**5.579**	0	1	0	0	1	0	1	0	0	0	0	1	1	1	0	0	0	0	0	1	0	0	0	0	0	2
**5.580**	0	1	0	0	0	0	1	0	0	1	0	1	0	0	0	0	0	0	0	0	0	1	0	0	0	2
**5.581**	0	0	1	1	0	0	0	0	0	1	0	0	0	0	0	0	0	0	0	0	0	0	0	0	0	2
**5.582**	0	0	0	0	1	0	1	0	0	0	0	1	1	0	0	0	0	0	0	0	0	0	1	0	0	2
**5.583**	0	0	0	0	1	0	1	0	0	0	0	0	1	0	0	1	0	0	0	1	0	0	0	0	0	2
**5.584**	0	0	0	0	1	0	1	0	0	0	0	0	0	0	0	0	0	0	0	0	0	1	0	0	0	2
**5.585**	0	0	0	0	0	0	1	0	1	0	0	0	0	1	0	0	0	0	0	1	0	0	1	0	0	2
**5.586**	0	1	1	0	0	0	0	0	0	0	0	0	0	0	0	0	0	0	0	0	0	1	0	0	1	2
**5.587**	0	1	0	0	1	0	1	0	0	1	0	1	1	0	0	0	0	0	0	0	0	1	0	0	0	2
**5.588**	0	1	0	0	1	0	1	0	0	1	0	1	0	0	0	0	0	0	0	0	0	1	0	0	1	2
**5.589**	0	1	0	0	1	0	0	0	1	0	0	0	0	0	0	0	0	0	0	1	0	0	0	1	0	2
**5.590**	0	0	1	0	0	0	0	0	0	0	0	1	0	0	0	1	0	0	0	0	0	0	0	0	1	2
**5.591**	0	0	1	0	0	0	0	0	0	0	0	1	0	0	0	1	0	0	0	0	0	0	0	0	0	2
**5.592**	0	0	0	0	1	0	1	0	0	1	1	1	0	0	0	0	0	0	0	0	0	0	0	0	0	2
**5.593**	0	0	0	0	0	0	1	0	1	0	0	0	1	0	0	0	0	0	0	1	0	1	0	1	0	2
**5.594**	0	0	0	0	0	0	1	0	0	1	0	0	1	0	0	0	0	0	0	0	0	1	0	0	0	2
**5.595**	0	0	0	0	0	0	0	0	0	0	0	0	1	1	0	1	0	0	0	1	0	1	0	1	0	2
**5.596**	0	0	0	0	1	0	1	0	1	0	0	0	0	0	0	0	0	0	0	1	0	1	1	0	0	2
**5.597**	0	0	0	0	0	0	1	0	0	1	0	1	1	0	0	0	0	0	0	0	0	1	0	0	0	2
**5.598**	0	0	0	0	0	0	0	0	1	0	0	0	1	0	0	0	0	0	0	0	0	1	0	0	1	2
**5.599**	0	1	0	0	0	0	0	0	0	1	0	1	1	0	0	0	0	0	0	1	0	1	0	0	0	2
**5.600**	0	0	0	0	0	0	0	0	0	0	0	0	0	0	0	1	0	0	0	1	0	0	1	0	0	2
**5.601**	0	1	1	1	1	0	1	0	1	1	0	1	1	1	0	0	0	0	0	1	0	0	0	1	0	1
**5.602**	0	1	1	1	1	0	1	0	1	1	0	1	1	0	0	0	0	0	0	0	0	0	0	0	0	1
**5.603**	0	1	1	1	1	0	1	0	1	1	0	1	0	0	0	0	0	0	0	1	0	1	1	1	1	1
**5.604**	0	1	1	1	1	0	1	0	1	0	0	1	1	1	0	0	0	0	0	1	0	1	0	1	1	1
**5.605**	0	1	1	1	1	0	1	0	1	0	0	1	0	0	0	0	0	0	0	1	0	0	0	1	1	1
**5.606**	0	1	1	1	1	0	0	0	1	1	0	1	0	1	0	0	0	0	0	0	0	0	0	0	1	1
**5.607**	0	1	1	1	1	0	0	0	1	1	0	1	0	1	0	0	0	0	0	0	0	0	0	0	0	1
**5.608**	0	1	1	1	1	0	0	0	1	0	0	1	1	0	0	0	0	0	0	1	0	0	1	1	0	1
**5.609**	0	1	1	1	1	0	0	0	1	0	0	1	1	0	0	0	0	0	0	0	0	0	0	1	1	1
**5.610**	0	1	1	1	1	0	0	0	1	0	0	1	0	1	0	0	0	0	0	1	0	1	0	1	1	1

CMP	CD2	CD3	CD4	CD7	CD8	CD10	CD13	CD18	CD26	CD29	CD36	CD44	CD45RA	CD49f	CD54	CD56	CD57	CD58	CD62L	CD71	CD80	Cytokeratin	Histone_H1	HLA-DQ	HLA-DR	Freq. (voxel)
**5.611**	0	1	1	1	1	0	0	0	0	0	0	1	1	0	0	0	0	0	0	1	0	1	0	0	1	1
**5.612**	0	1	1	1	1	0	0	0	0	0	0	1	0	1	0	0	0	0	0	0	0	0	0	0	0	1
**5.613**	0	1	1	1	0	0	0	0	0	0	0	0	0	1	0	0	0	0	0	1	0	0	0	0	0	1
**5.614**	0	1	1	1	0	0	0	0	0	0	0	0	0	1	0	0	0	0	0	0	0	0	0	0	0	1
**5.615**	0	1	1	0	1	0	1	0	1	1	0	1	1	1	0	0	0	0	0	1	0	0	0	1	0	1
**5.616**	0	1	1	0	1	0	1	0	1	1	0	1	0	0	0	0	0	0	0	1	0	0	1	1	0	1
**5.617**	0	1	1	0	1	0	1	0	1	0	0	1	0	1	0	0	0	0	0	1	0	1	0	1	1	1
**5.618**	0	1	1	0	1	0	1	0	1	0	0	1	0	1	0	0	0	0	0	0	0	1	0	1	1	1
**5.619**	0	1	1	0	1	0	0	0	1	1	0	1	1	0	0	0	0	0	0	0	0	0	0	0	1	1
**5.620**	0	1	1	0	1	0	0	0	1	1	0	1	0	0	0	0	0	0	0	1	0	1	1	1	1	1
**5.621**	0	1	1	0	1	0	0	0	1	0	0	1	1	0	0	0	0	0	0	1	0	0	1	1	0	1
**5.622**	0	1	1	0	1	0	0	0	1	0	0	1	0	1	0	0	0	0	0	1	0	0	0	0	0	1
**5.623**	0	1	1	0	1	0	0	0	1	0	0	0	0	0	0	0	0	0	0	1	0	1	1	1	1	1
**5.624**	0	1	1	0	1	0	0	0	1	0	0	0	0	0	0	0	0	0	0	0	0	0	0	0	0	1
**5.625**	0	1	1	0	1	0	0	0	0	0	0	1	0	0	0	0	0	0	0	0	0	1	0	1	1	1
**5.626**	0	1	1	0	0	0	1	0	1	1	0	1	0	0	0	0	0	0	0	1	0	1	0	1	1	1
**5.627**	0	1	1	0	0	0	1	0	1	1	0	1	0	0	0	0	0	0	0	0	0	0	0	0	0	1
**5.628**	0	1	1	0	0	0	1	0	1	0	0	1	1	0	0	0	0	0	0	1	0	1	0	1	1	1
**5.629**	0	1	1	0	0	0	0	0	1	1	0	1	0	0	0	0	0	0	0	0	0	1	0	1	0	1
**5.630**	0	1	1	0	0	0	0	0	1	0	0	0	0	0	0	0	0	0	0	0	0	0	1	0	1	1
**5.631**	0	1	0	1	1	0	1	0	1	1	0	1	1	1	0	0	0	0	0	1	0	0	0	0	0	1
**5.632**	0	1	0	1	1	0	1	0	1	1	0	1	1	0	0	0	0	0	0	0	0	0	0	0	0	1
**5.633**	0	1	0	1	1	0	1	0	1	1	0	1	0	0	0	0	0	0	0	1	0	0	0	0	1	1
**5.634**	0	1	0	1	1	0	1	0	1	0	0	1	1	1	0	0	0	0	0	1	0	1	0	1	1	1
**5.635**	0	1	0	1	1	0	1	0	1	0	0	1	1	0	0	0	0	0	0	0	0	0	0	1	1	1
**5.636**	0	1	0	1	1	0	0	0	1	1	0	1	1	0	0	0	0	0	0	0	0	1	0	1	0	1
**5.637**	0	1	0	1	1	0	0	0	1	1	0	1	0	1	0	0	0	0	0	1	0	1	0	0	0	1
**5.638**	0	1	0	1	1	0	0	0	1	0	0	1	0	0	0	0	0	0	0	0	0	1	1	1	1	1
**5.639**	0	1	0	1	1	0	0	0	1	0	0	0	0	0	0	0	0	0	0	1	0	1	1	1	0	1
**5.640**	0	1	0	1	1	0	0	0	0	1	0	1	1	1	0	0	0	0	0	0	0	0	1	0	0	1
**5.641**	0	1	0	1	1	0	0	0	0	1	0	1	1	0	0	0	0	0	0	0	0	1	1	0	0	1
**5.642**	0	1	0	1	1	0	0	0	0	0	0	1	1	1	0	0	0	0	0	0	0	0	0	1	1	1
**5.643**	0	1	0	1	1	0	0	0	0	0	0	0	1	0	0	0	0	0	0	1	0	0	0	1	0	1
**5.644**	0	1	0	1	0	0	0	0	1	1	0	1	0	0	0	0	0	0	0	1	0	1	0	1	1	1
**5.645**	0	1	0	1	0	0	0	0	1	1	0	1	0	0	0	0	0	0	0	0	0	1	0	0	1	1
**5.646**	0	1	0	1	0	0	0	0	1	0	0	1	1	0	0	0	0	0	0	1	0	1	1	0	0	1
**5.647**	0	1	0	1	0	0	0	0	1	0	0	1	1	0	0	0	0	0	0	0	0	1	1	0	0	1
**5.648**	0	1	0	1	0	0	0	0	1	0	0	1	1	0	0	0	0	0	0	0	0	1	0	0	0	1
**5.649**	0	1	0	1	0	0	0	0	1	0	0	1	1	0	0	0	0	0	0	0	0	0	1	1	1	1
**5.650**	0	1	0	1	0	0	0	0	1	0	0	1	1	0	0	0	0	0	0	0	0	0	1	0	1	1
**5.651**	0	1	0	1	0	0	0	0	1	0	0	1	0	0	0	0	0	0	0	0	0	1	1	1	0	1
**5.652**	0	1	0	1	0	0	0	0	1	0	0	0	0	0	0	0	0	0	0	0	0	0	1	1	0	1
**5.653**	0	1	0	1	0	0	0	0	0	1	0	1	0	0	0	0	0	0	0	0	0	1	1	1	0	1
**5.654**	0	1	0	0	1	0	1	0	1	0	0	1	0	0	0	0	0	0	0	0	0	0	0	0	0	1
**5.655**	0	1	0	0	1	0	1	0	0	0	0	1	0	0	0	0	0	0	0	1	0	0	0	1	0	1
**5.656**	0	1	0	0	1	0	0	0	1	1	0	1	1	1	0	0	0	0	0	1	0	1	0	1	1	1
**5.657**	0	1	0	0	1	0	0	0	1	1	0	1	0	1	0	0	0	0	0	1	0	1	0	0	0	1
**5.658**	0	1	0	0	1	0	0	0	1	1	0	1	0	0	0	0	0	0	0	1	0	1	1	1	1	1
**5.659**	0	1	0	0	1	0	0	0	1	1	0	0	0	0	0	0	0	0	0	1	0	0	1	0	1	1
**5.660**	0	1	0	0	1	0	0	0	1	1	0	0	0	0	0	0	0	0	0	1	0	0	0	0	0	1
**5.661**	0	1	0	0	1	0	0	0	0	0	0	1	0	1	0	0	0	0	0	0	0	1	1	0	1	1
**5.662**	0	1	0	0	0	0	1	0	1	0	0	1	0	0	0	0	0	0	0	1	0	1	1	1	1	1
**5.663**	0	1	0	0	0	0	1	0	1	0	0	1	0	0	0	0	0	0	0	0	0	1	1	1	1	1
**5.664**	0	1	0	0	0	0	0	0	1	1	0	1	0	0	0	0	0	0	0	1	0	1	1	1	0	1
**5.665**	0	1	0	0	0	0	0	0	1	1	0	1	0	0	0	0	0	0	0	1	0	1	1	0	1	1
**5.666**	0	1	0	0	0	0	0	0	1	1	0	1	0	0	0	0	0	0	0	1	0	0	1	1	0	1
**5.667**	0	1	0	0	0	0	0	0	1	0	0	0	1	0	0	0	0	0	0	0	0	0	1	1	0	1
**5.668**	0	1	0	0	0	0	0	0	0	1	0	1	0	0	0	0	0	0	0	1	0	1	0	1	0	1
**5.669**	0	0	1	1	1	0	1	0	1	0	0	1	0	0	0	1	0	0	0	1	0	0	0	1	1	1
**5.670**	0	0	1	1	1	0	1	0	1	0	0	1	0	0	0	0	0	0	0	1	0	0	0	1	1	1
**5.671**	0	0	1	1	1	0	1	0	1	0	0	1	0	0	0	0	0	0	0	0	0	0	0	1	1	1
**5.672**	0	0	1	1	1	0	0	0	1	1	0	1	0	0	0	0	0	0	0	1	0	0	1	0	0	1
**5.673**	0	0	1	1	1	0	0	0	1	1	0	1	0	0	0	0	0	0	0	1	0	0	0	1	1	1
**5.674**	0	0	1	1	1	0	0	0	0	1	0	1	0	1	0	0	0	0	0	1	0	0	0	0	0	1
**5.675**	0	0	1	1	1	0	0	0	0	1	0	1	0	0	0	0	0	0	0	1	0	0	0	1	1	1
**5.676**	0	0	1	1	1	0	0	0	0	1	0	1	0	0	0	0	0	0	0	0	0	0	0	1	1	1
**5.677**	0	0	1	1	0	0	1	0	1	0	0	0	0	0	0	0	0	0	0	0	0	0	0	0	0	1
**5.678**	0	0	1	1	0	0	1	0	0	1	0	1	0	0	0	0	0	0	0	1	0	0	0	1	0	1
**5.679**	0	0	1	1	0	0	1	0	0	1	0	1	0	0	0	0	0	0	0	0	0	0	0	1	0	1
**5.680**	0	0	1	1	0	0	1	0	0	0	0	1	1	0	0	0	0	0	0	1	0	1	0	0	0	1
**5.681**	0	0	1	1	0	0	1	0	0	0	0	1	0	0	0	1	0	0	0	0	0	0	0	1	1	1
**5.682**	0	0	1	1	0	0	1	0	0	0	0	0	1	0	0	0	0	0	0	1	0	1	0	0	0	1
**5.683**	0	0	1	1	0	0	0	0	1	0	0	1	1	0	0	0	0	0	0	1	0	0	0	1	0	1
**5.684**	0	0	1	1	0	0	0	0	1	0	0	1	1	0	0	0	0	0	0	0	0	0	0	0	0	1
**5.685**	0	0	1	1	0	0	0	0	1	0	0	1	0	1	0	0	0	0	0	0	0	1	0	1	0	1
**5.686**	0	0	1	1	0	0	0	0	1	0	0	1	0	0	0	0	0	0	0	1	0	0	0	1	0	1
**5.687**	0	0	1	1	0	0	0	0	1	0	0	1	0	0	0	0	0	0	0	1	0	0	0	0	0	1
**5.688**	0	0	1	1	0	0	0	0	1	0	0	1	0	0	0	0	0	0	0	0	0	0	0	1	0	1
**5.689**	0	0	1	1	0	0	0	0	1	0	0	0	1	0	0	0	0	0	0	0	0	1	0	0	0	1
**5.690**	0	0	1	1	0	0	0	0	1	0	0	0	1	0	0	0	0	0	0	0	0	0	0	1	0	1
**5.691**	0	0	1	1	0	0	0	0	0	1	0	0	0	0	0	0	0	0	0	1	0	0	0	1	0	1
**5.692**	0	0	1	1	0	0	0	0	0	0	0	1	0	1	0	0	0	0	0	1	0	0	0	0	0	1
**5.693**	0	0	1	0	1	0	1	0	1	1	0	1	0	0	0	0	0	0	0	1	0	0	1	0	0	1
**5.694**	0	0	1	0	1	0	1	0	1	0	0	1	0	0	0	0	0	0	0	1	0	1	0	1	1	1
**5.695**	0	0	1	0	1	0	0	0	1	0	0	1	0	0	0	0	0	0	0	1	0	0	0	0	1	1
**5.696**	0	0	1	0	1	0	0	0	0	1	0	1	1	0	0	0	0	0	0	1	0	0	1	0	0	1
**5.697**	0	0	1	0	1	0	0	0	0	1	0	1	1	0	0	0	0	0	0	1	0	0	0	1	1	1
**5.698**	0	0	1	0	1	0	0	0	0	1	0	1	0	0	0	0	0	0	0	0	0	0	0	0	1	1
**5.699**	0	0	1	0	1	0	0	0	0	0	0	1	0	1	0	0	0	0	0	1	0	0	0	0	0	1
**5.700**	0	0	1	0	0	0	1	0	1	1	0	1	0	0	0	0	0	0	0	1	0	1	0	1	1	1
**5.701**	0	0	1	0	0	0	1	0	1	1	0	0	0	0	0	0	0	0	0	0	0	0	0	1	1	1
**5.702**	0	0	1	0	0	0	1	0	1	0	0	0	0	0	0	0	0	0	0	0	0	0	1	1	1	1
**5.703**	0	0	1	0	0	0	1	0	0	1	0	0	0	0	0	0	0	0	0	1	0	0	0	1	0	1
**5.704**	0	0	1	0	0	0	1	0	0	0	0	1	0	0	0	0	0	0	0	0	0	1	0	1	0	1
**5.705**	0	0	1	0	0	0	1	0	0	0	0	0	0	0	0	0	0	0	0	0	0	1	0	1	1	1
**5.706**	0	0	1	0	0	0	0	0	1	0	0	0	0	1	0	0	0	0	0	0	0	1	0	1	0	1
**5.707**	0	0	1	0	0	0	0	0	1	0	0	0	0	0	0	0	0	0	0	1	0	1	1	1	1	1
**5.708**	0	0	1	0	0	0	0	0	0	1	0	1	0	0	0	1	0	0	0	1	0	1	0	1	0	1
**5.709**	0	0	1	0	0	0	0	0	0	0	0	1	1	1	0	0	0	0	0	0	0	0	0	1	1	1
**5.710**	0	0	1	0	0	0	0	0	0	0	0	1	0	1	0	0	0	0	0	0	0	0	0	0	1	1
**5.711**	0	0	1	0	0	0	0	0	0	0	0	0	0	0	0	0	0	0	0	1	0	1	1	1	1	1
**5.712**	0	0	1	0	0	0	0	0	0	0	0	0	0	0	0	0	0	0	0	1	0	1	1	1	0	1
**5.713**	0	0	0	1	1	0	1	0	1	1	0	1	0	0	0	0	0	0	0	0	0	0	0	0	1	1
**5.714**	0	0	0	1	1	0	1	0	1	0	0	1	0	0	0	0	0	0	0	1	0	0	0	1	1	1
**5.715**	0	0	0	1	1	0	1	0	1	0	0	1	0	0	0	0	0	0	0	0	0	0	0	0	0	1
**5.716**	0	0	0	1	1	0	1	0	0	1	0	1	0	0	0	0	0	0	0	1	0	1	0	1	1	1
**5.717**	0	0	0	1	1	0	1	0	0	1	0	1	0	0	0	0	0	0	0	1	0	0	0	1	1	1
**5.718**	0	0	0	1	1	0	1	0	0	1	0	1	0	0	0	0	0	0	0	0	0	1	0	1	1	1
**5.719**	0	0	0	1	1	0	1	0	0	0	0	1	0	0	0	1	0	0	0	1	0	0	0	1	1	1
**5.720**	0	0	0	1	1	0	0	0	1	1	0	1	1	0	0	0	0	0	0	0	0	0	1	0	0	1
**5.721**	0	0	0	1	1	0	0	0	1	1	0	1	1	0	0	0	0	0	0	0	0	0	0	1	0	1
**5.722**	0	0	0	1	1	0	0	0	1	1	0	1	0	1	0	0	0	0	0	1	0	1	0	0	0	1
**5.723**	0	0	0	1	1	0	0	0	1	1	0	1	0	1	0	0	0	0	0	0	0	0	0	0	0	1
**5.724**	0	0	0	1	1	0	0	0	1	0	0	1	1	0	0	0	0	0	0	1	0	1	1	1	1	1
**5.725**	0	0	0	1	1	0	0	0	1	0	0	1	0	0	0	0	0	0	0	1	0	0	0	0	1	1
**5.726**	0	0	0	1	1	0	0	0	1	0	0	1	0	0	0	0	0	0	0	0	0	1	1	1	1	1
**5.727**	0	0	0	1	1	0	0	0	1	0	0	1	0	0	0	0	0	0	0	0	0	1	0	1	1	1
**5.728**	0	0	0	1	1	0	0	0	1	0	0	0	1	0	0	0	0	0	0	1	0	0	1	1	1	1
**5.729**	0	0	0	1	1	0	0	0	1	0	0	0	1	0	0	0	0	0	0	1	0	0	1	0	1	1
**5.730**	0	0	0	1	1	0	0	0	1	0	0	0	0	0	0	0	0	0	0	1	0	1	1	0	0	1
**5.731**	0	0	0	1	1	0	0	0	1	0	0	0	0	0	0	0	0	0	0	0	0	0	1	0	0	1
**5.732**	0	0	0	1	1	0	0	0	1	0	0	0	0	0	0	0	0	0	0	0	0	0	0	1	0	1

CMP	CD2	CD3	CD4	CD7	CD8	CD10	CD13	CD18	CD26	CD29	CD36	CD44	CD45RA	CD49f	CD54	CD56	CD57	CD58	CD62L	CD71	CD80	Cytokeratin	Histone_H1	HLA-DQ	HLA-DR	Freq. (voxel)
**5.733**	0	0	0	1	1	0	0	0	1	0	0	0	0	0	0	0	0	0	0	0	0	0	0	0	0	1
**5.734**	0	0	0	1	1	0	0	0	0	1	0	1	1	0	0	0	0	0	0	1	0	0	0	0	1	1
**5.735**	0	0	0	1	1	0	0	0	0	1	0	1	0	0	0	0	0	0	0	1	0	1	1	0	0	1
**5.736**	0	0	0	1	1	0	0	0	0	1	0	1	0	0	0	0	0	0	0	0	0	1	0	0	1	1
**5.737**	0	0	0	1	1	0	0	0	0	1	0	0	0	0	0	1	0	0	0	0	0	0	0	1	0	1
**5.738**	0	0	0	1	1	0	0	0	0	0	0	1	1	0	0	0	0	0	0	1	0	1	0	1	0	1
**5.739**	0	0	0	1	1	0	0	0	0	0	0	1	1	0	0	0	0	0	0	1	0	0	1	1	1	1
**5.740**	0	0	0	1	1	0	0	0	0	0	0	1	0	0	0	0	0	0	0	1	0	1	1	1	1	1
**5.741**	0	0	0	1	1	0	0	0	0	0	0	1	0	0	0	0	0	0	0	1	0	1	1	0	1	1
**5.742**	0	0	0	1	1	0	0	0	0	0	0	1	0	0	0	0	0	0	0	0	0	1	0	1	1	1
**5.743**	0	0	0	1	0	0	1	0	1	1	0	1	1	0	0	0	0	0	0	1	0	1	0	1	0	1
**5.744**	0	0	0	1	0	0	1	0	1	1	0	0	0	0	0	0	0	0	0	1	0	0	0	0	0	1
**5.745**	0	0	0	1	0	0	1	0	1	0	0	1	1	0	0	0	0	0	0	0	0	0	0	0	0	1
**5.746**	0	0	0	1	0	0	1	0	1	0	0	1	0	0	0	0	0	0	0	0	0	0	1	0	0	1
**5.747**	0	0	0	1	0	0	1	0	1	0	0	0	0	0	0	1	0	0	0	0	0	0	0	1	1	1
**5.748**	0	0	0	1	0	0	1	0	1	0	0	0	0	0	0	0	0	0	0	1	0	0	0	0	1	1
**5.749**	0	0	0	1	0	0	1	0	1	0	0	0	0	0	0	0	0	0	0	0	0	1	0	0	0	1
**5.750**	0	0	0	1	0	0	1	0	1	0	0	0	0	0	0	0	0	0	0	0	0	0	1	1	0	1
**5.751**	0	0	0	1	0	0	1	0	0	0	0	1	0	1	0	0	0	0	0	0	0	0	0	1	0	1
**5.752**	0	0	0	1	0	0	1	0	0	0	0	0	1	0	0	1	0	0	0	1	0	1	0	1	0	1
**5.753**	0	0	0	1	0	0	1	0	0	0	0	0	1	0	0	1	0	0	0	0	0	0	0	0	0	1
**5.754**	0	0	0	1	0	0	0	0	1	1	0	0	1	1	0	0	0	0	0	1	0	0	0	1	1	1
**5.755**	0	0	0	1	0	0	0	0	1	0	0	1	1	0	0	0	0	0	0	0	0	0	0	1	0	1
**5.756**	0	0	0	1	0	0	0	0	1	0	0	1	0	1	0	0	0	0	0	1	0	0	0	1	1	1
**5.757**	0	0	0	1	0	0	0	0	1	0	0	1	0	0	0	0	0	0	0	0	0	1	1	0	1	1
**5.758**	0	0	0	1	0	0	0	0	1	0	0	0	1	0	0	0	0	0	0	0	0	0	0	1	1	1
**5.759**	0	0	0	1	0	0	0	0	1	0	0	0	0	1	0	0	0	0	0	0	0	0	0	1	1	1
**5.760**	0	0	0	1	0	0	0	0	0	1	0	1	0	0	0	1	0	0	0	0	0	0	0	0	0	1
**5.761**	0	0	0	1	0	0	0	0	0	1	0	0	1	0	0	0	0	0	0	0	0	0	0	1	0	1
**5.762**	0	0	0	1	0	0	0	0	0	0	0	1	1	0	0	0	0	0	0	0	0	1	0	1	1	1
**5.763**	0	0	0	1	0	0	0	0	0	0	0	1	0	0	0	0	0	0	0	0	0	1	1	1	0	1
**5.764**	0	0	0	1	0	0	0	0	0	0	0	0	1	0	0	0	0	0	0	1	0	1	0	1	1	1
**5.765**	0	0	0	1	0	0	0	0	0	0	0	0	0	1	0	0	0	0	0	1	0	1	1	0	0	1
**5.766**	0	0	0	1	0	0	0	0	0	0	0	0	0	1	0	0	0	0	0	0	0	1	0	1	0	1
**5.767**	0	0	0	1	0	0	0	0	0	0	0	0	0	0	0	1	0	0	0	1	0	0	0	1	0	1
**5.768**	0	0	0	1	0	0	0	0	0	0	0	0	0	0	0	1	0	0	0	0	0	0	0	0	0	1
**5.769**	0	0	0	0	1	0	1	0	1	1	0	1	1	0	0	0	0	0	0	1	0	0	1	0	0	1
**5.770**	0	0	0	0	1	0	1	0	1	1	0	1	1	0	0	0	0	0	0	1	0	0	0	0	0	1
**5.771**	0	0	0	0	1	0	1	0	1	0	0	0	0	0	0	0	0	0	0	0	0	0	1	1	1	1
**5.772**	0	0	0	0	1	0	1	0	0	0	0	1	0	0	0	1	0	0	0	0	0	0	0	1	1	1
**5.773**	0	0	0	0	1	0	1	0	0	0	0	0	0	0	0	0	0	0	0	0	0	0	0	1	0	1
**5.774**	0	0	0	0	1	0	0	0	1	1	0	1	1	1	0	0	0	0	0	0	0	0	0	1	1	1
**5.775**	0	0	0	0	1	0	0	0	1	1	0	1	1	0	0	0	0	0	0	0	0	1	1	1	1	1
**5.776**	0	0	0	0	1	0	0	0	1	1	0	1	0	1	0	0	0	0	0	1	0	1	0	0	0	1
**5.777**	0	0	0	0	1	0	0	0	1	1	0	1	0	0	0	0	0	0	0	0	0	0	1	1	1	1
**5.778**	0	0	0	0	1	0	0	0	1	0	0	1	1	1	0	0	0	0	0	1	0	1	0	1	1	1
**5.779**	0	0	0	0	1	0	0	0	1	0	0	1	1	1	0	0	0	0	0	0	0	0	1	1	0	1
**5.780**	0	0	0	0	1	0	0	0	1	0	0	1	1	1	0	0	0	0	0	0	0	0	1	0	0	1
**5.781**	0	0	0	0	1	0	0	0	1	0	0	1	1	0	0	0	0	0	0	1	0	1	1	0	1	1
**5.782**	0	0	0	0	1	0	0	0	1	0	0	1	1	0	0	0	0	0	0	0	0	1	1	1	1	1
**5.783**	0	0	0	0	1	0	0	0	1	0	0	1	1	0	0	0	0	0	0	0	0	0	1	1	0	1
**5.784**	0	0	0	0	1	0	0	0	1	0	0	1	0	1	0	0	0	0	0	1	0	0	0	1	1	1
**5.785**	0	0	0	0	1	0	0	0	1	0	0	1	0	0	0	0	0	0	0	1	0	0	0	0	1	1
**5.786**	0	0	0	0	1	0	0	0	1	0	0	0	1	0	0	0	0	0	0	1	0	1	1	0	1	1
**5.787**	0	0	0	0	1	0	0	0	1	0	0	0	0	0	0	0	0	0	0	0	0	0	1	0	1	1
**5.788**	0	0	0	0	1	0	0	0	0	1	0	0	0	0	0	1	0	0	0	0	0	0	0	1	0	1
**5.789**	0	0	0	0	1	0	0	0	0	0	0	1	1	0	0	0	0	0	0	1	0	0	1	1	1	1
**5.790**	0	0	0	0	1	0	0	0	0	0	0	1	1	0	0	0	0	0	0	0	0	1	1	1	0	1
**5.791**	0	0	0	0	1	0	0	0	0	0	0	1	0	0	0	0	0	0	0	0	0	1	1	1	0	1
**5.792**	0	0	0	0	1	0	0	0	0	0	0	0	1	0	0	0	0	0	0	0	0	1	0	1	0	1
**5.793**	0	0	0	0	1	0	0	0	0	0	0	0	1	0	0	0	0	0	0	0	0	0	0	1	1	1
**5.794**	0	0	0	0	0	0	1	0	1	1	0	1	1	0	0	0	0	0	0	1	0	0	0	1	0	1
**5.795**	0	0	0	0	0	0	1	0	1	1	0	1	1	0	0	0	0	0	0	0	0	1	0	1	0	1
**5.796**	0	0	0	0	0	0	1	0	1	1	0	1	0	0	0	0	0	0	0	1	0	0	0	0	1	1
**5.797**	0	0	0	0	0	0	1	0	1	1	0	1	0	0	0	0	0	0	0	0	0	0	0	0	1	1
**5.798**	0	0	0	0	0	0	1	0	1	0	0	1	0	1	0	0	0	0	0	0	0	0	0	0	0	1
**5.799**	0	0	0	0	0	0	1	0	1	0	0	1	0	0	0	0	0	0	0	1	0	1	0	1	0	1
**5.800**	0	0	0	0	0	0	1	0	1	0	0	1	0	0	0	0	0	0	0	0	0	0	1	0	0	1
**5.801**	0	0	0	0	0	0	1	0	0	1	0	1	1	0	0	0	0	0	0	0	0	0	0	1	0	1
**5.802**	0	0	0	0	0	0	1	0	0	0	0	1	1	0	0	0	0	0	0	0	0	1	0	1	0	1
**5.803**	0	0	0	0	0	0	1	0	0	0	0	0	1	0	0	1	0	0	0	1	0	0	0	1	1	1
**5.804**	0	0	0	0	0	0	1	0	0	0	0	0	0	0	0	1	0	0	0	0	0	0	1	0	1	1
**5.805**	0	0	0	0	0	0	0	0	1	1	0	1	1	0	0	0	0	0	0	1	0	0	0	1	1	1
**5.806**	0	0	0	0	0	0	0	0	1	1	0	1	0	0	0	1	0	0	0	1	0	1	0	1	1	1
**5.807**	0	0	0	0	0	0	0	0	1	1	0	1	0	0	0	1	0	0	0	1	0	0	1	1	1	1
**5.808**	0	0	0	0	0	0	0	0	1	1	0	1	0	0	0	1	0	0	0	0	0	1	0	1	1	1
**5.809**	0	0	0	0	0	0	0	0	1	1	0	1	0	0	0	1	0	0	0	0	0	0	0	1	1	1
**5.810**	0	0	0	0	0	0	0	0	1	1	0	1	0	0	0	0	0	0	0	1	0	1	1	1	1	1
**5.811**	0	0	0	0	0	0	0	0	1	0	0	1	1	0	0	0	0	0	0	0	0	0	0	1	1	1
**5.812**	0	1	1	1	1	0	1	0	1	1	0	1	1	1	0	0	0	0	0	1	0	0	1	1	0	1
**5.813**	0	1	1	1	1	0	1	0	1	0	0	1	0	0	0	0	0	0	0	1	0	0	0	0	0	1
**5.814**	0	1	1	1	1	0	0	0	1	1	0	1	1	1	0	0	0	0	0	1	0	1	0	1	1	1
**5.815**	0	1	1	1	1	0	0	0	1	1	0	1	1	0	0	0	0	0	0	1	0	1	1	1	1	1
**5.816**	0	1	1	1	1	0	0	0	1	1	0	1	0	0	0	0	0	0	0	0	0	0	0	1	1	1
**5.817**	0	1	1	1	1	0	0	0	1	0	0	1	1	1	0	0	0	0	0	1	0	1	0	1	1	1
**5.818**	0	1	1	1	1	0	0	0	1	0	0	1	1	0	0	0	0	0	0	0	0	0	0	0	1	1
**5.819**	0	1	1	1	0	0	0	0	1	0	0	1	0	0	0	0	0	0	0	0	0	0	0	0	0	1
**5.820**	0	1	1	0	1	0	0	0	1	0	0	1	0	0	0	0	0	0	0	1	0	1	1	1	1	1
**5.821**	0	1	1	0	1	0	0	0	1	0	0	1	0	0	0	0	0	0	0	0	0	1	0	0	1	1
**5.822**	0	1	1	0	1	0	0	0	0	1	0	1	0	1	0	0	0	0	0	1	0	0	0	0	1	1
**5.823**	0	1	1	0	1	0	0	0	0	1	0	1	0	0	0	0	0	0	0	1	0	0	1	1	0	1
**5.824**	0	1	1	0	1	0	0	0	0	0	0	1	1	0	0	0	0	0	0	1	0	1	0	1	1	1
**5.825**	0	1	1	0	1	0	0	0	0	0	0	1	0	0	0	0	0	0	0	0	0	1	0	0	1	1
**5.826**	0	1	1	0	0	0	1	0	1	0	0	0	0	0	0	0	0	0	0	1	0	0	0	1	1	1
**5.827**	0	1	1	0	0	0	0	0	1	1	0	1	0	0	0	0	0	0	0	1	0	0	1	0	0	1
**5.828**	0	1	1	0	0	0	0	0	1	0	0	1	0	0	0	0	0	0	0	0	0	1	0	1	1	1
**5.829**	0	1	0	1	1	0	1	0	1	1	0	1	1	1	0	0	0	0	0	1	0	1	1	0	0	1
**5.830**	0	1	0	1	1	0	1	0	0	1	0	1	1	0	0	0	0	0	0	1	0	1	0	1	1	1
**5.831**	0	1	0	1	1	0	0	0	1	1	0	1	1	0	0	0	0	0	0	1	0	1	1	1	1	1
**5.832**	0	1	0	1	1	0	0	0	1	0	0	1	1	0	0	0	0	0	0	1	0	0	1	1	0	1
**5.833**	0	1	0	1	1	0	0	0	1	0	0	0	1	0	0	0	0	0	0	1	0	0	1	1	1	1
**5.834**	0	1	0	1	1	0	0	0	0	1	0	1	1	0	0	0	0	0	0	1	0	1	0	0	0	1
**5.835**	0	1	0	1	1	0	0	0	0	1	0	1	1	0	0	0	0	0	0	0	0	1	0	1	1	1
**5.836**	0	1	0	1	1	0	0	0	0	1	0	1	0	1	0	0	0	0	0	1	0	1	0	0	1	1
**5.837**	0	1	0	1	1	0	0	0	0	1	0	1	0	1	0	0	0	0	0	0	0	0	0	0	1	1
**5.838**	0	1	0	1	1	0	0	0	0	1	0	0	0	1	0	0	0	0	0	1	0	0	0	0	0	1
**5.839**	0	1	0	1	1	0	0	0	0	1	0	0	0	0	0	0	0	0	0	1	0	0	0	1	1	1
**5.840**	0	1	0	1	1	0	0	0	0	0	0	1	1	1	0	0	0	0	0	1	0	1	1	1	1	1
**5.841**	0	1	0	1	0	0	1	0	1	1	0	1	1	1	0	0	0	0	0	1	0	1	0	0	0	1
**5.842**	0	1	0	1	0	0	1	0	1	1	0	1	1	0	0	0	0	0	0	0	0	0	0	0	0	1
**5.843**	0	1	0	1	0	0	0	0	1	1	0	1	0	1	0	0	0	0	0	1	0	1	0	0	0	1
**5.844**	0	1	0	1	0	0	0	0	1	1	0	1	0	0	0	0	0	0	0	0	0	0	1	1	0	1
**5.845**	0	1	0	1	0	0	0	0	1	0	0	1	1	1	0	0	0	0	0	1	0	0	0	0	0	1
**5.846**	0	1	0	1	0	0	0	0	1	0	0	1	1	0	0	0	0	0	0	0	0	1	0	1	0	1
**5.847**	0	1	0	1	0	0	0	0	1	0	0	1	0	0	0	0	0	0	0	0	0	1	0	0	1	1
**5.848**	0	1	0	1	0	0	0	0	0	0	0	1	1	0	0	0	0	0	0	1	0	0	1	0	1	1
**5.849**	0	1	0	1	0	0	0	0	0	0	0	0	1	0	0	0	0	0	0	0	0	0	0	1	1	1
**5.850**	0	1	0	0	1	0	1	0	1	1	0	1	1	1	0	0	0	0	0	1	0	0	1	1	0	1
**5.851**	0	1	0	0	1	0	1	0	0	0	0	0	0	0	0	0	0	0	0	1	0	0	1	1	1	1
**5.852**	0	1	0	0	1	0	0	0	1	0	0	1	1	1	0	0	0	0	0	0	0	0	0	0	0	1
**5.853**	0	1	0	0	1	0	0	0	1	0	0	1	1	0	0	0	0	0	0	1	0	1	1	0	0	1
**5.854**	0	1	0	0	1	0	0	0	0	1	0	0	0	1	0	0	0	0	0	1	0	0	0	0	0	1

CMP	CD2	CD3	CD4	CD7	CD8	CD10	CD13	CD18	CD26	CD29	CD36	CD44	CD45RA	CD49f	CD54	CD56	CD57	CD58	CD62L	CD71	CD80	Cytokeratin	Histone_H1	HLA-DQ	HLA-DR	Freq. (voxel)
**5.855**	0	1	0	0	1	0	0	0	0	1	0	0	0	0	0	0	0	0	0	1	0	1	0	1	1	1
**5.856**	0	1	0	0	1	0	0	0	0	0	0	1	1	1	0	0	0	0	0	1	0	1	1	1	1	1
**5.857**	0	1	0	0	1	0	0	0	0	0	0	1	0	1	0	0	0	0	0	1	0	1	1	1	1	1
**5.858**	0	1	0	0	1	0	0	0	0	0	0	0	0	1	0	0	0	0	0	1	0	0	0	0	1	1
**5.859**	0	1	0	0	1	0	0	0	0	0	0	0	0	0	0	0	0	0	0	0	0	0	1	1	0	1
**5.860**	0	1	0	0	0	0	1	0	1	1	0	1	1	0	0	0	0	0	0	1	0	1	0	1	1	1
**5.861**	0	1	0	0	0	0	1	0	1	0	0	1	0	0	0	0	0	0	0	1	0	1	0	1	1	1
**5.862**	0	1	0	0	0	0	1	0	1	0	0	1	0	0	0	0	0	0	0	0	0	0	1	1	1	1
**5.863**	0	1	0	0	0	0	1	0	0	1	0	1	0	0	0	0	0	0	0	1	0	1	0	0	0	1
**5.864**	0	1	0	0	0	0	1	0	0	0	0	1	1	0	0	0	0	0	0	1	0	1	0	1	1	1
**5.865**	0	1	0	0	0	0	0	0	1	1	0	1	0	0	0	0	0	0	0	1	0	1	0	0	1	1
**5.866**	0	1	0	0	0	0	0	0	1	0	0	1	1	1	0	0	0	0	0	1	0	0	0	0	0	1
**5.867**	0	1	0	0	0	0	0	0	1	0	0	1	1	1	0	0	0	0	0	0	0	1	1	1	1	1
**5.868**	0	1	0	0	0	0	0	0	1	0	0	1	1	1	0	0	0	0	0	0	0	1	1	0	0	1
**5.869**	0	1	0	0	0	0	0	0	1	0	0	1	1	0	0	0	0	0	0	1	0	1	0	1	0	1
**5.870**	0	1	0	0	0	0	0	0	1	0	0	1	1	0	0	0	0	0	0	0	0	1	1	1	1	1
**5.871**	0	1	0	0	0	0	0	0	1	0	0	1	0	1	0	0	0	0	0	1	0	1	1	1	1	1
**5.872**	0	1	0	0	0	0	0	0	0	1	0	1	1	0	0	0	0	0	0	0	0	0	1	0	1	1
**5.873**	0	1	0	0	0	0	0	0	0	0	0	1	0	1	0	0	0	0	0	1	0	1	1	1	1	1
**5.874**	0	1	0	0	0	0	0	0	0	0	0	0	1	0	0	0	0	0	0	1	0	1	1	0	0	1
**5.875**	0	1	0	0	0	0	0	0	0	0	0	0	1	0	0	0	0	0	0	1	0	0	1	1	1	1
**5.876**	0	0	1	1	1	0	0	0	1	0	0	1	0	0	0	0	0	0	0	0	0	0	0	0	1	1
**5.877**	0	0	1	1	0	0	1	0	0	1	0	1	1	0	0	0	0	0	0	1	0	0	0	1	1	1
**5.878**	0	0	1	1	0	0	0	0	0	0	0	0	1	0	0	0	0	0	0	1	0	0	0	1	1	1
**5.879**	0	0	1	0	1	0	1	0	1	0	0	1	0	0	0	0	0	0	0	0	0	0	1	1	1	1
**5.880**	0	0	1	0	1	0	1	0	0	1	0	0	0	0	0	0	0	0	0	1	0	0	0	1	1	1
**5.881**	0	0	1	0	1	0	0	0	1	0	0	1	0	0	0	0	0	0	0	0	0	0	1	0	0	1
**5.882**	0	0	1	0	1	0	0	0	0	1	0	1	0	0	0	0	0	0	0	1	0	0	1	1	0	1
**5.883**	0	0	1	0	1	0	0	0	0	0	0	1	1	0	0	0	0	0	0	1	0	0	0	1	1	1
**5.884**	0	0	1	0	1	0	0	0	0	0	0	0	1	0	0	0	0	0	0	1	0	0	0	0	1	1
**5.885**	0	0	1	0	0	0	0	0	1	1	0	1	0	0	0	1	0	0	0	0	0	1	0	0	0	1
**5.886**	0	0	1	0	0	0	0	0	1	0	0	0	0	1	0	0	0	0	0	1	0	1	0	1	0	1
**5.887**	0	0	1	0	0	0	0	0	0	0	0	1	1	1	0	0	0	0	0	1	0	0	0	1	1	1
**5.888**	0	0	1	0	0	0	0	0	0	0	0	1	0	0	0	0	0	0	0	1	0	1	1	0	1	1
**5.889**	0	0	1	0	0	0	0	0	0	0	0	0	1	0	0	1	0	0	0	1	0	1	0	0	1	1
**5.890**	0	0	0	1	1	0	1	0	1	0	0	1	1	0	0	0	0	0	0	0	0	0	0	0	1	1
**5.891**	0	0	0	1	1	0	1	0	1	0	0	0	0	0	0	0	0	0	0	0	0	0	1	0	1	1
**5.892**	0	0	0	1	1	0	0	0	1	1	0	1	0	1	0	0	0	0	0	0	0	0	1	0	0	1
**5.893**	0	0	0	1	1	0	0	0	1	1	0	1	0	0	0	0	0	0	0	0	0	1	0	1	1	1
**5.894**	0	0	0	1	1	0	0	0	1	0	0	1	1	0	0	0	0	0	0	1	0	0	1	0	0	1
**5.895**	0	0	0	1	1	0	0	0	1	0	0	1	1	0	0	0	0	0	0	0	0	1	0	1	1	1
**5.896**	0	0	0	1	1	0	0	0	1	0	0	1	0	1	0	0	0	0	0	0	0	0	1	0	0	1
**5.897**	0	0	0	1	1	0	0	0	1	0	0	1	0	1	0	0	0	0	0	0	0	0	0	1	1	1
**5.898**	0	0	0	1	1	0	0	0	1	0	0	0	0	0	0	0	0	0	0	0	0	0	0	1	1	1
**5.899**	0	0	0	1	1	0	0	0	0	1	0	1	0	1	0	0	0	0	0	1	0	1	0	1	0	1
**5.900**	0	0	0	1	1	0	0	0	0	1	0	0	0	0	0	0	0	0	0	0	0	0	0	1	0	1
**5.901**	0	0	0	1	1	0	0	0	0	0	0	1	0	1	0	0	0	0	0	1	0	0	1	0	0	1
**5.902**	0	0	0	1	1	0	0	0	0	0	0	1	0	0	0	0	0	0	0	0	0	1	1	0	1	1
**5.903**	0	0	0	1	1	0	0	0	0	0	0	1	0	0	0	0	0	0	0	0	0	0	1	1	0	1
**5.904**	0	0	0	1	1	0	0	0	0	0	0	0	0	0	0	0	0	0	0	1	0	0	1	1	1	1
**5.905**	0	0	0	1	1	0	0	0	0	0	0	0	0	0	0	0	0	0	0	1	0	0	1	0	1	1
**5.906**	0	0	0	1	0	0	1	0	0	1	0	1	1	0	0	0	0	0	0	1	0	0	0	0	0	1
**5.907**	0	0	0	1	0	0	1	0	0	0	0	0	0	1	0	1	0	0	0	0	0	1	0	0	0	1
**5.908**	0	0	0	1	0	0	1	0	0	0	0	0	0	1	0	0	0	0	0	1	0	0	0	0	0	1
**5.909**	0	0	0	1	0	0	1	0	0	0	0	0	0	0	0	1	0	0	0	1	0	0	0	1	1	1
**5.910**	0	0	0	1	0	0	1	0	0	0	0	0	0	0	0	1	0	0	0	0	0	1	0	0	0	1
**5.911**	0	0	0	1	0	0	0	0	1	1	0	1	0	1	0	0	0	0	0	1	0	1	0	1	1	1
**5.912**	0	0	0	1	0	0	0	0	1	1	0	1	0	0	0	0	0	0	0	1	0	1	0	1	0	1
**5.913**	0	0	0	1	0	0	0	0	1	1	0	0	0	0	0	0	0	0	0	0	0	0	1	0	0	1
**5.914**	0	0	0	1	0	0	0	0	1	0	0	1	1	1	0	0	0	0	0	1	0	0	0	1	1	1
**5.915**	0	0	0	1	0	0	0	0	1	0	0	1	0	0	0	0	0	0	0	1	0	1	1	1	0	1
**5.916**	0	0	0	1	0	0	0	0	1	0	0	1	0	0	0	0	0	0	0	1	0	0	0	1	0	1
**5.917**	0	0	0	1	0	0	0	0	1	0	0	0	0	0	0	0	0	0	0	0	0	0	1	1	0	1
**5.918**	0	0	0	1	0	0	0	0	0	1	0	0	1	0	0	0	0	0	0	1	0	0	1	0	0	1
**5.919**	0	0	0	1	0	0	0	0	0	0	0	1	1	0	0	0	0	0	0	0	0	1	1	0	0	1
**5.920**	0	0	0	1	0	0	0	0	0	0	0	1	0	0	0	0	0	0	0	1	0	0	1	0	1	1
**5.921**	0	0	0	1	0	0	0	0	0	0	0	0	0	0	0	1	0	0	0	1	0	0	0	0	0	1
**5.922**	0	0	0	0	1	0	1	0	1	0	0	1	0	0	0	0	0	0	0	0	0	0	0	0	0	1
**5.923**	0	0	0	0	1	0	1	0	0	1	0	1	0	0	0	0	0	0	0	0	0	1	0	1	0	1
**5.924**	0	0	0	0	1	0	1	0	0	1	0	0	0	0	0	0	0	0	0	1	0	0	0	1	1	1
**5.925**	0	0	0	0	1	0	1	0	0	0	0	0	0	0	0	0	0	0	0	1	0	0	0	1	1	1
**5.926**	0	0	0	0	1	0	0	0	1	1	0	1	1	1	0	0	0	0	0	0	0	0	1	0	0	1
**5.927**	0	0	0	0	1	0	0	0	1	1	0	1	0	1	0	0	0	0	0	0	0	1	0	1	0	1
**5.928**	0	0	0	0	1	0	0	0	1	1	0	0	0	0	0	0	0	0	0	0	0	1	0	1	1	1
**5.929**	0	0	0	0	1	0	0	0	1	0	0	1	1	1	0	0	0	0	0	0	0	1	1	0	0	1
**5.930**	0	0	0	0	1	0	0	0	1	0	0	1	1	0	0	0	0	0	0	1	0	1	0	0	0	1
**5.931**	0	0	0	0	1	0	0	0	1	0	0	1	0	1	0	0	0	0	0	1	0	1	0	1	1	1
**5.932**	0	0	0	0	1	0	0	0	0	1	0	1	0	1	0	0	0	0	0	0	0	1	0	1	0	1
**5.933**	0	0	0	0	1	0	0	0	0	1	0	1	0	0	0	0	0	0	0	1	0	1	1	0	1	1
**5.934**	0	0	0	0	1	0	0	0	0	1	0	0	0	0	0	0	0	0	0	0	0	1	0	1	1	1
**5.935**	0	0	0	0	1	0	0	0	0	0	0	1	1	0	0	0	0	0	0	1	0	1	1	0	1	1
**5.936**	0	0	0	0	1	0	0	0	0	0	0	0	1	1	0	0	0	0	0	1	0	0	0	0	0	1
**5.937**	0	0	0	0	1	0	0	0	0	0	0	0	0	0	0	0	0	0	0	1	0	1	1	0	1	1
**5.938**	0	0	0	0	0	0	1	0	1	1	0	1	1	0	0	0	0	0	0	0	0	0	0	1	0	1
**5.939**	0	0	0	0	0	0	1	0	1	1	0	1	0	0	0	0	0	0	0	1	0	1	1	0	0	1
**5.940**	0	0	0	0	0	0	1	0	1	1	0	0	0	0	0	0	0	0	0	1	0	0	0	1	1	1
**5.941**	0	0	0	0	0	0	1	0	1	0	0	1	0	0	0	1	0	0	0	0	0	0	1	1	0	1
**5.942**	0	0	0	0	0	0	1	0	1	0	0	1	0	0	0	0	0	0	0	0	0	1	1	0	0	1
**5.943**	0	0	0	0	0	0	1	0	1	0	0	0	0	0	0	0	0	0	0	0	0	0	1	0	1	1
**5.944**	0	0	0	0	0	0	1	0	0	0	0	1	0	0	0	0	0	0	0	1	0	0	1	1	0	1
**5.945**	0	0	0	0	0	0	1	0	0	0	0	0	1	0	0	1	0	0	0	0	0	0	0	1	1	1
**5.946**	0	0	0	0	0	0	0	0	1	1	0	1	0	0	0	0	0	0	0	0	0	1	1	1	0	1
**5.947**	0	0	0	0	0	0	0	0	1	0	1	0	0	0	0	0	0	0	0	1	0	0	0	0	1	1
**5.948**	0	0	0	0	0	0	0	0	1	0	1	0	0	0	0	0	0	0	0	1	0	0	0	0	0	1
**5.949**	0	0	0	0	0	0	0	0	1	0	0	1	1	0	0	0	0	0	0	1	0	0	0	1	0	1
**5.950**	0	0	0	0	0	0	0	0	1	0	0	1	0	1	0	0	0	0	0	0	0	0	1	0	0	1
**5.951**	0	0	0	0	0	0	0	0	1	0	0	1	0	0	0	1	0	0	0	0	0	0	1	1	0	1
**5.952**	0	0	0	0	0	0	0	0	0	1	0	1	1	0	0	0	0	0	0	0	0	1	0	1	1	1
**5.953**	0	1	1	1	1	0	1	0	0	1	0	1	1	1	0	0	0	0	0	1	0	0	0	0	0	1
**5.954**	0	1	1	1	1	0	0	0	0	1	0	0	0	0	0	0	0	0	0	1	0	0	0	0	0	1
**5.955**	0	1	1	1	1	0	0	0	0	1	0	0	0	0	0	0	0	0	0	0	0	0	0	0	0	1
**5.956**	0	1	1	1	1	0	0	0	0	0	0	1	1	1	0	0	0	0	0	0	0	1	0	1	1	1
**5.957**	0	1	1	1	0	0	1	0	0	0	0	1	0	0	0	0	0	0	0	0	0	0	0	1	1	1
**5.958**	0	1	1	1	0	0	0	0	1	1	0	1	0	0	0	0	0	0	0	0	0	0	1	0	0	1
**5.959**	0	1	1	1	0	0	0	0	1	0	0	1	0	1	0	0	0	0	0	1	0	1	0	1	1	1
**5.960**	0	1	1	1	0	0	0	0	0	0	0	1	0	0	0	0	0	0	0	0	0	0	0	1	1	1
**5.961**	0	1	1	1	0	0	0	0	0	0	0	0	1	0	0	0	0	0	0	0	0	0	1	0	0	1
**5.962**	0	1	1	0	1	0	1	0	1	1	0	1	1	1	0	0	0	0	0	1	0	0	1	0	0	1
**5.963**	0	1	1	0	1	0	1	0	1	0	0	0	0	0	0	0	0	0	0	1	0	0	0	1	1	1
**5.964**	0	1	1	0	1	0	1	0	0	1	0	1	1	0	0	0	0	0	0	0	0	1	0	1	1	1
**5.965**	0	1	1	0	1	0	0	0	1	1	0	1	1	0	0	0	0	0	0	1	0	1	1	1	1	1
**5.966**	0	1	1	0	1	0	0	0	1	1	0	1	0	1	0	0	0	0	0	1	0	0	0	0	0	1
**5.967**	0	1	1	0	1	0	0	0	1	0	0	1	0	0	0	0	0	0	0	0	0	0	1	0	0	1
**5.968**	0	1	1	0	1	0	0	0	0	1	0	1	1	0	0	0	0	0	0	1	0	1	0	1	1	1
**5.969**	0	1	1	0	1	0	0	0	0	1	0	1	1	0	0	0	0	0	0	0	0	1	1	1	1	1
**5.970**	0	1	1	0	0	0	0	0	1	0	0	1	1	0	0	0	0	0	0	0	0	0	0	1	1	1
**5.971**	0	1	1	0	0	0	0	0	1	0	0	1	1	0	0	0	0	0	0	0	0	0	0	0	0	1
**5.972**	0	1	1	0	0	0	0	0	1	0	0	1	0	1	0	0	0	0	0	1	0	1	1	1	1	1
**5.973**	0	1	1	0	0	0	0	0	1	0	0	1	0	1	0	0	0	0	0	1	0	1	0	1	1	1
**5.974**	0	1	1	0	0	0	0	0	0	0	0	1	1	0	0	0	0	0	0	0	0	1	1	1	1	1
**5.975**	0	1	1	0	0	0	0	0	0	0	0	1	1	0	0	0	0	0	0	0	0	1	0	1	1	1
**5.976**	0	1	1	0	0	0	0	0	0	0	0	1	0	1	0	0	0	0	0	1	0	1	1	1	1	1

CMP	CD2	CD3	CD4	CD7	CD8	CD10	CD13	CD18	CD26	CD29	CD36	CD44	CD45RA	CD49f	CD54	CD56	CD57	CD58	CD62L	CD71	CD80	Cytokeratin	Histone_H1	HLA-DQ	HLA-DR	Freq. (voxel)
**5.977**	0	1	1	0	0	0	0	0	0	0	0	0	0	0	0	0	0	0	0	1	0	1	1	1	1	1
**5.978**	0	1	0	1	1	0	1	0	1	0	0	1	1	0	0	0	0	0	0	1	0	1	1	1	1	1
**5.979**	0	1	0	1	1	0	1	0	1	0	0	1	0	0	0	0	0	0	0	0	0	0	1	0	1	1
**5.980**	0	1	0	1	1	0	1	0	1	0	0	0	0	0	0	0	0	0	0	0	0	0	1	0	1	1
**5.981**	0	1	0	1	1	0	1	0	0	1	0	1	1	1	0	0	0	0	0	1	0	1	1	0	0	1
**5.982**	0	1	0	1	1	0	1	0	0	1	0	1	0	1	0	0	0	0	0	1	0	0	0	0	0	1
**5.983**	0	1	0	1	1	0	0	0	1	1	0	1	1	0	0	0	0	0	0	1	0	1	1	1	0	1
**5.984**	0	1	0	1	1	0	0	0	1	0	0	1	1	1	0	0	0	0	0	0	0	1	0	1	1	1
**5.985**	0	1	0	1	1	0	0	0	1	0	0	1	0	1	0	0	0	0	0	1	0	0	0	1	1	1
**5.986**	0	1	0	1	1	0	0	0	1	0	0	0	1	0	0	0	0	0	0	1	0	0	0	0	1	1
**5.987**	0	1	0	1	1	0	0	0	1	0	0	0	0	0	0	0	0	0	0	1	0	0	0	1	1	1
**5.988**	0	1	0	1	1	0	0	0	0	0	0	1	1	1	0	0	0	0	0	1	0	0	0	1	0	1
**5.989**	0	1	0	1	1	0	0	0	0	0	0	1	1	1	0	0	0	0	0	0	0	1	0	1	1	1
**5.990**	0	1	0	1	0	0	1	0	1	1	0	1	1	0	0	0	0	0	0	1	0	0	0	0	0	1
**5.991**	0	1	0	1	0	0	1	0	1	1	0	1	0	0	0	0	0	0	0	1	0	0	0	0	0	1
**5.992**	0	1	0	1	0	0	1	0	0	0	0	1	1	1	0	0	0	0	0	0	0	1	0	1	1	1
**5.993**	0	1	0	1	0	0	0	0	1	1	0	1	1	0	0	0	0	0	0	1	0	1	0	1	1	1
**5.994**	0	1	0	1	0	0	0	0	1	1	0	1	0	0	0	1	0	0	0	0	0	0	0	0	0	1
**5.995**	0	1	0	1	0	0	0	0	1	0	0	1	0	1	0	0	0	0	0	0	0	1	1	0	1	1
**5.996**	0	1	0	1	0	0	0	0	0	1	0	1	0	0	0	1	0	0	0	0	0	0	0	0	0	1
**5.997**	0	1	0	1	0	0	0	0	0	1	0	1	0	0	0	0	0	0	0	0	0	1	1	0	0	1
**5.998**	0	1	0	1	0	0	0	0	0	0	0	1	1	1	0	0	0	0	0	1	0	1	1	1	1	1
**5.999**	0	1	0	1	0	0	0	0	0	0	0	1	1	1	0	0	0	0	0	1	0	0	0	1	0	1
**6.000**	0	1	0	1	0	0	0	0	0	0	0	1	1	1	0	0	0	0	0	0	0	1	0	1	1	1
**6.001**	0	1	0	1	0	0	0	0	0	0	0	1	1	0	0	0	0	0	0	1	0	1	0	0	1	1
**6.002**	0	1	0	0	1	0	1	0	1	1	0	1	1	1	0	0	0	0	0	1	0	1	1	1	0	1
**6.003**	0	1	0	0	1	0	1	0	1	1	0	1	0	0	0	0	0	0	0	1	0	1	0	1	1	1
**6.004**	0	1	0	0	1	0	1	0	0	0	0	1	0	0	0	0	0	0	0	1	0	1	0	0	0	1
**6.005**	0	1	0	0	1	0	1	0	0	0	0	1	0	0	0	0	0	0	0	0	0	0	1	0	1	1
**6.006**	0	1	0	0	1	0	0	0	1	1	0	1	1	1	0	1	0	0	0	1	0	0	0	0	0	1
**6.007**	0	1	0	0	1	0	0	0	1	1	0	1	1	0	0	0	0	0	0	1	0	1	0	1	0	1
**6.008**	0	1	0	0	1	0	0	0	1	1	0	1	0	1	0	0	0	0	0	0	0	1	1	1	1	1
**6.009**	0	1	0	0	1	0	0	0	1	1	0	1	0	1	0	0	0	0	0	0	0	1	1	0	1	1
**6.010**	0	1	0	0	1	0	0	0	1	1	0	0	0	1	0	0	0	0	0	1	0	1	0	1	1	1
**6.011**	0	1	0	0	1	0	0	0	1	0	0	1	0	1	0	0	0	0	0	1	0	0	0	1	1	1
**6.012**	0	1	0	0	1	0	0	0	0	1	0	1	1	0	0	0	0	0	0	1	0	1	1	0	1	1
**6.013**	0	1	0	0	1	0	0	0	0	1	0	0	1	0	0	0	0	0	0	1	0	0	0	0	1	1
**6.014**	0	1	0	0	1	0	0	0	0	1	0	0	1	0	0	0	0	0	0	0	0	0	0	0	1	1
**6.015**	0	1	0	0	1	0	0	0	0	1	0	0	0	0	0	0	0	0	0	1	0	1	0	0	0	1
**6.016**	0	1	0	0	1	0	0	0	0	1	0	0	0	0	0	0	0	0	0	0	0	0	1	0	1	1
**6.017**	0	1	0	0	1	0	0	0	0	0	0	1	1	1	0	0	0	0	0	0	0	1	0	0	0	1
**6.018**	0	1	0	0	0	0	1	0	1	1	0	1	1	0	0	0	0	0	0	1	0	0	1	0	0	1
**6.019**	0	1	0	0	0	0	1	0	1	1	0	0	0	0	0	0	0	0	0	1	0	0	0	1	1	1
**6.020**	0	1	0	0	0	0	0	0	1	1	0	1	1	1	0	0	0	0	0	1	0	1	1	0	1	1
**6.021**	0	1	0	0	0	0	0	0	1	1	0	1	1	1	0	0	0	0	0	1	0	1	0	1	1	1
**6.022**	0	1	0	0	0	0	0	0	1	1	0	1	1	1	0	0	0	0	0	1	0	1	0	1	0	1
**6.023**	0	1	0	0	0	0	0	0	1	1	0	1	0	1	0	0	0	0	0	0	0	1	1	0	1	1
**6.024**	0	1	0	0	0	0	0	0	1	0	0	1	1	1	0	0	0	0	0	1	0	1	1	0	0	1
**6.025**	0	1	0	0	0	0	0	0	1	0	0	1	1	1	0	0	0	0	0	0	0	1	1	0	1	1
**6.026**	0	1	0	0	0	0	0	0	1	0	0	1	1	0	0	0	0	0	0	1	0	0	1	1	0	1
**6.027**	0	1	0	0	0	0	0	0	1	0	0	1	0	1	0	0	0	0	0	1	0	0	0	1	1	1
**6.028**	0	1	0	0	0	0	0	0	1	0	0	0	0	1	0	0	0	0	0	0	0	1	1	1	1	1
**6.029**	0	1	0	0	0	0	0	0	0	1	0	1	1	1	0	0	0	0	0	1	0	1	1	0	1	1
**6.030**	0	1	0	0	0	0	0	0	0	1	0	0	1	0	0	0	0	0	0	0	0	0	0	0	1	1
**6.031**	0	1	0	0	0	0	0	0	0	0	0	1	1	1	0	0	0	0	0	1	0	1	1	1	1	1
**6.032**	0	1	0	0	0	0	0	0	0	0	0	1	1	1	0	0	0	0	0	1	0	1	1	0	0	1
**6.033**	0	0	1	1	1	0	0	0	1	1	0	1	0	0	0	0	0	0	0	1	0	0	0	0	1	1
**6.034**	0	0	1	1	1	0	0	0	0	0	0	1	0	0	0	0	0	0	0	1	0	0	0	0	0	1
**6.035**	0	0	1	1	0	0	1	0	1	1	0	1	1	0	0	0	0	0	0	1	0	0	0	1	1	1
**6.036**	0	0	1	1	0	0	1	0	1	1	0	1	0	0	0	1	0	0	0	1	0	0	0	1	1	1
**6.037**	0	0	1	1	0	0	1	0	0	1	0	1	1	0	0	1	0	0	0	1	0	0	0	1	1	1
**6.038**	0	0	1	1	0	0	1	0	0	1	0	1	1	0	0	0	0	0	0	0	0	0	0	1	1	1
**6.039**	0	0	1	1	0	0	0	0	1	0	0	1	1	0	0	0	0	0	0	0	0	0	0	1	1	1
**6.040**	0	0	1	1	0	0	0	0	1	0	0	0	0	0	0	0	0	0	0	0	0	1	0	0	0	1
**6.041**	0	0	1	1	0	0	0	0	0	0	0	0	1	1	0	0	0	0	0	1	0	0	0	0	1	1
**6.042**	0	0	1	0	1	0	0	0	1	1	0	1	0	0	0	0	0	0	0	1	0	1	0	0	0	1
**6.043**	0	0	1	0	1	0	0	0	0	1	0	1	0	0	0	0	0	0	0	1	0	1	0	0	1	1
**6.044**	0	0	1	0	1	0	0	0	0	0	0	1	0	1	0	0	0	0	0	1	0	1	0	1	1	1
**6.045**	0	0	1	0	1	0	0	0	0	0	0	1	0	0	0	0	0	0	0	1	0	0	0	0	1	1
**6.046**	0	0	1	0	1	0	0	0	0	0	0	0	0	0	0	0	0	0	0	1	0	0	0	0	1	1
**6.047**	0	0	1	0	0	0	1	0	0	0	0	0	1	0	0	0	0	0	0	0	0	1	0	1	0	1
**6.048**	0	0	1	0	0	0	0	0	1	1	0	1	1	0	0	0	0	0	0	1	0	0	1	1	0	1
**6.049**	0	0	1	0	0	0	0	0	0	1	0	1	1	0	0	0	0	0	0	1	0	0	1	0	0	1
**6.050**	0	0	1	0	0	0	0	0	0	1	0	0	1	1	0	0	0	0	0	0	0	0	1	1	0	1
**6.051**	0	0	1	0	0	0	0	0	0	1	0	0	1	0	0	0	0	0	0	1	0	0	0	1	0	1
**6.052**	0	0	1	0	0	0	0	0	0	0	0	0	1	0	0	0	0	0	0	1	0	0	1	1	0	1
**6.053**	0	0	1	0	0	0	0	0	0	0	0	0	1	0	0	0	0	0	0	1	0	0	1	0	1	1
**6.054**	0	0	0	1	1	0	1	0	0	1	0	1	0	1	0	0	0	0	0	0	0	0	0	0	0	1
**6.055**	0	0	0	1	1	0	1	0	0	1	0	1	0	0	0	0	0	0	0	0	0	0	0	0	1	1
**6.056**	0	0	0	1	1	0	1	0	0	0	0	1	0	0	0	0	0	0	0	0	0	0	1	1	0	1
**6.057**	0	0	0	1	1	0	0	0	1	0	0	1	0	0	0	0	0	0	0	0	0	0	1	0	1	1
**6.058**	0	0	0	1	1	0	0	0	0	1	0	0	0	0	0	0	0	0	0	0	0	0	0	0	1	1
**6.059**	0	0	0	1	0	0	1	0	0	1	0	0	1	1	0	0	0	0	0	1	0	0	0	0	0	1
**6.060**	0	0	0	1	0	0	1	0	0	0	0	0	0	0	0	1	0	0	0	0	0	0	0	1	1	1
**6.061**	0	0	0	1	0	0	0	0	1	1	0	1	1	1	0	0	0	0	0	1	0	1	1	1	0	1
**6.062**	0	0	0	1	0	0	0	0	1	1	0	1	0	0	0	0	0	0	0	1	0	0	0	0	1	1
**6.063**	0	0	0	1	0	0	0	0	1	0	0	1	0	1	0	0	0	0	0	0	0	1	0	0	1	1
**6.064**	0	0	0	1	0	0	0	0	0	1	0	1	1	1	0	0	0	0	0	1	0	1	0	0	0	1
**6.065**	0	0	0	1	0	0	0	0	0	1	0	1	1	1	0	0	0	0	0	0	0	1	0	1	0	1
**6.066**	0	0	0	1	0	0	0	0	0	1	0	1	1	0	0	0	0	0	0	1	0	0	0	1	1	1
**6.067**	0	0	0	1	0	0	0	0	0	1	0	1	1	0	0	0	0	0	0	0	0	1	1	0	0	1
**6.068**	0	0	0	1	0	0	0	0	0	1	0	1	1	0	0	0	0	0	0	0	0	0	0	1	1	1
**6.069**	0	0	0	1	0	0	0	0	0	1	0	1	0	1	0	0	0	0	0	1	0	1	0	0	1	1
**6.070**	0	0	0	1	0	0	0	0	0	1	0	1	0	0	0	0	0	0	0	1	0	1	1	1	0	1
**6.071**	0	0	0	1	0	0	0	0	0	1	0	0	1	1	0	1	0	0	0	0	0	0	0	0	0	1
**6.072**	0	0	0	1	0	0	0	0	0	1	0	0	1	1	0	0	0	0	0	1	0	0	0	1	0	1
**6.073**	0	0	0	1	0	0	0	0	0	0	0	0	1	0	0	1	0	0	0	1	0	1	0	0	1	1
**6.074**	0	0	0	1	0	0	0	0	0	0	0	0	1	0	0	1	0	0	0	1	0	0	0	0	1	1
**6.075**	0	0	0	1	0	0	0	0	0	0	0	0	0	0	0	1	0	0	0	1	0	0	1	0	0	1
**6.076**	0	0	0	1	0	0	0	0	0	0	0	0	0	0	0	0	0	0	0	1	0	0	0	0	1	1
**6.077**	0	0	0	0	1	0	1	0	0	0	0	0	0	0	0	0	0	0	0	0	0	0	1	0	1	1
**6.078**	0	0	0	0	1	0	0	0	1	0	0	0	1	0	0	0	0	0	0	0	0	0	0	0	1	1
**6.079**	0	0	0	0	1	0	0	0	0	1	0	1	1	0	0	0	0	0	0	0	0	1	0	1	0	1
**6.080**	0	0	0	0	1	0	0	0	0	1	0	1	0	0	0	0	0	0	0	1	0	0	1	0	1	1
**6.081**	0	0	0	0	1	0	0	0	0	1	0	0	1	0	0	0	0	0	0	1	0	0	1	0	0	1
**6.082**	0	0	0	0	1	0	0	0	0	1	0	0	0	0	0	0	0	0	0	1	0	0	0	1	1	1
**6.083**	0	0	0	0	1	0	0	0	0	0	0	1	1	0	0	0	0	0	0	1	0	1	1	0	0	1
**6.084**	0	0	0	0	0	0	1	0	1	1	0	0	0	0	0	0	0	0	0	1	0	1	0	0	0	1
**6.085**	0	0	0	0	0	0	1	0	0	1	0	1	1	0	0	0	0	0	0	1	0	0	1	0	0	1
**6.086**	0	0	0	0	0	0	1	0	0	1	0	0	1	1	0	0	0	0	0	1	0	0	0	1	0	1
**6.087**	0	0	0	0	0	0	1	0	0	1	0	0	0	0	0	0	0	0	0	1	0	1	1	0	0	1
**6.088**	0	0	0	0	0	0	1	0	0	1	0	0	0	0	0	0	0	0	0	1	0	0	1	1	0	1
**6.089**	0	0	0	0	0	0	1	0	0	1	0	0	0	0	0	0	0	0	0	0	0	1	0	1	0	1
**6.090**	0	0	0	0	0	0	1	0	0	0	0	1	0	0	0	1	0	0	0	1	0	0	1	1	1	1
**6.091**	0	0	0	0	0	0	1	0	0	0	0	0	0	0	0	1	0	0	0	1	0	1	0	0	0	1
**6.092**	0	0	0	0	0	0	1	0	0	0	0	0	0	0	0	1	0	0	0	1	0	0	1	1	1	1
**6.093**	0	0	0	0	0	0	0	0	1	1	0	1	1	1	0	0	0	0	0	1	0	1	1	0	0	1
**6.094**	0	0	0	0	0	0	0	0	1	1	0	1	1	0	0	0	0	0	0	1	0	1	1	0	0	1
**6.095**	0	0	0	0	0	0	0	0	1	1	0	1	0	0	0	0	0	0	0	1	0	0	1	1	0	1
**6.096**	0	0	0	0	0	0	0	0	1	0	0	1	0	0	0	0	0	0	0	1	0	1	1	1	0	1
**6.097**	0	0	0	0	0	0	0	0	1	0	0	0	1	1	0	0	0	0	0	0	0	1	0	1	1	1
**6.098**	0	0	0	0	0	0	0	0	1	0	0	0	1	1	0	0	0	0	0	0	0	1	0	1	0	1

CMP	CD2	CD3	CD4	CD7	CD8	CD10	CD13	CD18	CD26	CD29	CD36	CD44	CD45RA	CD49f	CD54	CD56	CD57	CD58	CD62L	CD71	CD80	Cytokeratin	Histone_H1	HLA-DQ	HLA-DR	Freq. (voxel)
**6.099**	0	0	0	0	0	0	0	0	1	0	0	0	0	0	0	1	0	0	0	0	0	1	0	1	1	1
**6.100**	0	0	0	0	0	0	0	0	0	1	0	1	1	1	0	0	0	0	0	1	0	1	0	1	0	1
**6.101**	0	0	0	0	0	0	0	0	0	1	0	1	1	1	0	0	0	0	0	1	0	1	0	0	0	1
**6.102**	0	0	0	0	0	0	0	0	0	1	0	1	1	0	0	1	0	0	0	0	0	0	0	1	0	1
**6.103**	0	0	0	0	0	0	0	0	0	1	0	1	1	0	0	0	0	0	0	1	0	1	0	1	0	1
**6.104**	0	0	0	0	0	0	0	0	0	1	0	0	1	1	0	0	0	0	0	0	0	0	1	0	0	1
**6.105**	0	0	0	0	0	0	0	0	0	1	0	0	1	0	0	1	0	0	0	0	0	0	0	1	1	1
**6.106**	0	0	0	0	0	0	0	0	0	1	0	0	1	0	0	0	0	0	0	1	0	1	1	0	0	1
**6.107**	0	0	0	0	0	0	0	0	0	0	0	1	1	1	0	0	0	0	0	0	0	1	0	0	1	1
**6.108**	0	0	0	0	0	0	0	0	0	0	0	1	1	0	0	1	0	0	0	0	0	1	0	1	1	1
**6.109**	0	0	0	0	0	0	0	0	0	0	0	1	1	0	0	0	0	0	0	1	0	1	1	0	1	1
**6.110**	0	0	0	0	0	0	0	0	0	0	0	0	1	1	0	0	0	0	0	1	0	1	0	1	0	1
**6.111**	0	0	0	0	0	0	0	0	0	0	0	0	1	1	0	0	0	0	0	1	0	0	0	0	1	1
**6.112**	0	0	0	0	0	0	0	0	0	0	0	0	1	0	0	1	0	0	0	1	0	1	0	0	1	1
**6.113**	0	0	0	0	0	0	0	0	0	0	0	0	1	0	0	1	0	0	0	1	0	0	0	0	1	1
**6.114**	0	1	1	1	1	0	0	0	1	1	0	1	1	1	0	0	0	0	0	1	0	1	0	0	0	1
**6.115**	0	1	1	1	1	0	0	0	1	1	0	1	0	0	0	0	0	0	0	1	0	1	0	0	1	1
**6.116**	0	1	1	1	1	0	0	0	1	1	0	1	0	0	0	0	0	0	0	0	0	0	1	0	0	1
**6.117**	0	1	1	1	1	0	0	0	1	0	0	1	1	0	0	1	0	0	0	1	0	0	0	1	1	1
**6.118**	0	1	1	1	1	0	0	0	0	1	0	1	0	1	0	0	0	0	0	1	0	0	1	0	0	1
**6.119**	0	1	1	1	1	0	0	0	0	1	0	0	1	0	0	0	0	0	0	0	0	0	0	1	1	1
**6.120**	0	1	1	1	1	0	0	0	0	0	0	0	0	1	0	0	0	0	0	1	0	0	0	0	0	1
**6.121**	0	1	1	1	0	0	1	0	1	0	0	1	1	0	0	0	0	0	0	1	0	0	0	0	0	1
**6.122**	0	1	1	1	0	0	0	0	1	1	0	1	1	1	0	0	0	0	0	1	0	0	0	1	0	1
**6.123**	0	1	1	1	0	0	0	0	1	0	0	1	0	0	0	0	0	0	0	1	0	1	0	1	1	1
**6.124**	0	1	1	1	0	0	0	0	0	1	0	0	1	0	0	0	0	0	0	0	0	0	1	1	0	1
**6.125**	0	1	1	1	0	0	0	0	0	1	0	0	0	0	0	0	0	0	0	1	0	0	0	0	0	1
**6.126**	0	1	1	1	0	0	0	0	0	0	0	0	0	0	0	0	0	0	0	0	0	0	1	0	0	1
**6.127**	0	1	1	0	1	0	0	0	1	1	0	1	1	0	0	0	0	0	0	1	0	1	0	1	0	1
**6.128**	0	1	1	0	1	0	0	0	1	0	0	1	1	0	0	1	0	0	0	1	0	0	0	1	1	1
**6.129**	0	1	1	0	1	0	0	0	0	1	0	1	1	0	0	0	0	0	0	1	0	0	1	0	0	1
**6.130**	0	1	1	0	1	0	0	0	0	1	0	1	0	1	0	1	0	0	0	1	0	0	0	0	0	1
**6.131**	0	1	1	0	1	0	0	0	0	1	0	1	0	0	0	0	0	0	0	0	0	1	0	1	1	1
**6.132**	0	1	1	0	1	0	0	0	0	0	0	1	1	0	0	0	0	0	0	1	0	1	1	0	1	1
**6.133**	0	1	1	0	1	0	0	0	0	0	0	1	0	0	0	0	0	0	0	1	0	1	1	1	0	1
**6.134**	0	1	1	0	0	0	1	0	0	0	0	1	0	0	0	0	0	0	0	1	0	0	0	0	1	1
**6.135**	0	1	1	0	0	0	1	0	0	0	0	1	0	0	0	0	0	0	0	0	0	0	0	0	1	1
**6.136**	0	1	1	0	0	0	0	0	1	1	0	1	1	1	0	0	0	0	0	1	0	0	0	1	1	1
**6.137**	0	1	1	0	0	0	0	0	1	1	0	1	1	0	0	0	0	0	0	1	0	0	0	1	1	1
**6.138**	0	1	1	0	0	0	0	0	1	0	0	1	1	0	0	0	0	0	0	1	0	0	0	0	1	1
**6.139**	0	1	1	0	0	0	0	0	1	0	0	1	0	0	0	0	0	0	0	0	0	0	1	1	1	1
**6.140**	0	1	1	0	0	0	0	0	0	1	0	1	1	1	0	0	0	0	0	1	0	0	1	0	0	1
**6.141**	0	1	0	1	1	0	1	0	1	0	0	1	0	0	0	0	0	0	0	1	0	1	0	1	1	1
**6.142**	0	1	0	1	1	0	1	0	0	1	0	0	0	0	0	0	0	0	0	1	0	0	0	0	0	1
**6.143**	0	1	0	1	1	0	1	0	0	0	0	0	1	0	0	0	0	0	0	1	0	0	0	0	0	1
**6.144**	0	1	0	1	1	0	1	0	0	0	0	0	0	0	0	0	0	0	0	1	0	0	0	0	0	1
**6.145**	0	1	0	1	1	0	0	0	1	1	0	1	1	0	0	0	0	0	0	0	0	1	1	0	0	1
**6.146**	0	1	0	1	1	0	0	0	1	1	0	1	0	1	0	0	0	0	0	0	0	1	0	0	1	1
**6.147**	0	1	0	1	1	0	0	0	1	1	0	1	0	0	0	0	0	0	0	1	0	0	1	1	1	1
**6.148**	0	1	0	1	1	0	0	0	1	0	0	1	1	1	0	0	0	0	0	1	0	0	1	1	0	1
**6.149**	0	1	0	1	1	0	0	0	1	0	0	1	1	0	0	0	0	0	0	1	0	1	1	1	0	1
**6.150**	0	1	0	1	1	0	0	0	1	0	0	1	0	0	0	1	0	0	0	1	0	1	0	1	1	1
**6.151**	0	1	0	1	1	0	0	0	0	1	1	1	0	0	0	0	0	0	0	0	0	0	0	1	0	1
**6.152**	0	1	0	1	1	0	0	0	0	1	0	1	1	0	0	0	0	0	0	0	0	1	0	1	0	1
**6.153**	0	1	0	1	1	0	0	0	0	1	0	1	0	0	0	1	0	0	0	1	0	0	0	0	0	1
**6.154**	0	1	0	1	1	0	0	0	0	1	0	0	1	0	0	0	0	0	0	0	0	0	0	0	1	1
**6.155**	0	1	0	1	1	0	0	0	0	1	0	0	0	0	0	0	0	0	0	0	0	0	0	0	1	1
**6.156**	0	1	0	1	1	0	0	0	0	0	0	1	1	0	0	0	0	0	0	1	0	0	1	1	1	1
**6.157**	0	1	0	1	1	0	0	0	0	0	0	1	0	1	0	0	0	0	0	1	0	0	0	1	0	1
**6.158**	0	1	0	1	1	0	0	0	0	0	0	0	1	0	0	0	0	0	0	0	0	0	1	1	0	1
**6.159**	0	1	0	1	0	0	1	0	1	1	0	0	0	0	0	0	0	0	0	1	0	0	1	0	0	1
**6.160**	0	1	0	1	0	0	1	0	1	1	0	0	0	0	0	0	0	0	0	1	0	0	0	0	0	1
**6.161**	0	1	0	1	0	0	1	0	1	0	0	1	1	0	0	0	0	0	0	1	0	0	0	0	0	1
**6.162**	0	1	0	1	0	0	1	0	1	0	0	1	0	1	0	0	0	0	0	1	0	1	0	1	1	1
**6.163**	0	1	0	1	0	0	1	0	1	0	0	1	0	0	0	0	0	0	0	1	0	0	1	0	1	1
**6.164**	0	1	0	1	0	0	1	0	1	0	0	1	0	0	0	0	0	0	0	0	0	0	1	1	1	1
**6.165**	0	1	0	1	0	0	1	0	1	0	0	1	0	0	0	0	0	0	0	0	0	0	1	0	1	1
**6.166**	0	1	0	1	0	0	1	0	0	1	0	0	0	1	0	0	0	0	0	1	0	0	0	0	0	1
**6.167**	0	1	0	1	0	0	1	0	0	0	0	1	1	1	0	0	0	0	0	1	0	1	1	1	1	1
**6.168**	0	1	0	1	0	0	1	0	0	0	0	1	1	1	0	0	0	0	0	1	0	1	0	1	0	1
**6.169**	0	1	0	1	0	0	1	0	0	0	0	1	1	0	0	0	0	0	0	1	0	0	0	0	1	1
**6.170**	0	1	0	1	0	0	1	0	0	0	0	1	0	0	0	1	0	0	0	1	0	0	0	1	1	1
**6.171**	0	1	0	1	0	0	1	0	0	0	0	1	0	0	0	0	0	0	0	1	0	0	1	0	1	1
**6.172**	0	1	0	1	0	0	0	0	1	1	0	1	0	0	0	0	0	0	0	1	0	0	1	1	0	1
**6.173**	0	1	0	1	0	0	0	0	1	1	0	0	1	0	0	0	0	0	0	1	0	0	0	0	0	1
**6.174**	0	1	0	1	0	0	0	0	1	0	0	1	1	0	0	0	0	0	0	1	0	1	0	1	1	1
**6.175**	0	1	0	1	0	0	0	0	1	0	0	1	1	0	0	0	0	0	0	1	0	1	0	1	0	1
**6.176**	0	1	0	1	0	0	0	0	1	0	0	1	1	0	0	0	0	0	0	0	0	0	1	1	0	1
**6.177**	0	1	0	1	0	0	0	0	0	1	0	1	1	1	0	0	0	0	0	1	0	0	1	0	0	1
**6.178**	0	1	0	1	0	0	0	0	0	1	0	0	1	0	0	0	0	0	0	0	0	0	1	0	1	1
**6.179**	0	1	0	1	0	0	0	0	0	1	0	0	0	1	0	0	0	0	0	1	0	0	0	0	0	1
**6.180**	0	1	0	1	0	0	0	0	0	0	0	1	1	0	0	0	0	0	0	1	0	0	0	1	1	1
**6.181**	0	1	0	1	0	0	0	0	0	0	0	1	1	0	0	0	0	0	0	0	0	0	1	1	0	1
**6.182**	0	1	0	0	1	0	1	0	1	0	0	1	0	0	0	0	0	0	0	1	0	0	1	0	1	1
**6.183**	0	1	0	0	1	0	1	0	0	1	0	1	0	0	0	0	0	0	0	0	0	0	1	0	1	1
**6.184**	0	1	0	0	1	0	0	0	1	1	0	1	1	1	0	0	0	0	0	0	0	0	1	0	1	1
**6.185**	0	1	0	0	1	0	0	0	1	1	0	1	0	1	0	0	0	0	0	0	0	1	0	1	1	1
**6.186**	0	1	0	0	1	0	0	0	1	1	0	1	0	1	0	0	0	0	0	0	0	1	0	0	1	1
**6.187**	0	1	0	0	1	0	0	0	1	0	1	1	0	0	0	0	0	0	0	0	0	0	1	1	0	1
**6.188**	0	1	0	0	1	0	0	0	1	0	0	1	1	1	0	0	0	0	0	1	0	0	1	0	0	1
**6.189**	0	1	0	0	1	0	0	0	1	0	0	1	1	1	0	0	0	0	0	0	0	0	0	0	1	1
**6.190**	0	1	0	0	1	0	0	0	1	0	0	1	0	0	0	0	0	0	0	1	0	1	0	1	0	1
**6.191**	0	1	0	0	1	0	0	0	0	1	1	1	0	0	0	0	0	0	0	0	0	0	0	1	0	1
**6.192**	0	1	0	0	1	0	0	0	0	1	0	1	1	0	0	0	0	0	0	1	0	1	1	1	0	1
**6.193**	0	1	0	0	1	0	0	0	0	0	0	1	1	1	0	0	0	0	0	1	0	1	0	1	1	1
**6.194**	0	1	0	0	0	0	1	0	1	0	0	1	0	0	0	0	0	0	0	1	0	0	1	0	1	1
**6.195**	0	1	0	0	0	0	1	0	1	0	0	0	0	0	0	0	0	0	0	1	0	0	1	0	1	1
**6.196**	0	1	0	0	0	0	1	0	0	1	0	1	0	1	0	0	0	0	0	1	0	0	0	0	0	1
**6.197**	0	1	0	0	0	0	1	0	0	0	0	1	0	0	0	1	0	0	0	1	0	0	1	1	1	1
**6.198**	0	1	0	0	0	0	1	0	0	0	0	1	0	0	0	0	0	0	0	0	0	0	1	1	1	1
**6.199**	0	1	0	0	0	0	1	0	0	0	0	0	0	0	0	0	0	0	0	0	0	0	1	1	1	1
**6.200**	0	1	0	0	0	0	0	0	1	1	0	1	1	1	0	0	0	0	0	1	0	0	1	1	0	1
**6.201**	0	1	0	0	0	0	0	0	1	1	0	1	1	1	0	0	0	0	0	0	0	0	1	0	0	1
**6.202**	0	1	0	0	0	0	0	0	1	1	0	1	1	0	0	0	0	0	0	1	0	0	1	1	0	1
**6.203**	0	1	0	0	0	0	0	0	1	1	0	1	1	0	0	0	0	0	0	1	0	0	0	1	0	1
**6.204**	0	1	0	0	0	0	0	0	1	1	0	0	0	1	0	0	0	0	0	0	0	0	1	0	0	1
**6.205**	0	1	0	0	0	0	0	0	1	0	0	0	0	1	0	0	0	0	0	1	0	1	0	1	1	1
**6.206**	0	1	0	0	0	0	0	0	1	0	0	0	0	0	0	0	0	0	0	0	0	0	1	1	1	1
**6.207**	0	1	0	0	0	0	0	0	0	1	0	1	1	1	0	0	0	0	0	1	0	1	1	0	0	1
**6.208**	0	1	0	0	0	0	0	0	0	1	0	1	1	1	0	0	0	0	0	0	0	1	1	0	0	1
**6.209**	0	1	0	0	0	0	0	0	0	1	0	1	1	0	0	0	0	0	0	1	0	1	1	1	0	1
**6.210**	0	1	0	0	0	0	0	0	0	1	0	0	1	1	0	0	0	0	0	0	0	0	1	0	0	1
**6.211**	0	1	0	0	0	0	0	0	0	1	0	0	1	0	0	1	0	0	0	0	0	0	1	0	0	1
**6.212**	0	1	0	0	0	0	0	0	0	0	0	1	1	1	0	0	0	0	0	1	0	0	1	0	0	1
**6.213**	0	0	1	1	0	0	1	0	1	1	0	1	1	1	0	1	0	0	0	0	0	0	0	1	1	1
**6.214**	0	0	1	1	0	0	0	0	1	0	0	1	0	1	0	0	0	0	0	0	0	0	0	0	0	1
**6.215**	0	0	1	1	0	0	0	0	0	1	0	1	1	0	0	0	0	0	0	1	0	0	0	1	1	1
**6.216**	0	0	1	1	0	0	0	0	0	1	0	1	0	1	0	0	0	0	0	1	0	1	0	0	1	1
**6.217**	0	0	1	1	0	0	0	0	0	1	0	1	0	0	0	0	0	0	0	0	0	1	0	0	0	1
**6.218**	0	0	1	1	0	0	0	0	0	0	0	0	1	1	0	1	0	0	0	1	0	1	0	0	1	1
**6.219**	0	0	1	1	0	0	0	0	0	0	0	0	1	0	0	1	0	0	0	1	0	1	0	0	0	1
**6.220**	0	0	1	0	1	0	0	0	1	1	0	1	0	1	0	0	0	0	0	1	0	0	0	0	0	1

CMP	CD2	CD3	CD4	CD7	CD8	CD10	CD13	CD18	CD26	CD29	CD36	CD44	CD45RA	CD49f	CD54	CD56	CD57	CD58	CD62L	CD71	CD80	Cytokeratin	Histone_H1	HLA-DQ	HLA-DR	Freq. (voxel)
**6.221**	0	0	1	0	1	0	0	0	1	0	0	0	0	0	0	0	0	0	0	1	0	0	0	0	1	1
**6.222**	0	0	1	0	1	0	0	0	0	1	0	1	0	1	0	0	0	0	0	1	0	1	0	1	1	1
**6.223**	0	0	1	0	1	0	0	0	0	1	0	1	0	1	0	0	0	0	0	1	0	0	0	0	0	1
**6.224**	0	0	1	0	1	0	0	0	0	1	0	1	0	0	0	0	0	0	0	0	0	0	1	0	0	1
**6.225**	0	0	1	0	1	0	0	0	0	0	0	1	0	0	0	0	0	0	0	0	0	0	0	0	1	1
**6.226**	0	0	1	0	0	0	1	0	1	1	0	1	0	0	0	1	0	0	0	0	0	0	0	1	1	1
**6.227**	0	0	1	0	0	0	1	0	0	1	0	0	0	0	0	0	0	0	0	1	0	1	1	0	0	1
**6.228**	0	0	1	0	0	0	1	0	0	0	0	1	1	0	0	0	0	0	0	1	0	1	0	0	0	1
**6.229**	0	0	1	0	0	0	1	0	0	0	0	1	0	0	0	0	0	0	0	1	0	0	1	1	1	1
**6.230**	0	0	1	0	0	0	1	0	0	0	0	1	0	0	0	0	0	0	0	0	0	0	1	1	1	1
**6.231**	0	0	1	0	0	0	0	0	1	1	0	1	0	0	0	0	0	0	0	1	0	1	1	0	0	1
**6.232**	0	0	1	0	0	0	0	0	1	0	0	1	1	0	0	0	0	0	0	0	0	0	0	1	0	1
**6.233**	0	0	1	0	0	0	0	0	1	0	0	1	0	0	0	0	0	0	0	0	0	1	1	0	1	1
**6.234**	0	0	1	0	0	0	0	0	1	0	0	1	0	0	0	0	0	0	0	0	0	0	1	0	0	1
**6.235**	0	0	1	0	0	0	0	0	1	0	0	0	0	0	0	1	0	0	0	1	0	1	0	0	0	1
**6.236**	0	0	1	0	0	0	0	0	1	0	0	0	0	0	0	0	0	0	0	1	0	1	1	0	0	1
**6.237**	0	0	1	0	0	0	0	0	0	1	0	0	1	0	0	0	0	0	0	0	0	0	0	1	1	1
**6.238**	0	0	1	0	0	0	0	0	0	0	0	0	1	1	0	0	0	0	0	0	0	0	1	0	0	1
**6.239**	0	0	0	1	1	0	0	0	1	1	0	1	0	0	0	0	0	0	0	1	0	1	1	0	1	1
**6.240**	0	0	0	1	0	0	1	0	1	1	0	1	1	1	0	0	0	0	0	0	0	0	0	1	1	1
**6.241**	0	0	0	1	0	0	1	0	1	1	0	0	1	0	0	0	0	0	0	1	0	0	0	0	0	1
**6.242**	0	0	0	1	0	0	1	0	0	1	0	1	1	1	0	1	0	0	0	0	0	0	0	1	1	1
**6.243**	0	0	0	1	0	0	1	0	0	0	0	1	0	0	0	0	0	0	0	0	0	0	1	1	0	1
**6.244**	0	0	0	1	0	0	0	0	1	1	0	1	0	0	0	0	0	0	0	0	0	0	0	0	1	1
**6.245**	0	0	0	1	0	0	0	0	1	1	0	0	1	0	0	0	0	0	0	1	0	0	1	0	0	1
**6.246**	0	0	0	1	0	0	0	0	1	0	0	1	0	1	0	0	0	0	0	1	0	1	0	1	0	1
**6.247**	0	0	0	1	0	0	0	0	0	1	1	1	0	0	0	0	0	0	0	0	0	0	0	1	1	1
**6.248**	0	0	0	1	0	0	0	0	0	1	0	1	1	1	0	1	0	0	0	1	0	1	1	0	0	1
**6.249**	0	0	0	1	0	0	0	0	0	1	0	1	1	1	0	1	0	0	0	0	0	1	1	1	0	1
**6.250**	0	0	0	1	0	0	0	0	0	1	0	1	0	1	0	0	0	0	0	1	0	1	0	1	0	1
**6.251**	0	0	0	1	0	0	0	0	0	1	0	0	1	0	0	1	0	0	0	1	0	1	0	0	0	1
**6.252**	0	0	0	1	0	0	0	0	0	1	0	0	1	0	0	0	0	0	0	0	0	0	0	1	1	1
**6.253**	0	0	0	1	0	0	0	0	0	0	0	1	1	0	0	0	0	0	0	1	0	0	0	1	0	1
**6.254**	0	0	0	1	0	0	0	0	0	0	0	1	0	0	0	1	0	0	0	0	0	0	0	1	1	1
**6.255**	0	0	0	0	1	0	1	0	1	1	0	1	0	1	0	0	0	0	0	0	0	1	0	1	1	1
**6.256**	0	0	0	0	1	0	1	0	0	0	0	0	0	0	0	0	0	0	0	0	0	0	0	1	1	1
**6.257**	0	0	0	0	1	0	0	0	1	1	0	1	0	1	0	0	0	0	0	0	0	1	1	0	1	1
**6.258**	0	0	0	0	1	0	0	0	1	1	0	1	0	1	0	0	0	0	0	0	0	1	1	0	0	1
**6.259**	0	0	0	0	1	0	0	0	0	1	0	1	1	1	0	0	0	0	0	0	0	0	1	0	0	1
**6.260**	0	0	0	0	1	0	0	0	0	0	0	0	0	0	0	0	0	0	0	0	0	1	1	1	1	1
**6.261**	0	0	0	0	0	0	1	0	0	1	0	1	1	1	0	0	0	0	0	1	0	0	0	1	0	1
**6.262**	0	0	0	0	0	0	1	0	0	1	0	0	1	0	0	1	0	0	0	0	0	1	0	0	0	1
**6.263**	0	0	0	0	0	0	1	0	0	1	0	0	1	0	0	0	0	0	0	1	0	1	0	0	0	1
**6.264**	0	0	0	0	0	0	1	0	0	0	0	1	1	0	0	0	0	0	0	1	0	0	0	0	1	1
**6.265**	0	0	0	0	0	0	0	0	0	1	1	1	0	1	0	0	0	0	0	1	0	1	1	1	1	1
**6.266**	0	0	0	0	0	0	0	0	0	1	1	1	0	1	0	0	0	0	0	1	0	1	1	1	0	1
**6.267**	0	0	0	0	0	0	0	0	0	1	0	1	1	0	0	0	0	0	0	1	0	0	0	1	1	1
**6.268**	0	0	0	0	0	0	0	0	0	1	0	0	1	1	0	0	0	0	0	1	0	0	0	0	0	1
**6.269**	0	0	0	0	0	0	0	0	0	0	0	0	1	0	0	0	0	0	0	0	0	1	1	1	0	1
**6.270**	0	0	0	0	0	0	0	0	0	0	0	0	0	1	0	0	0	0	0	1	0	1	1	1	1	1
**6.271**	0	1	1	1	1	0	0	0	1	1	0	1	1	1	0	0	0	0	0	0	0	1	0	0	0	1
**6.272**	0	1	1	1	1	0	0	0	0	1	0	0	1	0	0	0	0	0	0	1	0	0	1	1	1	1
**6.273**	0	1	1	1	1	0	0	0	0	1	0	0	1	0	0	0	0	0	0	0	0	0	1	1	1	1
**6.274**	0	1	1	1	1	0	0	0	0	0	0	1	1	0	0	0	0	0	0	1	0	1	0	1	0	1
**6.275**	0	1	1	1	1	0	0	0	0	0	0	0	1	0	0	0	0	0	0	1	0	0	0	1	1	1
**6.276**	0	1	1	1	0	0	0	0	0	1	0	1	0	0	0	0	0	0	0	1	0	0	1	0	0	1
**6.277**	0	1	1	1	0	0	0	0	0	0	0	1	0	1	0	0	0	0	0	1	0	0	0	0	1	1
**6.278**	0	1	1	1	0	0	0	0	0	0	0	1	0	0	0	0	0	0	0	0	0	0	0	0	1	1
**6.279**	0	1	1	0	1	0	0	0	1	1	0	1	1	1	0	0	0	0	0	1	0	1	0	0	0	1
**6.280**	0	1	1	0	1	0	0	0	1	1	0	1	1	1	0	0	0	0	0	0	0	1	0	0	0	1
**6.281**	0	1	1	0	1	0	0	0	0	0	0	1	1	0	0	0	0	0	0	0	0	1	0	0	0	1
**6.282**	0	1	1	0	1	0	0	0	0	0	0	1	0	0	0	0	0	0	0	1	0	1	1	1	1	1
**6.283**	0	1	1	0	0	0	1	0	1	0	0	1	0	0	0	0	0	0	0	0	0	0	0	1	1	1
**6.284**	0	1	1	0	0	0	1	0	0	1	0	1	1	0	0	0	0	0	0	1	0	0	1	0	0	1
**6.285**	0	1	1	0	0	0	0	0	1	1	0	1	1	0	0	0	0	0	0	0	0	1	1	0	0	1
**6.286**	0	1	1	0	0	0	0	0	0	1	0	1	1	0	0	0	0	0	0	0	0	0	1	0	0	1
**6.287**	0	1	1	0	0	0	0	0	0	0	0	1	0	0	0	0	0	0	0	1	0	1	0	1	0	1
**6.288**	0	1	0	1	1	0	1	0	0	1	0	1	0	0	0	0	0	0	0	1	0	0	0	1	0	1
**6.289**	0	1	0	1	1	0	0	0	1	1	0	1	1	1	0	0	0	0	0	1	0	0	0	1	0	1
**6.290**	0	1	0	1	1	0	0	0	1	1	0	0	1	0	0	0	0	0	0	1	0	0	1	0	0	1
**6.291**	0	1	0	1	1	0	0	0	1	0	0	0	1	0	0	0	0	0	0	1	0	0	0	0	0	1
**6.292**	0	1	0	1	1	0	0	0	1	0	0	0	0	0	0	0	0	0	0	1	0	1	1	1	1	1
**6.293**	0	1	0	1	1	0	0	0	0	1	0	1	1	1	0	0	0	0	0	0	0	1	0	0	1	1
**6.294**	0	1	0	1	1	0	0	0	0	1	0	1	1	1	0	0	0	0	0	0	0	1	0	0	0	1
**6.295**	0	1	0	1	1	0	0	0	0	1	0	0	1	0	0	0	0	0	0	1	0	0	1	1	1	1
**6.296**	0	1	0	1	1	0	0	0	0	1	0	0	1	0	0	0	0	0	0	0	0	0	1	1	0	1
**6.297**	0	1	0	1	1	0	0	0	0	0	0	1	1	1	0	0	0	0	0	1	0	0	0	0	1	1
**6.298**	0	1	0	1	1	0	0	0	0	0	0	0	0	1	0	0	0	0	0	1	0	0	0	1	1	1
**6.299**	0	1	0	1	0	0	1	0	0	0	0	1	1	0	0	0	0	0	0	1	0	0	1	0	0	1
**6.300**	0	1	0	1	0	0	1	0	0	0	0	0	1	1	0	0	0	0	0	1	0	1	1	1	1	1
**6.301**	0	1	0	1	0	0	1	0	0	0	0	0	1	1	0	0	0	0	0	1	0	1	0	1	1	1
**6.302**	0	1	0	1	0	0	1	0	0	0	0	0	0	1	0	0	0	0	0	0	0	1	1	1	1	1
**6.303**	0	1	0	1	0	0	0	0	1	1	0	1	1	1	0	0	0	0	0	1	0	0	1	0	0	1
**6.304**	0	1	0	1	0	0	0	0	1	1	0	1	1	0	0	0	0	0	0	1	0	1	0	0	0	1
**6.305**	0	1	0	1	0	0	0	0	0	1	1	1	0	1	0	0	0	0	0	1	0	1	0	1	1	1
**6.306**	0	1	0	1	0	0	0	0	0	1	0	1	1	1	0	1	0	0	0	1	0	0	0	0	0	1
**6.307**	0	1	0	1	0	0	0	0	0	1	0	1	0	0	0	0	0	0	0	1	0	1	0	1	1	1
**6.308**	0	1	0	1	0	0	0	0	0	1	0	0	1	0	0	0	0	0	0	0	0	0	1	1	0	1
**6.309**	0	1	0	1	0	0	0	0	0	1	0	0	0	1	0	0	0	0	0	1	0	0	1	0	0	1
**6.310**	0	1	0	1	0	0	0	0	0	0	0	1	0	1	0	0	0	0	0	1	0	1	0	1	0	1
**6.311**	0	1	0	1	0	0	0	0	0	0	0	0	0	1	0	0	0	0	0	1	0	0	0	0	1	1
**6.312**	0	1	0	1	0	0	0	0	0	0	0	0	0	0	0	0	0	0	0	0	0	1	1	0	1	1
**6.313**	0	1	0	0	1	0	1	0	1	1	0	1	0	1	0	0	0	0	0	1	0	0	0	1	0	1
**6.314**	0	1	0	0	1	0	1	0	1	0	0	1	1	0	0	0	0	0	0	1	0	0	0	0	1	1
**6.315**	0	1	0	0	1	0	1	0	0	1	0	1	0	0	0	0	0	0	0	0	0	1	0	1	1	1
**6.316**	0	1	0	0	1	0	0	0	1	1	0	1	1	1	0	0	0	0	0	1	0	0	0	0	1	1
**6.317**	0	1	0	0	1	0	0	0	1	1	0	1	0	1	0	0	0	0	0	1	0	0	0	1	0	1
**6.318**	0	1	0	0	1	0	0	0	1	0	0	1	1	0	0	0	0	0	0	1	0	1	0	1	0	1
**6.319**	0	1	0	0	1	0	0	0	0	0	0	1	1	0	0	0	0	0	0	1	0	0	1	1	0	1
**6.320**	0	1	0	0	1	0	0	0	0	0	0	0	0	1	0	0	0	0	0	1	0	1	0	1	1	1
**6.321**	0	1	0	0	0	0	1	0	0	1	0	1	0	1	0	1	0	0	0	1	0	0	1	0	0	1
**6.322**	0	1	0	0	0	0	1	0	0	0	0	1	0	1	0	0	0	0	0	0	0	1	0	1	1	1
**6.323**	0	1	0	0	0	0	1	0	0	0	0	1	0	1	0	0	0	0	0	0	0	0	0	0	1	1
**6.324**	0	1	0	0	0	0	1	0	0	0	0	1	0	0	0	0	0	0	0	0	0	1	0	0	1	1
**6.325**	0	1	0	0	0	0	0	0	1	1	0	1	1	0	0	0	0	0	0	0	0	1	0	1	0	1
**6.326**	0	1	0	0	0	0	0	0	1	0	0	1	1	1	0	0	0	0	0	0	0	1	0	1	1	1
**6.327**	0	1	0	0	0	0	0	0	0	1	0	1	1	1	0	1	0	0	0	1	0	0	1	0	0	1
**6.328**	0	1	0	0	0	0	0	0	0	1	0	1	1	1	0	1	0	0	0	1	0	0	0	0	0	1
**6.329**	0	1	0	0	0	0	0	0	0	1	0	1	0	1	0	1	0	0	0	1	0	0	1	0	0	1
**6.330**	0	1	0	0	0	0	0	0	0	1	0	1	0	1	0	0	0	0	0	1	0	1	1	1	1	1
**6.331**	0	1	0	0	0	0	0	0	0	1	0	1	0	1	0	0	0	0	0	0	0	1	1	1	1	1
**6.332**	0	1	0	0	0	0	0	0	0	1	0	1	0	1	0	0	0	0	0	0	0	1	0	1	0	1
**6.333**	0	1	0	0	0	0	0	0	0	1	0	0	0	0	0	1	0	0	0	0	0	0	1	0	0	1
**6.334**	0	1	0	0	0	0	0	0	0	0	0	1	1	1	0	0	0	0	0	1	0	1	0	1	1	1
**6.335**	0	1	0	0	0	0	0	0	0	0	0	1	1	1	0	0	0	0	0	0	0	1	1	1	0	1
**6.336**	0	0	1	1	1	0	0	0	1	1	0	1	0	0	0	0	0	0	0	1	0	1	0	0	1	1
**6.337**	0	0	1	1	0	0	1	0	1	0	0	1	0	1	0	0	0	0	0	1	0	1	0	0	0	1
**6.338**	0	0	1	1	0	0	0	0	1	1	0	1	0	0	0	0	0	0	0	1	0	1	0	0	1	1
**6.339**	0	0	1	1	0	0	0	0	0	1	0	1	0	1	0	0	0	0	0	1	0	0	1	0	0	1
**6.340**	0	0	1	1	0	0	0	0	0	1	0	1	0	1	0	0	0	0	0	1	0	0	0	0	0	1
**6.341**	0	0	1	1	0	0	0	0	0	1	0	1	0	0	0	0	0	0	0	1	0	1	0	0	1	1
**6.342**	0	0	1	1	0	0	0	0	0	0	0	1	0	1	0	0	0	0	0	0	0	0	0	0	0	1

CMP	CD2	CD3	CD4	CD7	CD8	CD10	CD13	CD18	CD26	CD29	CD36	CD44	CD45RA	CD49f	CD54	CD56	CD57	CD58	CD62L	CD71	CD80	Cytokeratin	Histone_H1	HLA-DQ	HLA-DR	Freq. (voxel)
**6.343**	0	0	1	1	0	0	0	0	0	0	0	0	1	1	0	1	0	0	0	1	0	1	0	1	0	1
**6.344**	0	0	1	1	0	0	0	0	0	0	0	0	1	1	0	1	0	0	0	1	0	0	0	1	0	1
**6.345**	0	0	1	1	0	0	0	0	0	0	0	0	0	0	0	0	0	0	0	0	0	0	0	1	1	1
**6.346**	0	0	1	0	1	0	1	0	1	1	0	0	0	0	0	1	0	0	0	0	0	0	0	1	1	1
**6.347**	0	0	1	0	1	0	1	0	1	1	0	0	0	0	0	0	0	0	0	0	0	0	0	1	1	1
**6.348**	0	0	1	0	1	0	1	0	0	0	0	0	1	1	0	1	0	0	0	1	0	1	0	1	0	1
**6.349**	0	0	1	0	1	0	1	0	0	0	0	0	1	1	0	1	0	0	0	1	0	1	0	0	0	1
**6.350**	0	0	1	0	1	0	1	0	0	0	0	0	0	1	0	0	0	0	0	1	0	0	0	1	1	1
**6.351**	0	0	1	0	1	0	0	0	1	0	0	1	0	0	0	0	0	0	0	0	0	0	0	0	0	1
**6.352**	0	0	1	0	1	0	0	0	1	0	0	0	0	0	0	0	0	0	0	1	0	1	0	0	0	1
**6.353**	0	0	1	0	1	0	0	0	0	1	0	1	0	0	0	0	0	0	0	0	0	1	0	1	1	1
**6.354**	0	0	1	0	1	0	0	0	0	0	0	1	0	0	0	0	0	0	0	1	0	0	0	1	0	1
**6.355**	0	0	1	0	0	0	1	0	0	1	0	0	1	1	0	0	0	0	0	0	0	1	1	1	0	1
**6.356**	0	0	1	0	0	0	1	0	0	1	0	0	0	1	0	0	0	0	0	1	0	0	0	1	0	1
**6.357**	0	0	1	0	0	0	1	0	0	0	0	1	0	1	0	0	0	0	0	1	0	0	1	1	1	1
**6.358**	0	0	1	0	0	0	0	0	1	1	0	1	1	1	0	0	0	0	0	1	0	1	0	1	1	1
**6.359**	0	0	1	0	0	0	0	0	1	1	0	1	1	1	0	0	0	0	0	1	0	1	0	1	0	1
**6.360**	0	0	1	0	0	0	0	0	1	0	0	1	0	0	0	0	0	0	0	1	0	1	1	0	1	1
**6.361**	0	0	1	0	0	0	0	0	0	1	0	1	1	0	0	0	0	0	0	1	0	0	0	0	0	1
**6.362**	0	0	1	0	0	0	0	0	0	1	0	0	0	1	0	0	0	0	0	1	0	0	1	1	0	1
**6.363**	0	0	1	0	0	0	0	0	0	0	1	0	1	0	0	0	0	0	0	0	0	1	0	0	0	1
**6.364**	0	0	1	0	0	0	0	0	0	0	1	0	1	0	0	0	0	0	0	0	0	0	0	0	0	1
**6.365**	0	0	1	0	0	0	0	0	0	0	0	1	1	0	0	0	0	0	0	0	0	0	1	0	0	1
**6.366**	0	0	1	0	0	0	0	0	0	0	0	1	1	0	0	0	0	0	0	0	0	0	0	0	1	1
**6.367**	0	0	1	0	0	0	0	0	0	0	0	1	0	1	0	0	0	0	0	1	0	0	1	0	0	1
**6.368**	0	0	0	1	1	0	1	0	1	1	0	1	0	1	0	0	0	0	0	1	0	0	0	1	1	1
**6.369**	0	0	0	1	1	0	0	0	1	0	0	1	1	0	0	0	0	0	0	0	0	1	0	0	1	1
**6.370**	0	0	0	1	1	0	0	0	0	0	0	0	1	0	0	0	0	0	0	1	0	0	0	0	1	1
**6.371**	0	0	0	1	0	0	1	0	1	1	0	1	0	1	0	0	0	0	0	1	0	1	0	0	0	1
**6.372**	0	0	0	1	0	0	1	0	1	0	0	0	1	1	0	0	0	0	0	1	0	1	0	0	0	1
**6.373**	0	0	0	1	0	0	1	0	0	1	0	1	0	1	0	0	0	0	0	0	0	0	1	1	1	1
**6.374**	0	0	0	1	0	0	1	0	0	1	0	1	0	0	0	0	0	0	0	1	0	0	1	0	0	1
**6.375**	0	0	0	1	0	0	1	0	0	1	0	0	0	0	0	0	0	0	0	1	0	0	1	0	0	1
**6.376**	0	0	0	1	0	0	0	0	0	1	0	1	0	1	0	1	0	0	0	1	0	1	1	1	0	1
**6.377**	0	0	0	1	0	0	0	0	0	0	0	0	0	1	0	1	0	0	0	1	0	0	0	1	1	1
**6.378**	0	0	0	1	0	0	0	0	0	0	0	0	0	1	0	0	0	0	0	1	0	1	0	1	1	1
**6.379**	0	0	0	0	1	0	1	0	1	1	0	1	1	1	0	0	0	0	0	1	0	1	0	0	1	1
**6.380**	0	0	0	0	1	0	1	0	1	1	0	0	0	0	0	1	0	0	0	0	0	0	0	1	1	1
**6.381**	0	0	0	0	1	0	1	0	1	0	0	0	0	0	0	0	0	0	0	0	0	0	0	1	1	1
**6.382**	0	0	0	0	1	0	1	0	0	1	0	1	1	1	0	0	0	0	0	1	0	1	0	1	1	1
**6.383**	0	0	0	0	1	0	1	0	0	1	0	1	1	1	0	0	0	0	0	1	0	1	0	0	0	1
**6.384**	0	0	0	0	1	0	0	0	0	1	0	1	1	1	0	0	0	0	0	0	0	1	0	0	1	1
**6.385**	0	0	0	0	1	0	0	0	0	1	0	1	0	1	0	0	0	0	0	1	0	0	0	1	0	1
**6.386**	0	0	0	0	0	0	1	0	1	1	1	1	0	1	0	0	0	0	0	1	0	1	0	0	0	1
**6.387**	0	0	0	0	0	0	1	0	1	1	0	1	0	1	0	0	0	0	0	1	0	0	0	1	0	1
**6.388**	0	0	0	0	0	0	1	0	1	1	0	0	1	1	0	0	0	0	0	0	0	1	0	0	0	1
**6.389**	0	0	0	0	0	0	1	0	1	1	0	0	0	0	0	0	0	0	0	0	0	0	0	1	1	1
**6.390**	0	0	0	0	0	0	1	0	1	0	0	1	0	0	0	0	0	0	0	1	0	0	0	1	0	1
**6.391**	0	0	0	0	0	0	1	0	0	1	1	0	0	0	0	0	0	0	0	0	0	1	0	0	0	1
**6.392**	0	0	0	0	0	0	1	0	0	1	0	1	1	0	0	0	0	0	0	1	0	0	0	0	0	1
**6.393**	0	0	0	0	0	0	1	0	0	1	0	1	0	0	0	0	0	0	0	0	0	1	1	1	1	1
**6.394**	0	0	0	0	0	0	1	0	0	1	0	0	0	1	0	1	0	0	0	0	0	0	0	1	0	1
**6.395**	0	0	0	0	0	0	1	0	0	0	0	0	0	1	0	1	0	0	0	0	0	0	0	1	0	1
**6.396**	0	0	0	0	0	0	0	0	1	1	0	0	0	0	0	1	0	0	0	0	0	0	0	1	1	1
**6.397**	0	0	0	0	0	0	0	0	0	1	1	1	0	1	0	0	0	0	0	1	0	1	0	0	1	1
**6.398**	0	0	0	0	0	0	0	0	0	1	1	1	0	1	0	0	0	0	0	0	0	1	1	1	1	1
**6.399**	0	0	0	0	0	0	0	0	0	0	1	0	1	0	0	0	0	0	0	1	0	1	0	0	0	1
**6.400**	0	0	0	0	0	0	0	0	0	0	0	1	1	0	0	1	0	0	0	0	0	1	1	0	0	1
**6.401**	0	0	0	0	0	0	0	0	0	0	0	1	0	1	0	1	0	0	0	1	0	1	0	1	1	1
**6.402**	0	0	0	0	0	0	0	0	0	0	0	1	0	0	0	1	0	0	0	1	0	0	1	1	0	1
**6.403**	0	0	0	0	0	0	0	0	0	0	0	0	1	0	0	0	0	0	0	1	0	0	1	1	0	1
**6.404**	0	0	0	0	0	0	0	0	0	0	0	0	0	1	0	1	0	0	0	1	0	0	0	1	1	1
**6.405**	0	0	0	0	0	0	0	0	0	0	0	0	0	0	0	1	0	0	0	1	0	1	0	0	1	1
**6.406**	0	0	0	0	0	0	0	0	0	0	0	0	0	0	0	1	0	0	0	1	0	0	1	1	0	1
**6.407**	0	1	1	1	1	0	1	0	0	1	0	1	0	1	0	0	0	0	0	1	0	0	0	1	1	1
**6.408**	0	1	1	1	1	0	0	0	1	1	0	1	1	1	0	1	0	0	0	0	0	0	0	0	0	1
**6.409**	0	1	1	1	1	0	0	0	1	1	0	1	1	0	0	0	0	0	0	1	0	1	0	0	0	1
**6.410**	0	1	1	1	1	0	0	0	0	1	0	1	1	1	0	0	0	0	0	1	0	0	0	1	0	1
**6.411**	0	1	1	1	1	0	0	0	0	1	0	1	1	1	0	0	0	0	0	0	0	0	0	1	1	1
**6.412**	0	1	1	1	1	0	0	0	0	1	0	1	1	1	0	0	0	0	0	0	0	0	0	1	0	1
**6.413**	0	1	1	1	1	0	0	0	0	1	0	1	1	0	0	0	0	0	0	0	0	0	0	1	1	1
**6.414**	0	1	1	1	1	0	0	0	0	1	0	1	1	0	0	0	0	0	0	0	0	0	0	0	1	1
**6.415**	0	1	1	1	1	0	0	0	0	1	0	1	0	1	0	0	0	0	0	1	0	0	0	0	1	1
**6.416**	0	1	1	1	1	0	0	0	0	1	0	1	0	0	0	0	0	0	0	0	0	0	0	1	1	1
**6.417**	0	1	1	1	0	0	1	0	0	1	0	1	0	1	0	0	0	0	0	0	0	0	0	0	1	1
**6.418**	0	1	1	1	0	0	0	0	1	1	0	1	1	1	0	1	0	0	0	0	0	0	0	0	0	1
**6.419**	0	1	1	1	0	0	0	0	1	1	0	1	1	1	0	0	0	0	0	1	0	0	0	1	1	1
**6.420**	0	1	1	1	0	0	0	0	1	0	0	1	1	0	0	0	0	0	0	1	0	0	0	1	1	1
**6.421**	0	1	1	1	0	0	0	0	0	1	0	1	0	1	0	0	0	0	0	0	0	0	0	0	1	1
**6.422**	0	1	1	0	1	0	1	0	0	1	0	0	0	0	0	0	0	0	0	0	0	0	0	1	1	1
**6.423**	0	1	1	0	1	0	0	0	1	1	0	1	0	1	0	0	0	0	0	1	0	0	0	1	0	1
**6.424**	0	1	1	0	1	0	0	0	0	1	0	1	0	1	0	1	0	0	0	1	0	0	0	1	1	1
**6.425**	0	1	1	0	1	0	0	0	0	0	0	1	0	0	0	0	0	0	0	1	0	1	1	0	0	1
**6.426**	0	1	1	0	1	0	0	0	0	0	0	1	0	0	0	0	0	0	0	1	0	1	0	0	1	1
**6.427**	0	1	1	0	1	0	0	0	0	0	0	1	0	0	0	0	0	0	0	1	0	0	1	0	1	1
**6.428**	0	1	1	0	1	0	0	0	0	0	0	1	0	0	0	0	0	0	0	0	0	1	1	0	0	1
**6.429**	0	1	1	0	1	0	0	0	0	0	0	0	1	0	0	0	0	0	0	1	0	0	0	0	0	1
**6.430**	0	1	1	0	0	0	1	0	0	0	0	1	0	1	0	0	0	0	0	0	0	0	1	0	0	1
**6.431**	0	1	1	0	0	0	1	0	0	0	0	1	0	1	0	0	0	0	0	0	0	0	0	0	0	1
**6.432**	0	1	1	0	0	0	0	0	1	1	0	1	1	1	0	0	0	0	0	0	0	0	1	0	0	1
**6.433**	0	1	1	0	0	0	0	0	1	1	0	1	0	0	0	1	0	0	0	0	0	0	0	1	1	1
**6.434**	0	1	1	0	0	0	0	0	1	1	0	1	0	0	0	0	0	0	0	0	0	0	0	1	0	1
**6.435**	0	1	1	0	0	0	0	0	1	0	0	1	0	0	0	1	0	0	0	1	0	1	0	0	1	1
**6.436**	0	1	1	0	0	0	0	0	0	1	0	1	0	1	0	0	0	0	0	1	0	0	0	1	1	1
**6.437**	0	1	1	0	0	0	0	0	0	1	0	1	0	1	0	0	0	0	0	0	0	0	1	0	0	1
**6.438**	0	1	1	0	0	0	0	0	0	0	0	1	0	0	0	0	0	0	0	0	0	1	1	0	0	1
**6.439**	0	1	1	0	0	0	0	0	0	0	0	1	0	0	0	0	0	0	0	0	0	1	0	0	1	1
**6.440**	0	1	0	1	1	0	1	0	0	1	0	1	0	1	0	0	0	0	0	0	0	1	0	1	1	1
**6.441**	0	1	0	1	1	0	0	0	1	1	0	0	1	0	0	0	0	0	0	0	0	0	0	0	0	1
**6.442**	0	1	0	1	1	0	0	0	0	1	0	1	1	1	0	0	0	0	0	0	0	0	0	1	0	1
**6.443**	0	1	0	1	1	0	0	0	0	1	0	1	0	1	0	0	0	0	0	1	0	1	1	1	1	1
**6.444**	0	1	0	1	1	0	0	0	0	0	0	1	0	1	0	0	0	0	0	1	0	1	0	1	0	1
**6.445**	0	1	0	1	1	0	0	0	0	0	0	1	0	1	0	0	0	0	0	0	0	1	0	0	0	1
**6.446**	0	1	0	1	1	0	0	0	0	0	0	0	1	0	0	0	0	0	0	0	0	1	1	1	0	1
**6.447**	0	1	0	1	0	0	1	0	0	1	0	1	0	0	0	0	0	0	0	1	0	0	0	1	1	1
**6.448**	0	1	0	1	0	0	0	0	1	1	0	1	1	1	0	0	0	0	0	1	0	1	0	0	0	1
**6.449**	0	1	0	1	0	0	0	0	0	1	0	0	0	0	0	0	0	0	0	0	0	0	1	1	0	1
**6.450**	0	1	0	1	0	0	0	0	0	0	0	1	1	1	0	0	0	0	0	0	0	0	0	1	1	1
**6.451**	0	1	0	1	0	0	0	0	0	0	0	1	0	1	0	0	0	0	0	0	0	1	0	0	0	1
**6.452**	0	1	0	1	0	0	0	0	0	0	0	0	0	0	0	0	0	0	0	0	0	1	1	1	1	1
**6.453**	0	1	0	0	1	0	1	0	1	1	0	1	0	1	0	0	0	0	0	1	0	1	0	0	0	1
**6.454**	0	1	0	0	1	0	1	0	0	1	0	1	0	1	0	0	0	0	0	1	0	1	0	1	0	1
**6.455**	0	1	0	0	1	0	0	0	0	1	0	1	1	1	0	1	0	0	0	0	0	0	1	0	0	1
**6.456**	0	1	0	0	1	0	0	0	0	1	0	1	1	1	0	0	0	0	0	0	0	0	0	1	0	1
**6.457**	0	1	0	0	1	0	0	0	0	1	0	1	1	0	0	0	0	0	0	1	0	0	1	0	1	1
**6.458**	0	1	0	0	1	0	0	0	0	1	0	0	0	1	0	0	0	0	0	0	0	0	0	0	0	1
**6.459**	0	1	0	0	1	0	0	0	0	0	0	0	1	1	0	0	0	0	0	0	0	0	0	0	0	1
**6.460**	0	1	0	0	1	0	0	0	0	0	0	0	1	0	0	0	0	0	0	1	0	1	1	1	1	1
**6.461**	0	1	0	0	0	0	1	0	0	1	0	1	1	0	0	0	0	0	0	1	0	0	0	0	0	1
**6.462**	0	1	0	0	0	0	1	0	0	1	0	1	0	1	0	0	0	0	0	1	0	1	0	1	0	1
**6.463**	0	1	0	0	0	0	1	0	0	1	0	1	0	0	0	0	0	0	0	0	0	0	1	1	0	1
**6.464**	0	1	0	0	0	0	1	0	0	0	0	1	1	0	0	0	0	0	0	1	0	0	1	0	0	1

CMP	CD2	CD3	CD4	CD7	CD8	CD10	CD13	CD18	CD26	CD29	CD36	CD44	CD45RA	CD49f	CD54	CD56	CD57	CD58	CD62L	CD71	CD80	Cytokeratin	Histone_H1	HLA-DQ	HLA-DR	Freq. (voxel)
**6.465**	0	1	0	0	0	0	0	0	1	1	0	1	1	1	0	1	0	0	0	0	0	0	0	0	0	1
**6.466**	0	1	0	0	0	0	0	0	1	1	0	1	1	1	0	0	0	0	0	0	0	1	0	0	0	1
**6.467**	0	1	0	0	0	0	0	0	1	0	0	1	0	1	0	0	0	0	0	1	0	0	1	0	0	1
**6.468**	0	1	0	0	0	0	0	0	0	1	0	1	1	1	0	0	0	0	0	1	0	1	0	0	0	1
**6.469**	0	1	0	0	0	0	0	0	0	0	1	1	0	0	0	0	0	0	0	1	0	1	0	0	0	1
**6.470**	0	1	0	0	0	0	0	0	0	0	0	1	1	1	0	0	0	0	0	1	0	0	0	0	1	1
**6.471**	0	1	0	0	0	0	0	0	0	0	0	0	1	0	0	0	0	0	0	0	0	1	1	1	1	1
**6.472**	0	1	0	0	0	0	0	0	0	0	0	0	0	0	0	0	0	0	0	1	0	1	0	1	0	1
**6.473**	0	0	1	1	1	0	0	0	0	0	0	1	0	0	0	0	0	0	0	1	0	0	1	0	0	1
**6.474**	0	0	1	1	0	0	0	0	0	1	0	1	0	1	0	0	0	0	0	1	0	1	0	1	0	1
**6.475**	0	0	1	1	0	0	0	0	0	0	0	1	0	0	0	0	0	0	0	1	0	1	0	0	1	1
**6.476**	0	0	1	1	0	0	0	0	0	0	0	0	0	1	0	0	0	0	0	0	0	0	0	1	1	1
**6.477**	0	0	1	1	0	0	0	0	0	0	0	0	0	0	0	0	0	0	0	1	0	1	0	1	0	1
**6.478**	0	0	1	0	1	0	1	0	1	0	0	0	1	0	0	1	0	0	0	1	0	1	0	1	0	1
**6.479**	0	0	1	0	1	0	1	0	0	0	0	0	1	0	0	1	0	0	0	0	0	1	0	1	0	1
**6.480**	0	0	1	0	1	0	1	0	0	0	0	0	1	0	0	0	0	0	0	1	0	0	0	1	1	1
**6.481**	0	0	1	0	1	0	0	0	1	0	0	1	1	0	0	0	0	0	0	1	0	1	0	0	1	1
**6.482**	0	0	1	0	1	0	0	0	1	0	0	0	0	0	0	0	0	0	0	0	0	0	1	0	0	1
**6.483**	0	0	1	0	0	0	1	0	1	1	0	1	0	0	0	0	0	0	0	0	0	1	0	0	0	1
**6.484**	0	0	1	0	0	0	1	0	1	0	1	1	0	1	0	0	0	0	0	1	0	1	0	1	1	1
**6.485**	0	0	1	0	0	0	1	0	1	0	0	0	1	0	0	1	0	0	0	0	0	1	0	1	0	1
**6.486**	0	0	1	0	0	0	1	0	1	0	0	0	1	0	0	0	0	0	0	0	0	0	0	1	1	1
**6.487**	0	0	1	0	0	0	1	0	0	1	0	0	1	1	0	0	0	0	0	0	0	1	1	0	0	1
**6.488**	0	0	1	0	0	0	1	0	0	1	0	0	0	1	0	0	0	0	0	0	0	0	0	0	1	1
**6.489**	0	0	1	0	0	0	1	0	0	0	0	0	1	1	0	1	0	0	0	0	0	1	0	0	0	1
**6.490**	0	0	1	0	0	0	1	0	0	0	0	0	1	1	0	0	0	0	0	0	0	0	0	1	0	1
**6.491**	0	0	1	0	0	0	1	0	0	0	0	0	1	0	0	1	0	0	0	1	0	1	0	0	0	1
**6.492**	0	0	1	0	0	0	1	0	0	0	0	0	1	0	0	0	0	0	0	0	0	1	1	0	0	1
**6.493**	0	0	1	0	0	0	0	0	0	1	0	0	0	1	0	1	0	0	0	1	0	0	0	1	0	1
**6.494**	0	0	1	0	0	0	0	0	0	1	0	0	0	0	0	0	0	0	0	0	0	1	1	1	1	1
**6.495**	0	0	1	0	0	0	0	0	0	0	1	1	0	1	0	0	0	0	0	1	0	1	0	1	1	1
**6.496**	0	0	1	0	0	0	0	0	0	0	1	0	1	0	0	1	0	0	0	0	0	0	0	0	0	1
**6.497**	0	0	1	0	0	0	0	0	0	0	0	0	1	1	0	1	0	0	0	1	0	1	0	0	0	1
**6.498**	0	0	0	1	0	0	1	0	1	1	0	0	0	1	0	0	0	0	0	0	0	1	0	1	1	1
**6.499**	0	0	0	1	0	0	1	0	1	0	0	0	0	1	0	0	0	0	0	0	0	1	0	1	1	1
**6.500**	0	0	0	1	0	0	0	0	1	0	0	0	0	1	0	0	0	0	0	1	0	1	0	0	0	1
**6.501**	0	0	0	1	0	0	0	0	0	1	1	0	0	1	0	0	0	0	0	0	0	1	0	0	0	1
**6.502**	0	0	0	1	0	0	0	0	0	0	0	0	1	1	0	1	0	0	0	1	0	1	0	1	0	1
**6.503**	0	0	0	1	0	0	0	0	0	0	0	0	1	1	0	1	0	0	0	0	0	1	0	1	0	1
**6.504**	0	0	0	0	1	0	1	0	1	1	0	1	0	1	0	0	0	0	0	1	0	1	0	1	0	1
**6.505**	0	0	0	0	1	0	1	0	0	1	0	0	0	0	0	0	0	0	0	0	0	0	1	0	0	1
**6.506**	0	0	0	0	1	0	0	0	0	1	0	1	1	0	0	1	0	0	0	0	0	1	0	0	0	1
**6.507**	0	0	0	0	1	0	0	0	0	1	0	1	0	1	0	0	0	0	0	0	0	1	1	1	0	1
**6.508**	0	0	0	0	1	0	0	0	0	0	0	1	1	0	0	0	0	0	0	1	0	0	0	1	0	1
**6.509**	0	0	0	0	0	0	1	0	1	0	0	0	0	1	0	1	0	0	0	0	0	1	0	0	0	1
**6.510**	0	0	0	0	0	0	1	0	0	1	0	0	1	1	0	1	0	0	0	0	0	0	0	0	0	1
**6.511**	0	0	0	0	0	0	1	0	0	1	0	0	1	0	0	0	0	0	0	0	0	0	0	1	0	1
**6.512**	0	0	0	0	0	0	1	0	0	1	0	0	0	0	0	1	0	0	0	0	0	0	0	1	0	1
**6.513**	0	0	0	0	0	0	1	0	0	0	1	0	1	1	0	0	0	0	0	1	0	1	0	0	0	1
**6.514**	0	0	0	0	0	0	1	0	0	0	0	0	1	1	0	1	0	0	0	0	0	1	0	0	0	1
**6.515**	0	0	0	0	0	0	0	0	1	0	0	1	0	1	0	1	0	0	0	1	0	1	0	1	0	1
**6.516**	0	0	0	0	0	0	0	0	1	0	0	1	0	0	0	1	0	0	0	1	0	1	0	0	0	1
**6.517**	0	0	0	0	0	0	0	0	0	1	0	1	1	1	0	0	0	0	0	0	0	1	1	0	0	1
**6.518**	0	0	0	0	0	0	0	0	0	1	0	1	1	1	0	0	0	0	0	0	0	0	1	0	0	1
**6.519**	0	0	0	0	0	0	0	0	0	1	0	1	0	0	0	1	0	0	0	0	0	1	0	1	1	1
**6.520**	0	0	0	0	0	0	0	0	0	0	1	1	0	1	0	1	0	0	0	0	0	1	0	0	0	1
**6.521**	0	0	0	0	0	0	0	0	0	0	0	1	1	1	0	0	0	0	0	0	0	1	0	1	0	1
**6.522**	0	1	1	1	1	0	0	0	1	0	0	1	0	0	0	1	0	0	0	1	0	1	0	0	1	1
**6.523**	0	1	1	1	1	0	0	0	0	0	0	1	0	0	0	0	0	0	0	0	0	0	1	0	0	1
**6.524**	0	1	1	1	0	0	1	0	0	1	0	1	0	1	0	0	0	0	0	0	0	0	0	0	0	1
**6.525**	0	1	1	1	0	0	0	0	1	1	0	0	0	0	0	0	0	0	0	0	0	0	0	1	0	1
**6.526**	0	1	1	0	1	0	0	0	1	0	0	1	1	0	0	0	0	0	0	0	0	0	0	1	1	1
**6.527**	0	1	1	0	1	0	0	0	0	1	0	1	1	0	0	0	0	0	0	0	0	0	0	0	1	1
**6.528**	0	1	1	0	1	0	0	0	0	1	0	1	0	1	0	1	0	0	0	0	0	0	0	1	1	1
**6.529**	0	1	1	0	1	0	0	0	0	1	0	1	0	1	0	0	0	0	0	0	0	0	0	1	0	1
**6.530**	0	1	1	0	1	0	0	0	0	1	0	1	0	0	0	0	0	0	0	0	0	0	0	1	0	1
**6.531**	0	1	1	0	1	0	0	0	0	1	0	0	0	0	0	0	0	0	0	0	0	0	1	1	1	1
**6.532**	0	1	1	0	1	0	0	0	0	0	0	0	1	0	0	0	0	0	0	0	0	0	1	0	1	1
**6.533**	0	1	1	0	1	0	0	0	0	0	0	0	1	0	0	0	0	0	0	0	0	0	0	0	1	1
**6.534**	0	1	1	0	1	0	0	0	0	0	0	0	0	0	0	0	0	0	0	1	0	0	1	1	0	1
**6.535**	0	1	1	0	0	0	1	0	0	1	0	0	0	0	0	0	0	0	0	1	0	0	1	0	0	1
**6.536**	0	1	1	0	0	0	1	0	0	0	0	0	0	0	0	0	0	0	0	1	0	0	1	0	0	1
**6.537**	0	1	1	0	0	0	0	0	1	1	0	1	1	0	0	0	0	0	0	0	0	0	0	0	0	1
**6.538**	0	1	1	0	0	0	0	0	0	1	0	1	1	1	0	1	0	0	0	0	0	0	0	0	0	1
**6.539**	0	1	1	0	0	0	0	0	0	1	0	1	1	1	0	0	0	0	0	0	0	0	0	1	0	1
**6.540**	0	1	1	0	0	0	0	0	0	1	0	1	1	1	0	0	0	0	0	0	0	0	0	0	0	1
**6.541**	0	1	1	0	0	0	0	0	0	1	0	1	0	1	0	1	0	0	0	0	0	0	0	1	1	1
**6.542**	0	1	1	0	0	0	0	0	0	0	0	1	0	1	0	0	0	0	0	1	0	0	1	0	1	1
**6.543**	0	1	1	0	0	0	0	0	0	0	0	1	0	1	0	0	0	0	0	1	0	0	0	0	0	1
**6.544**	0	1	1	0	0	0	0	0	0	0	0	1	0	0	0	0	0	0	0	0	0	1	0	0	0	1
**6.545**	0	1	1	0	0	0	0	0	0	0	0	0	1	0	0	0	0	0	0	0	0	0	1	0	1	1
**6.546**	0	1	0	1	1	0	0	0	1	0	0	0	1	0	0	0	0	0	0	0	0	0	0	1	0	1
**6.547**	0	1	0	1	1	0	0	0	1	0	0	0	0	0	0	0	0	0	0	1	0	1	0	0	0	1
**6.548**	0	1	0	1	1	0	0	0	0	1	0	1	0	1	0	0	0	0	0	1	0	0	1	0	0	1
**6.549**	0	1	0	1	1	0	0	0	0	0	0	0	0	1	0	0	0	0	0	0	0	0	0	0	0	1
**6.550**	0	1	0	1	1	0	0	0	0	0	0	0	0	0	0	0	0	0	0	0	0	1	0	0	1	1
**6.551**	0	1	0	1	0	0	0	0	1	1	0	1	1	1	0	0	0	0	0	0	0	0	0	1	0	1
**6.552**	0	1	0	1	0	0	0	0	1	1	0	1	1	1	0	0	0	0	0	0	0	0	0	0	0	1
**6.553**	0	1	0	1	0	0	0	0	0	1	0	1	1	1	0	0	0	0	0	1	0	0	0	1	1	1
**6.554**	0	1	0	1	0	0	0	0	0	1	0	1	1	1	0	0	0	0	0	0	0	0	0	1	0	1
**6.555**	0	1	0	1	0	0	0	0	0	1	0	1	1	0	0	0	0	0	0	0	0	1	0	0	0	1
**6.556**	0	1	0	1	0	0	0	0	0	0	0	1	0	1	0	0	0	0	0	1	0	0	1	0	1	1
**6.557**	0	1	0	1	0	0	0	0	0	0	0	0	0	0	0	0	0	0	0	0	0	1	1	1	0	1
**6.558**	0	1	0	0	1	0	1	0	0	1	0	1	1	0	0	0	0	0	0	0	0	0	0	1	1	1
**6.559**	0	1	0	0	1	0	0	0	0	1	0	1	0	1	0	0	0	0	0	1	0	0	1	1	1	1
**6.560**	0	1	0	0	1	0	0	0	0	1	0	1	0	1	0	0	0	0	0	1	0	0	0	1	0	1
**6.561**	0	1	0	0	1	0	0	0	0	0	0	0	1	0	0	0	0	0	0	0	0	1	1	1	0	1
**6.562**	0	1	0	0	1	0	0	0	0	0	0	0	0	1	0	0	0	0	0	0	0	1	0	1	1	1
**6.563**	0	1	0	0	0	0	1	0	1	0	0	1	0	0	0	0	0	0	0	0	0	0	0	0	1	1
**6.564**	0	1	0	0	0	0	1	0	0	0	0	1	1	1	0	0	0	0	0	0	0	0	0	0	0	1
**6.565**	0	1	0	0	0	0	1	0	0	0	0	0	0	1	0	0	0	0	0	0	0	0	1	0	0	1
**6.566**	0	1	0	0	0	0	0	0	1	0	0	1	1	0	0	0	0	0	0	1	0	0	0	1	0	1
**6.567**	0	1	0	0	0	0	0	0	1	0	0	1	0	0	0	1	0	0	0	1	0	1	0	0	1	1
**6.568**	0	1	0	0	0	0	0	0	0	1	0	0	0	0	0	0	0	0	0	0	0	1	1	1	0	1
**6.569**	0	1	0	0	0	0	0	0	0	0	0	1	1	0	0	0	0	0	0	1	0	0	0	1	0	1
**6.570**	0	1	0	0	0	0	0	0	0	0	0	1	0	0	0	1	0	0	0	1	0	1	0	0	1	1
**6.571**	0	1	0	0	0	0	0	0	0	0	0	1	0	0	0	1	0	0	0	1	0	1	0	0	0	1
**6.572**	0	0	1	1	0	0	1	0	1	1	0	1	0	1	0	0	0	0	0	1	0	1	1	1	0	1
**6.573**	0	0	1	1	0	0	1	0	1	0	0	1	1	1	0	0	0	0	0	1	0	1	0	0	1	1
**6.574**	0	0	1	1	0	0	1	0	0	1	0	1	1	1	0	0	0	0	0	1	0	1	1	1	1	1
**6.575**	0	0	1	1	0	0	0	0	0	0	1	1	0	1	0	0	0	0	0	1	0	1	0	1	0	1
**6.576**	0	0	1	1	0	0	0	0	0	0	0	0	1	1	0	0	0	0	0	0	0	1	0	0	0	1
**6.577**	0	0	1	1	0	0	0	0	0	0	0	0	1	0	0	0	0	0	0	0	0	1	0	1	0	1
**6.578**	0	0	1	1	0	0	0	0	0	0	0	0	1	0	0	0	0	0	0	0	0	1	0	0	0	1
**6.579**	0	0	1	1	0	0	0	0	0	0	0	0	0	1	0	0	0	0	0	1	0	1	0	1	0	1
**6.580**	0	0	1	1	0	0	0	0	0	0	0	0	0	0	0	0	0	0	0	0	0	1	0	1	0	1
**6.581**	0	0	1	0	1	0	1	0	1	0	0	0	0	0	0	0	0	0	0	1	0	1	0	0	0	1
**6.582**	0	0	1	0	1	0	1	0	0	1	0	1	0	1	0	0	0	0	0	0	0	1	0	1	1	1
**6.583**	0	0	1	0	1	0	0	0	0	1	0	0	0	0	0	0	0	0	0	0	0	0	1	1	1	1
**6.584**	0	0	1	0	1	0	0	0	0	0	0	1	0	0	0	0	0	0	0	1	0	1	0	0	1	1
**6.585**	0	0	1	0	1	0	0	0	0	0	0	1	0	0	0	0	0	0	0	1	0	1	0	0	0	1
**6.586**	0	0	1	0	1	0	0	0	0	0	0	1	0	0	0	0	0	0	0	0	0	1	0	0	1	1

CMP	CD2	CD3	CD4	CD7	CD8	CD10	CD13	CD18	CD26	CD29	CD36	CD44	CD45RA	CD49f	CD54	CD56	CD57	CD58	CD62L	CD71	CD80	Cytokeratin	Histone_H1	HLA-DQ	HLA-DR	Freq. (voxel)
**6.587**	0	0	1	0	1	0	0	0	0	0	0	0	0	0	0	0	0	0	0	0	0	0	0	0	1	1
**6.588**	0	0	1	0	0	0	1	0	1	1	0	1	1	1	0	0	0	0	0	1	0	1	1	1	1	1
**6.589**	0	0	1	0	0	0	1	0	1	1	0	1	0	1	0	0	0	0	0	0	0	0	0	1	0	1
**6.590**	0	0	1	0	0	0	1	0	1	0	0	1	0	0	0	0	0	0	0	0	0	1	0	0	1	1
**6.591**	0	0	1	0	0	0	1	0	0	1	0	1	1	1	0	0	0	0	0	1	0	1	1	1	1	1
**6.592**	0	0	1	0	0	0	1	0	0	1	0	0	0	1	0	0	0	0	0	1	0	1	0	1	0	1
**6.593**	0	0	1	0	0	0	1	0	0	0	1	0	0	0	0	1	0	0	0	0	0	0	0	0	0	1
**6.594**	0	0	1	0	0	0	1	0	0	0	0	1	0	1	0	0	0	0	0	0	0	1	0	1	0	1
**6.595**	0	0	1	0	0	0	1	0	0	0	0	1	0	1	0	0	0	0	0	0	0	0	1	1	1	1
**6.596**	0	0	1	0	0	0	1	0	0	0	0	1	0	0	0	0	0	0	0	0	0	0	1	1	0	1
**6.597**	0	0	1	0	0	0	1	0	0	0	0	0	0	1	0	0	0	0	0	1	0	1	1	0	0	1
**6.598**	0	0	1	0	0	0	0	0	1	1	0	1	0	1	0	0	0	0	0	0	0	1	1	0	0	1
**6.599**	0	0	1	0	0	0	0	0	0	1	1	1	1	0	0	0	0	0	0	0	0	1	0	0	1	1
**6.600**	0	0	1	0	0	0	0	0	0	1	1	1	1	0	0	0	0	0	0	0	0	0	0	0	1	1
**6.601**	0	0	1	0	0	0	0	0	0	1	1	0	0	1	0	0	0	0	0	1	0	1	0	0	0	1
**6.602**	0	0	1	0	0	0	0	0	0	1	1	0	0	0	0	0	0	0	0	0	0	1	0	0	0	1
**6.603**	0	0	1	0	0	0	0	0	0	1	0	1	1	0	0	0	0	0	0	1	0	0	0	0	1	1
**6.604**	0	0	1	0	0	0	0	0	0	1	0	1	0	0	0	1	0	0	0	1	0	0	0	0	0	1
**6.605**	0	0	1	0	0	0	0	0	0	1	0	0	0	1	0	0	0	0	0	0	0	0	0	0	1	1
**6.606**	0	0	1	0	0	0	0	0	0	0	1	1	0	1	0	0	0	0	0	1	0	1	0	0	0	1
**6.607**	0	0	1	0	0	0	0	0	0	0	0	0	1	1	0	0	0	0	0	0	0	1	1	0	0	1
**6.608**	0	0	1	0	0	0	0	0	0	0	0	0	1	0	0	0	0	0	0	0	0	0	1	1	1	1
**6.609**	0	0	0	1	1	0	1	0	0	1	0	1	0	1	0	0	0	0	0	1	0	0	0	0	0	1
**6.610**	0	0	0	1	1	0	1	0	0	1	0	1	0	1	0	0	0	0	0	0	0	1	0	0	0	1
**6.611**	0	0	0	1	1	0	0	0	1	0	0	0	0	0	0	0	0	0	0	0	0	0	0	0	1	1
**6.612**	0	0	0	1	0	0	1	0	1	0	0	0	1	1	0	0	0	0	0	0	0	1	0	0	0	1
**6.613**	0	0	0	1	0	0	1	0	0	1	0	1	0	1	0	0	0	0	0	0	0	1	0	1	1	1
**6.614**	0	0	0	1	0	0	1	0	0	0	0	0	0	1	0	0	0	0	0	0	0	0	0	0	0	1
**6.615**	0	0	0	0	1	0	0	0	0	1	1	1	1	0	0	0	0	0	0	0	0	0	0	0	0	1
**6.616**	0	0	0	0	1	0	0	0	0	0	1	1	1	0	0	0	0	0	0	0	0	0	0	0	0	1
**6.617**	0	0	0	0	0	0	1	0	1	1	0	1	1	1	0	0	0	0	0	0	0	0	0	0	1	1
**6.618**	0	0	0	0	0	0	1	0	1	1	0	1	0	1	0	0	0	0	0	1	0	1	1	1	0	1
**6.619**	0	0	0	0	0	0	1	0	1	1	0	0	0	0	0	0	0	0	0	0	0	0	1	1	1	1
**6.620**	0	0	0	0	0	0	1	0	1	0	0	1	1	1	0	0	0	0	0	1	0	1	0	0	1	1
**6.621**	0	0	0	0	0	0	1	0	0	1	1	1	0	1	0	0	0	0	0	1	0	1	1	0	1	1
**6.622**	0	0	0	0	0	0	1	0	0	1	1	1	0	1	0	0	0	0	0	0	0	1	1	0	1	1
**6.623**	0	0	0	0	0	0	1	0	0	1	1	0	0	0	0	0	0	0	0	0	0	0	1	1	1	1
**6.624**	0	0	0	0	0	0	1	0	0	1	0	1	1	1	0	0	0	0	0	1	0	1	0	1	0	1
**6.625**	0	0	0	0	0	0	1	0	0	1	0	1	0	0	0	1	0	0	0	1	0	0	0	1	1	1
**6.626**	0	0	0	0	0	0	1	0	0	0	1	1	0	0	0	0	0	0	0	1	0	1	0	0	0	1
**6.627**	0	0	0	0	0	0	1	0	0	0	1	0	0	1	0	1	0	0	0	1	0	1	0	0	0	1
**6.628**	0	0	0	0	0	0	1	0	0	0	1	0	0	0	0	1	0	0	0	0	0	0	0	0	0	1
**6.629**	0	0	0	0	0	0	1	0	0	0	0	1	1	1	0	0	0	0	0	0	0	0	1	0	1	1
**6.630**	0	0	0	0	0	0	1	0	0	0	0	1	0	1	0	1	0	0	0	1	0	0	0	1	1	1
**6.631**	0	0	0	0	0	0	0	0	1	1	0	0	0	1	0	0	0	0	0	1	0	1	0	0	1	1
**6.632**	0	0	0	0	0	0	0	0	0	1	0	1	1	1	0	0	0	0	0	1	0	0	1	0	0	1
**6.633**	0	0	0	0	0	0	0	0	0	1	0	1	0	0	0	1	0	0	0	1	0	0	0	0	0	1
**6.634**	0	0	0	0	0	0	0	0	0	0	1	0	1	1	0	0	0	0	0	1	0	1	0	0	0	1
**6.635**	0	1	1	1	1	0	1	0	0	0	0	1	0	0	0	0	0	0	0	0	0	0	0	0	0	1
**6.636**	0	1	1	1	1	0	0	0	0	1	0	1	1	0	0	0	0	0	0	0	0	0	0	1	0	1
**6.637**	0	1	1	1	0	0	1	0	0	0	0	1	0	0	0	0	0	0	0	1	0	0	0	0	0	1
**6.638**	0	1	1	1	0	0	0	0	0	1	0	1	0	0	0	0	0	0	0	0	0	0	0	1	0	1
**6.639**	0	1	1	0	1	0	0	0	0	1	0	1	0	0	0	0	0	0	0	0	0	0	1	1	0	1
**6.640**	0	1	1	0	1	0	0	0	0	0	0	1	0	1	0	0	0	0	0	0	0	0	0	0	1	1
**6.641**	0	1	1	0	0	0	1	0	0	1	0	1	0	1	0	0	0	0	0	0	0	1	0	1	1	1
**6.642**	0	1	1	0	0	0	1	0	0	0	0	0	0	0	0	0	0	0	0	0	0	0	0	0	0	1
**6.643**	0	1	1	0	0	0	0	0	0	0	0	1	0	1	0	0	0	0	0	0	0	0	0	1	1	1
**6.644**	0	1	0	1	1	0	0	0	1	0	0	1	0	1	0	0	0	0	0	0	0	1	0	0	0	1
**6.645**	0	1	0	1	1	0	0	0	1	0	0	1	0	1	0	0	0	0	0	0	0	0	0	0	0	1
**6.646**	0	1	0	1	1	0	0	0	1	0	0	1	0	0	0	1	0	0	0	1	0	1	0	0	1	1
**6.647**	0	1	0	1	0	0	0	0	1	1	0	0	0	0	0	0	0	0	0	0	0	0	0	1	0	1
**6.648**	0	1	0	1	0	0	0	0	1	0	0	1	0	0	0	1	0	0	0	1	0	1	0	0	0	1
**6.649**	0	1	0	1	0	0	0	0	0	1	0	1	0	0	0	0	0	0	0	1	0	0	1	1	1	1
**6.650**	0	1	0	1	0	0	0	0	0	1	0	0	0	1	0	0	0	0	0	0	0	0	0	1	0	1
**6.651**	0	1	0	1	0	0	0	0	0	0	0	0	0	1	0	0	0	0	0	0	0	0	1	1	0	1
**6.652**	0	1	0	1	0	0	0	0	0	0	0	0	0	1	0	0	0	0	0	0	0	0	0	1	0	1
**6.653**	0	1	0	0	1	0	1	0	0	1	1	1	0	1	0	0	0	0	0	1	0	1	0	0	0	1
**6.654**	0	1	0	0	1	0	1	0	0	0	0	0	0	0	0	0	0	0	0	1	0	0	0	0	0	1
**6.655**	0	1	0	0	1	0	0	0	1	1	0	0	1	0	0	0	0	0	0	0	0	0	0	1	0	1
**6.656**	0	1	0	0	1	0	0	0	1	0	0	1	1	0	0	0	0	0	0	1	0	0	1	0	1	1
**6.657**	0	1	0	0	1	0	0	0	1	0	0	0	1	0	0	0	0	0	0	1	0	0	1	1	1	1
**6.658**	0	1	0	0	1	0	0	0	0	1	0	0	0	0	0	0	0	0	0	1	0	0	1	1	1	1
**6.659**	0	1	0	0	1	0	0	0	0	0	0	1	0	1	0	0	0	0	0	1	0	1	0	1	0	1
**6.660**	0	1	0	0	1	0	0	0	0	0	0	1	0	1	0	0	0	0	0	0	0	0	0	0	1	1
**6.661**	0	1	0	0	1	0	0	0	0	0	0	1	0	0	0	1	0	0	0	1	0	1	0	0	1	1
**6.662**	0	1	0	0	0	0	1	0	0	1	0	1	0	0	0	0	0	0	0	1	0	0	1	1	1	1
**6.663**	0	1	0	0	0	0	1	0	0	0	1	1	0	0	0	0	0	0	0	0	0	1	0	1	1	1
**6.664**	0	1	0	0	0	0	1	0	0	0	0	0	0	0	0	0	0	0	0	0	0	0	0	0	0	1
**6.665**	0	1	0	0	0	0	0	0	1	1	0	1	0	1	0	0	0	0	0	1	0	0	0	0	1	1
**6.666**	0	1	0	0	0	0	0	0	1	0	0	1	1	0	0	0	0	0	0	0	0	0	1	1	1	1
**6.667**	0	1	0	0	0	0	0	0	1	0	0	0	1	1	0	0	0	0	0	0	0	0	0	1	1	1
**6.668**	0	1	0	0	0	0	0	0	0	1	0	1	1	1	0	1	0	0	0	0	0	0	0	1	0	1
**6.669**	0	1	0	0	0	0	0	0	0	0	1	1	0	1	0	0	0	0	0	0	0	1	0	1	0	1
**6.670**	0	1	0	0	0	0	0	0	0	0	1	1	0	1	0	0	0	0	0	0	0	0	0	1	1	1
**6.671**	0	1	0	0	0	0	0	0	0	0	1	0	0	1	0	0	0	0	0	0	0	1	0	1	1	1
**6.672**	0	1	0	0	0	0	0	0	0	0	0	0	0	1	0	0	0	0	0	1	0	0	1	1	1	1
**6.673**	0	1	0	0	0	0	0	0	0	0	0	0	0	1	0	0	0	0	0	1	0	0	1	1	0	1
**6.674**	0	0	1	1	0	0	1	0	1	1	0	1	1	1	0	0	0	0	0	1	0	1	1	1	1	1
**6.675**	0	0	1	1	0	0	1	0	1	1	0	1	0	1	0	0	0	0	0	0	0	0	0	0	1	1
**6.676**	0	0	1	1	0	0	1	0	0	0	0	0	1	0	0	1	0	0	0	0	0	0	0	1	0	1
**6.677**	0	0	1	1	0	0	0	0	0	1	0	1	0	1	0	0	0	0	0	1	0	1	1	0	1	1
**6.678**	0	0	1	1	0	0	0	0	0	0	0	1	1	1	0	1	0	0	0	1	0	1	0	1	1	1
**6.679**	0	0	1	1	0	0	0	0	0	0	0	1	0	1	0	0	0	0	0	0	0	1	0	0	0	1
**6.680**	0	0	1	0	1	0	0	0	1	0	0	1	0	0	0	0	0	0	0	0	0	1	0	0	0	1
**6.681**	0	0	1	0	1	0	0	0	0	1	0	1	0	1	0	0	0	0	0	0	0	1	0	0	0	1
**6.682**	0	0	1	0	1	0	0	0	0	1	0	0	0	0	0	0	0	0	0	0	0	0	1	0	0	1
**6.683**	0	0	1	0	1	0	0	0	0	0	0	0	0	0	0	0	0	0	0	0	0	1	0	0	1	1
**6.684**	0	0	1	0	0	0	1	0	1	1	0	1	0	1	0	0	0	0	0	0	0	0	0	0	1	1
**6.685**	0	0	1	0	0	0	1	0	1	0	0	0	1	1	0	0	0	0	0	1	0	0	0	1	1	1
**6.686**	0	0	1	0	0	0	1	0	1	0	0	0	0	0	0	0	0	0	0	0	0	1	1	0	0	1
**6.687**	0	0	1	0	0	0	1	0	0	0	1	1	0	1	0	0	0	0	0	1	0	1	0	1	0	1
**6.688**	0	0	1	0	0	0	0	0	1	1	0	1	0	0	0	0	0	0	0	0	0	0	1	1	0	1
**6.689**	0	0	1	0	0	0	0	0	1	1	0	0	0	0	0	0	0	0	0	0	0	0	0	0	1	1
**6.690**	0	0	1	0	0	0	0	0	1	0	0	1	0	0	0	1	0	0	0	0	0	0	0	1	1	1
**6.691**	0	0	1	0	0	0	0	0	1	0	0	1	0	0	0	0	0	0	0	0	0	1	1	1	0	1
**6.692**	0	0	1	0	0	0	0	0	1	0	0	0	1	1	0	0	0	0	0	0	0	0	0	1	0	1
**6.693**	0	0	1	0	0	0	0	0	0	0	1	0	0	1	0	0	0	0	0	1	0	1	0	1	0	1
**6.694**	0	0	0	1	1	0	0	0	0	0	0	0	1	0	0	0	0	0	0	0	0	0	0	1	0	1
**6.695**	0	0	0	1	0	0	1	0	1	1	1	1	1	1	0	0	0	0	0	1	0	0	0	1	1	1
**6.696**	0	0	0	1	0	0	1	0	1	1	0	1	0	1	0	0	0	0	0	1	0	1	1	0	0	1
**6.697**	0	0	0	1	0	0	1	0	0	1	0	1	1	1	0	0	0	0	0	1	0	1	0	1	1	1
**6.698**	0	0	0	1	0	0	1	0	0	0	0	0	0	1	0	0	0	0	0	0	0	1	1	0	0	1
**6.699**	0	0	0	1	0	0	0	0	1	1	0	1	0	1	0	1	0	0	0	0	0	1	0	0	0	1
**6.700**	0	0	0	1	0	0	0	0	0	1	0	1	0	1	0	1	0	0	0	1	0	1	0	0	0	1
**6.701**	0	0	0	1	0	0	0	0	0	1	0	1	0	1	0	0	0	0	0	1	0	1	1	0	0	1
**6.702**	0	0	0	0	1	0	0	0	0	1	0	1	0	1	0	0	0	0	0	1	0	1	1	1	1	1
**6.703**	0	0	0	0	1	0	0	0	0	1	0	1	0	0	0	1	0	0	0	0	0	0	0	0	0	1
**6.704**	0	0	0	0	0	0	1	0	1	1	1	1	1	1	0	0	0	0	0	0	0	0	0	1	1	1
**6.705**	0	0	0	0	0	0	1	0	1	1	0	1	0	1	0	0	0	0	0	0	0	0	0	1	0	1
**6.706**	0	0	0	0	0	0	1	0	1	0	0	1	1	0	0	0	0	0	0	0	0	0	0	0	0	1
**6.707**	0	0	0	0	0	0	1	0	1	0	0	1	0	0	0	1	0	0	0	0	0	0	0	0	1	1
**6.708**	0	0	0	0	0	0	1	0	0	1	1	1	0	1	0	1	0	0	0	1	0	0	0	1	1	1

CMP	CD2	CD3	CD4	CD7	CD8	CD10	CD13	CD18	CD26	CD29	CD36	CD44	CD45RA	CD49f	CD54	CD56	CD57	CD58	CD62L	CD71	CD80	Cytokeratin	Histone_H1	HLA-DQ	HLA-DR	Freq. (voxel)
**6.709**	0	0	0	0	0	0	1	0	0	1	0	1	0	1	0	1	0	0	0	0	0	0	0	1	0	1
**6.710**	0	0	0	0	0	0	1	0	0	0	0	1	0	0	0	0	0	0	0	1	0	0	1	0	1	1
**6.711**	0	0	0	0	0	0	1	0	0	0	0	0	0	1	0	1	0	0	0	1	0	1	0	0	1	1
**6.712**	0	0	0	0	0	0	0	0	1	1	0	1	1	1	0	0	0	0	0	1	0	0	0	0	0	1
**6.713**	0	0	0	0	0	0	0	0	1	1	0	1	0	1	0	1	0	0	0	1	0	1	0	0	0	1
**6.714**	0	0	0	0	0	0	0	0	1	1	0	0	0	1	0	0	0	0	0	0	0	0	1	1	0	1
**6.715**	0	0	0	0	0	0	0	0	0	1	0	0	0	0	0	1	0	0	0	0	0	1	0	1	1	1
**6.716**	0	0	0	0	0	0	0	0	0	0	0	0	0	1	0	1	0	0	0	0	0	0	0	1	1	1
**6.717**	0	1	1	1	1	0	0	0	0	0	0	0	1	0	0	0	0	0	0	0	0	0	0	0	0	1
**6.718**	0	1	1	1	0	0	0	0	1	1	0	1	0	1	0	0	0	0	0	0	0	0	0	1	0	1
**6.719**	0	1	1	0	1	0	0	0	1	0	0	0	1	0	0	0	0	0	0	1	0	0	1	1	0	1
**6.720**	0	1	1	0	1	0	0	0	1	0	0	0	1	0	0	0	0	0	0	0	0	0	0	1	0	1
**6.721**	0	1	1	0	0	0	0	0	1	0	0	1	0	0	0	1	0	0	0	0	0	0	0	1	0	1
**6.722**	0	1	1	0	0	0	0	0	0	1	0	1	0	1	0	0	0	0	0	0	0	1	0	0	0	1
**6.723**	0	1	1	0	0	0	0	0	0	0	0	0	0	1	0	0	0	0	0	0	0	0	1	0	0	1
**6.724**	0	1	0	1	1	0	0	0	1	0	0	0	1	0	0	0	0	0	0	0	0	1	0	0	0	1
**6.725**	0	1	0	1	1	0	0	0	0	1	0	1	0	0	0	0	0	0	0	0	0	0	1	1	1	1
**6.726**	0	1	0	1	1	0	0	0	0	0	0	0	0	1	0	0	0	0	0	0	0	1	1	0	0	1
**6.727**	0	1	0	1	0	0	0	0	1	1	0	1	0	0	0	0	0	0	0	1	0	1	0	0	0	1
**6.728**	0	1	0	1	0	0	0	0	1	1	0	0	0	0	0	0	0	0	0	0	0	0	1	1	0	1
**6.729**	0	1	0	1	0	0	0	0	1	0	0	0	1	0	0	0	0	0	0	0	0	0	1	0	0	1
**6.730**	0	1	0	1	0	0	0	0	1	0	0	0	0	0	0	0	0	0	0	1	0	1	0	0	0	1
**6.731**	0	1	0	1	0	0	0	0	0	0	0	0	0	1	0	0	0	0	0	0	0	1	1	0	0	1
**6.732**	0	1	0	0	1	0	1	0	0	0	0	1	0	0	0	0	0	0	0	0	0	1	1	0	0	1
**6.733**	0	1	0	0	1	0	0	0	1	1	0	0	1	0	0	0	0	0	0	1	0	0	1	1	1	1
**6.734**	0	1	0	0	1	0	0	0	1	1	0	0	0	0	0	0	0	0	0	0	0	0	0	0	1	1
**6.735**	0	1	0	0	1	0	0	0	1	0	0	0	1	0	0	0	0	0	0	0	0	0	0	0	0	1
**6.736**	0	1	0	0	1	0	0	0	1	0	0	0	0	0	0	0	0	0	0	1	0	0	1	1	1	1
**6.737**	0	1	0	0	1	0	0	0	0	1	1	1	1	0	0	0	0	0	0	0	0	0	1	1	1	1
**6.738**	0	1	0	0	1	0	0	0	0	0	1	1	0	0	0	0	0	0	0	0	0	1	0	1	1	1
**6.739**	0	1	0	0	1	0	0	0	0	0	0	1	1	1	0	0	0	0	0	0	0	0	0	1	0	1
**6.740**	0	1	0	0	1	0	0	0	0	0	0	1	0	1	0	0	0	0	0	0	0	0	0	1	0	1
**6.741**	0	1	0	0	0	0	1	0	0	1	0	1	0	1	0	0	0	0	0	1	0	1	0	0	1	1
**6.742**	0	1	0	0	0	0	1	0	0	1	0	1	0	0	0	0	0	0	0	0	0	0	1	0	1	1
**6.743**	0	1	0	0	0	0	1	0	0	1	0	0	0	1	0	0	0	0	0	0	0	0	1	0	0	1
**6.744**	0	1	0	0	0	0	0	0	1	1	0	1	1	0	0	1	0	0	0	0	0	0	0	0	0	1
**6.745**	0	1	0	0	0	0	0	0	1	1	0	1	0	1	0	1	0	0	0	0	0	0	0	0	0	1
**6.746**	0	1	0	0	0	0	0	0	1	1	0	0	0	0	0	0	0	0	0	0	0	0	1	1	0	1
**6.747**	0	1	0	0	0	0	0	0	1	0	0	1	1	0	0	0	0	0	0	0	0	0	1	1	0	1
**6.748**	0	1	0	0	0	0	0	0	1	0	0	1	1	0	0	0	0	0	0	0	0	0	0	1	0	1
**6.749**	0	1	0	0	0	0	0	0	0	1	0	1	1	0	0	1	0	0	0	0	0	0	0	0	0	1
**6.750**	0	1	0	0	0	0	0	0	0	1	0	1	0	1	0	1	0	0	0	0	0	0	0	0	0	1
**6.751**	0	1	0	0	0	0	0	0	0	0	1	1	0	0	0	0	0	0	0	1	0	0	0	0	0	1
**6.752**	0	1	0	0	0	0	0	0	0	0	1	0	0	1	0	0	0	0	0	0	0	0	0	1	0	1
**6.753**	0	0	1	1	0	0	1	0	1	1	1	1	0	1	0	1	0	0	0	0	0	0	0	1	1	1
**6.754**	0	0	1	1	0	0	1	0	1	1	0	1	0	1	0	0	0	0	0	1	0	0	0	0	1	1
**6.755**	0	0	1	1	0	0	1	0	1	1	0	1	0	1	0	0	0	0	0	0	0	1	0	0	1	1
**6.756**	0	0	1	1	0	0	1	0	0	1	0	1	1	1	0	0	0	0	0	1	0	0	1	1	1	1
**6.757**	0	0	1	1	0	0	1	0	0	1	0	1	0	1	0	0	0	0	0	0	0	1	0	0	1	1
**6.758**	0	0	1	1	0	0	1	0	0	1	0	1	0	1	0	0	0	0	0	0	0	0	0	1	0	1
**6.759**	0	0	1	1	0	0	0	0	1	0	0	1	0	1	0	0	0	0	0	0	0	1	0	0	0	1
**6.760**	0	0	1	1	0	0	0	0	0	1	0	1	0	1	0	1	0	0	0	1	0	1	0	1	1	1
**6.761**	0	0	1	0	1	0	1	0	1	0	0	0	1	0	0	0	0	0	0	0	0	0	0	1	0	1
**6.762**	0	0	1	0	1	0	1	0	1	0	0	0	1	0	0	0	0	0	0	0	0	0	0	0	0	1
**6.763**	0	0	1	0	1	0	1	0	0	1	0	1	0	1	0	0	0	0	0	0	0	1	0	0	0	1
**6.764**	0	0	1	0	1	0	0	0	1	0	0	0	1	0	0	0	0	0	0	0	0	0	0	1	0	1
**6.765**	0	0	1	0	1	0	0	0	1	0	0	0	0	0	0	0	0	0	0	0	0	0	0	1	0	1
**6.766**	0	0	1	0	1	0	0	0	0	0	0	1	1	0	0	0	0	0	0	0	0	1	0	0	1	1
**6.767**	0	0	1	0	0	0	1	0	1	1	0	1	0	1	0	1	0	0	0	1	0	1	0	0	0	1
**6.768**	0	0	1	0	0	0	1	0	1	1	0	1	0	1	0	1	0	0	0	0	0	1	0	0	1	1
**6.769**	0	0	1	0	0	0	1	0	1	1	0	1	0	1	0	0	0	0	0	1	0	1	1	1	0	1
**6.770**	0	0	1	0	0	0	1	0	1	1	0	1	0	1	0	0	0	0	0	1	0	1	1	0	1	1
**6.771**	0	0	1	0	0	0	1	0	1	1	0	1	0	1	0	0	0	0	0	1	0	1	1	0	0	1
**6.772**	0	0	1	0	0	0	1	0	1	1	0	0	0	1	0	0	0	0	0	1	0	0	0	0	0	1
**6.773**	0	0	1	0	0	0	1	0	1	0	0	1	0	1	0	0	0	0	0	0	0	1	1	1	0	1
**6.774**	0	0	1	0	0	0	1	0	1	0	0	0	1	0	0	1	0	0	0	0	0	0	0	0	0	1
**6.775**	0	0	1	0	0	0	1	0	1	0	0	0	0	1	0	0	0	0	0	0	0	0	0	1	0	1
**6.776**	0	0	1	0	0	0	1	0	1	0	0	0	0	0	0	1	0	0	0	0	0	1	0	0	0	1
**6.777**	0	0	1	0	0	0	1	0	0	1	0	1	0	1	0	0	0	0	0	0	0	0	1	0	1	1
**6.778**	0	0	1	0	0	0	1	0	0	1	0	0	0	1	0	0	0	0	0	1	0	1	1	0	1	1
**6.779**	0	0	1	0	0	0	1	0	0	1	0	0	0	1	0	0	0	0	0	0	0	0	1	0	1	1
**6.780**	0	0	1	0	0	0	1	0	0	0	1	1	0	1	0	0	0	0	0	0	0	1	0	1	0	1
**6.781**	0	0	1	0	0	0	1	0	0	0	1	0	0	1	0	0	0	0	0	0	0	1	0	1	0	1
**6.782**	0	0	1	0	0	0	1	0	0	0	0	1	1	1	0	0	0	0	0	1	0	1	0	0	1	1
**6.783**	0	0	1	0	0	0	1	0	0	0	0	1	0	0	0	0	0	0	0	0	0	0	1	0	0	1
**6.784**	0	0	1	0	0	0	1	0	0	0	0	0	1	1	0	0	0	0	0	0	0	0	0	0	0	1
**6.785**	0	0	1	0	0	0	1	0	0	0	0	0	0	0	0	0	0	0	0	1	0	0	1	0	0	1
**6.786**	0	0	1	0	0	0	0	0	1	0	0	0	1	0	0	1	0	0	0	0	0	1	0	0	0	1
**6.787**	0	0	1	0	0	0	0	0	0	1	1	0	0	1	0	0	0	0	0	0	0	1	0	0	0	1
**6.788**	0	0	1	0	0	0	0	0	0	1	0	1	1	1	0	0	0	0	0	1	0	1	1	0	0	1
**6.789**	0	0	1	0	0	0	0	0	0	1	0	1	0	1	0	1	0	0	0	0	0	1	0	0	1	1
**6.790**	0	0	1	0	0	0	0	0	0	1	0	0	0	1	0	0	0	0	0	1	0	0	1	1	1	1
**6.791**	0	0	1	0	0	0	0	0	0	0	1	1	0	1	0	0	0	0	0	1	0	0	0	1	1	1
**6.792**	0	0	1	0	0	0	0	0	0	0	1	1	0	1	0	0	0	0	0	0	0	1	0	0	1	1
**6.793**	0	0	1	0	0	0	0	0	0	0	0	1	1	1	0	0	0	0	0	1	0	1	0	1	0	1
**6.794**	0	0	1	0	0	0	0	0	0	0	0	0	0	1	0	0	0	0	0	1	0	0	0	0	1	1
**6.795**	0	0	0	1	1	0	0	0	1	1	0	1	1	0	0	0	0	0	0	0	0	1	0	0	0	1
**6.796**	0	0	0	1	0	0	1	0	0	1	1	1	1	1	0	0	0	0	0	0	0	0	0	1	1	1
**6.797**	0	0	0	1	0	0	1	0	0	1	0	1	0	1	0	0	0	0	0	1	0	1	1	1	1	1
**6.798**	0	0	0	1	0	0	1	0	0	1	0	0	1	1	0	0	0	0	0	1	0	1	0	0	0	1
**6.799**	0	0	0	1	0	0	0	0	1	1	1	1	0	0	0	0	0	0	0	0	0	0	0	1	1	1
**6.800**	0	0	0	1	0	0	0	0	1	1	0	1	0	1	0	0	0	0	0	1	0	1	1	0	1	1
**6.801**	0	0	0	1	0	0	0	0	1	1	0	1	0	1	0	0	0	0	0	0	0	1	0	0	1	1
**6.802**	0	0	0	1	0	0	0	0	1	0	1	0	0	1	0	0	0	0	0	0	0	1	0	0	0	1
**6.803**	0	0	0	1	0	0	0	0	1	0	0	0	1	0	0	0	0	0	0	0	0	0	0	1	0	1
**6.804**	0	0	0	1	0	0	0	0	0	1	0	0	1	0	0	1	0	0	0	0	0	0	0	0	0	1
**6.805**	0	0	0	1	0	0	0	0	0	1	0	0	0	1	0	0	0	0	0	0	0	0	0	1	1	1
**6.806**	0	0	0	0	1	0	1	0	1	1	0	1	0	0	0	0	0	0	0	0	0	0	0	0	0	1
**6.807**	0	0	0	0	1	0	1	0	1	0	0	0	0	0	0	0	0	0	0	0	0	0	0	1	0	1
**6.808**	0	0	0	0	1	0	1	0	0	0	0	0	1	0	0	0	0	0	0	0	0	1	0	0	0	1
**6.809**	0	0	0	0	1	0	0	0	1	0	0	1	0	1	0	1	0	0	0	0	0	0	0	1	1	1
**6.810**	0	0	0	0	0	0	1	0	1	1	0	0	0	1	0	0	0	0	0	0	0	1	0	1	0	1
**6.811**	0	0	0	0	0	0	1	0	1	0	0	1	0	1	0	1	0	0	0	0	0	0	0	1	1	1
**6.812**	0	0	0	0	0	0	1	0	1	0	0	1	0	1	0	0	0	0	0	1	0	0	0	0	0	1
**6.813**	0	0	0	0	0	0	1	0	1	0	0	1	0	1	0	0	0	0	0	0	0	0	1	0	1	1
**6.814**	0	0	0	0	0	0	1	0	1	0	0	0	1	0	0	1	0	0	0	0	0	0	1	0	0	1
**6.815**	0	0	0	0	0	0	1	0	1	0	0	0	1	0	0	1	0	0	0	0	0	0	0	1	0	1
**6.816**	0	0	0	0	0	0	1	0	0	1	0	1	1	0	0	0	0	0	0	0	0	0	1	0	0	1
**6.817**	0	0	0	0	0	0	1	0	0	1	0	1	0	1	0	1	0	0	0	0	0	1	0	0	0	1
**6.818**	0	0	0	0	0	0	1	0	0	1	0	0	1	1	0	1	0	0	0	0	0	1	0	0	0	1
**6.819**	0	0	0	0	0	0	1	0	0	0	1	0	0	1	0	1	0	0	0	0	0	1	0	1	0	1
**6.820**	0	0	0	0	0	0	1	0	0	0	1	0	0	0	0	0	0	0	0	0	0	1	0	1	0	1
**6.821**	0	0	0	0	0	0	1	0	0	0	0	1	0	1	0	0	0	0	0	0	0	1	1	0	1	1
**6.822**	0	0	0	0	0	0	1	0	0	0	0	0	0	1	0	0	0	0	0	1	0	0	1	0	1	1
**6.823**	0	0	0	0	0	0	1	0	0	0	0	0	0	0	0	0	0	0	0	0	0	1	1	1	0	1
**6.824**	0	0	0	0	0	0	0	0	1	1	1	0	0	1	0	0	0	0	0	1	0	1	0	0	0	1
**6.825**	0	0	0	0	0	0	0	0	1	0	0	1	0	1	0	0	0	0	0	0	0	0	0	0	1	1
**6.826**	0	0	0	0	0	0	0	0	1	0	0	1	0	0	0	1	0	0	0	0	0	0	0	0	1	1
**6.827**	0	0	0	0	0	0	0	0	1	0	0	0	1	1	0	0	0	0	0	0	0	0	1	1	0	1
**6.828**	0	0	0	0	0	0	0	0	1	0	0	0	0	0	0	1	0	0	0	0	0	0	0	0	1	1
**6.829**	0	0	0	0	0	0	0	0	0	1	0	1	0	1	0	1	0	0	0	0	0	1	0	1	1	1
**6.830**	0	0	0	0	0	0	0	0	0	1	0	1	0	1	0	1	0	0	0	0	0	0	0	1	1	1

CMP	CD2	CD3	CD4	CD7	CD8	CD10	CD13	CD18	CD26	CD29	CD36	CD44	CD45RA	CD49f	CD54	CD56	CD57	CD58	CD62L	CD71	CD80	Cytokeratin	Histone_H1	HLA-DQ	HLA-DR	Freq. (voxel)
**6.831**	0	0	0	0	0	0	0	0	0	1	0	0	1	1	0	0	0	0	0	0	0	1	0	1	1	1
**6.832**	0	0	0	0	0	0	0	0	0	1	0	0	1	1	0	0	0	0	0	0	0	0	0	1	1	1
**6.833**	0	0	0	0	0	0	0	0	0	1	0	0	1	0	0	1	0	0	0	0	0	0	1	1	0	1
**6.834**	0	0	0	0	0	0	0	0	0	0	0	1	1	1	0	0	0	0	0	1	0	1	0	0	1	1
**6.835**	0	0	0	0	0	0	0	0	0	0	0	1	0	1	0	1	0	0	0	0	0	0	0	1	1	1
**6.836**	0	0	0	0	0	0	0	0	0	0	0	1	0	0	0	1	0	0	0	1	0	0	0	1	0	1
**6.837**	0	0	0	0	0	0	0	0	0	0	0	0	0	1	0	1	0	0	0	0	0	1	0	0	1	1
**6.838**	0	1	1	1	1	0	0	0	0	0	0	1	1	0	0	0	0	0	0	0	0	0	0	0	0	1
**6.839**	0	1	1	1	0	0	0	0	1	1	0	1	1	0	0	0	0	0	0	1	0	0	0	1	0	1
**6.840**	0	1	1	0	1	0	0	0	1	1	0	0	1	0	0	0	0	0	0	1	0	0	1	1	0	1
**6.841**	0	1	1	0	1	0	0	0	1	0	0	1	1	0	0	0	0	0	0	0	0	1	0	0	0	1
**6.842**	0	1	1	0	0	0	0	0	1	1	0	1	0	0	0	0	0	0	0	1	0	0	0	1	0	1
**6.843**	0	1	1	0	0	0	0	0	0	0	0	1	0	0	0	0	0	0	0	0	0	1	0	1	0	1
**6.844**	0	1	0	1	1	0	1	0	0	0	0	1	0	0	0	0	0	0	0	0	0	1	0	0	0	1
**6.845**	0	1	0	1	1	0	0	0	1	0	0	1	0	1	0	0	0	0	0	1	0	0	0	0	1	1
**6.846**	0	1	0	1	1	0	0	0	1	0	0	0	0	0	0	0	0	0	0	1	0	1	0	0	1	1
**6.847**	0	1	0	1	1	0	0	0	1	0	0	0	0	0	0	0	0	0	0	1	0	0	1	1	0	1
**6.848**	0	1	0	1	0	0	0	0	0	1	1	1	0	1	0	0	0	0	0	0	0	1	0	0	0	1
**6.849**	0	1	0	0	1	0	0	0	1	1	1	1	0	0	0	0	0	0	0	0	0	0	1	0	1	1
**6.850**	0	1	0	0	1	0	0	0	1	1	0	0	1	0	0	0	0	0	0	1	0	0	1	1	0	1
**6.851**	0	1	0	0	1	0	0	0	1	1	0	0	0	0	0	0	0	0	0	1	0	0	1	1	0	1
**6.852**	0	1	0	0	1	0	0	0	1	0	0	0	0	0	0	0	0	0	0	1	0	0	1	1	0	1
**6.853**	0	1	0	0	1	0	0	0	0	1	1	1	0	1	0	0	0	0	0	0	0	1	1	1	1	1
**6.854**	0	1	0	0	1	0	0	0	0	0	0	1	0	0	0	1	0	0	0	0	0	0	0	1	0	1
**6.855**	0	1	0	0	1	0	0	0	0	0	0	1	0	0	0	1	0	0	0	0	0	0	0	0	1	1
**6.856**	0	1	0	0	0	0	0	0	1	1	0	0	0	0	0	0	0	0	0	1	0	0	1	1	0	1
**6.857**	0	1	0	0	0	0	0	0	0	1	0	1	0	1	0	0	0	0	0	1	0	1	0	0	1	1
**6.858**	0	1	0	0	0	0	0	0	0	0	1	0	0	0	0	0	0	0	0	1	0	0	0	0	0	1
**6.859**	0	1	0	0	0	0	0	0	0	0	1	0	0	0	0	0	0	0	0	0	0	1	0	1	0	1
**6.860**	0	1	0	0	0	0	0	0	0	0	1	0	0	0	0	0	0	0	0	0	0	0	0	1	0	1
**6.861**	0	1	0	0	0	0	0	0	0	0	0	1	0	0	0	1	0	0	0	0	0	0	0	0	1	1
**6.862**	0	0	1	1	0	0	0	0	1	1	0	1	0	1	0	0	0	0	0	1	0	1	0	1	1	1
**6.863**	0	0	1	1	0	0	0	0	0	0	0	0	1	0	0	1	0	0	0	0	0	0	1	1	0	1
**6.864**	0	0	1	0	1	0	1	0	1	0	0	0	1	0	0	0	0	0	0	1	0	0	0	1	0	1
**6.865**	0	0	1	0	1	0	1	0	0	1	0	1	0	0	0	0	0	0	0	0	0	1	0	0	0	1
**6.866**	0	0	1	0	0	0	1	0	1	1	0	1	1	1	0	1	0	0	0	1	0	1	0	0	1	1
**6.867**	0	0	1	0	0	0	1	0	0	1	0	1	0	1	0	1	0	0	0	1	0	1	0	0	1	1
**6.868**	0	0	1	0	0	0	1	0	0	1	0	1	0	1	0	1	0	0	0	1	0	1	0	0	0	1
**6.869**	0	0	1	0	0	0	1	0	0	1	0	1	0	1	0	1	0	0	0	1	0	0	1	0	1	1
**6.870**	0	0	1	0	0	0	1	0	0	1	0	1	0	1	0	1	0	0	0	1	0	0	0	0	1	1
**6.871**	0	0	1	0	0	0	1	0	0	1	0	1	0	1	0	0	0	0	0	1	0	0	1	0	1	1
**6.872**	0	0	1	0	0	0	1	0	0	0	0	0	0	1	0	0	0	0	0	1	0	1	0	0	1	1
**6.873**	0	0	1	0	0	0	0	0	1	1	0	0	1	1	0	0	0	0	0	0	0	0	0	1	1	1
**6.874**	0	0	1	0	0	0	0	0	1	0	1	1	0	1	0	0	0	0	0	1	0	1	0	0	0	1
**6.875**	0	0	1	0	0	0	0	0	1	0	1	0	0	1	0	0	0	0	0	1	0	1	1	0	0	1
**6.876**	0	0	1	0	0	0	0	0	0	1	1	1	0	1	0	0	0	0	0	1	0	1	0	0	0	1
**6.877**	0	0	1	0	0	0	0	0	0	0	1	1	0	0	0	0	0	0	0	1	0	0	0	1	1	1
**6.878**	0	0	1	0	0	0	0	0	0	0	1	0	0	1	0	0	0	0	0	1	0	1	1	0	0	1
**6.879**	0	0	1	0	0	0	0	0	0	0	0	1	1	1	0	0	0	0	0	0	0	1	0	0	1	1
**6.880**	0	0	1	0	0	0	0	0	0	0	0	1	0	1	0	0	0	0	0	1	0	1	0	1	0	1
**6.881**	0	0	0	1	1	0	0	0	0	1	1	1	0	1	0	0	0	0	0	0	0	1	0	0	0	1
**6.882**	0	0	0	1	1	0	0	0	0	0	0	1	1	0	0	0	0	0	0	0	0	1	1	0	1	1
**6.883**	0	0	0	1	1	0	0	0	0	0	0	0	0	0	0	0	0	0	0	0	0	1	1	0	1	1
**6.884**	0	0	0	1	0	0	1	0	1	1	0	1	0	1	0	0	0	0	0	0	0	0	0	0	0	1
**6.885**	0	0	0	1	0	0	1	0	1	1	0	0	0	1	0	0	0	0	0	0	0	0	0	0	0	1
**6.886**	0	0	0	1	0	0	1	0	1	0	0	0	0	1	0	0	0	0	0	0	0	1	0	0	1	1
**6.887**	0	0	0	1	0	0	0	0	0	0	0	0	1	0	0	0	0	0	0	0	0	0	1	0	0	1
**6.888**	0	0	0	0	1	0	1	0	0	1	0	1	1	0	0	0	0	0	0	0	0	0	0	0	0	1
**6.889**	0	0	0	0	1	0	0	0	1	0	0	0	1	0	0	1	0	0	0	0	0	1	0	0	0	1
**6.890**	0	0	0	0	1	0	0	0	0	1	1	1	0	0	0	0	0	0	0	0	0	0	1	1	1	1
**6.891**	0	0	0	0	1	0	0	0	0	0	0	0	1	0	0	1	0	0	0	0	0	1	0	0	0	1
**6.892**	0	0	0	0	0	0	1	0	1	0	1	0	1	1	0	0	0	0	0	0	0	1	0	0	0	1
**6.893**	0	0	0	0	0	0	1	0	0	1	0	0	0	1	0	0	0	0	0	1	0	0	0	1	0	1
**6.894**	0	0	0	0	0	0	0	0	1	1	0	1	1	1	0	0	0	0	0	1	0	1	0	1	1	1
**6.895**	0	0	0	0	0	0	0	0	1	1	0	0	0	1	0	0	0	0	0	0	0	1	0	1	0	1
**6.896**	0	0	0	0	0	0	0	0	1	1	0	0	0	0	0	0	0	0	0	0	0	0	0	0	1	1
**6.897**	0	0	0	0	0	0	0	0	1	0	1	0	0	0	0	0	0	0	0	0	0	0	0	1	0	1
**6.898**	0	0	0	0	0	0	0	0	0	1	1	0	0	1	0	0	0	0	0	0	0	1	0	1	1	1
**6.899**	0	0	0	0	0	0	0	0	0	1	0	1	0	1	0	1	0	0	0	1	0	1	0	1	0	1
**6.900**	0	0	0	0	0	0	0	0	0	0	1	1	0	0	0	1	0	0	0	1	0	0	0	1	1	1
**6.901**	0	0	0	0	0	0	0	0	0	0	1	0	0	1	0	1	0	0	0	0	0	1	0	1	0	1
**6.902**	0	0	0	0	0	0	0	0	0	0	0	1	1	1	0	1	0	0	0	0	0	1	0	0	1	1
**6.903**	0	0	0	0	0	0	0	0	0	0	0	1	1	1	0	1	0	0	0	0	0	1	0	0	0	1
**6.904**	0	0	0	0	0	0	0	0	0	0	0	1	0	1	0	1	0	0	0	0	0	1	0	1	0	1
**6.905**	0	0	0	0	0	0	0	0	0	0	0	0	1	1	0	1	0	0	0	0	0	1	0	0	1	1
**6.906**	0	1	1	1	1	0	0	0	1	0	0	1	0	0	0	0	0	0	0	1	0	0	0	0	1	1
**6.907**	0	1	1	1	1	0	0	0	1	0	0	0	0	0	0	0	0	0	0	1	0	0	1	1	0	1
**6.908**	0	1	1	1	0	0	0	0	1	1	0	0	1	0	0	0	0	0	0	0	0	0	0	1	0	1
**6.909**	0	1	1	1	0	0	0	0	0	1	0	0	1	0	0	0	0	0	0	0	0	0	0	1	0	1
**6.910**	0	1	1	1	0	0	0	0	0	0	0	1	1	0	0	0	0	0	0	0	0	0	0	0	0	1
**6.911**	0	1	1	1	0	0	0	0	0	0	0	0	1	0	0	0	0	0	0	0	0	0	0	0	0	1
**6.912**	0	1	1	1	0	0	0	0	0	0	0	0	0	0	0	0	0	0	0	0	0	0	0	1	0	1
**6.913**	0	1	1	0	1	0	0	0	1	1	0	0	0	0	0	0	0	0	0	1	0	0	0	0	0	1
**6.914**	0	1	1	0	1	0	0	0	1	0	0	0	0	0	0	0	0	0	0	1	0	0	1	1	0	1
**6.915**	0	1	0	1	1	0	0	0	1	1	0	0	1	0	0	0	0	0	0	0	0	0	1	0	0	1
**6.916**	0	1	0	1	1	0	0	0	0	0	0	1	0	1	0	0	0	0	0	0	0	0	1	0	1	1
**6.917**	0	1	0	1	0	0	0	0	1	1	0	0	1	0	0	0	0	0	0	0	0	0	0	1	0	1
**6.918**	0	1	0	1	0	0	0	0	0	1	0	0	0	0	0	0	0	0	0	0	0	0	0	1	0	1
**6.919**	0	1	0	0	1	0	1	0	0	0	0	0	0	0	0	0	0	0	0	0	0	0	0	0	1	1
**6.920**	0	1	0	0	0	0	1	0	0	0	0	0	0	0	0	0	0	0	0	1	0	0	0	0	0	1
**6.921**	0	1	0	0	0	0	0	0	1	1	0	1	0	0	0	1	0	0	0	0	0	0	0	1	0	1
**6.922**	0	1	0	0	0	0	0	0	1	1	0	1	0	0	0	1	0	0	0	0	0	0	0	0	0	1
**6.923**	0	1	0	0	0	0	0	0	1	1	0	1	0	0	0	0	0	0	0	0	0	0	1	0	1	1
**6.924**	0	1	0	0	0	0	0	0	1	1	0	0	0	0	0	0	0	0	0	1	0	0	1	0	0	1
**6.925**	0	1	0	0	0	0	0	0	0	1	1	1	0	1	0	0	0	0	0	0	0	0	1	1	1	1
**6.926**	0	1	0	0	0	0	0	0	0	1	1	1	0	1	0	0	0	0	0	0	0	0	0	1	0	1
**6.927**	0	1	0	0	0	0	0	0	0	0	1	1	0	0	0	0	0	0	0	0	0	1	0	0	1	1
**6.928**	0	1	0	0	0	0	0	0	0	0	0	1	0	1	0	1	0	0	0	0	0	0	0	0	0	1
**6.929**	0	0	1	1	0	0	1	0	1	1	0	1	1	1	0	1	0	0	0	1	0	0	1	0	1	1
**6.930**	0	0	1	1	0	0	1	0	0	1	1	1	0	1	0	1	0	0	0	0	0	0	0	1	1	1
**6.931**	0	0	1	1	0	0	0	0	0	0	1	0	0	1	0	0	0	0	0	1	0	1	0	0	0	1
**6.932**	0	0	1	1	0	0	0	0	0	0	0	1	0	0	0	0	0	0	0	0	0	1	0	0	1	1
**6.933**	0	0	1	1	0	0	0	0	0	0	0	0	0	0	0	0	0	0	0	0	0	0	1	1	0	1
**6.934**	0	0	1	0	0	0	1	0	1	1	0	1	0	1	0	1	0	0	0	1	0	0	0	0	1	1
**6.935**	0	0	1	0	0	0	1	0	0	0	1	0	0	0	0	0	0	0	0	0	0	1	0	0	0	1
**6.936**	0	0	1	0	0	0	1	0	0	0	0	0	1	1	0	0	0	0	0	1	0	0	0	1	1	1
**6.937**	0	0	1	0	0	0	1	0	0	0	0	0	0	0	0	0	0	0	0	1	0	0	0	0	1	1
**6.938**	0	0	1	0	0	0	0	0	1	1	0	1	0	1	0	0	0	0	0	0	0	0	0	0	1	1
**6.939**	0	0	1	0	0	0	0	0	1	0	0	1	1	1	0	0	0	0	0	1	0	1	0	0	0	1
**6.940**	0	0	1	0	0	0	0	0	1	0	0	1	0	0	0	1	0	0	0	0	0	1	0	0	0	1
**6.941**	0	0	1	0	0	0	0	0	0	1	1	1	0	0	0	0	0	0	0	0	0	0	0	1	0	1
**6.942**	0	0	1	0	0	0	0	0	0	1	0	1	1	1	0	1	0	0	0	0	0	1	0	1	1	1
**6.943**	0	0	1	0	0	0	0	0	0	1	0	1	1	1	0	0	0	0	0	0	0	1	0	0	1	1
**6.944**	0	0	1	0	0	0	0	0	0	0	1	1	0	0	0	0	0	0	0	0	0	0	0	1	0	1
**6.945**	0	0	1	0	0	0	0	0	0	0	0	1	1	1	0	1	0	0	0	0	0	1	0	0	0	1
**6.946**	0	0	1	0	0	0	0	0	0	0	0	1	0	1	0	1	0	0	0	0	0	1	0	0	0	1
**6.947**	0	0	0	1	0	0	1	0	1	1	1	1	0	1	0	1	0	0	0	1	0	0	0	1	1	1
**6.948**	0	0	0	1	0	0	1	0	0	1	0	0	0	1	0	0	0	0	0	1	0	0	0	0	0	1
**6.949**	0	0	0	1	0	0	0	0	1	1	0	1	0	1	0	0	0	0	0	0	0	0	0	1	1	1
**6.950**	0	0	0	1	0	0	0	0	0	1	0	1	0	1	0	0	0	0	0	0	0	1	0	1	1	1
**6.951**	0	0	0	1	0	0	0	0	0	1	0	1	0	1	0	0	0	0	0	0	0	1	0	1	0	1
**6.952**	0	0	0	0	1	0	1	0	0	1	0	1	1	1	0	0	0	0	0	1	0	1	0	0	1	1

CMP	CD2	CD3	CD4	CD7	CD8	CD10	CD13	CD18	CD26	CD29	CD36	CD44	CD45RA	CD49f	CD54	CD56	CD57	CD58	CD62L	CD71	CD80	Cytokeratin	Histone_H1	HLA-DQ	HLA-DR	Freq. (voxel)
**6.953**	0	0	0	0	1	0	1	0	0	0	0	0	0	0	0	0	0	0	0	1	0	0	0	0	0	1
**6.954**	0	0	0	0	1	0	0	0	0	1	0	1	1	1	0	0	0	0	0	0	0	0	1	0	1	1
**6.955**	0	0	0	0	0	0	1	0	1	1	1	0	0	1	0	0	0	0	0	0	0	1	0	0	1	1
**6.956**	0	0	0	0	0	0	1	0	1	1	0	1	1	1	0	0	0	0	0	1	0	0	1	0	1	1
**6.957**	0	0	0	0	0	0	1	0	1	1	0	1	1	1	0	0	0	0	0	1	0	0	1	0	0	1
**6.958**	0	0	0	0	0	0	1	0	1	1	0	1	0	1	0	1	0	0	0	1	0	1	0	0	0	1
**6.959**	0	0	0	0	0	0	1	0	1	1	0	1	0	1	0	0	0	0	0	0	0	0	1	0	0	1
**6.960**	0	0	0	0	0	0	1	0	1	0	1	0	0	1	0	0	0	0	0	0	0	1	0	0	1	1
**6.961**	0	0	0	0	0	0	1	0	1	0	0	0	0	0	0	0	0	0	0	1	0	0	1	0	1	1
**6.962**	0	0	0	0	0	0	1	0	0	1	1	1	0	1	0	0	0	0	0	0	0	1	0	0	1	1
**6.963**	0	0	0	0	0	0	1	0	0	1	1	1	0	0	0	0	0	0	0	0	0	0	0	1	0	1
**6.964**	0	0	0	0	0	0	1	0	0	1	0	0	0	1	0	0	0	0	0	1	0	1	0	1	0	1
**6.965**	0	0	0	0	0	0	1	0	0	0	1	0	0	0	0	0	0	0	0	0	0	1	1	0	0	1
**6.966**	0	0	0	0	0	0	0	0	1	1	0	1	1	1	0	0	0	0	0	0	0	0	1	0	1	1
**6.967**	0	0	0	0	0	0	0	0	1	1	0	1	1	1	0	0	0	0	0	0	0	0	0	0	1	1
**6.968**	0	0	0	0	0	0	0	0	1	0	1	0	0	1	0	0	0	0	0	0	0	1	1	0	0	1
**6.969**	0	0	0	0	0	0	0	0	0	1	0	1	0	1	0	1	0	0	0	0	0	0	1	1	1	1
**6.970**	0	0	0	0	0	0	0	0	0	0	1	0	1	0	0	0	0	0	0	0	0	0	1	0	0	1
**6.971**	0	0	0	0	0	0	0	0	0	0	0	1	0	1	0	1	0	0	0	0	0	0	0	1	0	1
**6.972**	0	1	1	0	1	0	0	0	1	1	0	0	0	0	0	0	0	0	0	1	0	0	1	0	0	1
**6.973**	0	1	1	0	0	0	0	0	0	1	0	1	0	1	0	0	0	0	0	0	0	1	0	0	1	1
**6.974**	0	1	0	1	1	0	0	0	1	1	0	0	0	0	0	0	0	0	0	0	0	0	1	0	0	1
**6.975**	0	1	0	1	1	0	0	0	1	0	0	1	1	0	0	0	0	0	0	1	0	0	1	0	1	1
**6.976**	0	1	0	0	1	0	1	0	1	1	0	1	0	0	0	0	0	0	0	0	0	0	1	0	0	1
**6.977**	0	1	0	0	1	0	0	0	0	1	1	1	1	0	0	0	0	0	0	1	0	0	1	0	1	1
**6.978**	0	1	0	0	1	0	0	0	0	1	1	1	0	0	0	0	0	0	0	1	0	0	1	0	1	1
**6.979**	0	1	0	0	1	0	0	0	0	1	1	1	0	0	0	0	0	0	0	1	0	0	1	0	0	1
**6.980**	0	1	0	0	1	0	0	0	0	1	0	1	1	1	0	0	0	0	0	1	0	0	0	0	1	1
**6.981**	0	1	0	0	1	0	0	0	0	0	0	1	0	1	0	0	0	0	0	1	0	0	1	0	1	1
**6.982**	0	1	0	0	1	0	0	0	0	0	0	0	1	0	0	0	0	0	0	1	0	1	1	0	0	1
**6.983**	0	1	0	0	0	0	1	0	0	1	0	1	0	1	0	0	0	0	0	0	0	0	0	0	1	1
**6.984**	0	1	0	0	0	0	0	0	1	0	0	0	0	0	0	0	0	0	0	0	0	1	0	1	1	1
**6.985**	0	1	0	0	0	0	0	0	0	1	1	1	0	0	0	0	0	0	0	0	0	0	1	1	0	1
**6.986**	0	1	0	0	0	0	0	0	0	1	1	1	0	0	0	0	0	0	0	0	0	0	1	0	0	1
**6.987**	0	1	0	0	0	0	0	0	0	1	0	1	0	0	0	1	0	0	0	0	0	0	0	1	0	1
**6.988**	0	1	0	0	0	0	0	0	0	1	0	0	0	0	0	0	0	0	0	1	0	0	0	0	1	1
**6.989**	0	1	0	0	0	0	0	0	0	1	0	0	0	0	0	0	0	0	0	0	0	0	0	1	0	1
**6.990**	0	0	1	1	0	0	1	0	1	1	1	1	0	1	0	1	0	0	0	1	0	0	0	1	1	1
**6.991**	0	0	1	1	0	0	1	0	0	1	0	1	0	1	0	0	0	0	0	1	0	1	0	0	0	1
**6.992**	0	0	1	0	0	0	1	0	0	1	1	1	0	1	0	0	0	0	0	0	0	0	0	0	0	1
**6.993**	0	0	1	0	0	0	1	0	0	1	0	1	1	1	0	1	0	0	0	1	0	0	0	0	0	1
**6.994**	0	0	1	0	0	0	1	0	0	1	0	0	0	1	0	1	0	0	0	1	0	0	0	0	0	1
**6.995**	0	0	1	0	0	0	1	0	0	0	1	0	0	0	0	0	0	0	0	1	0	0	0	1	1	1
**6.996**	0	0	1	0	0	0	0	0	1	1	1	1	0	1	0	0	0	0	0	0	0	0	0	1	1	1
**6.997**	0	0	1	0	0	0	0	0	1	0	0	1	0	0	0	1	0	0	0	1	0	0	0	1	1	1
**6.998**	0	0	1	0	0	0	0	0	1	0	0	0	1	0	0	1	0	0	0	0	0	0	0	0	0	1
**6.999**	0	0	1	0	0	0	0	0	1	0	0	0	1	0	0	0	0	0	0	1	0	0	0	0	0	1
**7.000**	0	0	1	0	0	0	0	0	0	1	0	1	0	1	0	0	0	0	0	0	0	0	1	1	1	1
**7.001**	0	0	0	1	1	0	0	0	0	0	0	0	0	0	0	0	0	0	0	1	0	1	0	0	0	1
**7.002**	0	0	0	1	0	0	1	0	1	1	1	0	0	1	0	0	0	0	0	0	0	0	0	1	1	1
**7.003**	0	0	0	1	0	0	1	0	1	1	0	1	0	1	0	0	0	0	0	1	0	1	0	0	1	1
**7.004**	0	0	0	1	0	0	1	0	1	1	0	0	0	1	0	0	0	0	0	1	0	0	0	1	1	1
**7.005**	0	0	0	1	0	0	1	0	1	0	0	0	0	1	0	0	0	0	0	1	0	0	0	0	0	1
**7.006**	0	0	0	1	0	0	1	0	0	1	1	0	0	1	0	0	0	0	0	0	0	0	0	1	1	1
**7.007**	0	0	0	1	0	0	1	0	0	1	0	1	0	1	0	0	0	0	0	1	0	0	1	0	0	1
**7.008**	0	0	0	1	0	0	1	0	0	0	0	0	1	1	0	0	0	0	0	0	0	1	0	0	0	1
**7.009**	0	0	0	1	0	0	0	0	1	1	1	0	0	1	0	0	0	0	0	0	0	0	0	1	1	1
**7.010**	0	0	0	1	0	0	0	0	1	0	1	0	0	0	0	0	0	0	0	1	0	1	0	0	0	1
**7.011**	0	0	0	1	0	0	0	0	0	1	1	1	0	1	0	0	0	0	0	0	0	0	0	1	0	1
**7.012**	0	0	0	1	0	0	0	0	0	1	0	0	0	1	0	0	0	0	0	0	0	0	0	0	1	1
**7.013**	0	0	0	1	0	0	0	0	0	0	0	0	1	1	0	0	0	0	0	0	0	0	0	0	0	1
**7.014**	0	0	0	0	0	0	1	0	1	0	0	1	1	0	0	0	0	0	0	0	0	1	0	0	1	1
**7.015**	0	0	0	0	0	0	1	0	1	0	0	0	1	0	0	0	0	0	0	1	0	0	0	0	1	1
**7.016**	0	0	0	0	0	0	1	0	0	1	0	1	1	1	0	1	0	0	0	1	0	0	0	0	0	1
**7.017**	0	0	0	0	0	0	1	0	0	1	0	1	1	1	0	0	0	0	0	1	0	0	0	0	1	1
**7.018**	0	0	0	0	0	0	1	0	0	1	0	0	0	1	0	0	0	0	0	1	0	0	1	1	1	1
**7.019**	0	0	0	0	0	0	1	0	0	1	0	0	0	1	0	0	0	0	0	0	0	1	0	1	1	1
**7.020**	0	0	0	0	0	0	1	0	0	0	1	0	0	0	0	0	0	0	0	0	0	0	1	0	0	1
**7.021**	0	0	0	0	0	0	0	0	1	1	0	1	0	1	0	0	0	0	0	1	0	0	1	1	0	1
**7.022**	0	0	0	0	0	0	0	0	1	1	0	1	0	1	0	0	0	0	0	1	0	0	1	0	0	1
**7.023**	0	0	0	0	0	0	0	0	1	1	0	0	0	0	0	0	0	0	0	0	0	0	1	1	1	1
**7.024**	0	0	0	0	0	0	0	0	1	0	1	1	0	1	0	0	0	0	0	0	0	1	0	0	1	1
**7.025**	0	0	0	0	0	0	0	0	1	0	0	0	1	0	0	0	0	0	0	1	0	0	0	0	1	1
**7.026**	0	0	0	0	0	0	0	0	0	1	0	0	1	1	0	0	0	0	0	1	0	1	0	0	1	1
**7.027**	0	0	0	0	0	0	0	0	0	1	0	0	1	1	0	0	0	0	0	1	0	1	0	0	0	1
**7.028**	0	0	0	0	0	0	0	0	0	1	0	0	1	1	0	0	0	0	0	0	0	1	0	0	1	1
**7.029**	0	0	0	0	0	0	0	0	0	1	0	0	0	1	0	1	0	0	0	0	0	1	1	0	0	1
**7.030**	0	1	0	1	1	0	1	0	1	0	0	1	0	0	0	0	0	0	0	1	0	0	1	0	0	1
**7.031**	0	1	0	1	1	0	1	0	0	1	0	1	0	1	0	0	0	0	0	0	0	1	0	0	1	1
**7.032**	0	1	0	1	1	0	0	0	0	1	0	1	0	0	0	0	0	0	0	0	0	0	1	1	0	1
**7.033**	0	1	0	0	1	0	1	0	0	1	1	1	0	1	0	0	0	0	0	0	0	0	1	0	0	1
**7.034**	0	1	0	0	1	0	1	0	0	0	0	0	0	0	0	0	0	0	0	0	0	0	0	0	0	1
**7.035**	0	1	0	0	0	0	0	0	1	0	0	0	1	0	0	0	0	0	0	1	0	0	1	0	1	1
**7.036**	0	1	0	0	0	0	0	0	1	0	0	0	0	0	0	0	0	0	0	1	0	0	0	0	1	1
**7.037**	0	0	1	1	0	0	1	0	0	1	0	0	1	1	0	1	0	0	0	1	0	0	0	1	1	1
**7.038**	0	0	1	1	0	0	1	0	0	1	0	0	0	1	0	1	0	0	0	1	0	0	0	1	1	1
**7.039**	0	0	1	1	0	0	0	0	0	1	0	1	0	0	0	0	0	0	0	1	0	0	0	0	0	1
**7.040**	0	0	1	0	0	0	1	0	1	1	1	0	0	1	0	0	0	0	0	0	0	0	0	1	1	1
**7.041**	0	0	1	0	0	0	1	0	0	1	1	1	1	1	0	1	0	0	0	0	0	0	0	1	1	1
**7.042**	0	0	1	0	0	0	1	0	0	1	1	1	0	1	0	1	0	0	0	0	0	0	0	1	0	1
**7.043**	0	0	1	0	0	0	1	0	0	0	0	0	0	0	0	0	0	0	0	1	0	1	0	0	1	1
**7.044**	0	0	1	0	0	0	0	0	1	1	1	1	0	1	0	0	0	0	0	1	0	0	0	1	1	1
**7.045**	0	0	1	0	0	0	0	0	0	1	0	1	1	0	0	0	0	0	0	0	0	1	0	0	1	1
**7.046**	0	0	1	0	0	0	0	0	0	0	1	0	0	0	0	0	0	0	0	0	0	0	0	0	1	1
**7.047**	0	0	1	0	0	0	0	0	0	0	0	1	0	0	0	1	0	0	0	1	0	0	1	1	1	1
**7.048**	0	0	0	1	1	0	1	0	0	1	0	1	0	1	0	0	0	0	0	0	0	1	0	0	1	1
**7.049**	0	0	0	1	0	0	1	0	0	0	0	1	0	1	0	0	0	0	0	0	0	0	0	0	0	1
**7.050**	0	0	0	1	0	0	0	0	1	1	0	1	0	0	0	1	0	0	0	0	0	1	0	0	1	1
**7.051**	0	0	0	0	1	0	1	0	0	1	0	1	1	1	0	0	0	0	0	0	0	1	1	0	1	1
**7.052**	0	0	0	0	1	0	1	0	0	1	0	0	0	1	0	0	0	0	0	0	0	1	1	0	1	1
**7.053**	0	0	0	0	1	0	0	0	0	1	1	1	0	1	0	0	0	0	0	0	0	0	1	1	1	1
**7.054**	0	0	0	0	0	0	1	0	1	1	0	0	0	1	0	1	0	0	0	0	0	0	0	1	1	1
**7.055**	0	0	0	0	0	0	1	0	0	1	1	0	0	1	0	0	0	0	0	0	0	0	0	1	1	1
**7.056**	0	0	0	0	0	0	1	0	0	1	0	1	1	1	0	0	0	0	0	0	0	1	0	1	1	1
**7.057**	0	0	0	0	0	0	1	0	0	1	0	1	0	0	0	1	0	0	0	1	0	0	0	0	1	1
**7.058**	0	0	0	0	0	0	1	0	0	1	0	0	0	1	0	0	0	0	0	1	0	0	1	1	0	1
**7.059**	0	0	0	0	0	0	1	0	0	0	0	1	0	1	0	1	0	0	0	0	0	0	0	0	1	1
**7.060**	0	0	0	0	0	0	1	0	0	0	0	0	1	0	0	0	0	0	0	1	0	0	0	0	1	1
**7.061**	0	0	0	0	0	0	0	0	1	1	0	0	0	0	0	1	0	0	0	0	0	1	0	0	0	1
**7.062**	0	0	0	0	0	0	0	0	1	0	1	1	0	0	0	0	0	0	0	0	0	0	0	0	0	1
**7.063**	0	0	0	0	0	0	0	0	1	0	1	0	0	0	0	0	0	0	0	1	0	1	1	0	1	1
**7.064**	0	0	0	0	0	0	0	0	1	0	1	0	0	0	0	0	0	0	0	1	0	1	1	0	0	1
**7.065**	0	0	0	0	0	0	0	0	1	0	0	1	0	1	0	0	0	0	0	0	0	0	1	0	1	1
**7.066**	0	0	0	0	0	0	0	0	1	0	0	0	1	1	0	0	0	0	0	0	0	0	0	0	0	1
**7.067**	0	0	0	0	0	0	0	0	0	0	1	0	0	1	0	0	0	0	0	0	0	0	1	0	0	1
**7.068**	0	0	0	0	0	0	0	0	0	0	0	1	1	1	0	0	0	0	0	1	0	1	1	0	0	1
**7.069**	0	0	0	0	0	0	0	0	0	0	0	1	0	1	0	1	0	0	0	0	0	0	0	0	1	1
**7.070**	0	1	1	0	0	0	0	0	0	0	0	1	0	0	0	0	0	0	0	0	0	0	1	1	0	1
**7.071**	0	1	0	1	1	0	1	0	0	1	0	1	0	1	0	0	0	0	0	1	0	1	0	0	1	1
**7.072**	0	1	0	0	1	0	0	0	0	1	1	1	0	1	0	0	0	0	0	0	0	1	0	0	1	1
**7.073**	0	1	0	0	1	0	0	0	0	0	0	1	1	0	0	1	0	0	0	0	0	0	1	0	0	1
**7.074**	0	1	0	0	0	0	1	0	0	0	1	1	0	0	0	0	0	0	0	0	0	0	0	0	0	1

CMP	CD2	CD3	CD4	CD7	CD8	CD10	CD13	CD18	CD26	CD29	CD36	CD44	CD45RA	CD49f	CD54	CD56	CD57	CD58	CD62L	CD71	CD80	Cytokeratin	Histone_H1	HLA-DQ	HLA-DR	Freq. (voxel)
**7.075**	0	1	0	0	0	0	0	0	0	1	0	1	1	0	0	0	0	0	0	1	0	0	0	0	1	1
**7.076**	0	0	1	1	0	0	0	0	0	1	1	0	0	1	0	0	0	0	0	0	0	0	0	1	1	1
**7.077**	0	0	1	1	0	0	0	0	0	1	0	1	1	1	0	1	0	0	0	1	0	0	0	1	1	1
**7.078**	0	0	1	1	0	0	0	0	0	0	1	1	0	0	0	0	0	0	0	0	0	0	0	1	0	1
**7.079**	0	0	1	0	0	0	1	0	0	1	0	1	0	0	0	0	0	0	0	1	0	0	0	0	1	1
**7.080**	0	0	1	0	0	0	0	0	1	1	0	1	0	1	0	0	0	0	0	1	0	1	1	1	0	1
**7.081**	0	0	1	0	0	0	0	0	1	1	0	1	0	1	0	0	0	0	0	0	0	1	1	1	0	1
**7.082**	0	0	1	0	0	0	0	0	1	0	0	0	0	1	0	0	0	0	0	0	0	0	1	1	1	1
**7.083**	0	0	1	0	0	0	0	0	1	0	0	0	0	0	0	0	0	0	0	1	0	0	1	0	0	1
**7.084**	0	0	1	0	0	0	0	0	0	1	0	1	0	1	0	1	0	0	0	1	0	1	1	1	0	1
**7.085**	0	0	1	0	0	0	0	0	0	1	0	1	0	1	0	1	0	0	0	1	0	0	0	1	1	1
**7.086**	0	0	1	0	0	0	0	0	0	1	0	1	0	1	0	0	0	0	0	1	0	0	1	1	0	1
**7.087**	0	0	1	0	0	0	0	0	0	0	1	0	0	0	0	0	0	0	0	1	0	0	0	0	0	1
**7.088**	0	0	1	0	0	0	0	0	0	0	0	0	0	0	0	0	0	0	0	0	0	1	1	0	1	1
**7.089**	0	0	0	1	0	0	0	0	1	0	0	0	0	0	0	1	0	0	0	0	0	1	0	0	0	1
**7.090**	0	0	0	0	1	0	1	0	0	0	0	1	1	1	0	0	0	0	0	0	0	1	1	0	0	1
**7.091**	0	0	0	0	1	0	1	0	0	0	0	0	0	1	0	0	0	0	0	0	0	1	1	0	0	1
**7.092**	0	0	0	0	0	0	1	0	1	1	0	1	0	0	0	0	0	0	0	1	0	1	1	0	1	1
**7.093**	0	0	0	0	0	0	1	0	0	1	0	0	1	0	0	1	0	0	0	1	0	0	0	1	0	1
**7.094**	0	0	0	0	0	0	1	0	0	1	0	0	0	0	0	0	0	0	0	1	0	1	0	0	1	1
**7.095**	0	0	0	0	0	0	0	0	1	1	1	0	0	0	0	0	0	0	0	0	0	0	0	1	1	1
**7.096**	0	0	0	0	0	0	0	0	1	1	0	1	0	1	0	1	0	0	0	0	0	0	0	1	1	1
**7.097**	0	0	0	0	0	0	0	0	1	1	0	0	0	1	0	1	0	0	0	0	0	0	0	1	1	1
**7.098**	0	0	0	0	0	0	0	0	0	1	0	0	0	1	0	1	0	0	0	0	0	0	1	1	1	1
**7.099**	0	0	0	0	0	0	0	0	0	0	0	0	0	1	0	1	0	0	0	0	0	0	1	1	1	1
**7.100**	0	1	0	1	1	0	0	0	1	0	0	1	1	0	0	1	0	0	0	1	0	1	0	0	1	1
**7.101**	0	1	0	1	1	0	0	0	1	0	0	1	0	0	0	1	0	0	0	1	0	1	0	0	0	1
**7.102**	0	1	0	1	0	0	1	0	0	0	0	1	0	1	0	0	0	0	0	0	0	0	0	0	0	1
**7.103**	0	1	0	0	1	0	1	0	0	1	0	1	0	1	0	0	0	0	0	0	0	1	1	0	0	1
**7.104**	0	1	0	0	0	0	0	0	1	0	0	1	1	0	0	0	0	0	0	1	0	0	0	0	1	1
**7.105**	0	0	1	0	0	0	1	0	0	0	0	0	0	0	0	0	0	0	0	0	0	1	0	0	1	1
**7.106**	0	0	1	0	0	0	0	0	0	1	0	1	1	1	0	0	0	0	0	1	0	0	0	1	1	1
**7.107**	0	0	0	0	1	0	1	0	0	1	0	1	0	0	0	0	0	0	0	0	0	1	1	0	1	1
**7.108**	0	0	0	0	1	0	1	0	0	0	0	1	0	1	0	0	0	0	0	1	0	1	1	0	0	1
**7.109**	0	0	0	0	1	0	0	0	0	1	0	0	1	0	0	1	0	0	0	1	0	0	0	0	0	1
**7.110**	0	0	0	0	0	0	1	0	0	1	0	0	0	0	0	0	0	0	0	0	0	1	1	0	1	1
**7.111**	0	0	0	0	0	0	0	0	0	0	0	0	1	0	0	0	0	0	0	1	0	1	1	0	0	1
**7.112**	0	1	0	1	1	0	1	0	1	0	0	1	1	0	0	0	0	0	0	1	0	1	0	0	1	1
**7.113**	0	1	0	1	1	0	1	0	0	1	0	1	1	1	0	0	0	0	0	1	0	0	0	0	0	1
**7.114**	0	1	0	1	1	0	1	0	0	1	0	1	1	0	0	0	0	0	0	1	0	0	0	0	0	1
**7.115**	0	1	0	1	1	0	1	0	0	0	0	1	1	1	0	0	0	0	0	1	0	0	0	0	0	1
**7.116**	0	1	0	1	1	0	1	0	0	0	0	1	1	1	0	0	0	0	0	0	0	0	0	0	0	1
**7.117**	0	1	0	1	1	0	0	0	1	0	0	0	0	0	0	0	0	0	0	1	0	0	1	0	1	1
**7.118**	0	1	0	1	0	0	1	0	1	0	0	1	0	0	0	0	0	0	0	1	0	1	0	0	1	1
**7.119**	0	1	0	1	0	0	0	0	1	0	0	1	1	0	0	1	0	0	0	1	0	1	0	0	1	1
**7.120**	0	1	0	1	0	0	0	0	1	0	0	0	0	0	0	0	0	0	0	1	0	0	0	1	0	1
**7.121**	0	1	0	1	0	0	0	0	1	0	0	0	0	0	0	0	0	0	0	0	0	0	0	1	0	1
**7.122**	0	1	0	0	0	0	0	0	1	1	0	0	0	0	0	0	0	0	0	0	0	0	0	1	0	1
**7.123**	0	0	1	0	0	0	1	0	0	0	0	0	1	0	0	1	0	0	0	0	0	0	1	0	0	1
**7.124**	0	0	1	0	0	0	0	0	0	0	1	0	0	1	0	0	0	0	0	0	0	0	0	1	1	1
**7.125**	0	0	0	1	1	0	1	0	0	0	0	1	1	0	0	0	0	0	0	0	0	0	1	0	0	1
**7.126**	0	0	0	1	0	0	0	0	1	0	0	0	1	0	0	1	0	0	0	1	0	1	0	1	0	1
**7.127**	0	0	0	0	1	0	0	0	1	0	0	0	1	0	0	0	0	0	0	0	0	0	1	0	0	1
**7.128**	0	0	0	0	1	0	0	0	0	1	1	1	0	0	0	0	0	0	0	0	0	0	0	1	1	1
**7.129**	0	0	0	0	0	0	1	0	1	0	0	0	1	0	0	0	0	0	0	1	0	0	1	0	0	1
**7.130**	0	0	0	0	0	0	1	0	0	1	0	0	0	0	0	0	0	0	0	1	0	0	1	0	1	1
**7.131**	0	0	0	0	0	0	0	0	0	1	1	0	0	1	0	0	0	0	0	0	0	0	0	1	0	1
**7.132**	0	0	0	0	0	0	0	0	0	1	1	0	0	0	0	0	0	0	0	0	0	1	0	1	1	1
**7.133**	0	1	1	1	0	0	0	0	0	0	0	1	1	0	0	0	0	0	0	0	0	0	1	0	0	1
**7.134**	0	1	0	1	1	0	1	0	0	1	0	1	0	0	0	0	0	0	0	1	0	0	1	0	0	1
**7.135**	0	1	0	1	0	0	1	0	1	0	0	1	1	0	0	0	0	0	0	1	0	1	0	0	0	1
**7.136**	0	1	0	0	1	0	1	0	0	0	0	0	1	0	0	0	0	0	0	0	0	0	1	0	0	1
**7.137**	0	1	0	0	1	0	0	0	0	1	0	1	1	0	0	0	0	0	0	1	0	1	1	0	0	1
**7.138**	0	1	0	0	0	0	0	0	1	0	0	0	0	0	0	0	0	0	0	1	0	0	0	1	0	1
**7.139**	0	0	1	0	0	0	1	0	0	0	1	0	0	0	0	0	0	0	0	0	0	0	0	0	1	1
**7.140**	0	0	1	0	0	0	1	0	0	0	0	0	1	0	0	1	0	0	0	1	0	0	1	0	0	1
**7.141**	0	0	0	1	0	0	0	0	0	0	0	0	0	0	0	1	0	0	0	1	0	1	0	1	0	1
**7.142**	0	0	0	0	0	0	1	0	0	1	0	0	1	0	0	0	0	0	0	1	0	0	0	1	1	1
**7.143**	0	0	0	0	0	0	1	0	0	0	1	0	0	0	0	0	0	0	0	0	0	0	0	0	1	1
**7.144**	0	0	0	0	0	0	1	0	0	0	0	1	1	0	0	0	0	0	0	1	0	1	0	0	0	1
**7.145**	0	0	0	0	0	0	0	0	0	1	0	0	1	0	0	0	0	0	0	1	0	0	0	1	1	1
**7.146**	0	1	0	1	1	0	1	0	0	0	0	1	0	0	0	0	0	0	0	1	0	0	1	0	0	1
**7.147**	0	1	0	1	0	0	1	0	0	1	0	1	1	0	0	0	0	0	0	0	0	0	0	0	0	1
**7.148**	0	1	0	1	0	0	0	0	0	0	0	0	1	0	0	0	0	0	0	0	0	1	0	0	1	1
**7.149**	0	1	0	0	1	0	1	0	0	1	0	1	1	0	0	0	0	0	0	0	0	1	0	0	1	1
**7.150**	0	0	0	0	1	0	1	0	1	0	0	0	0	1	0	0	0	0	0	1	0	0	1	0	0	1
**7.151**	0	0	0	0	1	0	0	0	0	0	0	0	1	0	0	0	0	0	0	0	0	1	1	0	0	1
**7.152**	0	1	1	0	0	0	0	0	0	0	0	0	0	0	0	0	0	0	0	0	0	1	0	1	0	1
**7.153**	0	0	0	1	1	0	1	0	1	0	0	0	0	0	0	0	0	0	0	1	0	0	1	0	0	1
**7.154**	0	0	0	1	1	0	0	0	1	0	0	0	0	0	0	0	0	0	0	1	0	0	1	0	0	1
**7.155**	0	0	0	1	0	0	0	0	1	0	0	0	0	0	0	0	0	0	0	0	0	0	0	0	1	1
**7.156**	0	0	0	0	1	0	0	0	0	0	1	1	0	0	0	0	0	0	0	0	0	0	0	1	0	1
**7.157**	0	0	0	0	0	0	1	0	1	0	0	0	0	0	0	1	0	0	0	1	0	1	0	1	0	1
**7.158**	0	1	0	0	1	0	1	0	0	0	0	1	0	0	0	0	0	0	0	0	0	1	0	0	0	1
**7.159**	0	1	0	0	0	0	1	0	0	1	0	1	1	0	0	0	0	0	0	0	0	1	1	0	0	1
**7.160**	0	0	0	0	1	0	1	0	0	1	0	0	0	0	0	0	0	0	0	0	0	0	0	0	0	1

Supplementary Table 2. Complete list of altogether 7,161 distinct 3D-CMPs expressed in the investigated MF tissue. The CMPs are sorted according to frequency (numbers of voxels expressing the respective CMP).

	CD2	CD3	CD4	CD7	CD8	CD10	CD13	CD18	CD26	CD29	CD36	CD44	CD45RA	CD49f	CD54	CD56	CD57	CD58	CD62L	CD71	CD80	Cytokeratin	Histone_H1	HLA-DQ	HLA-DR	Freq. (Voxel)
**0**	0	0	0	0	0	0	0	0	0	0	0	0	0	0	0	0	0	0	0	0	0	0	0	1	1	112.817
**1**	0	0	0	0	0	0	0	0	0	0	0	0	0	0	0	0	0	0	0	0	0	0	0	1	0	75.525
**2**	0	0	0	0	0	0	0	0	0	0	0	1	0	0	0	0	0	0	0	0	0	0	0	1	1	36.192
**3**	0	0	0	0	0	0	0	0	0	0	0	0	0	0	0	0	0	0	0	0	0	1	0	1	1	32.158
**4**	0	0	0	0	0	0	0	0	0	0	0	0	0	0	0	0	0	0	0	0	0	1	0	1	0	30.319
**5**	0	0	0	0	0	0	0	0	0	0	0	1	0	0	0	0	0	0	0	0	0	1	0	1	1	28.916
**6**	0	0	0	0	0	0	0	0	0	0	0	0	0	0	0	0	0	0	0	0	0	0	1	1	0	28.324
**7**	0	0	0	0	0	0	0	0	0	0	0	1	0	0	0	0	0	0	0	0	0	0	0	1	0	22.854
**8**	0	0	0	0	0	0	0	0	0	0	0	0	0	0	0	0	0	0	0	0	0	0	1	1	1	17.435
**9**	0	0	0	0	0	0	0	0	0	0	0	1	0	0	0	0	0	0	0	0	0	1	0	1	0	17.400
**10**	0	0	0	0	0	0	0	0	0	1	0	1	0	0	0	0	0	0	0	0	0	0	0	1	1	12.770
**11**	0	0	0	0	0	0	0	0	0	1	0	1	0	0	0	0	0	0	0	0	0	0	0	1	0	10.998
**12**	0	0	0	0	0	0	1	0	0	0	0	1	0	0	0	0	0	0	0	0	0	0	0	1	1	7.826
**13**	0	0	0	0	0	0	1	0	0	0	0	0	0	0	0	0	0	0	0	0	0	0	0	1	1	5.895
**14**	0	0	0	0	0	0	0	0	0	1	0	1	0	0	0	0	0	0	0	1	0	0	0	1	1	4.748
**15**	0	0	0	0	0	0	0	0	1	0	0	1	0	0	0	0	0	0	0	0	0	1	0	1	1	3.700
**16**	0	0	1	0	0	0	0	0	0	0	0	0	0	0	0	0	0	0	0	0	0	0	0	1	0	3.298
**17**	0	0	0	0	0	0	0	0	0	0	0	0	0	1	0	0	0	0	0	0	0	0	0	1	1	3.290
**18**	0	0	0	0	0	0	0	0	0	0	0	1	0	0	0	0	0	0	0	1	0	1	0	1	1	3.215
**19**	0	0	0	0	0	0	0	0	0	1	0	1	0	0	0	0	0	0	0	1	0	0	0	1	0	3.103
**20**	0	0	0	0	0	0	1	0	0	0	0	1	0	0	0	0	0	0	0	0	0	0	0	1	0	2.997
**21**	0	0	0	0	0	0	0	0	0	0	0	0	0	0	0	0	0	0	0	0	0	1	1	1	0	2.878
**22**	0	0	0	0	0	0	1	0	0	0	0	0	0	0	0	0	0	0	0	0	0	0	0	1	0	2.835
**23**	0	0	1	0	0	0	0	0	0	0	0	0	0	0	0	0	0	0	0	0	0	0	0	1	1	2.763
**24**	0	0	0	0	0	0	0	0	0	0	0	0	0	0	0	0	0	0	0	1	0	0	0	1	1	2.403
**25**	0	0	0	0	0	0	0	0	1	0	0	1	0	0	0	0	0	0	0	0	0	1	0	1	0	2.355
**26**	0	0	0	0	0	0	0	0	0	0	0	1	0	0	0	0	0	0	0	0	0	0	1	1	0	2.153
**27**	0	0	0	0	0	0	0	0	0	0	1	0	0	0	0	0	0	0	0	0	0	0	0	1	0	2.072
**28**	0	0	0	0	0	0	0	0	0	0	0	1	0	0	0	0	0	0	0	1	0	0	0	1	1	2.017
**29**	0	0	0	0	0	0	0	0	0	1	0	1	0	0	0	0	0	0	0	0	0	1	0	1	0	1.943
**30**	0	0	0	0	0	0	1	0	0	1	0	1	0	1	0	0	0	0	0	0	0	0	0	1	1	1.861
**31**	0	0	0	0	0	0	1	0	0	0	0	1	0	0	0	0	0	0	0	1	0	0	0	1	1	1.827
**32**	0	0	0	0	0	0	0	0	0	0	0	0	0	1	0	0	0	0	0	0	0	0	0	1	0	1.801
**33**	0	0	0	0	0	0	0	0	0	0	0	0	0	0	0	0	0	0	0	1	0	1	0	1	1	1.801
**34**	0	0	0	0	0	0	0	0	0	0	0	0	0	0	0	0	0	0	0	1	0	1	0	1	0	1.792
**35**	0	0	0	0	0	0	0	0	0	1	0	1	0	0	0	0	0	0	0	0	0	1	0	1	1	1.694
**36**	0	0	0	0	0	0	1	0	0	1	0	1	0	1	0	0	0	0	0	0	0	0	0	1	0	1.666
**37**	0	0	0	0	0	0	0	0	0	0	0	1	0	0	0	0	0	0	0	1	0	1	0	1	0	1.649
**38**	0	1	0	0	1	0	0	0	0	0	0	1	0	0	0	0	0	0	0	0	0	0	0	1	1	1.569
**39**	0	0	0	0	0	0	0	0	0	0	0	1	0	0	0	0	0	0	0	0	0	0	1	1	1	1.544
**40**	0	0	0	0	1	0	0	0	0	0	0	1	0	0	0	0	0	0	0	0	0	0	0	1	1	1.528
**41**	0	0	0	0	0	0	0	0	0	1	0	0	0	0	0	0	0	0	0	0	0	0	0	1	1	1.467
**42**	0	0	0	0	0	0	0	0	0	1	0	0	0	0	0	0	0	0	0	0	0	0	0	1	0	1.444
**43**	0	0	0	0	0	0	0	0	0	0	1	0	0	0	0	0	0	0	0	0	0	0	0	1	1	1.442
**44**	0	0	0	0	0	0	0	0	1	0	0	1	0	0	0	0	0	0	0	1	0	1	0	1	1	1.424
**45**	0	0	0	0	0	0	0	0	0	0	0	0	0	0	0	0	0	0	0	1	0	0	0	1	0	1.388
**46**	0	1	0	0	0	0	0	0	0	0	0	1	0	0	0	0	0	0	0	0	0	0	0	1	1	1.366
**47**	0	0	0	0	0	0	0	0	1	0	0	0	0	0	0	0	0	0	0	0	0	1	0	1	0	1.286
**48**	0	0	0	0	0	0	0	0	0	1	0	1	0	1	0	0	0	0	0	0	0	0	0	1	0	1.266
**49**	0	0	0	0	0	0	0	0	0	0	0	0	1	0	0	0	0	0	0	0	0	0	0	1	0	1.265
**50**	0	0	0	0	0	0	0	0	0	0	0	1	0	1	0	0	0	0	0	0	0	0	0	1	1	1.232
**51**	0	0	1	0	0	0	0	0	0	0	0	1	0	0	0	0	0	0	0	0	0	0	0	1	1	1.176
**52**	0	0	0	0	0	0	0	0	0	0	0	1	0	1	0	0	0	0	0	0	0	0	0	1	0	1.161
**53**	0	0	0	0	0	0	0	0	1	0	0	0	0	0	0	0	0	0	0	0	0	1	0	1	1	1.112
**54**	0	0	0	0	0	0	0	0	1	0	0	1	0	0	0	0	0	0	0	0	0	0	0	1	1	1.067
**55**	0	0	0	0	0	0	0	0	0	0	0	1	0	0	0	1	0	0	0	0	0	0	0	1	1	1.058
**56**	0	0	0	0	0	0	1	0	0	1	0	1	0	0	0	0	0	0	0	0	0	0	0	1	1	1.022
**57**	0	0	0	0	0	0	0	0	0	1	0	1	0	1	0	0	0	0	0	0	0	0	0	1	1	1.004
**58**	0	0	0	0	0	0	1	0	0	0	0	1	0	1	0	0	0	0	0	0	0	0	0	1	1	957
**59**	0	0	0	0	0	0	0	0	0	0	0	0	0	0	0	1	0	0	0	0	0	0	0	1	1	913
**60**	0	0	0	0	0	0	0	0	0	0	0	0	0	1	0	0	0	0	0	0	0	1	0	1	1	908
**61**	0	0	0	0	0	0	0	0	0	0	0	0	0	0	0	0	0	0	0	0	0	1	1	1	1	906
**62**	0	1	0	0	0	0	0	0	0	0	0	1	0	0	0	0	0	0	0	0	0	0	0	1	0	898
**63**	0	0	0	0	0	0	0	0	0	0	0	0	1	0	0	1	0	0	0	0	0	0	0	1	0	893
**64**	0	0	0	0	0	0	1	0	0	1	0	1	0	1	0	0	0	0	0	1	0	0	0	1	1	886
**65**	0	1	0	0	0	0	0	0	0	0	0	1	0	0	0	0	0	0	0	0	0	1	0	1	1	881
**66**	0	0	0	0	0	0	0	0	0	1	0	1	0	0	0	0	0	0	0	0	0	0	1	1	0	870
**67**	0	0	0	0	0	0	1	0	1	0	0	1	0	0	0	0	0	0	0	0	0	0	0	1	1	851
**68**	0	0	0	0	0	0	0	0	0	0	1	1	0	0	0	0	0	0	0	0	0	0	0	1	1	848
**69**	0	0	0	0	0	0	0	0	0	1	0	0	0	0	0	0	0	0	0	0	0	0	1	1	0	847
**70**	0	0	0	0	0	0	1	0	0	1	0	1	0	0	0	0	0	0	0	0	0	0	0	1	0	830
**71**	0	1	0	0	1	0	0	0	0	1	0	1	0	0	0	0	0	0	0	0	0	0	0	1	1	816
**72**	0	1	0	0	1	0	0	0	1	0	0	1	0	0	0	0	0	0	0	0	0	0	0	1	1	797
**73**	0	0	0	0	0	0	0	0	1	0	0	1	0	0	0	0	0	0	0	1	0	1	0	1	0	767
**74**	0	0	0	0	0	0	0	0	0	0	0	1	0	0	0	0	0	0	0	1	0	0	0	1	0	761
**75**	0	0	1	0	0	0	0	0	0	1	0	1	0	0	0	0	0	0	0	1	0	0	0	1	1	760
**76**	0	0	0	0	0	0	0	0	0	0	0	0	0	1	0	0	0	0	0	0	0	1	0	1	0	748
**77**	0	0	0	0	0	0	0	0	0	1	0	1	0	1	0	0	0	0	0	1	0	0	0	1	1	739
**78**	0	0	1	0	0	0	1	0	0	1	0	1	0	1	0	0	0	0	0	0	0	0	0	1	1	719
**79**	0	0	0	0	0	0	0	0	1	0	0	0	0	0	0	0	0	0	0	0	0	0	0	1	1	700
**80**	0	0	0	0	0	0	1	0	0	1	0	1	0	0	0	0	0	0	0	1	0	0	0	1	1	673
**81**	0	0	0	0	0	0	1	0	0	0	0	0	0	1	0	0	0	0	0	0	0	0	0	1	1	667
**82**	0	0	0	0	1	0	0	0	0	1	0	1	0	0	0	0	0	0	0	0	0	0	0	1	1	644
**83**	0	0	0	0	0	0	0	0	0	0	0	0	0	0	0	1	0	0	0	0	0	0	0	1	0	636
**84**	0	0	0	0	0	0	0	0	0	1	1	1	0	0	0	0	0	0	0	0	0	0	0	1	1	627
**85**	0	0	0	0	0	0	0	0	0	0	0	1	0	1	0	0	0	0	0	0	0	1	0	1	1	613
**86**	0	0	1	0	0	0	1	0	0	1	0	1	0	1	0	0	0	0	0	1	0	0	0	1	1	611
**87**	0	0	1	0	0	0	0	0	0	0	0	1	0	0	0	0	0	0	0	0	0	1	0	1	1	603
**88**	0	0	1	0	0	0	0	0	0	1	0	1	0	0	0	0	0	0	0	0	0	0	0	1	1	593
**89**	0	0	1	0	0	0	0	0	0	0	0	0	1	0	0	0	0	0	0	0	0	0	0	1	0	584
**90**	0	0	0	0	0	0	0	0	1	1	0	1	0	0	0	0	0	0	0	0	0	0	0	1	1	570
**91**	0	0	0	0	0	0	0	0	0	0	0	1	0	0	0	0	0	0	0	0	0	1	1	1	1	563
**92**	0	0	0	0	0	0	1	0	0	0	0	1	0	0	0	1	0	0	0	0	0	0	0	1	1	554
**93**	0	1	0	0	1	0	0	0	0	0	0	1	0	0	0	0	0	0	0	0	0	0	0	1	0	554
**94**	0	0	0	0	0	0	0	0	0	1	0	1	0	1	0	0	0	0	0	1	0	0	0	1	0	540
**95**	0	1	0	0	1	0	0	0	0	0	0	1	1	0	0	0	0	0	0	0	0	0	0	1	1	521
**96**	0	1	0	0	1	0	0	0	0	0	0	1	0	0	0	0	0	0	0	0	0	1	0	1	1	521
**97**	0	0	0	0	0	0	0	0	0	0	1	1	0	0	0	0	0	0	0	0	0	0	0	1	0	521

	CD2	CD3	CD4	CD7	CD8	CD10	CD13	CD18	CD26	CD29	CD36	CD44	CD45RA	CD49f	CD54	CD56	CD57	CD58	CD62L	CD71	CD80	Cytokeratin	Histone_H1	HLA-DQ	HLA-DR	Freq. (Voxel)
**98**	0	0	0	0	0	0	0	0	0	0	1	0	0	0	0	0	0	0	0	0	0	1	0	1	0	517
**99**	0	0	0	0	1	0	0	0	0	0	0	1	0	0	0	0	0	0	0	0	0	1	0	1	1	515
**100**	0	0	0	0	0	0	1	0	0	0	0	0	0	0	0	0	0	0	0	1	0	0	0	1	1	514
**101**	0	0	0	0	0	0	0	0	0	0	0	1	0	0	0	0	0	0	0	0	0	1	1	1	0	508
**102**	0	0	0	0	0	0	0	0	0	0	0	0	0	0	0	0	0	0	0	1	0	0	1	1	0	502
**103**	0	1	0	0	0	0	0	0	0	1	0	1	0	0	0	0	0	0	0	0	0	0	0	1	0	498
**104**	0	0	0	0	1	0	0	0	0	0	0	1	0	0	0	0	0	0	0	0	0	0	0	1	0	494
**105**	0	0	1	0	0	0	0	0	0	0	0	0	0	0	0	0	0	0	0	0	0	1	0	1	0	494
**106**	0	0	0	0	0	0	0	0	0	1	0	0	0	1	0	0	0	0	0	0	0	0	0	1	0	489
**107**	0	1	0	1	1	0	0	0	1	1	0	1	0	0	0	0	0	0	0	1	0	0	0	1	1	471
**108**	0	1	0	1	1	0	0	0	0	1	0	1	0	0	0	0	0	0	0	1	0	0	0	1	1	457
**109**	0	0	0	0	0	0	0	0	0	0	1	0	0	0	0	0	0	0	0	0	0	1	0	1	1	456
**110**	0	1	0	0	1	0	0	0	1	1	0	1	0	0	0	0	0	0	0	0	0	0	0	1	1	455
**111**	0	0	1	0	0	0	0	0	0	0	0	1	0	0	0	0	0	0	0	1	0	0	0	1	1	454
**112**	0	0	0	0	0	0	0	0	0	0	0	0	0	0	0	0	0	0	0	1	0	0	1	1	1	453
**113**	0	0	0	0	0	0	0	0	0	1	0	1	0	0	0	0	0	0	0	0	0	0	1	1	1	451
**114**	0	0	0	0	0	0	0	0	0	0	0	0	0	1	0	0	0	0	0	0	0	0	1	1	0	441
**115**	0	0	0	0	0	0	0	0	1	0	0	0	0	0	0	0	0	0	0	1	0	1	0	1	1	439
**116**	0	0	1	0	0	0	0	0	0	0	0	0	1	0	0	1	0	0	0	0	0	0	0	1	0	426
**117**	0	1	0	0	0	0	0	0	0	1	0	1	0	0	0	0	0	0	0	0	0	0	0	1	1	423
**118**	0	1	0	0	1	0	0	0	0	1	0	1	0	0	0	0	0	0	0	1	0	0	0	1	1	417
**119**	0	0	1	0	0	0	0	0	0	0	0	0	0	0	0	0	0	0	0	1	0	0	0	1	1	416
**120**	0	0	0	0	0	0	0	0	1	1	0	1	0	0	0	0	0	0	0	1	0	0	0	1	1	411
**121**	0	0	0	0	0	0	0	0	0	1	0	0	0	0	0	0	0	0	0	0	0	0	1	1	1	409
**122**	0	0	1	0	0	0	1	0	0	0	0	1	0	0	0	0	0	0	0	0	0	0	0	1	1	404
**123**	0	0	1	0	0	0	0	0	0	0	0	0	0	0	0	1	0	0	0	0	0	0	0	1	0	403
**124**	0	1	0	0	0	0	0	0	0	0	0	1	0	0	0	0	0	0	0	0	0	0	1	1	0	396
**125**	0	0	1	0	0	0	0	0	0	0	0	0	0	0	0	0	0	0	0	0	0	1	0	1	1	393
**126**	0	0	0	0	0	0	0	0	0	0	0	1	0	1	0	0	0	0	0	0	0	1	0	1	0	384
**127**	0	0	0	0	0	0	0	0	0	0	0	0	1	0	0	0	0	0	0	0	0	1	0	1	0	373
**128**	0	0	0	0	0	0	0	0	0	1	0	1	0	0	0	0	0	0	0	1	0	1	0	1	1	358
**129**	0	0	0	0	1	0	0	0	0	1	0	1	0	0	0	0	0	0	0	0	0	0	0	1	0	357
**130**	0	0	0	0	0	0	1	0	0	0	0	0	0	1	0	0	0	0	0	0	0	0	0	1	0	353
**131**	0	0	1	0	0	0	1	0	0	1	0	1	0	0	0	0	0	0	0	1	0	0	0	1	1	349
**132**	0	0	0	0	0	0	0	0	0	1	0	1	0	0	0	0	0	0	0	1	0	1	0	1	0	348
**133**	0	1	0	0	1	0	0	0	0	1	0	1	0	0	0	0	0	0	0	0	0	0	0	1	0	343
**134**	0	0	0	0	0	0	0	0	0	1	1	1	0	0	0	0	0	0	0	0	0	0	0	1	0	334
**135**	0	1	0	1	1	0	0	0	0	0	0	1	0	0	0	0	0	0	0	1	0	0	0	1	1	333
**136**	0	1	0	0	1	0	0	0	0	0	0	1	0	0	0	0	0	0	0	1	0	0	0	1	1	332
**137**	0	0	0	0	0	0	0	0	0	0	0	0	0	1	0	0	0	0	0	0	0	0	1	1	1	330
**138**	0	0	0	0	0	0	0	0	0	1	0	1	0	1	0	0	0	0	0	1	0	1	0	1	1	330
**139**	0	0	0	0	0	0	0	0	1	1	0	1	0	0	0	0	0	0	0	0	0	0	0	1	0	323
**140**	0	0	0	0	0	0	0	0	1	0	0	0	0	0	0	0	0	0	0	1	0	1	0	1	0	318
**141**	0	0	0	0	0	0	0	0	0	0	0	0	0	0	0	1	0	0	0	0	0	0	1	1	0	312
**142**	0	1	0	0	0	0	0	0	0	0	0	1	0	0	0	0	0	0	0	0	0	0	1	1	1	306
**143**	0	0	1	0	0	0	0	0	0	0	0	1	0	0	0	0	0	0	0	0	0	0	0	1	0	304
**144**	0	1	0	0	1	0	0	0	1	1	0	1	0	0	0	0	0	0	0	1	0	0	0	1	1	299
**145**	0	1	0	0	1	0	0	0	1	0	0	1	0	0	0	0	0	0	0	0	0	0	0	1	0	297
**146**	0	1	0	0	1	0	0	0	0	0	0	1	0	0	0	0	0	0	0	0	0	0	1	1	1	294
**147**	0	0	1	0	0	0	1	0	0	0	0	1	0	0	0	0	0	0	0	1	0	0	0	1	1	294
**148**	0	1	0	1	1	0	0	0	0	1	0	1	0	0	0	0	0	0	0	0	0	0	0	1	1	293
**149**	0	0	0	0	0	0	1	0	1	0	0	0	0	0	0	0	0	0	0	0	0	0	0	1	1	292
**150**	0	1	0	0	0	0	0	0	1	0	0	1	0	0	0	0	0	0	0	0	0	1	0	1	1	292
**151**	0	0	0	0	0	0	0	0	1	1	0	1	0	0	0	0	0	0	0	1	0	1	0	1	1	292
**152**	0	0	1	0	0	0	0	0	0	0	0	1	0	0	0	0	0	0	0	0	0	1	0	1	0	292
**153**	0	0	1	0	0	0	0	0	0	1	0	0	0	0	0	0	0	0	0	0	0	0	0	1	1	290
**154**	0	0	0	0	1	0	0	0	1	0	0	1	0	0	0	0	0	0	0	0	0	0	0	1	1	289
**155**	0	0	0	0	0	0	0	0	0	1	0	0	0	0	0	0	0	0	0	1	0	0	0	1	0	284
**156**	0	1	0	0	0	0	0	0	0	0	0	0	0	0	0	0	0	0	0	0	0	0	0	1	1	281
**157**	0	0	0	0	0	0	0	0	1	0	0	1	0	0	0	0	0	0	0	1	0	0	0	1	1	277
**158**	0	0	1	0	0	0	0	0	0	0	0	0	0	1	0	0	0	0	0	0	0	0	0	1	1	275
**159**	0	0	0	0	0	0	0	0	0	0	0	0	1	0	0	0	0	0	0	1	0	0	0	1	0	257
**160**	0	0	1	0	0	0	1	0	0	1	0	1	0	0	0	0	0	0	0	0	0	0	0	1	1	256
**161**	0	0	0	0	0	0	0	0	1	0	0	1	0	0	0	0	0	0	0	0	0	0	0	1	0	254
**162**	0	0	0	0	0	0	0	0	0	0	0	0	0	0	0	1	0	0	0	0	0	0	1	1	1	253
**163**	0	0	0	0	0	0	1	0	0	0	0	0	0	0	0	0	0	0	0	0	0	0	1	1	1	253
**164**	0	0	0	0	0	0	0	0	0	0	0	0	0	1	0	0	0	0	0	1	0	0	0	1	1	252
**165**	0	0	0	0	0	0	0	0	0	1	0	1	0	1	0	0	0	0	0	1	0	1	0	1	0	252
**166**	0	0	0	0	0	0	0	0	0	1	0	0	0	0	0	0	0	0	0	1	0	0	0	1	1	251
**167**	0	1	0	0	0	0	0	0	0	0	0	1	0	0	0	0	0	0	0	0	0	1	1	1	1	249
**168**	0	0	0	0	0	0	0	0	0	1	0	1	0	1	0	0	0	0	0	0	0	1	0	1	1	249
**169**	0	0	1	0	0	0	1	0	1	1	0	1	0	1	0	0	0	0	0	1	0	0	0	1	1	243
**170**	0	0	1	0	0	0	1	0	0	0	0	1	0	1	0	0	0	0	0	0	0	0	0	1	1	242
**171**	0	0	1	0	0	0	0	0	0	1	0	0	0	0	0	0	0	0	0	0	0	0	0	1	0	240
**172**	0	0	0	0	1	0	0	0	0	1	0	1	0	0	0	0	0	0	0	1	0	0	0	1	1	240
**173**	0	1	0	0	0	0	0	0	0	0	0	1	0	0	0	0	0	0	0	0	0	1	0	1	0	237
**174**	0	0	1	0	0	0	0	0	0	0	0	0	0	0	0	0	0	0	0	1	0	0	0	1	0	233
**175**	0	0	0	0	0	0	0	0	0	1	0	1	0	1	0	0	0	0	0	0	0	1	0	1	0	232
**176**	0	0	0	0	0	0	0	0	0	1	1	1	0	1	0	0	0	0	0	0	0	0	0	1	1	231
**177**	0	1	0	0	1	0	0	0	1	0	0	1	0	0	0	0	0	0	0	0	0	1	0	1	1	229
**178**	0	1	0	0	0	0	0	0	0	0	0	1	0	0	0	0	0	0	0	1	0	0	0	1	1	225
**179**	0	0	0	0	0	0	0	0	1	1	0	1	0	0	0	0	0	0	0	0	0	1	0	1	0	222
**180**	0	1	0	0	1	0	0	0	0	1	0	1	1	0	0	0	0	0	0	0	0	0	0	1	1	221
**181**	0	0	0	0	0	0	1	0	1	0	0	1	0	0	0	0	0	0	0	1	0	0	0	1	1	219
**182**	0	0	0	0	0	0	0	0	0	0	0	0	1	0	0	1	0	0	0	1	0	0	0	1	0	210
**183**	0	0	0	0	0	0	1	0	0	0	0	1	0	1	0	0	0	0	0	0	0	0	0	1	0	208
**184**	0	0	0	0	0	0	0	0	1	1	0	1	0	0	0	0	0	0	0	0	0	1	0	1	1	205
**185**	0	0	0	0	0	0	1	0	0	0	0	1	0	0	0	0	0	0	0	0	0	1	0	1	1	202
**186**	0	0	0	0	0	0	1	0	0	0	0	0	0	0	0	0	0	0	0	0	0	1	0	1	1	200
**187**	0	0	0	0	0	0	0	0	1	1	0	1	0	1	0	0	0	0	0	1	0	1	0	1	1	199
**188**	0	1	0	0	1	0	0	0	0	0	0	1	1	0	0	0	0	0	0	0	0	1	0	1	1	198
**189**	0	0	0	0	0	0	0	0	0	0	0	1	0	0	0	1	0	0	0	0	0	0	0	1	0	197
**190**	0	0	1	0	0	0	1	0	0	0	0	0	0	0	0	0	0	0	0	0	0	0	0	1	1	197
**191**	0	1	0	0	0	0	0	0	0	0	0	0	0	0	0	0	0	0	0	0	0	0	1	1	1	193
**192**	0	1	0	0	1	0	0	0	1	0	0	1	0	0	0	0	0	0	0	1	0	0	0	1	1	191
**193**	0	0	0	0	0	0	0	0	0	0	0	0	1	0	0	1	0	0	0	0	0	1	0	1	0	190
**194**	0	0	0	0	0	0	1	0	0	1	1	1	0	1	0	0	0	0	0	0	0	0	0	1	1	187
**195**	0	0	1	1	0	0	1	0	1	1	0	1	1	1	0	1	0	0	0	1	0	0	0	1	1	186

	CD2	CD3	CD4	CD7	CD8	CD10	CD13	CD18	CD26	CD29	CD36	CD44	CD45RA	CD49f	CD54	CD56	CD57	CD58	CD62L	CD71	CD80	Cytokeratin	Histone_H1	HLA-DQ	HLA-DR	Freq. (Voxel)
**196**	0	0	0	0	0	0	1	0	0	1	0	1	0	1	0	0	0	0	0	1	0	0	0	1	0	186
**197**	0	0	1	0	0	0	0	0	0	1	0	1	0	1	0	0	0	0	0	0	0	0	0	1	1	186
**198**	0	0	1	0	0	0	0	0	0	0	0	0	0	0	0	0	0	0	0	0	0	0	1	1	0	185
**199**	0	0	0	0	0	0	0	0	0	0	1	1	0	0	0	0	0	0	0	0	0	1	0	1	1	184
**200**	0	1	0	0	1	0	0	0	1	1	0	1	0	0	0	0	0	0	0	0	0	0	0	1	0	182
**201**	0	1	0	1	1	0	0	0	1	1	0	1	0	0	0	0	0	0	0	0	0	0	0	1	1	180
**202**	0	1	0	1	1	0	0	0	0	0	0	1	0	0	0	0	0	0	0	0	0	0	0	1	1	180
**203**	0	1	0	0	1	0	0	0	1	0	0	1	1	0	0	0	0	0	0	0	0	0	0	1	1	179
**204**	0	0	0	0	0	0	0	0	0	1	0	0	0	1	0	0	0	0	0	0	0	0	0	1	1	179
**205**	0	0	1	0	0	0	0	0	0	0	0	0	0	0	0	0	0	0	0	0	0	0	1	1	1	175
**206**	0	1	0	0	0	0	0	0	0	0	0	1	0	0	0	0	0	0	0	1	0	1	0	1	1	175
**207**	0	0	0	0	0	0	0	0	0	0	0	1	0	1	0	0	0	0	0	1	0	1	0	1	1	174
**208**	0	1	0	1	1	0	0	0	1	0	0	1	0	0	0	0	0	0	0	1	0	1	0	1	1	173
**209**	0	0	0	0	0	0	0	0	1	0	0	1	0	1	0	0	0	0	0	0	0	1	0	1	1	171
**210**	0	1	0	0	0	0	0	0	1	0	0	1	0	0	0	0	0	0	0	0	0	0	0	1	0	169
**211**	0	1	0	0	0	0	0	0	0	0	0	1	0	1	0	0	0	0	0	0	0	0	0	1	1	168
**212**	0	1	0	0	0	0	0	0	1	0	0	1	0	0	0	0	0	0	0	0	0	0	0	1	1	168
**213**	0	1	0	1	1	0	0	0	0	0	0	1	1	0	0	0	0	0	0	0	0	0	0	1	1	167
**214**	0	0	0	0	0	0	1	0	0	1	0	1	0	1	0	0	0	0	0	0	0	0	1	1	1	163
**215**	0	0	0	0	0	0	0	0	0	1	0	0	0	0	0	0	0	0	0	0	0	1	0	1	0	163
**216**	0	1	0	0	1	0	0	0	0	0	0	1	0	0	0	0	0	0	0	0	0	0	1	1	0	163
**217**	0	0	0	0	1	0	0	0	0	0	0	1	0	0	0	0	0	0	0	1	0	0	0	1	1	162
**218**	0	0	0	0	0	0	1	0	1	1	0	1	0	0	0	0	0	0	0	0	0	0	0	1	1	159
**219**	0	1	0	0	0	0	0	0	0	1	0	1	0	0	0	0	0	0	0	1	0	0	0	1	0	158
**220**	0	1	0	1	1	0	0	0	0	0	0	1	1	0	0	0	0	0	0	1	0	0	0	1	1	157
**221**	0	0	0	0	1	0	0	0	0	0	0	0	0	0	0	0	0	0	0	0	0	0	0	1	1	156
**222**	0	0	0	0	0	0	0	0	1	0	0	1	0	1	0	0	0	0	0	1	0	1	0	1	1	154
**223**	0	0	0	0	0	0	0	0	1	0	0	0	0	0	0	0	0	0	0	1	0	0	0	1	1	154
**224**	0	0	1	0	0	0	0	0	1	0	0	1	0	0	0	0	0	0	0	0	0	1	0	1	1	153
**225**	0	0	0	0	0	0	0	0	0	0	1	1	0	0	0	0	0	0	0	0	0	1	0	1	0	152
**226**	0	0	0	0	0	0	0	0	0	0	0	1	0	0	0	0	0	0	0	1	0	1	1	1	1	151
**227**	0	1	0	0	0	0	0	0	0	0	0	1	0	0	0	0	0	0	0	0	0	1	1	1	0	150
**228**	0	1	0	0	0	0	0	0	0	0	0	0	0	0	0	0	0	0	0	0	0	0	1	1	0	147
**229**	0	0	1	0	0	0	0	0	0	1	0	1	0	0	0	0	0	0	0	0	0	0	0	1	0	147
**230**	0	0	0	1	1	0	0	0	0	0	0	1	0	0	0	0	0	0	0	0	0	0	0	1	1	145
**231**	0	0	1	1	0	0	1	0	1	1	0	1	1	1	0	0	0	0	0	1	0	0	0	1	1	144
**232**	0	0	1	0	0	0	0	0	1	0	0	0	0	0	0	0	0	0	0	0	0	0	0	1	1	143
**233**	0	1	0	0	0	0	0	0	0	0	0	0	0	0	0	0	0	0	0	0	0	0	0	1	0	142
**234**	0	0	0	0	0	0	0	0	0	0	1	0	0	0	0	0	0	0	0	0	0	1	1	1	0	138
**235**	0	0	0	0	0	0	0	0	0	0	0	0	0	0	0	0	0	0	0	1	0	1	1	1	0	138
**236**	0	1	0	1	1	0	0	0	1	0	0	1	1	0	0	0	0	0	0	1	0	0	0	1	1	137
**237**	0	0	0	0	1	0	0	0	0	1	0	1	0	0	0	0	0	0	0	0	0	1	0	1	1	137
**238**	0	0	0	0	0	0	1	0	0	1	0	0	0	1	0	0	0	0	0	0	0	0	0	1	0	136
**239**	0	0	0	0	0	0	1	0	1	0	0	1	0	1	0	0	0	0	0	0	0	0	0	1	1	135
**240**	0	0	0	0	0	0	1	0	0	0	0	0	0	0	0	1	0	0	0	0	0	0	0	1	1	135
**241**	0	0	1	0	0	0	0	0	0	0	0	1	0	1	0	0	0	0	0	0	0	0	0	1	1	132
**242**	0	0	0	1	0	0	0	0	0	1	0	1	0	0	0	0	0	0	0	0	0	0	0	1	1	131
**243**	0	0	0	0	0	0	0	0	0	0	0	0	1	0	0	0	0	0	0	0	0	0	0	1	1	131
**244**	0	0	1	0	0	0	1	0	0	1	0	1	0	1	0	0	0	0	0	0	0	0	0	1	0	129
**245**	0	0	0	0	0	0	0	0	0	0	1	1	0	1	0	0	0	0	0	0	0	0	0	1	1	129
**246**	0	1	0	1	1	0	0	0	1	0	0	1	0	0	0	0	0	0	0	1	0	0	0	1	1	129
**247**	0	1	0	0	1	0	0	0	0	0	0	1	1	0	0	0	0	0	0	0	0	0	1	1	1	128
**248**	0	1	0	0	1	0	0	0	0	1	0	1	1	0	0	0	0	0	0	1	0	0	0	1	1	128
**249**	0	0	0	0	1	0	0	0	0	0	0	1	1	0	0	0	0	0	0	0	0	0	0	1	1	127
**250**	0	0	0	0	0	0	1	0	0	1	0	0	0	1	0	0	0	0	0	0	0	0	0	1	1	127
**251**	0	0	1	0	0	0	1	0	1	1	0	1	0	1	0	0	0	0	0	1	0	1	0	1	1	126
**252**	0	0	0	0	0	0	0	0	1	0	0	0	0	0	0	0	0	0	0	0	0	0	0	1	0	126
**253**	0	0	0	0	0	0	1	0	0	1	0	1	0	1	0	0	0	0	0	1	0	1	0	1	1	125
**254**	0	1	0	0	0	0	0	0	1	1	0	1	0	0	0	0	0	0	0	0	0	0	0	1	1	124
**255**	0	0	1	0	0	0	0	0	0	1	0	1	0	1	0	0	0	0	0	1	0	0	0	1	1	122
**256**	0	0	0	0	0	0	0	0	0	0	0	1	0	0	0	0	0	0	0	1	0	0	1	1	1	121
**257**	0	0	0	0	0	0	0	0	0	1	0	0	0	0	0	0	0	0	0	0	0	1	0	1	1	119
**258**	0	0	0	1	0	0	0	0	0	0	0	1	0	0	0	0	0	0	0	0	0	0	0	1	1	119
**259**	0	0	1	0	0	0	0	0	0	1	0	0	0	1	0	0	0	0	0	0	0	0	0	1	0	118
**260**	0	1	0	1	1	0	1	0	0	0	0	1	0	0	0	0	0	0	0	1	0	0	0	1	1	118
**261**	0	0	0	0	1	0	0	0	1	0	0	1	0	0	0	0	0	0	0	0	0	1	0	1	1	118
**262**	0	0	1	0	0	0	1	0	0	1	0	1	0	1	0	0	0	0	0	1	0	1	0	1	1	117
**263**	0	0	1	0	0	0	0	0	1	0	0	1	0	0	0	0	0	0	0	0	0	0	0	1	1	117
**264**	0	1	0	1	1	0	0	0	0	1	0	1	0	0	0	0	0	0	0	0	0	0	0	1	0	116
**265**	0	0	0	0	0	0	1	0	0	0	0	0	1	0	0	0	0	0	0	0	0	0	0	1	0	115
**266**	0	0	0	0	0	0	1	0	0	0	0	1	0	1	0	0	0	0	0	1	0	0	0	1	1	115
**267**	0	1	0	0	1	0	0	0	1	0	0	1	1	0	0	0	0	0	0	0	0	1	0	1	1	114
**268**	0	0	0	0	0	0	0	0	0	0	0	1	0	1	0	0	0	0	0	1	0	0	0	1	1	112
**269**	0	0	0	1	0	0	1	0	0	1	0	1	0	1	0	0	0	0	0	1	0	0	0	1	1	112
**270**	0	0	0	0	0	0	0	0	0	1	0	1	0	1	0	0	0	0	0	0	0	1	1	1	1	110
**271**	0	0	0	0	0	0	0	0	0	0	0	0	0	0	0	1	0	0	0	0	0	1	0	1	0	110
**272**	0	0	0	0	0	0	0	0	0	0	0	0	1	0	0	0	0	0	0	1	0	1	0	1	0	109
**273**	0	0	1	0	0	0	0	0	0	0	0	0	0	1	0	0	0	0	0	0	0	0	0	1	0	109
**274**	0	0	1	0	0	0	0	0	1	1	0	1	0	1	0	0	0	0	0	1	0	1	0	1	1	108
**275**	0	0	0	0	0	0	0	0	1	1	0	1	0	0	0	0	0	0	0	1	0	0	0	1	0	108
**276**	0	1	0	0	1	0	0	0	1	1	0	1	1	0	0	0	0	0	0	0	0	0	0	1	1	107
**277**	0	0	0	0	0	0	1	0	0	0	0	1	0	0	0	0	0	0	0	1	0	0	0	1	0	107
**278**	0	1	0	0	1	0	0	0	0	0	0	1	1	0	0	0	0	0	0	1	0	0	0	1	1	106
**279**	0	1	0	1	1	0	0	0	1	1	0	1	1	0	0	0	0	0	0	1	0	0	0	1	1	106
**280**	0	1	0	0	1	0	1	0	1	0	0	1	0	0	0	0	0	0	0	1	0	0	0	1	1	105
**281**	0	1	0	0	1	0	0	0	0	0	0	1	0	0	0	0	0	0	0	0	0	1	0	1	0	105
**282**	0	0	0	0	0	0	0	0	0	1	0	1	0	0	0	0	0	0	0	1	0	0	1	1	1	104
**283**	0	0	1	0	0	0	1	0	1	0	0	1	0	0	0	0	0	0	0	0	0	0	0	1	1	104
**284**	0	0	1	0	0	0	1	0	1	1	0	1	0	1	0	0	0	0	0	0	0	0	0	1	1	103
**285**	0	0	1	0	0	0	1	0	1	0	0	1	0	0	0	0	0	0	0	1	0	0	0	1	1	103
**286**	0	1	0	0	1	0	1	0	0	0	0	1	0	0	0	0	0	0	0	0	0	0	0	1	1	103
**287**	0	1	0	0	0	0	0	0	0	0	0	1	0	0	0	0	0	0	0	1	0	0	0	1	0	103
**288**	0	0	0	0	0	0	0	0	0	1	0	1	0	1	0	0	0	0	0	1	0	1	1	1	0	102
**289**	0	0	0	1	0	0	0	0	0	0	0	0	0	0	0	0	0	0	0	0	0	0	0	1	1	102
**290**	0	0	1	0	0	0	0	0	0	0	0	1	0	0	0	0	0	0	0	1	0	1	0	1	1	102
**291**	0	0	0	0	0	0	0	0	1	0	0	1	0	0	0	1	0	0	0	0	0	0	0	1	1	101
**292**	0	0	0	0	0	0	1	0	0	0	0	0	0	1	0	0	0	0	0	1	0	0	0	1	1	100
**293**	0	0	0	0	0	0	0	0	0	1	1	1	0	0	0	0	0	0	0	1	0	0	0	1	1	100

	CD2	CD3	CD4	CD7	CD8	CD10	CD13	CD18	CD26	CD29	CD36	CD44	CD45RA	CD49f	CD54	CD56	CD57	CD58	CD62L	CD71	CD80	Cytokeratin	Histone_H1	HLA-DQ	HLA-DR	Freq. (Voxel)
**294**	0	0	0	0	0	0	1	0	1	1	0	1	0	1	0	0	0	0	0	1	0	0	0	1	1	100
**295**	0	0	0	0	0	0	1	0	1	1	0	1	0	1	0	0	0	0	0	0	0	0	0	1	1	99
**296**	0	1	1	1	1	0	0	0	1	0	0	1	1	0	0	0	0	0	0	1	0	0	0	1	1	98
**297**	0	0	0	0	0	0	0	0	0	1	0	1	0	1	0	0	0	0	0	0	0	0	1	1	1	98
**298**	0	0	0	0	0	0	1	0	1	0	0	1	0	0	0	0	0	0	0	0	0	0	0	1	0	98
**299**	0	1	0	0	0	0	0	0	1	1	0	1	0	0	0	0	0	0	0	0	0	0	0	1	0	98
**300**	0	0	1	0	0	0	1	0	0	1	0	1	0	1	0	0	0	0	0	1	0	0	0	1	0	97
**301**	0	1	0	0	0	0	0	0	0	1	0	1	0	0	0	0	0	0	0	1	0	0	0	1	1	97
**302**	0	0	0	0	0	0	0	0	0	0	0	0	1	0	0	1	0	0	0	1	0	1	0	1	0	95
**303**	0	0	1	0	0	0	0	0	0	1	0	1	0	0	0	0	0	0	0	1	0	0	0	1	0	95
**304**	0	0	1	0	0	0	1	0	0	0	0	0	0	0	0	0	0	0	0	1	0	0	0	1	1	94
**305**	0	0	0	0	0	0	0	0	0	1	0	0	0	1	0	0	0	0	0	0	0	1	0	1	0	94
**306**	0	0	1	1	0	0	1	0	0	1	0	1	0	1	0	0	0	0	0	1	0	0	0	1	1	94
**307**	0	1	0	1	1	0	0	0	1	0	0	1	0	0	0	0	0	0	0	0	0	0	0	1	1	93
**308**	0	0	1	0	0	0	0	0	1	1	0	1	0	0	0	0	0	0	0	1	0	0	0	1	1	93
**309**	0	0	0	0	0	0	0	0	1	1	0	1	0	0	0	0	0	0	0	1	0	1	0	1	0	92
**310**	0	0	1	0	0	0	1	0	0	0	0	1	0	1	0	0	0	0	0	1	0	0	0	1	1	92
**311**	0	1	0	1	1	0	1	0	0	0	0	1	0	0	0	0	0	0	0	0	0	0	0	1	1	91
**312**	0	0	0	0	0	0	1	0	0	1	1	1	0	1	0	0	0	0	0	1	0	0	0	1	1	91
**313**	0	0	0	0	1	0	0	0	1	0	0	1	0	0	0	0	0	0	0	0	0	0	0	1	0	91
**314**	0	0	1	1	0	0	1	0	0	1	0	1	1	1	0	1	0	0	0	1	0	0	0	1	1	90
**315**	0	0	0	0	0	0	0	0	1	0	0	0	0	0	0	0	0	0	0	0	0	0	1	1	1	90
**316**	0	1	0	0	1	0	0	0	1	1	0	1	1	0	0	0	0	0	0	1	0	0	0	1	1	90
**317**	0	1	0	0	1	0	0	0	0	0	0	1	0	0	0	0	0	0	0	1	0	1	0	1	1	90
**318**	0	1	0	0	0	0	0	0	0	0	0	0	0	0	0	0	0	0	0	0	0	1	1	1	1	89
**319**	0	0	0	0	1	0	1	0	1	0	0	1	0	0	0	0	0	0	0	0	0	0	0	1	1	89
**320**	0	0	0	0	0	0	0	0	1	0	0	1	0	1	0	0	0	0	0	1	0	1	0	1	0	89
**321**	0	1	0	0	1	0	0	0	1	0	0	1	0	0	0	0	0	0	0	1	0	1	0	1	1	89
**322**	0	0	0	0	0	0	1	0	0	1	0	0	0	0	0	0	0	0	0	0	0	0	0	1	1	89
**323**	0	0	1	0	0	0	0	0	0	1	0	0	0	0	0	0	0	0	0	1	0	0	0	1	0	88
**324**	0	1	0	0	0	0	0	0	1	0	0	1	0	0	0	0	0	0	0	1	0	1	0	1	1	88
**325**	0	1	0	0	0	0	0	0	0	0	0	1	0	0	0	0	0	0	0	1	0	1	1	1	1	87
**326**	0	0	1	0	0	0	0	0	0	1	0	1	0	1	0	0	0	0	0	1	0	1	0	1	1	87
**327**	0	0	0	0	0	0	1	0	0	0	0	0	0	0	0	0	0	0	0	0	0	0	1	1	0	87
**328**	0	0	1	0	0	0	1	0	0	0	0	0	0	1	0	0	0	0	0	0	0	0	0	1	1	86
**329**	0	1	0	0	1	0	1	0	1	0	0	1	0	0	0	0	0	0	0	0	0	0	0	1	1	85
**330**	0	1	0	1	1	0	1	0	1	0	0	1	0	0	0	0	0	0	0	1	0	0	0	1	1	85
**331**	0	1	0	1	1	0	0	0	0	0	0	1	0	0	0	0	0	0	0	0	0	0	0	1	0	85
**332**	0	1	0	1	1	0	0	0	0	0	0	1	0	0	0	0	0	0	0	1	0	0	0	1	0	84
**333**	0	1	0	0	0	0	0	0	0	1	0	1	0	0	0	0	0	0	0	0	0	1	0	1	1	84
**334**	0	0	0	0	0	0	0	0	0	1	0	1	0	0	0	0	0	0	0	1	0	0	1	1	0	84
**335**	0	0	0	0	0	0	0	0	0	1	0	1	0	1	0	0	0	0	0	0	0	0	1	1	0	84
**336**	0	0	0	0	1	0	0	0	0	1	0	1	0	0	0	0	0	0	0	1	0	0	0	1	0	84
**337**	0	0	1	0	0	0	0	0	1	0	0	1	0	0	0	0	0	0	0	1	0	1	0	1	1	82
**338**	0	1	0	0	1	0	0	0	1	0	0	1	1	0	0	0	0	0	0	1	0	0	0	1	1	82
**339**	0	0	0	0	0	0	1	0	1	0	0	1	0	0	0	1	0	0	0	0	0	0	0	1	1	82
**340**	0	1	0	0	1	0	0	0	0	1	0	1	0	0	0	0	0	0	0	1	0	0	0	1	0	81
**341**	0	0	1	0	0	0	0	0	0	1	0	1	0	1	0	0	0	0	0	0	0	0	0	1	0	80
**342**	0	1	0	1	1	0	0	0	1	0	0	1	0	0	0	0	0	0	0	1	0	0	0	1	0	79
**343**	0	0	0	0	0	0	0	0	0	1	0	0	0	1	0	0	0	0	0	0	0	1	0	1	1	79
**344**	0	1	0	0	1	0	0	0	1	1	0	1	0	0	0	0	0	0	0	0	0	1	0	1	1	79
**345**	0	0	1	0	0	0	0	0	1	0	0	0	0	0	0	0	0	0	0	1	0	1	0	1	1	79
**346**	0	0	0	0	0	0	1	0	0	1	0	1	0	0	0	0	0	0	0	1	0	0	0	1	0	79
**347**	0	0	1	1	0	0	1	0	0	1	0	1	1	1	0	0	0	0	0	1	0	0	0	1	1	79
**348**	0	1	0	0	1	0	0	0	1	0	0	1	0	0	0	0	0	0	0	0	0	0	1	1	1	78
**349**	0	1	0	0	0	0	0	0	0	0	0	0	0	0	0	0	0	0	0	1	0	1	1	1	1	77
**350**	0	0	0	0	0	0	0	0	1	0	0	0	0	0	0	0	0	0	0	1	0	0	0	1	0	77
**351**	0	0	0	0	0	0	0	0	1	0	0	1	0	0	0	0	0	0	0	0	0	0	1	1	1	76
**352**	0	1	0	1	1	0	0	0	0	1	0	1	1	0	0	0	0	0	0	1	0	0	0	1	1	76
**353**	0	0	0	0	1	0	0	0	1	1	0	1	0	0	0	0	0	0	0	0	0	0	0	1	1	76
**354**	0	1	0	1	1	0	0	0	1	0	0	1	0	0	0	0	0	0	0	0	0	0	0	1	0	76
**355**	0	0	0	0	0	0	1	0	0	0	0	0	0	0	0	0	0	0	0	0	0	1	0	1	0	76
**356**	0	0	1	0	1	0	1	0	1	0	0	1	0	0	0	0	0	0	0	0	0	0	0	1	1	75
**357**	0	0	0	0	0	0	0	0	0	0	0	1	0	0	0	1	0	0	0	0	0	0	1	1	1	75
**358**	0	1	0	1	1	0	1	0	1	0	0	1	0	0	0	0	0	0	0	0	0	0	0	1	1	73
**359**	0	1	0	0	0	0	0	0	0	0	0	1	1	0	0	0	0	0	0	0	0	0	0	1	1	73
**360**	0	0	1	0	0	0	0	0	1	1	0	1	0	0	0	0	0	0	0	0	0	0	0	1	1	73
**361**	0	1	0	1	1	0	1	0	0	1	0	1	0	0	0	0	0	0	0	1	0	0	0	1	1	72
**362**	0	0	1	0	0	0	0	0	1	0	0	0	0	0	0	0	0	0	0	0	0	0	0	1	0	72
**363**	0	0	1	0	0	0	1	0	1	0	0	1	0	1	0	0	0	0	0	1	0	0	0	1	1	71
**364**	0	0	1	0	0	0	0	0	0	1	0	0	0	1	0	0	0	0	0	0	0	0	0	1	1	71
**365**	0	0	0	0	0	0	1	0	0	1	0	1	0	1	0	0	0	0	0	1	0	1	0	1	0	71
**366**	0	0	0	0	0	0	1	0	0	0	0	0	0	0	0	1	0	0	0	1	0	0	0	1	1	69
**367**	0	0	1	0	0	0	0	0	0	0	0	0	1	0	0	1	0	0	0	0	0	1	0	1	0	69
**368**	0	0	0	0	0	0	1	0	0	0	0	0	1	0	0	1	0	0	0	0	0	0	0	1	0	69
**369**	0	0	0	1	0	0	1	0	0	0	0	1	0	0	0	0	0	0	0	0	0	0	0	1	1	69
**370**	0	0	0	1	0	0	1	0	0	1	0	1	0	1	0	0	0	0	0	0	0	0	0	1	1	69
**371**	0	0	0	0	0	0	0	0	0	0	0	0	0	0	0	0	0	0	0	1	0	1	1	1	1	69
**372**	0	1	0	1	1	0	0	0	0	1	0	1	0	0	0	0	0	0	0	1	0	0	0	1	0	69
**373**	0	1	0	0	1	0	0	0	0	1	0	1	0	0	0	0	0	0	0	0	0	0	1	1	1	68
**374**	0	0	1	0	0	0	0	0	0	1	0	1	0	1	0	0	0	0	0	1	0	1	0	1	0	68
**375**	0	0	0	0	0	0	0	0	0	0	1	0	0	0	0	0	0	0	0	0	0	1	1	1	1	67
**376**	0	0	1	0	0	0	0	0	1	0	0	1	0	0	0	0	0	0	0	0	0	1	0	1	0	67
**377**	0	0	0	0	0	0	1	0	0	1	0	1	0	1	0	0	0	0	0	0	0	1	0	1	0	67
**378**	0	1	0	0	1	0	0	0	0	0	0	1	0	0	0	0	0	0	0	1	0	0	0	1	0	67
**379**	0	0	0	0	0	0	1	0	0	0	0	1	0	0	0	1	0	0	0	1	0	0	0	1	1	66
**380**	0	0	1	0	0	0	0	0	0	1	0	1	0	0	0	0	0	0	0	0	0	1	0	1	1	65
**381**	0	0	0	0	0	0	1	0	0	1	0	1	0	1	0	0	0	0	0	0	0	1	0	1	1	65
**382**	0	0	0	1	1	0	0	0	0	1	0	1	0	0	0	0	0	0	0	0	0	0	0	1	1	65
**383**	0	0	1	0	0	0	1	0	0	0	0	0	0	0	0	0	0	0	0	0	0	0	0	1	0	65
**384**	0	1	0	1	0	0	0	0	0	1	0	1	0	0	0	0	0	0	0	0	0	0	0	1	0	64
**385**	0	0	0	1	0	0	0	0	0	0	0	0	0	1	0	0	0	0	0	0	0	0	0	1	1	64
**386**	0	0	1	0	0	0	0	0	1	1	0	1	0	0	0	0	0	0	0	1	0	1	0	1	1	64
**387**	0	0	0	0	0	0	1	0	1	0	0	0	0	0	0	0	0	0	0	1	0	0	0	1	1	63
**388**	0	1	0	1	0	0	0	0	0	1	0	1	0	0	0	0	0	0	0	0	0	0	0	1	1	63
**389**	0	1	0	0	1	0	0	0	1	0	0	1	0	0	0	0	0	0	0	0	0	0	1	1	0	63
**390**	0	0	0	0	0	0	0	0	0	0	0	0	1	0	0	0	0	0	0	0	0	0	1	1	0	62
**391**	0	0	0	0	0	0	1	0	0	1	0	1	0	1	0	0	0	0	0	1	0	1	1	1	0	62

	CD2	CD3	CD4	CD7	CD8	CD10	CD13	CD18	CD26	CD29	CD36	CD44	CD45RA	CD49f	CD54	CD56	CD57	CD58	CD62L	CD71	CD80	Cytokeratin	Histone_H1	HLA-DQ	HLA-DR	Freq. (Voxel)
**392**	0	0	0	0	1	0	1	0	1	0	0	1	0	0	0	0	0	0	0	1	0	0	0	1	1	62
**393**	0	1	0	0	1	0	0	0	1	1	0	1	0	0	0	0	0	0	0	0	0	0	1	1	1	62
**394**	0	0	0	1	0	0	0	0	0	1	0	1	0	0	0	0	0	0	0	0	0	0	0	1	0	62
**395**	0	0	1	0	0	0	0	0	0	0	0	0	0	0	0	0	0	0	0	1	0	1	0	1	1	62
**396**	0	0	0	0	0	0	0	0	1	1	0	1	0	1	0	0	0	0	0	0	0	1	0	1	1	61
**397**	0	0	0	0	0	0	0	0	0	1	0	1	0	0	0	0	0	0	0	0	0	1	1	1	0	61
**398**	0	0	0	0	0	0	0	0	1	0	0	1	0	1	0	0	0	0	0	0	0	1	0	1	0	61
**399**	0	1	0	0	1	0	0	0	0	1	0	1	0	0	0	0	0	0	0	0	0	1	0	1	1	61
**400**	0	0	0	0	0	0	0	0	0	1	0	1	0	1	0	0	0	0	0	1	0	1	1	1	1	61
**401**	0	0	1	0	0	0	0	0	1	0	0	0	0	0	0	0	0	0	0	0	0	1	0	1	1	61
**402**	0	0	1	0	0	0	0	0	0	0	0	0	0	1	0	0	0	0	0	1	0	0	0	1	1	60
**403**	0	0	0	0	0	0	0	0	0	0	0	0	0	1	0	0	0	0	0	1	0	1	0	1	0	60
**404**	0	0	0	0	1	0	1	0	0	0	0	1	0	0	0	0	0	0	0	0	0	0	0	1	1	60
**405**	0	0	1	0	0	0	1	0	0	1	0	1	1	1	0	0	0	0	0	1	0	0	0	1	1	60
**406**	0	0	1	0	0	0	0	0	0	1	0	1	0	0	0	0	0	0	0	1	0	1	0	1	1	60
**407**	0	1	0	0	1	0	1	0	0	1	0	1	0	0	0	0	0	0	0	0	0	0	0	1	1	59
**408**	0	0	1	0	0	0	0	0	0	1	0	0	0	1	0	0	0	0	0	1	0	0	0	1	0	59
**409**	0	0	1	0	0	0	0	0	0	1	0	1	0	1	0	0	0	0	0	1	0	0	0	1	0	59
**410**	0	0	0	0	0	0	1	0	1	1	0	1	0	0	0	0	0	0	0	1	0	0	0	1	1	59
**411**	0	1	1	0	1	0	0	0	0	1	0	1	0	0	0	0	0	0	0	1	0	0	0	1	1	58
**412**	0	0	0	0	0	0	0	0	1	0	0	0	0	1	0	0	0	0	0	0	0	1	0	1	0	58
**413**	0	1	0	0	0	0	0	0	0	0	0	1	0	0	0	0	0	0	0	1	0	0	1	1	0	58
**414**	0	0	0	0	1	0	0	0	1	0	0	1	0	0	0	0	0	0	0	1	0	0	0	1	1	58
**415**	0	1	0	0	1	0	0	0	0	0	0	1	0	0	0	0	0	0	0	1	0	1	1	1	1	57
**416**	0	0	1	0	0	0	0	0	0	1	0	1	0	1	0	0	0	0	0	0	0	1	0	1	0	57
**417**	0	0	1	0	0	0	0	0	0	0	0	1	0	0	0	0	0	0	0	1	0	0	0	1	0	57
**418**	0	0	1	0	0	0	0	0	0	0	0	0	0	0	0	0	0	0	0	1	0	1	0	1	0	57
**419**	0	0	0	0	0	0	0	0	0	1	0	0	1	0	0	0	0	0	0	0	0	0	0	1	0	56
**420**	0	1	0	0	0	0	0	0	0	0	0	0	0	0	0	0	0	0	0	0	0	1	0	1	1	56
**421**	0	0	1	0	0	0	1	0	1	1	0	1	0	0	0	0	0	0	0	1	0	0	0	1	1	56
**422**	0	1	0	1	1	0	0	0	0	0	0	1	0	0	0	0	0	0	0	1	0	1	0	1	1	56
**423**	0	0	0	0	1	0	0	0	1	1	0	1	0	0	0	0	0	0	0	1	0	0	0	1	1	56
**424**	0	0	0	1	1	0	0	0	0	1	0	1	0	0	0	0	0	0	0	1	0	0	0	1	1	56
**425**	0	1	0	0	0	0	1	0	0	0	0	1	0	0	0	0	0	0	0	0	0	0	0	1	1	56
**426**	0	0	0	0	0	0	0	0	0	0	0	0	0	1	0	0	0	0	0	0	0	1	1	1	0	56
**427**	0	0	0	1	0	0	0	0	0	0	0	1	0	0	0	0	0	0	0	0	0	0	0	1	0	56
**428**	0	0	1	0	0	0	0	0	0	0	0	0	1	0	0	0	0	0	0	1	0	0	0	1	0	56
**429**	0	0	0	0	1	0	1	0	0	1	0	1	0	1	0	0	0	0	0	1	0	1	0	1	1	55
**430**	0	0	0	0	1	0	0	0	0	0	0	1	1	0	0	0	0	0	0	0	0	1	0	1	1	55
**431**	0	1	0	0	1	0	0	0	0	1	0	1	1	0	0	0	0	0	0	0	0	0	0	1	0	55
**432**	0	1	0	0	1	0	0	0	0	0	0	1	1	1	0	0	0	0	0	0	0	0	0	1	1	54
**433**	0	1	0	1	1	0	0	0	1	1	0	1	1	0	0	0	0	0	0	1	0	0	0	1	0	54
**434**	0	1	0	0	0	0	0	0	0	0	0	0	0	0	0	0	0	0	0	0	0	1	1	1	0	54
**435**	0	0	0	0	0	0	1	0	0	1	0	0	0	0	0	0	0	0	0	0	0	0	0	1	0	54
**436**	0	0	0	0	0	0	1	0	0	0	0	0	0	0	0	1	0	0	0	0	0	0	0	1	0	54
**437**	0	0	0	0	0	0	0	0	0	0	0	1	0	1	0	0	0	0	0	1	0	1	0	1	0	54
**438**	0	1	0	0	1	0	0	0	1	0	0	1	0	0	0	0	0	0	0	1	0	0	0	1	0	54
**439**	0	0	0	0	0	0	0	0	1	1	0	1	0	1	0	0	0	0	0	1	0	1	0	1	0	54
**440**	0	0	0	0	1	0	0	0	0	0	0	1	0	0	0	0	0	0	0	0	0	0	1	1	0	54
**441**	0	0	0	0	0	0	0	0	0	0	0	1	0	1	0	0	0	0	0	0	0	0	1	1	0	54
**442**	0	0	1	0	0	0	0	0	0	1	0	0	0	0	0	0	0	0	0	1	0	0	0	1	1	53
**443**	0	0	0	0	0	0	0	0	0	1	0	0	0	1	0	0	0	0	0	0	0	0	1	1	1	53
**444**	0	1	0	1	0	0	0	0	1	1	0	1	0	0	0	0	0	0	0	0	0	0	0	1	1	53
**445**	0	0	0	0	1	0	1	0	0	1	0	1	0	0	0	0	0	0	0	1	0	0	0	1	1	52
**446**	0	0	0	0	0	0	1	0	0	0	0	1	0	0	0	1	0	0	0	0	0	0	0	1	0	52
**447**	0	0	1	0	0	0	0	0	0	0	1	0	0	0	0	0	0	0	0	0	0	0	0	1	0	52
**448**	0	1	1	1	1	0	0	0	1	1	0	1	1	0	0	0	0	0	0	1	0	0	0	1	1	52
**449**	0	1	0	0	1	0	1	0	0	0	0	1	0	0	0	0	0	0	0	1	0	0	0	1	1	52
**450**	0	0	0	0	1	0	0	0	0	0	0	1	0	0	0	0	0	0	0	1	0	1	0	1	1	52
**451**	0	0	0	0	1	0	0	0	1	0	0	1	0	0	0	0	0	0	0	1	0	1	0	1	1	52
**452**	0	1	0	1	1	0	0	0	1	1	0	1	0	0	0	0	0	0	0	1	0	0	0	1	0	52
**453**	0	0	0	0	0	0	1	0	0	1	0	1	0	0	0	0	0	0	0	0	0	0	1	1	1	51
**454**	0	1	0	0	1	0	0	0	0	0	0	1	0	1	0	0	0	0	0	0	0	0	0	1	1	51
**455**	0	0	1	0	0	0	0	0	0	0	0	0	1	0	0	0	0	0	0	0	0	1	0	1	0	51
**456**	0	0	0	0	0	0	1	0	1	1	0	1	0	1	0	0	0	0	0	1	0	1	0	1	0	51
**457**	0	0	1	1	0	0	1	0	1	1	0	1	0	1	0	0	0	0	0	1	0	0	0	1	1	51
**458**	0	0	0	0	0	0	1	0	1	1	0	1	0	1	0	0	0	0	0	1	0	1	0	1	1	51
**459**	0	0	0	1	0	0	0	0	0	0	0	0	0	0	0	0	0	0	0	0	0	0	0	1	0	51
**460**	0	0	0	0	0	0	0	0	0	0	0	0	0	1	0	0	0	0	0	1	0	0	0	1	0	50
**461**	0	0	0	1	1	0	0	0	0	0	0	1	0	0	0	0	0	0	0	1	0	0	0	1	1	50
**462**	0	0	0	0	0	0	1	0	0	0	0	1	0	1	0	0	0	0	0	0	0	0	1	1	1	50
**463**	0	0	0	0	0	0	1	0	0	1	0	0	0	0	0	0	0	0	0	1	0	0	0	1	1	50
**464**	0	0	0	0	0	0	0	0	0	0	0	1	0	1	0	0	0	0	0	1	0	0	0	1	0	50
**465**	0	0	0	0	0	0	0	0	0	0	0	1	1	0	0	0	0	0	0	0	0	0	0	1	1	50
**466**	0	0	1	0	0	0	0	0	0	1	0	1	0	0	0	0	0	0	0	0	0	1	0	1	0	50
**467**	0	0	1	0	0	0	1	0	0	1	1	1	0	1	0	0	0	0	0	0	0	0	0	1	1	50
**468**	0	0	0	0	0	0	1	0	1	0	0	1	0	0	0	0	0	0	0	0	0	1	0	1	1	50
**469**	0	1	0	0	0	0	0	0	1	0	0	1	0	0	0	0	0	0	0	0	0	0	1	1	0	50
**470**	0	1	0	0	1	0	0	0	0	1	0	1	0	0	0	0	0	0	0	1	0	0	1	1	1	49
**471**	0	1	0	0	0	0	0	0	0	0	1	1	0	0	0	0	0	0	0	0	0	1	0	1	1	49
**472**	0	1	0	0	0	0	1	0	0	1	0	1	0	0	0	0	0	0	0	0	0	0	0	1	1	49
**473**	0	0	1	0	0	0	0	0	1	0	0	0	0	0	0	0	0	0	0	0	0	1	0	1	0	49
**474**	0	1	0	1	0	0	0	0	1	0	0	1	0	0	0	0	0	0	0	1	0	1	0	1	1	49
**475**	0	0	0	0	0	0	1	0	0	1	0	1	0	0	0	0	0	0	0	0	0	1	0	1	0	49
**476**	0	0	0	0	0	0	1	0	1	0	0	1	0	0	0	0	0	0	0	1	0	1	0	1	1	49
**477**	0	0	0	0	0	0	0	0	1	0	0	0	0	1	0	0	0	0	0	0	0	1	0	1	1	49
**478**	0	0	1	0	0	0	0	0	1	0	0	1	0	1	0	0	0	0	0	1	0	1	0	1	1	49
**479**	0	0	0	0	0	0	0	0	1	0	0	0	1	0	0	0	0	0	0	1	0	0	0	1	0	48
**480**	0	0	0	0	1	0	1	0	0	1	0	1	0	0	0	0	0	0	0	0	0	0	0	1	1	48
**481**	0	1	0	0	1	0	0	0	0	1	0	1	1	0	0	0	0	0	0	1	0	0	0	1	0	48
**482**	0	0	0	0	0	0	0	0	1	0	0	0	0	0	0	0	0	0	0	0	0	0	1	1	0	48
**483**	0	0	0	0	0	0	1	0	0	1	0	0	0	1	0	0	0	0	0	1	0	0	0	1	1	48
**484**	0	0	0	0	1	0	0	0	0	0	0	0	0	0	0	0	0	0	0	0	0	0	0	1	0	48
**485**	0	1	0	1	1	0	0	0	1	1	0	1	0	0	0	0	0	0	0	0	0	0	0	1	0	48
**486**	0	0	0	0	0	0	0	0	0	0	1	0	0	0	0	0	0	0	0	0	0	0	1	1	0	47
**487**	0	1	0	0	1	0	0	0	0	1	0	1	1	0	0	0	0	0	0	0	0	1	0	1	1	47
**488**	0	0	0	0	0	0	0	0	0	0	0	1	0	0	0	1	0	0	0	0	0	0	1	1	0	47
**489**	0	0	0	0	0	0	0	0	0	0	0	1	0	1	0	0	0	0	0	0	0	0	1	1	1	47

	CD2	CD3	CD4	CD7	CD8	CD10	CD13	CD18	CD26	CD29	CD36	CD44	CD45RA	CD49f	CD54	CD56	CD57	CD58	CD62L	CD71	CD80	Cytokeratin	Histone_H1	HLA-DQ	HLA-DR	Freq. (Voxel)
**490**	0	0	0	0	0	0	0	0	0	1	1	0	0	0	0	0	0	0	0	0	0	0	0	1	1	47
**491**	0	0	0	0	1	0	0	0	0	0	0	1	0	0	0	0	0	0	0	0	0	0	1	1	1	47
**492**	0	0	0	0	0	0	1	0	0	1	0	1	0	1	0	0	0	0	0	0	0	1	1	1	0	46
**493**	0	0	1	0	0	0	1	0	0	1	0	1	0	1	0	0	0	0	0	1	0	1	1	1	1	46
**494**	0	0	1	0	0	0	1	0	1	0	0	1	0	1	0	0	0	0	0	1	0	1	0	1	1	46
**495**	0	0	0	0	1	0	0	0	0	1	0	1	0	0	0	0	0	0	0	0	0	0	1	1	0	46
**496**	0	0	1	0	0	0	0	0	0	0	0	0	1	0	0	1	0	0	0	1	0	0	0	1	0	46
**497**	0	0	1	0	0	0	0	0	0	0	0	1	0	1	0	0	0	0	0	1	0	0	0	1	1	46
**498**	0	0	0	0	1	0	0	0	0	0	0	1	0	0	0	0	0	0	0	0	0	1	1	1	1	46
**499**	0	0	0	0	1	0	0	0	0	0	0	1	0	0	0	0	0	0	0	0	0	1	0	1	0	46
**500**	0	1	0	1	1	0	0	0	0	0	0	0	1	0	0	0	0	0	0	1	0	0	0	1	1	45
**501**	0	1	0	0	1	0	1	0	0	1	0	1	0	1	0	0	0	0	0	0	0	1	0	1	1	45
**502**	0	0	0	1	0	0	1	0	0	1	0	1	1	1	0	0	0	0	0	1	0	0	0	1	1	45
**503**	0	0	1	0	0	0	0	0	0	0	0	1	0	0	0	0	0	0	0	0	0	0	1	1	1	45
**504**	0	1	1	1	1	0	0	0	0	1	0	1	0	0	0	0	0	0	0	1	0	0	0	1	1	45
**505**	0	1	1	0	1	0	0	0	1	1	0	1	1	0	0	0	0	0	0	1	0	0	0	1	1	45
**506**	0	0	1	0	0	0	0	0	0	1	0	1	0	1	0	0	0	0	0	0	0	1	0	1	1	45
**507**	0	0	0	1	0	0	0	0	0	1	0	1	0	0	0	0	0	0	0	1	0	0	0	1	1	45
**508**	0	1	0	1	0	0	0	0	0	0	0	1	0	0	0	0	0	0	0	1	0	0	0	1	0	45
**509**	0	0	0	0	0	0	1	0	0	0	0	0	0	0	0	0	0	0	0	1	0	0	1	1	1	44
**510**	0	1	0	1	1	0	0	0	1	0	0	1	1	0	0	0	0	0	0	0	0	0	0	1	1	44
**511**	0	1	0	0	1	0	0	0	0	0	0	1	0	0	0	0	0	0	0	1	0	0	1	1	0	44
**512**	0	0	0	0	0	0	1	0	0	0	0	0	0	0	0	0	0	0	0	1	0	0	0	1	0	44
**513**	0	1	0	1	1	0	0	0	0	0	0	1	0	0	0	0	0	0	0	1	0	0	1	1	1	43
**514**	0	1	0	0	1	0	0	0	0	1	0	1	0	1	0	0	0	0	0	0	0	1	1	1	1	43
**515**	0	0	1	0	0	0	1	0	0	1	0	1	0	1	0	0	0	0	0	1	0	1	0	1	0	43
**516**	0	1	0	0	1	0	0	0	0	1	0	1	0	0	0	0	0	0	0	0	0	0	1	1	0	43
**517**	0	0	1	0	0	0	0	0	0	0	1	0	0	0	0	0	0	0	0	0	0	0	0	1	1	43
**518**	0	0	0	0	0	0	0	0	0	1	0	0	0	1	0	0	0	0	0	1	0	0	0	1	1	43
**519**	0	0	1	0	1	0	1	0	1	1	0	1	0	0	0	0	0	0	0	0	0	0	0	1	1	42
**520**	0	0	0	1	0	0	1	0	1	1	0	1	0	1	0	0	0	0	0	1	0	0	0	1	1	42
**521**	0	1	0	1	1	0	0	0	1	1	0	1	0	1	0	0	0	0	0	1	0	1	0	1	1	42
**522**	0	1	0	1	1	0	0	0	1	1	0	1	1	0	0	0	0	0	0	0	0	0	0	1	0	42
**523**	0	0	0	1	1	0	0	0	0	0	0	1	1	0	0	0	0	0	0	0	0	0	0	1	1	42
**524**	0	0	0	0	0	0	1	0	0	1	0	1	0	1	0	0	0	0	0	1	0	0	1	1	1	42
**525**	0	0	1	0	0	0	0	0	1	0	0	0	0	0	0	0	0	0	0	1	0	0	0	1	1	42
**526**	0	0	0	0	0	0	0	0	0	1	1	1	0	1	0	0	0	0	0	0	0	0	0	1	0	42
**527**	0	1	0	0	0	0	0	0	1	1	0	1	0	0	0	0	0	0	0	0	0	1	0	1	1	42
**528**	0	1	0	0	0	0	0	0	1	0	0	1	0	0	0	0	0	0	0	1	0	0	0	1	1	42
**529**	0	1	0	0	1	0	0	0	0	0	0	1	1	0	0	0	0	0	0	0	0	0	0	1	0	42
**530**	0	0	1	0	0	0	1	0	1	1	0	1	0	0	0	0	0	0	0	0	0	0	0	1	1	41
**531**	0	1	0	0	1	0	0	0	0	0	0	0	0	0	0	0	0	0	0	0	0	0	0	1	1	41
**532**	0	0	0	0	1	0	1	0	0	1	0	1	0	1	0	0	0	0	0	0	0	1	0	1	1	41
**533**	0	0	0	0	0	0	0	0	1	1	0	1	0	1	0	0	0	0	0	1	0	0	0	1	1	41
**534**	0	0	0	1	0	0	0	0	0	0	0	1	0	0	0	0	0	0	0	1	0	0	0	1	1	41
**535**	0	0	0	0	1	0	0	0	0	1	0	1	0	1	0	0	0	0	0	0	0	1	1	1	1	41
**536**	0	0	0	0	0	0	0	0	0	1	0	0	0	1	0	0	0	0	0	1	0	0	0	1	0	41
**537**	0	0	0	0	0	0	1	0	0	1	0	0	1	1	0	0	0	0	0	1	0	0	0	1	1	41
**538**	0	1	0	0	1	0	0	0	0	0	0	1	0	0	0	0	0	0	0	1	0	0	1	1	1	41
**539**	0	1	0	1	0	0	0	0	0	1	0	1	0	0	0	0	0	0	0	1	0	0	0	1	1	41
**540**	0	1	0	1	1	0	0	0	0	1	0	1	0	1	0	0	0	0	0	1	0	0	0	1	1	40
**541**	0	0	0	0	0	0	1	0	0	0	0	0	0	0	0	1	0	0	0	0	0	0	1	1	1	40
**542**	0	0	1	0	0	0	0	0	0	0	0	0	1	0	0	0	0	0	0	0	0	0	1	1	0	40
**543**	0	0	1	0	0	0	0	0	0	0	0	0	1	0	0	0	0	0	0	1	0	1	0	1	0	40
**544**	0	0	0	1	0	0	0	0	0	0	0	1	0	1	0	0	0	0	0	0	0	0	0	1	1	40
**545**	0	0	0	0	0	0	1	0	0	1	0	1	0	0	0	0	0	0	0	0	0	1	0	1	1	40
**546**	0	0	0	0	0	0	0	0	0	0	1	1	0	0	0	0	0	0	0	0	0	1	1	1	0	40
**547**	0	1	0	1	1	0	0	0	0	1	0	1	0	0	0	0	0	0	0	1	0	1	0	1	1	40
**548**	0	0	0	0	0	0	0	0	1	1	0	1	0	1	0	0	0	0	0	0	0	1	0	1	0	40
**549**	0	0	0	0	0	0	1	0	0	1	1	1	0	0	0	0	0	0	0	1	0	0	0	1	1	39
**550**	0	1	0	0	0	0	0	0	0	1	0	1	0	0	0	0	0	0	0	0	0	0	1	1	0	39
**551**	0	1	0	0	1	0	0	0	1	1	0	1	1	0	0	0	0	0	0	0	0	1	0	1	1	39
**552**	0	0	1	0	0	0	1	0	1	0	0	1	0	1	0	0	0	0	0	0	0	0	0	1	1	39
**553**	0	0	0	0	0	0	1	0	1	0	0	0	0	0	0	1	0	0	0	0	0	0	0	1	1	39
**554**	0	0	0	0	0	0	0	0	0	1	1	1	0	1	0	0	0	0	0	1	0	0	0	1	1	39
**555**	0	0	0	0	0	0	1	0	0	1	0	1	0	0	0	0	0	0	0	1	0	0	1	1	1	38
**556**	0	1	0	0	1	0	0	0	0	0	0	1	1	0	0	0	0	0	0	1	0	1	0	1	1	38
**557**	0	1	0	1	1	0	0	0	0	0	0	1	1	0	0	0	0	0	0	1	0	0	0	1	0	38
**558**	0	0	0	1	0	0	0	0	0	0	0	0	0	1	0	0	0	0	0	0	0	0	0	1	0	38
**559**	0	0	0	0	0	0	1	0	1	0	0	0	0	1	0	0	0	0	0	0	0	0	0	1	1	38
**560**	0	1	0	0	1	0	0	0	0	1	0	1	0	1	0	0	0	0	0	0	0	0	1	1	1	38
**561**	0	0	1	0	0	0	1	0	0	0	0	0	0	1	0	0	0	0	0	1	0	0	0	1	1	38
**562**	0	0	0	0	0	0	1	0	0	0	0	1	0	0	0	1	0	0	0	0	0	0	1	1	1	38
**563**	0	0	1	0	0	0	0	0	0	0	0	0	0	1	0	0	0	0	0	0	0	1	0	1	1	38
**564**	0	0	1	0	0	0	0	0	1	1	0	1	0	0	0	0	0	0	0	0	0	1	0	1	1	38
**565**	0	0	0	0	0	0	1	0	0	0	0	0	1	0	0	0	0	0	0	1	0	0	0	1	0	38
**566**	0	0	0	0	0	0	1	0	0	0	0	1	0	0	0	0	0	0	0	1	0	1	0	1	1	38
**567**	0	0	0	0	0	0	1	0	0	0	0	0	0	1	0	0	0	0	0	0	0	1	0	1	1	38
**568**	0	1	0	1	1	0	0	0	0	1	0	1	1	0	0	0	0	0	0	0	0	0	0	1	1	38
**569**	0	1	0	0	1	0	0	0	1	0	0	1	0	0	0	0	0	0	0	0	0	1	0	1	0	38
**570**	0	0	1	0	0	0	0	0	0	0	0	0	0	0	0	1	0	0	0	0	0	1	0	1	0	38
**571**	0	1	1	0	1	0	1	0	1	1	0	1	0	0	0	0	0	0	0	1	0	0	0	1	1	37
**572**	0	0	1	0	0	0	0	0	0	0	0	0	0	0	0	1	0	0	0	0	0	0	0	1	1	37
**573**	0	1	0	1	0	0	0	0	1	0	0	1	0	0	0	0	0	0	0	1	0	0	0	1	1	37
**574**	0	0	0	0	0	0	1	0	1	0	0	0	0	0	0	0	0	0	0	0	0	0	0	1	0	37
**575**	0	0	0	0	0	0	0	0	1	0	0	1	0	0	0	0	0	0	0	0	0	0	1	1	0	37
**576**	0	0	0	0	0	0	0	0	0	1	0	0	0	0	0	0	0	0	0	1	0	0	1	1	0	37
**577**	0	0	1	0	0	0	1	0	0	0	0	1	0	0	0	1	0	0	0	1	0	0	0	1	1	36
**578**	0	0	1	0	0	0	0	0	0	0	0	0	1	0	0	1	0	0	0	1	0	1	0	1	0	36
**579**	0	1	0	0	1	0	0	0	1	1	0	1	1	1	0	0	0	0	0	0	0	0	0	1	1	36
**580**	0	1	0	0	0	0	1	0	0	1	0	1	0	0	0	0	0	0	0	1	0	0	0	1	1	36
**581**	0	1	0	0	0	0	0	0	0	0	1	1	0	0	0	0	0	0	0	1	0	1	0	1	1	36
**582**	0	0	0	0	0	0	0	0	0	0	1	1	0	0	0	0	0	0	0	0	0	1	1	1	1	36
**583**	0	0	0	0	1	0	0	0	0	1	0	1	1	0	0	0	0	0	0	0	0	0	0	1	1	36
**584**	0	0	1	0	0	0	0	0	0	0	0	0	0	1	0	0	0	0	0	0	0	1	0	1	0	36
**585**	0	1	0	0	0	0	0	0	1	0	0	1	0	0	0	0	0	0	0	1	0	0	0	1	0	36
**586**	0	0	0	0	1	0	0	0	0	1	0	1	0	0	0	0	0	0	0	0	0	1	0	1	0	36
**587**	0	1	0	1	0	0	0	0	0	0	0	1	0	0	0	0	0	0	0	1	0	0	0	1	1	36

	CD2	CD3	CD4	CD7	CD8	CD10	CD13	CD18	CD26	CD29	CD36	CD44	CD45RA	CD49f	CD54	CD56	CD57	CD58	CD62L	CD71	CD80	Cytokeratin	Histone_H1	HLA-DQ	HLA-DR	Freq. (Voxel)
**588**	0	0	0	0	0	0	1	0	0	1	0	1	0	1	0	0	0	0	0	1	0	1	1	1	1	36
**589**	0	0	0	0	1	0	0	0	0	1	0	1	0	0	0	0	0	0	0	1	0	1	0	1	1	36
**590**	0	1	0	0	1	0	0	0	0	1	0	1	0	1	0	0	0	0	0	0	0	0	0	1	1	36
**591**	0	1	0	1	1	0	0	0	1	1	0	1	1	0	0	0	0	0	0	0	0	0	0	1	1	36
**592**	0	0	1	1	0	0	1	0	0	1	0	1	0	1	0	0	0	0	0	0	0	0	0	1	1	36
**593**	0	0	0	0	0	0	1	0	0	0	0	1	0	0	0	0	0	0	0	0	0	0	1	1	1	36
**594**	0	0	0	0	0	0	0	0	0	0	0	1	0	0	0	0	0	0	0	1	0	0	1	1	0	36
**595**	0	1	1	1	1	0	0	0	1	1	0	1	1	1	0	0	0	0	0	1	0	0	0	1	1	35
**596**	0	1	0	0	0	0	0	0	1	0	0	1	0	0	0	0	0	0	0	1	0	1	1	1	1	35
**597**	0	0	0	0	1	0	0	0	0	0	0	1	1	0	0	0	0	0	0	0	0	0	0	1	0	35
**598**	0	1	0	0	1	0	0	0	1	1	0	1	1	0	0	0	0	0	0	1	0	0	0	1	0	35
**599**	0	0	0	0	0	0	0	0	0	1	0	1	0	0	0	0	0	0	0	0	0	1	1	1	1	35
**600**	0	1	0	1	0	0	0	0	0	0	0	1	0	0	0	0	0	0	0	0	0	0	0	1	1	35
**601**	0	0	1	0	0	0	1	0	0	1	0	0	0	0	0	0	0	0	0	0	0	0	0	1	1	35
**602**	0	1	0	1	1	0	0	0	1	1	0	1	0	0	0	0	0	0	0	1	0	1	0	1	1	35
**603**	0	0	0	1	0	0	0	0	0	1	0	1	0	0	0	0	0	0	0	1	0	0	0	1	0	35
**604**	0	0	1	0	0	0	1	0	0	0	0	0	1	0	0	0	0	0	0	0	0	0	0	1	0	34
**605**	0	0	0	0	0	0	0	0	0	0	0	1	0	0	0	0	0	0	0	1	0	1	1	1	0	34
**606**	0	0	0	1	0	0	0	0	0	0	0	0	1	0	0	0	0	0	0	1	0	0	0	1	0	34
**607**	0	1	0	0	0	0	0	0	0	0	0	1	0	1	0	0	0	0	0	0	0	1	0	1	1	34
**608**	0	0	0	0	0	0	0	0	0	1	1	0	0	0	0	0	0	0	0	0	0	0	0	1	0	34
**609**	0	1	0	1	0	0	0	0	1	0	0	1	0	0	0	0	0	0	0	0	0	0	0	1	1	34
**610**	0	0	1	0	0	0	1	0	0	1	1	1	0	1	0	0	0	0	0	1	0	0	0	1	1	34
**611**	0	0	0	0	0	0	0	0	0	1	0	1	0	1	0	0	0	0	0	0	0	1	1	1	0	34
**612**	0	0	1	0	0	0	0	0	0	1	0	0	0	1	0	0	0	0	0	1	0	0	0	1	1	34
**613**	0	1	0	1	0	0	0	0	0	0	0	1	0	0	0	0	0	0	0	0	0	0	0	1	0	34
**614**	0	0	0	0	1	0	0	0	1	1	0	1	0	0	0	0	0	0	0	0	0	0	0	1	0	34
**615**	0	0	0	0	0	0	1	0	0	0	0	0	1	0	0	0	0	0	0	1	0	1	0	1	0	34
**616**	0	0	0	0	0	0	0	0	1	0	0	1	0	0	0	0	0	0	0	1	0	0	0	1	0	34
**617**	0	1	0	0	1	0	0	0	1	0	0	1	1	0	0	0	0	0	0	1	0	1	0	1	1	33
**618**	0	1	1	1	1	0	0	0	0	1	0	1	1	0	0	0	0	0	0	1	0	0	0	1	1	33
**619**	0	1	0	1	1	0	1	0	1	0	0	1	0	0	0	0	0	0	0	0	0	0	1	1	1	33
**620**	0	0	1	0	0	0	1	0	1	0	0	0	0	0	0	0	0	0	0	1	0	0	0	1	1	33
**621**	0	0	0	0	0	0	0	0	0	0	0	0	0	1	0	0	0	0	0	1	0	1	0	1	1	33
**622**	0	0	0	0	1	0	0	0	0	1	0	1	1	0	0	0	0	0	0	0	0	0	0	1	0	33
**623**	0	0	0	1	1	0	0	0	0	1	0	1	0	0	0	0	0	0	0	0	0	0	0	1	0	33
**624**	0	1	0	0	0	0	0	0	1	0	0	1	0	0	0	0	0	0	0	0	0	1	1	1	1	33
**625**	0	0	1	0	0	0	0	0	0	0	0	1	0	1	0	0	0	0	0	0	0	1	0	1	1	33
**626**	0	1	0	1	0	0	0	0	1	0	0	1	0	0	0	0	0	0	0	0	0	1	0	1	1	33
**627**	0	0	0	0	1	0	0	0	0	0	0	1	0	0	0	0	0	0	0	1	0	0	0	1	0	33
**628**	0	0	0	0	0	0	0	0	1	1	0	0	0	0	0	0	0	0	0	0	0	1	0	1	1	32
**629**	0	1	0	0	0	0	0	0	0	1	0	1	0	1	0	0	0	0	0	1	0	0	0	1	0	32
**630**	0	0	1	0	0	0	0	0	0	0	0	1	0	0	0	1	0	0	0	0	0	0	0	1	1	32
**631**	0	0	0	0	0	0	1	0	1	0	0	1	0	1	0	0	0	0	0	1	0	1	0	1	1	32
**632**	0	0	0	0	0	0	1	0	0	1	0	1	1	1	0	0	0	0	0	1	0	0	0	1	1	32
**633**	0	0	1	0	0	0	0	0	1	0	0	1	0	0	0	0	0	0	0	1	0	0	0	1	1	32
**634**	0	1	0	0	0	0	0	0	0	0	0	0	0	1	0	0	0	0	0	0	0	0	0	1	1	31
**635**	0	0	0	1	1	0	0	0	1	0	0	1	0	0	0	0	0	0	0	1	0	0	0	1	1	31
**636**	0	0	0	0	0	0	0	0	0	0	0	0	1	0	0	1	0	0	0	0	0	0	1	1	0	31
**637**	0	1	0	0	1	0	1	0	0	1	0	1	0	1	0	0	0	0	0	1	0	1	0	1	1	31
**638**	0	1	0	0	1	0	0	0	1	0	0	1	1	0	0	0	0	0	0	0	0	1	0	1	0	31
**639**	0	0	1	0	1	0	1	0	1	0	0	1	0	0	0	0	0	0	0	1	0	0	0	1	1	31
**640**	0	0	0	0	1	0	1	0	0	0	0	1	0	0	0	0	0	0	0	1	0	0	0	1	1	31
**641**	0	0	0	0	1	0	1	0	0	1	0	1	0	0	0	0	0	0	0	0	0	1	0	1	1	31
**642**	0	0	0	0	0	0	0	0	0	0	1	0	0	1	0	0	0	0	0	0	0	1	0	1	1	31
**643**	0	1	0	0	1	0	0	0	0	0	0	1	0	0	0	0	0	0	0	0	0	1	1	1	1	31
**644**	0	1	1	0	1	0	0	0	1	1	0	1	0	0	0	0	0	0	0	1	0	0	0	1	1	31
**645**	0	0	1	0	0	0	1	0	0	1	0	1	0	1	0	0	0	0	0	1	0	1	1	1	0	30
**646**	0	0	1	0	1	0	0	0	0	1	0	0	0	0	0	0	0	0	0	0	0	0	0	1	1	30
**647**	0	1	1	0	1	0	0	0	1	1	0	1	0	0	0	0	0	0	0	0	0	0	0	1	1	30
**648**	0	0	1	0	0	0	1	0	0	1	0	0	0	1	0	0	0	0	0	0	0	0	0	1	1	30
**649**	0	0	0	1	1	0	0	0	1	0	0	1	0	0	0	0	0	0	0	0	0	0	0	1	1	30
**650**	0	1	0	1	1	0	0	0	1	0	0	1	1	0	0	0	0	0	0	1	0	1	0	1	1	30
**651**	0	0	0	0	1	0	0	0	0	1	0	1	0	1	0	0	0	0	0	0	0	1	0	1	1	30
**652**	0	0	1	0	0	0	1	0	0	0	0	0	1	0	0	1	0	0	0	0	0	0	0	1	0	30
**653**	0	1	0	1	1	0	0	0	0	0	0	1	0	0	0	0	0	0	0	0	0	0	1	1	0	30
**654**	0	0	1	0	0	0	0	0	1	1	0	0	0	0	0	0	0	0	0	1	0	0	0	1	0	30
**655**	0	0	1	0	0	0	0	0	0	0	0	0	0	1	0	0	0	0	0	1	0	0	0	1	0	30
**656**	0	0	1	1	0	0	1	0	1	1	0	1	0	1	0	1	0	0	0	1	0	0	0	1	1	30
**657**	0	0	0	0	0	0	0	0	0	1	0	0	0	0	0	0	0	0	0	1	0	1	0	1	0	30
**658**	0	0	1	0	1	0	0	0	1	0	0	1	0	0	0	0	0	0	0	1	0	0	0	1	1	30
**659**	0	0	0	0	0	0	1	0	0	0	0	0	0	0	0	0	0	0	0	1	0	1	0	1	1	30
**660**	0	0	0	1	0	0	0	0	0	0	0	1	0	0	0	0	0	0	0	1	0	0	0	1	0	30
**661**	0	0	1	0	1	0	1	0	1	1	0	1	0	0	0	0	0	0	0	1	0	0	0	1	1	30
**662**	0	0	1	0	0	0	0	0	0	1	1	1	0	0	0	0	0	0	0	0	0	0	0	1	1	30
**663**	0	0	0	0	0	0	1	0	0	1	0	1	0	1	0	0	0	0	0	0	0	0	1	1	0	30
**664**	0	0	0	1	0	0	0	0	0	0	0	1	0	0	0	0	0	0	0	0	0	1	0	1	0	30
**665**	0	1	1	0	1	0	0	0	1	0	0	1	0	0	0	0	0	0	0	0	0	0	0	1	0	29
**666**	0	1	1	0	0	0	0	0	1	0	0	1	0	0	0	0	0	0	0	1	0	0	0	1	1	29
**667**	0	0	0	0	0	0	1	0	0	0	0	1	0	0	0	0	0	0	0	1	0	0	1	1	1	29
**668**	0	1	1	0	1	0	1	0	1	1	0	1	0	0	0	0	0	0	0	0	0	0	0	1	1	29
**669**	0	1	0	1	1	0	0	0	0	1	0	1	0	1	0	0	0	0	0	0	0	1	0	1	1	29
**670**	0	1	0	0	0	0	0	0	0	0	0	1	0	1	0	0	0	0	0	0	0	0	0	1	0	29
**671**	0	0	1	1	0	0	1	0	0	1	0	0	0	1	0	0	0	0	0	1	0	0	0	1	1	29
**672**	0	0	0	1	0	0	0	0	0	0	0	1	0	0	0	0	0	0	0	0	0	1	0	1	1	29
**673**	0	0	0	0	1	0	0	0	0	0	0	0	0	0	0	0	0	0	0	1	0	0	0	1	1	29
**674**	0	1	0	1	1	0	0	0	0	0	0	1	0	0	0	0	0	0	0	0	0	0	1	1	1	29
**675**	0	0	0	0	0	0	1	0	0	0	0	1	0	0	0	0	0	0	0	0	0	1	0	1	0	29
**676**	0	0	1	0	1	0	0	0	0	0	0	1	0	0	0	0	0	0	0	0	0	0	0	1	1	28
**677**	0	1	0	0	1	0	0	0	1	0	0	1	1	0	0	0	0	0	0	1	0	0	0	1	0	28
**678**	0	0	1	0	0	0	1	0	1	1	0	1	0	1	0	1	0	0	0	1	0	0	0	1	1	28
**679**	0	1	0	1	1	0	0	0	0	0	0	1	1	0	0	0	0	0	0	0	0	1	0	1	1	28
**680**	0	0	0	0	0	0	1	0	0	0	0	0	1	0	0	1	0	0	0	0	0	1	0	1	0	28
**681**	0	1	0	0	1	0	0	0	0	0	0	0	0	0	0	0	0	0	0	1	0	0	0	1	1	28
**682**	0	1	1	0	0	0	0	0	1	1	0	1	0	0	0	0	0	0	0	1	0	0	0	1	1	28
**683**	0	0	1	0	0	0	0	0	1	1	0	1	0	1	0	0	0	0	0	1	0	1	0	1	0	28
**684**	0	0	1	0	0	0	1	0	0	1	0	0	1	1	0	0	0	0	0	1	0	0	0	1	1	28
**685**	0	0	0	0	0	0	0	0	1	0	0	0	0	0	0	1	0	0	0	0	0	0	0	1	1	28

	CD2	CD3	CD4	CD7	CD8	CD10	CD13	CD18	CD26	CD29	CD36	CD44	CD45RA	CD49f	CD54	CD56	CD57	CD58	CD62L	CD71	CD80	Cytokeratin	Histone_H1	HLA-DQ	HLA-DR	Freq. (Voxel)
**686**	0	1	0	0	0	0	1	0	0	0	0	1	0	0	0	0	0	0	0	1	0	0	0	1	1	28
**687**	0	0	1	0	0	0	1	0	0	1	0	0	0	1	0	0	0	0	0	0	0	0	0	1	0	28
**688**	0	0	1	0	0	0	0	0	0	1	1	1	0	1	0	0	0	0	0	0	0	0	0	1	1	28
**689**	0	0	1	0	0	0	0	0	0	0	0	0	1	0	0	0	0	0	0	1	0	0	0	1	1	27
**690**	0	0	1	0	0	0	0	0	0	1	1	1	0	0	0	0	0	0	0	1	0	0	0	1	1	27
**691**	0	0	0	0	0	0	0	0	1	0	0	0	0	0	0	0	0	0	0	0	0	1	1	1	0	27
**692**	0	0	1	1	0	0	1	0	0	1	0	1	0	0	0	0	0	0	0	0	0	0	0	1	1	27
**693**	0	1	0	0	1	0	0	0	0	1	1	1	0	1	0	0	0	0	0	0	0	0	1	1	1	27
**694**	0	0	1	0	0	0	0	0	1	0	0	1	0	0	0	0	0	0	0	1	0	1	0	1	0	27
**695**	0	1	0	0	0	0	0	0	0	0	0	1	0	1	0	0	0	0	0	1	0	1	0	1	1	27
**696**	0	1	0	1	1	0	0	0	0	0	0	1	1	0	0	0	0	0	0	1	0	1	0	1	1	27
**697**	0	0	0	0	1	0	0	0	0	0	0	0	0	0	0	0	0	0	0	0	0	0	1	1	1	27
**698**	0	1	0	0	1	0	0	0	0	0	0	1	0	0	0	0	0	0	0	0	0	1	1	1	0	27
**699**	0	1	0	1	1	0	0	0	1	0	0	1	1	0	0	0	0	0	0	1	0	0	0	1	0	27
**700**	0	1	0	1	1	0	1	0	0	1	0	1	0	0	0	0	0	0	0	0	0	0	0	1	1	27
**701**	0	0	0	0	1	0	0	0	0	1	0	1	0	1	0	0	0	0	0	1	0	1	0	1	1	27
**702**	0	0	0	0	0	0	0	0	1	0	0	0	0	1	0	0	0	0	0	0	0	0	0	1	1	27
**703**	0	1	0	0	1	0	0	0	1	1	0	1	0	0	0	0	0	0	0	1	0	0	0	1	0	27
**704**	0	0	0	0	0	0	0	0	0	0	0	1	1	0	0	0	0	0	0	0	0	0	0	1	0	27
**705**	0	0	0	0	1	0	0	0	0	0	0	1	0	0	0	1	0	0	0	0	0	0	0	1	1	26
**706**	0	0	0	1	0	0	0	0	0	0	0	0	0	1	0	0	0	0	0	0	0	1	0	1	1	26
**707**	0	0	0	0	1	0	1	0	1	1	0	1	0	0	0	0	0	0	0	1	0	1	0	1	1	26
**708**	0	0	1	0	1	0	0	0	0	1	0	1	0	0	0	0	0	0	0	0	0	0	0	1	1	26
**709**	0	0	1	0	0	0	0	0	0	0	0	0	1	1	0	0	0	0	0	0	0	0	0	1	0	26
**710**	0	0	0	0	1	0	1	0	0	0	0	1	0	0	0	0	0	0	0	0	0	0	0	1	0	26
**711**	0	1	0	0	0	0	0	0	0	0	0	1	0	0	0	0	0	0	0	1	0	0	1	1	1	26
**712**	0	1	0	0	1	0	0	0	1	1	0	1	0	0	0	0	0	0	0	1	0	0	1	1	1	26
**713**	0	1	0	1	0	0	0	0	0	0	0	1	0	0	0	0	0	0	0	1	0	1	0	1	1	26
**714**	0	0	0	0	0	0	1	0	1	1	0	1	0	1	0	0	0	0	0	0	0	0	1	1	1	26
**715**	0	0	1	0	0	0	1	0	1	0	0	0	0	0	0	0	0	0	0	0	0	0	0	1	1	26
**716**	0	0	0	0	0	0	0	0	0	1	0	1	0	1	0	0	0	0	0	1	0	0	1	1	0	26
**717**	0	1	1	0	0	0	0	0	0	1	0	1	0	0	0	0	0	0	0	1	0	0	0	1	1	26
**718**	0	1	1	0	0	0	0	0	0	1	0	1	0	0	0	0	0	0	0	0	0	0	0	1	1	26
**719**	0	0	0	0	0	0	1	0	1	1	0	1	0	0	0	0	0	0	0	1	0	0	1	1	1	25
**720**	0	0	0	0	0	0	0	0	1	1	0	0	0	0	0	0	0	0	0	0	0	0	1	1	0	25
**721**	0	0	0	0	0	0	0	0	0	0	1	0	0	0	0	0	0	0	0	1	0	0	0	1	0	25
**722**	0	0	0	0	0	0	0	0	1	0	0	0	1	0	0	0	0	0	0	1	0	0	0	1	1	25
**723**	0	1	1	0	1	0	0	0	0	0	0	1	0	0	0	0	0	0	0	0	0	0	0	1	1	25
**724**	0	1	0	0	1	0	0	0	0	1	0	1	1	0	0	0	0	0	0	0	0	0	1	1	1	25
**725**	0	0	0	0	0	0	1	0	1	1	0	0	0	1	0	0	0	0	0	0	0	0	0	1	1	25
**726**	0	0	1	0	1	0	0	0	0	0	0	1	0	0	0	0	0	0	0	1	0	0	0	1	1	25
**727**	0	0	0	0	0	0	1	0	0	1	1	1	0	0	0	0	0	0	0	0	0	0	0	1	1	25
**728**	0	0	0	0	0	0	0	0	0	0	0	1	1	0	0	0	0	0	0	0	0	1	0	1	1	25
**729**	0	1	1	0	0	0	0	0	1	1	0	1	0	0	0	0	0	0	0	0	0	0	0	1	1	25
**730**	0	0	1	0	0	0	1	0	0	1	0	1	1	1	0	0	0	0	0	0	0	0	0	1	1	25
**731**	0	1	0	0	1	0	0	0	0	1	0	1	0	1	0	0	0	0	0	0	0	1	0	1	1	25
**732**	0	0	0	0	0	0	0	0	0	0	0	0	1	0	0	1	0	0	0	0	0	0	0	1	1	25
**733**	0	0	1	0	0	0	1	0	1	1	0	1	1	1	0	0	0	0	0	1	0	0	0	1	1	25
**734**	0	1	0	0	1	0	1	0	1	1	0	1	0	0	0	0	0	0	0	0	0	0	0	1	1	25
**735**	0	0	1	0	1	0	0	0	0	1	0	1	0	0	0	0	0	0	0	1	0	0	0	1	1	25
**736**	0	1	0	0	1	0	0	0	1	1	0	1	0	0	0	0	0	0	0	0	0	0	1	1	0	25
**737**	0	0	1	0	0	0	0	0	0	0	1	1	0	0	0	0	0	0	0	0	0	0	0	1	1	25
**738**	0	0	1	0	0	0	0	0	0	1	0	0	0	0	0	0	0	0	0	0	0	1	0	1	1	25
**739**	0	1	0	0	0	0	0	0	0	0	0	0	0	1	0	0	0	0	0	0	0	0	0	1	0	25
**740**	0	0	0	0	0	0	0	0	0	1	0	0	0	1	0	0	0	0	0	0	0	0	1	1	0	25
**741**	0	0	1	0	0	0	1	0	1	0	0	1	0	0	0	0	0	0	0	0	0	1	0	1	1	25
**742**	0	0	1	0	0	0	0	0	0	1	0	1	0	1	0	0	0	0	0	1	0	1	1	1	1	24
**743**	0	1	0	1	0	0	0	0	0	0	0	1	0	0	0	0	0	0	0	0	0	0	1	1	1	24
**744**	0	0	0	0	0	0	1	0	0	1	0	1	1	1	0	0	0	0	0	0	0	0	0	1	1	24
**745**	0	0	1	0	0	0	0	0	1	1	0	0	0	0	0	0	0	0	0	0	0	0	0	1	0	24
**746**	0	1	0	0	0	0	0	0	1	1	0	1	0	0	0	0	0	0	0	1	0	0	0	1	0	24
**747**	0	0	0	0	0	0	1	0	1	0	0	0	1	0	0	0	0	0	0	1	0	0	0	1	0	24
**748**	0	0	0	0	0	0	1	0	1	0	0	1	0	1	0	0	0	0	0	1	0	0	0	1	1	24
**749**	0	1	0	0	0	0	0	0	0	0	0	0	0	0	0	0	0	0	0	0	0	1	0	1	0	24
**750**	0	0	0	1	1	0	0	0	1	1	0	1	0	0	0	0	0	0	0	1	0	0	0	1	1	24
**751**	0	0	0	1	1	0	1	0	0	0	0	1	0	0	0	0	0	0	0	1	0	0	0	1	1	24
**752**	0	1	0	0	0	0	1	0	1	0	0	1	0	0	0	0	0	0	0	0	0	0	0	1	1	23
**753**	0	1	0	0	1	0	1	0	1	1	0	1	0	0	0	0	0	0	0	1	0	0	0	1	1	23
**754**	0	0	0	0	1	0	0	0	1	1	0	1	0	0	0	0	0	0	0	1	0	1	0	1	1	23
**755**	0	0	1	0	0	0	1	0	0	0	0	1	0	0	0	0	0	0	0	0	0	0	0	1	0	23
**756**	0	0	0	0	0	0	1	0	1	0	0	1	0	1	0	0	0	0	0	0	0	0	1	1	1	23
**757**	0	0	1	0	1	0	0	0	0	0	0	0	0	0	0	0	0	0	0	0	0	0	0	1	1	23
**758**	0	1	0	0	0	0	0	0	0	1	0	1	0	1	0	0	0	0	0	0	0	1	0	1	1	23
**759**	0	0	0	0	0	0	0	0	1	0	0	0	0	0	0	0	0	0	0	1	0	0	1	1	0	23
**760**	0	1	0	0	1	0	0	0	1	0	0	1	0	1	0	0	0	0	0	1	0	1	0	1	1	23
**761**	0	0	1	1	0	0	1	0	1	1	1	1	1	1	0	1	0	0	0	1	0	0	0	1	1	23
**762**	0	1	1	0	1	0	0	0	1	0	0	1	1	0	0	0	0	0	0	1	0	0	0	1	1	23
**763**	0	1	1	1	1	0	0	0	0	1	0	1	0	1	0	0	0	0	0	1	0	0	0	1	1	23
**764**	0	0	0	0	0	0	1	0	0	0	1	1	0	0	0	0	0	0	0	0	0	0	0	1	1	23
**765**	0	1	0	0	1	0	0	0	0	0	0	1	0	0	0	0	0	0	0	1	0	1	0	1	0	23
**766**	0	0	0	1	0	0	0	0	0	0	0	0	0	0	0	0	0	0	0	1	0	0	0	1	0	23
**767**	0	1	0	1	0	0	0	0	1	1	0	1	0	0	0	0	0	0	0	1	0	0	0	1	1	23
**768**	0	0	1	0	0	0	1	0	0	1	0	0	0	1	0	0	0	0	0	1	0	0	0	1	1	23
**769**	0	0	1	0	0	0	0	0	0	0	0	0	0	0	0	0	0	0	0	0	0	1	1	1	1	22
**770**	0	0	0	0	0	0	0	0	0	0	0	0	0	1	0	0	0	0	0	1	0	0	1	1	1	22
**771**	0	0	1	0	0	0	1	0	0	0	0	0	1	0	0	0	0	0	0	1	0	1	0	1	0	22
**772**	0	1	0	0	1	0	0	0	0	0	0	1	0	1	0	0	0	0	0	0	0	1	0	1	1	22
**773**	0	0	1	0	0	0	0	0	0	0	0	1	0	1	0	0	0	0	0	1	0	1	0	1	1	22
**774**	0	0	1	0	0	0	0	0	0	0	0	1	0	1	0	0	0	0	0	0	0	0	0	1	0	22
**775**	0	0	1	0	1	0	0	0	1	0	0	1	0	0	0	0	0	0	0	0	0	0	0	1	1	22
**776**	0	0	1	1	0	0	1	0	1	1	1	1	1	1	0	0	0	0	0	1	0	0	0	1	1	22
**777**	0	1	0	1	1	0	1	0	0	0	0	1	1	0	0	0	0	0	0	1	0	0	0	1	1	22
**778**	0	0	0	0	0	0	1	0	0	0	0	0	1	0	0	0	0	0	0	0	0	1	0	1	0	22
**779**	0	0	1	0	0	0	1	0	0	0	0	1	0	0	0	1	0	0	0	0	0	0	0	1	1	22
**780**	0	0	1	0	0	0	0	0	0	0	0	0	1	0	0	0	0	0	0	0	0	0	0	1	1	22
**781**	0	0	0	0	0	0	0	0	1	1	0	0	0	0	0	0	0	0	0	0	0	0	0	1	0	22
**782**	0	0	0	0	0	0	0	0	0	0	0	0	0	0	0	1	0	0	0	1	0	0	0	1	1	22
**783**	0	0	0	1	1	0	1	0	0	0	0	1	0	0	0	0	0	0	0	0	0	0	0	1	1	22

	CD2	CD3	CD4	CD7	CD8	CD10	CD13	CD18	CD26	CD29	CD36	CD44	CD45RA	CD49f	CD54	CD56	CD57	CD58	CD62L	CD71	CD80	Cytokeratin	Histone_H1	HLA-DQ	HLA-DR	Freq. (Voxel)
**784**	0	1	0	1	1	0	0	0	1	0	0	1	1	0	0	0	0	0	0	0	0	0	0	1	0	22
**785**	0	0	1	0	0	0	1	0	0	1	0	1	0	1	0	0	0	0	0	0	0	1	0	1	0	22
**786**	0	0	0	1	0	0	1	0	0	1	0	1	0	0	0	0	0	0	0	0	0	0	0	1	1	22
**787**	0	1	1	0	0	0	0	0	1	0	0	1	0	0	0	0	0	0	0	0	0	0	0	1	1	22
**788**	0	0	1	0	0	0	1	0	0	1	1	1	1	1	0	0	0	0	0	1	0	0	0	1	1	22
**789**	0	0	1	1	0	0	1	0	0	1	0	1	0	0	0	0	0	0	0	1	0	0	0	1	1	22
**790**	0	0	1	0	0	0	0	0	0	1	0	1	0	0	0	0	0	0	0	1	0	1	0	1	0	22
**791**	0	1	0	1	0	0	0	0	0	1	0	1	0	1	0	0	0	0	0	1	0	0	0	1	0	21
**792**	0	0	1	0	0	0	0	0	0	0	0	1	0	0	0	0	0	0	0	0	0	0	1	1	0	21
**793**	0	1	0	0	1	0	0	0	1	0	0	1	1	0	0	0	0	0	0	0	0	0	0	1	0	21
**794**	0	0	0	0	0	0	1	0	0	1	0	0	0	0	0	0	0	0	0	0	0	0	1	1	1	21
**795**	0	1	1	1	1	0	1	0	1	1	0	1	1	0	0	0	0	0	0	1	0	1	0	1	1	21
**796**	0	0	1	0	0	0	0	0	0	0	0	1	0	1	0	0	0	0	0	0	0	1	0	1	0	21
**797**	0	0	0	0	0	0	0	0	1	0	0	0	1	0	0	0	0	0	0	0	0	0	0	1	0	21
**798**	0	0	0	0	0	0	0	0	0	0	0	0	1	1	0	1	0	0	0	0	0	0	0	1	0	21
**799**	0	0	0	0	0	0	1	0	0	0	0	0	0	1	0	0	0	0	0	0	0	1	0	1	0	21
**800**	0	0	1	0	0	0	1	0	1	1	0	1	0	1	0	0	0	0	0	1	0	1	0	1	0	21
**801**	0	1	0	0	1	0	0	0	0	0	0	1	1	0	0	0	0	0	0	0	0	1	1	1	1	21
**802**	0	1	0	0	1	0	0	0	1	1	0	1	1	0	0	0	0	0	0	0	0	0	1	1	1	21
**803**	0	0	0	0	0	0	0	0	0	1	0	0	0	0	0	1	0	0	0	0	0	0	0	1	1	21
**804**	0	0	0	0	0	0	1	0	0	1	0	1	0	0	0	0	0	0	0	0	0	0	1	1	0	21
**805**	0	1	0	0	0	0	0	0	1	0	0	1	0	0	0	0	0	0	0	0	0	1	0	1	0	21
**806**	0	1	0	1	1	0	0	0	0	0	0	1	1	0	0	0	0	0	0	0	0	0	0	1	0	21
**807**	0	1	1	0	1	0	0	0	0	0	0	0	0	0	0	0	0	0	0	0	0	0	0	1	1	20
**808**	0	0	0	1	0	0	0	0	0	0	0	0	1	0	0	1	0	0	0	1	0	0	0	1	0	20
**809**	0	0	0	1	0	0	0	0	0	0	0	0	0	0	0	0	0	0	0	0	0	0	1	1	0	20
**810**	0	1	1	0	1	0	0	0	1	0	0	1	0	0	0	0	0	0	0	1	0	0	0	1	1	20
**811**	0	0	1	0	0	0	1	0	0	1	0	1	1	1	0	0	0	0	0	1	0	1	0	1	1	20
**812**	0	1	1	0	1	0	0	0	1	1	0	1	1	0	0	0	0	0	0	1	0	0	0	1	0	20
**813**	0	1	0	1	1	0	0	0	0	1	1	1	0	1	0	0	0	0	0	0	0	0	1	1	1	20
**814**	0	1	0	1	1	0	0	0	0	0	0	0	0	0	0	0	0	0	0	1	0	0	0	1	1	20
**815**	0	1	1	1	1	0	0	0	1	0	0	1	1	0	0	0	0	0	0	1	0	0	0	1	0	20
**816**	0	0	0	0	0	0	0	0	1	0	0	0	1	0	0	1	0	0	0	1	0	0	0	1	0	20
**817**	0	0	1	0	1	0	0	0	1	1	0	1	0	0	0	0	0	0	0	1	0	0	0	1	1	20
**818**	0	1	1	0	1	0	0	0	1	0	0	1	0	0	0	0	0	0	0	0	0	0	0	1	1	20
**819**	0	1	0	0	0	0	0	0	0	0	0	1	0	0	0	0	0	0	0	1	0	1	0	1	0	20
**820**	0	0	0	0	0	0	0	0	1	1	0	0	0	0	0	0	0	0	0	0	0	1	0	1	0	20
**821**	0	0	0	0	0	0	1	0	0	0	0	1	0	0	0	0	0	0	0	0	0	0	1	1	0	20
**822**	0	0	0	0	0	0	0	0	0	1	0	0	0	0	0	0	0	0	0	1	0	1	0	1	1	20
**823**	0	0	0	0	0	0	1	0	0	0	0	1	0	1	0	0	0	0	0	0	0	0	1	1	0	20
**824**	0	0	0	0	0	0	0	0	1	0	0	1	0	1	0	0	0	0	0	0	0	0	0	1	1	20
**825**	0	0	0	0	0	0	0	0	0	1	0	0	0	0	0	0	0	0	0	1	0	0	1	1	1	20
**826**	0	0	0	0	0	0	1	0	0	0	1	0	0	0	0	0	0	0	0	0	0	0	0	1	1	20
**827**	0	1	0	1	1	0	0	0	1	0	0	1	0	1	0	0	0	0	0	1	0	1	0	1	1	20
**828**	0	0	1	0	0	0	0	0	0	0	0	1	0	0	0	0	0	0	0	1	0	1	0	1	0	20
**829**	0	0	0	0	1	0	0	0	1	0	0	0	0	0	0	0	0	0	0	0	0	0	0	1	1	20
**830**	0	0	0	0	1	0	0	0	0	1	0	1	0	1	0	0	0	0	0	0	0	0	0	1	1	20
**831**	0	0	1	0	1	0	0	0	1	0	0	0	0	0	0	0	0	0	0	0	0	0	0	1	1	19
**832**	0	1	1	0	1	0	0	0	0	0	0	0	0	0	0	0	0	0	0	1	0	0	0	1	1	19
**833**	0	0	1	0	0	0	0	0	1	0	0	0	1	0	0	0	0	0	0	0	0	0	0	1	0	19
**834**	0	1	0	0	1	0	0	0	1	0	0	1	0	1	0	0	0	0	0	0	0	1	0	1	1	19
**835**	0	0	0	0	0	0	0	0	1	1	0	1	0	0	0	0	0	0	0	0	0	0	1	1	1	19
**836**	0	0	1	0	0	0	0	0	1	1	0	1	0	1	0	0	0	0	0	0	0	0	0	1	1	19
**837**	0	0	0	0	0	0	0	0	0	0	0	1	0	0	0	1	0	0	0	1	0	0	0	1	1	19
**838**	0	1	0	0	1	0	0	0	0	0	0	0	0	0	0	0	0	0	0	0	0	0	1	1	1	19
**839**	0	0	1	0	0	0	0	0	1	1	0	1	0	0	0	0	0	0	0	0	0	0	0	1	0	19
**840**	0	0	0	0	0	0	0	0	0	0	1	1	0	0	0	0	0	0	0	1	0	0	0	1	1	19
**841**	0	0	0	0	0	0	0	0	0	0	1	0	0	0	0	0	0	0	0	1	0	1	0	1	0	19
**842**	0	0	0	1	0	0	0	0	0	0	0	0	0	0	0	0	0	0	0	0	0	1	0	1	1	19
**843**	0	1	0	0	1	0	0	0	1	0	0	1	1	0	0	0	0	0	0	1	0	0	1	1	1	19
**844**	0	1	1	1	1	0	0	0	0	0	0	1	0	0	0	0	0	0	0	1	0	0	0	1	1	19
**845**	0	0	0	0	0	0	0	0	0	0	0	0	1	0	0	0	0	0	0	1	0	0	0	1	1	19
**846**	0	0	0	0	0	0	0	0	0	0	0	0	0	0	0	1	0	0	0	1	0	0	0	1	0	19
**847**	0	1	0	1	1	0	1	0	0	0	0	1	0	0	0	0	0	0	0	0	0	0	1	1	1	19
**848**	0	0	0	1	1	0	0	0	0	1	0	1	0	0	0	0	0	0	0	1	0	0	0	1	0	19
**849**	0	1	1	1	1	0	0	0	1	1	0	1	1	0	0	0	0	0	0	1	0	0	0	1	0	19
**850**	0	1	0	1	1	0	0	0	0	0	0	1	1	0	0	0	0	0	0	0	0	0	1	1	1	19
**851**	0	1	0	0	0	0	0	0	0	1	0	1	0	1	0	0	0	0	0	0	0	0	0	1	0	19
**852**	0	0	0	0	0	0	0	0	0	0	1	0	0	1	0	0	0	0	0	0	0	0	0	1	1	19
**853**	0	1	0	0	1	0	0	0	0	0	0	1	1	0	0	0	0	0	0	0	0	0	1	1	0	19
**854**	0	0	1	0	0	0	0	0	0	0	0	0	0	0	0	1	0	0	0	0	0	0	1	1	0	18
**855**	0	1	0	1	1	0	0	0	0	0	0	1	0	1	0	0	0	0	0	1	0	1	0	1	1	18
**856**	0	0	0	0	1	0	1	0	1	0	0	1	0	0	0	1	0	0	0	0	0	0	0	1	1	18
**857**	0	1	0	0	0	0	0	0	0	0	0	1	1	1	0	0	0	0	0	0	0	0	0	1	1	18
**858**	0	1	0	0	0	0	0	0	1	0	0	1	0	1	0	0	0	0	0	0	0	0	0	1	1	18
**859**	0	0	0	0	0	0	1	0	0	0	1	0	0	1	0	0	0	0	0	0	0	1	0	1	1	18
**860**	0	0	1	0	0	0	0	0	0	0	0	0	1	1	0	0	0	0	0	1	0	0	0	1	0	18
**861**	0	1	0	0	1	0	0	0	0	0	0	1	1	0	0	0	0	0	0	1	0	1	1	1	1	18
**862**	0	0	0	1	0	0	0	0	1	0	0	0	0	0	0	0	0	0	0	1	0	1	0	1	0	18
**863**	0	1	0	1	0	0	0	0	0	1	0	1	0	0	0	0	0	0	0	1	0	0	0	1	0	18
**864**	0	0	0	0	0	0	0	0	1	1	0	1	0	1	0	0	0	0	0	0	0	0	0	1	1	18
**865**	0	1	0	1	0	0	0	0	1	1	0	1	0	0	0	0	0	0	0	0	0	1	0	1	1	18
**866**	0	0	0	0	1	0	0	0	0	1	0	1	0	0	0	0	0	0	0	1	0	1	0	1	0	18
**867**	0	1	0	1	1	0	0	0	0	1	0	1	1	0	0	0	0	0	0	0	0	0	0	1	0	18
**868**	0	0	0	0	1	0	0	0	0	1	0	1	0	0	0	0	0	0	0	0	0	1	1	1	1	18
**869**	0	0	0	0	0	0	0	0	0	0	0	1	0	0	0	1	0	0	0	0	0	1	0	1	0	18
**870**	0	0	0	0	0	0	0	0	0	1	0	0	1	0	0	1	0	0	0	0	0	0	0	1	0	18
**871**	0	1	0	1	1	0	0	0	0	1	0	1	0	1	0	0	0	0	0	1	0	1	0	1	1	18
**872**	0	0	1	0	0	0	1	0	1	0	0	0	0	0	0	0	0	0	0	0	0	0	0	1	0	17
**873**	0	1	0	0	0	0	1	0	0	1	0	1	0	1	0	0	0	0	0	0	0	1	0	1	1	17
**874**	0	0	0	1	0	0	0	0	0	1	0	0	0	0	0	0	0	0	0	0	0	0	1	1	1	17
**875**	0	0	0	0	1	0	0	0	0	1	0	1	0	0	0	0	0	0	0	0	0	0	1	1	1	17
**876**	0	1	0	0	0	0	0	0	0	0	0	1	0	1	0	0	0	0	0	0	0	0	1	1	1	17
**877**	0	0	0	0	0	0	0	0	0	0	1	0	0	0	0	0	0	0	0	0	0	0	1	1	1	17
**878**	0	0	0	0	0	0	1	0	0	1	0	0	0	1	0	0	0	0	0	1	0	1	1	1	0	17
**879**	0	0	0	0	0	0	0	0	1	0	0	0	1	0	0	1	0	0	0	0	0	0	0	1	0	17
**880**	0	0	1	0	0	0	0	0	0	1	0	0	0	1	0	0	0	0	0	0	0	0	1	1	0	17
**881**	0	0	0	0	0	0	1	0	1	1	0	1	0	1	0	1	0	0	0	0	0	0	0	1	1	17

	CD2	CD3	CD4	CD7	CD8	CD10	CD13	CD18	CD26	CD29	CD36	CD44	CD45RA	CD49f	CD54	CD56	CD57	CD58	CD62L	CD71	CD80	Cytokeratin	Histone_H1	HLA-DQ	HLA-DR	Freq. (Voxel)
**882**	0	0	1	0	0	0	1	0	1	1	1	1	0	1	0	0	0	0	0	1	0	0	0	1	1	17
**883**	0	0	0	1	0	0	0	0	1	0	0	1	0	0	0	0	0	0	0	0	0	1	0	1	0	17
**884**	0	0	1	0	1	0	1	0	0	0	0	0	1	0	0	1	0	0	0	1	0	1	0	1	0	17
**885**	0	0	1	0	0	0	1	0	0	1	0	1	0	1	0	0	0	0	0	0	0	0	1	1	1	17
**886**	0	0	1	0	0	0	1	0	0	1	0	1	0	1	0	0	0	0	0	1	0	0	1	1	1	17
**887**	0	1	0	0	1	0	0	0	0	0	0	1	0	0	0	0	0	0	0	1	0	1	1	1	0	17
**888**	0	1	0	1	1	0	0	0	0	0	0	1	0	0	0	0	0	0	0	1	0	1	0	1	0	17
**889**	0	0	1	1	0	0	1	0	1	1	0	1	0	1	0	0	0	0	0	1	0	1	0	1	1	17
**890**	0	0	0	0	0	0	1	0	0	0	0	0	0	1	0	0	0	0	0	0	0	0	1	1	1	17
**891**	0	0	0	0	0	0	0	0	0	1	1	1	0	0	0	0	0	0	0	0	0	1	0	1	0	17
**892**	0	1	0	0	1	0	0	0	1	1	0	1	0	0	0	0	0	0	0	1	0	1	0	1	1	17
**893**	0	0	1	0	0	0	0	0	1	0	0	1	0	1	0	0	0	0	0	1	0	1	0	1	0	17
**894**	0	1	0	0	0	0	0	0	1	0	0	1	0	0	0	0	0	0	0	0	0	0	1	1	1	17
**895**	0	0	0	0	0	0	0	0	0	0	1	1	0	1	0	0	0	0	0	1	0	0	0	1	1	17
**896**	0	0	1	0	0	0	1	0	0	1	0	1	0	0	0	0	0	0	0	1	0	0	0	1	0	17
**897**	0	0	1	0	0	0	1	0	0	1	0	0	0	0	0	0	0	0	0	1	0	0	0	1	1	17
**898**	0	1	0	1	0	0	0	0	1	0	0	1	0	0	0	0	0	0	0	0	0	0	0	1	0	17
**899**	0	0	0	0	0	0	0	0	1	0	0	1	1	0	0	0	0	0	0	1	0	1	0	1	1	17
**900**	0	0	0	0	0	0	0	0	0	1	0	0	0	0	0	0	0	0	0	0	0	1	1	1	1	17
**901**	0	0	0	0	0	0	0	0	0	1	1	0	0	1	0	0	0	0	0	0	0	0	0	1	1	17
**902**	0	1	0	1	1	0	1	0	1	0	0	1	0	0	0	1	0	0	0	1	0	0	0	1	1	16
**903**	0	0	0	0	0	0	0	0	0	0	1	1	0	0	0	0	0	0	0	1	0	1	0	1	1	16
**904**	0	1	0	1	1	0	0	0	0	1	0	1	1	0	0	0	0	0	0	1	0	1	0	1	1	16
**905**	0	0	0	0	0	0	0	0	0	1	0	1	0	0	0	1	0	0	0	0	0	1	0	1	0	16
**906**	0	1	1	0	1	0	0	0	0	1	0	1	1	0	0	0	0	0	0	0	0	0	1	1	1	16
**907**	0	0	0	1	0	0	0	0	0	1	0	0	0	0	0	0	0	0	0	1	0	0	0	1	1	16
**908**	0	1	1	0	0	0	0	0	1	0	0	1	0	0	0	0	0	0	0	0	0	0	0	1	0	16
**909**	0	0	0	1	0	0	1	0	0	1	1	1	0	1	0	0	0	0	0	1	0	0	0	1	1	16
**910**	0	0	1	0	0	0	0	0	0	0	0	0	1	1	0	1	0	0	0	0	0	1	0	1	0	16
**911**	0	0	0	1	1	0	0	0	0	0	0	0	0	0	0	0	0	0	0	1	0	0	0	1	1	16
**912**	0	0	0	0	1	0	0	0	1	0	0	1	1	0	0	0	0	0	0	1	0	1	0	1	1	16
**913**	0	0	0	1	0	0	1	0	0	1	0	1	0	1	0	1	0	0	0	0	0	0	0	1	1	16
**914**	0	0	0	1	0	0	0	0	0	0	0	0	0	0	0	0	0	0	0	0	0	0	1	1	1	16
**915**	0	0	0	0	0	0	1	0	1	1	0	1	0	1	0	1	0	0	0	1	0	0	0	1	1	16
**916**	0	0	0	0	0	0	0	0	1	1	0	1	0	0	0	0	0	0	0	0	0	0	1	1	0	16
**917**	0	0	0	0	0	0	1	0	0	0	0	1	0	1	0	0	0	0	0	1	0	1	0	1	1	16
**918**	0	0	1	0	0	0	1	0	0	0	0	0	1	0	0	1	0	0	0	0	0	1	0	1	0	16
**919**	0	1	0	0	0	0	0	0	0	1	0	1	0	0	0	0	0	0	0	0	0	1	1	1	0	16
**920**	0	1	1	0	1	0	0	0	0	1	0	0	0	0	0	0	0	0	0	0	0	0	0	1	1	16
**921**	0	1	0	1	0	0	0	0	0	0	0	1	0	0	0	0	0	0	0	1	0	1	0	1	0	16
**922**	0	0	0	0	0	0	1	0	0	0	0	1	0	1	0	0	0	0	0	0	0	1	0	1	1	16
**923**	0	0	0	0	0	0	0	0	1	0	0	0	0	1	0	0	0	0	0	1	0	0	0	1	1	16
**924**	0	0	1	1	0	0	0	0	0	0	0	0	1	0	0	1	0	0	0	0	0	0	0	1	0	16
**925**	0	0	0	1	1	0	0	0	1	1	0	1	0	0	0	0	0	0	0	0	0	0	0	1	1	16
**926**	0	0	0	0	0	0	0	0	0	1	0	1	1	0	0	0	0	0	0	0	0	0	0	1	0	16
**927**	0	1	0	1	1	0	0	0	1	0	0	1	1	0	0	0	0	0	0	0	0	1	0	1	1	16
**928**	0	0	1	0	0	0	0	0	0	1	1	0	0	1	0	0	0	0	0	0	0	0	0	1	1	16
**929**	0	0	0	0	0	0	1	0	1	1	0	1	0	1	0	0	0	0	0	1	0	0	1	1	1	16
**930**	0	0	0	0	0	0	0	0	0	1	0	0	1	1	0	0	0	0	0	0	0	0	0	1	0	16
**931**	0	0	0	0	0	0	1	0	0	1	1	1	0	1	0	0	0	0	0	0	0	0	0	1	0	16
**932**	0	0	0	0	0	0	0	0	0	1	0	1	0	1	0	0	0	0	0	1	0	0	1	1	1	16
**933**	0	1	0	0	1	0	0	0	0	0	0	1	1	0	0	0	0	0	0	0	0	1	0	1	0	16
**934**	0	0	0	0	0	0	1	0	0	0	0	0	0	0	0	0	0	0	0	1	0	1	0	1	0	16
**935**	0	0	0	0	0	0	1	0	1	0	0	0	0	1	0	0	0	0	0	0	0	1	0	1	1	16
**936**	0	0	1	0	0	0	0	0	0	1	0	0	0	0	0	0	0	0	0	0	0	1	0	1	0	16
**937**	0	1	1	1	1	0	1	0	0	1	0	1	0	0	0	0	0	0	0	1	0	0	0	1	1	15
**938**	0	0	0	1	0	0	0	0	1	0	0	1	0	0	0	0	0	0	0	0	0	1	0	1	1	15
**939**	0	0	1	0	0	0	1	0	0	0	0	0	1	0	0	1	0	0	0	1	0	1	0	1	0	15
**940**	0	1	0	0	0	0	0	0	0	0	0	1	0	1	0	0	0	0	0	1	0	0	0	1	1	15
**941**	0	1	1	1	1	0	0	0	1	1	0	1	0	0	0	0	0	0	0	0	0	0	0	1	0	15
**942**	0	0	0	0	0	0	0	0	0	1	0	1	1	1	0	0	0	0	0	1	0	0	0	1	1	15
**943**	0	0	0	1	1	0	0	0	0	0	0	1	1	0	0	0	0	0	0	1	0	0	0	1	1	15
**944**	0	0	0	0	1	0	0	0	1	1	0	1	0	1	0	0	0	0	0	1	0	1	0	1	1	15
**945**	0	0	1	0	1	0	0	0	0	0	0	0	0	0	0	0	0	0	0	1	0	0	0	1	1	15
**946**	0	0	1	0	0	0	0	0	0	0	0	0	0	1	0	0	0	0	0	0	0	0	1	1	0	15
**947**	0	0	0	0	0	0	1	0	1	0	0	0	1	0	0	0	0	0	0	0	0	0	0	1	0	15
**948**	0	1	0	1	1	0	1	0	1	1	0	1	0	0	0	0	0	0	0	0	0	0	0	1	1	15
**949**	0	1	1	0	1	0	0	0	0	0	0	1	0	0	0	0	0	0	0	1	0	0	0	1	1	15
**950**	0	1	0	0	0	0	0	0	0	1	0	1	0	0	0	0	0	0	0	1	0	1	0	1	1	15
**951**	0	0	1	0	0	0	0	0	0	0	0	0	1	0	0	1	0	0	0	0	0	0	0	1	1	15
**952**	0	0	1	1	0	0	0	0	0	1	0	0	0	1	0	0	0	0	0	1	0	0	0	1	1	15
**953**	0	1	0	1	1	0	1	0	1	0	0	1	0	0	0	0	0	0	0	1	0	0	1	1	1	15
**954**	0	0	0	1	0	0	0	0	1	1	0	1	0	0	0	0	0	0	0	0	0	0	0	1	1	15
**955**	0	1	0	0	0	0	0	0	1	1	0	1	0	0	0	0	0	0	0	0	0	0	1	1	0	15
**956**	0	1	0	0	0	0	0	0	0	1	0	1	1	0	0	0	0	0	0	0	0	1	0	1	0	15
**957**	0	1	0	0	0	0	1	0	0	0	0	1	0	0	0	0	0	0	0	0	0	0	0	1	0	15
**958**	0	0	0	0	0	0	0	0	0	1	0	0	0	0	0	0	0	0	0	0	0	1	1	1	0	15
**959**	0	0	0	0	1	0	0	0	1	0	0	1	0	0	0	0	0	0	0	0	0	1	0	1	0	15
**960**	0	1	0	1	1	0	0	0	0	1	0	1	1	0	0	0	0	0	0	1	0	0	0	1	0	15
**961**	0	0	0	0	0	0	1	0	0	1	0	1	0	1	0	0	0	0	0	1	0	0	1	1	0	14
**962**	0	0	0	0	0	0	1	0	0	0	0	1	0	1	0	0	0	0	0	1	0	0	1	1	1	14
**963**	0	1	0	0	0	0	0	0	0	0	0	1	0	0	0	0	0	0	0	1	0	1	1	1	0	14
**964**	0	0	0	0	0	0	1	0	1	1	0	1	0	1	0	0	0	0	0	1	0	1	1	1	1	14
**965**	0	1	0	1	1	0	0	0	0	0	0	0	1	0	0	0	0	0	0	0	0	0	0	1	0	14
**966**	0	0	1	0	0	0	0	0	1	1	0	1	0	1	0	0	0	0	0	0	0	1	0	1	1	14
**967**	0	0	0	0	0	0	0	0	1	0	0	1	0	1	0	0	0	0	0	1	0	0	0	1	1	14
**968**	0	1	0	1	1	0	0	0	1	0	0	1	0	0	0	0	0	0	0	0	0	1	0	1	1	14
**969**	0	1	0	0	1	0	0	0	1	1	0	1	0	1	0	0	0	0	0	0	0	0	0	1	1	14
**970**	0	0	0	0	0	0	1	0	0	0	1	1	0	1	0	0	0	0	0	0	0	0	0	1	1	14
**971**	0	0	1	0	0	0	0	0	0	1	0	1	0	1	0	0	0	0	0	0	0	0	1	1	0	14
**972**	0	1	1	1	1	0	0	0	1	0	0	1	0	0	0	0	0	0	0	1	0	1	0	1	1	14
**973**	0	1	0	0	1	0	0	0	1	1	0	1	0	0	0	0	0	0	0	0	0	1	1	1	0	14
**974**	0	1	0	0	1	0	0	0	1	1	0	1	1	0	0	0	0	0	0	0	0	1	0	1	0	14
**975**	0	0	0	0	0	0	0	0	0	1	0	1	0	0	0	0	0	0	0	1	0	1	1	1	0	14
**976**	0	1	0	0	0	0	0	0	1	0	0	1	0	1	0	0	0	0	0	0	0	1	0	1	1	14
**977**	0	0	0	0	0	0	1	0	1	0	0	1	0	1	0	0	0	0	0	0	0	1	0	1	1	14
**978**	0	0	1	1	0	0	1	0	0	1	0	1	0	1	0	1	0	0	0	1	0	0	0	1	1	14
**979**	0	1	1	0	1	0	0	0	0	1	0	1	0	0	0	0	0	0	0	0	0	0	0	1	1	14

	CD2	CD3	CD4	CD7	CD8	CD10	CD13	CD18	CD26	CD29	CD36	CD44	CD45RA	CD49f	CD54	CD56	CD57	CD58	CD62L	CD71	CD80	Cytokeratin	Histone_H1	HLA-DQ	HLA-DR	Freq. (Voxel)
**980**	0	1	1	0	1	0	0	0	0	1	0	1	1	0	0	0	0	0	0	0	0	0	0	1	1	14
**981**	0	1	0	1	1	0	0	0	0	1	1	1	0	1	0	0	0	0	0	0	0	1	0	1	1	14
**982**	0	0	1	0	0	0	1	0	1	0	0	0	0	1	0	0	0	0	0	0	0	0	0	1	1	14
**983**	0	0	0	1	0	0	0	0	0	0	0	0	0	0	0	0	0	0	0	1	0	1	0	1	1	14
**984**	0	1	0	0	0	0	0	0	1	0	0	1	1	0	0	0	0	0	0	1	0	1	0	1	1	14
**985**	0	1	1	0	1	0	0	0	0	1	0	1	0	0	0	0	0	0	0	1	0	0	0	1	0	14
**986**	0	1	1	1	1	0	0	0	1	1	0	1	1	0	0	0	0	0	0	1	0	1	0	1	1	14
**987**	0	0	0	1	1	0	0	0	1	1	0	1	1	0	0	0	0	0	0	1	0	0	0	1	1	14
**988**	0	0	0	0	0	0	0	0	1	1	0	1	0	1	0	0	0	0	0	1	0	0	0	1	0	14
**989**	0	1	0	1	0	0	0	0	0	0	0	1	0	0	0	0	0	0	0	0	0	0	1	1	0	14
**990**	0	1	0	0	0	0	0	0	1	1	0	1	0	0	0	0	0	0	0	1	0	0	0	1	1	14
**991**	0	0	0	1	0	0	0	0	1	0	0	1	0	0	0	0	0	0	0	0	0	0	0	1	1	14
**992**	0	0	0	1	0	0	0	0	1	0	0	1	0	0	0	0	0	0	0	1	0	0	0	1	1	14
**993**	0	1	0	0	1	0	0	0	1	0	0	0	0	0	0	0	0	0	0	1	0	1	1	1	1	13
**994**	0	1	0	1	1	0	0	0	1	1	0	1	1	0	0	0	0	0	0	1	0	1	0	1	1	13
**995**	0	1	0	1	1	0	0	0	0	0	0	0	0	0	0	0	0	0	0	0	0	0	1	1	1	13
**996**	0	0	1	0	0	0	1	0	0	1	0	0	0	0	0	0	0	0	0	0	0	0	0	1	0	13
**997**	0	0	0	0	1	0	0	0	0	0	0	1	0	0	0	0	0	0	0	1	0	0	1	1	1	13
**998**	0	1	0	1	1	0	0	0	1	0	0	1	0	0	0	0	0	0	0	1	0	0	1	1	1	13
**999**	0	0	0	0	1	0	0	0	0	0	0	1	1	0	0	0	0	0	0	1	0	0	0	1	1	13
**1.000**	0	1	0	0	0	0	0	0	0	0	0	0	0	1	0	0	0	0	0	1	0	0	0	1	0	13
**1.001**	0	1	0	0	1	0	0	0	1	0	0	1	0	1	0	0	0	0	0	0	0	0	0	1	1	13
**1.002**	0	0	1	0	0	0	0	0	0	0	1	1	0	1	0	0	0	0	0	0	0	0	0	1	1	13
**1.003**	0	0	0	0	0	0	0	0	0	0	0	0	1	1	0	1	0	0	0	1	0	0	0	1	0	13
**1.004**	0	0	0	1	1	0	0	0	0	1	0	1	0	0	0	0	0	0	0	1	0	1	0	1	1	13
**1.005**	0	1	0	0	0	0	1	0	1	1	0	1	0	0	0	0	0	0	0	0	0	0	0	1	1	13
**1.006**	0	1	0	0	1	0	0	0	1	0	0	1	0	0	0	0	0	0	0	1	0	1	1	1	1	13
**1.007**	0	1	0	0	1	0	0	0	1	0	0	1	1	0	0	0	0	0	0	1	0	1	1	1	1	13
**1.008**	0	0	1	0	0	0	1	0	0	0	0	0	0	0	0	0	0	0	0	1	0	0	0	1	0	13
**1.009**	0	0	0	0	1	0	0	0	1	1	0	1	0	0	0	0	0	0	0	0	0	1	0	1	1	13
**1.010**	0	1	0	0	0	0	0	0	0	0	0	1	1	0	0	0	0	0	0	0	0	1	0	1	1	13
**1.011**	0	1	0	0	0	0	0	0	0	0	0	1	1	0	0	0	0	0	0	0	0	1	1	1	0	13
**1.012**	0	0	1	1	0	0	1	0	1	1	0	1	0	1	0	0	0	0	0	0	0	0	0	1	1	13
**1.013**	0	0	0	1	0	0	0	0	0	0	0	0	1	0	0	1	0	0	0	0	0	0	0	1	0	13
**1.014**	0	0	0	1	0	0	1	0	1	1	0	1	1	1	0	0	0	0	0	1	0	0	0	1	1	13
**1.015**	0	1	1	0	0	0	0	0	0	0	0	1	0	0	0	0	0	0	0	0	0	0	0	1	1	13
**1.016**	0	0	1	0	0	0	1	0	1	1	1	1	1	1	0	1	0	0	0	1	0	0	0	1	1	13
**1.017**	0	0	1	0	0	0	0	0	1	1	0	1	0	1	0	0	0	0	0	1	0	0	0	1	1	13
**1.018**	0	1	0	0	0	0	0	0	0	1	0	1	0	1	0	0	0	0	0	0	0	0	0	1	1	13
**1.019**	0	0	0	0	0	0	0	0	0	1	0	1	0	0	0	1	0	0	0	0	0	0	0	1	1	13
**1.020**	0	0	0	0	0	0	0	0	1	1	0	1	0	1	0	0	0	0	0	0	0	0	0	1	0	13
**1.021**	0	0	0	0	0	0	1	0	1	0	0	0	0	0	0	0	0	0	0	0	0	1	0	1	1	13
**1.022**	0	1	0	0	1	0	0	0	1	1	0	1	1	0	0	0	0	0	0	1	0	1	0	1	1	13
**1.023**	0	0	0	0	1	0	0	0	0	1	0	1	1	0	0	0	0	0	0	1	0	0	0	1	1	13
**1.024**	0	0	0	0	0	0	0	0	0	1	0	0	1	0	0	0	0	0	0	1	0	0	0	1	0	13
**1.025**	0	0	0	0	0	0	0	0	0	0	0	0	0	1	0	0	0	0	0	0	0	1	1	1	1	13
**1.026**	0	0	0	0	1	0	0	0	0	1	0	0	0	0	0	0	0	0	0	0	0	0	0	1	1	13
**1.027**	0	1	0	1	0	0	0	0	1	1	0	1	0	0	0	0	0	0	0	0	0	0	0	1	0	13
**1.028**	0	0	0	0	0	0	0	0	0	1	1	1	0	0	0	0	0	0	0	0	0	1	0	1	1	13
**1.029**	0	0	0	0	0	0	0	0	1	1	0	0	0	0	0	0	0	0	0	1	0	1	0	1	1	13
**1.030**	0	0	1	0	0	0	1	0	0	1	0	1	0	0	0	0	0	0	0	0	0	0	0	1	0	13
**1.031**	0	0	0	0	0	0	1	0	0	0	0	0	0	1	0	0	0	0	0	0	0	0	1	1	0	13
**1.032**	0	0	0	0	1	0	0	0	1	0	0	1	0	0	0	0	0	0	0	0	0	0	1	1	0	13
**1.033**	0	1	0	0	0	0	0	0	0	0	1	1	0	0	0	0	0	0	0	0	0	0	0	1	0	13
**1.034**	0	0	1	0	0	0	0	0	1	1	0	1	0	0	0	0	0	0	0	1	0	1	0	1	0	13
**1.035**	0	0	0	0	0	0	1	0	1	0	0	0	0	0	0	0	0	0	0	0	0	0	1	1	1	13
**1.036**	0	0	0	0	0	0	0	0	0	1	1	1	0	0	0	0	0	0	0	1	0	0	0	1	0	13
**1.037**	0	0	0	0	0	0	1	0	0	1	0	0	0	0	0	0	0	0	0	0	0	0	1	1	0	13
**1.038**	0	0	1	0	0	0	1	0	0	1	0	1	0	0	0	0	0	0	0	1	0	0	1	1	1	12
**1.039**	0	0	0	1	1	0	0	0	0	1	0	1	1	0	0	0	0	0	0	0	0	0	0	1	1	12
**1.040**	0	0	0	0	0	0	0	0	0	1	0	0	1	0	0	0	0	0	0	0	0	0	0	1	1	12
**1.041**	0	0	1	0	0	0	0	0	0	0	0	1	1	0	0	0	0	0	0	0	0	0	0	1	1	12
**1.042**	0	0	1	0	0	0	1	0	0	1	0	0	0	0	0	0	0	0	0	0	0	0	1	1	0	12
**1.043**	0	0	0	0	0	0	0	0	1	0	1	1	0	0	0	0	0	0	0	0	0	0	0	1	1	12
**1.044**	0	1	0	1	1	0	0	0	0	0	0	1	0	0	0	0	0	0	0	0	0	1	0	1	1	12
**1.045**	0	0	1	0	1	0	0	0	0	0	0	0	1	0	0	0	0	0	0	1	0	1	0	1	0	12
**1.046**	0	0	1	0	0	0	0	0	1	1	0	0	0	0	0	0	0	0	0	1	0	1	0	1	1	12
**1.047**	0	0	1	0	0	0	1	0	0	1	0	1	0	0	0	0	0	0	0	0	0	0	1	1	1	12
**1.048**	0	1	1	1	1	0	0	0	0	1	0	1	1	1	0	0	0	0	0	1	0	0	0	1	1	12
**1.049**	0	0	1	0	0	0	0	0	0	0	0	0	0	1	0	1	0	0	0	0	0	1	0	1	0	12
**1.050**	0	0	1	0	0	0	0	0	0	1	0	1	1	1	0	0	0	0	0	0	0	0	0	1	1	12
**1.051**	0	1	0	0	0	0	0	0	0	0	1	1	0	0	0	0	0	0	0	0	0	0	0	1	1	12
**1.052**	0	1	0	0	1	0	0	0	1	1	0	1	1	0	0	0	0	0	0	0	0	0	0	1	0	12
**1.053**	0	0	0	0	0	0	1	0	1	0	0	0	0	0	0	0	0	0	0	1	0	1	0	1	1	12
**1.054**	0	0	0	1	0	0	0	0	1	0	0	1	0	1	0	0	0	0	0	1	0	1	0	1	1	12
**1.055**	0	0	0	1	0	0	0	0	0	0	0	0	1	0	0	0	0	0	0	0	0	0	0	1	1	12
**1.056**	0	0	0	0	0	0	1	0	0	1	0	1	0	1	0	0	0	0	0	0	0	1	1	1	1	12
**1.057**	0	1	0	1	0	0	0	0	0	0	0	1	0	1	0	0	0	0	0	0	0	0	0	1	1	12
**1.058**	0	1	1	1	1	0	0	0	0	1	0	1	0	0	0	0	0	0	0	1	0	0	0	1	0	12
**1.059**	0	1	0	0	1	0	0	0	0	1	1	1	0	1	0	0	0	0	0	0	0	0	1	1	0	12
**1.060**	0	0	0	0	0	0	0	0	0	0	0	0	1	0	0	0	0	0	0	0	0	1	0	1	1	12
**1.061**	0	0	1	0	0	0	0	0	0	0	0	0	0	0	0	0	0	0	0	1	0	0	1	1	1	12
**1.062**	0	1	0	0	1	0	0	0	1	0	0	1	0	0	0	0	0	0	0	1	0	0	1	1	1	12
**1.063**	0	1	1	0	1	0	0	0	1	0	0	1	0	0	0	0	0	0	0	1	0	1	0	1	1	12
**1.064**	0	1	0	1	1	0	0	0	1	1	0	1	0	0	0	0	0	0	0	0	0	1	0	1	1	12
**1.065**	0	1	0	1	0	0	1	0	0	0	0	1	0	0	0	0	0	0	0	0	0	0	0	1	1	12
**1.066**	0	1	1	1	0	0	0	0	0	1	0	1	0	0	0	0	0	0	0	1	0	0	0	1	1	12
**1.067**	0	0	0	0	0	0	0	0	0	1	0	0	0	1	0	1	0	0	0	0	0	0	0	1	0	12
**1.068**	0	1	0	1	1	0	0	0	1	0	0	1	0	0	0	0	0	0	0	0	0	0	1	1	0	12
**1.069**	0	0	0	0	0	0	1	0	0	0	0	1	0	1	0	0	0	0	0	0	0	1	0	1	0	12
**1.070**	0	1	0	1	0	0	0	0	1	0	0	1	0	1	0	0	0	0	0	1	0	1	0	1	1	12
**1.071**	0	0	0	0	0	0	1	0	1	0	0	1	0	0	0	0	0	0	0	0	0	0	1	1	1	12
**1.072**	0	1	1	0	0	0	0	0	0	1	0	1	0	0	0	0	0	0	0	1	0	0	0	1	0	12
**1.073**	0	0	0	0	0	0	0	0	0	0	0	0	0	1	0	1	0	0	0	0	0	0	0	1	0	12
**1.074**	0	0	0	0	0	0	0	0	1	0	0	0	0	0	0	1	0	0	0	0	0	0	1	1	1	12
**1.075**	0	1	0	0	0	0	0	0	1	0	0	0	0	0	0	0	0	0	0	0	0	0	1	1	0	12
**1.076**	0	0	1	0	0	0	1	0	0	1	1	1	1	1	0	0	0	0	0	0	0	0	0	1	1	12
**1.077**	0	0	0	0	0	0	0	0	1	0	0	1	0	0	0	0	0	0	0	0	0	1	1	1	0	12

	CD2	CD3	CD4	CD7	CD8	CD10	CD13	CD18	CD26	CD29	CD36	CD44	CD45RA	CD49f	CD54	CD56	CD57	CD58	CD62L	CD71	CD80	Cytokeratin	Histone_H1	HLA-DQ	HLA-DR	Freq. (Voxel)
**1.078**	0	0	0	1	1	0	0	0	0	0	0	1	0	0	0	0	0	0	0	0	0	0	0	1	0	12
**1.079**	0	0	1	1	0	0	1	0	0	1	0	1	0	1	0	0	0	0	0	1	0	1	0	1	1	12
**1.080**	0	1	0	1	0	0	0	0	0	0	0	0	0	0	0	0	0	0	0	0	0	0	1	1	1	11
**1.081**	0	0	0	0	0	0	1	0	1	1	0	0	0	0	0	0	0	0	0	0	0	0	0	1	0	11
**1.082**	0	1	0	1	1	0	0	0	0	0	0	1	1	1	0	0	0	0	0	1	0	0	0	1	1	11
**1.083**	0	1	1	1	1	0	1	0	0	0	0	1	0	0	0	0	0	0	0	1	0	0	0	1	1	11
**1.084**	0	1	0	0	0	0	0	0	0	0	0	0	0	1	0	0	0	0	0	1	0	1	0	1	1	11
**1.085**	0	0	1	0	0	0	0	0	0	0	1	0	0	0	0	0	0	0	0	0	0	1	0	1	1	11
**1.086**	0	1	0	0	1	0	0	0	0	1	0	0	0	0	0	0	0	0	0	0	0	0	1	1	1	11
**1.087**	0	0	0	0	1	0	0	0	0	0	0	1	0	0	0	0	0	0	0	1	0	1	1	1	1	11
**1.088**	0	1	0	0	1	0	0	0	0	1	0	1	0	0	0	0	0	0	0	1	0	1	0	1	1	11
**1.089**	0	0	0	0	1	0	0	0	1	1	0	1	0	1	0	0	0	0	0	1	0	0	0	1	0	11
**1.090**	0	1	1	0	0	0	0	0	0	0	0	1	0	0	0	0	0	0	0	0	0	1	1	1	1	11
**1.091**	0	1	1	0	1	0	1	0	0	0	0	1	0	0	0	0	0	0	0	0	0	0	0	1	1	11
**1.092**	0	0	1	0	0	0	1	0	0	0	0	0	1	0	0	0	0	0	0	1	0	0	0	1	0	11
**1.093**	0	1	0	0	0	0	0	0	0	1	0	1	0	1	0	0	0	0	0	1	0	0	0	1	1	11
**1.094**	0	1	0	0	0	0	0	0	0	0	0	0	0	0	0	0	0	0	0	1	0	0	0	1	0	11
**1.095**	0	1	0	0	1	0	0	0	0	1	0	0	0	0	0	0	0	0	0	0	0	0	0	1	1	11
**1.096**	0	0	0	0	0	0	0	0	1	0	1	0	0	0	0	0	0	0	0	0	0	1	0	1	0	11
**1.097**	0	1	0	0	1	0	0	0	0	1	0	1	0	1	0	0	0	0	0	1	0	0	0	1	1	11
**1.098**	0	1	1	0	1	0	0	0	1	1	0	1	0	0	0	0	0	0	0	0	0	0	0	1	0	11
**1.099**	0	0	0	1	1	0	0	0	0	0	0	1	1	0	0	0	0	0	0	0	0	1	0	1	1	11
**1.100**	0	0	0	0	1	0	0	0	0	1	0	1	0	1	0	0	0	0	0	1	0	1	0	1	0	11
**1.101**	0	0	0	1	0	0	0	0	0	0	0	1	0	1	0	0	0	0	0	0	0	1	0	1	1	11
**1.102**	0	1	1	0	0	0	0	0	0	0	0	1	0	0	0	0	0	0	0	1	0	0	0	1	1	11
**1.103**	0	0	0	0	0	0	0	0	0	0	0	0	1	1	0	0	0	0	0	0	0	0	0	1	0	11
**1.104**	0	1	0	1	1	0	0	0	0	0	0	1	1	0	0	0	0	0	0	1	0	1	0	1	0	11
**1.105**	0	0	1	1	0	0	1	0	0	1	0	1	1	1	0	0	0	0	0	0	0	0	0	1	1	11
**1.106**	0	0	0	0	0	0	1	0	1	1	0	0	0	1	0	0	0	0	0	1	0	0	0	1	1	11
**1.107**	0	0	0	0	0	0	0	0	1	0	0	0	1	0	0	0	0	0	0	0	0	0	0	1	1	11
**1.108**	0	1	0	0	0	0	0	0	1	0	0	1	0	1	0	0	0	0	0	1	0	1	0	1	1	11
**1.109**	0	1	0	1	1	0	0	0	0	1	0	1	1	0	0	0	0	0	0	1	0	1	0	1	0	11
**1.110**	0	0	1	1	0	0	0	0	0	0	0	0	1	0	0	0	0	0	0	1	0	0	0	1	0	11
**1.111**	0	1	1	1	1	0	0	0	1	1	0	1	1	1	0	0	0	0	0	1	0	0	0	1	0	11
**1.112**	0	1	0	1	0	0	0	0	0	0	0	1	0	0	0	0	0	0	0	1	0	1	1	1	1	11
**1.113**	0	0	0	0	0	0	1	0	1	0	0	0	0	1	0	0	0	0	0	0	0	0	1	1	1	11
**1.114**	0	1	0	1	0	0	0	0	0	0	0	1	0	0	0	0	0	0	0	1	0	0	1	1	1	11
**1.115**	0	1	1	0	1	0	0	0	0	1	0	1	0	1	0	0	0	0	0	1	0	0	0	1	1	11
**1.116**	0	1	0	0	1	0	0	0	0	0	0	1	1	0	0	0	0	0	0	1	0	0	1	1	1	11
**1.117**	0	0	0	0	1	0	1	0	0	1	0	1	0	1	0	0	0	0	0	0	0	1	1	1	1	11
**1.118**	0	1	0	0	0	0	0	0	0	0	0	1	0	1	0	0	0	0	0	0	0	1	0	1	0	11
**1.119**	0	1	0	1	1	0	0	0	0	1	0	1	0	1	0	0	0	0	0	0	0	0	1	1	1	11
**1.120**	0	0	1	0	0	0	1	0	1	1	0	1	0	1	0	0	0	0	0	1	0	1	1	1	1	11
**1.121**	0	0	0	0	0	0	1	0	1	0	0	0	0	1	0	0	0	0	0	1	0	0	0	1	1	11
**1.122**	0	0	1	0	0	0	0	0	1	1	0	0	0	0	0	0	0	0	0	1	0	0	0	1	1	11
**1.123**	0	0	0	0	1	0	1	0	1	0	0	0	1	0	0	1	0	0	0	1	0	0	0	1	0	11
**1.124**	0	0	0	0	1	0	0	0	1	0	0	1	0	0	0	0	0	0	0	0	0	0	1	1	1	11
**1.125**	0	1	0	0	0	0	0	0	1	1	0	1	0	0	0	0	0	0	0	1	0	1	0	1	1	11
**1.126**	0	1	0	0	1	0	0	0	0	0	0	1	0	1	0	0	0	0	0	1	0	1	0	1	1	11
**1.127**	0	0	1	0	0	0	0	0	0	1	0	0	0	0	0	0	0	0	0	0	0	0	1	1	0	11
**1.128**	0	1	0	0	1	0	0	0	1	0	0	1	0	0	0	0	0	0	0	0	0	1	1	1	1	11
**1.129**	0	0	1	0	0	0	0	0	1	1	0	1	0	0	0	0	0	0	0	0	0	1	0	1	0	11
**1.130**	0	0	0	0	0	0	0	0	1	0	0	0	0	1	0	0	0	0	0	1	0	1	0	1	0	11
**1.131**	0	1	0	0	1	0	0	0	1	1	0	1	0	0	0	0	0	0	0	0	0	1	1	1	1	11
**1.132**	0	0	1	0	0	0	1	0	1	1	0	1	0	1	0	0	0	0	0	1	0	0	1	1	1	11
**1.133**	0	1	0	0	0	0	0	0	0	0	1	0	0	0	0	0	0	0	0	0	0	1	0	1	1	11
**1.134**	0	0	0	0	0	0	1	0	0	1	0	1	0	1	0	1	0	0	0	0	0	0	1	1	1	10
**1.135**	0	1	0	1	1	0	0	0	0	0	0	1	0	0	0	0	0	0	0	0	0	1	1	1	1	10
**1.136**	0	1	0	0	0	0	0	0	1	0	0	0	0	0	0	0	0	0	0	0	0	1	1	1	1	10
**1.137**	0	1	1	0	0	0	0	0	0	0	0	1	0	0	0	0	0	0	0	1	0	1	1	1	1	10
**1.138**	0	0	1	1	0	0	0	0	1	0	0	1	0	1	0	0	0	0	0	1	0	1	0	1	1	10
**1.139**	0	0	0	0	0	0	0	0	0	1	0	0	0	1	0	0	0	0	0	1	0	1	0	1	1	10
**1.140**	0	0	0	0	0	0	0	0	1	1	0	0	0	0	0	0	0	0	0	1	0	0	0	1	0	10
**1.141**	0	0	0	1	0	0	0	0	0	0	0	1	0	1	0	0	0	0	0	1	0	0	0	1	1	10
**1.142**	0	1	0	1	0	0	0	0	0	0	0	1	1	1	0	0	0	0	0	1	0	1	0	1	0	10
**1.143**	0	0	1	0	1	0	1	0	0	0	0	0	0	0	0	0	0	0	0	0	0	0	0	1	1	10
**1.144**	0	1	0	1	0	0	0	0	0	1	0	1	0	1	0	0	0	0	0	0	0	0	0	1	1	10
**1.145**	0	1	0	1	1	0	0	0	0	1	0	1	0	1	0	0	0	0	0	0	0	1	1	1	1	10
**1.146**	0	0	1	0	0	0	0	0	0	0	0	1	1	1	0	0	0	0	0	1	0	1	0	1	1	10
**1.147**	0	0	0	0	1	0	0	0	0	0	0	0	0	0	0	0	0	0	0	0	0	1	0	1	1	10
**1.148**	0	1	0	1	1	0	0	0	0	1	1	1	0	1	0	0	0	0	0	0	0	1	1	1	1	10
**1.149**	0	0	0	0	1	0	0	0	0	1	0	1	0	1	0	0	0	0	0	0	0	0	1	1	1	10
**1.150**	0	1	0	1	1	0	0	0	0	0	0	1	0	0	0	0	0	0	0	1	0	0	1	1	0	10
**1.151**	0	0	0	1	1	0	0	0	0	0	0	1	0	0	0	0	0	0	0	0	0	0	1	1	1	10
**1.152**	0	1	0	1	1	0	1	0	1	1	0	1	0	0	0	0	0	0	0	1	0	0	0	1	1	10
**1.153**	0	1	0	0	1	0	0	0	0	1	0	1	0	0	0	0	0	0	0	0	0	1	1	1	1	10
**1.154**	0	1	0	0	1	0	0	0	1	1	0	1	1	0	0	0	0	0	0	1	0	0	1	1	1	10
**1.155**	0	1	1	0	1	0	0	0	0	1	0	1	1	0	0	0	0	0	0	1	0	0	0	1	1	10
**1.156**	0	1	0	1	1	0	0	0	0	1	0	1	1	0	0	0	0	0	0	0	0	0	1	1	1	10
**1.157**	0	1	0	0	0	0	0	0	1	0	0	1	0	0	0	0	0	0	0	0	0	1	1	1	0	10
**1.158**	0	1	0	0	1	0	0	0	0	0	0	1	1	0	0	0	0	0	0	1	0	0	0	1	0	10
**1.159**	0	1	0	1	0	0	0	0	0	0	0	1	0	0	0	0	0	0	0	0	0	1	1	1	0	10
**1.160**	0	0	1	0	0	0	1	0	0	1	1	1	0	1	0	1	0	0	0	0	0	0	0	1	1	10
**1.161**	0	0	1	0	0	0	0	0	1	0	0	1	0	1	0	0	0	0	0	1	0	0	0	1	1	10
**1.162**	0	0	0	0	1	0	0	0	0	0	0	1	0	0	0	1	0	0	0	0	0	0	0	1	0	10
**1.163**	0	0	1	0	0	0	0	0	0	0	0	0	0	0	0	1	0	0	0	1	0	0	0	1	0	10
**1.164**	0	1	0	0	1	0	0	0	1	0	0	1	1	1	0	0	0	0	0	0	0	0	0	1	1	10
**1.165**	0	1	0	1	1	0	0	0	1	0	0	1	0	0	0	0	0	0	0	0	0	0	1	1	1	10
**1.166**	0	1	0	0	1	0	0	0	0	1	0	1	1	0	0	0	0	0	0	0	0	1	0	1	0	10
**1.167**	0	0	1	0	0	0	0	0	0	0	0	0	0	1	0	0	0	0	0	1	0	1	0	1	1	10
**1.168**	0	1	0	0	0	0	0	0	0	0	0	1	1	0	0	0	0	0	0	0	0	0	1	1	0	10
**1.169**	0	0	0	0	0	0	1	0	0	1	0	1	0	1	0	1	0	0	0	0	0	0	0	1	1	10
**1.170**	0	0	0	0	1	0	0	0	1	0	0	1	1	0	0	0	0	0	0	0	0	0	0	1	1	10
**1.171**	0	0	1	1	0	0	0	0	0	0	0	0	0	0	0	0	0	0	0	0	0	0	0	1	0	10
**1.172**	0	0	0	1	0	0	0	0	0	0	0	0	1	0	0	0	0	0	0	0	0	0	0	1	0	10
**1.173**	0	0	0	0	0	0	0	0	1	0	0	0	1	0	0	1	0	0	0	1	0	1	0	1	0	10
**1.174**	0	0	0	1	1	0	0	0	1	0	0	1	1	0	0	0	0	0	0	0	0	0	0	1	1	10
**1.175**	0	0	0	1	0	0	1	0	0	0	0	1	0	0	0	0	0	0	0	1	0	0	0	1	1	10

	CD2	CD3	CD4	CD7	CD8	CD10	CD13	CD18	CD26	CD29	CD36	CD44	CD45RA	CD49f	CD54	CD56	CD57	CD58	CD62L	CD71	CD80	Cytokeratin	Histone_H1	HLA-DQ	HLA-DR	Freq. (Voxel)
**1.176**	0	1	1	1	1	0	0	0	1	0	0	1	0	0	0	0	0	0	0	1	0	0	0	1	1	10
**1.177**	0	1	0	0	0	0	0	0	0	1	0	1	1	0	0	0	0	0	0	0	0	0	0	1	0	10
**1.178**	0	0	1	1	0	0	0	0	0	1	0	1	0	1	0	0	0	0	0	0	0	0	0	1	1	10
**1.179**	0	1	1	1	1	0	0	0	1	1	0	1	0	0	0	0	0	0	0	1	0	0	0	1	0	10
**1.180**	0	0	0	0	0	0	0	0	0	0	0	1	0	1	0	0	0	0	0	0	0	1	1	1	0	10
**1.181**	0	0	0	0	0	0	0	0	0	0	1	0	0	0	0	0	0	0	0	1	0	0	0	1	1	10
**1.182**	0	0	1	0	0	0	0	0	1	0	0	0	0	1	0	0	0	0	0	0	0	0	0	1	1	10
**1.183**	0	0	0	1	0	0	1	0	0	0	0	1	0	0	0	1	0	0	0	1	0	0	0	1	1	9
**1.184**	0	0	0	0	1	0	1	0	1	0	0	0	0	0	0	1	0	0	0	0	0	0	0	1	1	9
**1.185**	0	1	0	0	0	0	0	0	1	0	0	1	1	1	0	0	0	0	0	1	0	1	0	1	1	9
**1.186**	0	0	1	1	0	0	0	0	0	0	0	0	0	0	0	0	0	0	0	1	0	0	0	1	0	9
**1.187**	0	1	0	1	0	0	0	0	0	0	0	0	0	0	0	0	0	0	0	1	0	1	1	1	1	9
**1.188**	0	1	1	1	1	0	0	0	1	0	0	1	0	0	0	0	0	0	0	0	0	0	0	1	0	9
**1.189**	0	0	1	0	0	0	0	0	0	1	0	1	0	0	0	0	0	0	0	0	0	0	1	1	0	9
**1.190**	0	1	0	0	0	0	0	0	0	0	0	0	0	1	0	0	0	0	0	0	0	1	0	1	0	9
**1.191**	0	1	0	1	1	0	0	0	0	1	0	1	0	0	0	0	0	0	0	1	0	0	1	1	1	9
**1.192**	0	0	0	0	0	0	0	0	1	0	0	1	1	0	0	0	0	0	0	0	0	1	0	1	0	9
**1.193**	0	0	1	1	0	0	0	0	0	0	0	0	1	1	0	0	0	0	0	1	0	0	0	1	0	9
**1.194**	0	1	0	0	1	0	0	0	0	1	0	1	1	1	0	0	0	0	0	1	0	0	0	1	0	9
**1.195**	0	1	0	0	0	0	0	0	0	0	1	1	0	1	0	0	0	0	0	1	0	1	0	1	1	9
**1.196**	0	1	0	1	1	0	0	0	0	0	0	1	1	0	0	0	0	0	0	0	0	1	1	1	0	9
**1.197**	0	1	0	0	1	0	0	0	1	0	0	0	0	0	0	0	0	0	0	0	0	0	0	1	1	9
**1.198**	0	0	0	0	0	0	1	0	1	1	0	1	0	1	0	0	0	0	0	0	0	1	0	1	1	9
**1.199**	0	0	0	0	0	0	0	0	1	0	0	1	0	1	0	0	0	0	0	0	0	0	1	1	1	9
**1.200**	0	0	1	1	0	0	1	0	1	1	0	1	1	1	0	0	0	0	0	1	0	0	1	1	1	9
**1.201**	0	0	0	0	0	0	0	0	1	0	0	0	1	0	0	0	0	0	0	0	0	1	0	1	0	9
**1.202**	0	0	0	1	0	0	1	0	0	1	1	1	0	1	0	0	0	0	0	0	0	0	0	1	0	9
**1.203**	0	0	1	0	0	0	1	0	1	0	0	0	0	1	0	0	0	0	0	1	0	0	0	1	1	9
**1.204**	0	0	0	0	0	0	0	0	0	0	0	0	0	0	0	1	0	0	0	1	0	0	1	1	1	9
**1.205**	0	0	0	0	0	0	0	0	1	0	1	1	0	0	0	0	0	0	0	1	0	0	0	1	1	9
**1.206**	0	0	1	0	0	0	0	0	0	1	0	1	0	1	0	0	0	0	0	1	0	1	1	1	0	9
**1.207**	0	1	0	0	0	0	0	0	1	0	0	0	0	0	0	0	0	0	0	0	0	0	0	1	1	9
**1.208**	0	0	0	1	0	0	0	0	0	0	0	0	0	0	0	0	0	0	0	1	0	1	0	1	0	9
**1.209**	0	0	0	1	1	0	0	0	0	1	0	1	0	0	0	0	0	0	0	1	0	1	0	1	0	9
**1.210**	0	1	0	0	1	0	0	0	1	0	0	1	1	1	0	0	0	0	0	1	0	0	0	1	1	9
**1.211**	0	1	0	1	1	0	1	0	1	0	0	1	1	0	0	0	0	0	0	1	0	0	0	1	1	9
**1.212**	0	0	0	1	0	0	0	0	0	0	0	0	0	0	0	0	0	0	0	1	0	0	0	1	1	9
**1.213**	0	0	0	0	0	0	0	0	0	0	0	1	0	0	0	1	0	0	0	1	0	0	1	1	1	9
**1.214**	0	1	1	0	1	0	0	0	1	0	0	0	1	0	0	0	0	0	0	1	0	0	0	1	1	9
**1.215**	0	1	0	0	1	0	0	0	0	1	0	1	1	1	0	0	0	0	0	0	0	0	0	1	1	9
**1.216**	0	1	0	1	1	0	0	0	0	0	0	1	1	0	0	0	0	0	0	1	0	0	1	1	0	9
**1.217**	0	0	0	0	0	0	0	0	0	0	0	0	1	1	0	0	0	0	0	0	0	1	0	1	0	9
**1.218**	0	1	0	1	1	0	0	0	1	0	0	1	0	0	0	0	0	0	0	1	0	0	1	1	0	9
**1.219**	0	0	0	0	0	0	0	0	1	0	0	0	0	0	0	0	0	0	0	1	0	0	1	1	1	9
**1.220**	0	0	1	0	0	0	0	0	1	1	0	1	0	0	0	0	0	0	0	1	0	0	0	1	0	9
**1.221**	0	1	0	0	0	0	0	0	1	1	0	1	0	0	0	0	0	0	0	0	0	1	0	1	0	9
**1.222**	0	0	0	0	1	0	0	0	1	0	0	0	0	0	0	0	0	0	0	0	0	1	0	1	1	9
**1.223**	0	0	0	0	1	0	1	0	0	1	0	1	0	0	0	0	0	0	0	1	0	1	0	1	1	9
**1.224**	0	0	0	0	1	0	1	0	1	1	0	1	0	1	0	0	0	0	0	1	0	1	0	1	1	9
**1.225**	0	1	0	1	1	0	1	0	0	1	0	1	1	0	0	0	0	0	0	1	0	0	0	1	1	9
**1.226**	0	0	0	0	1	0	0	0	0	1	0	1	1	0	0	0	0	0	0	0	0	1	0	1	1	9
**1.227**	0	1	1	1	1	0	0	0	0	0	0	1	0	0	0	0	0	0	0	0	0	0	0	1	1	9
**1.228**	0	0	1	1	0	0	1	0	1	1	0	1	1	1	0	0	0	0	0	1	0	1	0	1	1	9
**1.229**	0	1	0	0	0	0	0	0	0	0	0	1	1	0	0	0	0	0	0	0	0	1	1	1	1	9
**1.230**	0	0	0	1	0	0	1	0	1	1	0	1	0	1	0	0	0	0	0	0	0	0	0	1	1	9
**1.231**	0	0	1	0	0	0	1	0	1	1	0	0	0	1	0	0	0	0	0	1	0	0	0	1	1	9
**1.232**	0	1	0	1	0	0	0	0	0	0	0	1	0	0	0	0	0	0	0	0	0	1	0	1	0	9
**1.233**	0	0	1	0	0	0	0	0	0	0	0	0	1	1	0	0	0	0	0	0	0	1	0	1	0	9
**1.234**	0	0	1	1	0	0	1	0	1	1	1	1	0	1	0	0	0	0	0	1	0	0	0	1	1	9
**1.235**	0	0	1	1	0	0	0	0	0	0	0	0	0	0	0	1	0	0	0	0	0	0	0	1	0	9
**1.236**	0	0	0	0	0	0	0	0	1	0	0	1	0	0	0	0	0	0	0	0	0	1	1	1	1	9
**1.237**	0	0	0	0	0	0	1	0	1	1	0	1	0	1	0	0	0	0	0	0	0	1	0	1	0	9
**1.238**	0	1	0	0	0	0	0	0	0	0	0	1	1	0	0	0	0	0	0	0	0	0	0	1	0	9
**1.239**	0	0	1	1	1	0	0	0	1	1	0	1	1	0	0	0	0	0	0	1	0	0	0	1	1	9
**1.240**	0	0	1	0	1	0	0	0	1	0	0	1	0	0	0	0	0	0	0	1	0	1	0	1	1	9
**1.241**	0	1	1	1	1	0	0	0	1	1	0	1	0	0	0	0	0	0	0	1	0	1	0	1	1	9
**1.242**	0	0	0	0	1	0	0	0	0	1	0	1	0	1	0	0	0	0	0	1	0	0	0	1	1	9
**1.243**	0	0	0	0	1	0	1	0	1	1	0	1	0	0	0	0	0	0	0	1	0	0	0	1	1	9
**1.244**	0	0	1	0	0	0	0	0	1	0	0	0	0	0	0	0	0	0	0	1	0	1	0	1	0	9
**1.245**	0	1	1	0	1	0	1	0	1	0	0	1	0	0	0	0	0	0	0	0	0	0	0	1	1	9
**1.246**	0	1	0	0	0	0	0	0	1	1	0	1	1	1	0	0	0	0	0	1	0	0	0	1	0	9
**1.247**	0	1	0	0	0	0	0	0	0	0	0	1	0	1	0	0	0	0	0	1	0	0	0	1	0	9
**1.248**	0	0	0	0	0	0	0	0	1	1	0	0	0	0	0	0	0	0	0	1	0	0	0	1	1	9
**1.249**	0	0	1	0	0	0	0	0	0	1	1	1	0	1	0	0	0	0	0	1	0	0	0	1	1	9
**1.250**	0	0	1	1	0	0	1	0	0	1	1	1	1	1	0	1	0	0	0	1	0	0	0	1	1	9
**1.251**	0	0	0	0	1	0	0	0	0	0	0	0	0	0	0	0	0	0	0	0	0	0	1	1	0	9
**1.252**	0	0	0	0	0	0	0	0	0	1	0	0	1	1	0	0	0	0	0	1	0	0	0	1	1	9
**1.253**	0	0	1	0	0	0	1	0	1	1	0	1	0	0	0	0	0	0	0	1	0	0	1	1	1	8
**1.254**	0	1	0	1	1	0	0	0	0	0	0	1	1	1	0	0	0	0	0	0	0	0	0	1	0	8
**1.255**	0	0	0	0	1	0	1	0	0	0	0	1	0	0	0	0	0	0	0	0	0	1	0	1	1	8
**1.256**	0	0	1	0	1	0	0	0	0	0	0	1	0	0	0	0	0	0	0	0	0	0	0	1	0	8
**1.257**	0	0	1	1	0	0	1	0	0	1	1	1	0	1	0	0	0	0	0	0	0	0	0	1	1	8
**1.258**	0	0	1	0	0	0	0	0	1	1	0	1	0	1	0	0	0	0	0	1	0	1	1	1	1	8
**1.259**	0	1	1	0	1	0	1	0	1	1	0	1	1	0	0	0	0	0	0	1	0	1	0	1	1	8
**1.260**	0	0	0	1	0	0	0	0	0	0	0	0	1	0	0	1	0	0	0	0	0	1	0	1	0	8
**1.261**	0	0	0	1	1	0	1	0	1	0	0	1	0	0	0	1	0	0	0	1	0	0	0	1	1	8
**1.262**	0	0	0	1	1	0	1	0	1	1	0	1	0	0	0	0	0	0	0	1	0	1	0	1	1	8
**1.263**	0	0	1	0	0	0	0	0	1	0	0	0	1	0	0	0	0	0	0	1	0	1	0	1	1	8
**1.264**	0	1	0	0	1	0	0	0	1	0	0	1	1	1	0	0	0	0	0	1	0	1	0	1	1	8
**1.265**	0	1	0	1	1	0	0	0	1	0	0	1	1	1	0	0	0	0	0	1	0	0	0	1	1	8
**1.266**	0	1	0	1	1	0	0	0	0	0	0	0	0	0	0	0	0	0	0	1	0	0	1	1	1	8
**1.267**	0	0	0	0	0	0	0	0	0	0	0	1	1	0	0	0	0	0	0	1	0	0	0	1	1	8
**1.268**	0	0	0	0	0	0	1	0	0	1	0	0	1	1	0	0	0	0	0	0	0	0	0	1	0	8
**1.269**	0	0	0	0	0	0	1	0	0	1	0	0	1	1	0	0	0	0	0	0	0	0	1	1	0	8
**1.270**	0	0	1	0	0	0	1	0	0	0	0	0	0	0	0	1	0	0	0	1	0	0	0	1	1	8
**1.271**	0	0	0	1	1	0	0	0	0	1	0	1	0	1	0	0	0	0	0	0	0	1	0	1	1	8
**1.272**	0	0	0	1	0	0	1	0	1	1	0	1	1	1	0	0	0	0	0	1	0	0	1	1	1	8
**1.273**	0	0	0	0	0	0	0	0	1	1	1	1	0	0	0	0	0	0	0	1	0	0	0	1	1	8

	CD2	CD3	CD4	CD7	CD8	CD10	CD13	CD18	CD26	CD29	CD36	CD44	CD45RA	CD49f	CD54	CD56	CD57	CD58	CD62L	CD71	CD80	Cytokeratin	Histone_H1	HLA-DQ	HLA-DR	Freq. (Voxel)
**1.274**	0	0	0	1	0	0	1	0	0	0	0	0	1	0	0	1	0	0	0	0	0	1	0	1	0	8
**1.275**	0	1	0	0	1	0	0	0	1	1	0	1	1	0	0	0	0	0	0	0	0	1	1	1	0	8
**1.276**	0	1	0	1	0	0	0	0	1	0	0	1	0	0	0	0	0	0	0	1	0	1	1	1	1	8
**1.277**	0	1	1	1	1	0	0	0	1	0	0	1	1	0	0	0	0	0	0	1	0	1	0	1	1	8
**1.278**	0	0	0	0	0	0	0	0	1	0	0	1	1	0	0	0	0	0	0	1	0	0	0	1	1	8
**1.279**	0	0	1	0	0	0	0	0	0	1	0	0	0	1	0	0	0	0	0	0	0	1	0	1	0	8
**1.280**	0	0	1	0	1	0	1	0	0	1	0	0	0	0	0	0	0	0	0	0	0	0	0	1	1	8
**1.281**	0	1	0	1	1	0	1	0	0	1	0	1	0	0	0	0	0	0	0	0	0	0	0	1	0	8
**1.282**	0	1	1	0	0	0	1	0	1	0	0	1	0	0	0	0	0	0	0	1	0	0	0	1	1	8
**1.283**	0	1	0	0	0	0	0	0	0	1	1	1	0	0	0	0	0	0	0	0	0	0	0	1	1	8
**1.284**	0	0	1	0	0	0	1	0	1	1	0	1	1	1	0	0	0	0	0	1	0	0	1	1	1	8
**1.285**	0	0	0	1	0	0	1	0	0	1	1	1	0	1	0	0	0	0	0	0	0	0	0	1	1	8
**1.286**	0	0	0	0	0	0	1	0	0	1	0	1	0	1	0	1	0	0	0	1	0	0	0	1	1	8
**1.287**	0	0	1	0	0	0	1	0	1	1	1	1	0	1	0	0	0	0	0	0	0	0	0	1	1	8
**1.288**	0	0	0	0	0	0	1	0	1	0	0	0	0	0	0	0	0	0	0	1	0	0	0	1	0	8
**1.289**	0	0	0	0	1	0	1	0	0	1	0	1	0	0	0	0	0	0	0	0	0	0	0	1	0	8
**1.290**	0	0	0	0	0	0	0	0	0	0	0	0	0	0	0	1	0	0	0	1	0	1	0	1	0	8
**1.291**	0	0	0	0	0	0	1	0	0	1	0	0	0	0	0	0	0	0	0	1	0	0	0	1	0	8
**1.292**	0	0	0	0	1	0	0	0	1	1	0	1	0	1	0	0	0	0	0	1	0	0	0	1	1	8
**1.293**	0	0	1	0	1	0	0	0	0	0	0	1	0	0	0	0	0	0	0	1	0	1	0	1	1	8
**1.294**	0	0	0	0	0	0	0	0	0	0	0	1	1	1	0	0	0	0	0	0	0	1	0	1	1	8
**1.295**	0	0	0	0	0	0	1	0	0	1	0	1	0	0	0	0	0	0	0	1	0	1	0	1	1	8
**1.296**	0	0	0	0	1	0	0	0	0	1	0	1	1	0	0	0	0	0	0	1	0	0	0	1	0	8
**1.297**	0	0	1	0	1	0	0	0	1	1	0	1	0	0	0	0	0	0	0	0	0	0	0	1	1	8
**1.298**	0	1	1	0	0	0	0	0	0	0	0	0	0	0	0	0	0	0	0	0	0	0	0	1	1	8
**1.299**	0	0	1	0	1	0	1	0	0	0	0	0	0	0	0	0	0	0	0	1	0	0	0	1	1	8
**1.300**	0	1	0	0	0	0	0	0	0	0	0	0	0	0	0	0	0	0	0	1	0	0	0	1	1	8
**1.301**	0	1	0	1	1	0	1	0	0	0	0	1	0	0	0	0	0	0	0	0	0	0	0	1	0	8
**1.302**	0	1	1	1	1	0	0	0	0	1	0	1	0	1	0	0	0	0	0	1	0	0	0	1	0	8
**1.303**	0	1	0	0	1	0	0	0	0	0	0	0	1	0	0	0	0	0	0	0	0	0	0	1	0	8
**1.304**	0	1	1	1	0	0	0	0	1	1	0	1	0	0	0	0	0	0	0	0	0	0	0	1	0	8
**1.305**	0	1	1	1	1	0	0	0	0	1	0	1	0	0	0	0	0	0	0	0	0	0	0	1	0	8
**1.306**	0	0	0	0	0	0	0	0	1	0	0	1	0	1	0	0	0	0	0	0	0	0	0	1	0	8
**1.307**	0	0	0	1	0	0	0	0	0	1	0	1	0	1	0	0	0	0	0	1	0	0	0	1	0	8
**1.308**	0	0	0	0	0	0	0	0	1	1	0	1	0	1	0	0	0	0	0	0	0	0	1	1	1	8
**1.309**	0	0	1	0	0	0	0	0	0	1	0	1	0	1	0	1	0	0	0	0	0	1	0	1	0	8
**1.310**	0	1	0	0	0	0	0	0	1	0	0	0	0	0	0	0	0	0	0	0	0	0	0	1	0	8
**1.311**	0	0	0	0	0	0	0	0	0	1	1	0	0	0	0	0	0	0	0	0	0	1	0	1	0	8
**1.312**	0	0	0	1	0	0	0	0	1	0	0	0	0	0	0	0	0	0	0	1	0	1	0	1	1	8
**1.313**	0	0	1	0	0	0	1	0	0	0	0	1	0	0	0	0	0	0	0	0	0	1	0	1	1	8
**1.314**	0	0	0	1	1	0	0	0	1	1	0	1	0	0	0	0	0	0	0	0	0	0	0	1	0	8
**1.315**	0	0	0	0	0	0	0	0	1	0	1	1	0	0	0	0	0	0	0	0	0	1	0	1	1	8
**1.316**	0	1	0	0	1	0	0	0	1	1	0	1	1	1	0	0	0	0	0	1	0	0	0	1	0	8
**1.317**	0	1	0	0	0	0	0	0	0	0	0	0	0	0	0	0	0	0	0	1	0	1	1	1	0	8
**1.318**	0	0	1	1	0	0	1	0	1	1	0	1	1	1	0	0	0	0	0	0	0	0	0	1	1	8
**1.319**	0	1	0	0	1	0	0	0	1	0	0	1	1	0	0	0	0	0	0	0	0	0	1	1	1	8
**1.320**	0	0	0	1	0	0	0	0	0	0	0	0	0	0	0	0	0	0	0	0	0	1	0	1	0	8
**1.321**	0	0	1	0	0	0	0	0	1	1	0	1	0	1	0	0	0	0	0	0	0	0	1	1	1	8
**1.322**	0	0	1	0	0	0	1	0	0	1	1	1	0	1	0	0	0	0	0	0	0	0	0	1	0	8
**1.323**	0	0	1	1	0	0	1	0	0	1	0	0	1	1	0	0	0	0	0	1	0	0	0	1	1	8
**1.324**	0	0	0	0	0	0	0	0	1	0	0	0	1	0	0	0	0	0	0	1	0	1	0	1	0	8
**1.325**	0	0	1	0	0	0	0	0	0	1	1	0	0	0	0	0	0	0	0	0	0	0	0	1	1	8
**1.326**	0	0	1	0	0	0	0	0	0	0	0	0	1	1	0	1	0	0	0	0	0	0	0	1	0	8
**1.327**	0	1	0	0	0	0	0	0	1	0	0	1	1	0	0	0	0	0	0	1	0	1	1	1	1	7
**1.328**	0	0	0	0	0	0	0	0	0	1	0	1	0	0	0	1	0	0	0	1	0	0	0	1	1	7
**1.329**	0	1	0	0	1	0	0	0	1	1	0	1	0	1	0	0	0	0	0	1	0	0	0	1	1	7
**1.330**	0	0	0	1	1	0	0	0	1	0	0	1	0	0	0	0	0	0	0	0	0	0	0	1	0	7
**1.331**	0	1	0	0	1	0	0	0	1	0	0	1	1	1	0	0	0	0	0	0	0	1	0	1	1	7
**1.332**	0	1	0	1	1	0	1	0	0	0	0	1	0	0	0	0	0	0	0	1	0	0	1	1	1	7
**1.333**	0	1	0	0	0	0	0	0	0	0	0	0	0	1	0	0	0	0	0	1	0	0	0	1	1	7
**1.334**	0	0	1	0	0	0	0	0	0	0	0	1	1	0	0	0	0	0	0	0	0	0	0	1	0	7
**1.335**	0	0	1	0	1	0	0	0	0	1	0	1	0	0	0	0	0	0	0	0	0	0	0	1	0	7
**1.336**	0	1	0	0	1	0	1	0	0	0	0	1	0	0	0	0	0	0	0	0	0	0	0	1	0	7
**1.337**	0	0	1	1	0	0	0	0	1	0	0	1	0	0	0	0	0	0	0	0	0	1	0	1	1	7
**1.338**	0	1	0	1	0	0	0	0	1	0	0	1	0	0	0	0	0	0	0	0	0	1	1	1	1	7
**1.339**	0	1	1	0	1	0	1	0	1	0	0	1	0	0	0	0	0	0	0	1	0	0	0	1	1	7
**1.340**	0	1	1	1	1	0	1	0	0	0	0	1	0	0	0	0	0	0	0	0	0	0	0	1	1	7
**1.341**	0	0	1	0	1	0	1	0	1	0	0	0	0	0	0	0	0	0	0	1	0	0	0	1	1	7
**1.342**	0	0	1	1	0	0	0	0	0	0	0	1	0	0	0	0	0	0	0	1	0	0	0	1	0	7
**1.343**	0	1	0	0	0	0	0	0	0	0	0	0	0	1	0	0	0	0	0	0	0	1	1	1	1	7
**1.344**	0	1	1	0	1	0	1	0	0	0	0	0	0	0	0	0	0	0	0	0	0	0	0	1	1	7
**1.345**	0	0	0	1	0	0	0	0	0	0	0	1	0	1	0	0	0	0	0	0	0	1	0	1	0	7
**1.346**	0	1	0	1	1	0	0	0	0	1	0	1	1	1	0	0	0	0	0	1	0	0	0	1	1	7
**1.347**	0	1	0	1	0	0	0	0	0	1	0	1	0	1	0	0	0	0	0	0	0	0	0	1	0	7
**1.348**	0	0	1	0	0	0	0	0	0	0	0	0	1	1	0	0	0	0	0	0	0	0	0	1	1	7
**1.349**	0	1	1	0	1	0	0	0	1	1	0	1	1	0	0	0	0	0	0	0	0	0	0	1	1	7
**1.350**	0	0	1	0	0	0	0	0	0	1	0	0	0	1	0	1	0	0	0	0	0	0	0	1	0	7
**1.351**	0	1	0	0	0	0	0	0	1	1	1	1	0	0	0	0	0	0	0	0	0	0	0	1	1	7
**1.352**	0	1	0	1	1	0	0	0	0	0	0	0	0	0	0	0	0	0	0	0	0	0	1	1	0	7
**1.353**	0	0	0	0	0	0	0	0	0	0	1	1	0	0	0	1	0	0	0	0	0	0	0	1	1	7
**1.354**	0	0	1	0	0	0	0	0	0	0	0	0	0	1	0	0	0	0	0	0	0	0	1	1	1	7
**1.355**	0	0	1	0	0	0	0	0	0	0	0	1	1	1	0	0	0	0	0	0	0	1	0	1	0	7
**1.356**	0	1	0	0	1	0	0	0	0	0	0	0	0	0	0	0	0	0	0	0	0	0	0	1	0	7
**1.357**	0	0	0	0	0	0	0	0	1	0	1	1	0	0	0	0	0	0	0	0	0	1	1	1	1	7
**1.358**	0	0	1	1	0	0	0	0	0	1	0	0	0	0	0	0	0	0	0	1	0	0	0	1	1	7
**1.359**	0	0	0	1	0	0	0	0	1	1	0	1	0	0	0	0	0	0	0	1	0	0	0	1	0	7
**1.360**	0	1	0	0	1	0	0	0	1	0	0	1	0	0	0	0	0	0	0	0	0	1	1	1	0	7
**1.361**	0	0	0	0	0	0	0	0	1	0	0	0	0	1	0	0	0	0	0	1	0	1	0	1	1	7
**1.362**	0	0	0	1	0	0	1	0	1	0	0	1	0	0	0	0	0	0	0	0	0	0	0	1	1	7
**1.363**	0	0	0	0	0	0	0	0	0	1	0	0	1	1	0	1	0	0	0	0	0	0	0	1	0	7
**1.364**	0	0	0	1	1	0	0	0	0	0	0	1	0	0	0	0	0	0	0	1	0	0	0	1	0	7
**1.365**	0	0	1	0	0	0	0	0	0	0	0	0	1	1	0	0	0	0	0	1	0	0	0	1	1	7
**1.366**	0	0	1	0	0	0	0	0	0	0	0	1	0	1	0	0	0	0	0	0	0	0	1	1	1	7
**1.367**	0	0	0	1	0	0	0	0	0	0	0	1	0	1	0	0	0	0	0	0	0	0	0	1	0	7
**1.368**	0	0	1	0	0	0	0	0	0	1	0	0	0	0	0	0	0	0	0	1	0	1	0	1	1	7
**1.369**	0	1	0	0	1	0	0	0	0	1	0	1	0	1	0	0	0	0	0	1	0	1	0	1	1	7
**1.370**	0	1	0	0	1	0	0	0	0	1	0	1	0	0	0	0	0	0	0	0	0	1	1	1	0	7
**1.371**	0	1	0	1	0	0	1	0	0	0	0	1	0	0	0	0	0	0	0	1	0	0	0	1	1	7

	CD2	CD3	CD4	CD7	CD8	CD10	CD13	CD18	CD26	CD29	CD36	CD44	CD45RA	CD49f	CD54	CD56	CD57	CD58	CD62L	CD71	CD80	Cytokeratin	Histone_H1	HLA-DQ	HLA-DR	Freq. (Voxel)
**1.372**	0	0	0	0	0	0	1	0	0	0	0	0	1	0	0	0	0	0	0	0	0	0	1	1	0	7
**1.373**	0	0	0	0	0	0	1	0	0	1	0	0	0	1	0	0	0	0	0	0	0	0	1	1	0	7
**1.374**	0	0	0	1	0	0	0	0	0	1	0	1	0	0	0	0	0	0	0	0	0	1	0	1	0	7
**1.375**	0	0	1	0	0	0	1	0	1	1	0	1	0	1	0	0	0	0	0	0	0	1	0	1	1	7
**1.376**	0	0	0	0	1	0	1	0	1	1	0	1	0	0	0	0	0	0	0	0	0	0	0	1	1	7
**1.377**	0	0	0	1	0	0	0	0	0	1	0	0	0	0	0	0	0	0	0	0	0	0	0	1	0	7
**1.378**	0	0	1	0	0	0	1	0	1	1	0	1	1	1	0	1	0	0	0	1	0	0	0	1	1	7
**1.379**	0	0	0	0	0	0	1	0	1	0	0	0	0	0	0	0	0	0	0	0	0	0	1	1	0	7
**1.380**	0	0	0	0	0	0	1	0	1	1	1	1	0	1	0	0	0	0	0	1	0	0	0	1	1	7
**1.381**	0	0	1	0	0	0	1	0	1	1	0	1	0	1	0	0	0	0	0	0	0	0	1	1	1	7
**1.382**	0	1	0	0	0	0	0	0	0	1	0	1	0	1	0	0	0	0	0	0	0	0	1	1	1	7
**1.383**	0	0	0	0	0	0	0	0	1	1	0	0	0	0	0	0	0	0	0	0	0	0	0	1	1	7
**1.384**	0	1	0	0	0	0	0	0	0	1	0	1	0	0	0	0	0	0	0	0	0	0	1	1	1	7
**1.385**	0	1	0	1	1	0	0	0	1	1	0	1	0	0	0	0	0	0	0	1	0	0	1	1	0	7
**1.386**	0	0	0	1	0	0	0	0	0	0	0	0	1	0	0	1	0	0	0	1	0	1	0	1	0	7
**1.387**	0	0	0	0	0	0	0	0	0	1	1	0	0	0	0	0	0	0	0	1	0	1	0	1	0	7
**1.388**	0	1	0	1	0	0	0	0	1	0	0	1	0	0	0	0	0	0	0	1	0	0	0	1	0	7
**1.389**	0	0	0	0	0	0	1	0	0	0	0	0	0	0	0	0	0	0	0	1	0	0	1	1	0	7
**1.390**	0	0	0	0	1	0	1	0	1	0	0	1	0	0	0	0	0	0	0	0	0	1	0	1	1	7
**1.391**	0	0	0	0	1	0	0	0	1	1	0	1	1	0	0	0	0	0	0	1	0	0	0	1	0	7
**1.392**	0	1	0	1	1	0	1	0	1	1	0	1	1	0	0	0	0	0	0	1	0	1	0	1	1	7
**1.393**	0	1	1	1	1	0	1	0	1	0	0	1	1	0	0	0	0	0	0	1	0	1	0	1	1	7
**1.394**	0	0	0	0	1	0	0	0	1	0	0	1	1	0	0	0	0	0	0	0	0	1	0	1	0	7
**1.395**	0	0	0	0	1	0	0	0	1	1	0	1	0	0	0	0	0	0	0	1	0	0	0	1	0	7
**1.396**	0	0	0	1	0	0	0	0	0	0	0	1	0	0	0	0	0	0	0	1	0	1	0	1	0	7
**1.397**	0	0	0	1	0	0	1	0	0	1	0	1	0	0	0	0	0	0	0	1	0	0	0	1	1	7
**1.398**	0	1	0	0	0	0	1	0	0	1	0	1	0	1	0	0	0	0	0	1	0	0	0	1	1	7
**1.399**	0	1	0	1	0	0	0	0	1	0	0	1	0	0	0	0	0	0	0	0	0	0	1	1	0	7
**1.400**	0	1	0	1	1	0	0	0	0	1	0	0	1	0	0	0	0	0	0	1	0	0	0	1	1	7
**1.401**	0	1	1	0	0	0	1	0	1	1	0	1	0	0	0	0	0	0	0	1	0	0	0	1	1	7
**1.402**	0	1	0	1	1	0	0	0	0	0	0	1	1	0	0	0	0	0	0	0	0	0	1	1	0	7
**1.403**	0	1	1	0	0	0	0	0	1	1	0	1	1	1	0	0	0	0	0	1	0	0	0	1	0	7
**1.404**	0	1	1	0	1	0	0	0	1	1	0	1	1	1	0	0	0	0	0	1	0	0	0	1	0	7
**1.405**	0	1	0	0	1	0	0	0	0	1	0	1	1	0	0	0	0	0	0	0	0	0	1	1	0	7
**1.406**	0	0	0	0	0	0	0	0	0	0	1	1	0	1	0	0	0	0	0	1	0	1	0	1	0	7
**1.407**	0	0	1	0	0	0	0	0	1	0	0	0	0	1	0	0	0	0	0	0	0	0	0	1	0	7
**1.408**	0	0	1	0	0	0	1	0	0	1	0	1	0	1	0	0	0	0	0	0	0	1	0	1	1	7
**1.409**	0	1	1	1	1	0	0	0	1	1	0	1	0	0	0	0	0	0	0	1	0	0	0	1	1	7
**1.410**	0	0	0	0	1	0	0	0	0	0	0	0	1	0	0	0	0	0	0	0	0	0	0	1	0	7
**1.411**	0	0	1	1	0	0	1	0	0	1	1	1	1	1	0	1	0	0	0	0	0	0	0	1	1	7
**1.412**	0	0	0	1	0	0	1	0	0	1	0	1	0	1	0	0	0	0	0	1	0	1	0	1	1	7
**1.413**	0	0	1	0	0	0	0	0	0	1	0	1	0	0	0	0	0	0	0	0	0	0	0	1	0	7
**1.414**	0	1	0	0	0	0	0	0	0	1	1	1	0	0	0	0	0	0	0	0	0	0	0	1	0	7
**1.415**	0	0	0	0	0	0	0	0	0	0	0	1	1	0	0	0	0	0	0	0	0	1	0	1	0	7
**1.416**	0	0	0	0	0	0	0	0	0	1	1	1	0	0	0	0	0	0	0	1	0	1	0	1	0	7
**1.417**	0	0	0	0	0	0	0	0	0	1	0	0	0	1	0	1	0	0	0	0	0	0	0	1	1	7
**1.418**	0	0	0	0	0	0	1	0	1	0	0	0	1	0	0	1	0	0	0	1	0	0	0	1	0	7
**1.419**	0	0	0	0	1	0	1	0	1	0	0	0	1	0	0	0	0	0	0	0	0	0	0	1	0	7
**1.420**	0	1	0	0	0	0	0	0	0	0	0	1	1	0	0	0	0	0	0	0	0	1	0	1	0	7
**1.421**	0	0	0	0	0	0	1	0	0	0	0	0	1	0	0	1	0	0	0	1	0	0	0	1	0	7
**1.422**	0	0	0	0	0	0	0	0	0	1	1	0	0	0	0	0	0	0	0	1	0	0	0	1	1	7
**1.423**	0	0	0	0	1	0	0	0	1	0	0	0	0	0	0	0	0	0	0	1	0	1	0	1	1	6
**1.424**	0	0	1	0	0	0	1	0	0	0	0	0	0	0	0	0	0	0	0	1	0	1	0	1	0	6
**1.425**	0	1	0	0	0	0	0	0	0	0	0	1	0	1	0	0	0	0	0	0	0	1	1	1	1	6
**1.426**	0	1	0	1	0	0	0	0	0	0	0	0	0	1	0	0	0	0	0	0	0	1	1	1	0	6
**1.427**	0	0	0	0	0	0	1	0	0	0	1	1	0	0	0	0	0	0	0	0	0	1	0	1	0	6
**1.428**	0	0	0	0	0	0	0	0	1	0	0	1	0	1	0	1	0	0	0	0	0	0	0	1	1	6
**1.429**	0	0	0	0	0	0	0	0	1	0	1	1	0	1	0	0	0	0	0	1	0	0	0	1	1	6
**1.430**	0	1	0	0	1	0	0	0	0	0	0	0	1	0	0	0	0	0	0	0	0	0	1	1	1	6
**1.431**	0	0	0	0	1	0	1	0	0	1	0	1	0	1	0	0	0	0	0	0	0	0	0	1	1	6
**1.432**	0	0	0	0	1	0	0	0	1	1	0	1	1	0	0	0	0	0	0	0	0	0	0	1	1	6
**1.433**	0	0	0	1	0	0	0	0	1	0	0	1	0	1	0	0	0	0	0	0	0	1	0	1	1	6
**1.434**	0	0	1	1	0	0	0	0	0	0	0	1	0	0	0	0	0	0	0	0	0	1	0	1	1	6
**1.435**	0	1	0	0	1	0	0	0	1	1	0	1	1	0	0	0	0	0	0	1	0	1	1	1	1	6
**1.436**	0	0	0	1	0	0	0	0	1	1	0	1	1	0	0	0	0	0	0	1	0	0	0	1	1	6
**1.437**	0	0	0	0	0	0	0	0	0	1	0	1	0	0	0	1	0	0	0	0	0	0	1	1	0	6
**1.438**	0	1	0	1	1	0	0	0	0	0	0	1	1	0	0	0	0	0	0	1	0	1	1	1	1	6
**1.439**	0	0	0	0	1	0	1	0	1	1	0	1	0	1	0	0	0	0	0	1	0	0	0	1	0	6
**1.440**	0	0	1	1	0	0	0	0	0	1	0	1	0	0	0	0	0	0	0	1	0	0	0	1	0	6
**1.441**	0	0	1	1	0	0	0	0	1	1	0	1	0	0	0	0	0	0	0	0	0	0	0	1	1	6
**1.442**	0	0	1	0	0	0	1	0	0	0	0	0	1	0	0	0	0	0	0	1	0	0	0	1	1	6
**1.443**	0	1	0	1	0	0	0	0	0	0	0	0	0	0	0	0	0	0	0	0	0	0	1	1	0	6
**1.444**	0	0	0	0	0	0	0	0	0	0	0	0	1	1	0	0	0	0	0	0	0	0	0	1	1	6
**1.445**	0	0	0	0	0	0	0	0	0	0	1	1	0	1	0	0	0	0	0	1	0	1	0	1	1	6
**1.446**	0	0	0	0	0	0	1	0	1	0	0	0	0	1	0	0	0	0	0	1	0	1	0	1	1	6
**1.447**	0	0	0	0	0	0	1	0	0	0	0	1	0	1	0	1	0	0	0	0	0	0	0	1	1	6
**1.448**	0	0	1	0	1	0	0	0	1	0	0	1	1	0	0	0	0	0	0	1	0	0	0	1	1	6
**1.449**	0	1	0	1	0	0	0	0	1	1	1	1	0	0	0	0	0	0	0	0	0	0	0	1	1	6
**1.450**	0	0	0	0	0	0	0	0	0	0	1	0	0	1	0	0	0	0	0	0	0	0	0	1	0	6
**1.451**	0	0	0	0	0	0	1	0	1	1	0	0	0	1	0	0	0	0	0	0	0	1	0	1	1	6
**1.452**	0	0	1	0	0	0	0	0	0	1	0	1	0	1	0	0	0	0	0	1	0	0	1	1	1	6
**1.453**	0	1	0	0	1	0	0	0	0	0	0	0	1	0	0	0	0	0	0	0	0	1	0	1	1	6
**1.454**	0	0	0	0	0	0	1	0	0	0	1	1	0	0	0	0	0	0	0	0	0	0	0	1	0	6
**1.455**	0	0	0	0	0	0	1	0	1	0	0	0	0	0	0	0	0	0	0	1	0	1	0	1	0	6
**1.456**	0	0	0	0	0	0	0	0	1	0	0	0	1	0	0	0	0	0	0	0	0	1	0	1	1	6
**1.457**	0	0	0	1	0	0	0	0	0	1	0	0	0	1	0	0	0	0	0	0	0	1	0	1	1	6
**1.458**	0	1	0	0	1	0	0	0	0	0	0	1	1	1	0	0	0	0	0	0	0	1	0	1	1	6
**1.459**	0	1	0	1	1	0	1	0	0	1	0	1	0	0	0	0	0	0	0	0	0	0	1	1	1	6
**1.460**	0	1	0	0	0	0	1	0	0	0	0	1	0	1	0	0	0	0	0	1	0	0	0	1	1	6
**1.461**	0	1	0	0	0	0	1	0	0	1	0	1	0	1	0	0	0	0	0	0	0	0	0	1	1	6
**1.462**	0	1	0	0	1	0	0	0	0	1	0	1	1	0	0	0	0	0	0	1	0	1	0	1	1	6
**1.463**	0	1	0	1	0	0	1	0	0	0	0	1	1	1	0	0	0	0	0	1	0	1	0	1	1	6
**1.464**	0	1	1	0	0	0	0	0	1	0	0	1	1	0	0	0	0	0	0	1	0	1	0	1	1	6
**1.465**	0	0	0	1	0	0	1	0	0	1	0	1	0	1	0	0	0	0	0	1	0	0	1	1	1	6
**1.466**	0	0	0	0	0	0	0	0	0	0	0	1	1	0	0	0	0	0	0	1	0	1	0	1	1	6
**1.467**	0	0	0	0	0	0	0	0	1	0	0	1	1	0	0	0	0	0	0	0	0	1	0	1	1	6
**1.468**	0	0	0	0	0	0	1	0	0	0	0	0	0	1	0	0	0	0	0	1	0	1	0	1	1	6
**1.469**	0	1	0	0	1	0	0	0	0	1	0	1	1	0	0	0	0	0	0	1	0	0	1	1	0	6

	CD2	CD3	CD4	CD7	CD8	CD10	CD13	CD18	CD26	CD29	CD36	CD44	CD45RA	CD49f	CD54	CD56	CD57	CD58	CD62L	CD71	CD80	Cytokeratin	Histone_H1	HLA-DQ	HLA-DR	Freq. (Voxel)
**1.470**	0	0	0	0	0	0	0	0	0	1	0	0	0	0	0	1	0	0	0	0	0	0	0	1	0	6
**1.471**	0	1	0	0	1	0	0	0	0	1	0	1	1	0	0	0	0	0	0	1	0	0	1	1	1	6
**1.472**	0	1	0	1	0	0	0	0	0	0	0	1	0	1	0	0	0	0	0	0	0	1	0	1	0	6
**1.473**	0	0	1	0	0	0	1	0	1	0	0	1	0	0	0	0	0	0	0	0	0	0	0	1	0	6
**1.474**	0	1	1	1	0	0	0	0	1	0	0	1	0	0	0	0	0	0	0	0	0	0	0	1	0	6
**1.475**	0	0	0	0	0	0	0	0	0	0	0	1	0	1	0	1	0	0	0	1	0	1	0	1	0	6
**1.476**	0	0	0	0	0	0	1	0	1	1	0	0	0	1	0	0	0	0	0	0	0	0	0	1	0	6
**1.477**	0	1	0	1	0	0	0	0	1	1	0	1	0	0	0	0	0	0	0	1	0	0	0	1	0	6
**1.478**	0	0	1	0	0	0	1	0	0	1	0	1	0	1	0	1	0	0	0	0	0	0	0	1	1	6
**1.479**	0	0	1	0	0	0	1	0	1	1	0	1	0	1	0	1	0	0	0	0	0	0	0	1	1	6
**1.480**	0	0	1	1	0	0	1	0	0	1	1	1	0	1	0	1	0	0	0	1	0	0	0	1	1	6
**1.481**	0	0	1	0	0	0	1	0	0	1	0	1	0	1	0	1	0	0	0	1	0	0	0	1	1	6
**1.482**	0	0	1	0	0	0	0	0	1	1	0	1	0	1	0	0	0	0	0	0	0	1	0	1	0	6
**1.483**	0	0	0	0	0	0	0	0	1	0	0	0	0	0	0	0	0	0	0	0	0	1	1	1	1	6
**1.484**	0	0	0	0	0	0	0	0	0	0	0	0	0	1	0	1	0	0	0	0	0	0	1	1	0	6
**1.485**	0	0	0	1	1	0	0	0	0	1	0	1	1	0	0	0	0	0	0	0	0	0	0	1	0	6
**1.486**	0	0	0	1	1	0	1	0	1	0	0	1	0	0	0	0	0	0	0	0	0	0	1	1	0	6
**1.487**	0	0	1	1	0	0	1	0	1	0	0	1	0	0	0	0	0	0	0	1	0	0	0	1	1	6
**1.488**	0	0	0	0	0	0	0	0	0	1	0	0	1	0	0	0	0	0	0	0	0	1	0	1	0	6
**1.489**	0	0	0	0	1	0	0	0	0	1	0	1	0	1	0	0	0	0	0	0	0	0	0	1	0	6
**1.490**	0	0	0	0	1	0	0	0	1	1	0	1	0	1	0	0	0	0	0	0	0	0	0	1	0	6
**1.491**	0	0	0	0	1	0	1	0	1	0	0	1	0	0	0	0	0	0	0	1	0	1	0	1	1	6
**1.492**	0	0	0	1	0	0	1	0	0	0	0	1	0	0	0	0	0	0	0	0	0	0	0	1	0	6
**1.493**	0	0	0	1	0	0	1	0	0	1	0	1	0	0	0	0	0	0	0	0	0	0	0	1	0	6
**1.494**	0	0	0	1	1	0	0	0	0	0	0	1	0	0	0	0	0	0	0	1	0	0	1	1	1	6
**1.495**	0	0	1	0	0	0	0	0	1	0	0	1	0	1	0	0	0	0	0	0	0	1	0	1	0	6
**1.496**	0	1	0	0	1	0	0	0	1	0	0	1	1	0	0	0	0	0	0	0	0	1	1	1	0	6
**1.497**	0	0	0	0	0	0	0	0	1	0	0	1	0	0	0	0	0	0	0	1	0	0	1	1	0	6
**1.498**	0	0	0	0	1	0	0	0	1	0	0	1	1	0	0	0	0	0	0	0	0	1	0	1	1	6
**1.499**	0	0	0	1	0	0	0	0	0	1	0	1	1	1	0	0	0	0	0	1	0	1	0	1	0	6
**1.500**	0	0	0	1	0	0	0	0	1	1	0	1	0	0	0	0	0	0	0	1	0	0	0	1	1	6
**1.501**	0	0	1	0	0	0	1	0	1	1	0	1	1	0	0	0	0	0	0	1	0	0	0	1	1	6
**1.502**	0	0	0	0	1	0	1	0	0	1	0	1	0	1	0	0	0	0	0	1	0	0	0	1	1	6
**1.503**	0	0	0	1	1	0	0	0	0	1	0	1	0	1	0	0	0	0	0	1	0	1	0	1	1	6
**1.504**	0	0	1	0	0	0	0	0	0	0	0	0	0	0	0	0	0	0	0	1	0	0	1	1	0	6
**1.505**	0	1	0	0	0	0	0	0	1	1	0	1	1	0	0	0	0	0	0	1	0	1	0	1	0	6
**1.506**	0	1	0	0	0	0	1	0	0	0	0	1	0	1	0	0	0	0	0	0	0	0	0	1	1	6
**1.507**	0	1	0	0	1	0	0	0	1	0	0	1	1	0	0	0	0	0	0	1	0	0	1	1	0	6
**1.508**	0	1	0	0	1	0	0	0	0	1	0	1	0	0	0	0	0	0	0	1	0	0	1	1	0	6
**1.509**	0	0	0	0	0	0	0	0	0	1	0	1	1	0	0	0	0	0	0	0	0	0	0	1	1	6
**1.510**	0	0	1	0	0	0	1	0	0	0	1	1	0	1	0	0	0	0	0	1	0	1	0	1	1	6
**1.511**	0	1	0	0	1	0	0	0	1	0	0	0	0	0	0	0	0	0	0	0	0	0	1	1	1	6
**1.512**	0	1	1	0	0	0	0	0	0	1	0	1	0	1	0	0	0	0	0	0	0	0	0	1	0	6
**1.513**	0	0	0	1	0	0	0	0	0	0	0	1	0	0	0	0	0	0	0	0	0	0	1	1	1	6
**1.514**	0	1	0	0	1	0	0	0	1	0	0	1	1	0	0	0	0	0	0	0	0	0	1	1	0	6
**1.515**	0	1	0	0	1	0	1	0	0	1	0	1	0	1	0	0	0	0	0	0	0	1	1	1	1	6
**1.516**	0	0	0	0	0	0	1	0	1	0	0	0	0	1	0	0	0	0	0	0	0	1	0	1	0	6
**1.517**	0	0	0	0	0	0	1	0	1	1	0	0	0	0	0	0	0	0	0	1	0	0	0	1	0	6
**1.518**	0	0	1	0	0	0	1	0	0	1	1	1	0	0	0	0	0	0	0	0	0	0	0	1	1	6
**1.519**	0	1	0	0	1	0	0	0	1	1	0	1	1	0	0	0	0	0	0	0	0	1	1	1	1	6
**1.520**	0	0	0	0	0	0	1	0	0	1	0	0	0	1	0	0	0	0	0	0	0	0	1	1	1	6
**1.521**	0	0	1	0	0	0	1	0	1	1	1	1	1	1	0	0	0	0	0	1	0	0	0	1	1	6
**1.522**	0	0	0	0	0	0	0	0	1	0	0	1	0	0	0	1	0	0	0	1	0	0	0	1	1	6
**1.523**	0	0	0	0	0	0	0	0	1	1	0	0	0	1	0	0	0	0	0	0	0	0	0	1	1	6
**1.524**	0	1	0	0	1	0	0	0	1	1	0	1	0	0	0	0	0	0	0	1	0	0	1	1	0	6
**1.525**	0	0	1	0	0	0	1	0	0	1	0	1	1	1	0	1	0	0	0	1	0	0	0	1	1	6
**1.526**	0	0	0	0	0	0	0	0	0	0	1	1	0	0	0	0	0	0	0	0	0	0	1	1	0	6
**1.527**	0	0	1	0	0	0	0	0	0	1	1	0	0	1	0	0	0	0	0	1	0	0	0	1	1	6
**1.528**	0	0	1	0	0	0	1	0	0	0	1	1	0	0	0	0	0	0	0	0	0	0	0	1	1	6
**1.529**	0	0	1	0	0	0	1	0	0	0	1	0	0	0	0	0	0	0	0	0	0	0	0	1	1	6
**1.530**	0	1	0	1	1	0	0	0	0	0	0	1	0	0	0	0	0	0	0	0	0	1	0	1	0	6
**1.531**	0	1	0	1	1	0	0	0	1	1	0	1	1	0	0	0	0	0	0	1	0	0	1	1	0	5
**1.532**	0	0	0	1	0	0	0	0	0	1	0	1	1	0	0	1	0	0	0	0	0	0	0	1	0	5
**1.533**	0	1	1	1	1	0	1	0	1	0	0	1	1	0	0	0	0	0	0	1	0	0	0	1	1	5
**1.534**	0	0	1	0	0	0	1	0	1	1	0	0	0	0	0	0	0	0	0	1	0	0	0	1	1	5
**1.535**	0	1	0	1	1	0	0	0	1	0	0	1	1	0	0	0	0	0	0	0	0	0	1	1	0	5
**1.536**	0	0	0	0	0	0	0	0	1	0	0	0	0	0	0	0	0	0	0	1	0	1	1	1	0	5
**1.537**	0	0	0	0	1	0	1	0	1	0	0	1	0	0	0	1	0	0	0	1	0	0	0	1	1	5
**1.538**	0	1	1	0	0	0	0	0	1	0	0	1	0	0	0	0	0	0	0	1	0	1	1	1	1	5
**1.539**	0	1	0	1	0	0	0	0	0	0	0	1	0	1	0	0	0	0	0	1	0	0	0	1	1	5
**1.540**	0	1	0	1	0	0	0	0	0	0	0	1	1	0	0	0	0	0	0	1	0	1	0	1	1	5
**1.541**	0	1	1	0	0	0	0	0	1	1	0	1	0	0	0	1	0	0	0	1	0	0	0	1	1	5
**1.542**	0	0	1	0	1	0	0	0	0	1	0	1	0	0	0	0	0	0	0	1	0	0	0	1	0	5
**1.543**	0	1	0	0	0	0	0	0	0	1	0	1	0	1	0	0	0	0	0	1	0	0	1	1	0	5
**1.544**	0	0	1	0	0	0	1	0	0	1	0	1	1	1	0	0	0	0	0	1	0	1	0	1	0	5
**1.545**	0	0	1	1	0	0	0	0	1	0	0	1	1	1	0	0	0	0	0	1	0	1	0	1	1	5
**1.546**	0	1	0	0	1	0	0	0	1	0	0	0	1	0	0	0	0	0	0	0	0	0	0	1	1	5
**1.547**	0	1	0	1	0	0	0	0	0	1	0	1	0	1	0	0	0	0	0	1	0	0	0	1	1	5
**1.548**	0	0	0	0	0	0	0	0	0	1	0	1	1	1	0	0	0	0	0	0	0	0	0	1	1	5
**1.549**	0	1	0	0	1	0	0	0	0	0	0	0	1	0	0	0	0	0	0	0	0	1	1	1	1	5
**1.550**	0	1	0	1	1	0	0	0	0	1	0	1	0	1	0	0	0	0	0	0	0	0	0	1	1	5
**1.551**	0	0	1	1	0	0	1	0	1	1	0	1	1	1	0	1	0	0	0	1	0	0	1	1	1	5
**1.552**	0	0	0	0	0	0	0	0	0	0	1	0	0	0	0	1	0	0	0	0	0	0	0	1	0	5
**1.553**	0	1	0	0	1	0	0	0	0	1	1	1	0	0	0	0	0	0	0	0	0	0	1	1	1	5
**1.554**	0	0	0	0	1	0	0	0	1	1	0	1	1	0	0	0	0	0	0	0	0	0	0	1	0	5
**1.555**	0	0	0	1	1	0	0	0	1	0	0	1	0	0	0	1	0	0	0	1	0	0	0	1	1	5
**1.556**	0	0	1	0	0	0	1	0	0	0	0	0	0	0	0	1	0	0	0	0	0	0	0	1	1	5
**1.557**	0	1	0	0	0	0	1	0	1	1	0	1	0	0	0	0	0	0	0	1	0	0	0	1	1	5
**1.558**	0	1	0	0	1	0	0	0	1	0	0	0	0	0	0	0	0	0	0	1	0	1	0	1	1	5
**1.559**	0	0	0	0	0	0	0	0	0	0	0	1	1	0	0	1	0	0	0	0	0	0	0	1	0	5
**1.560**	0	0	0	1	0	0	0	0	0	1	0	0	1	0	0	0	0	0	0	1	0	0	0	1	0	5
**1.561**	0	0	0	1	0	0	0	0	1	0	0	0	0	1	0	0	0	0	0	1	0	1	0	1	0	5
**1.562**	0	1	0	1	1	0	0	0	1	0	0	1	0	0	0	0	0	0	0	1	0	1	1	1	1	5
**1.563**	0	1	1	0	1	0	0	0	0	0	0	1	1	0	0	0	0	0	0	0	0	0	0	1	1	5
**1.564**	0	0	0	0	0	0	0	0	0	0	0	0	1	0	0	0	0	0	0	1	0	1	0	1	1	5
**1.565**	0	0	0	0	1	0	0	0	0	0	0	1	1	0	0	0	0	0	0	1	0	1	0	1	1	5
**1.566**	0	0	0	1	0	0	0	0	0	0	0	1	0	0	0	0	0	0	0	1	0	0	1	1	1	5
**1.567**	0	0	0	1	0	0	0	0	0	0	0	1	0	1	0	0	0	0	0	1	0	1	0	1	0	5

	CD2	CD3	CD4	CD7	CD8	CD10	CD13	CD18	CD26	CD29	CD36	CD44	CD45RA	CD49f	CD54	CD56	CD57	CD58	CD62L	CD71	CD80	Cytokeratin	Histone_H1	HLA-DQ	HLA-DR	Freq. (Voxel)
**1.568**	0	0	0	1	0	0	1	0	1	0	0	1	0	0	0	0	0	0	0	1	0	0	0	1	1	5
**1.569**	0	0	1	1	0	0	0	0	0	0	0	0	1	0	0	0	0	0	0	1	0	1	0	1	1	5
**1.570**	0	0	0	0	0	0	0	0	1	0	0	1	1	1	0	0	0	0	0	1	0	1	0	1	1	5
**1.571**	0	1	0	0	1	0	0	0	0	1	0	1	1	0	0	0	0	0	0	0	0	1	1	1	1	5
**1.572**	0	1	0	1	1	0	0	0	1	1	0	1	0	1	0	0	0	0	0	0	0	1	0	1	1	5
**1.573**	0	1	1	0	0	0	0	0	0	0	0	1	0	0	0	0	0	0	0	0	0	1	0	1	1	5
**1.574**	0	1	1	0	1	0	0	0	0	0	0	0	1	0	0	0	0	0	0	1	0	0	0	1	1	5
**1.575**	0	1	1	0	0	0	0	0	1	0	0	0	0	0	0	0	0	0	0	1	0	0	0	1	1	5
**1.576**	0	1	1	1	1	0	0	0	0	0	0	1	1	0	0	0	0	0	0	1	0	0	0	1	0	5
**1.577**	0	0	1	0	0	0	1	0	0	1	0	0	0	1	0	0	0	0	0	0	0	0	1	1	0	5
**1.578**	0	1	0	0	0	0	0	0	0	1	0	1	0	0	0	0	0	0	0	1	0	0	1	1	0	5
**1.579**	0	0	1	0	0	0	0	0	1	0	0	1	1	1	0	0	0	0	0	1	0	1	0	1	1	5
**1.580**	0	0	1	0	0	0	0	0	1	1	0	1	0	1	0	0	0	0	0	0	0	0	0	1	0	5
**1.581**	0	0	1	0	0	0	1	0	0	0	0	1	0	1	0	0	0	0	0	0	0	0	0	1	0	5
**1.582**	0	1	0	0	0	0	0	0	0	0	0	0	0	1	0	0	0	0	0	0	0	0	1	1	1	5
**1.583**	0	0	0	1	0	0	0	0	0	1	0	1	0	1	0	0	0	0	0	0	0	0	0	1	1	5
**1.584**	0	0	1	0	0	0	0	0	1	0	1	0	0	0	0	0	0	0	0	0	0	1	0	1	0	5
**1.585**	0	1	0	0	0	0	0	0	0	0	0	0	1	0	0	0	0	0	0	0	0	1	1	1	0	5
**1.586**	0	0	0	1	0	0	0	0	0	1	0	0	0	1	0	0	0	0	0	0	0	0	0	1	0	5
**1.587**	0	0	1	0	0	0	1	0	1	0	0	0	1	0	0	0	0	0	0	0	0	0	0	1	0	5
**1.588**	0	1	0	1	1	0	0	0	0	0	0	1	0	0	0	0	0	0	0	1	0	1	1	1	0	5
**1.589**	0	0	0	1	0	0	1	0	0	1	0	1	0	1	0	0	0	0	0	1	0	0	0	1	0	5
**1.590**	0	0	1	0	0	0	1	0	1	1	0	0	0	0	0	0	0	0	0	1	0	0	0	1	0	5
**1.591**	0	0	1	1	0	0	1	0	0	1	1	1	1	1	0	0	0	0	0	0	0	0	0	1	1	5
**1.592**	0	1	0	0	0	0	0	0	1	1	0	1	0	0	0	0	0	0	0	0	0	0	1	1	1	5
**1.593**	0	1	1	0	1	0	0	0	0	0	0	1	0	0	0	0	0	0	0	0	0	0	0	1	0	5
**1.594**	0	1	1	1	1	0	0	0	1	0	0	1	0	0	0	0	0	0	0	1	0	0	0	1	0	5
**1.595**	0	0	1	1	0	0	1	0	1	1	0	0	0	1	0	0	0	0	0	1	0	0	0	1	1	5
**1.596**	0	1	0	0	0	0	0	0	0	0	0	0	1	0	0	0	0	0	0	0	0	0	0	1	1	5
**1.597**	0	0	0	0	0	0	0	0	0	0	0	0	1	0	0	1	0	0	0	1	0	0	1	1	0	5
**1.598**	0	0	0	0	0	0	0	0	1	1	1	0	0	1	0	0	0	0	0	0	0	0	0	1	1	5
**1.599**	0	0	1	0	0	0	0	0	0	1	0	1	0	1	0	0	0	0	0	0	0	1	1	1	0	5
**1.600**	0	0	0	0	0	0	0	0	0	1	0	0	0	1	0	0	0	0	0	0	0	1	1	1	0	5
**1.601**	0	0	1	0	0	0	0	0	0	0	0	0	1	0	0	1	0	0	0	0	0	0	1	1	0	5
**1.602**	0	0	0	1	1	0	0	0	1	0	0	1	0	0	0	0	0	0	0	0	0	1	0	1	0	5
**1.603**	0	1	0	0	0	0	0	0	1	0	0	1	0	0	0	0	0	0	0	1	0	1	0	1	0	5
**1.604**	0	1	0	1	0	0	0	0	0	0	0	0	1	0	0	0	0	0	0	1	0	0	0	1	1	5
**1.605**	0	1	0	1	1	0	0	0	1	1	0	1	0	0	0	0	0	0	0	0	0	1	0	1	0	5
**1.606**	0	0	0	1	0	0	0	0	1	0	0	1	1	0	0	0	0	0	0	0	0	0	0	1	1	5
**1.607**	0	0	0	1	0	0	0	0	1	1	0	1	1	1	0	0	0	0	0	1	0	0	0	1	0	5
**1.608**	0	0	0	1	1	0	1	0	1	0	0	1	0	0	0	0	0	0	0	0	0	0	1	1	1	5
**1.609**	0	0	1	0	0	0	0	0	1	1	0	0	0	0	0	0	0	0	0	0	0	1	0	1	1	5
**1.610**	0	0	1	0	0	0	1	0	1	0	0	1	0	0	0	0	0	0	0	1	0	1	0	1	1	5
**1.611**	0	1	0	1	0	0	0	0	1	0	0	1	0	0	0	0	0	0	0	0	0	1	0	1	0	5
**1.612**	0	1	1	0	0	0	1	0	1	1	0	1	1	0	0	0	0	0	0	1	0	1	0	1	1	5
**1.613**	0	1	0	0	0	0	0	0	1	0	0	1	1	1	0	0	0	0	0	1	0	0	0	1	1	5
**1.614**	0	1	0	1	1	0	0	0	1	1	0	1	0	0	0	0	0	0	0	0	0	0	1	1	0	5
**1.615**	0	0	0	0	0	0	1	0	0	0	0	1	0	0	0	1	0	0	0	0	0	0	1	1	0	5
**1.616**	0	0	0	1	0	0	0	0	0	0	0	0	0	1	0	0	0	0	0	1	0	1	0	1	0	5
**1.617**	0	0	0	1	1	0	0	0	1	1	0	1	0	1	0	0	0	0	0	1	0	1	0	1	1	5
**1.618**	0	0	1	0	0	0	0	0	0	0	0	1	0	0	0	0	0	0	0	1	0	0	1	1	1	5
**1.619**	0	0	0	1	0	0	0	0	0	0	0	1	0	1	0	0	0	0	0	1	0	1	0	1	1	5
**1.620**	0	0	0	1	0	0	0	0	0	1	0	1	0	0	0	0	0	0	0	1	0	1	0	1	0	5
**1.621**	0	1	0	0	0	0	0	0	1	0	0	1	1	0	0	0	0	0	0	0	0	1	0	1	1	5
**1.622**	0	1	0	0	1	0	0	0	0	0	0	1	1	0	0	0	0	0	0	1	0	1	0	1	0	5
**1.623**	0	1	0	0	1	0	0	0	0	1	0	1	1	0	0	0	0	0	0	1	0	1	0	1	0	5
**1.624**	0	1	0	1	0	0	0	0	0	0	0	1	0	0	0	0	0	0	0	0	0	1	1	1	1	5
**1.625**	0	1	0	1	0	0	0	0	0	0	0	1	0	1	0	0	0	0	0	1	0	1	0	1	1	5
**1.626**	0	0	0	0	0	0	0	0	0	1	0	1	1	0	0	0	0	0	0	0	0	1	0	1	0	5
**1.627**	0	0	0	0	1	0	0	0	0	1	0	1	0	0	0	0	0	0	0	1	0	0	1	1	0	5
**1.628**	0	0	0	1	0	0	1	0	0	1	0	1	0	1	0	1	0	0	0	1	0	0	0	1	1	5
**1.629**	0	1	0	1	1	0	0	0	0	0	0	1	0	0	0	0	0	0	0	1	0	1	1	1	1	5
**1.630**	0	1	0	1	1	0	0	0	0	1	0	1	1	1	0	0	0	0	0	1	0	0	0	1	0	5
**1.631**	0	0	0	0	1	0	1	0	0	1	0	1	0	1	0	0	0	0	0	1	0	1	0	1	0	5
**1.632**	0	0	0	0	1	0	1	0	0	1	0	1	0	1	0	0	0	0	0	1	0	1	1	1	1	5
**1.633**	0	0	1	0	0	0	1	0	0	0	0	1	0	1	0	0	0	0	0	1	0	1	0	1	1	5
**1.634**	0	1	0	0	0	0	0	0	1	1	0	1	0	1	0	0	0	0	0	1	0	0	0	1	0	5
**1.635**	0	1	1	1	0	0	0	0	0	1	0	1	0	0	0	0	0	0	0	0	0	0	0	1	1	5
**1.636**	0	0	1	0	0	0	0	0	0	1	0	0	1	1	0	0	0	0	0	0	0	0	0	1	0	5
**1.637**	0	0	1	1	0	0	0	0	0	0	0	0	1	0	0	0	0	0	0	0	0	0	0	1	0	5
**1.638**	0	0	1	1	0	0	1	0	0	1	0	1	1	1	0	0	0	0	0	1	0	1	0	1	1	5
**1.639**	0	1	0	0	1	0	0	0	0	1	0	1	1	1	0	0	0	0	0	1	0	0	0	1	1	5
**1.640**	0	0	0	0	0	0	0	0	0	0	1	0	0	1	0	0	0	0	0	0	0	1	0	1	0	5
**1.641**	0	1	0	0	0	0	0	0	1	0	0	0	0	0	0	0	0	0	0	1	0	0	1	1	0	5
**1.642**	0	1	0	0	1	0	0	0	0	0	0	1	0	1	0	0	0	0	0	0	0	1	0	1	0	5
**1.643**	0	0	0	0	0	0	0	0	1	0	0	0	0	1	0	0	0	0	0	0	0	0	0	1	0	5
**1.644**	0	0	1	0	0	0	1	0	0	1	0	1	0	1	0	1	0	0	0	1	0	1	0	1	1	5
**1.645**	0	1	0	0	0	0	0	0	0	0	0	0	0	0	0	0	0	0	0	1	0	0	1	1	0	5
**1.646**	0	0	0	0	0	0	0	0	0	1	1	1	0	0	0	1	0	0	0	0	0	0	0	1	1	5
**1.647**	0	0	0	0	0	0	1	0	0	1	1	1	0	1	0	1	0	0	0	0	0	0	0	1	1	5
**1.648**	0	0	1	0	0	0	1	0	1	1	0	1	1	1	0	0	0	0	0	0	0	0	0	1	1	5
**1.649**	0	0	1	1	0	0	1	0	0	1	1	1	1	1	0	0	0	0	0	1	0	0	0	1	1	5
**1.650**	0	1	0	0	0	0	0	0	0	0	0	1	0	0	0	1	0	0	0	0	0	0	0	1	0	5
**1.651**	0	1	0	1	0	0	0	0	0	0	0	0	0	0	0	0	0	0	0	0	0	0	0	1	0	5
**1.652**	0	0	0	0	0	0	0	0	1	1	0	0	0	0	0	0	0	0	0	1	0	0	1	1	0	5
**1.653**	0	0	0	0	0	0	1	0	0	1	0	1	1	1	0	0	0	0	0	1	0	1	0	1	1	5
**1.654**	0	0	1	0	0	0	1	0	0	1	1	1	1	1	0	1	0	0	0	1	0	0	0	1	1	5
**1.655**	0	0	1	0	0	0	0	0	1	0	0	1	0	1	0	0	0	0	0	0	0	0	0	1	1	5
**1.656**	0	0	0	0	0	0	0	0	0	1	0	0	0	1	0	0	0	0	0	1	0	0	1	1	0	5
**1.657**	0	0	0	0	1	0	0	0	0	0	0	0	1	0	0	1	0	0	0	1	0	0	0	1	0	5
**1.658**	0	0	0	0	0	0	0	0	0	0	1	1	0	1	0	0	0	0	0	0	0	0	0	1	0	5
**1.659**	0	0	0	1	0	0	0	0	0	1	0	0	0	0	0	0	0	0	0	0	0	0	0	1	1	5
**1.660**	0	0	1	0	0	0	0	0	0	0	0	0	0	0	0	0	0	0	0	0	0	1	1	1	0	5
**1.661**	0	1	0	1	1	0	0	0	0	0	0	1	1	0	0	0	0	0	0	0	0	1	0	1	0	5
**1.662**	0	0	1	0	0	0	0	0	0	0	0	0	1	1	0	1	0	0	0	1	0	0	0	1	0	5
**1.663**	0	0	1	0	1	0	1	0	0	0	0	1	0	0	0	0	0	0	0	0	0	0	0	1	1	4
**1.664**	0	1	1	1	1	0	1	0	1	0	0	1	0	0	0	1	0	0	0	0	0	0	0	1	1	4
**1.665**	0	0	1	0	1	0	0	0	0	1	0	1	0	0	0	0	0	0	0	0	0	1	0	1	0	4

	CD2	CD3	CD4	CD7	CD8	CD10	CD13	CD18	CD26	CD29	CD36	CD44	CD45RA	CD49f	CD54	CD56	CD57	CD58	CD62L	CD71	CD80	Cytokeratin	Histone_H1	HLA-DQ	HLA-DR	Freq. (Voxel)
**1.666**	0	1	1	0	0	0	0	0	0	1	0	1	0	0	0	0	0	0	0	1	0	1	1	1	1	4
**1.667**	0	1	0	0	0	0	1	0	0	0	0	1	0	1	0	0	0	0	0	1	0	1	0	1	1	4
**1.668**	0	1	0	0	0	0	1	0	1	0	0	1	0	1	0	0	0	0	0	1	0	1	0	1	1	4
**1.669**	0	0	0	1	0	0	0	0	0	0	0	0	0	0	0	0	0	0	0	0	0	1	1	1	0	4
**1.670**	0	0	1	0	0	0	0	0	1	0	0	0	0	0	0	0	0	0	0	0	0	0	1	1	0	4
**1.671**	0	0	1	1	0	0	0	0	0	0	0	1	0	0	0	0	0	0	0	0	0	0	0	1	0	4
**1.672**	0	0	1	1	0	0	1	0	1	1	0	1	0	1	0	1	0	0	0	0	0	0	0	1	1	4
**1.673**	0	1	0	0	0	0	0	0	0	0	0	0	0	1	0	0	0	0	0	0	0	1	0	1	1	4
**1.674**	0	1	1	0	1	0	0	0	1	0	0	0	1	0	0	0	0	0	0	0	0	0	0	1	1	4
**1.675**	0	1	1	0	1	0	0	0	1	0	0	1	1	0	0	0	0	0	0	1	0	0	0	1	0	4
**1.676**	0	1	1	1	1	0	0	0	1	1	0	0	1	0	0	0	0	0	0	0	0	0	0	1	0	4
**1.677**	0	0	1	0	0	0	0	0	1	0	0	0	1	0	0	0	0	0	0	1	0	0	0	1	0	4
**1.678**	0	1	1	0	1	0	0	0	1	0	0	0	0	0	0	0	0	0	0	1	0	0	0	1	0	4
**1.679**	0	0	0	1	0	0	1	0	0	1	0	0	0	1	0	0	0	0	0	0	0	0	0	1	1	4
**1.680**	0	0	1	0	0	0	0	0	1	1	0	1	0	1	0	0	0	0	0	1	0	0	1	1	1	4
**1.681**	0	0	1	1	0	0	1	0	0	1	1	1	0	1	0	0	0	0	0	1	0	0	0	1	1	4
**1.682**	0	1	0	0	0	0	0	0	0	0	0	0	1	0	0	0	0	0	0	0	0	1	0	1	1	4
**1.683**	0	1	0	0	0	0	0	0	0	0	1	1	0	0	0	0	0	0	0	0	0	0	1	1	0	4
**1.684**	0	0	1	1	0	0	0	0	0	1	0	1	0	0	0	0	0	0	0	0	0	0	0	1	0	4
**1.685**	0	0	0	0	0	0	0	0	0	1	0	0	1	0	0	0	0	0	0	0	0	0	1	1	0	4
**1.686**	0	0	0	0	0	0	1	0	1	1	0	1	0	0	0	0	0	0	0	0	0	1	0	1	1	4
**1.687**	0	0	0	0	1	0	0	0	0	0	0	1	1	0	0	0	0	0	0	0	0	1	1	1	1	4
**1.688**	0	1	0	0	1	0	1	0	1	1	0	1	1	0	0	0	0	0	0	1	0	1	0	1	1	4
**1.689**	0	0	0	1	0	0	0	0	0	0	0	1	1	0	0	1	0	0	0	0	0	0	0	1	0	4
**1.690**	0	0	0	1	0	0	0	0	1	1	0	1	0	0	0	0	0	0	0	1	0	1	0	1	1	4
**1.691**	0	1	1	1	1	0	1	0	0	1	0	1	1	0	0	0	0	0	0	1	0	1	0	1	1	4
**1.692**	0	0	0	0	0	0	0	0	0	0	0	1	1	1	0	0	0	0	0	1	0	1	0	1	1	4
**1.693**	0	0	0	0	0	0	0	0	1	1	0	0	0	0	0	0	0	0	0	1	0	1	0	1	0	4
**1.694**	0	0	0	1	0	0	0	0	0	0	0	1	0	0	0	0	0	0	0	1	0	1	0	1	1	4
**1.695**	0	0	0	1	0	0	0	0	0	1	0	1	1	1	0	0	0	0	0	1	0	1	1	1	0	4
**1.696**	0	0	1	0	1	0	1	0	0	0	0	0	1	0	0	0	0	0	0	1	0	1	0	1	0	4
**1.697**	0	1	0	0	1	0	0	0	0	1	0	1	1	1	0	0	0	0	0	0	0	1	0	1	1	4
**1.698**	0	1	0	1	1	0	0	0	0	0	0	1	0	1	0	0	0	0	0	0	0	1	0	1	1	4
**1.699**	0	1	0	1	1	0	0	0	1	0	0	1	1	0	0	0	0	0	0	1	0	1	1	1	1	4
**1.700**	0	1	0	1	1	0	0	0	1	1	0	1	0	0	0	0	0	0	0	0	0	0	1	1	1	4
**1.701**	0	0	0	0	1	0	0	0	0	0	0	1	0	1	0	0	0	0	0	0	0	0	0	1	1	4
**1.702**	0	0	0	1	1	0	1	0	0	1	0	1	0	1	0	0	0	0	0	1	0	1	0	1	1	4
**1.703**	0	1	0	0	0	0	0	0	0	0	0	0	0	1	0	0	0	0	0	1	0	1	1	1	1	4
**1.704**	0	1	0	0	0	0	0	0	0	0	0	1	0	1	0	0	0	0	0	1	0	0	1	1	0	4
**1.705**	0	1	0	1	1	0	0	0	0	0	0	1	1	0	0	0	0	0	0	1	0	1	1	1	0	4
**1.706**	0	1	1	0	1	0	0	0	0	0	0	1	0	0	0	0	0	0	0	1	0	1	0	1	0	4
**1.707**	0	0	0	0	0	0	0	0	0	0	1	0	1	0	0	0	0	0	0	0	0	0	0	1	0	4
**1.708**	0	0	1	0	0	0	0	0	0	0	0	1	0	1	0	0	0	0	0	1	0	0	0	1	0	4
**1.709**	0	0	1	0	0	0	1	0	0	1	0	1	1	1	0	0	0	0	0	1	0	0	0	1	0	4
**1.710**	0	0	1	1	0	0	0	0	0	0	0	0	1	0	0	1	0	0	0	1	0	1	0	1	0	4
**1.711**	0	1	0	1	1	0	0	0	0	0	1	1	0	0	0	0	0	0	0	1	0	1	0	1	1	4
**1.712**	0	0	0	0	0	0	0	0	0	0	1	1	0	0	0	0	0	0	0	0	0	0	1	1	1	4
**1.713**	0	1	0	1	0	0	0	0	0	0	0	0	0	1	0	0	0	0	0	0	0	1	0	1	0	4
**1.714**	0	1	0	1	0	0	0	0	0	0	0	0	1	0	0	0	0	0	0	0	0	1	1	1	0	4
**1.715**	0	1	0	1	0	0	0	0	0	1	0	1	1	1	0	0	0	0	0	0	0	0	0	1	1	4
**1.716**	0	1	1	0	1	0	0	0	0	0	0	1	1	0	0	0	0	0	0	1	0	0	0	1	1	4
**1.717**	0	1	1	1	1	0	0	0	1	0	0	1	0	0	0	0	0	0	0	0	0	0	0	1	1	4
**1.718**	0	1	0	0	0	0	1	0	0	0	0	0	0	0	0	0	0	0	0	0	0	1	1	1	1	4
**1.719**	0	1	0	0	1	0	0	0	0	0	0	0	0	0	0	0	0	0	0	0	0	1	1	1	1	4
**1.720**	0	1	0	1	0	0	0	0	0	0	0	0	0	1	0	0	0	0	0	0	0	0	0	1	1	4
**1.721**	0	0	0	0	0	0	1	0	0	1	0	0	0	1	0	0	0	0	0	0	0	1	1	1	0	4
**1.722**	0	0	1	0	0	0	1	0	0	1	0	0	1	1	0	0	0	0	0	0	0	0	0	1	1	4
**1.723**	0	1	0	0	1	0	0	0	0	1	0	1	0	0	0	0	0	0	0	0	0	1	0	1	0	4
**1.724**	0	0	0	0	0	0	0	0	0	0	1	1	0	1	0	1	0	0	0	0	0	0	0	1	1	4
**1.725**	0	0	1	0	0	0	0	0	0	1	0	0	0	1	0	0	0	0	0	0	0	1	0	1	1	4
**1.726**	0	0	1	0	0	0	0	0	0	1	0	0	1	1	0	0	0	0	0	0	0	0	0	1	1	4
**1.727**	0	0	1	0	0	0	1	0	1	0	0	0	0	1	0	0	0	0	0	0	0	1	1	1	0	4
**1.728**	0	0	1	1	0	0	1	0	0	1	0	1	1	1	0	1	0	0	0	0	0	0	0	1	1	4
**1.729**	0	1	0	0	0	0	0	0	0	0	1	1	0	0	0	0	0	0	0	0	0	1	0	1	0	4
**1.730**	0	1	1	1	0	0	0	0	1	1	0	1	0	0	0	0	0	0	0	1	0	0	0	1	0	4
**1.731**	0	1	1	1	1	0	0	0	0	0	0	1	1	0	0	0	0	0	0	0	0	0	0	1	0	4
**1.732**	0	0	1	0	0	0	0	0	0	1	0	1	1	1	0	0	0	0	0	0	0	1	0	1	1	4
**1.733**	0	1	0	1	0	0	0	0	1	1	0	1	1	0	0	0	0	0	0	1	0	0	0	1	0	4
**1.734**	0	1	0	0	1	0	0	0	0	0	0	0	0	0	0	0	0	0	0	0	0	1	0	1	1	4
**1.735**	0	0	0	1	1	0	0	0	0	0	0	0	0	0	0	0	0	0	0	0	0	0	0	1	1	4
**1.736**	0	0	1	1	0	0	0	0	0	1	1	0	0	1	0	0	0	0	0	1	0	0	0	1	1	4
**1.737**	0	0	0	0	0	0	0	0	0	1	1	0	0	0	0	0	0	0	0	1	0	0	0	1	0	4
**1.738**	0	0	0	0	0	0	1	0	1	0	0	1	0	0	0	0	0	0	0	0	0	1	0	1	0	4
**1.739**	0	0	0	0	1	0	0	0	1	0	0	0	0	0	0	0	0	0	0	0	0	1	0	1	0	4
**1.740**	0	0	0	0	1	0	0	0	1	1	0	1	0	0	0	0	0	0	0	0	0	1	0	1	0	4
**1.741**	0	0	0	1	0	0	1	0	1	0	0	1	0	1	0	0	0	0	0	1	0	1	0	1	1	4
**1.742**	0	0	0	1	1	0	0	0	1	1	0	1	0	0	0	0	0	0	0	1	0	0	0	1	0	4
**1.743**	0	0	0	1	1	0	1	0	1	0	0	1	0	0	0	0	0	0	0	0	0	0	0	1	1	4
**1.744**	0	0	0	1	1	0	1	0	1	0	0	1	1	0	0	0	0	0	0	0	0	0	0	1	1	4
**1.745**	0	0	1	0	0	0	1	0	1	0	0	1	0	0	0	0	0	0	0	0	0	1	0	1	0	4
**1.746**	0	1	0	1	0	0	0	0	0	0	0	0	1	0	0	0	0	0	0	1	0	0	0	1	0	4
**1.747**	0	1	1	0	0	0	0	0	0	0	0	1	0	0	0	0	0	0	0	1	0	0	0	1	0	4
**1.748**	0	0	0	1	0	0	0	0	0	1	0	1	0	1	0	0	0	0	0	0	0	0	0	1	0	4
**1.749**	0	0	0	1	0	0	0	0	1	0	0	0	0	1	0	0	0	0	0	0	0	1	0	1	0	4
**1.750**	0	0	1	0	0	0	0	0	0	0	0	1	1	0	0	0	0	0	0	1	0	0	0	1	1	4
**1.751**	0	1	0	0	1	0	1	0	1	1	0	1	1	1	0	0	0	0	0	1	0	0	0	1	0	4
**1.752**	0	1	0	1	0	0	1	0	1	1	0	1	0	0	0	0	0	0	0	0	0	0	0	1	1	4
**1.753**	0	1	1	0	0	0	0	0	1	1	0	1	1	0	0	0	0	0	0	1	0	1	0	1	1	4
**1.754**	0	0	0	0	0	0	0	0	1	0	0	1	0	1	0	0	0	0	0	1	0	0	0	1	0	4
**1.755**	0	0	0	0	0	0	0	0	1	0	0	1	1	0	0	0	0	0	0	1	0	1	0	1	0	4
**1.756**	0	0	0	0	0	0	1	0	0	0	0	1	0	0	0	0	0	0	0	1	0	1	0	1	0	4
**1.757**	0	0	0	0	1	0	0	0	0	0	0	1	1	0	0	0	0	0	0	0	0	0	1	1	1	4
**1.758**	0	0	0	1	0	0	0	0	1	0	0	0	0	0	0	0	0	0	0	0	0	1	0	1	1	4
**1.759**	0	1	0	0	0	0	0	0	0	1	0	1	1	0	0	0	0	0	0	1	0	1	0	1	1	4
**1.760**	0	1	0	0	1	0	0	0	1	0	0	0	0	1	0	0	0	0	0	1	0	1	0	1	1	4
**1.761**	0	1	0	1	0	0	0	0	0	0	0	0	1	0	0	0	0	0	0	0	0	0	0	1	0	4
**1.762**	0	1	1	0	1	0	0	0	0	0	0	1	0	0	0	0	0	0	0	1	0	1	0	1	1	4
**1.763**	0	0	0	0	0	0	0	0	0	1	0	1	1	0	0	0	0	0	0	1	0	0	0	1	0	4

	CD2	CD3	CD4	CD7	CD8	CD10	CD13	CD18	CD26	CD29	CD36	CD44	CD45RA	CD49f	CD54	CD56	CD57	CD58	CD62L	CD71	CD80	Cytokeratin	Histone_H1	HLA-DQ	HLA-DR	Freq. (Voxel)
**1.764**	0	0	0	0	0	0	0	0	1	0	0	0	1	0	0	0	0	0	0	1	0	1	0	1	1	4
**1.765**	0	0	0	1	0	0	0	0	0	0	0	1	0	0	0	0	0	0	0	0	0	0	1	1	0	4
**1.766**	0	0	0	1	0	0	0	0	0	0	0	1	0	1	0	0	0	0	0	1	0	0	0	1	0	4
**1.767**	0	0	0	1	1	0	0	0	1	1	0	1	0	0	0	0	0	0	0	1	0	1	0	1	1	4
**1.768**	0	0	1	1	0	0	0	0	1	0	0	1	1	0	0	0	0	0	0	1	0	0	0	1	1	4
**1.769**	0	1	0	0	0	0	0	0	1	0	0	1	1	0	0	0	0	0	0	1	0	0	0	1	1	4
**1.770**	0	1	0	0	0	0	0	0	1	1	0	1	0	0	0	0	0	0	0	0	0	1	1	1	0	4
**1.771**	0	1	0	0	1	0	0	0	0	0	0	0	1	0	0	0	0	0	0	1	0	0	0	1	1	4
**1.772**	0	1	0	1	0	0	0	0	1	1	0	1	0	1	0	0	0	0	0	1	0	1	0	1	1	4
**1.773**	0	1	0	1	1	0	0	0	0	0	0	0	1	0	0	0	0	0	0	0	0	0	0	1	1	4
**1.774**	0	0	0	0	0	0	1	0	0	0	0	0	0	1	0	1	0	0	0	1	0	0	0	1	1	4
**1.775**	0	0	0	0	1	0	1	0	1	0	0	0	0	0	0	0	0	0	0	1	0	0	0	1	1	4
**1.776**	0	0	1	0	0	0	0	0	0	0	0	0	0	0	0	1	0	0	0	0	0	0	1	1	1	4
**1.777**	0	0	1	0	0	0	0	0	1	0	0	0	1	0	0	0	0	0	0	0	0	0	0	1	1	4
**1.778**	0	1	0	0	0	0	0	0	0	0	0	1	1	0	0	0	0	0	0	1	0	1	0	1	1	4
**1.779**	0	1	0	0	0	0	0	0	1	0	0	1	0	0	0	0	0	0	0	1	0	0	1	1	1	4
**1.780**	0	1	0	1	0	0	0	0	0	0	0	1	0	0	0	0	0	0	0	0	0	1	0	1	1	4
**1.781**	0	1	0	1	0	0	0	0	0	0	0	1	0	1	0	0	0	0	0	0	0	1	0	1	1	4
**1.782**	0	1	0	1	0	0	0	0	0	1	0	1	0	0	0	0	0	0	0	0	0	1	0	1	1	4
**1.783**	0	1	1	1	1	0	0	0	0	0	0	1	0	0	0	0	0	0	0	1	0	0	0	1	0	4
**1.784**	0	0	0	0	0	0	0	0	0	0	1	1	0	0	0	0	0	0	0	1	0	1	0	1	0	4
**1.785**	0	0	1	0	0	0	0	0	0	0	0	0	0	1	0	0	0	0	0	1	0	1	0	1	0	4
**1.786**	0	0	1	1	0	0	0	0	0	0	0	0	1	0	0	0	0	0	0	1	0	1	0	1	0	4
**1.787**	0	1	0	0	0	0	0	0	0	1	0	1	0	0	0	0	0	0	0	0	0	1	0	1	0	4
**1.788**	0	1	0	1	0	0	0	0	0	0	1	1	0	0	0	0	0	0	0	1	0	1	0	1	1	4
**1.789**	0	1	0	1	0	0	1	0	0	0	0	1	0	0	0	0	0	0	0	0	0	0	0	1	0	4
**1.790**	0	1	1	0	0	0	0	0	0	1	0	1	0	0	0	0	0	0	0	0	0	0	0	1	0	4
**1.791**	0	1	1	0	1	0	0	0	1	0	0	0	0	0	0	0	0	0	0	0	0	0	0	1	1	4
**1.792**	0	0	0	0	0	0	1	0	0	1	0	0	0	1	0	0	0	0	0	0	0	1	0	1	0	4
**1.793**	0	0	0	0	0	0	1	0	1	0	0	1	0	1	0	0	0	0	0	1	0	0	1	1	1	4
**1.794**	0	0	1	0	0	0	1	0	0	0	0	0	0	1	0	0	0	0	0	0	0	0	0	1	0	4
**1.795**	0	0	1	0	0	0	1	0	0	1	1	1	0	0	0	0	0	0	0	1	0	0	0	1	1	4
**1.796**	0	0	1	0	1	0	1	0	0	0	0	0	1	0	0	1	0	0	0	1	0	0	0	1	0	4
**1.797**	0	1	0	0	0	0	0	0	0	0	0	0	0	0	0	0	0	0	0	1	0	1	0	1	1	4
**1.798**	0	1	0	0	1	0	0	0	0	0	0	1	0	1	0	0	0	0	0	1	0	0	0	1	1	4
**1.799**	0	1	0	0	1	0	0	0	0	0	0	1	1	0	0	0	0	0	0	0	0	1	1	1	0	4
**1.800**	0	1	0	1	1	0	0	0	0	1	0	1	1	1	0	0	0	0	0	0	0	1	0	1	1	4
**1.801**	0	0	1	0	0	0	1	0	0	1	0	1	0	1	0	1	0	0	0	1	0	0	0	1	0	4
**1.802**	0	0	1	1	0	0	1	0	0	1	0	1	0	1	0	1	0	0	0	0	0	0	0	1	1	4
**1.803**	0	1	0	0	0	0	0	0	0	0	0	1	1	0	0	0	0	0	0	0	0	0	1	1	1	4
**1.804**	0	1	0	0	0	0	0	0	0	1	0	1	1	1	0	0	0	0	0	0	0	0	0	1	0	4
**1.805**	0	1	0	0	1	0	0	0	1	0	0	0	1	0	0	0	0	0	0	0	0	0	0	1	0	4
**1.806**	0	0	0	0	0	0	0	0	0	1	0	1	1	1	0	0	0	0	0	1	0	1	0	1	1	4
**1.807**	0	0	0	1	0	0	1	0	0	1	0	1	0	1	0	0	0	0	0	1	0	1	1	1	0	4
**1.808**	0	0	1	0	0	0	0	0	0	0	0	0	0	0	0	1	0	0	0	0	0	1	0	1	1	4
**1.809**	0	0	1	0	0	0	0	0	0	1	0	1	0	1	0	1	0	0	0	1	0	1	0	1	1	4
**1.810**	0	0	1	0	0	0	1	0	1	1	0	1	1	1	0	0	0	0	0	1	0	1	0	1	1	4
**1.811**	0	1	0	0	0	0	1	0	0	1	0	1	0	1	0	0	0	0	0	1	0	1	0	1	1	4
**1.812**	0	1	1	1	1	0	0	0	0	0	0	1	1	0	0	0	0	0	0	1	0	0	0	1	1	4
**1.813**	0	1	1	1	1	0	0	0	1	0	0	0	1	0	0	0	0	0	0	0	0	0	0	1	0	4
**1.814**	0	0	0	0	0	0	0	0	0	0	0	0	0	1	0	1	0	0	0	0	0	1	0	1	0	4
**1.815**	0	0	0	0	0	0	0	0	0	0	0	1	1	1	0	1	0	0	0	0	0	1	0	1	1	4
**1.816**	0	0	0	1	0	0	1	0	1	1	0	1	0	1	0	1	0	0	0	1	0	0	0	1	1	4
**1.817**	0	0	0	1	1	0	0	0	0	0	0	1	1	0	0	0	0	0	0	0	0	0	0	1	0	4
**1.818**	0	0	1	0	1	0	0	0	0	0	0	1	1	0	0	0	0	0	0	0	0	0	0	1	1	4
**1.819**	0	1	0	0	0	0	0	0	0	0	0	0	0	1	0	0	0	0	0	0	0	0	1	1	0	4
**1.820**	0	1	1	1	1	0	0	0	1	1	0	1	1	0	0	0	0	0	0	0	0	0	0	1	0	4
**1.821**	0	0	1	0	0	0	0	0	1	0	0	0	0	0	0	0	0	0	0	1	0	0	0	1	0	4
**1.822**	0	1	0	0	1	0	0	0	1	0	0	1	1	0	0	0	0	0	0	0	0	1	1	1	1	4
**1.823**	0	1	1	0	0	0	0	0	0	1	0	1	0	1	0	0	0	0	0	0	0	1	0	1	1	4
**1.824**	0	1	1	0	1	0	0	0	1	1	0	1	0	0	0	0	0	0	0	1	0	0	0	1	0	4
**1.825**	0	0	0	0	1	0	0	0	0	0	0	1	0	0	0	0	0	0	0	1	0	0	1	1	0	4
**1.826**	0	0	1	0	0	0	1	0	0	0	0	1	0	1	0	0	0	0	0	1	0	1	0	1	0	4
**1.827**	0	0	0	0	0	0	0	0	0	0	1	0	0	1	0	0	0	0	0	1	0	0	0	1	1	4
**1.828**	0	0	0	0	0	0	1	0	1	0	0	1	0	1	0	0	0	0	0	1	0	1	0	1	0	4
**1.829**	0	0	1	0	0	0	1	0	1	1	0	0	0	1	0	0	0	0	0	0	0	0	0	1	1	4
**1.830**	0	0	0	0	0	0	0	0	0	1	0	1	0	1	0	1	0	0	0	0	0	1	0	1	0	4
**1.831**	0	0	0	0	0	0	1	0	0	0	0	1	0	1	0	0	0	0	0	1	0	0	0	1	0	4
**1.832**	0	0	1	0	0	0	0	0	0	1	0	1	0	0	0	1	0	0	0	0	0	0	0	1	1	4
**1.833**	0	0	1	1	0	0	0	0	0	1	0	0	0	1	0	0	0	0	0	0	0	0	0	1	1	4
**1.834**	0	1	0	0	0	0	0	0	1	0	0	1	0	0	0	0	0	0	0	1	0	0	1	1	0	4
**1.835**	0	0	0	0	0	0	0	0	1	1	1	1	0	0	0	0	0	0	0	0	0	0	0	1	1	4
**1.836**	0	0	0	0	0	0	1	0	0	1	0	1	0	0	0	1	0	0	0	0	0	0	0	1	1	4
**1.837**	0	0	1	0	0	0	0	0	0	1	0	0	1	1	0	0	0	0	0	1	0	0	0	1	1	4
**1.838**	0	1	1	0	0	0	0	0	1	0	0	1	0	0	0	0	0	0	0	1	0	1	0	1	1	3
**1.839**	0	1	0	1	0	0	1	0	0	1	0	1	0	1	0	0	0	0	0	0	0	0	0	1	1	3
**1.840**	0	0	1	0	0	0	1	0	0	0	0	0	0	0	0	0	0	0	0	0	0	0	1	1	0	3
**1.841**	0	0	0	0	0	0	0	0	1	0	0	0	0	0	0	0	0	0	0	1	0	1	1	1	1	3
**1.842**	0	0	0	0	0	0	1	0	0	0	0	0	0	0	0	0	0	0	0	0	0	1	1	1	1	3
**1.843**	0	0	0	0	0	0	1	0	0	0	0	0	0	0	0	1	0	0	0	0	0	1	0	1	0	3
**1.844**	0	0	0	0	0	0	1	0	0	0	0	1	0	0	0	1	0	0	0	0	0	1	0	1	0	3
**1.845**	0	0	0	0	1	0	0	0	1	0	0	1	0	1	0	0	0	0	0	0	0	0	0	1	1	3
**1.846**	0	0	0	1	1	0	0	0	0	0	0	1	0	0	0	0	0	0	0	1	0	1	0	1	1	3
**1.847**	0	0	1	0	0	0	0	0	1	0	0	0	0	1	0	0	0	0	0	1	0	0	0	1	1	3
**1.848**	0	0	1	1	0	0	0	0	1	0	0	1	0	0	0	0	0	0	0	1	0	0	0	1	1	3
**1.849**	0	0	1	1	1	0	1	0	0	0	0	1	0	0	0	0	0	0	0	1	0	0	0	1	1	3
**1.850**	0	1	0	0	1	0	0	0	0	1	0	1	0	1	0	0	0	0	0	1	0	1	0	1	0	3
**1.851**	0	1	0	1	1	0	0	0	1	1	0	1	1	1	0	0	0	0	0	0	0	0	0	1	0	3
**1.852**	0	1	0	1	1	0	1	0	1	0	0	1	0	0	0	0	0	0	0	0	0	0	1	1	0	3
**1.853**	0	1	1	1	1	0	1	0	1	1	0	1	1	0	0	0	0	0	0	1	0	0	0	1	1	3
**1.854**	0	0	0	0	0	0	1	0	0	0	0	1	1	0	0	1	0	0	0	0	0	1	0	1	0	3
**1.855**	0	0	0	1	0	0	0	0	0	0	0	1	1	0	0	0	0	0	0	0	0	0	0	1	0	3
**1.856**	0	0	0	1	0	0	0	0	0	1	0	1	1	0	0	0	0	0	0	0	0	0	0	1	0	3
**1.857**	0	0	0	1	0	0	0	0	1	0	0	1	1	0	0	0	0	0	0	1	0	0	0	1	1	3
**1.858**	0	0	0	1	0	0	0	0	1	1	0	1	1	0	0	0	0	0	0	1	0	1	1	1	0	3
**1.859**	0	0	0	1	0	0	1	0	1	1	0	1	0	0	0	0	0	0	0	0	0	0	0	1	1	3
**1.860**	0	0	1	0	1	0	1	0	0	1	0	1	0	0	0	0	0	0	0	1	0	0	0	1	1	3
**1.861**	0	1	0	1	0	0	0	0	0	0	0	1	1	0	0	0	0	0	0	0	0	0	0	1	0	3

	CD2	CD3	CD4	CD7	CD8	CD10	CD13	CD18	CD26	CD29	CD36	CD44	CD45RA	CD49f	CD54	CD56	CD57	CD58	CD62L	CD71	CD80	Cytokeratin	Histone_H1	HLA-DQ	HLA-DR	Freq. (Voxel)
**1.862**	0	1	0	1	0	0	0	0	0	1	0	1	1	0	0	0	0	0	0	1	0	0	0	1	1	3
**1.863**	0	1	0	1	1	0	0	0	0	1	0	1	1	1	0	0	0	0	0	1	0	1	0	1	1	3
**1.864**	0	1	1	0	1	0	0	0	1	0	0	1	1	0	0	0	0	0	0	0	0	1	0	1	1	3
**1.865**	0	0	0	0	0	0	0	0	0	0	0	1	1	0	0	1	0	0	0	0	0	1	0	1	0	3
**1.866**	0	0	0	0	0	0	0	0	0	1	0	0	1	1	0	1	0	0	0	0	0	0	1	1	0	3
**1.867**	0	0	1	0	0	0	0	0	1	0	0	1	1	0	0	0	0	0	0	1	0	0	0	1	1	3
**1.868**	0	1	0	1	0	0	0	0	0	0	0	0	1	0	0	0	0	0	0	0	0	0	1	1	0	3
**1.869**	0	1	1	0	0	0	0	0	1	0	0	1	1	0	0	0	0	0	0	1	0	0	0	1	1	3
**1.870**	0	1	0	0	0	0	0	0	0	1	0	0	0	0	0	0	0	0	0	0	0	0	0	1	1	3
**1.871**	0	1	0	1	0	0	0	0	0	1	0	1	0	1	0	0	0	0	0	1	0	1	0	1	1	3
**1.872**	0	1	0	1	0	0	1	0	0	1	0	1	0	0	0	0	0	0	0	0	0	0	0	1	1	3
**1.873**	0	1	1	0	0	0	0	0	1	0	0	1	0	0	0	1	0	0	0	1	0	0	0	1	1	3
**1.874**	0	0	1	0	0	0	1	0	1	0	0	1	0	1	0	0	0	0	0	1	0	1	0	1	0	3
**1.875**	0	1	0	1	1	0	0	0	0	0	0	0	1	0	0	0	0	0	0	0	0	0	1	1	1	3
**1.876**	0	1	0	1	1	0	0	0	0	1	0	0	1	0	0	0	0	0	0	0	0	0	1	1	1	3
**1.877**	0	0	1	0	0	0	0	0	0	1	0	0	0	0	0	0	0	0	0	0	0	0	1	1	1	3
**1.878**	0	0	1	0	0	0	1	0	0	0	1	1	0	1	0	0	0	0	0	0	0	0	0	1	1	3
**1.879**	0	0	1	0	0	0	1	0	1	1	0	0	0	1	0	0	0	0	0	0	0	0	0	1	0	3
**1.880**	0	0	1	1	0	0	0	0	0	0	0	0	1	1	0	1	0	0	0	0	0	0	0	1	0	3
**1.881**	0	0	1	1	0	0	1	0	0	1	0	1	0	1	0	0	0	0	0	1	0	1	0	1	0	3
**1.882**	0	1	0	1	0	0	0	0	0	0	0	0	1	1	0	0	0	0	0	0	0	0	0	1	1	3
**1.883**	0	0	1	0	0	0	0	0	1	1	0	0	0	1	0	0	0	0	0	0	0	0	0	1	0	3
**1.884**	0	1	0	0	0	0	0	0	0	1	0	1	0	1	0	0	0	0	0	1	0	0	1	1	1	3
**1.885**	0	1	0	1	0	0	0	0	0	0	0	1	0	1	0	0	0	0	0	0	0	0	1	1	1	3
**1.886**	0	1	1	0	1	0	0	0	0	1	0	1	1	0	0	0	0	0	0	0	0	0	1	1	0	3
**1.887**	0	0	0	0	0	0	0	0	0	0	0	0	0	0	0	1	0	0	0	0	0	1	0	1	1	3
**1.888**	0	0	1	1	0	0	1	0	1	1	1	1	0	1	0	0	0	0	0	1	0	1	0	1	1	3
**1.889**	0	1	1	0	1	0	0	0	1	1	0	1	1	0	0	0	0	0	0	0	0	0	1	1	1	3
**1.890**	0	0	0	0	0	0	0	0	0	0	1	0	0	0	0	1	0	0	0	0	0	0	0	1	1	3
**1.891**	0	0	0	0	0	0	1	0	0	1	0	1	1	0	0	0	0	0	0	0	0	0	0	1	1	3
**1.892**	0	0	0	0	1	0	0	0	0	1	0	1	0	0	0	0	0	0	0	1	0	0	1	1	1	3
**1.893**	0	0	0	1	0	0	1	0	0	1	0	1	0	1	0	0	0	0	0	0	0	0	0	1	0	3
**1.894**	0	0	1	0	0	0	0	0	0	1	0	0	1	1	0	0	0	0	0	0	0	1	0	1	1	3
**1.895**	0	0	1	0	0	0	1	0	1	1	0	1	1	1	0	1	0	0	0	1	0	0	1	1	1	3
**1.896**	0	1	1	0	1	0	0	0	1	1	0	0	1	0	0	0	0	0	0	0	0	0	0	1	0	3
**1.897**	0	0	0	0	0	0	0	0	0	0	1	0	0	1	0	0	0	0	0	0	0	0	1	1	0	3
**1.898**	0	0	1	0	0	0	0	0	0	0	0	0	0	1	0	0	0	0	0	0	0	1	1	1	0	3
**1.899**	0	1	1	0	1	0	0	0	1	1	0	0	0	0	0	0	0	0	0	1	0	0	1	1	0	3
**1.900**	0	0	1	0	0	0	0	0	1	1	0	0	0	1	0	0	0	0	0	1	0	0	0	1	1	3
**1.901**	0	0	0	1	0	0	1	0	0	1	0	1	1	1	0	1	0	0	0	1	0	0	0	1	1	3
**1.902**	0	0	1	0	0	0	0	0	0	0	0	1	0	0	0	1	0	0	0	1	0	0	0	1	1	3
**1.903**	0	0	1	1	0	0	0	0	0	1	0	1	0	0	0	0	0	0	0	0	0	0	0	1	1	3
**1.904**	0	0	0	0	0	0	1	0	1	0	0	0	1	0	0	1	0	0	0	1	0	1	0	1	0	3
**1.905**	0	0	0	0	0	0	1	0	1	1	0	1	0	0	0	0	0	0	0	1	0	1	0	1	1	3
**1.906**	0	0	0	0	1	0	0	0	1	0	0	1	1	0	0	0	0	0	0	1	0	0	1	1	1	3
**1.907**	0	0	0	0	1	0	0	0	1	0	0	1	1	1	0	0	0	0	0	0	0	0	0	1	1	3
**1.908**	0	0	0	0	1	0	0	0	1	1	0	1	1	0	0	0	0	0	0	1	0	0	0	1	1	3
**1.909**	0	0	0	1	0	0	0	0	1	1	0	1	1	1	0	0	0	0	0	1	0	0	0	1	1	3
**1.910**	0	0	0	1	0	0	1	0	0	0	0	0	1	0	0	1	0	0	0	1	0	0	0	1	0	3
**1.911**	0	0	0	1	1	0	0	0	0	0	0	0	1	0	0	0	0	0	0	1	0	0	0	1	1	3
**1.912**	0	0	0	1	1	0	0	0	0	0	0	1	0	0	0	0	0	0	0	0	0	1	1	1	0	3
**1.913**	0	0	0	1	1	0	0	0	1	0	0	1	1	1	0	0	0	0	0	0	0	0	0	1	1	3
**1.914**	0	0	1	0	0	0	1	0	1	0	0	0	0	0	0	0	0	0	0	1	0	1	0	1	1	3
**1.915**	0	1	0	0	0	0	0	0	1	0	0	1	1	0	0	0	0	0	0	1	0	0	1	1	1	3
**1.916**	0	1	0	0	1	0	1	0	1	0	0	1	0	0	0	0	0	0	0	1	0	1	0	1	1	3
**1.917**	0	1	0	1	1	0	0	0	1	0	0	0	0	0	0	0	0	0	0	0	0	0	1	1	0	3
**1.918**	0	1	0	1	1	0	0	0	1	0	0	1	1	0	0	0	0	0	0	0	0	1	0	1	0	3
**1.919**	0	1	1	0	1	0	1	0	1	0	0	1	1	1	0	0	0	0	0	1	0	1	0	1	1	3
**1.920**	0	1	1	0	1	0	1	0	1	1	0	1	1	0	0	0	0	0	0	1	0	0	1	1	0	3
**1.921**	0	1	1	1	1	0	1	0	1	0	0	1	0	0	0	0	0	0	0	1	0	1	0	1	1	3
**1.922**	0	1	1	1	1	0	1	0	1	1	0	1	0	0	0	0	0	0	0	1	0	1	0	1	1	3
**1.923**	0	1	1	1	1	0	1	0	1	1	0	1	1	0	0	0	0	0	0	1	0	0	0	1	0	3
**1.924**	0	0	0	0	0	0	0	0	1	0	0	1	0	0	0	1	0	0	0	0	0	0	0	1	0	3
**1.925**	0	0	0	0	0	0	1	0	0	1	0	1	0	0	0	0	0	0	0	1	0	1	0	1	0	3
**1.926**	0	0	0	0	1	0	0	0	1	1	0	1	0	0	0	0	0	0	0	1	0	0	1	1	0	3
**1.927**	0	0	0	0	1	0	0	0	1	1	0	1	0	0	0	0	0	0	0	1	0	1	0	1	0	3
**1.928**	0	0	0	0	1	0	0	0	1	1	0	1	0	1	0	0	0	0	0	0	0	0	0	1	1	3
**1.929**	0	0	0	0	1	0	0	0	1	1	0	1	1	0	0	0	0	0	0	0	0	1	0	1	1	3
**1.930**	0	0	0	1	0	0	0	0	0	1	0	0	0	0	0	0	0	0	0	0	0	1	0	1	1	3
**1.931**	0	0	0	1	0	0	0	0	0	1	0	0	0	0	0	0	0	0	0	1	0	0	0	1	0	3
**1.932**	0	0	0	1	0	0	0	0	0	1	0	1	1	1	0	0	0	0	0	1	0	0	0	1	0	3
**1.933**	0	0	0	1	0	0	0	0	1	0	0	0	0	0	0	0	0	0	0	0	0	1	0	1	0	3
**1.934**	0	0	0	1	0	0	0	0	1	0	0	1	0	0	0	0	0	0	0	1	0	1	0	1	1	3
**1.935**	0	0	0	1	1	0	0	0	0	0	0	0	0	0	0	0	0	0	0	0	0	0	1	1	1	3
**1.936**	0	1	0	0	0	0	0	0	1	0	0	1	0	1	0	0	0	0	0	0	0	1	1	1	1	3
**1.937**	0	1	0	0	0	0	0	0	1	0	0	1	1	1	0	0	0	0	0	1	0	1	1	1	1	3
**1.938**	0	1	0	0	0	0	1	0	1	0	0	1	0	0	0	0	0	0	0	1	0	0	0	1	1	3
**1.939**	0	1	0	0	1	0	0	0	1	0	0	1	0	0	0	0	0	0	0	1	0	0	1	1	0	3
**1.940**	0	1	0	0	1	0	0	0	1	1	0	0	0	0	0	0	0	0	0	1	0	1	0	1	1	3
**1.941**	0	1	0	0	1	0	0	0	1	1	0	1	1	0	0	0	0	0	0	0	0	0	1	1	0	3
**1.942**	0	1	0	0	1	0	1	0	0	1	0	1	0	0	0	0	0	0	0	0	0	0	0	1	0	3
**1.943**	0	1	0	1	1	0	1	0	1	0	0	1	1	0	0	0	0	0	0	1	0	1	0	1	1	3
**1.944**	0	1	1	0	1	0	0	0	1	0	0	1	0	0	0	0	0	0	0	0	0	1	0	1	1	3
**1.945**	0	1	1	0	1	0	0	0	1	0	0	1	0	1	0	0	0	0	0	1	0	1	0	1	1	3
**1.946**	0	1	1	0	1	0	1	0	1	1	0	1	1	0	0	0	0	0	0	1	0	0	0	1	0	3
**1.947**	0	0	0	0	0	0	0	0	0	1	0	1	0	0	0	0	0	0	0	1	0	1	1	1	1	3
**1.948**	0	0	0	0	0	0	0	0	1	0	0	1	1	1	0	0	0	0	0	0	0	1	0	1	1	3
**1.949**	0	0	0	0	0	0	1	0	1	1	0	1	0	0	0	0	0	0	0	0	0	0	1	1	1	3
**1.950**	0	0	0	0	1	0	0	0	0	0	0	0	0	0	0	0	0	0	0	1	0	1	0	1	1	3
**1.951**	0	0	0	0	1	0	0	0	0	0	0	1	0	0	0	0	0	0	0	1	0	1	0	1	0	3
**1.952**	0	0	0	0	1	0	0	0	0	0	0	1	1	0	0	0	0	0	0	0	0	1	0	1	0	3
**1.953**	0	0	0	1	0	0	0	0	0	0	0	0	1	0	0	0	0	0	0	1	0	0	0	1	1	3
**1.954**	0	0	0	1	0	0	0	0	0	0	0	1	0	0	0	0	0	0	0	0	0	1	1	1	1	3
**1.955**	0	0	0	1	0	0	0	0	1	0	0	0	1	0	0	0	0	0	0	1	0	0	0	1	1	3
**1.956**	0	0	0	1	0	0	0	0	1	0	0	1	0	0	0	0	0	0	0	1	0	1	0	1	0	3
**1.957**	0	0	0	1	1	0	1	0	0	0	0	0	0	0	0	0	0	0	0	0	0	0	1	1	0	3
**1.958**	0	0	1	0	1	0	0	0	1	0	0	1	0	0	0	0	0	0	0	0	0	0	1	1	1	3
**1.959**	0	0	1	1	0	0	0	0	1	0	0	1	0	0	0	0	0	0	0	1	0	1	0	1	1	3

	CD2	CD3	CD4	CD7	CD8	CD10	CD13	CD18	CD26	CD29	CD36	CD44	CD45RA	CD49f	CD54	CD56	CD57	CD58	CD62L	CD71	CD80	Cytokeratin	Histone_H1	HLA-DQ	HLA-DR	Freq. (Voxel)
**1.960**	0	1	0	0	1	0	1	0	0	0	0	1	0	0	0	0	0	0	0	1	0	0	1	1	1	3
**1.961**	0	1	0	0	1	0	1	0	1	0	0	1	0	0	0	0	0	0	0	1	0	0	1	1	1	3
**1.962**	0	1	0	1	0	0	1	0	1	0	0	1	0	0	0	0	0	0	0	0	0	0	0	1	1	3
**1.963**	0	1	0	1	1	0	1	0	0	1	0	1	1	0	0	0	0	0	0	1	0	1	0	1	0	3
**1.964**	0	1	1	0	1	0	1	0	1	1	0	1	1	0	0	0	0	0	0	1	0	0	0	1	1	3
**1.965**	0	0	0	0	0	0	1	0	0	0	0	0	0	1	0	0	0	0	0	1	0	0	1	1	1	3
**1.966**	0	0	0	0	1	0	0	0	0	0	0	1	0	1	0	0	0	0	0	0	0	1	0	1	1	3
**1.967**	0	0	0	1	0	0	0	0	0	0	0	1	0	0	0	1	0	0	0	1	0	0	0	1	1	3
**1.968**	0	0	0	1	0	0	0	0	0	0	0	1	1	0	0	0	0	0	0	0	0	0	0	1	1	3
**1.969**	0	0	0	1	0	0	0	0	0	1	0	1	0	1	0	0	0	0	0	1	0	1	1	1	0	3
**1.970**	0	0	0	1	0	0	0	0	0	1	0	1	1	1	0	1	0	0	0	1	0	1	1	1	0	3
**1.971**	0	0	0	1	0	0	0	0	1	0	0	1	0	0	0	1	0	0	0	1	0	0	0	1	1	3
**1.972**	0	0	0	1	0	0	1	0	1	0	0	1	0	0	0	1	0	0	0	1	0	0	0	1	1	3
**1.973**	0	0	1	0	0	0	0	0	1	0	0	0	0	1	0	0	0	0	0	1	0	1	0	1	1	3
**1.974**	0	0	1	0	1	0	1	0	0	1	0	1	0	0	0	0	0	0	0	0	0	0	0	1	1	3
**1.975**	0	0	1	1	0	0	0	0	0	0	0	1	1	0	0	0	0	0	0	0	0	0	0	1	1	3
**1.976**	0	0	1	1	0	0	0	0	0	1	0	0	1	0	0	0	0	0	0	1	0	0	0	1	1	3
**1.977**	0	1	0	0	0	0	0	0	0	0	0	0	0	0	0	0	0	0	0	1	0	0	1	1	1	3
**1.978**	0	1	0	0	0	0	0	0	0	0	0	1	1	0	0	0	0	0	0	1	0	1	1	1	0	3
**1.979**	0	1	0	0	0	0	0	0	0	1	0	1	1	0	0	0	0	0	0	1	0	1	0	1	0	3
**1.980**	0	1	0	0	0	0	0	0	1	0	0	0	0	0	0	0	0	0	0	1	0	1	1	1	1	3
**1.981**	0	1	0	0	1	0	0	0	1	1	0	1	0	1	0	0	0	0	0	1	0	1	0	1	1	3
**1.982**	0	1	0	1	0	0	0	0	0	0	0	1	1	1	0	0	0	0	0	1	0	1	0	1	1	3
**1.983**	0	1	0	1	0	0	0	0	1	0	0	1	1	1	0	0	0	0	0	1	0	1	0	1	1	3
**1.984**	0	1	0	1	0	0	1	0	0	0	0	1	0	1	0	0	0	0	0	1	0	0	0	1	1	3
**1.985**	0	1	0	1	0	0	1	0	1	0	0	1	0	0	0	1	0	0	0	1	0	0	0	1	1	3
**1.986**	0	1	0	1	1	0	0	0	0	1	0	1	1	0	0	0	0	0	0	1	0	0	1	1	0	3
**1.987**	0	1	1	0	0	0	0	0	0	1	0	1	0	0	0	0	0	0	0	0	0	1	1	1	1	3
**1.988**	0	1	1	1	1	0	0	0	0	0	0	1	1	0	0	0	0	0	0	1	0	1	0	1	1	3
**1.989**	0	0	0	0	0	0	0	0	0	0	0	0	1	0	0	1	0	0	0	0	0	1	0	1	1	3
**1.990**	0	0	0	0	0	0	0	0	0	0	0	0	1	1	0	0	0	0	0	1	0	0	0	1	1	3
**1.991**	0	0	0	0	0	0	0	0	0	1	0	0	1	1	0	0	0	0	0	1	0	0	0	1	0	3
**1.992**	0	0	0	0	0	0	1	0	1	1	0	1	0	0	0	0	0	0	0	0	0	0	0	1	0	3
**1.993**	0	0	0	0	1	0	1	0	0	1	0	1	0	1	0	0	0	0	0	1	0	0	0	1	0	3
**1.994**	0	0	0	1	0	0	0	0	0	1	0	1	0	0	0	0	0	0	0	0	0	1	0	1	1	3
**1.995**	0	0	1	0	0	0	0	0	0	1	0	0	1	0	0	0	0	0	0	0	0	0	0	1	0	3
**1.996**	0	0	1	0	0	0	0	0	0	1	0	0	1	1	0	0	0	0	0	1	0	0	0	1	0	3
**1.997**	0	0	1	0	1	0	0	0	0	1	0	0	0	0	0	0	0	0	0	1	0	0	0	1	1	3
**1.998**	0	0	1	0	1	0	1	0	0	0	0	1	0	0	0	0	0	0	0	1	0	0	0	1	1	3
**1.999**	0	1	0	0	0	0	0	0	0	1	0	1	1	0	0	0	0	0	0	0	0	1	1	1	0	3
**2.000**	0	1	0	0	1	0	0	0	1	0	0	1	1	0	0	0	0	0	0	1	0	1	1	1	0	3
**2.001**	0	1	0	1	0	0	0	0	0	0	0	0	0	1	0	0	0	0	0	1	0	1	0	1	1	3
**2.002**	0	1	0	1	0	0	0	0	0	0	1	1	0	1	0	0	0	0	0	1	0	1	0	1	1	3
**2.003**	0	1	0	1	1	0	0	0	0	0	0	1	1	0	0	0	0	0	0	0	0	1	1	1	1	3
**2.004**	0	1	0	1	1	0	0	0	0	0	0	1	1	1	0	0	0	0	0	1	0	1	0	1	1	3
**2.005**	0	1	0	1	1	0	0	0	0	1	0	1	1	0	0	0	0	0	0	1	0	0	1	1	1	3
**2.006**	0	1	1	0	0	0	0	0	0	0	0	0	0	0	0	0	0	0	0	0	0	1	1	1	1	3
**2.007**	0	1	1	0	1	0	0	0	1	0	0	0	0	0	0	0	0	0	0	1	0	0	0	1	1	3
**2.008**	0	1	1	1	0	0	0	0	0	1	0	1	0	1	0	0	0	0	0	1	0	0	0	1	1	3
**2.009**	0	0	0	0	1	0	0	0	1	0	0	0	0	0	0	0	0	0	0	1	0	0	0	1	1	3
**2.010**	0	0	1	0	0	0	0	0	1	0	0	1	0	1	0	0	0	0	0	0	0	1	0	1	1	3
**2.011**	0	1	0	1	0	0	0	0	0	0	0	0	0	0	0	0	0	0	0	1	0	0	1	1	1	3
**2.012**	0	1	0	1	0	0	0	0	0	0	0	0	1	1	0	0	0	0	0	0	0	1	0	1	0	3
**2.013**	0	1	1	0	1	0	0	0	0	1	0	1	1	1	0	0	0	0	0	1	0	0	0	1	1	3
**2.014**	0	1	1	1	0	0	0	0	1	0	0	1	0	0	0	0	0	0	0	1	0	0	0	1	1	3
**2.015**	0	0	0	0	0	0	0	0	0	0	0	0	1	1	0	0	0	0	0	1	0	0	0	1	0	3
**2.016**	0	0	0	0	0	0	1	0	0	0	1	1	0	1	0	0	0	0	0	1	0	1	0	1	1	3
**2.017**	0	0	0	0	1	0	0	0	0	1	0	0	0	0	0	0	0	0	0	0	0	0	1	1	1	3
**2.018**	0	0	0	1	0	0	1	0	1	1	0	1	0	1	0	0	0	0	0	1	0	1	0	1	1	3
**2.019**	0	0	1	0	0	0	0	0	0	0	0	1	0	1	0	0	0	0	0	0	0	0	1	1	0	3
**2.020**	0	0	1	0	0	0	0	0	0	0	1	1	0	1	0	0	0	0	0	1	0	1	0	1	0	3
**2.021**	0	0	1	0	0	0	1	0	0	0	0	0	1	0	0	1	0	0	0	1	0	0	0	1	0	3
**2.022**	0	1	0	0	0	0	0	0	0	1	0	1	1	0	0	0	0	0	0	0	0	0	0	1	1	3
**2.023**	0	1	0	1	0	0	0	0	0	0	0	0	0	0	0	0	0	0	0	1	0	1	0	1	1	3
**2.024**	0	1	0	1	1	0	0	0	0	0	0	1	0	0	0	0	0	0	0	0	0	1	1	1	0	3
**2.025**	0	1	0	1	1	0	0	0	0	1	1	1	1	1	0	0	0	0	0	0	0	1	0	1	1	3
**2.026**	0	1	1	0	1	0	0	0	0	1	0	1	0	1	0	0	0	0	0	1	0	0	0	1	0	3
**2.027**	0	1	1	0	1	0	0	0	0	1	0	1	1	1	0	0	0	0	0	0	0	0	0	1	0	3
**2.028**	0	1	1	0	1	0	0	0	1	1	0	1	1	1	0	0	0	0	0	1	0	0	0	1	1	3
**2.029**	0	1	1	1	0	0	0	0	1	0	0	1	1	0	0	0	0	0	0	1	0	0	0	1	0	3
**2.030**	0	0	0	1	0	0	1	0	1	1	0	1	0	1	0	0	0	0	0	0	0	1	0	1	1	3
**2.031**	0	0	1	0	0	0	1	0	1	0	0	1	0	0	0	1	0	0	0	0	0	0	0	1	1	3
**2.032**	0	0	1	0	1	0	0	0	0	0	0	1	1	0	0	0	0	0	0	0	0	0	0	1	0	3
**2.033**	0	0	1	0	1	0	1	0	0	0	0	0	0	0	0	0	0	0	0	0	0	0	0	1	0	3
**2.034**	0	0	1	0	1	0	1	0	0	0	0	0	1	0	0	0	0	0	0	0	0	0	0	1	0	3
**2.035**	0	1	0	0	0	0	0	0	0	0	1	1	0	1	0	0	0	0	0	0	0	1	0	1	1	3
**2.036**	0	1	0	0	0	0	0	0	0	1	0	1	0	1	0	0	0	0	0	1	0	1	0	1	1	3
**2.037**	0	1	1	0	1	0	0	0	1	0	0	1	1	0	0	0	0	0	0	0	0	0	0	1	0	3
**2.038**	0	1	1	1	1	0	0	0	1	0	0	1	1	0	0	0	0	0	0	0	0	0	0	1	0	3
**2.039**	0	0	0	0	0	0	0	0	0	1	1	1	0	1	0	0	0	0	0	0	0	1	0	1	0	3
**2.040**	0	0	0	0	0	0	0	0	1	0	1	0	0	0	0	0	0	0	0	0	0	1	0	1	1	3
**2.041**	0	0	0	0	0	0	0	0	1	1	0	0	0	1	0	0	0	0	0	0	0	1	0	1	1	3
**2.042**	0	0	0	1	1	0	0	0	1	0	0	1	1	0	0	0	0	0	0	0	0	0	0	1	0	3
**2.043**	0	0	1	0	0	0	1	0	1	1	0	1	0	1	0	1	0	0	0	1	0	1	0	1	1	3
**2.044**	0	0	1	1	0	0	0	0	0	0	0	1	0	1	0	0	0	0	0	1	0	1	0	1	1	3
**2.045**	0	0	1	1	0	0	1	0	0	1	0	1	0	1	0	0	0	0	0	1	0	0	0	1	0	3
**2.046**	0	0	1	1	0	0	1	0	1	1	1	1	1	1	0	0	0	0	0	1	0	1	0	1	1	3
**2.047**	0	1	0	0	0	0	0	0	0	0	0	0	1	1	0	0	0	0	0	0	0	0	0	1	0	3
**2.048**	0	1	0	0	0	0	1	0	0	0	0	0	0	0	0	0	0	0	0	0	0	1	1	1	0	3
**2.049**	0	1	0	0	1	0	0	0	0	0	0	1	1	0	0	0	0	0	0	1	0	1	1	1	0	3
**2.050**	0	1	0	1	1	0	0	0	0	1	1	1	0	1	0	0	0	0	0	0	0	0	0	1	1	3
**2.051**	0	1	0	1	1	0	0	0	0	1	1	1	1	1	0	0	0	0	0	0	0	1	1	1	1	3
**2.052**	0	1	1	0	1	0	0	0	1	1	0	1	0	0	0	0	0	0	0	0	0	0	1	1	1	3
**2.053**	0	0	0	0	0	0	0	0	1	0	0	0	0	0	0	1	0	0	0	1	0	0	0	1	1	3
**2.054**	0	0	0	0	0	0	0	0	1	0	0	1	0	0	0	1	0	0	0	0	0	0	1	1	1	3
**2.055**	0	0	1	0	0	0	0	0	0	1	0	0	1	0	0	1	0	0	0	0	0	0	0	1	0	3
**2.056**	0	0	1	0	1	0	0	0	1	0	0	1	1	0	0	0	0	0	0	0	0	0	0	1	1	3
**2.057**	0	1	0	0	0	0	0	0	0	1	0	1	0	1	0	0	0	0	0	0	0	0	1	1	0	3

	CD2	CD3	CD4	CD7	CD8	CD10	CD13	CD18	CD26	CD29	CD36	CD44	CD45RA	CD49f	CD54	CD56	CD57	CD58	CD62L	CD71	CD80	Cytokeratin	Histone_H1	HLA-DQ	HLA-DR	Freq. (Voxel)
**2.058**	0	1	0	0	1	0	0	0	0	1	0	1	0	1	0	0	0	0	0	0	0	0	1	1	0	3
**2.059**	0	0	0	0	0	0	1	0	0	1	0	1	1	1	0	0	0	0	0	1	0	0	1	1	1	3
**2.060**	0	0	0	0	0	0	1	0	1	1	0	1	0	1	0	1	0	0	0	0	0	0	1	1	1	3
**2.061**	0	0	0	0	0	0	1	0	1	1	1	1	0	1	0	0	0	0	0	0	0	0	0	1	1	3
**2.062**	0	0	0	1	0	0	0	0	0	0	0	0	1	0	0	1	0	0	0	0	0	0	0	1	1	3
**2.063**	0	0	1	0	0	0	0	0	1	0	0	0	1	0	0	1	0	0	0	1	0	0	0	1	0	3
**2.064**	0	0	1	0	0	0	1	0	1	1	0	0	0	0	0	0	0	0	0	0	0	0	0	1	0	3
**2.065**	0	1	0	0	0	0	0	0	1	0	0	1	0	0	0	1	0	0	0	0	0	0	0	1	0	3
**2.066**	0	1	1	1	0	0	0	0	1	0	0	1	1	0	0	0	0	0	0	0	0	0	0	1	0	3
**2.067**	0	1	1	1	1	0	0	0	0	0	0	1	0	0	0	0	0	0	0	0	0	0	0	1	0	3
**2.068**	0	0	0	0	0	0	1	0	0	0	0	1	0	1	0	0	0	0	0	1	0	1	1	1	0	3
**2.069**	0	0	0	0	1	0	0	0	1	0	0	0	0	0	0	0	0	0	0	0	0	0	0	1	0	3
**2.070**	0	0	0	1	0	0	1	0	1	1	1	1	0	1	0	0	0	0	0	1	0	0	0	1	1	3
**2.071**	0	1	0	0	1	0	0	0	0	1	1	1	0	1	0	0	0	0	0	0	0	0	0	1	0	3
**2.072**	0	0	0	0	0	0	0	0	0	0	0	0	0	1	0	0	0	0	0	1	0	0	1	1	0	3
**2.073**	0	0	1	0	0	0	0	0	1	1	0	0	0	0	0	0	0	0	0	0	0	0	0	1	1	3
**2.074**	0	0	1	0	0	0	0	0	1	1	1	0	0	1	0	0	0	0	0	0	0	0	0	1	1	3
**2.075**	0	0	1	0	0	0	1	0	0	1	1	1	0	1	0	1	0	0	0	1	0	0	0	1	1	3
**2.076**	0	1	0	0	1	0	1	0	0	1	0	1	0	1	0	0	0	0	0	0	0	0	0	1	1	3
**2.077**	0	0	0	0	0	0	0	0	0	1	0	0	0	0	0	0	0	0	0	1	0	1	1	1	0	3
**2.078**	0	0	0	0	0	0	0	0	0	1	1	0	0	1	0	0	0	0	0	1	0	0	0	1	1	3
**2.079**	0	0	0	0	1	0	1	0	1	0	0	0	1	0	0	0	0	0	0	1	0	0	0	1	0	3
**2.080**	0	0	1	0	0	0	0	0	0	1	0	0	0	0	0	1	0	0	0	0	0	0	0	1	0	3
**2.081**	0	0	1	0	0	0	0	0	1	1	0	1	0	0	0	1	0	0	0	0	0	0	0	1	1	3
**2.082**	0	0	1	0	0	0	1	0	0	1	0	1	0	0	0	1	0	0	0	1	0	0	0	1	1	3
**2.083**	0	0	1	1	0	0	0	0	0	0	0	1	0	0	0	0	0	0	0	0	0	0	0	1	1	3
**2.084**	0	1	0	0	0	0	0	0	0	0	0	0	1	0	0	0	0	0	0	0	0	1	0	1	0	3
**2.085**	0	0	0	0	0	0	0	0	1	0	1	0	0	0	0	0	0	0	0	0	0	1	1	1	1	3
**2.086**	0	0	0	0	1	0	1	0	0	0	0	0	1	0	0	1	0	0	0	1	0	0	0	1	0	3
**2.087**	0	0	0	1	0	0	0	0	0	0	0	0	0	1	0	0	0	0	0	1	0	0	0	1	1	3
**2.088**	0	1	0	1	0	0	0	0	0	0	0	0	0	0	0	0	0	0	0	1	0	0	0	1	0	3
**2.089**	0	0	1	0	0	0	0	0	0	0	0	1	0	0	0	1	0	0	0	0	0	0	0	1	0	3
**2.090**	0	0	0	0	0	0	1	0	0	0	1	0	0	0	0	0	0	0	0	0	0	0	0	1	0	3
**2.091**	0	0	1	0	0	0	0	0	1	0	0	0	0	0	0	0	0	0	0	0	0	0	1	1	1	3
**2.092**	0	0	1	0	0	0	0	0	0	0	0	0	0	1	0	1	0	0	0	0	0	0	0	1	0	3
**2.093**	0	0	0	0	0	0	0	0	1	1	0	1	0	0	0	1	0	0	0	0	0	0	1	1	1	2
**2.094**	0	0	0	0	0	0	1	0	1	0	0	0	0	0	0	0	0	0	0	0	0	1	1	1	1	2
**2.095**	0	0	0	0	1	0	0	0	0	0	0	1	0	1	0	0	0	0	0	1	0	0	0	1	1	2
**2.096**	0	0	0	0	1	0	0	0	1	0	0	1	0	0	0	0	0	0	0	1	0	0	0	1	0	2
**2.097**	0	0	0	0	1	0	1	0	0	0	0	1	0	1	0	0	0	0	0	0	0	0	0	1	1	2
**2.098**	0	0	0	1	0	0	0	0	1	0	0	0	0	0	0	0	0	0	0	0	0	0	1	1	1	2
**2.099**	0	0	0	1	0	0	0	0	1	0	0	1	0	0	0	0	0	0	0	0	0	0	0	1	0	2
**2.100**	0	0	0	1	0	0	1	0	0	0	0	0	1	0	0	1	0	0	0	0	0	0	0	1	0	2
**2.101**	0	0	1	0	0	0	1	0	1	0	0	1	0	0	0	0	0	0	0	0	0	0	1	1	1	2
**2.102**	0	1	0	1	1	0	1	0	1	0	0	1	0	0	0	0	0	0	0	0	0	0	0	1	0	2
**2.103**	0	1	1	1	1	0	1	0	1	0	0	1	0	0	0	1	0	0	0	1	0	0	0	1	1	2
**2.104**	0	0	0	0	0	0	1	0	1	0	0	0	0	0	0	1	0	0	0	1	0	0	0	1	1	2
**2.105**	0	0	0	0	0	0	1	0	1	0	0	0	1	0	0	0	0	0	0	1	0	0	0	1	1	2
**2.106**	0	0	0	0	1	0	1	0	1	0	0	0	0	0	0	1	0	0	0	1	0	0	0	1	1	2
**2.107**	0	0	0	1	0	0	1	0	1	0	0	0	0	0	0	1	0	0	0	1	0	0	1	1	1	2
**2.108**	0	0	1	1	0	0	0	0	1	0	0	0	0	0	0	0	0	0	0	1	0	1	0	1	1	2
**2.109**	0	0	1	0	0	0	0	0	0	1	0	1	0	0	0	0	0	0	0	0	0	0	1	1	1	2
**2.110**	0	0	1	0	0	0	0	0	1	0	0	0	0	1	0	0	0	0	0	0	0	1	0	1	1	2
**2.111**	0	1	0	1	1	0	0	0	0	1	0	1	1	0	0	0	0	0	0	1	0	1	1	1	0	2
**2.112**	0	1	0	1	0	0	0	0	0	1	0	1	1	1	0	0	0	0	0	1	0	1	0	1	0	2
**2.113**	0	0	0	0	0	0	0	0	0	0	0	1	0	0	0	1	0	0	0	0	0	1	0	1	1	2
**2.114**	0	0	0	0	0	0	0	0	1	0	0	1	0	1	0	1	0	0	0	1	0	1	0	1	1	2
**2.115**	0	0	0	0	0	0	1	0	1	0	0	0	0	0	0	0	0	0	0	1	0	0	1	1	1	2
**2.116**	0	1	0	0	0	0	0	0	0	1	0	1	1	1	0	0	0	0	0	1	0	0	1	1	0	2
**2.117**	0	1	1	0	0	0	0	0	0	1	0	1	1	1	0	0	0	0	0	1	0	0	0	1	1	2
**2.118**	0	1	1	0	0	0	0	0	1	1	0	1	1	0	0	0	0	0	0	1	0	0	0	1	0	2
**2.119**	0	1	1	1	1	0	0	0	0	0	0	1	1	0	0	0	0	0	0	1	0	0	1	1	1	2
**2.120**	0	0	1	0	0	0	0	0	1	0	0	1	0	0	0	0	0	0	0	0	0	0	1	1	0	2
**2.121**	0	0	1	1	0	0	0	0	0	0	0	0	1	1	0	0	0	0	0	0	0	0	0	1	0	2
**2.122**	0	1	0	0	1	0	0	0	0	0	0	0	0	1	0	0	0	0	0	1	0	0	0	1	1	2
**2.123**	0	1	0	1	0	0	0	0	0	0	0	0	1	0	0	0	0	0	0	0	0	1	0	1	0	2
**2.124**	0	1	1	0	1	0	0	0	0	0	0	1	0	0	0	0	0	0	0	1	0	0	1	1	1	2
**2.125**	0	1	1	0	1	0	0	0	0	0	0	1	0	1	0	0	0	0	0	1	0	0	0	1	1	2
**2.126**	0	0	1	0	0	0	1	0	0	1	0	1	1	1	0	0	0	0	0	1	0	0	1	1	1	2
**2.127**	0	0	1	0	1	0	0	0	1	0	0	0	0	0	0	0	0	0	0	1	0	0	0	1	1	2
**2.128**	0	1	0	1	0	0	0	0	0	1	0	1	1	0	0	0	0	0	0	0	0	0	0	1	0	2
**2.129**	0	1	0	0	1	0	0	0	0	0	0	0	0	0	0	0	0	0	0	1	0	0	1	1	0	2
**2.130**	0	0	0	0	0	0	0	0	0	0	0	0	1	1	0	0	0	0	0	0	0	0	1	1	0	2
**2.131**	0	0	0	0	0	0	1	0	1	0	0	1	0	0	0	1	0	0	0	1	0	0	0	1	1	2
**2.132**	0	0	1	0	0	0	1	0	0	1	0	1	0	1	0	1	0	0	0	1	0	1	0	1	0	2
**2.133**	0	1	0	1	0	0	0	0	0	1	0	1	0	1	0	0	0	0	0	1	0	0	1	1	1	2
**2.134**	0	0	0	0	0	0	1	0	1	1	0	0	0	0	0	0	0	0	0	1	0	0	1	1	0	2
**2.135**	0	1	0	0	0	0	0	0	1	0	1	1	0	0	0	0	0	0	0	0	0	0	1	1	0	2
**2.136**	0	1	0	0	1	0	0	0	1	1	1	1	0	0	0	0	0	0	0	0	0	0	1	1	1	2
**2.137**	0	1	0	1	0	0	0	0	0	1	0	1	0	0	0	0	0	0	0	1	0	0	1	1	0	2
**2.138**	0	0	0	0	0	0	0	0	1	1	0	0	0	1	0	0	0	0	0	1	0	0	0	1	0	2
**2.139**	0	0	0	0	0	0	0	0	1	1	0	1	0	1	0	0	0	0	0	1	0	0	1	1	1	2
**2.140**	0	0	0	0	0	0	1	0	1	0	0	0	0	1	0	0	0	0	0	1	0	1	1	1	0	2
**2.141**	0	0	1	0	0	0	0	0	0	1	0	1	0	1	0	0	0	0	0	0	0	1	1	1	1	2
**2.142**	0	1	0	1	1	0	0	0	1	1	0	1	1	0	0	0	0	0	0	0	0	1	0	1	1	2
**2.143**	0	0	1	0	0	0	0	0	0	1	1	1	0	1	0	0	0	0	0	0	0	0	0	1	0	2
**2.144**	0	0	1	0	0	0	1	0	0	1	1	0	0	0	0	0	0	0	0	0	0	0	0	1	1	2
**2.145**	0	0	1	1	0	0	0	0	0	1	0	0	0	0	0	0	0	0	0	0	0	0	0	1	0	2
**2.146**	0	0	0	0	0	0	0	0	1	0	0	0	0	0	0	1	0	0	0	0	0	0	1	1	0	2
**2.147**	0	0	0	0	0	0	0	0	1	0	0	1	0	0	0	1	0	0	0	1	0	0	0	1	0	2
**2.148**	0	0	0	0	0	0	1	0	1	0	0	0	0	0	0	0	0	0	0	0	0	1	0	1	0	2
**2.149**	0	0	0	0	0	0	1	0	1	0	0	1	0	0	0	1	0	0	0	0	0	0	0	1	0	2
**2.150**	0	0	0	0	1	0	0	0	0	0	0	1	1	1	0	0	0	0	0	0	0	0	0	1	1	2
**2.151**	0	0	0	0	1	0	0	0	0	1	0	0	0	0	0	0	0	0	0	0	0	0	0	1	0	2
**2.152**	0	0	0	0	1	0	0	0	1	0	0	1	0	0	0	0	0	0	0	0	0	1	1	1	0	2
**2.153**	0	0	0	0	1	0	0	0	1	0	0	1	0	0	0	0	0	0	0	1	0	1	1	1	1	2
**2.154**	0	0	0	0	1	0	0	0	1	0	0	1	1	0	0	0	0	0	0	1	0	0	0	1	1	2
**2.155**	0	0	0	0	1	0	0	0	1	1	0	1	0	1	0	0	0	0	0	1	0	1	0	1	0	2

	CD2	CD3	CD4	CD7	CD8	CD10	CD13	CD18	CD26	CD29	CD36	CD44	CD45RA	CD49f	CD54	CD56	CD57	CD58	CD62L	CD71	CD80	Cytokeratin	Histone_H1	HLA-DQ	HLA-DR	Freq. (Voxel)
**2.156**	0	0	0	0	1	0	1	0	1	0	0	1	0	0	0	0	0	0	0	0	0	0	1	1	1	2
**2.157**	0	0	0	0	1	0	1	0	1	0	0	1	0	1	0	0	0	0	0	1	0	1	0	1	1	2
**2.158**	0	0	0	1	0	0	0	0	1	0	0	1	1	1	0	0	0	0	0	0	0	1	0	1	0	2
**2.159**	0	0	0	1	0	0	1	0	0	0	0	1	0	0	0	0	0	0	0	1	0	0	0	1	0	2
**2.160**	0	0	0	1	0	0	1	0	0	0	0	1	0	0	0	1	0	0	0	0	0	0	0	1	1	2
**2.161**	0	0	0	1	0	0	1	0	0	0	0	1	0	1	0	0	0	0	0	1	0	0	0	1	1	2
**2.162**	0	0	0	1	0	0	1	0	1	0	0	1	0	0	0	0	0	0	0	0	0	0	1	1	0	2
**2.163**	0	0	0	1	0	0	1	0	1	1	0	0	1	0	0	0	0	0	0	1	0	0	0	1	1	2
**2.164**	0	0	0	1	0	0	1	0	1	1	0	1	1	0	0	0	0	0	0	1	0	0	0	1	1	2
**2.165**	0	0	0	1	1	0	0	0	0	0	0	1	0	0	0	0	0	0	0	0	0	1	1	1	1	2
**2.166**	0	0	0	1	1	0	0	0	0	0	0	1	0	0	0	0	0	0	0	1	0	1	0	1	0	2
**2.167**	0	0	0	1	1	0	0	0	1	0	0	1	0	0	0	0	0	0	0	1	0	0	0	1	0	2
**2.168**	0	0	0	1	1	0	0	0	1	0	0	1	1	1	0	0	0	0	0	1	0	0	0	1	1	2
**2.169**	0	0	0	1	1	0	1	0	0	0	0	1	0	0	0	0	0	0	0	0	0	0	0	1	0	2
**2.170**	0	0	0	1	1	0	1	0	1	1	0	1	1	0	0	0	0	0	0	1	0	0	0	1	1	2
**2.171**	0	0	1	0	0	0	1	0	1	0	0	0	0	0	0	0	0	0	0	0	0	1	0	1	1	2
**2.172**	0	0	1	0	1	0	0	0	0	0	0	1	0	0	0	0	0	0	0	0	0	1	0	1	1	2
**2.173**	0	0	1	0	1	0	0	0	1	1	0	1	1	0	0	0	0	0	0	1	0	0	0	1	1	2
**2.174**	0	0	1	1	0	0	0	0	0	0	0	1	0	0	0	0	0	0	0	1	0	1	0	1	1	2
**2.175**	0	0	1	1	0	0	0	0	1	0	0	0	0	1	0	0	0	0	0	0	0	1	0	1	0	2
**2.176**	0	0	1	1	0	0	0	0	1	0	0	1	0	0	0	0	0	0	0	0	0	1	0	1	0	2
**2.177**	0	0	1	1	0	0	0	0	1	0	0	1	1	0	0	0	0	0	0	0	0	0	0	1	0	2
**2.178**	0	1	0	0	0	0	1	0	0	0	0	1	0	0	0	0	0	0	0	1	0	1	0	1	1	2
**2.179**	0	1	0	0	1	0	0	0	0	1	0	1	0	0	0	0	0	0	0	1	0	1	0	1	0	2
**2.180**	0	1	0	0	1	0	0	0	1	1	0	1	0	0	0	0	0	0	0	0	0	1	0	1	0	2
**2.181**	0	1	0	0	1	0	0	0	1	1	0	1	0	1	0	0	0	0	0	0	0	0	0	1	0	2
**2.182**	0	1	0	0	1	0	1	0	1	0	0	1	0	1	0	0	0	0	0	0	0	1	0	1	1	2
**2.183**	0	1	0	0	1	0	1	0	1	0	0	1	1	1	0	0	0	0	0	1	0	1	0	1	1	2
**2.184**	0	1	0	1	1	0	0	0	0	0	0	1	0	1	0	0	0	0	0	1	0	0	0	1	1	2
**2.185**	0	1	0	1	1	0	0	0	1	0	0	1	0	0	0	0	0	0	0	0	0	1	0	1	0	2
**2.186**	0	1	0	1	1	0	0	0	1	0	0	1	0	0	0	0	0	0	0	0	0	1	1	1	0	2
**2.187**	0	1	0	1	1	0	0	0	1	0	0	1	0	0	0	0	0	0	0	1	0	1	1	1	0	2
**2.188**	0	1	0	1	1	0	0	0	1	0	0	1	1	0	0	0	0	0	0	1	0	0	1	1	1	2
**2.189**	0	1	0	1	1	0	0	0	1	1	0	1	0	1	0	0	0	0	0	0	0	0	0	1	0	2
**2.190**	0	1	0	1	1	0	1	0	1	1	0	1	0	0	0	0	0	0	0	0	0	0	1	1	1	2
**2.191**	0	1	0	1	1	0	1	0	1	1	0	1	1	0	0	0	0	0	0	1	0	0	0	1	0	2
**2.192**	0	1	1	0	0	0	0	0	1	1	0	1	0	0	0	0	0	0	0	0	0	1	1	1	1	2
**2.193**	0	1	1	0	1	0	1	0	1	0	0	1	1	0	0	0	0	0	0	1	0	1	0	1	1	2
**2.194**	0	1	1	0	1	0	1	0	1	1	0	1	0	0	0	0	0	0	0	1	0	1	0	1	1	2
**2.195**	0	1	1	1	0	0	1	0	1	1	0	1	1	0	0	0	0	0	0	1	0	1	0	1	1	2
**2.196**	0	0	0	0	0	0	0	0	0	0	1	1	0	1	0	0	0	0	0	1	0	0	0	1	0	2
**2.197**	0	0	0	0	0	0	0	0	1	0	0	1	0	0	0	0	0	0	0	1	0	1	1	1	1	2
**2.198**	0	0	0	0	0	0	0	0	1	1	0	1	0	0	0	0	0	0	0	1	0	0	1	1	1	2
**2.199**	0	0	0	0	0	0	1	0	1	0	0	0	0	0	0	1	0	0	0	0	0	0	1	1	1	2
**2.200**	0	0	0	0	0	0	1	0	1	0	0	1	0	0	0	1	0	0	0	0	0	0	1	1	1	2
**2.201**	0	0	0	0	1	0	0	0	0	0	0	1	0	1	0	0	0	0	0	0	0	0	0	1	0	2
**2.202**	0	0	0	0	1	0	0	0	0	0	0	1	1	0	0	0	0	0	0	1	0	1	1	1	1	2
**2.203**	0	0	0	0	1	0	0	0	1	1	0	1	1	1	0	0	0	0	0	0	0	0	0	1	0	2
**2.204**	0	0	0	0	1	0	1	0	1	1	0	1	0	0	0	0	0	0	0	0	0	1	0	1	1	2
**2.205**	0	0	0	1	0	0	0	0	1	0	0	1	0	1	0	0	0	0	0	1	0	0	0	1	0	2
**2.206**	0	0	0	1	0	0	0	0	1	1	0	1	1	0	0	0	0	0	0	1	0	1	0	1	0	2
**2.207**	0	0	0	1	0	0	1	0	0	0	0	0	1	0	0	0	0	0	0	1	0	0	0	1	0	2
**2.208**	0	0	0	1	0	0	1	0	1	0	0	1	0	0	0	1	0	0	0	0	0	0	0	1	1	2
**2.209**	0	0	0	1	0	0	1	0	1	1	0	1	1	0	0	0	0	0	0	1	0	0	0	1	0	2
**2.210**	0	0	0	1	1	0	0	0	0	0	0	1	1	0	0	0	0	0	0	1	0	1	0	1	1	2
**2.211**	0	0	0	1	1	0	0	0	0	1	0	1	1	0	0	0	0	0	0	1	0	0	0	1	1	2
**2.212**	0	1	0	0	0	0	0	0	1	1	0	1	0	0	0	0	0	0	0	0	0	1	1	1	1	2
**2.213**	0	1	0	0	0	0	0	0	1	1	0	1	1	0	0	0	0	0	0	0	0	1	1	1	1	2
**2.214**	0	1	0	0	1	0	0	0	0	1	0	1	1	1	0	0	0	0	0	1	0	0	1	1	0	2
**2.215**	0	1	0	0	1	0	1	0	0	1	0	1	1	1	0	0	0	0	0	1	0	0	1	1	0	2
**2.216**	0	1	0	1	0	0	1	0	0	0	0	1	0	0	0	0	0	0	0	0	0	0	1	1	0	2
**2.217**	0	1	0	1	1	0	0	0	1	0	0	1	1	1	0	0	0	0	0	0	0	0	0	1	1	2
**2.218**	0	1	0	1	1	0	0	0	1	0	0	1	1	1	0	0	0	0	0	1	0	1	0	1	1	2
**2.219**	0	1	0	1	1	0	0	0	1	1	0	1	1	1	0	0	0	0	0	1	0	1	0	1	0	2
**2.220**	0	1	0	1	1	0	0	0	1	1	0	1	1	1	0	0	0	0	0	1	0	1	0	1	1	2
**2.221**	0	1	0	1	1	0	1	0	0	0	0	1	0	0	0	0	0	0	0	0	0	0	1	1	0	2
**2.222**	0	1	0	1	1	0	1	0	1	1	0	1	1	1	0	0	0	0	0	1	0	0	1	1	0	2
**2.223**	0	1	1	0	0	0	0	0	0	0	0	1	0	0	0	0	0	0	0	1	0	1	0	1	1	2
**2.224**	0	1	1	0	0	0	0	0	1	0	0	0	0	0	0	0	0	0	0	1	0	1	1	1	1	2
**2.225**	0	1	1	0	1	0	0	0	1	0	0	1	1	1	0	0	0	0	0	1	0	0	0	1	1	2
**2.226**	0	1	1	0	1	0	0	0	1	0	0	1	1	1	0	0	0	0	0	1	0	1	0	1	1	2
**2.227**	0	1	1	1	1	0	0	0	1	1	0	1	0	1	0	0	0	0	0	1	0	1	0	1	1	2
**2.228**	0	1	1	1	1	0	0	0	1	1	0	1	1	1	0	0	0	0	0	0	0	0	0	1	0	2
**2.229**	0	0	0	0	0	0	0	0	0	0	0	0	0	1	0	0	0	0	0	1	0	1	1	1	0	2
**2.230**	0	0	0	0	0	0	0	0	0	1	1	1	0	1	0	0	0	0	0	1	0	0	0	1	0	2
**2.231**	0	0	0	0	0	0	0	0	1	1	0	1	0	0	0	0	0	0	0	1	0	1	1	1	0	2
**2.232**	0	0	0	0	0	0	1	0	1	1	0	1	0	0	0	0	0	0	0	0	0	1	0	1	0	2
**2.233**	0	0	0	0	1	0	0	0	0	0	0	1	0	1	0	0	0	0	0	1	0	1	0	1	1	2
**2.234**	0	0	0	0	1	0	0	0	1	0	0	0	0	0	0	0	0	0	0	0	0	0	1	1	1	2
**2.235**	0	0	0	0	1	0	0	0	1	0	0	1	0	1	0	0	0	0	0	0	0	1	0	1	1	2
**2.236**	0	0	0	1	0	0	0	0	1	1	0	1	0	0	0	0	0	0	0	0	0	0	0	1	0	2
**2.237**	0	0	0	1	0	0	1	0	0	1	0	1	1	0	0	0	0	0	0	0	0	0	0	1	1	2
**2.238**	0	0	0	1	0	0	1	0	0	1	0	1	1	0	0	0	0	0	0	1	0	0	0	1	1	2
**2.239**	0	0	0	1	1	0	0	0	0	1	0	1	0	0	0	0	0	0	0	0	0	1	0	1	0	2
**2.240**	0	0	0	1	1	0	0	0	0	1	0	1	0	0	0	0	0	0	0	0	0	1	0	1	1	2
**2.241**	0	0	1	0	0	0	0	0	1	0	0	1	1	0	0	0	0	0	0	1	0	1	0	1	0	2
**2.242**	0	0	1	0	1	0	0	0	0	0	0	1	0	0	0	0	0	0	0	0	0	0	1	1	1	2
**2.243**	0	0	1	1	0	0	0	0	0	0	0	1	0	1	0	0	0	0	0	0	0	0	0	1	1	2
**2.244**	0	0	1	1	0	0	1	0	0	1	0	0	0	0	0	0	0	0	0	1	0	0	0	1	1	2
**2.245**	0	0	1	1	0	0	1	0	1	1	0	1	1	0	0	1	0	0	0	1	0	0	0	1	1	2
**2.246**	0	1	0	0	0	0	0	0	0	0	0	1	1	0	0	0	0	0	0	1	0	0	0	1	1	2
**2.247**	0	1	0	0	0	0	0	0	0	0	0	1	1	0	0	0	0	0	0	1	0	0	1	1	0	2
**2.248**	0	1	0	0	0	0	0	0	0	0	0	1	1	0	0	0	0	0	0	1	0	1	1	1	1	2
**2.249**	0	1	0	0	0	0	1	0	0	1	0	1	0	0	0	0	0	0	0	0	0	0	0	1	0	2
**2.250**	0	1	0	0	1	0	0	0	0	1	0	0	0	0	0	0	0	0	0	1	0	1	1	1	1	2
**2.251**	0	1	0	0	1	0	0	0	1	0	0	0	0	0	0	0	0	0	0	1	0	0	0	1	1	2
**2.252**	0	1	0	0	1	0	1	0	0	0	0	0	0	0	0	0	0	0	0	0	0	0	1	1	1	2
**2.253**	0	1	0	0	1	0	1	0	0	0	0	1	0	0	0	0	0	0	0	0	0	0	1	1	1	2

	CD2	CD3	CD4	CD7	CD8	CD10	CD13	CD18	CD26	CD29	CD36	CD44	CD45RA	CD49f	CD54	CD56	CD57	CD58	CD62L	CD71	CD80	Cytokeratin	Histone_H1	HLA-DQ	HLA-DR	Freq. (Voxel)
**2.254**	0	1	0	0	1	0	1	0	0	1	0	1	0	0	0	0	0	0	0	0	0	0	1	1	1	2
**2.255**	0	1	0	1	0	0	0	0	0	0	0	1	1	1	0	0	0	0	0	0	0	0	0	1	0	2
**2.256**	0	1	0	1	0	0	0	0	1	0	0	1	0	0	0	0	0	0	0	1	0	0	1	1	1	2
**2.257**	0	1	0	1	1	0	0	0	0	0	0	1	1	1	0	0	0	0	0	1	0	1	0	1	0	2
**2.258**	0	1	0	1	1	0	0	0	1	1	0	1	1	0	0	0	0	0	0	1	0	1	0	1	0	2
**2.259**	0	1	0	1	1	0	1	0	0	0	0	1	1	0	0	0	0	0	0	1	0	1	0	1	1	2
**2.260**	0	1	0	1	1	0	1	0	0	1	0	1	0	0	0	0	0	0	0	0	0	0	1	1	0	2
**2.261**	0	1	0	1	1	0	1	0	1	1	0	1	0	0	0	0	0	0	0	1	0	1	0	1	1	2
**2.262**	0	1	1	0	0	0	0	0	0	0	0	0	0	0	0	0	0	0	0	1	0	0	0	1	1	2
**2.263**	0	1	1	0	0	0	0	0	1	0	0	1	0	0	0	0	0	0	0	0	0	1	1	1	1	2
**2.264**	0	1	1	0	0	0	1	0	1	1	0	0	0	0	0	0	0	0	0	1	0	0	0	1	1	2
**2.265**	0	1	1	0	1	0	0	0	1	0	0	1	1	0	0	0	0	0	0	1	0	0	1	1	1	2
**2.266**	0	1	1	0	1	0	0	0	1	1	0	1	1	0	0	0	0	0	0	1	0	0	1	1	0	2
**2.267**	0	1	1	0	1	0	1	0	0	1	0	1	1	0	0	0	0	0	0	1	0	1	0	1	1	2
**2.268**	0	0	0	0	0	0	0	0	0	1	0	0	1	1	0	0	0	0	0	0	0	0	1	1	0	2
**2.269**	0	0	0	0	0	0	1	0	0	0	0	0	0	0	0	1	0	0	0	0	0	0	1	1	0	2
**2.270**	0	0	0	0	1	0	1	0	1	1	0	1	0	1	0	0	0	0	0	1	0	0	0	1	1	2
**2.271**	0	0	0	1	0	0	1	0	0	0	0	0	0	0	0	0	0	0	0	0	0	0	0	1	1	2
**2.272**	0	0	0	1	1	0	1	0	1	1	0	1	0	1	0	0	0	0	0	1	0	0	0	1	0	2
**2.273**	0	0	1	0	0	0	0	0	0	0	0	1	0	0	0	1	0	0	0	0	0	0	1	1	1	2
**2.274**	0	0	1	0	0	0	0	0	0	1	0	0	1	0	0	0	0	0	0	1	0	0	0	1	1	2
**2.275**	0	0	1	0	0	0	1	0	0	1	0	0	0	0	0	0	0	0	0	1	0	0	1	1	0	2
**2.276**	0	0	1	1	0	0	0	0	0	0	0	0	0	0	0	0	0	0	0	1	0	0	0	1	1	2
**2.277**	0	0	1	1	1	0	1	0	1	1	0	1	0	1	0	0	0	0	0	1	0	0	0	1	1	2
**2.278**	0	1	0	0	0	0	0	0	1	1	0	0	0	0	0	0	0	0	0	1	0	0	0	1	1	2
**2.279**	0	1	0	0	0	0	0	0	1	1	0	1	1	0	0	0	0	0	0	0	0	0	0	1	0	2
**2.280**	0	1	0	0	0	0	1	0	1	0	0	1	0	1	0	0	0	0	0	0	0	1	0	1	1	2
**2.281**	0	1	0	0	1	0	0	0	0	0	0	0	0	0	0	0	0	0	0	1	0	1	1	1	1	2
**2.282**	0	1	0	0	1	0	0	0	0	0	0	1	1	1	0	0	0	0	0	1	0	0	0	1	1	2
**2.283**	0	1	0	0	1	0	0	0	0	1	1	1	1	0	0	0	0	0	0	0	0	0	0	1	0	2
**2.284**	0	1	0	1	0	0	0	0	0	0	0	1	0	0	0	0	0	0	0	1	0	0	1	1	0	2
**2.285**	0	1	0	1	0	0	0	0	0	0	0	1	1	0	0	0	0	0	0	1	0	1	1	1	1	2
**2.286**	0	1	0	1	0	0	0	0	0	0	0	1	1	1	0	0	0	0	0	0	0	0	1	1	0	2
**2.287**	0	1	0	1	0	0	1	0	1	0	0	1	0	0	0	1	0	0	0	1	0	0	1	1	1	2
**2.288**	0	1	0	1	0	0	1	0	1	1	0	1	0	1	0	0	0	0	0	1	0	1	0	1	1	2
**2.289**	0	1	0	1	1	0	0	0	0	0	0	1	1	1	0	0	0	0	0	1	0	0	1	1	0	2
**2.290**	0	1	0	1	1	0	0	0	0	1	0	1	0	0	0	0	0	0	0	0	0	1	0	1	1	2
**2.291**	0	1	0	1	1	0	0	0	1	0	0	1	0	1	0	0	0	0	0	0	0	1	0	1	1	2
**2.292**	0	1	0	1	1	0	0	0	1	0	0	1	1	1	0	0	0	0	0	1	0	0	0	1	0	2
**2.293**	0	1	1	0	0	0	0	0	0	0	0	1	0	0	0	0	0	0	0	0	0	0	1	1	1	2
**2.294**	0	1	1	0	0	0	0	0	1	0	0	0	0	0	0	0	0	0	0	0	0	0	0	1	1	2
**2.295**	0	0	0	0	0	0	0	0	0	0	0	0	1	1	0	1	0	0	0	0	0	1	0	1	0	2
**2.296**	0	0	0	0	0	0	0	0	0	0	1	1	0	1	0	0	0	0	0	0	0	1	0	1	0	2
**2.297**	0	0	0	0	0	0	0	0	0	1	0	1	1	1	0	0	0	0	0	1	0	0	0	1	0	2
**2.298**	0	0	0	0	0	0	1	0	0	0	0	0	1	1	0	1	0	0	0	0	0	0	0	1	0	2
**2.299**	0	0	0	0	0	0	1	0	0	1	0	0	1	1	0	1	0	0	0	0	0	0	0	1	0	2
**2.300**	0	0	0	0	0	0	1	0	1	0	0	0	0	1	0	0	0	0	0	1	0	0	1	1	1	2
**2.301**	0	0	0	0	1	0	0	0	1	0	0	1	0	0	0	1	0	0	0	1	0	0	0	1	1	2
**2.302**	0	0	0	1	0	0	0	0	0	0	0	0	0	0	0	0	0	0	0	0	0	1	1	1	1	2
**2.303**	0	0	0	1	0	0	0	0	0	0	0	0	1	1	0	0	0	0	0	0	0	0	0	1	0	2
**2.304**	0	0	0	1	0	0	1	0	0	1	0	1	1	1	0	0	0	0	0	0	0	0	0	1	1	2
**2.305**	0	0	1	0	0	0	1	0	0	0	0	0	0	0	0	0	0	0	0	0	0	0	1	1	1	2
**2.306**	0	0	1	0	0	0	1	0	0	0	0	0	0	0	0	0	0	0	0	0	0	1	0	1	0	2
**2.307**	0	0	1	0	0	0	1	0	0	0	0	0	1	1	0	1	0	0	0	0	0	0	0	1	0	2
**2.308**	0	0	1	0	0	0	1	0	0	0	0	0	1	1	0	1	0	0	0	1	0	1	0	1	0	2
**2.309**	0	0	1	1	0	0	0	0	0	0	0	0	0	1	0	0	0	0	0	1	0	0	0	1	1	2
**2.310**	0	0	1	1	1	0	1	0	0	1	0	1	1	1	0	1	0	0	0	1	0	0	0	1	1	2
**2.311**	0	1	0	0	0	0	0	0	0	1	0	0	0	0	0	0	0	0	0	1	0	0	0	1	1	2
**2.312**	0	1	0	0	1	0	0	0	0	0	0	0	0	0	0	0	0	0	0	1	0	0	0	1	0	2
**2.313**	0	1	0	1	0	0	0	0	0	0	0	0	0	0	0	0	0	0	0	1	0	0	0	1	1	2
**2.314**	0	1	0	1	0	0	0	0	0	0	0	1	1	0	0	0	0	0	0	1	0	1	0	1	0	2
**2.315**	0	1	0	1	0	0	0	0	0	1	0	1	1	0	0	0	0	0	0	1	0	1	0	1	0	2
**2.316**	0	1	0	1	0	0	1	0	0	0	0	0	0	0	0	0	0	0	0	0	0	1	1	1	1	2
**2.317**	0	0	0	0	0	0	0	0	0	0	0	1	1	0	0	0	0	0	0	1	0	0	0	1	0	2
**2.318**	0	0	0	0	0	0	0	0	0	1	0	0	0	0	0	1	0	0	0	0	0	1	0	1	0	2
**2.319**	0	0	0	0	0	0	0	0	1	0	1	1	0	1	0	0	0	0	0	1	0	1	0	1	1	2
**2.320**	0	0	0	0	0	0	1	0	0	1	0	0	0	0	0	0	0	0	0	1	0	0	1	1	1	2
**2.321**	0	0	0	0	0	0	1	0	1	0	0	1	0	0	0	0	0	0	0	1	0	0	1	1	1	2
**2.322**	0	0	0	1	1	0	1	0	0	1	0	1	0	1	0	0	0	0	0	0	0	1	0	1	1	2
**2.323**	0	0	1	0	0	0	0	0	1	1	0	1	0	1	0	0	0	0	0	1	0	0	0	1	0	2
**2.324**	0	0	1	0	0	0	1	0	0	0	0	1	0	0	0	0	0	0	0	1	0	0	0	1	0	2
**2.325**	0	0	1	0	1	0	0	0	0	0	0	0	1	0	0	1	0	0	0	1	0	1	0	1	0	2
**2.326**	0	1	0	0	1	0	0	0	0	1	0	0	0	0	0	0	0	0	0	1	0	0	0	1	1	2
**2.327**	0	1	0	0	1	0	1	0	0	1	0	1	0	0	0	0	0	0	0	1	0	0	0	1	1	2
**2.328**	0	1	0	0	1	0	1	0	1	1	0	1	0	1	0	0	0	0	0	1	0	1	0	1	0	2
**2.329**	0	1	0	1	0	0	0	0	0	0	0	1	0	0	0	0	0	0	0	1	0	1	1	1	0	2
**2.330**	0	1	0	1	0	0	0	0	0	0	0	1	1	0	0	0	0	0	0	0	0	0	0	1	1	2
**2.331**	0	1	0	1	0	0	0	0	0	0	0	1	1	1	0	0	0	0	0	0	0	1	0	1	0	2
**2.332**	0	1	0	1	0	0	0	0	1	0	0	1	0	0	0	0	0	0	0	0	0	0	1	1	1	2
**2.333**	0	1	1	0	1	0	0	0	0	1	0	0	0	0	0	0	0	0	0	1	0	0	0	1	1	2
**2.334**	0	1	1	0	1	0	0	0	1	0	0	1	0	0	0	0	0	0	0	1	0	0	0	1	0	2
**2.335**	0	0	0	0	0	0	0	0	1	1	0	1	0	1	0	0	0	0	0	1	0	1	1	1	1	2
**2.336**	0	0	1	0	0	0	0	0	0	1	0	0	0	0	0	1	0	0	0	0	0	0	0	1	1	2
**2.337**	0	0	1	0	0	0	1	0	0	0	0	1	0	1	0	1	0	0	0	1	0	0	0	1	1	2
**2.338**	0	0	1	0	0	0	1	0	0	0	0	1	1	1	0	0	0	0	0	1	0	0	0	1	1	2
**2.339**	0	0	1	0	0	0	1	0	0	1	0	0	0	1	0	0	0	0	0	0	0	1	1	1	0	2
**2.340**	0	0	1	0	0	0	1	0	0	1	0	1	0	1	0	1	0	0	0	0	0	0	1	1	1	2
**2.341**	0	0	1	0	1	0	0	0	0	0	0	0	1	0	0	0	0	0	0	0	0	0	0	1	0	2
**2.342**	0	0	1	0	1	0	0	0	0	1	0	1	1	0	0	0	0	0	0	0	0	0	0	1	0	2
**2.343**	0	0	1	0	1	0	1	0	1	0	0	0	0	0	0	0	0	0	0	0	0	0	0	1	1	2
**2.344**	0	0	1	1	0	0	0	0	0	0	0	0	1	1	0	0	0	0	0	0	0	1	0	1	0	2
**2.345**	0	0	1	1	0	0	0	0	0	0	0	0	1	1	0	1	0	0	0	0	0	1	0	1	0	2
**2.346**	0	0	1	1	0	0	0	0	0	0	0	1	1	1	0	0	0	0	0	1	0	1	0	1	1	2
**2.347**	0	1	0	0	0	0	0	0	0	0	0	0	0	1	0	0	0	0	0	1	0	1	0	1	0	2
**2.348**	0	1	0	0	0	0	0	0	0	0	0	0	0	1	0	0	0	0	0	1	0	1	1	1	0	2
**2.349**	0	1	0	0	0	0	0	0	1	0	0	1	0	0	0	1	0	0	0	0	0	0	0	1	1	2
**2.350**	0	1	0	0	0	0	0	0	1	1	0	1	0	1	0	0	0	0	0	0	0	0	0	1	0	2
**2.351**	0	1	0	0	0	0	0	0	1	1	0	1	1	1	0	0	0	0	0	0	0	0	0	1	0	2

	CD2	CD3	CD4	CD7	CD8	CD10	CD13	CD18	CD26	CD29	CD36	CD44	CD45RA	CD49f	CD54	CD56	CD57	CD58	CD62L	CD71	CD80	Cytokeratin	Histone_H1	HLA-DQ	HLA-DR	Freq. (Voxel)
**2.352**	0	1	0	0	1	0	1	0	0	1	0	1	0	1	0	0	0	0	0	1	0	1	1	1	1	2
**2.353**	0	1	0	1	0	0	0	0	0	0	0	0	0	1	0	0	0	0	0	1	0	0	0	1	1	2
**2.354**	0	1	0	1	0	0	0	0	0	0	0	1	1	0	0	0	0	0	0	0	0	1	0	1	0	2
**2.355**	0	1	0	1	0	0	0	0	0	1	0	1	1	0	0	0	0	0	0	1	0	0	0	1	0	2
**2.356**	0	1	0	1	0	0	0	0	1	1	0	1	1	1	0	0	0	0	0	1	0	0	0	1	0	2
**2.357**	0	1	0	1	1	0	0	0	0	1	0	1	0	1	0	0	0	0	0	1	0	0	0	1	0	2
**2.358**	0	1	1	0	0	0	0	0	0	1	0	1	0	1	0	0	0	0	0	0	0	0	0	1	1	2
**2.359**	0	1	1	1	0	0	0	0	1	0	0	1	0	0	0	0	0	0	0	1	0	0	0	1	0	2
**2.360**	0	1	1	1	1	0	0	0	0	0	0	1	1	0	0	0	0	0	0	0	0	0	1	1	1	2
**2.361**	0	0	0	0	0	0	0	0	0	1	0	0	0	1	0	0	0	0	0	1	0	1	0	1	0	2
**2.362**	0	0	0	0	0	0	1	0	0	1	0	1	0	1	0	1	0	0	0	0	0	0	1	1	0	2
**2.363**	0	0	0	0	0	0	1	0	0	1	0	1	1	1	0	0	0	0	0	1	0	1	1	1	0	2
**2.364**	0	0	0	0	0	0	1	0	1	0	0	0	1	0	0	0	0	0	0	0	0	0	1	1	0	2
**2.365**	0	0	1	0	0	0	0	0	0	1	0	0	1	0	0	1	0	0	0	0	0	1	0	1	0	2
**2.366**	0	0	1	0	0	0	0	0	0	1	1	0	0	1	0	0	0	0	0	0	0	1	0	1	0	2
**2.367**	0	0	1	0	0	0	0	0	1	0	0	0	1	0	0	1	0	0	0	0	0	0	0	1	0	2
**2.368**	0	0	1	0	0	0	1	0	0	0	0	0	1	1	0	0	0	0	0	0	0	0	0	1	1	2
**2.369**	0	0	1	0	0	0	1	0	0	0	1	1	0	1	0	0	0	0	0	0	0	1	0	1	1	2
**2.370**	0	0	1	0	1	0	0	0	0	0	0	0	0	0	0	0	0	0	0	0	0	0	0	1	0	2
**2.371**	0	1	0	0	0	0	0	0	0	0	0	1	0	1	0	0	0	0	0	1	0	0	1	1	1	2
**2.372**	0	1	0	0	0	0	0	0	0	0	1	0	0	1	0	0	0	0	0	1	0	0	0	1	0	2
**2.373**	0	1	0	0	0	0	0	0	0	0	1	1	0	1	0	0	0	0	0	1	0	0	0	1	0	2
**2.374**	0	1	0	0	0	0	0	0	0	1	0	0	0	0	0	0	0	0	0	0	0	0	1	1	0	2
**2.375**	0	1	0	0	0	0	0	0	0	1	0	1	1	1	0	0	0	0	0	1	0	0	0	1	0	2
**2.376**	0	1	0	0	1	0	0	0	1	0	0	0	1	0	0	0	0	0	0	0	0	0	1	1	1	2
**2.377**	0	1	0	1	0	0	0	0	0	0	0	1	0	1	0	0	0	0	0	0	0	0	0	1	0	2
**2.378**	0	1	0	1	1	0	0	0	0	1	0	1	0	1	0	0	0	0	0	1	0	0	1	1	1	2
**2.379**	0	1	1	0	1	0	0	0	0	0	0	1	0	0	0	0	0	0	0	0	0	0	1	1	1	2
**2.380**	0	1	1	0	1	0	0	0	0	1	0	1	0	0	0	0	0	0	0	0	0	0	1	1	1	2
**2.381**	0	1	1	0	1	0	0	0	0	1	0	1	1	0	0	0	0	0	0	0	0	0	0	1	0	2
**2.382**	0	1	1	0	1	0	0	0	0	1	0	1	1	0	0	0	0	0	0	1	0	0	0	1	0	2
**2.383**	0	1	1	0	1	0	1	0	0	1	0	1	0	1	0	0	0	0	0	0	0	1	0	1	1	2
**2.384**	0	0	0	0	0	0	0	0	0	0	1	0	0	1	0	0	0	0	0	1	0	1	0	1	0	2
**2.385**	0	0	0	0	0	0	0	0	1	1	1	1	0	1	0	0	0	0	0	1	0	0	0	1	1	2
**2.386**	0	0	0	0	0	0	1	0	0	1	1	1	1	1	0	0	0	0	0	1	0	0	0	1	1	2
**2.387**	0	0	0	0	0	0	1	0	1	1	0	1	1	1	0	0	0	0	0	1	0	0	0	1	1	2
**2.388**	0	0	0	0	0	0	1	0	1	1	1	1	0	0	0	0	0	0	0	1	0	0	0	1	1	2
**2.389**	0	0	0	0	1	0	0	0	0	1	0	0	0	0	0	0	0	0	0	1	0	0	1	1	1	2
**2.390**	0	0	0	0	1	0	0	0	1	0	0	1	0	0	0	0	0	0	0	0	0	1	1	1	1	2
**2.391**	0	0	0	0	1	0	0	0	1	0	0	1	0	0	0	1	0	0	0	0	0	0	0	1	1	2
**2.392**	0	0	0	0	1	0	0	0	1	0	0	1	1	0	0	0	0	0	0	0	0	0	0	1	0	2
**2.393**	0	0	0	0	1	0	1	0	0	0	0	0	1	0	0	0	0	0	0	0	0	0	0	1	0	2
**2.394**	0	0	1	0	0	0	0	0	0	0	1	0	0	1	0	0	0	0	0	0	0	1	0	1	0	2
**2.395**	0	0	1	0	0	0	0	0	0	0	1	1	0	1	0	0	0	0	0	0	0	1	0	1	0	2
**2.396**	0	0	1	1	0	0	1	0	1	1	1	1	0	1	0	0	0	0	0	0	0	0	0	1	1	2
**2.397**	0	1	0	0	0	0	0	0	0	0	0	1	0	1	0	0	0	0	0	0	0	0	1	1	0	2
**2.398**	0	1	0	0	0	0	0	0	0	0	0	1	1	1	0	0	0	0	0	0	0	0	0	1	0	2
**2.399**	0	1	0	0	1	0	0	0	0	1	1	1	0	0	0	0	0	0	0	0	0	0	0	1	1	2
**2.400**	0	1	0	0	1	0	0	0	1	1	0	0	1	0	0	0	0	0	0	0	0	0	1	1	0	2
**2.401**	0	1	0	1	1	0	0	0	0	1	1	1	1	1	0	0	0	0	0	0	0	0	1	1	1	2
**2.402**	0	1	1	1	1	0	0	0	0	0	0	0	1	0	0	0	0	0	0	0	0	0	0	1	0	2
**2.403**	0	0	0	0	0	0	0	0	0	1	1	1	0	1	0	1	0	0	0	0	0	0	0	1	1	2
**2.404**	0	0	0	0	0	0	0	0	1	0	0	0	1	0	0	0	0	0	0	0	0	0	1	1	0	2
**2.405**	0	0	0	0	0	0	0	0	1	0	0	0	1	1	0	0	0	0	0	0	0	0	0	1	0	2
**2.406**	0	0	0	0	0	0	0	0	1	1	0	0	0	1	0	0	0	0	0	0	0	0	0	1	0	2
**2.407**	0	0	0	0	0	0	1	0	0	0	0	1	0	1	0	0	0	0	0	1	0	1	1	1	1	2
**2.408**	0	0	0	0	0	0	1	0	0	0	0	1	0	1	0	1	0	0	0	0	0	0	0	1	0	2
**2.409**	0	0	1	0	0	0	1	0	1	1	0	1	0	1	0	1	0	0	0	0	0	0	1	1	1	2
**2.410**	0	1	0	0	0	0	0	0	0	0	0	0	0	1	0	0	0	0	0	0	0	1	1	1	0	2
**2.411**	0	1	0	0	1	0	0	0	0	1	0	1	0	1	0	0	0	0	0	0	0	0	0	1	0	2
**2.412**	0	1	0	0	1	0	0	0	1	1	1	1	0	0	0	0	0	0	0	0	0	0	0	1	0	2
**2.413**	0	0	0	0	0	0	0	0	0	1	1	1	0	0	0	0	0	0	0	0	0	0	1	1	1	2
**2.414**	0	0	0	0	0	0	1	0	0	0	0	0	1	0	0	0	0	0	0	0	0	0	0	1	1	2
**2.415**	0	0	1	0	0	0	0	0	0	1	0	1	0	1	0	1	0	0	0	0	0	1	0	1	1	2
**2.416**	0	0	1	0	0	0	0	0	1	1	0	0	0	1	0	0	0	0	0	0	0	0	0	1	1	2
**2.417**	0	0	1	0	0	0	1	0	1	0	0	0	1	0	0	0	0	0	0	1	0	0	0	1	0	2
**2.418**	0	1	0	0	0	0	0	0	1	1	1	1	0	0	0	0	0	0	0	0	0	0	0	1	0	2
**2.419**	0	1	0	1	0	0	0	0	1	1	0	1	1	0	0	0	0	0	0	0	0	0	0	1	0	2
**2.420**	0	1	0	1	1	0	0	0	0	0	0	0	0	0	0	0	0	0	0	0	0	0	0	1	0	2
**2.421**	0	1	1	0	1	0	0	0	1	1	0	0	0	0	0	0	0	0	0	1	0	0	0	1	0	2
**2.422**	0	1	1	1	1	0	0	0	0	0	0	0	0	0	0	0	0	0	0	0	0	0	0	1	0	2
**2.423**	0	0	0	0	0	0	0	0	0	0	1	1	0	0	0	0	0	0	0	1	0	0	0	1	0	2
**2.424**	0	0	0	0	0	0	0	0	0	1	1	1	0	0	0	0	0	0	0	0	0	0	1	1	0	2
**2.425**	0	0	0	0	0	0	0	0	1	0	1	1	0	1	0	0	0	0	0	0	0	0	0	1	1	2
**2.426**	0	0	0	0	0	0	1	0	0	0	0	1	0	1	0	0	0	0	0	1	0	1	0	1	0	2
**2.427**	0	0	0	0	0	0	1	0	1	0	0	0	0	1	0	0	0	0	0	0	0	1	1	1	0	2
**2.428**	0	0	0	0	0	0	1	0	1	1	1	0	0	1	0	0	0	0	0	0	0	0	0	1	1	2
**2.429**	0	0	0	0	0	0	1	0	1	1	1	1	0	1	0	1	0	0	0	1	0	0	0	1	1	2
**2.430**	0	0	1	0	0	0	0	0	1	1	0	0	0	1	0	0	0	0	0	1	0	0	0	1	0	2
**2.431**	0	0	1	0	0	0	1	0	0	0	0	1	0	1	0	0	0	0	0	1	0	1	1	1	0	2
**2.432**	0	0	1	0	0	0	1	0	0	0	1	0	0	0	0	0	0	0	0	0	0	0	0	1	0	2
**2.433**	0	0	1	0	0	0	1	0	1	1	1	1	1	1	0	1	0	0	0	0	0	0	0	1	1	2
**2.434**	0	1	0	1	0	0	0	0	1	0	0	0	1	0	0	0	0	0	0	0	0	0	0	1	0	2
**2.435**	0	1	0	1	0	0	0	0	1	0	0	1	1	0	0	0	0	0	0	0	0	0	0	1	0	2
**2.436**	0	1	0	1	0	0	0	0	1	0	0	1	1	0	0	0	0	0	0	1	0	0	0	1	0	2
**2.437**	0	0	0	0	0	0	0	0	0	1	0	1	1	1	0	0	0	0	0	0	0	1	0	1	1	2
**2.438**	0	0	0	0	0	0	0	0	0	1	1	1	0	1	0	0	0	0	0	0	0	0	1	1	1	2
**2.439**	0	0	0	0	0	0	0	0	1	0	0	1	0	1	0	0	0	0	0	1	0	0	1	1	1	2
**2.440**	0	0	0	0	0	0	0	0	1	0	1	0	0	1	0	0	0	0	0	0	0	0	0	1	1	2
**2.441**	0	0	0	0	0	0	0	0	1	1	0	0	0	1	0	0	0	0	0	0	0	0	1	1	1	2
**2.442**	0	0	0	0	0	0	0	0	1	1	0	1	0	1	0	1	0	0	0	0	0	0	1	1	1	2
**2.443**	0	0	0	0	0	0	1	0	0	0	0	0	0	1	0	0	0	0	0	1	0	1	1	1	0	2
**2.444**	0	0	0	1	0	0	0	0	0	1	0	0	0	1	0	0	0	0	0	0	0	0	1	1	1	2
**2.445**	0	0	0	1	0	0	1	0	0	1	0	0	0	1	0	0	0	0	0	1	0	0	0	1	1	2
**2.446**	0	0	1	0	0	0	0	0	0	1	1	1	0	1	0	1	0	0	0	0	0	0	0	1	1	2
**2.447**	0	0	1	0	0	0	0	0	1	1	0	1	0	1	0	1	0	0	0	0	0	0	0	1	1	2
**2.448**	0	0	1	0	0	0	1	0	0	1	0	1	0	1	0	0	0	0	0	1	0	0	1	1	0	2
**2.449**	0	1	0	0	1	0	0	0	0	0	1	1	0	0	0	0	0	0	0	0	0	0	1	1	0	2

	CD2	CD3	CD4	CD7	CD8	CD10	CD13	CD18	CD26	CD29	CD36	CD44	CD45RA	CD49f	CD54	CD56	CD57	CD58	CD62L	CD71	CD80	Cytokeratin	Histone_H1	HLA-DQ	HLA-DR	Freq. (Voxel)
**2.450**	0	0	0	0	0	0	0	0	0	0	0	1	0	1	0	0	0	0	0	1	0	0	1	1	1	2
**2.451**	0	0	0	0	0	0	0	0	0	1	0	0	1	0	0	1	0	0	0	1	0	0	0	1	0	2
**2.452**	0	0	0	0	0	0	0	0	0	1	0	1	0	1	0	1	0	0	0	0	0	1	1	1	0	2
**2.453**	0	0	0	0	0	0	0	0	1	0	0	0	0	1	0	0	0	0	0	0	0	0	1	1	1	2
**2.454**	0	0	0	0	0	0	0	0	1	0	0	0	0	1	0	1	0	0	0	0	0	0	0	1	1	2
**2.455**	0	0	0	0	0	0	0	0	1	0	1	0	0	0	0	0	0	0	0	0	0	0	0	1	1	2
**2.456**	0	0	0	0	0	0	1	0	0	1	1	0	0	1	0	0	0	0	0	1	0	0	0	1	1	2
**2.457**	0	0	1	0	0	0	0	0	0	1	0	1	0	1	0	1	0	0	0	0	0	0	0	1	1	2
**2.458**	0	0	0	0	0	0	0	0	1	1	0	1	0	1	0	0	0	0	0	0	0	0	1	1	0	2
**2.459**	0	0	0	0	1	0	0	0	0	0	0	0	1	0	0	1	0	0	0	0	0	0	0	1	0	2
**2.460**	0	0	0	1	0	0	1	0	0	1	0	0	1	1	0	0	0	0	0	1	0	0	0	1	1	2
**2.461**	0	0	1	0	0	0	0	0	0	0	1	0	0	0	0	0	0	0	0	1	0	0	0	1	1	2
**2.462**	0	0	1	0	0	0	0	0	0	1	1	0	0	0	0	0	0	0	0	1	0	0	0	1	1	2
**2.463**	0	1	0	0	0	0	0	0	0	0	0	0	1	0	0	0	0	0	0	0	0	0	1	1	1	2
**2.464**	0	0	0	0	0	0	0	0	1	0	0	0	0	0	0	1	0	0	0	1	0	0	0	1	0	2
**2.465**	0	0	0	1	0	0	0	0	0	1	0	0	0	1	0	0	0	0	0	1	0	0	0	1	1	2
**2.466**	0	0	1	1	0	0	0	0	0	1	0	0	0	0	0	0	0	0	0	0	0	0	0	1	1	2
**2.467**	0	0	0	0	0	0	0	0	0	0	0	0	1	1	0	1	0	0	0	1	0	1	0	1	0	2
**2.468**	0	0	0	0	0	0	1	0	1	0	0	0	1	0	0	0	0	0	0	1	0	1	0	1	0	2
**2.469**	0	1	0	0	1	0	0	0	1	0	0	0	0	0	0	0	0	0	0	1	0	0	0	1	0	2
**2.470**	0	0	0	0	0	0	0	0	1	0	0	1	1	0	0	0	0	0	0	0	0	0	0	1	1	1
**2.471**	0	0	0	0	0	0	0	0	1	1	0	1	0	0	0	0	0	0	0	1	0	1	1	1	1	1
**2.472**	0	0	0	0	0	0	0	0	1	1	0	1	0	0	0	1	0	0	0	0	0	0	0	1	1	1
**2.473**	0	0	0	0	0	0	0	0	1	1	0	1	0	0	0	1	0	0	0	0	0	1	0	1	1	1
**2.474**	0	0	0	0	0	0	0	0	1	1	0	1	0	0	0	1	0	0	0	1	0	0	1	1	1	1
**2.475**	0	0	0	0	0	0	0	0	1	1	0	1	0	0	0	1	0	0	0	1	0	1	0	1	1	1
**2.476**	0	0	0	0	0	0	0	0	1	1	0	1	1	0	0	0	0	0	0	1	0	0	0	1	1	1
**2.477**	0	0	0	0	0	0	1	0	0	0	0	0	1	0	0	1	0	0	0	1	0	0	0	1	1	1
**2.478**	0	0	0	0	0	0	1	0	0	0	0	1	1	0	0	0	0	0	0	0	0	1	0	1	0	1
**2.479**	0	0	0	0	0	0	1	0	0	1	0	1	1	0	0	0	0	0	0	0	0	0	0	1	0	1
**2.480**	0	0	0	0	0	0	1	0	1	0	0	1	0	0	0	0	0	0	0	1	0	1	0	1	0	1
**2.481**	0	0	0	0	0	0	1	0	1	1	0	1	1	0	0	0	0	0	0	0	0	1	0	1	0	1
**2.482**	0	0	0	0	0	0	1	0	1	1	0	1	1	0	0	0	0	0	0	1	0	0	0	1	0	1
**2.483**	0	0	0	0	1	0	0	0	0	0	0	0	1	0	0	0	0	0	0	0	0	0	0	1	1	1
**2.484**	0	0	0	0	1	0	0	0	0	0	0	0	1	0	0	0	0	0	0	0	0	1	0	1	0	1
**2.485**	0	0	0	0	1	0	0	0	0	0	0	1	0	0	0	0	0	0	0	0	0	1	1	1	0	1
**2.486**	0	0	0	0	1	0	0	0	0	0	0	1	1	0	0	0	0	0	0	0	0	1	1	1	0	1
**2.487**	0	0	0	0	1	0	0	0	0	0	0	1	1	0	0	0	0	0	0	1	0	0	1	1	1	1
**2.488**	0	0	0	0	1	0	0	0	0	1	0	0	0	0	0	1	0	0	0	0	0	0	0	1	0	1
**2.489**	0	0	0	0	1	0	0	0	1	0	0	1	0	1	0	0	0	0	0	1	0	0	0	1	1	1
**2.490**	0	0	0	0	1	0	0	0	1	0	0	1	1	0	0	0	0	0	0	0	0	0	1	1	0	1
**2.491**	0	0	0	0	1	0	0	0	1	0	0	1	1	0	0	0	0	0	0	0	0	1	1	1	1	1
**2.492**	0	0	0	0	1	0	0	0	1	0	0	1	1	1	0	0	0	0	0	0	0	0	1	1	0	1
**2.493**	0	0	0	0	1	0	0	0	1	0	0	1	1	1	0	0	0	0	0	1	0	1	0	1	1	1
**2.494**	0	0	0	0	1	0	0	0	1	1	0	1	0	0	0	0	0	0	0	0	0	0	1	1	1	1
**2.495**	0	0	0	0	1	0	0	0	1	1	0	1	1	0	0	0	0	0	0	0	0	1	1	1	1	1
**2.496**	0	0	0	0	1	0	0	0	1	1	0	1	1	1	0	0	0	0	0	0	0	0	0	1	1	1
**2.497**	0	0	0	0	1	0	1	0	0	0	0	0	0	0	0	0	0	0	0	0	0	0	0	1	0	1
**2.498**	0	0	0	0	1	0	1	0	0	0	0	1	0	0	0	1	0	0	0	0	0	0	0	1	1	1
**2.499**	0	0	0	0	1	0	1	0	1	0	0	0	0	0	0	0	0	0	0	0	0	0	1	1	1	1
**2.500**	0	0	0	1	0	0	0	0	0	0	0	0	0	0	0	1	0	0	0	1	0	0	0	1	0	1
**2.501**	0	0	0	1	0	0	0	0	0	0	0	0	0	1	0	0	0	0	0	0	0	1	0	1	0	1
**2.502**	0	0	0	1	0	0	0	0	0	0	0	0	1	0	0	0	0	0	0	1	0	1	0	1	1	1
**2.503**	0	0	0	1	0	0	0	0	0	0	0	1	0	0	0	0	0	0	0	0	0	1	1	1	0	1
**2.504**	0	0	0	1	0	0	0	0	0	0	0	1	1	0	0	0	0	0	0	0	0	1	0	1	1	1
**2.505**	0	0	0	1	0	0	0	0	0	1	0	0	1	0	0	0	0	0	0	0	0	0	0	1	0	1
**2.506**	0	0	0	1	0	0	0	0	1	0	0	0	0	1	0	0	0	0	0	0	0	0	0	1	1	1
**2.507**	0	0	0	1	0	0	0	0	1	0	0	0	1	0	0	0	0	0	0	0	0	0	0	1	1	1
**2.508**	0	0	0	1	0	0	0	0	1	0	0	1	0	1	0	0	0	0	0	1	0	0	0	1	1	1
**2.509**	0	0	0	1	0	0	0	0	1	0	0	1	1	0	0	0	0	0	0	0	0	0	0	1	0	1
**2.510**	0	0	0	1	0	0	0	0	1	1	0	0	1	1	0	0	0	0	0	1	0	0	0	1	1	1
**2.511**	0	0	0	1	0	0	1	0	0	0	0	0	1	0	0	1	0	0	0	1	0	1	0	1	0	1
**2.512**	0	0	0	1	0	0	1	0	0	0	0	1	0	1	0	0	0	0	0	0	0	0	0	1	0	1
**2.513**	0	0	0	1	0	0	1	0	1	0	0	0	0	0	0	0	0	0	0	0	0	0	1	1	0	1
**2.514**	0	0	0	1	0	0	1	0	1	0	0	0	0	0	0	1	0	0	0	0	0	0	0	1	1	1
**2.515**	0	0	0	1	0	0	1	0	1	1	0	1	1	0	0	0	0	0	0	1	0	1	0	1	0	1
**2.516**	0	0	0	1	1	0	0	0	0	0	0	1	0	0	0	0	0	0	0	0	0	1	0	1	1	1
**2.517**	0	0	0	1	1	0	0	0	0	0	0	1	0	0	0	0	0	0	0	1	0	1	1	1	1	1
**2.518**	0	0	0	1	1	0	0	0	0	0	0	1	1	0	0	0	0	0	0	1	0	0	1	1	1	1
**2.519**	0	0	0	1	1	0	0	0	0	0	0	1	1	0	0	0	0	0	0	1	0	1	0	1	0	1
**2.520**	0	0	0	1	1	0	0	0	0	1	0	0	0	0	0	1	0	0	0	0	0	0	0	1	0	1
**2.521**	0	0	0	1	1	0	0	0	1	0	0	0	0	0	0	0	0	0	0	0	0	0	0	1	0	1
**2.522**	0	0	0	1	1	0	0	0	1	0	0	0	1	0	0	0	0	0	0	1	0	0	1	1	1	1
**2.523**	0	0	0	1	1	0	0	0	1	0	0	1	0	0	0	0	0	0	0	0	0	1	0	1	1	1
**2.524**	0	0	0	1	1	0	0	0	1	0	0	1	0	0	0	0	0	0	0	0	0	1	1	1	1	1
**2.525**	0	0	0	1	1	0	0	0	1	0	0	1	1	0	0	0	0	0	0	1	0	1	1	1	1	1
**2.526**	0	0	0	1	1	0	0	0	1	1	0	1	1	0	0	0	0	0	0	0	0	0	0	1	0	1
**2.527**	0	0	0	1	1	0	1	0	0	0	0	1	0	0	0	1	0	0	0	1	0	0	0	1	1	1
**2.528**	0	0	0	1	1	0	1	0	0	1	0	1	0	0	0	0	0	0	0	0	0	1	0	1	1	1
**2.529**	0	0	0	1	1	0	1	0	0	1	0	1	0	0	0	0	0	0	0	1	0	0	0	1	1	1
**2.530**	0	0	0	1	1	0	1	0	0	1	0	1	0	0	0	0	0	0	0	1	0	1	0	1	1	1
**2.531**	0	0	0	1	1	0	1	0	1	0	0	1	0	0	0	0	0	0	0	1	0	0	0	1	1	1
**2.532**	0	0	1	0	0	0	0	0	0	0	0	0	0	0	0	0	0	0	0	1	0	1	1	1	0	1
**2.533**	0	0	1	0	0	0	0	0	0	0	0	0	0	0	0	0	0	0	0	1	0	1	1	1	1	1
**2.534**	0	0	1	0	0	0	0	0	0	0	0	1	1	1	0	0	0	0	0	0	0	0	0	1	1	1
**2.535**	0	0	1	0	0	0	0	0	0	1	0	1	0	0	0	1	0	0	0	1	0	1	0	1	0	1
**2.536**	0	0	1	0	0	0	0	0	1	0	0	0	0	0	0	0	0	0	0	1	0	1	1	1	1	1
**2.537**	0	0	1	0	0	0	0	0	1	0	0	0	0	1	0	0	0	0	0	0	0	1	0	1	0	1
**2.538**	0	0	1	0	0	0	1	0	0	0	0	0	0	0	0	0	0	0	0	0	0	1	0	1	1	1
**2.539**	0	0	1	0	0	0	1	0	0	0	0	1	0	0	0	0	0	0	0	0	0	1	0	1	0	1
**2.540**	0	0	1	0	0	0	1	0	0	1	0	0	0	0	0	0	0	0	0	1	0	0	0	1	0	1
**2.541**	0	0	1	0	0	0	1	0	1	0	0	0	0	0	0	0	0	0	0	0	0	0	1	1	1	1
**2.542**	0	0	1	0	0	0	1	0	1	1	0	0	0	0	0	0	0	0	0	0	0	0	0	1	1	1
**2.543**	0	0	1	0	0	0	1	0	1	1	0	1	0	0	0	0	0	0	0	1	0	1	0	1	1	1
**2.544**	0	0	1	0	1	0	0	0	0	1	0	1	1	0	0	0	0	0	0	1	0	0	0	1	1	1
**2.545**	0	0	1	0	1	0	1	0	1	0	0	1	0	0	0	0	0	0	0	1	0	1	0	1	1	1
**2.546**	0	0	1	1	0	0	0	0	0	1	0	0	0	0	0	0	0	0	0	1	0	0	0	1	0	1
**2.547**	0	0	1	1	0	0	0	0	1	0	0	0	1	0	0	0	0	0	0	0	0	0	0	1	0	1

	CD2	CD3	CD4	CD7	CD8	CD10	CD13	CD18	CD26	CD29	CD36	CD44	CD45RA	CD49f	CD54	CD56	CD57	CD58	CD62L	CD71	CD80	Cytokeratin	Histone_H1	HLA-DQ	HLA-DR	Freq. (Voxel)
**2.548**	0	0	1	1	0	0	0	0	1	0	0	1	0	0	0	0	0	0	0	0	0	0	0	1	0	1
**2.549**	0	0	1	1	0	0	0	0	1	0	0	1	0	0	0	0	0	0	0	1	0	0	0	1	0	1
**2.550**	0	0	1	1	0	0	0	0	1	0	0	1	0	1	0	0	0	0	0	0	0	1	0	1	0	1
**2.551**	0	0	1	1	0	0	0	0	1	0	0	1	1	0	0	0	0	0	0	1	0	0	0	1	0	1
**2.552**	0	0	1	1	0	0	1	0	0	0	0	1	0	0	0	1	0	0	0	0	0	0	0	1	1	1
**2.553**	0	0	1	1	0	0	1	0	0	1	0	1	0	0	0	0	0	0	0	0	0	0	0	1	0	1
**2.554**	0	0	1	1	0	0	1	0	0	1	0	1	0	0	0	0	0	0	0	1	0	0	0	1	0	1
**2.555**	0	0	1	1	1	0	0	0	0	1	0	1	0	0	0	0	0	0	0	0	0	0	0	1	1	1
**2.556**	0	0	1	1	1	0	0	0	0	1	0	1	0	0	0	0	0	0	0	1	0	0	0	1	1	1
**2.557**	0	0	1	1	1	0	0	0	1	1	0	1	0	0	0	0	0	0	0	1	0	0	0	1	1	1
**2.558**	0	0	1	1	1	0	1	0	1	0	0	1	0	0	0	0	0	0	0	0	0	0	0	1	1	1
**2.559**	0	0	1	1	1	0	1	0	1	0	0	1	0	0	0	0	0	0	0	1	0	0	0	1	1	1
**2.560**	0	0	1	1	1	0	1	0	1	0	0	1	0	0	0	1	0	0	0	1	0	0	0	1	1	1
**2.561**	0	1	0	0	0	0	0	0	0	1	0	1	0	0	0	0	0	0	0	1	0	1	0	1	0	1
**2.562**	0	1	0	0	0	0	0	0	1	0	0	0	1	0	0	0	0	0	0	0	0	0	1	1	0	1
**2.563**	0	1	0	0	0	0	0	0	1	1	0	1	0	0	0	0	0	0	0	1	0	0	1	1	0	1
**2.564**	0	1	0	0	0	0	0	0	1	1	0	1	0	0	0	0	0	0	0	1	0	1	1	1	0	1
**2.565**	0	1	0	0	0	0	1	0	1	0	0	1	0	0	0	0	0	0	0	0	0	1	1	1	1	1
**2.566**	0	1	0	0	0	0	1	0	1	0	0	1	0	0	0	0	0	0	0	1	0	1	1	1	1	1
**2.567**	0	1	0	0	1	0	0	0	1	1	0	1	0	0	0	0	0	0	0	1	0	1	1	1	1	1
**2.568**	0	1	0	0	1	0	0	0	1	1	0	1	1	1	0	0	0	0	0	1	0	1	0	1	1	1
**2.569**	0	1	0	0	1	0	1	0	0	0	0	1	0	0	0	0	0	0	0	1	0	0	0	1	0	1
**2.570**	0	1	0	1	0	0	0	0	0	1	0	1	0	0	0	0	0	0	0	0	0	1	1	1	0	1
**2.571**	0	1	0	1	0	0	0	0	1	0	0	0	0	0	0	0	0	0	0	0	0	0	1	1	0	1
**2.572**	0	1	0	1	0	0	0	0	1	0	0	1	0	0	0	0	0	0	0	0	0	1	1	1	0	1
**2.573**	0	1	0	1	0	0	0	0	1	0	0	1	1	0	0	0	0	0	0	0	0	0	1	1	1	1
**2.574**	0	1	0	1	0	0	0	0	1	1	0	1	0	0	0	0	0	0	0	1	0	1	0	1	1	1
**2.575**	0	1	0	1	1	0	0	0	0	0	0	0	1	0	0	0	0	0	0	1	0	0	0	1	0	1
**2.576**	0	1	0	1	1	0	0	0	0	0	0	1	1	1	0	0	0	0	0	0	0	0	0	1	1	1
**2.577**	0	1	0	1	1	0	0	0	1	0	0	0	0	0	0	0	0	0	0	1	0	1	1	1	0	1
**2.578**	0	1	0	1	1	0	0	0	1	0	0	1	0	0	0	0	0	0	0	0	0	1	1	1	1	1
**2.579**	0	1	0	1	1	0	0	0	1	1	0	1	1	0	0	0	0	0	0	0	0	1	0	1	0	1
**2.580**	0	1	0	1	1	0	1	0	1	0	0	1	1	0	0	0	0	0	0	0	0	0	0	1	1	1
**2.581**	0	1	0	1	1	0	1	0	1	0	0	1	1	1	0	0	0	0	0	1	0	1	0	1	1	1
**2.582**	0	1	1	0	0	0	0	0	1	1	0	1	0	0	0	0	0	0	0	0	0	1	0	1	0	1
**2.583**	0	1	1	0	0	0	1	0	1	0	0	1	1	0	0	0	0	0	0	1	0	1	0	1	1	1
**2.584**	0	1	1	0	0	0	1	0	1	1	0	1	0	0	0	0	0	0	0	1	0	1	0	1	1	1
**2.585**	0	1	1	0	1	0	0	0	0	0	0	1	0	0	0	0	0	0	0	0	0	1	0	1	1	1
**2.586**	0	1	1	0	1	0	0	0	1	0	0	0	0	0	0	0	0	0	0	1	0	1	1	1	1	1
**2.587**	0	1	1	0	1	0	0	0	1	0	0	1	1	0	0	0	0	0	0	1	0	0	1	1	0	1
**2.588**	0	1	1	0	1	0	0	0	1	1	0	1	0	0	0	0	0	0	0	1	0	1	1	1	1	1
**2.589**	0	1	1	0	1	0	1	0	1	0	0	1	0	1	0	0	0	0	0	0	0	1	0	1	1	1
**2.590**	0	1	1	0	1	0	1	0	1	0	0	1	0	1	0	0	0	0	0	1	0	1	0	1	1	1
**2.591**	0	1	1	0	1	0	1	0	1	1	0	1	0	0	0	0	0	0	0	1	0	0	1	1	0	1
**2.592**	0	1	1	0	1	0	1	0	1	1	0	1	1	1	0	0	0	0	0	1	0	0	0	1	0	1
**2.593**	0	1	1	1	1	0	0	0	1	0	0	1	0	1	0	0	0	0	0	1	0	1	0	1	1	1
**2.594**	0	1	1	1	1	0	0	0	1	0	0	1	1	0	0	0	0	0	0	0	0	0	0	1	1	1
**2.595**	0	1	1	1	1	0	0	0	1	0	0	1	1	0	0	0	0	0	0	1	0	0	1	1	0	1
**2.596**	0	1	1	1	1	0	1	0	1	0	0	1	0	0	0	0	0	0	0	1	0	0	0	1	1	1
**2.597**	0	1	1	1	1	0	1	0	1	0	0	1	1	1	0	0	0	0	0	1	0	1	0	1	1	1
**2.598**	0	1	1	1	1	0	1	0	1	1	0	1	0	0	0	0	0	0	0	1	0	1	1	1	1	1
**2.599**	0	1	1	1	1	0	1	0	1	1	0	1	1	1	0	0	0	0	0	1	0	0	0	1	0	1
**2.600**	0	0	0	0	0	0	0	0	0	1	0	1	1	0	0	0	0	0	0	0	0	1	0	1	1	1
**2.601**	0	0	0	0	0	0	0	0	1	0	0	1	0	0	0	1	0	0	0	0	0	0	1	1	0	1
**2.602**	0	0	0	0	0	0	0	0	1	0	0	1	1	0	0	0	0	0	0	1	0	0	0	1	0	1
**2.603**	0	0	0	0	0	0	0	0	1	1	0	1	0	0	0	0	0	0	0	0	0	1	1	1	0	1
**2.604**	0	0	0	0	0	0	1	0	0	0	0	0	1	0	0	1	0	0	0	0	0	0	0	1	1	1
**2.605**	0	0	0	0	0	0	1	0	0	0	0	1	0	0	0	0	0	0	0	1	0	0	1	1	0	1
**2.606**	0	0	0	0	0	0	1	0	1	0	0	1	0	0	0	1	0	0	0	0	0	0	1	1	0	1
**2.607**	0	0	0	0	0	0	1	0	1	1	0	0	0	0	0	0	0	0	0	1	0	0	0	1	1	1
**2.608**	0	0	0	0	0	0	1	0	1	1	0	1	1	0	0	0	0	0	0	0	0	0	0	1	0	1
**2.609**	0	0	0	0	1	0	0	0	0	1	0	0	0	0	0	0	0	0	0	0	0	1	0	1	1	1
**2.610**	0	0	0	0	1	0	0	0	0	1	0	1	0	1	0	0	0	0	0	0	0	1	0	1	0	1
**2.611**	0	0	0	0	1	0	0	0	1	0	0	1	0	1	0	0	0	0	0	1	0	1	0	1	1	1
**2.612**	0	0	0	0	1	0	0	0	1	1	0	0	0	0	0	0	0	0	0	0	0	1	0	1	1	1
**2.613**	0	0	0	0	1	0	0	0	1	1	0	1	0	1	0	0	0	0	0	0	0	1	0	1	0	1
**2.614**	0	0	0	0	1	0	1	0	0	0	0	0	0	0	0	0	0	0	0	1	0	0	0	1	1	1
**2.615**	0	0	0	0	1	0	1	0	0	1	0	0	0	0	0	0	0	0	0	1	0	0	0	1	1	1
**2.616**	0	0	0	0	1	0	1	0	0	1	0	1	0	0	0	0	0	0	0	0	0	1	0	1	0	1
**2.617**	0	0	0	1	0	0	0	0	1	0	0	0	0	0	0	0	0	0	0	0	0	0	1	1	0	1
**2.618**	0	0	0	1	0	0	0	0	1	0	0	1	0	0	0	0	0	0	0	1	0	0	0	1	0	1
**2.619**	0	0	0	1	0	0	0	0	1	0	0	1	0	0	0	0	0	0	0	1	0	1	1	1	0	1
**2.620**	0	0	0	1	0	0	0	0	1	0	0	1	1	1	0	0	0	0	0	1	0	0	0	1	1	1
**2.621**	0	0	0	1	0	0	0	0	1	1	0	1	0	0	0	0	0	0	0	1	0	1	0	1	0	1
**2.622**	0	0	0	1	0	0	0	0	1	1	0	1	0	1	0	0	0	0	0	1	0	1	0	1	1	1
**2.623**	0	0	0	1	0	0	1	0	0	0	0	0	0	0	0	1	0	0	0	1	0	0	0	1	1	1
**2.624**	0	0	0	1	1	0	0	0	0	0	0	0	0	0	0	0	0	0	0	1	0	0	1	1	1	1
**2.625**	0	0	0	1	1	0	0	0	0	0	0	1	0	0	0	0	0	0	0	0	0	0	1	1	0	1
**2.626**	0	0	0	1	1	0	0	0	0	1	0	0	0	0	0	0	0	0	0	0	0	0	0	1	0	1
**2.627**	0	0	0	1	1	0	0	0	0	1	0	1	0	1	0	0	0	0	0	1	0	1	0	1	0	1
**2.628**	0	0	0	1	1	0	0	0	1	0	0	0	0	0	0	0	0	0	0	0	0	0	0	1	1	1
**2.629**	0	0	0	1	1	0	0	0	1	0	0	1	0	1	0	0	0	0	0	0	0	0	0	1	1	1
**2.630**	0	0	0	1	1	0	0	0	1	0	0	1	1	0	0	0	0	0	0	0	0	1	0	1	1	1
**2.631**	0	0	0	1	1	0	0	0	1	1	0	1	0	0	0	0	0	0	0	0	0	1	0	1	1	1
**2.632**	0	0	1	0	0	0	0	0	0	0	0	1	1	1	0	0	0	0	0	1	0	0	0	1	1	1
**2.633**	0	0	1	0	0	0	0	0	1	0	0	0	0	1	0	0	0	0	0	1	0	1	0	1	0	1
**2.634**	0	0	1	0	1	0	0	0	0	0	0	1	1	0	0	0	0	0	0	1	0	0	0	1	1	1
**2.635**	0	0	1	0	1	0	0	0	0	1	0	1	0	0	0	0	0	0	0	1	0	0	1	1	0	1
**2.636**	0	0	1	0	1	0	1	0	0	1	0	0	0	0	0	0	0	0	0	1	0	0	0	1	1	1
**2.637**	0	0	1	0	1	0	1	0	1	0	0	1	0	0	0	0	0	0	0	0	0	0	1	1	1	1
**2.638**	0	0	1	1	0	0	0	0	0	0	0	0	1	0	0	0	0	0	0	1	0	0	0	1	1	1
**2.639**	0	0	1	1	0	0	1	0	0	1	0	1	1	0	0	0	0	0	0	1	0	0	0	1	1	1
**2.640**	0	1	0	0	0	0	0	0	0	0	0	0	1	0	0	0	0	0	0	1	0	0	1	1	1	1
**2.641**	0	1	0	0	0	0	0	0	0	0	0	1	0	1	0	0	0	0	0	1	0	1	1	1	1	1
**2.642**	0	1	0	0	0	0	0	0	1	0	0	1	0	1	0	0	0	0	0	1	0	1	1	1	1	1
**2.643**	0	1	0	0	0	0	0	0	1	0	0	1	1	0	0	0	0	0	0	0	0	1	1	1	1	1
**2.644**	0	1	0	0	0	0	0	0	1	0	0	1	1	0	0	0	0	0	0	1	0	1	0	1	0	1
**2.645**	0	1	0	0	0	0	0	0	1	0	0	1	1	1	0	0	0	0	0	0	0	1	1	1	1	1

	CD2	CD3	CD4	CD7	CD8	CD10	CD13	CD18	CD26	CD29	CD36	CD44	CD45RA	CD49f	CD54	CD56	CD57	CD58	CD62L	CD71	CD80	Cytokeratin	Histone_H1	HLA-DQ	HLA-DR	Freq. (Voxel)
**2.646**	0	1	0	0	0	0	1	0	0	0	0	1	1	0	0	0	0	0	0	1	0	1	0	1	1	1
**2.647**	0	1	0	0	0	0	1	0	1	0	0	1	0	0	0	0	0	0	0	0	0	0	1	1	1	1
**2.648**	0	1	0	0	0	0	1	0	1	0	0	1	0	0	0	0	0	0	0	1	0	1	0	1	1	1
**2.649**	0	1	0	0	0	0	1	0	1	1	0	1	1	0	0	0	0	0	0	1	0	1	0	1	1	1
**2.650**	0	1	0	0	1	0	0	0	0	0	0	0	0	0	0	0	0	0	0	0	0	0	1	1	0	1
**2.651**	0	1	0	0	1	0	0	0	0	0	0	1	0	1	0	0	0	0	0	1	0	1	1	1	1	1
**2.652**	0	1	0	0	1	0	0	0	0	0	0	1	1	1	0	0	0	0	0	1	0	1	1	1	1	1
**2.653**	0	1	0	0	1	0	0	0	0	1	0	0	0	0	0	0	0	0	0	1	0	1	0	1	1	1
**2.654**	0	1	0	0	1	0	1	0	0	0	0	0	0	0	0	0	0	0	0	1	0	0	1	1	1	1
**2.655**	0	1	0	0	1	0	1	0	1	1	0	1	1	1	0	0	0	0	0	1	0	0	1	1	0	1
**2.656**	0	1	0	1	0	0	0	0	0	0	0	0	1	0	0	0	0	0	0	0	0	0	0	1	1	1
**2.657**	0	1	0	1	0	0	0	0	1	0	0	1	1	0	0	0	0	0	0	0	0	1	0	1	0	1
**2.658**	0	1	0	1	0	0	0	0	1	1	0	1	0	0	0	0	0	0	0	0	0	0	1	1	0	1
**2.659**	0	1	0	1	1	0	0	0	0	0	0	1	1	1	0	0	0	0	0	1	0	1	1	1	1	1
**2.660**	0	1	0	1	1	0	0	0	0	1	0	0	0	0	0	0	0	0	0	1	0	0	0	1	1	1
**2.661**	0	1	0	1	1	0	0	0	0	1	0	1	1	0	0	0	0	0	0	0	0	1	0	1	1	1
**2.662**	0	1	0	1	1	0	0	0	1	0	0	0	1	0	0	0	0	0	0	1	0	0	1	1	1	1
**2.663**	0	1	0	1	1	0	0	0	1	0	0	1	1	0	0	0	0	0	0	1	0	0	1	1	0	1
**2.664**	0	1	0	1	1	0	0	0	1	1	0	1	1	0	0	0	0	0	0	1	0	1	1	1	1	1
**2.665**	0	1	0	1	1	0	1	0	0	1	0	1	1	0	0	0	0	0	0	1	0	1	0	1	1	1
**2.666**	0	1	1	0	0	0	0	0	1	0	0	1	0	0	0	0	0	0	0	0	0	1	0	1	1	1
**2.667**	0	1	1	0	0	0	1	0	1	0	0	0	0	0	0	0	0	0	0	1	0	0	0	1	1	1
**2.668**	0	1	1	0	1	0	0	0	0	0	0	1	1	0	0	0	0	0	0	1	0	1	0	1	1	1
**2.669**	0	1	1	0	1	0	0	0	0	1	0	1	0	0	0	0	0	0	0	1	0	0	1	1	0	1
**2.670**	0	1	1	0	1	0	0	0	1	0	0	1	0	0	0	0	0	0	0	1	0	1	1	1	1	1
**2.671**	0	1	1	1	1	0	0	0	1	0	0	1	1	1	0	0	0	0	0	1	0	1	0	1	1	1
**2.672**	0	1	1	1	1	0	0	0	1	1	0	1	0	0	0	0	0	0	0	0	0	0	0	1	1	1
**2.673**	0	1	1	1	1	0	0	0	1	1	0	1	1	0	0	0	0	0	0	1	0	1	1	1	1	1
**2.674**	0	1	1	1	1	0	0	0	1	1	0	1	1	1	0	0	0	0	0	1	0	1	0	1	1	1
**2.675**	0	1	1	1	1	0	1	0	1	1	0	1	1	1	0	0	0	0	0	1	0	0	1	1	0	1
**2.676**	0	0	0	0	0	0	0	0	0	0	0	0	1	1	0	0	0	0	0	1	0	1	0	1	0	1
**2.677**	0	0	0	0	0	0	0	0	0	0	0	1	1	0	0	1	0	0	0	0	0	1	0	1	1	1
**2.678**	0	0	0	0	0	0	0	0	0	1	0	0	1	0	0	1	0	0	0	0	0	0	0	1	1	1
**2.679**	0	0	0	0	0	0	0	0	0	1	0	1	1	0	0	0	0	0	0	1	0	1	0	1	0	1
**2.680**	0	0	0	0	0	0	0	0	0	1	0	1	1	0	0	1	0	0	0	0	0	0	0	1	0	1
**2.681**	0	0	0	0	0	0	0	0	0	1	0	1	1	1	0	0	0	0	0	1	0	1	0	1	0	1
**2.682**	0	0	0	0	0	0	0	0	1	0	0	0	0	0	0	1	0	0	0	0	0	1	0	1	1	1
**2.683**	0	0	0	0	0	0	0	0	1	0	0	0	1	1	0	0	0	0	0	0	0	1	0	1	0	1
**2.684**	0	0	0	0	0	0	0	0	1	0	0	0	1	1	0	0	0	0	0	0	0	1	0	1	1	1
**2.685**	0	0	0	0	0	0	0	0	1	0	0	1	0	0	0	0	0	0	0	1	0	1	1	1	0	1
**2.686**	0	0	0	0	0	0	0	0	1	1	0	1	0	0	0	0	0	0	0	1	0	0	1	1	0	1
**2.687**	0	0	0	0	0	0	1	0	0	0	0	0	0	0	0	1	0	0	0	1	0	0	1	1	1	1
**2.688**	0	0	0	0	0	0	1	0	0	0	0	1	0	0	0	1	0	0	0	1	0	0	1	1	1	1
**2.689**	0	0	0	0	0	0	1	0	0	1	0	0	0	0	0	0	0	0	0	0	0	1	0	1	0	1
**2.690**	0	0	0	0	0	0	1	0	0	1	0	0	0	0	0	0	0	0	0	1	0	0	1	1	0	1
**2.691**	0	0	0	0	0	0	1	0	0	1	0	0	1	1	0	0	0	0	0	1	0	0	0	1	0	1
**2.692**	0	0	0	0	1	0	0	0	0	1	0	0	0	0	0	0	0	0	0	1	0	0	0	1	1	1
**2.693**	0	0	0	0	1	0	0	0	0	1	0	1	1	0	0	0	0	0	0	0	0	1	0	1	0	1
**2.694**	0	0	0	1	0	0	0	0	0	1	0	0	1	1	0	0	0	0	0	1	0	0	0	1	0	1
**2.695**	0	0	0	1	0	0	0	0	0	1	0	1	0	0	0	0	0	0	0	1	0	1	1	1	0	1
**2.696**	0	0	0	1	0	0	0	0	0	1	0	1	1	0	0	0	0	0	0	0	0	0	0	1	1	1
**2.697**	0	0	0	1	0	0	0	0	0	1	0	1	1	0	0	0	0	0	0	1	0	0	0	1	1	1
**2.698**	0	0	0	1	0	0	0	0	0	1	0	1	1	1	0	0	0	0	0	0	0	1	0	1	0	1
**2.699**	0	0	0	1	0	0	0	0	1	1	0	1	1	1	0	0	0	0	0	1	0	1	1	1	0	1
**2.700**	0	0	0	1	0	0	1	0	0	0	0	0	0	0	0	1	0	0	0	0	0	0	0	1	1	1
**2.701**	0	0	0	1	1	0	1	0	0	0	0	1	0	0	0	0	0	0	0	0	0	0	1	1	0	1
**2.702**	0	0	1	0	0	0	0	0	0	0	0	0	1	0	0	0	0	0	0	1	0	0	1	1	0	1
**2.703**	0	0	1	0	0	0	0	0	0	1	0	0	1	0	0	0	0	0	0	1	0	0	0	1	0	1
**2.704**	0	0	1	0	0	0	0	0	0	1	0	0	1	1	0	0	0	0	0	0	0	0	1	1	0	1
**2.705**	0	0	1	0	0	0	0	0	1	1	0	1	1	0	0	0	0	0	0	1	0	0	1	1	0	1
**2.706**	0	0	1	0	0	0	1	0	0	0	0	0	1	0	0	0	0	0	0	0	0	1	0	1	0	1
**2.707**	0	0	1	0	1	0	0	0	0	0	0	1	0	1	0	0	0	0	0	1	0	1	0	1	1	1
**2.708**	0	0	1	1	0	0	0	0	1	0	0	1	1	0	0	0	0	0	0	0	0	0	0	1	1	1
**2.709**	0	0	1	1	0	0	1	0	0	1	0	1	1	0	0	0	0	0	0	0	0	0	0	1	1	1
**2.710**	0	0	1	1	0	0	1	0	0	1	0	1	1	0	0	1	0	0	0	1	0	0	0	1	1	1
**2.711**	0	0	1	1	0	0	1	0	1	1	0	1	0	0	0	1	0	0	0	1	0	0	0	1	1	1
**2.712**	0	0	1	1	0	0	1	0	1	1	0	1	1	0	0	0	0	0	0	1	0	0	0	1	1	1
**2.713**	0	1	0	0	0	0	0	0	0	0	0	1	1	1	0	0	0	0	0	1	0	1	1	1	1	1
**2.714**	0	1	0	0	0	0	0	0	1	0	0	0	0	1	0	0	0	0	0	0	0	1	1	1	1	1
**2.715**	0	1	0	0	0	0	0	0	1	0	0	1	0	1	0	0	0	0	0	1	0	0	0	1	1	1
**2.716**	0	1	0	0	0	0	0	0	1	0	0	1	1	0	0	0	0	0	0	1	0	0	1	1	0	1
**2.717**	0	1	0	0	0	0	0	0	1	1	0	1	1	1	0	0	0	0	0	1	0	1	0	1	0	1
**2.718**	0	1	0	0	0	0	0	0	1	1	0	1	1	1	0	0	0	0	0	1	0	1	0	1	1	1
**2.719**	0	1	0	0	0	0	1	0	1	1	0	0	0	0	0	0	0	0	0	1	0	0	0	1	1	1
**2.720**	0	1	0	0	1	0	0	0	1	0	0	1	0	1	0	0	0	0	0	1	0	0	0	1	1	1
**2.721**	0	1	0	0	1	0	0	0	1	1	0	0	0	1	0	0	0	0	0	1	0	1	0	1	1	1
**2.722**	0	1	0	0	1	0	0	0	1	1	0	1	0	1	0	0	0	0	0	0	0	1	1	1	1	1
**2.723**	0	1	0	0	1	0	0	0	1	1	0	1	1	0	0	0	0	0	0	1	0	1	0	1	0	1
**2.724**	0	1	0	0	1	0	1	0	1	1	0	1	0	0	0	0	0	0	0	1	0	1	0	1	1	1
**2.725**	0	1	0	0	1	0	1	0	1	1	0	1	1	1	0	0	0	0	0	1	0	1	1	1	0	1
**2.726**	0	1	0	1	0	0	0	0	0	0	0	1	1	1	0	0	0	0	0	0	0	1	0	1	1	1
**2.727**	0	1	0	1	0	0	0	0	0	0	0	1	1	1	0	0	0	0	0	1	0	0	0	1	0	1
**2.728**	0	1	0	1	0	0	0	0	0	0	0	1	1	1	0	0	0	0	0	1	0	1	1	1	1	1
**2.729**	0	1	0	1	0	0	0	0	1	1	0	1	1	0	0	0	0	0	0	1	0	1	0	1	1	1
**2.730**	0	1	0	1	0	0	1	0	0	0	0	1	1	1	0	0	0	0	0	0	0	1	0	1	1	1
**2.731**	0	1	0	1	1	0	0	0	0	0	0	1	1	1	0	0	0	0	0	0	0	1	0	1	1	1
**2.732**	0	1	0	1	1	0	0	0	0	0	0	1	1	1	0	0	0	0	0	1	0	0	0	1	0	1
**2.733**	0	1	0	1	1	0	0	0	1	0	0	0	0	0	0	0	0	0	0	1	0	0	0	1	1	1
**2.734**	0	1	0	1	1	0	0	0	1	0	0	1	0	1	0	0	0	0	0	1	0	0	0	1	1	1
**2.735**	0	1	0	1	1	0	0	0	1	0	0	1	1	1	0	0	0	0	0	0	0	1	0	1	1	1
**2.736**	0	1	0	1	1	0	0	0	1	1	0	1	1	0	0	0	0	0	0	1	0	1	1	1	0	1
**2.737**	0	1	0	1	1	0	1	0	1	0	0	1	1	0	0	0	0	0	0	1	0	1	1	1	1	1
**2.738**	0	1	1	0	0	0	0	0	0	0	0	0	0	0	0	0	0	0	0	1	0	1	1	1	1	1
**2.739**	0	1	1	0	0	0	0	0	0	0	0	1	0	1	0	0	0	0	0	1	0	1	1	1	1	1
**2.740**	0	1	1	0	0	0	0	0	0	0	0	1	1	0	0	0	0	0	0	0	0	1	0	1	1	1
**2.741**	0	1	1	0	0	0	0	0	0	0	0	1	1	0	0	0	0	0	0	0	0	1	1	1	1	1
**2.742**	0	1	1	0	0	0	0	0	1	0	0	1	0	1	0	0	0	0	0	1	0	1	0	1	1	1
**2.743**	0	1	1	0	0	0	0	0	1	0	0	1	0	1	0	0	0	0	0	1	0	1	1	1	1	1

	CD2	CD3	CD4	CD7	CD8	CD10	CD13	CD18	CD26	CD29	CD36	CD44	CD45RA	CD49f	CD54	CD56	CD57	CD58	CD62L	CD71	CD80	Cytokeratin	Histone_H1	HLA-DQ	HLA-DR	Freq. (Voxel)
**2.744**	0	1	1	0	0	0	0	0	1	0	0	1	1	0	0	0	0	0	0	0	0	0	0	1	1	1
**2.745**	0	1	1	0	1	0	0	0	0	1	0	1	1	0	0	0	0	0	0	0	0	1	1	1	1	1
**2.746**	0	1	1	0	1	0	0	0	0	1	0	1	1	0	0	0	0	0	0	1	0	1	0	1	1	1
**2.747**	0	1	1	0	1	0	0	0	1	1	0	1	1	0	0	0	0	0	0	1	0	1	1	1	1	1
**2.748**	0	1	1	0	1	0	1	0	0	1	0	1	1	0	0	0	0	0	0	0	0	1	0	1	1	1
**2.749**	0	1	1	0	1	0	1	0	1	0	0	0	0	0	0	0	0	0	0	1	0	0	0	1	1	1
**2.750**	0	1	1	1	0	0	0	0	0	0	0	1	0	0	0	0	0	0	0	0	0	0	0	1	1	1
**2.751**	0	1	1	1	0	0	0	0	1	0	0	1	0	1	0	0	0	0	0	1	0	1	0	1	1	1
**2.752**	0	1	1	1	0	0	1	0	0	0	0	1	0	0	0	0	0	0	0	0	0	0	0	1	1	1
**2.753**	0	1	1	1	1	0	0	0	0	0	0	1	1	1	0	0	0	0	0	0	0	1	0	1	1	1
**2.754**	0	0	0	0	0	0	0	0	0	0	0	0	0	1	0	0	0	0	0	1	0	1	1	1	1	1
**2.755**	0	0	0	0	0	0	0	0	0	0	0	0	1	0	0	0	0	0	0	0	0	1	1	1	0	1
**2.756**	0	0	0	0	0	0	0	0	0	1	0	1	1	0	0	0	0	0	0	1	0	0	0	1	1	1
**2.757**	0	0	0	0	0	0	0	0	0	1	1	1	0	1	0	0	0	0	0	1	0	1	1	1	0	1
**2.758**	0	0	0	0	0	0	0	0	0	1	1	1	0	1	0	0	0	0	0	1	0	1	1	1	1	1
**2.759**	0	0	0	0	0	0	1	0	0	1	0	1	1	1	0	0	0	0	0	1	0	0	0	1	0	1
**2.760**	0	0	0	0	1	0	0	0	0	0	0	0	0	0	0	0	0	0	0	0	0	1	1	1	1	1
**2.761**	0	0	0	0	1	0	1	0	0	0	0	0	0	0	0	0	0	0	0	0	0	0	0	1	1	1
**2.762**	0	0	0	0	1	0	1	0	1	1	0	1	0	1	0	0	0	0	0	0	0	1	0	1	1	1
**2.763**	0	0	0	1	0	0	0	0	0	0	0	1	0	0	0	1	0	0	0	0	0	0	0	1	1	1
**2.764**	0	0	0	1	0	0	0	0	0	0	0	1	1	0	0	0	0	0	0	1	0	0	0	1	0	1
**2.765**	0	0	0	1	0	0	0	0	0	1	0	0	1	0	0	0	0	0	0	0	0	0	0	1	1	1
**2.766**	0	0	0	1	0	0	0	0	0	1	0	1	0	1	0	0	0	0	0	1	0	1	0	1	0	1
**2.767**	0	0	0	1	0	0	0	0	0	1	0	1	1	1	0	1	0	0	0	0	0	1	1	1	0	1
**2.768**	0	0	0	1	0	0	0	0	0	1	1	1	0	0	0	0	0	0	0	0	0	0	0	1	1	1
**2.769**	0	0	0	1	0	0	0	0	1	0	0	1	0	1	0	0	0	0	0	1	0	1	0	1	0	1
**2.770**	0	0	0	1	0	0	1	0	0	0	0	1	0	0	0	0	0	0	0	0	0	0	1	1	0	1
**2.771**	0	0	0	1	0	0	1	0	0	1	0	1	1	1	0	1	0	0	0	0	0	0	0	1	1	1
**2.772**	0	0	0	1	0	0	1	0	1	1	0	1	1	1	0	0	0	0	0	0	0	0	0	1	1	1
**2.773**	0	0	1	0	0	0	0	0	0	1	0	0	1	0	0	0	0	0	0	0	0	0	0	1	1	1
**2.774**	0	0	1	0	0	0	0	0	1	0	0	1	1	0	0	0	0	0	0	0	0	0	0	1	0	1
**2.775**	0	0	1	0	0	0	1	0	0	0	0	1	0	0	0	0	0	0	0	0	0	0	1	1	1	1
**2.776**	0	0	1	0	0	0	1	0	0	0	0	1	0	0	0	0	0	0	0	1	0	0	1	1	1	1
**2.777**	0	0	1	0	0	0	1	0	1	1	0	1	0	0	0	1	0	0	0	0	0	0	0	1	1	1
**2.778**	0	0	1	0	1	0	0	0	0	1	0	1	0	1	0	0	0	0	0	1	0	1	0	1	1	1
**2.779**	0	0	1	1	0	0	0	0	0	1	0	1	1	0	0	0	0	0	0	1	0	0	0	1	1	1
**2.780**	0	0	1	1	0	0	1	0	1	1	0	1	1	1	0	1	0	0	0	0	0	0	0	1	1	1
**2.781**	0	1	0	0	0	0	0	0	0	1	0	1	1	0	0	0	0	0	0	1	0	1	1	1	0	1
**2.782**	0	1	0	0	0	0	0	0	1	0	0	0	0	0	0	0	0	0	0	0	0	0	1	1	1	1
**2.783**	0	1	0	0	0	0	0	0	1	0	0	0	0	1	0	0	0	0	0	1	0	1	0	1	1	1
**2.784**	0	1	0	0	0	0	0	0	1	1	0	1	1	0	0	0	0	0	0	1	0	0	0	1	0	1
**2.785**	0	1	0	0	0	0	0	0	1	1	0	1	1	0	0	0	0	0	0	1	0	0	1	1	0	1
**2.786**	0	1	0	0	0	0	0	0	1	1	0	1	1	1	0	0	0	0	0	1	0	0	1	1	0	1
**2.787**	0	1	0	0	0	0	1	0	0	0	0	0	0	0	0	0	0	0	0	0	0	0	1	1	1	1
**2.788**	0	1	0	0	0	0	1	0	0	0	0	1	0	0	0	0	0	0	0	0	0	0	1	1	1	1
**2.789**	0	1	0	0	0	0	1	0	0	0	0	1	0	0	0	1	0	0	0	1	0	0	1	1	1	1
**2.790**	0	1	0	0	1	0	0	0	0	0	0	1	1	1	0	0	0	0	0	1	0	1	0	1	1	1
**2.791**	0	1	0	0	1	0	0	0	0	1	0	1	1	0	0	0	0	0	0	1	0	1	1	1	0	1
**2.792**	0	1	0	0	1	0	0	0	0	1	1	1	0	0	0	0	0	0	0	0	0	0	0	1	0	1
**2.793**	0	1	0	0	1	0	0	0	1	0	0	1	0	0	0	0	0	0	0	1	0	1	0	1	0	1
**2.794**	0	1	0	0	1	0	0	0	1	0	1	1	0	0	0	0	0	0	0	0	0	0	1	1	0	1
**2.795**	0	1	0	0	1	0	0	0	1	1	0	1	0	1	0	0	0	0	0	0	0	1	0	1	1	1
**2.796**	0	1	0	1	0	0	0	0	0	0	0	1	1	0	0	0	0	0	0	0	0	0	1	1	0	1
**2.797**	0	1	0	1	0	0	0	0	0	0	0	1	1	0	0	0	0	0	0	1	0	0	0	1	1	1
**2.798**	0	1	0	1	0	0	0	0	1	0	0	1	1	0	0	0	0	0	0	0	0	0	1	1	0	1
**2.799**	0	1	0	1	0	0	0	0	1	0	0	1	1	0	0	0	0	0	0	1	0	1	0	1	0	1
**2.800**	0	1	0	1	0	0	0	0	1	0	0	1	1	0	0	0	0	0	0	1	0	1	0	1	1	1
**2.801**	0	1	0	1	0	0	0	0	1	1	0	1	0	0	0	0	0	0	0	1	0	0	1	1	0	1
**2.802**	0	1	0	1	0	0	1	0	0	0	0	1	0	0	0	1	0	0	0	1	0	0	0	1	1	1
**2.803**	0	1	0	1	0	0	1	0	0	0	0	1	1	1	0	0	0	0	0	1	0	1	0	1	0	1
**2.804**	0	1	0	1	0	0	1	0	0	0	0	1	1	1	0	0	0	0	0	1	0	1	1	1	1	1
**2.805**	0	1	0	1	0	0	1	0	1	0	0	1	0	0	0	0	0	0	0	0	0	0	1	1	1	1
**2.806**	0	1	0	1	0	0	1	0	1	0	0	1	0	1	0	0	0	0	0	1	0	1	0	1	1	1
**2.807**	0	1	0	1	1	0	0	0	0	0	0	0	1	0	0	0	0	0	0	0	0	0	1	1	0	1
**2.808**	0	1	0	1	1	0	0	0	0	0	0	1	0	1	0	0	0	0	0	1	0	0	0	1	0	1
**2.809**	0	1	0	1	1	0	0	0	0	0	0	1	1	0	0	0	0	0	0	1	0	0	1	1	1	1
**2.810**	0	1	0	1	1	0	0	0	0	1	0	1	1	0	0	0	0	0	0	0	0	1	0	1	0	1
**2.811**	0	1	0	1	1	0	0	0	0	1	1	1	0	0	0	0	0	0	0	0	0	0	0	1	0	1
**2.812**	0	1	0	1	1	0	0	0	1	0	0	1	0	0	0	1	0	0	0	1	0	1	0	1	1	1
**2.813**	0	1	0	1	1	0	0	0	1	0	0	1	1	0	0	0	0	0	0	1	0	1	1	1	0	1
**2.814**	0	1	0	1	1	0	0	0	1	0	0	1	1	1	0	0	0	0	0	1	0	0	1	1	0	1
**2.815**	0	1	0	1	1	0	0	0	1	1	0	1	0	0	0	0	0	0	0	1	0	0	1	1	1	1
**2.816**	0	1	0	1	1	0	1	0	1	0	0	1	0	0	0	0	0	0	0	1	0	1	0	1	1	1
**2.817**	0	1	1	0	0	0	0	0	1	0	0	1	0	0	0	0	0	0	0	0	0	0	1	1	1	1
**2.818**	0	1	1	0	0	0	0	0	1	1	0	1	1	0	0	0	0	0	0	1	0	0	0	1	1	1
**2.819**	0	1	1	0	0	0	0	0	1	1	0	1	1	1	0	0	0	0	0	1	0	0	0	1	1	1
**2.820**	0	1	1	0	1	0	0	0	0	0	0	1	0	0	0	0	0	0	0	1	0	1	1	1	0	1
**2.821**	0	1	1	0	1	0	0	0	0	1	0	1	0	0	0	0	0	0	0	0	0	1	0	1	1	1
**2.822**	0	1	1	0	1	0	0	0	1	0	0	1	1	0	0	1	0	0	0	1	0	0	0	1	1	1
**2.823**	0	1	1	0	1	0	0	0	1	1	0	1	1	0	0	0	0	0	0	1	0	1	0	1	0	1
**2.824**	0	1	1	1	0	0	0	0	0	1	0	0	1	0	0	0	0	0	0	0	0	0	1	1	0	1
**2.825**	0	1	1	1	0	0	0	0	1	0	0	1	0	0	0	0	0	0	0	1	0	1	0	1	1	1
**2.826**	0	1	1	1	0	0	0	0	1	1	0	1	1	1	0	0	0	0	0	1	0	0	0	1	0	1
**2.827**	0	1	1	1	1	0	0	0	0	1	0	0	1	0	0	0	0	0	0	0	0	0	0	1	1	1
**2.828**	0	1	1	1	1	0	0	0	1	0	0	1	1	0	0	1	0	0	0	1	0	0	0	1	1	1
**2.829**	0	0	0	0	0	0	0	0	0	0	0	0	0	0	0	1	0	0	0	1	0	0	1	1	0	1
**2.830**	0	0	0	0	0	0	0	0	0	0	0	0	0	1	0	1	0	0	0	1	0	0	0	1	1	1
**2.831**	0	0	0	0	0	0	0	0	0	0	0	0	1	0	0	0	0	0	0	1	0	0	1	1	0	1
**2.832**	0	0	0	0	0	0	0	0	0	0	0	1	0	0	0	1	0	0	0	1	0	0	1	1	0	1
**2.833**	0	0	0	0	0	0	0	0	0	0	0	1	0	1	0	1	0	0	0	1	0	1	0	1	1	1
**2.834**	0	0	0	0	0	0	0	0	0	1	1	1	0	1	0	0	0	0	0	0	0	1	1	1	1	1
**2.835**	0	0	0	0	0	0	0	0	1	1	0	0	0	0	0	1	0	0	0	0	0	0	0	1	1	1
**2.836**	0	0	0	0	0	0	1	0	0	0	0	0	0	1	0	1	0	0	0	0	0	0	0	1	0	1
**2.837**	0	0	0	0	0	0	1	0	0	1	0	0	0	1	0	1	0	0	0	0	0	0	0	1	0	1
**2.838**	0	0	0	0	0	0	1	0	0	1	0	1	0	0	0	0	0	0	0	0	0	1	1	1	1	1
**2.839**	0	0	0	0	0	0	1	0	1	0	0	1	0	0	0	0	0	0	0	1	0	0	0	1	0	1
**2.840**	0	0	0	0	0	0	1	0	1	1	0	0	0	0	0	0	0	0	0	0	0	0	0	1	1	1
**2.841**	0	0	0	0	0	0	1	0	1	1	0	1	0	1	0	0	0	0	0	1	0	0	0	1	0	1

	CD2	CD3	CD4	CD7	CD8	CD10	CD13	CD18	CD26	CD29	CD36	CD44	CD45RA	CD49f	CD54	CD56	CD57	CD58	CD62L	CD71	CD80	Cytokeratin	Histone_H1	HLA-DQ	HLA-DR	Freq. (Voxel)
**2.842**	0	0	0	0	1	0	0	0	0	1	0	1	0	1	0	0	0	0	0	1	0	0	0	1	0	1
**2.843**	0	0	0	0	1	0	1	0	0	1	0	1	1	1	0	0	0	0	0	1	0	1	0	1	1	1
**2.844**	0	0	0	0	1	0	1	0	1	0	0	0	0	0	0	0	0	0	0	0	0	0	0	1	1	1
**2.845**	0	0	0	0	1	0	1	0	1	1	0	0	0	0	0	1	0	0	0	0	0	0	0	1	1	1
**2.846**	0	0	0	1	0	0	0	0	0	0	0	0	0	1	0	0	0	0	0	1	0	1	0	1	1	1
**2.847**	0	0	0	1	0	0	0	0	0	0	0	0	0	1	0	1	0	0	0	1	0	0	0	1	1	1
**2.848**	0	0	0	1	0	0	0	0	0	1	0	1	0	1	0	1	0	0	0	1	0	1	1	1	0	1
**2.849**	0	0	0	1	0	0	1	0	0	1	0	1	0	1	0	0	0	0	0	0	0	0	1	1	1	1
**2.850**	0	0	0	1	1	0	1	0	1	1	0	1	0	1	0	0	0	0	0	1	0	0	0	1	1	1
**2.851**	0	0	1	0	0	0	0	0	0	1	0	0	0	1	0	0	0	0	0	1	0	0	1	1	0	1
**2.852**	0	0	1	0	0	0	0	0	1	1	0	1	1	1	0	0	0	0	0	1	0	1	0	1	0	1
**2.853**	0	0	1	0	0	0	0	0	1	1	0	1	1	1	0	0	0	0	0	1	0	1	0	1	1	1
**2.854**	0	0	1	0	0	0	1	0	0	0	0	1	0	1	0	0	0	0	0	1	0	0	1	1	1	1
**2.855**	0	0	1	0	0	0	1	0	0	1	0	0	0	1	0	0	0	0	0	1	0	0	0	1	0	1
**2.856**	0	0	1	0	0	0	1	0	0	1	0	0	1	1	0	0	0	0	0	0	0	1	1	1	0	1
**2.857**	0	0	1	0	1	0	0	0	0	0	0	1	0	0	0	0	0	0	0	1	0	0	0	1	0	1
**2.858**	0	0	1	0	1	0	0	0	0	1	0	1	0	0	0	0	0	0	0	0	0	1	0	1	1	1
**2.859**	0	0	1	0	1	0	1	0	0	0	0	0	0	1	0	0	0	0	0	1	0	0	0	1	1	1
**2.860**	0	0	1	0	1	0	1	0	0	0	0	0	1	1	0	1	0	0	0	1	0	1	0	1	0	1
**2.861**	0	0	1	0	1	0	1	0	1	1	0	0	0	0	0	0	0	0	0	0	0	0	0	1	1	1
**2.862**	0	0	1	0	1	0	1	0	1	1	0	0	0	0	0	1	0	0	0	0	0	0	0	1	1	1
**2.863**	0	0	1	1	0	0	0	0	0	0	0	0	0	0	0	0	0	0	0	0	0	0	0	1	1	1
**2.864**	0	0	1	1	0	0	0	0	0	0	0	0	1	1	0	1	0	0	0	1	0	0	0	1	0	1
**2.865**	0	0	1	1	0	0	0	0	0	0	0	0	1	1	0	1	0	0	0	1	0	1	0	1	0	1
**2.866**	0	1	0	0	0	0	0	0	0	0	0	1	1	1	0	0	0	0	0	0	0	1	1	1	0	1
**2.867**	0	1	0	0	0	0	0	0	0	0	0	1	1	1	0	0	0	0	0	1	0	1	0	1	1	1
**2.868**	0	1	0	0	0	0	0	0	0	1	0	1	0	1	0	0	0	0	0	0	0	1	0	1	0	1
**2.869**	0	1	0	0	0	0	0	0	0	1	0	1	0	1	0	0	0	0	0	0	0	1	1	1	1	1
**2.870**	0	1	0	0	0	0	0	0	0	1	0	1	0	1	0	0	0	0	0	1	0	1	1	1	1	1
**2.871**	0	1	0	0	0	0	0	0	1	0	0	1	1	1	0	0	0	0	0	0	0	1	0	1	1	1
**2.872**	0	1	0	0	0	0	0	0	1	1	0	1	1	0	0	0	0	0	0	0	0	1	0	1	0	1
**2.873**	0	1	0	0	0	0	1	0	0	0	0	1	0	1	0	0	0	0	0	0	0	1	0	1	1	1
**2.874**	0	1	0	0	1	0	0	0	0	0	0	0	0	1	0	0	0	0	0	1	0	1	0	1	1	1
**2.875**	0	1	0	0	1	0	0	0	0	0	0	1	1	0	0	0	0	0	0	1	0	0	1	1	0	1
**2.876**	0	1	0	0	1	0	0	0	1	0	0	1	1	0	0	0	0	0	0	1	0	1	0	1	0	1
**2.877**	0	1	0	0	1	0	0	0	1	1	0	1	0	1	0	0	0	0	0	1	0	0	0	1	0	1
**2.878**	0	1	0	0	1	0	1	0	0	1	0	1	0	0	0	0	0	0	0	0	0	1	0	1	1	1
**2.879**	0	1	0	0	1	0	1	0	1	1	0	1	0	1	0	0	0	0	0	1	0	0	0	1	0	1
**2.880**	0	1	0	1	0	0	0	0	0	0	0	1	0	1	0	0	0	0	0	1	0	1	0	1	0	1
**2.881**	0	1	0	1	0	0	0	0	0	1	0	0	1	0	0	0	0	0	0	0	0	0	1	1	0	1
**2.882**	0	1	0	1	0	0	0	0	0	1	0	1	0	0	0	0	0	0	0	1	0	1	0	1	1	1
**2.883**	0	1	0	1	0	0	0	0	0	1	1	1	0	1	0	0	0	0	0	1	0	1	0	1	1	1
**2.884**	0	1	0	1	0	0	1	0	0	0	0	0	0	1	0	0	0	0	0	0	0	1	1	1	1	1
**2.885**	0	1	0	1	0	0	1	0	0	0	0	0	1	1	0	0	0	0	0	1	0	1	0	1	1	1
**2.886**	0	1	0	1	0	0	1	0	0	0	0	0	1	1	0	0	0	0	0	1	0	1	1	1	1	1
**2.887**	0	1	0	1	1	0	0	0	0	0	0	0	0	1	0	0	0	0	0	1	0	0	0	1	1	1
**2.888**	0	1	0	1	1	0	0	0	0	1	0	0	1	0	0	0	0	0	0	0	0	0	1	1	0	1
**2.889**	0	1	0	1	1	0	0	0	0	1	0	0	1	0	0	0	0	0	0	1	0	0	1	1	1	1
**2.890**	0	1	0	1	1	0	0	0	1	0	0	0	0	0	0	0	0	0	0	1	0	1	1	1	1	1
**2.891**	0	1	0	1	1	0	0	0	1	1	0	1	1	1	0	0	0	0	0	1	0	0	0	1	0	1
**2.892**	0	1	0	1	1	0	1	0	0	1	0	1	0	0	0	0	0	0	0	1	0	0	0	1	0	1
**2.893**	0	1	1	0	0	0	0	0	0	0	0	1	0	0	0	0	0	0	0	1	0	1	0	1	0	1
**2.894**	0	1	1	0	0	0	1	0	1	0	0	1	0	0	0	0	0	0	0	0	0	0	0	1	1	1
**2.895**	0	1	1	0	1	0	0	0	0	0	0	1	0	0	0	0	0	0	0	1	0	1	1	1	1	1
**2.896**	0	1	1	1	1	0	0	0	0	0	0	0	1	0	0	0	0	0	0	1	0	0	0	1	1	1
**2.897**	0	1	1	1	1	0	0	0	0	0	0	1	1	0	0	0	0	0	0	1	0	1	0	1	0	1
**2.898**	0	1	1	1	1	0	0	0	0	1	0	0	1	0	0	0	0	0	0	0	0	0	1	1	1	1
**2.899**	0	1	1	1	1	0	0	0	0	1	0	0	1	0	0	0	0	0	0	1	0	0	1	1	1	1
**2.900**	0	0	0	0	0	0	0	0	0	0	0	1	1	1	0	0	0	0	0	0	0	1	0	1	0	1
**2.901**	0	0	0	0	0	0	0	0	0	1	0	1	0	0	0	1	0	0	0	0	0	1	0	1	1	1
**2.902**	0	0	0	0	0	0	0	0	1	0	0	1	0	1	0	1	0	0	0	1	0	1	0	1	0	1
**2.903**	0	0	0	0	0	0	1	0	0	1	0	0	0	0	0	1	0	0	0	0	0	0	0	1	0	1
**2.904**	0	0	0	0	0	0	1	0	0	1	0	0	1	0	0	0	0	0	0	0	0	0	0	1	0	1
**2.905**	0	0	0	0	1	0	0	0	0	0	0	1	1	0	0	0	0	0	0	1	0	0	0	1	0	1
**2.906**	0	0	0	0	1	0	0	0	0	1	0	1	0	1	0	0	0	0	0	0	0	1	1	1	0	1
**2.907**	0	0	0	0	1	0	1	0	1	1	0	1	0	1	0	0	0	0	0	1	0	1	0	1	0	1
**2.908**	0	0	0	1	0	0	0	0	0	0	0	0	1	1	0	1	0	0	0	0	0	1	0	1	0	1
**2.909**	0	0	0	1	0	0	0	0	0	0	0	0	1	1	0	1	0	0	0	1	0	1	0	1	0	1
**2.910**	0	0	0	1	0	0	1	0	1	0	0	0	0	1	0	0	0	0	0	0	0	1	0	1	1	1
**2.911**	0	0	0	1	0	0	1	0	1	1	0	0	0	1	0	0	0	0	0	0	0	1	0	1	1	1
**2.912**	0	0	1	0	0	0	0	0	0	0	1	1	0	1	0	0	0	0	0	1	0	1	0	1	1	1
**2.913**	0	0	1	0	0	0	0	0	0	1	0	0	0	0	0	0	0	0	0	0	0	1	1	1	1	1
**2.914**	0	0	1	0	0	0	0	0	0	1	0	0	0	1	0	1	0	0	0	1	0	0	0	1	0	1
**2.915**	0	0	1	0	0	0	1	0	0	0	0	0	1	1	0	0	0	0	0	0	0	0	0	1	0	1
**2.916**	0	0	1	0	0	0	1	0	1	0	0	0	1	0	0	0	0	0	0	0	0	0	0	1	1	1
**2.917**	0	0	1	0	0	0	1	0	1	0	0	0	1	0	0	1	0	0	0	0	0	1	0	1	0	1
**2.918**	0	0	1	0	0	0	1	0	1	0	1	1	0	1	0	0	0	0	0	1	0	1	0	1	1	1
**2.919**	0	0	1	0	1	0	1	0	0	0	0	0	1	0	0	0	0	0	0	1	0	0	0	1	1	1
**2.920**	0	0	1	0	1	0	1	0	0	0	0	0	1	0	0	1	0	0	0	0	0	1	0	1	0	1
**2.921**	0	0	1	0	1	0	1	0	1	0	0	0	1	0	0	1	0	0	0	1	0	1	0	1	0	1
**2.922**	0	0	1	1	0	0	0	0	0	0	0	0	0	0	0	0	0	0	0	1	0	1	0	1	0	1
**2.923**	0	0	1	1	0	0	0	0	0	0	0	0	0	1	0	0	0	0	0	0	0	0	0	1	1	1
**2.924**	0	0	1	1	0	0	0	0	0	1	0	1	0	1	0	0	0	0	0	1	0	1	0	1	0	1
**2.925**	0	1	0	0	0	0	0	0	0	0	0	0	0	0	0	0	0	0	0	1	0	1	0	1	0	1
**2.926**	0	1	0	0	0	0	0	0	0	0	0	0	1	0	0	0	0	0	0	0	0	1	1	1	1	1
**2.927**	0	1	0	0	0	0	1	0	0	1	0	1	0	0	0	0	0	0	0	0	0	0	1	1	0	1
**2.928**	0	1	0	0	0	0	1	0	0	1	0	1	0	1	0	0	0	0	0	1	0	1	0	1	0	1
**2.929**	0	1	0	0	1	0	0	0	0	0	0	0	1	0	0	0	0	0	0	1	0	1	1	1	1	1
**2.930**	0	1	0	0	1	0	0	0	0	1	0	1	1	1	0	0	0	0	0	0	0	0	0	1	0	1
**2.931**	0	1	0	0	1	0	1	0	0	1	0	1	0	1	0	0	0	0	0	1	0	1	0	1	0	1
**2.932**	0	1	0	1	0	0	0	0	0	0	0	0	0	0	0	0	0	0	0	0	0	1	1	1	1	1
**2.933**	0	1	0	1	0	0	0	0	0	0	0	1	1	1	0	0	0	0	0	0	0	0	0	1	1	1
**2.934**	0	1	0	1	0	0	0	0	0	1	0	0	0	0	0	0	0	0	0	0	0	0	1	1	0	1
**2.935**	0	1	0	1	0	0	1	0	0	1	0	1	0	0	0	0	0	0	0	1	0	0	0	1	1	1
**2.936**	0	1	0	1	1	0	0	0	0	0	0	0	1	0	0	0	0	0	0	0	0	1	1	1	0	1
**2.937**	0	1	0	1	1	0	0	0	0	0	0	1	0	1	0	0	0	0	0	1	0	1	0	1	0	1
**2.938**	0	1	0	1	1	0	0	0	0	1	0	1	0	1	0	0	0	0	0	1	0	1	1	1	1	1
**2.939**	0	1	0	1	1	0	0	0	0	1	0	1	1	1	0	0	0	0	0	0	0	0	0	1	0	1

	CD2	CD3	CD4	CD7	CD8	CD10	CD13	CD18	CD26	CD29	CD36	CD44	CD45RA	CD49f	CD54	CD56	CD57	CD58	CD62L	CD71	CD80	Cytokeratin	Histone_H1	HLA-DQ	HLA-DR	Freq. (Voxel)
**2.940**	0	1	0	1	1	0	1	0	0	1	0	1	0	1	0	0	0	0	0	0	0	1	0	1	1	1
**2.941**	0	1	1	0	0	0	0	0	0	1	0	1	0	1	0	0	0	0	0	1	0	0	0	1	1	1
**2.942**	0	1	1	0	0	0	0	0	1	1	0	1	0	0	0	0	0	0	0	0	0	0	0	1	0	1
**2.943**	0	1	1	0	0	0	0	0	1	1	0	1	0	0	0	1	0	0	0	0	0	0	0	1	1	1
**2.944**	0	1	1	0	1	0	0	0	0	1	0	1	0	1	0	1	0	0	0	1	0	0	0	1	1	1
**2.945**	0	1	1	0	1	0	0	0	1	1	0	1	0	1	0	0	0	0	0	1	0	0	0	1	0	1
**2.946**	0	1	1	0	1	0	1	0	0	1	0	0	0	0	0	0	0	0	0	0	0	0	0	1	1	1
**2.947**	0	1	1	1	0	0	0	0	1	0	0	1	1	0	0	0	0	0	0	1	0	0	0	1	1	1
**2.948**	0	1	1	1	0	0	0	0	1	1	0	1	1	1	0	0	0	0	0	1	0	0	0	1	1	1
**2.949**	0	1	1	1	1	0	0	0	0	1	0	1	0	0	0	0	0	0	0	0	0	0	0	1	1	1
**2.950**	0	1	1	1	1	0	0	0	0	1	0	1	1	0	0	0	0	0	0	0	0	0	0	1	1	1
**2.951**	0	1	1	1	1	0	0	0	0	1	0	1	1	1	0	0	0	0	0	0	0	0	0	1	0	1
**2.952**	0	1	1	1	1	0	0	0	0	1	0	1	1	1	0	0	0	0	0	0	0	0	0	1	1	1
**2.953**	0	1	1	1	1	0	0	0	0	1	0	1	1	1	0	0	0	0	0	1	0	0	0	1	0	1
**2.954**	0	1	1	1	1	0	1	0	0	1	0	1	0	1	0	0	0	0	0	1	0	0	0	1	1	1
**2.955**	0	0	0	0	0	0	1	0	0	0	0	1	0	1	0	1	0	0	0	1	0	0	0	1	1	1
**2.956**	0	0	0	0	0	0	1	0	0	1	0	1	0	0	0	1	0	0	0	1	0	0	0	1	1	1
**2.957**	0	0	0	0	0	0	1	0	0	1	0	1	1	1	0	0	0	0	0	1	0	1	0	1	0	1
**2.958**	0	0	0	0	0	0	1	0	0	1	1	0	0	0	0	0	0	0	0	0	0	0	1	1	1	1
**2.959**	0	0	0	0	0	0	1	0	1	1	0	0	0	0	0	0	0	0	0	0	0	0	1	1	1	1
**2.960**	0	0	0	0	0	0	1	0	1	1	0	1	0	1	0	0	0	0	0	1	0	1	1	1	0	1
**2.961**	0	0	0	1	0	0	1	0	0	1	0	1	0	1	0	0	0	0	0	0	0	1	0	1	1	1
**2.962**	0	0	1	0	0	0	0	0	0	0	0	0	1	0	0	0	0	0	0	0	0	0	1	1	1	1
**2.963**	0	0	1	0	0	0	1	0	0	0	0	1	0	0	0	0	0	0	0	0	0	0	1	1	0	1
**2.964**	0	0	1	0	0	0	1	0	0	0	0	1	0	1	0	0	0	0	0	0	0	0	1	1	1	1
**2.965**	0	0	1	0	0	0	1	0	0	0	0	1	0	1	0	0	0	0	0	0	0	1	0	1	0	1
**2.966**	0	0	1	0	0	0	1	0	0	1	0	0	0	1	0	0	0	0	0	1	0	1	0	1	0	1
**2.967**	0	0	1	0	0	0	1	0	0	1	0	1	1	1	0	0	0	0	0	1	0	1	1	1	1	1
**2.968**	0	0	1	0	0	0	1	0	1	1	0	1	0	1	0	0	0	0	0	0	0	0	0	1	0	1
**2.969**	0	0	1	0	0	0	1	0	1	1	0	1	1	1	0	0	0	0	0	1	0	1	1	1	1	1
**2.970**	0	0	1	0	1	0	0	0	0	1	0	0	0	0	0	0	0	0	0	0	0	0	1	1	1	1
**2.971**	0	0	1	0	1	0	1	0	0	1	0	1	0	1	0	0	0	0	0	0	0	1	0	1	1	1
**2.972**	0	0	1	1	0	0	0	0	0	0	0	0	0	0	0	0	0	0	0	0	0	1	0	1	0	1
**2.973**	0	0	1	1	0	0	0	0	0	0	0	0	0	1	0	0	0	0	0	1	0	1	0	1	0	1
**2.974**	0	0	1	1	0	0	0	0	0	0	0	0	1	0	0	0	0	0	0	0	0	1	0	1	0	1
**2.975**	0	0	1	1	0	0	0	0	0	0	1	1	0	1	0	0	0	0	0	1	0	1	0	1	0	1
**2.976**	0	0	1	1	0	0	1	0	0	1	0	1	1	1	0	0	0	0	0	1	0	1	1	1	1	1
**2.977**	0	0	1	1	0	0	1	0	1	1	0	1	0	1	0	0	0	0	0	1	0	1	1	1	0	1
**2.978**	0	1	0	0	0	0	0	0	0	0	0	1	1	0	0	0	0	0	0	1	0	0	0	1	0	1
**2.979**	0	1	0	0	0	0	0	0	0	1	0	0	0	0	0	0	0	0	0	0	0	1	1	1	0	1
**2.980**	0	1	0	0	0	0	0	0	1	0	0	1	1	0	0	0	0	0	0	1	0	0	0	1	0	1
**2.981**	0	1	0	0	1	0	0	0	0	0	0	0	0	1	0	0	0	0	0	0	0	1	0	1	1	1
**2.982**	0	1	0	0	1	0	0	0	0	0	0	0	1	0	0	0	0	0	0	0	0	1	1	1	0	1
**2.983**	0	1	0	0	1	0	0	0	0	1	0	1	0	1	0	0	0	0	0	1	0	0	0	1	0	1
**2.984**	0	1	0	0	1	0	0	0	0	1	0	1	0	1	0	0	0	0	0	1	0	0	1	1	1	1
**2.985**	0	1	0	0	1	0	1	0	0	1	0	1	1	0	0	0	0	0	0	0	0	0	0	1	1	1
**2.986**	0	1	0	1	0	0	0	0	0	0	0	0	0	0	0	0	0	0	0	0	0	1	1	1	0	1
**2.987**	0	1	0	1	0	0	0	0	0	1	0	1	1	1	0	0	0	0	0	0	0	0	0	1	0	1
**2.988**	0	1	0	1	0	0	0	0	0	1	0	1	1	1	0	0	0	0	0	1	0	0	0	1	1	1
**2.989**	0	1	0	1	0	0	0	0	1	1	0	1	1	1	0	0	0	0	0	0	0	0	0	1	0	1
**2.990**	0	1	0	1	1	0	0	0	1	0	0	0	1	0	0	0	0	0	0	0	0	0	0	1	0	1
**2.991**	0	1	1	0	0	0	0	0	0	1	0	1	0	1	0	1	0	0	0	0	0	0	0	1	1	1
**2.992**	0	1	1	0	0	0	0	0	0	1	0	1	1	1	0	0	0	0	0	0	0	0	0	1	0	1
**2.993**	0	1	1	0	1	0	0	0	0	0	0	0	0	0	0	0	0	0	0	1	0	0	1	1	0	1
**2.994**	0	1	1	0	1	0	0	0	0	1	0	0	0	0	0	0	0	0	0	0	0	0	1	1	1	1
**2.995**	0	1	1	0	1	0	0	0	0	1	0	1	0	0	0	0	0	0	0	0	0	0	0	1	0	1
**2.996**	0	1	1	0	1	0	0	0	0	1	0	1	0	1	0	0	0	0	0	0	0	0	0	1	0	1
**2.997**	0	1	1	0	1	0	0	0	0	1	0	1	0	1	0	1	0	0	0	0	0	0	0	1	1	1
**2.998**	0	1	1	0	1	0	0	0	1	0	0	1	1	0	0	0	0	0	0	0	0	0	0	1	1	1
**2.999**	0	1	1	1	0	0	0	0	1	1	0	0	0	0	0	0	0	0	0	0	0	0	0	1	0	1
**3.000**	0	0	0	0	0	0	0	0	0	0	0	0	0	1	0	1	0	0	0	0	0	0	0	1	1	1
**3.001**	0	0	0	0	0	0	0	0	0	1	0	0	0	0	0	1	0	0	0	0	0	1	0	1	1	1
**3.002**	0	0	0	0	0	0	0	0	1	1	0	0	0	1	0	0	0	0	0	0	0	0	1	1	0	1
**3.003**	0	0	0	0	0	0	1	0	0	1	0	1	0	1	0	1	0	0	0	0	0	0	0	1	0	1
**3.004**	0	0	0	0	0	0	1	0	0	1	1	1	0	1	0	1	0	0	0	1	0	0	0	1	1	1
**3.005**	0	0	0	0	0	0	1	0	1	1	0	1	0	1	0	0	0	0	0	0	0	0	0	1	0	1
**3.006**	0	0	0	0	0	0	1	0	1	1	1	1	1	1	0	0	0	0	0	0	0	0	0	1	1	1
**3.007**	0	0	0	0	1	0	0	0	0	1	0	1	0	1	0	0	0	0	0	1	0	1	1	1	1	1
**3.008**	0	0	0	1	0	0	1	0	0	1	0	1	1	1	0	0	0	0	0	1	0	1	0	1	1	1
**3.009**	0	0	0	1	0	0	1	0	1	1	1	1	1	1	0	0	0	0	0	1	0	0	0	1	1	1
**3.010**	0	0	0	1	1	0	0	0	0	0	0	0	1	0	0	0	0	0	0	0	0	0	0	1	0	1
**3.011**	0	0	1	0	0	0	0	0	0	0	1	0	0	1	0	0	0	0	0	1	0	1	0	1	0	1
**3.012**	0	0	1	0	0	0	0	0	1	0	0	0	1	1	0	0	0	0	0	0	0	0	0	1	0	1
**3.013**	0	0	1	0	0	0	0	0	1	0	0	1	0	0	0	0	0	0	0	0	0	1	1	1	0	1
**3.014**	0	0	1	0	0	0	0	0	1	0	0	1	0	0	0	1	0	0	0	0	0	0	0	1	1	1
**3.015**	0	0	1	0	0	0	0	0	1	1	0	1	0	0	0	0	0	0	0	0	0	0	1	1	0	1
**3.016**	0	0	1	0	0	0	1	0	0	0	1	1	0	1	0	0	0	0	0	1	0	1	0	1	0	1
**3.017**	0	0	1	0	0	0	1	0	1	0	0	0	1	1	0	0	0	0	0	1	0	0	0	1	1	1
**3.018**	0	0	1	1	0	0	0	0	0	0	0	1	1	1	0	1	0	0	0	1	0	1	0	1	1	1
**3.019**	0	0	1	1	0	0	1	0	0	0	0	0	1	0	0	1	0	0	0	0	0	0	0	1	0	1
**3.020**	0	0	1	1	0	0	1	0	1	1	0	1	1	1	0	0	0	0	0	1	0	1	1	1	1	1
**3.021**	0	1	0	0	0	0	0	0	0	0	0	0	0	1	0	0	0	0	0	1	0	0	1	1	0	1
**3.022**	0	1	0	0	0	0	0	0	0	0	0	0	0	1	0	0	0	0	0	1	0	0	1	1	1	1
**3.023**	0	1	0	0	0	0	0	0	0	0	1	0	0	1	0	0	0	0	0	0	0	1	0	1	1	1
**3.024**	0	1	0	0	0	0	0	0	0	0	1	1	0	1	0	0	0	0	0	0	0	0	0	1	1	1
**3.025**	0	1	0	0	0	0	0	0	0	0	1	1	0	1	0	0	0	0	0	0	0	1	0	1	0	1
**3.026**	0	1	0	0	0	0	0	0	0	1	0	1	1	1	0	1	0	0	0	0	0	0	0	1	0	1
**3.027**	0	1	0	0	0	0	0	0	1	0	0	0	1	1	0	0	0	0	0	0	0	0	0	1	1	1
**3.028**	0	1	0	0	0	0	0	0	1	0	0	1	1	0	0	0	0	0	0	0	0	0	1	1	1	1
**3.029**	0	1	0	0	0	0	1	0	0	0	1	1	0	0	0	0	0	0	0	0	0	1	0	1	1	1
**3.030**	0	1	0	0	0	0	1	0	0	1	0	1	0	0	0	0	0	0	0	1	0	0	1	1	1	1
**3.031**	0	1	0	0	1	0	0	0	0	0	0	1	0	1	0	0	0	0	0	1	0	1	0	1	0	1
**3.032**	0	1	0	0	1	0	0	0	0	1	0	0	0	0	0	0	0	0	0	1	0	0	1	1	1	1
**3.033**	0	1	0	0	1	0	0	0	1	0	0	0	1	0	0	0	0	0	0	1	0	0	1	1	1	1
**3.034**	0	1	0	0	1	0	0	0	1	1	0	0	1	0	0	0	0	0	0	0	0	0	0	1	0	1
**3.035**	0	1	0	1	0	0	0	0	0	0	0	0	0	1	0	0	0	0	0	0	0	0	0	1	0	1
**3.036**	0	1	0	1	0	0	0	0	0	0	0	0	0	1	0	0	0	0	0	0	0	0	1	1	0	1
**3.037**	0	1	0	1	0	0	0	0	0	1	0	0	0	1	0	0	0	0	0	0	0	0	0	1	0	1

	CD2	CD3	CD4	CD7	CD8	CD10	CD13	CD18	CD26	CD29	CD36	CD44	CD45RA	CD49f	CD54	CD56	CD57	CD58	CD62L	CD71	CD80	Cytokeratin	Histone_H1	HLA-DQ	HLA-DR	Freq. (Voxel)
**3.038**	0	1	0	1	0	0	0	0	0	1	0	1	0	0	0	0	0	0	0	1	0	0	1	1	1	1
**3.039**	0	1	0	1	0	0	0	0	1	1	0	0	0	0	0	0	0	0	0	0	0	0	0	1	0	1
**3.040**	0	1	1	0	0	0	0	0	0	0	0	1	0	1	0	0	0	0	0	0	0	0	0	1	1	1
**3.041**	0	1	1	0	0	0	1	0	0	1	0	1	0	1	0	0	0	0	0	0	0	1	0	1	1	1
**3.042**	0	1	1	0	1	0	0	0	0	1	0	1	0	0	0	0	0	0	0	0	0	0	1	1	0	1
**3.043**	0	1	1	1	0	0	0	0	0	1	0	1	0	0	0	0	0	0	0	0	0	0	0	1	0	1
**3.044**	0	1	1	1	1	0	0	0	0	1	0	1	1	0	0	0	0	0	0	0	0	0	0	1	0	1
**3.045**	0	0	0	0	0	0	0	0	0	0	0	1	0	0	0	1	0	0	0	1	0	0	0	1	0	1
**3.046**	0	0	0	0	0	0	0	0	0	0	0	1	0	1	0	1	0	0	0	0	0	0	0	1	1	1
**3.047**	0	0	0	0	0	0	0	0	0	1	0	0	1	0	0	1	0	0	0	0	0	0	1	1	0	1
**3.048**	0	0	0	0	0	0	0	0	0	1	0	0	1	1	0	0	0	0	0	0	0	0	0	1	1	1
**3.049**	0	0	0	0	0	0	0	0	0	1	0	0	1	1	0	0	0	0	0	0	0	1	0	1	1	1
**3.050**	0	0	0	0	0	0	0	0	0	1	0	1	0	1	0	1	0	0	0	0	0	0	0	1	1	1
**3.051**	0	0	0	0	0	0	0	0	0	1	0	1	0	1	0	1	0	0	0	0	0	1	0	1	1	1
**3.052**	0	0	0	0	0	0	0	0	1	0	0	0	1	1	0	0	0	0	0	0	0	0	1	1	0	1
**3.053**	0	0	0	0	0	0	1	0	0	0	0	0	0	0	0	0	0	0	0	0	0	1	1	1	0	1
**3.054**	0	0	0	0	0	0	1	0	0	0	1	0	0	0	0	0	0	0	0	0	0	1	0	1	0	1
**3.055**	0	0	0	0	0	0	1	0	0	0	1	0	0	1	0	1	0	0	0	0	0	1	0	1	0	1
**3.056**	0	0	0	0	0	0	1	0	1	0	0	0	1	0	0	1	0	0	0	0	0	0	0	1	0	1
**3.057**	0	0	0	0	0	0	1	0	1	0	0	1	0	1	0	1	0	0	0	0	0	0	0	1	1	1
**3.058**	0	0	0	0	0	0	1	0	1	1	0	0	0	1	0	0	0	0	0	0	0	1	0	1	0	1
**3.059**	0	0	0	0	1	0	0	0	1	0	0	1	0	1	0	1	0	0	0	0	0	0	0	1	1	1
**3.060**	0	0	0	0	1	0	1	0	1	0	0	0	0	0	0	0	0	0	0	0	0	0	0	1	0	1
**3.061**	0	0	0	1	0	0	0	0	0	1	0	0	0	1	0	0	0	0	0	0	0	0	0	1	1	1
**3.062**	0	0	0	1	0	0	0	0	1	0	0	0	1	0	0	0	0	0	0	0	0	0	0	1	0	1
**3.063**	0	0	0	1	0	0	0	0	1	1	1	1	0	0	0	0	0	0	0	0	0	0	0	1	1	1
**3.064**	0	0	0	1	0	0	1	0	0	1	0	1	0	1	0	0	0	0	0	1	0	1	1	1	1	1
**3.065**	0	0	0	1	0	0	1	0	0	1	1	1	1	1	0	0	0	0	0	0	0	0	0	1	1	1
**3.066**	0	0	1	0	0	0	0	0	0	0	0	1	1	1	0	0	0	0	0	1	0	1	0	1	0	1
**3.067**	0	0	1	0	0	0	0	0	0	0	1	1	0	1	0	0	0	0	0	1	0	0	0	1	1	1
**3.068**	0	0	1	0	0	0	0	0	0	1	0	0	0	1	0	0	0	0	0	1	0	0	1	1	1	1
**3.069**	0	0	1	0	0	0	1	0	0	0	1	0	0	1	0	0	0	0	0	0	0	1	0	1	0	1
**3.070**	0	0	1	0	0	0	1	0	0	0	1	1	0	1	0	0	0	0	0	0	0	1	0	1	0	1
**3.071**	0	0	1	0	0	0	1	0	1	0	0	0	0	1	0	0	0	0	0	0	0	0	0	1	0	1
**3.072**	0	0	1	0	0	0	1	0	1	0	0	1	0	1	0	0	0	0	0	0	0	1	1	1	0	1
**3.073**	0	0	1	0	0	0	1	0	1	1	0	1	0	1	0	0	0	0	0	1	0	1	1	1	0	1
**3.074**	0	0	1	0	1	0	0	0	1	0	0	0	0	0	0	0	0	0	0	0	0	0	0	1	0	1
**3.075**	0	0	1	0	1	0	0	0	1	0	0	0	1	0	0	0	0	0	0	0	0	0	0	1	0	1
**3.076**	0	0	1	0	1	0	1	0	1	0	0	0	1	0	0	0	0	0	0	0	0	0	0	1	0	1
**3.077**	0	0	1	1	0	0	0	0	0	1	0	1	0	1	0	1	0	0	0	1	0	1	0	1	1	1
**3.078**	0	0	1	1	0	0	1	0	0	1	0	1	0	1	0	0	0	0	0	0	0	0	0	1	0	1
**3.079**	0	0	1	1	0	0	1	0	0	1	0	1	1	1	0	0	0	0	0	1	0	0	1	1	1	1
**3.080**	0	0	1	1	0	0	1	0	1	1	1	1	0	1	0	1	0	0	0	0	0	0	0	1	1	1
**3.081**	0	1	0	0	0	0	0	0	0	0	1	0	0	1	0	0	0	0	0	0	0	0	0	1	0	1
**3.082**	0	1	0	0	0	0	0	0	1	0	0	1	1	0	0	0	0	0	0	0	0	0	0	1	0	1
**3.083**	0	1	0	0	0	0	0	0	1	0	0	1	1	0	0	0	0	0	0	0	0	0	1	1	0	1
**3.084**	0	1	0	0	0	0	0	0	1	1	0	0	0	0	0	0	0	0	0	0	0	0	1	1	0	1
**3.085**	0	1	0	0	1	0	0	0	0	0	0	1	0	1	0	0	0	0	0	0	0	0	0	1	0	1
**3.086**	0	1	0	0	1	0	0	0	0	0	0	1	1	1	0	0	0	0	0	0	0	0	0	1	0	1
**3.087**	0	1	0	0	1	0	0	0	0	0	1	1	0	0	0	0	0	0	0	0	0	1	0	1	1	1
**3.088**	0	1	0	0	1	0	0	0	0	1	1	1	1	0	0	0	0	0	0	0	0	0	1	1	1	1
**3.089**	0	1	0	0	1	0	0	0	1	0	0	0	0	0	0	0	0	0	0	1	0	0	1	1	1	1
**3.090**	0	1	0	0	1	0	0	0	1	1	0	0	1	0	0	0	0	0	0	1	0	0	1	1	1	1
**3.091**	0	1	0	1	0	0	0	0	1	1	0	0	0	0	0	0	0	0	0	0	0	0	1	1	0	1
**3.092**	0	1	0	1	1	0	0	0	0	1	0	1	0	0	0	0	0	0	0	0	0	0	1	1	1	1
**3.093**	0	1	1	0	0	0	0	0	1	0	0	1	0	0	0	1	0	0	0	0	0	0	0	1	0	1
**3.094**	0	1	1	0	1	0	0	0	1	0	0	0	1	0	0	0	0	0	0	0	0	0	0	1	0	1
**3.095**	0	1	1	0	1	0	0	0	1	0	0	0	1	0	0	0	0	0	0	1	0	0	1	1	0	1
**3.096**	0	1	1	1	0	0	0	0	1	1	0	1	0	1	0	0	0	0	0	0	0	0	0	1	0	1
**3.097**	0	0	0	0	0	0	0	0	0	0	0	1	0	1	0	1	0	0	0	0	0	1	0	1	0	1
**3.098**	0	0	0	0	0	0	0	0	0	0	1	0	0	1	0	1	0	0	0	0	0	1	0	1	0	1
**3.099**	0	0	0	0	0	0	0	0	0	0	1	1	0	0	0	1	0	0	0	1	0	0	0	1	1	1
**3.100**	0	0	0	0	0	0	0	0	0	1	0	1	0	1	0	1	0	0	0	1	0	1	0	1	0	1
**3.101**	0	0	0	0	0	0	0	0	0	1	1	0	0	1	0	0	0	0	0	0	0	1	0	1	1	1
**3.102**	0	0	0	0	0	0	0	0	1	0	1	0	0	0	0	0	0	0	0	0	0	0	0	1	0	1
**3.103**	0	0	0	0	0	0	0	0	1	1	0	0	0	1	0	0	0	0	0	0	0	1	0	1	0	1
**3.104**	0	0	0	0	0	0	0	0	1	1	0	1	1	1	0	0	0	0	0	1	0	1	0	1	1	1
**3.105**	0	0	0	0	0	0	1	0	0	1	0	0	0	1	0	0	0	0	0	1	0	0	0	1	0	1
**3.106**	0	0	0	0	1	0	0	0	0	1	1	1	0	0	0	0	0	0	0	0	0	0	1	1	1	1
**3.107**	0	0	1	0	0	0	0	0	0	0	0	1	0	1	0	0	0	0	0	1	0	1	0	1	0	1
**3.108**	0	0	1	0	0	0	0	0	0	0	1	1	0	0	0	0	0	0	0	1	0	0	0	1	1	1
**3.109**	0	0	1	0	0	0	0	0	1	1	0	0	1	1	0	0	0	0	0	0	0	0	0	1	1	1
**3.110**	0	0	1	0	1	0	1	0	1	0	0	0	1	0	0	0	0	0	0	1	0	0	0	1	0	1
**3.111**	0	0	1	1	0	0	0	0	0	0	0	0	1	0	0	1	0	0	0	0	0	0	1	1	0	1
**3.112**	0	0	1	1	0	0	0	0	1	1	0	1	0	1	0	0	0	0	0	1	0	1	0	1	1	1
**3.113**	0	1	0	0	0	0	0	0	0	0	1	0	0	0	0	0	0	0	0	0	0	0	0	1	0	1
**3.114**	0	1	0	0	0	0	0	0	0	0	1	0	0	0	0	0	0	0	0	0	0	1	0	1	0	1
**3.115**	0	1	0	0	0	0	0	0	1	1	0	0	0	0	0	0	0	0	0	1	0	0	1	1	0	1
**3.116**	0	1	0	0	1	0	0	0	0	0	0	1	0	0	0	1	0	0	0	0	0	0	0	1	0	1
**3.117**	0	1	0	0	1	0	0	0	0	1	1	1	0	1	0	0	0	0	0	0	0	1	1	1	1	1
**3.118**	0	1	0	0	1	0	0	0	1	0	0	0	0	0	0	0	0	0	0	1	0	0	1	1	0	1
**3.119**	0	1	0	0	1	0	0	0	1	1	0	0	0	0	0	0	0	0	0	1	0	0	1	1	0	1
**3.120**	0	1	0	0	1	0	0	0	1	1	0	0	1	0	0	0	0	0	0	1	0	0	1	1	0	1
**3.121**	0	1	0	1	1	0	0	0	1	0	0	0	0	0	0	0	0	0	0	1	0	0	1	1	0	1
**3.122**	0	1	1	0	0	0	0	0	0	0	0	1	0	0	0	0	0	0	0	0	0	1	0	1	0	1
**3.123**	0	1	1	0	0	0	0	0	1	1	0	1	0	0	0	0	0	0	0	1	0	0	0	1	0	1
**3.124**	0	1	1	0	1	0	0	0	1	1	0	0	1	0	0	0	0	0	0	1	0	0	1	1	0	1
**3.125**	0	1	1	1	0	0	0	0	1	1	0	1	1	0	0	0	0	0	0	1	0	0	0	1	0	1
**3.126**	0	0	0	0	0	0	0	0	0	0	0	1	0	1	0	1	0	0	0	0	0	0	0	1	0	1
**3.127**	0	0	0	0	0	0	0	0	0	1	0	1	0	1	0	1	0	0	0	0	0	0	1	1	1	1
**3.128**	0	0	0	0	0	0	1	0	0	1	0	0	0	1	0	0	0	0	0	1	0	1	0	1	0	1
**3.129**	0	0	0	0	0	0	1	0	0	1	1	1	0	0	0	0	0	0	0	0	0	0	0	1	0	1
**3.130**	0	0	0	1	0	0	0	0	0	1	0	1	0	1	0	0	0	0	0	0	0	1	0	1	0	1
**3.131**	0	0	0	1	0	0	0	0	0	1	0	1	0	1	0	0	0	0	0	0	0	1	0	1	1	1
**3.132**	0	0	0	1	0	0	0	0	1	1	0	1	0	1	0	0	0	0	0	0	0	0	0	1	1	1
**3.133**	0	0	0	1	0	0	1	0	1	1	1	1	0	1	0	1	0	0	0	1	0	0	0	1	1	1
**3.134**	0	0	1	0	0	0	0	0	0	0	1	1	0	0	0	0	0	0	0	0	0	0	0	1	0	1
**3.135**	0	0	1	0	0	0	0	0	0	1	0	1	1	1	0	1	0	0	0	0	0	1	0	1	1	1

	CD2	CD3	CD4	CD7	CD8	CD10	CD13	CD18	CD26	CD29	CD36	CD44	CD45RA	CD49f	CD54	CD56	CD57	CD58	CD62L	CD71	CD80	Cytokeratin	Histone_H1	HLA-DQ	HLA-DR	Freq. (Voxel)
**3.136**	0	0	1	0	0	0	0	0	0	1	1	1	0	0	0	0	0	0	0	0	0	0	0	1	0	1
**3.137**	0	0	1	0	0	0	1	0	0	0	0	0	1	1	0	0	0	0	0	1	0	0	0	1	1	1
**3.138**	0	0	1	1	0	0	0	0	0	0	0	0	0	0	0	0	0	0	0	0	0	0	1	1	0	1
**3.139**	0	0	1	1	0	0	1	0	0	1	1	1	0	1	0	1	0	0	0	0	0	0	0	1	1	1
**3.140**	0	1	0	0	0	0	0	0	0	1	1	1	0	1	0	0	0	0	0	0	0	0	0	1	0	1
**3.141**	0	1	0	0	0	0	0	0	0	1	1	1	0	1	0	0	0	0	0	0	0	0	1	1	1	1
**3.142**	0	1	0	0	0	0	0	0	1	1	0	1	0	0	0	1	0	0	0	0	0	0	0	1	0	1
**3.143**	0	1	0	1	0	0	0	0	0	1	0	0	0	0	0	0	0	0	0	0	0	0	0	1	0	1
**3.144**	0	1	0	1	0	0	0	0	1	1	0	0	1	0	0	0	0	0	0	0	0	0	0	1	0	1
**3.145**	0	1	1	0	1	0	0	0	1	0	0	0	0	0	0	0	0	0	0	1	0	0	1	1	0	1
**3.146**	0	1	1	1	0	0	0	0	0	0	0	0	0	0	0	0	0	0	0	0	0	0	0	1	0	1
**3.147**	0	1	1	1	0	0	0	0	0	1	0	0	1	0	0	0	0	0	0	0	0	0	0	1	0	1
**3.148**	0	1	1	1	0	0	0	0	1	1	0	0	1	0	0	0	0	0	0	0	0	0	0	1	0	1
**3.149**	0	1	1	1	1	0	0	0	1	0	0	0	0	0	0	0	0	0	0	1	0	0	1	1	0	1
**3.150**	0	0	0	0	0	0	0	0	1	1	0	0	0	0	0	0	0	0	0	0	0	0	1	1	1	1
**3.151**	0	0	0	0	0	0	0	0	1	1	0	1	0	1	0	0	0	0	0	1	0	0	1	1	0	1
**3.152**	0	0	0	0	0	0	1	0	0	1	0	0	0	1	0	0	0	0	0	0	0	1	0	1	1	1
**3.153**	0	0	0	0	0	0	1	0	0	1	0	0	0	1	0	0	0	0	0	1	0	0	1	1	1	1
**3.154**	0	0	0	1	0	0	0	0	0	1	1	1	0	1	0	0	0	0	0	0	0	0	0	1	0	1
**3.155**	0	0	0	1	0	0	0	0	1	1	1	0	0	1	0	0	0	0	0	0	0	0	0	1	1	1
**3.156**	0	0	0	1	0	0	1	0	0	1	1	0	0	1	0	0	0	0	0	0	0	0	0	1	1	1
**3.157**	0	0	0	1	0	0	1	0	1	1	0	0	0	1	0	0	0	0	0	1	0	0	0	1	1	1
**3.158**	0	0	0	1	0	0	1	0	1	1	1	0	0	1	0	0	0	0	0	0	0	0	0	1	1	1
**3.159**	0	0	1	0	0	0	0	0	0	1	0	1	0	1	0	0	0	0	0	0	0	0	1	1	1	1
**3.160**	0	0	1	0	0	0	0	0	1	0	0	1	0	0	0	1	0	0	0	1	0	0	0	1	1	1
**3.161**	0	0	1	0	0	0	0	0	1	1	1	1	0	1	0	0	0	0	0	0	0	0	0	1	1	1
**3.162**	0	0	1	0	0	0	1	0	0	0	1	0	0	0	0	0	0	0	0	1	0	0	0	1	1	1
**3.163**	0	0	1	1	0	0	1	0	1	1	1	1	0	1	0	1	0	0	0	1	0	0	0	1	1	1
**3.164**	0	1	0	0	0	0	0	0	0	1	0	0	0	0	0	0	0	0	0	0	0	0	0	1	0	1
**3.165**	0	1	0	0	0	0	0	0	0	1	0	1	0	0	0	1	0	0	0	0	0	0	0	1	0	1
**3.166**	0	1	0	0	0	0	0	0	0	1	1	1	0	0	0	0	0	0	0	0	0	0	1	1	0	1
**3.167**	0	1	0	0	0	0	0	0	1	0	0	0	0	0	0	0	0	0	0	0	0	1	0	1	1	1
**3.168**	0	0	0	0	0	0	1	0	0	1	0	0	0	1	0	0	0	0	0	1	0	0	1	1	0	1
**3.169**	0	0	0	0	0	0	1	0	0	1	0	1	1	1	0	0	0	0	0	0	0	1	0	1	1	1
**3.170**	0	0	0	0	0	0	1	0	0	1	1	0	0	1	0	0	0	0	0	0	0	0	0	1	1	1
**3.171**	0	0	0	0	0	0	1	0	1	1	0	0	0	1	0	1	0	0	0	0	0	0	0	1	1	1
**3.172**	0	0	0	0	1	0	0	0	0	1	1	1	0	1	0	0	0	0	0	0	0	0	1	1	1	1
**3.173**	0	0	1	0	0	0	0	0	0	0	0	1	0	0	0	1	0	0	0	1	0	0	1	1	1	1
**3.174**	0	0	1	0	0	0	0	0	1	1	1	1	0	1	0	0	0	0	0	1	0	0	0	1	1	1
**3.175**	0	0	1	0	0	0	1	0	0	1	1	1	0	1	0	1	0	0	0	0	0	0	0	1	0	1
**3.176**	0	0	1	0	0	0	1	0	0	1	1	1	1	1	0	1	0	0	0	0	0	0	0	1	1	1
**3.177**	0	0	1	0	0	0	1	0	1	1	1	0	0	1	0	0	0	0	0	0	0	0	0	1	1	1
**3.178**	0	0	1	1	0	0	1	0	0	1	0	0	0	1	0	1	0	0	0	1	0	0	0	1	1	1
**3.179**	0	0	1	1	0	0	1	0	0	1	0	0	1	1	0	1	0	0	0	1	0	0	0	1	1	1
**3.180**	0	1	0	1	1	0	0	0	0	1	0	1	0	0	0	0	0	0	0	0	0	0	1	1	0	1
**3.181**	0	0	0	0	0	0	0	0	0	0	0	0	0	1	0	1	0	0	0	0	0	0	1	1	1	1
**3.182**	0	0	0	0	0	0	0	0	0	1	0	0	0	1	0	1	0	0	0	0	0	0	1	1	1	1
**3.183**	0	0	0	0	0	0	0	0	1	1	0	0	0	1	0	1	0	0	0	0	0	0	0	1	1	1
**3.184**	0	0	0	0	0	0	0	0	1	1	0	1	0	1	0	1	0	0	0	0	0	0	0	1	1	1
**3.185**	0	0	0	0	0	0	0	0	1	1	1	0	0	0	0	0	0	0	0	0	0	0	0	1	1	1
**3.186**	0	0	0	0	0	0	1	0	0	1	0	0	1	0	0	1	0	0	0	1	0	0	0	1	0	1
**3.187**	0	0	1	0	0	0	0	0	0	1	0	1	0	1	0	0	0	0	0	1	0	0	1	1	0	1
**3.188**	0	0	1	0	0	0	0	0	0	1	0	1	0	1	0	1	0	0	0	1	0	0	0	1	1	1
**3.189**	0	0	1	0	0	0	0	0	0	1	0	1	0	1	0	1	0	0	0	1	0	1	1	1	0	1
**3.190**	0	0	1	0	0	0	0	0	1	0	0	0	0	1	0	0	0	0	0	0	0	0	1	1	1	1
**3.191**	0	0	1	0	0	0	0	0	1	1	0	1	0	1	0	0	0	0	0	0	0	1	1	1	0	1
**3.192**	0	0	1	0	0	0	0	0	1	1	0	1	0	1	0	0	0	0	0	1	0	1	1	1	0	1
**3.193**	0	0	1	1	0	0	0	0	0	0	1	1	0	0	0	0	0	0	0	0	0	0	0	1	0	1
**3.194**	0	0	1	1	0	0	0	0	0	1	0	1	1	1	0	1	0	0	0	1	0	0	0	1	1	1
**3.195**	0	0	1	1	0	0	0	0	0	1	1	0	0	1	0	0	0	0	0	0	0	0	0	1	1	1
**3.196**	0	1	1	0	0	0	0	0	0	0	0	1	0	0	0	0	0	0	0	0	0	0	1	1	0	1
**3.197**	0	0	1	0	0	0	0	0	0	1	0	1	1	1	0	0	0	0	0	1	0	0	0	1	1	1
**3.198**	0	0	0	0	0	0	0	0	0	1	1	0	0	0	0	0	0	0	0	0	0	1	0	1	1	1
**3.199**	0	0	0	0	0	0	0	0	0	1	1	0	0	1	0	0	0	0	0	0	0	0	0	1	0	1
**3.200**	0	0	0	0	1	0	0	0	0	1	1	1	0	0	0	0	0	0	0	0	0	0	0	1	1	1
**3.201**	0	0	0	1	0	0	0	0	1	0	0	0	1	0	0	1	0	0	0	1	0	1	0	1	0	1
**3.202**	0	0	1	0	0	0	0	0	0	0	1	0	0	1	0	0	0	0	0	0	0	0	0	1	1	1
**3.203**	0	1	0	0	0	0	0	0	1	1	0	0	0	0	0	0	0	0	0	0	0	0	0	1	0	1
**3.204**	0	1	0	1	0	0	0	0	1	0	0	0	0	0	0	0	0	0	0	0	0	0	0	1	0	1
**3.205**	0	1	0	1	0	0	0	0	1	0	0	0	0	0	0	0	0	0	0	1	0	0	0	1	0	1
**3.206**	0	0	0	0	0	0	0	0	0	1	0	0	1	0	0	0	0	0	0	1	0	0	0	1	1	1
**3.207**	0	0	0	0	0	0	1	0	0	1	0	0	1	0	0	0	0	0	0	1	0	0	0	1	1	1
**3.208**	0	0	0	1	0	0	0	0	0	0	0	0	0	0	0	1	0	0	0	1	0	1	0	1	0	1
**3.209**	0	1	0	0	0	0	0	0	1	0	0	0	0	0	0	0	0	0	0	1	0	0	0	1	0	1
**3.210**	0	0	0	0	0	0	1	0	1	0	0	0	0	0	0	1	0	0	0	1	0	1	0	1	0	1
**3.211**	0	0	0	0	1	0	0	0	0	0	1	1	0	0	0	0	0	0	0	0	0	0	0	1	0	1
**3.212**	0	1	1	0	0	0	0	0	0	0	0	0	0	0	0	0	0	0	0	0	0	1	0	1	0	1

Supplementary Table 3. Complete sublist of 3,213 3D-CMPs sharing HLA-DQ as lead protein corresponding to CMP Motif 1 expressed selectively in the SPIKE structures (compare Figure 1 and Figure 3). The CMPs are sorted according to frequency (numbers of voxels expressing the respective CMP).
